# *Pseudomonas putida* KT2440 is HV1 certified, not GRAS

**DOI:** 10.1111/1751-7915.13443

**Published:** 2019-06-14

**Authors:** Linde F. C. Kampers, Rita J. M. Volkers, Vitor A. P. Martins dos Santos

**Affiliations:** ^1^ Laboratory of Systems and Synthetic Biology Wageningen University and Research Centre Stippeneng 4 6708 Wageningen The Netherlands; ^2^ Lifeglimmer GmbH Markelstr. 38 12163 Berlin Germany

## Abstract

*Pseudomonas putida* is rapidly becoming a workhorse for industrial production due to its metabolic versatility, genetic accessibility and stress‐resistance properties. The *P. putida* strain KT2440 is often described as Generally Regarded as Safe, or GRAS, indicating the strain is safe to use as food additive. This description is incorrect. *P. putida* KT2440 is classified by the FDA as HV1 certified, indicating it is safe to use in a P1 or ML1 environment.

## Abstract 2

KT2440 is referred to as GRAS. Careful study of the FDA report shows instead it is HV1 certified. It appears that this is caused by incorrect transitive referencing.

After the discovery and full sequencing of the bacterium thus far known as *Pseudomonas putida* KT2440 (Bagdasarian *et al*., 1981; Nelson *et al*., 2002; Timmis, 2002), this strain has rapidly become a workhorse for industrial biotechnology owing to its metabolic versatility, genetic accessibility and stress‐resistance properties (Puchałka *et al*., 2008; Poblete‐Castro *et al*., 2012; Nikel and de Lorenzo, 2018). This specific set of characteristics are made more accessible by the recent expansion of its metabolic toolkit, including specific inducible promoters (Cook *et al*., 2018) and introduction of the CRISPR‐system (Mougiakos *et al*., 2017; Aparicio *et al*., 2018; Sun *et al*., 2018). Its many benefits as a species have launched *P. putida* KT2440 as a model organism for industrial production of biobased chemicals such as citrulline (Patil *et al*., 2017), muconate (Johnson *et al*., 2017), precursors of biobased plastics (Koopman *et al*., 2010), precursor of biodegradable plastics (Ribera *et al*., 2001; Poblete‐Castro *et al*., 2013) and waste biodegradation purposes (Wierckx *et al*., 2015; Ravi *et al*., 2017).

Often *P. putida* KT2440, and sometimes the *P. putida* species, is described as Generally Regarded as Safe, or GRAS, indicating it is safe to use as a food additive (Nutrition, 2018) (e.g. (Aparicio *et al*., 2018; Arnfinnsdottir *et al*., 2016; Belda *et al*., 2016; Bojanovic, 2016; Calero *et al*., 2016; Cesarini *et al*., 2014; Choi *et al*., 2018; Classen and Pietruszka, 2018; Cook *et al*., 2018; Cornelissen *et al*., 2011; Cuenca *et al*., 2016; Domröse *et al*., 2015; Dvorak and de Lorenzo, 2018; Escapa *et al*., 2011; Fröhlich *et al*., 2014; Gemperlein *et al*., 2014; Gómez *et al*., 2016; Gong *et al*., 2017; de las Heras and de Lorenzo, 2011; Jayakody *et al*., 2018; Kahlon, 2016; Kim *et al*., 2019; Klein *et al*., 2017; Köppen *et al*., 2014; Kusumawardhani *et al*., 2018; Lee and Wendisch, 2017; Lieder *et al*., 2015; Loeschcke and Thies, 2015; Martínez *et al*., 2014; Mindt *et al*., 2018; Nikel and de Lorenzo, 2018; Pandi *et al*., 2017; Pernicova *et al*., 2019; Poblete‐Castro *et al*., 2016; Puchałka *et al*., 2008; Ravi *et al*., 2017; Rühl *et al*., 2012; Sun *et al*., 2018; Taghavi *et al*., 2011; Troeschel *et al*., 2012; Wang *et al*., 2014; Weyens *et al*., 2009; Wierckx *et al*., 2015; Wu *et al*., 2011)).

When a reference is given by the authors for this GRAS claim, the following is used: the US Food and Drug Regulation Administration (FDA) report, specifically the FDA vol. 47, no. 77, Appendix E, page 17197, Certified host–vector systems from 21st April 1982 (see Appendix S1) (National Archives And Records Administration, 1982). However, *P. putida* KT2440 is herein not certified as GRAS, but as host–vector (HV) system safety level 1: ‘HV1 – Pseudomonas putida strain KT2440 with plasmid vectors pKT262, pKT263 and pKT264’. The HV1 certification indicates that *P. putida* KT2440 is determined to be safe to work with at a P1 (or ML1) facility, as elaborately described by the FDA on page 17181 of the report (National Archives and Records Administration, 1982). Similarly to *E. coli* K12, no additional safety measures are required to work with this strain.

The GRAS status determined by the FDA is only awarded to chemicals or specific strains of microorganisms that are proven to be safe for ingestion (Nutrition, C. for F.S. and A., 2018). This is not proven for *P. putida* KT2440, contrary to food‐related microorganisms such as *Lactobacillus helveticus* R0052, *Lactobacillus casei* subsp. *paracasei* Lpc‐37, *Bifidobacterium bifidum* R0071 or *Streptococcus salivarius* K12, which are examples of strains that carry the GRAS status. Intense literature research suggests the GRAS reference occurs due to incorrect transitive referencing. A full GRAS certificate list can be found at the FDA website: https://www.accessdata.fda.gov/scripts/fdcc/?set=GRASNotices.

However, this does not mean that *P. putida* KT2440 is unsafe to work with. The safety of the strain is proven by the overall absence of virulence factors (Dos Santos *et al*., 2004; Belda *et al*., 2016) and the intensive research over the past 40 years by a large community without any fortuitous cases of infection reported. This correct certification does therefore not change the many useful applications of this strain. Furthermore, the widespread use of KT2440, the massive evidence of safe performance and the development of more refined methods for risk assessment than those available in the early 80s ask for a revised classification of the strains at stake under the current FDA and EFSA regulations.

As a note, the phylogenetic classification of *P. putida* KT2440 within the *P. putida* group has been questioned for long (Regenhardt *et al*., 2002), and recent work has provided a new classification (Keshavarz‐Tohid *et al*., 2019) (Moore *et al*., in preparation). However, for the purpose of safety certification, the specific bacterium formerly known as *P. putida* KT2440 remains classified as HV1, regardless of its precise phylogenetic status.

We urge other scientists to properly describe the safety status of all used strains, since we also discovered the same is true for example *Corynebacterium glutamicum*. Similarly to *P. putida* KT2440, *C. glutamicum* is sometimes reported to have GRAS status (e.g. (Baritugo *et al*., 2018; Kim *et al*., 2018; Levin, 2002; Neuner *et al*., 2013)), but when checked using the website stated above, only cultured corn starch fermented by *C. glutamicum* is GRAS, not the organism itself.

## Conflict of interest

None declared.

## Author contributions

LFCK conceived the subject. LFCK/RJMV involved in literature search and analysis. LFCK wrote the manuscript. RJMV/VAPMdS supervised the work. VAPMdS arranged the funding.

## Supporting information


**Appendix S1**. FDA report vol. 47, no. 77, Certified host–vector systems from 21st April 1982. Includes Appendix E, page 17197.
