# Supplementary material for: Pseudomonas putida KT2440 is HV1 certified, not GRAS
Source: Microb Biotechnol. 2019 Jun 14;12(5):845–8. doi: 10.1111/1751-7915.13443 (PMC6680625; doi:10.1111/1751-7915.13443)
Supplement: Supplementary file 1 — Appendix S1. FDA report vol. 47, no. 77, Certified host–vector systems from 21st April 1982. Includes Appendix E, page 17197. [file MBT2-12-845-s001.pdf]

4-21-82
Vol. 47 No. 77
Pages 17033-17270

federal register

Wednesday
April 21, 1982

FEDERAL REGISTER Published daily, Monday through Friday, (not published on Saturdays, Sundays, or on official holidays), by the Office of the Federal Register, National Archives and Records Service, General Services Administration, Washington, D.C. 20408, under the Federal Register Act (49 Stat. 500, as amended; 44 U.S.C. Ch. 15) and the regulations of the Administrative Committee of the Federal Register (1 CFR Ch. I). Distribution is made only by the Superintendent of Documents, U.S. Government Printing Office, Washington, D.C. 20402.

The **Federal Register** provides a uniform system for making available to the public regulations and legal notices issued by Federal agencies. These include Presidential proclamations and Executive Orders and Federal agency documents having general applicability and legal effect, documents required to be published by Act of Congress and other Federal agency documents of public interest. Documents are on file for public inspection in the Office of the Federal Register the day before they are published, unless earlier filing is requested by the issuing agency.

The **Federal Register** will be furnished by mail to subscribers, free of postage, for $300.00 per year, or $150.00 for six months, payable in advance. The charge for individual copies is $1.50 for each issue, or $1.50 for each group of pages as actually bound. Remit check or money order, made payable to the Superintendent of Documents, U.S. Government Printing Office, Washington, D.C. 20402.

There are no restrictions on the republication of material appearing in the **Federal Register.**

Questions and requests for specific information may be directed to the telephone numbers listed under INFORMATION AND ASSISTANCE in the READER AIDS section of this issue.

Contents

Federal Register
Vol. 47, No. 77
Wednesday, April 21, 1982

Agricultural Marketing Service
RULES
17034 Hops of domestic production; emergency
Milk marketing areas:
17036 Lake Mead; suspension
17035 Oklahoma Metropolitan; suspension

Agriculture Department
See Agricultural Marketing Service; Animal and Plant Health Inspection Service; Federal Crop Insurance Corporation.

Army Department
NOTICES
Meetings:
17108 Medical Research and Development Advisory Committee
17108 Military personal property symposium

Animal and Plant Health Inspection Service
PROPOSED RULES
17068 Animals, purebred; recognized breeds and books of record; listing for Russian Arabian Stud Book of Moscow

Arts and Humanities, National Foundation
NOTICES
Meetings:
17136 Humanities Advisory Panel
17136 Music Advisory Panel

Civil Aeronautics Board
NOTICES
Hearings, etc.:
17099 Avia International Airlines, Inc., et al.
17099 Flying Tiger Line Inc. et al.

Commerce Department
See International Trade Administration; Minority Business Development Agency; National Bureau of Standards; National Oceanic and Atmospheric Administration.

Commodity Futures Trading Commission
NOTICES
17156 Meetings; Sunshine Act (2 documents)

Comptroller of Currency
PROPOSED RULES
National banks:
17069 Accrual accounting

Customs Service
PROPOSED RULES
Customs relations with Canada and Mexico:
17072 Vehicles or vessels of less than five net tons arriving otherwise than by sea from Canada or Mexico; Customs Form 7533 elimination and standardized form development

Defense Department
See Army Department.

Economic Regulatory Administration
RULES
Powerplant and industrial fuel use:
17037 Existing electric powerplants; involuntary conversion to coal or alternate fuel; elimination of prohbition orders, etc.

Education Department
RULES
Elementary and secondary education:
17246 Migrant education program activities; grants to State educational agencies to improve interstate and intrastate coordination
Postsecondary education:
17252 Cooperative education program
17200 Parent loans for undergraduate students (PLUS) program
NOTICES
Grant applications and proposals; closing dates:
17109 Cooperative education program; administration, demonstration, research, and training projects
17108 School construction program

Energy Department
See also Economic Regulatory Administration; Federal Energy Regulatory Commission.
NOTICES
International atomic energy agreements; civil uses; subsequent arrangements:
17111 European Atomic Energy Community
Meetings:
17111 Dose Assessment Advisory group
17112 High Energy Physics Advisory Panel
Patent licenses, exclusive:
17112 Atom Sciences, Inc.
17110 Uranium depleted in isotope U-235 (tails); sale pricing policy change; revision

Environmental Protection Agency
RULES
Hazardous waste programs; interim authorizations; various States:
17055 Connecticut
Pesticide chemicals in or on raw agricultural commodities; tolerances and exemptions, etc.:
17056 Cyano (3-phenoxyphenyl)methyl 4-chloro-alpha-(1-methylethyl)benzeneacetate
17057 Norflurazon
17058 Terbufos
Waste management, solid; State plans:
17059 Massachusetts
PROPOSED RULES
Pesticide chemicals in or on raw agricultural commodities; tolerances and exemptions, etc.:
17078 Potassium sorbate
NOTICES
Pesticide, food, and feed additive petitions:
17113 Diamond Shamrock Corp.
17112 ICI American, Inc., et al.
Pesticides; experimental use permit applications:
17114 Upjohn Co.; correction

Pesticides; temporary tolerances:
17113 Chlorpyrifos
17114 Dow Chemical Co.

Federal Communications Commission
RULES
Radio broadcasting:
17065 Broadcast construction permit applications; proof of publication of local notice submission requirement eliminated
PROPOSED RULES
Common carrier services:
17083 Second computer inquiry (telephone deregulation); detarifing of customer premises equipment and enhanced services; implementing procedures

Federal Crop Insurance Corporation
RULES
Crop insurance; various commodities:
17033 Wheat; interim rule and request for comments

Federal Deposit Insurance Corporation
PROPOSED RULES
Unsafe and unsound banking practices:
17264 Accrual accounting
NOTICES
17156, 17157 Meetings; Sunshine Act (3 documents)

Federal Emergency Management Agency
RULES
Flood insurance; special hazard areas; map corrections:
17061 California
17064 Florida
17062 Mississippi
17063 New Jersey
17063 South Carolina
17064 Texas
PROPOSED RULES
Flood elevation determinations:
17078 California
17079, 17080 Illinois (2 documents)
17081 Kansas
17081 Louisiana
17082 Nebraska
NOTICES
Disaster and emergency areas:
17115 Mississippi
17115 Ohio (2 documents)
17115 Texas

Federal Energy Regulatory Commission
RULES
Natural Gas Policy Act; ceiling prices for high cost natural gas produced from tight formations; various States:
17054 Colorado
PROPOSED RULES
Natural Gas Policy Act; ceiling prices for high cost natural gas produced from tight formations; various States:
17070 Wyoming

Federal Home Loan Mortgage Corporation
NOTICES
17157 Meetings; Sunshine Act

Fish and Wildlife Service
RULES
Public entry and use:
17066 Wichita Mountains Wildlife Refuge, Okla.; extension of time
NOTICES
17118, 17119 Endangered and threatened species permit applications (2 documents)

Geological Survey
PROPOSED RULES
Oil and gas operating regulations:
17076 Onshore Federal and Indian oil and gas leases; site security; advance notice

Government National Mortgage Association
RULES
17055 Attorney-in-fact list

Health and Human Services Department
See Health Services Administration; National Institutes of Health.

Health Services Administration
NOTICES
Grants; availability, etc.:
17116 Scholarships for first-year students of exceptional financial need program; medical, osteopathy and dental schools awards

Housing and Urban Development Department
See Government National Mortgage Association.

Indian Affairs Bureau
PROPOSED RULES
Enrollment:
17072 Preparation of roll of Pribilof Islands Aleut Communities of St. Paul and St. George

Interior Department
See Fish and Wildlife Service; Geological Survey; Indian Affairs Bureau; Land Management Bureau; National Park Service; Reclamation Bureau; Surface Mining Reclamation and Enforcement Office.

Internal Revenue Service
NOTICES
Authority delegations:
17153 Chief Counsel Office; technical and appeals functions transferred from Corporation Tax Division et al.
Meetings:
17153 Commissioner's Advisory Group

International Trade Administration
NOTICES
Committees; establishment, renewals, terminations, etc.:
17100 Trade Policy Matters Industry Policy Advisory Committee et al.
Meetings:
17099 President's Export Council
Scientific articles; duty free entry:
17101 Johns Hopkins University
17101 University of Louisville

Steel trigger price mechanism:
17102 Stainless steel wire imports, monitoring; correction
Trade adjustment assistance determination petitions:
17100 Century Industries, Inc., et al.

International Trade Commission
NOTICES
17135 Harmonized commodity description and coding system; inquiry
Import investigations:
17132 Audiovisual games and components, coin-operated (Rally-X and Pac Man)
17135 Doxycycline
17133 Methods for extruding plastic tubing
17133 Multi-sequential coded radio pagers
17134 Silica-coated lead chromate pigments
17136 Sodium nitrate from Chile
17134 Termal conductivity sensing gem testers and components

Interstate Commerce Commission
NOTICES
Motor carriers:
17129 Fuel surcharge program modification; owner-operator reimbursement rate decrease
17122, 17125 Permanent authority applications (2 documents)
17127 Temporary authority applications
Rail carriers:
17130 Missouri Pacific Railroad Co. et al.; contract tariff exemption
17130 Richmond, Fredericksburg & Potomac Railroad Co.; contract tariff exemption
Railroad operation, acquisition, construction, etc.:
17131 Southern Railway Co.
Railroad services abandonment:
17131, 17132 Consolidated Rail Corp. (4 documents)

Land Management Bureau
RULES
Public land orders:
17061 Alaska
17060 Oregon
NOTICES
Classification of public lands:
17117 Utah
Conveyance of lands:
17117 California (2 documents)
Environmental statements; availability, etc.:
17116 Tonopah Resource Area, Nev.; preliminary wilderness recommendations
Resource management plans:
17117 Glenwood Springs Resource Area, Colo.; workshop schedule

Minority Business Development Agency
NOTICES
Financial assistance application announcements:
17102 New York

National Bureau of Standards
NOTICES
Meetings:
17104 Carpet testing public workshop; national voluntary laboratory accreditation program

National Institutes of Health
NOTICES
Recombinant DNA molecules research:
17166 Actions under guidelines
17180 Guidelines

National Oceanic and Atmospheric Administration
PROPOSED RULES
Tuna, Atlantic fisheries:
17086 Bluefin tuna
NOTICES
Meetings:
17104 New England Fishery Management Council; date change

National Park Service
PROPOSED RULES
Special regulations:
17077 Olympic National Park, Wash.; snowmobile regulations
NOTICES
Environmental statements; availability, etc.:
17122 Grand Canyon National Park, Ariz.; adjacent lands study
17119 Grant Village, Yellowstone National Park, Wyo.
Meetings:
17119 Kalaupapa National Historical Park Advisory Commission
17120 World heritage properties list; U.S. nominations process; 1982 FY; inquiry

National Science Foundation
NOTICES
Meetings:
17137 Advisory Council

Nuclear Regulatory Commission
NOTICES
Applications, etc.:
17137 Consumers Power Co.
Environmental statements; availability, etc.:
17137 Commonwealth Edison Co.; Byron Station, Units 1 and 2

Pension Benefit Guaranty Corporation
NOTICES
Multiemployer pension plans; bond/escrow exemption requests, etc.:
17137 RGZ, Inc./Gulf Elevator & Transfer Co., Inc., et al.

Reclamation Bureau
NOTICES
Contract negotiations:
17118 Deaver Irrigation District, Shoshone Project, Wyo.

Securities and Exchange Commission
RULES
17046 Small business; system of classification of small issuers; reporting exemptions, etc.
NOTICES
Hearings, etc.:
17139 Allied Capital Corp. et al.
17144 Chancellor Cash Fund, Inc., et al.
17149 Clabir Corp.
17149 Cummins Engine Co., Inc.

17145 Gotham Funds
17150 Prudential Insurance Co. of America
Self-regulatory organizations; proposed rule changes:
17152 Boston Stock Exchange, Inc.
17147 Midwest Securities Trust Co.
17152 Pacific Stock Exchange, Inc. (2 documents)
17148 New York Stock Exchange, Inc.
Self-regulatory organizations; unlisted trading privileges:
17149 Boston Stock Exchange, Inc.

Surface Mining Reclamation and Enforcement Office
PROPOSED RULES
Permanent and interim regulatory programs:
17268 State regulatory authority inspection and enforcement, Federal inspection and monitoring, and approval procedures for program submission; partial deferral of requirements; advance notice
Permanent program submission; various States:
17240 Georgia

Textile Agreements Implementation Committee
NOTICES
Export visa requirements; certification, etc.:
17104 Korea

Treasury Department
See Comptroller of Currency; Customs Service; Internal Revenue Service.

Veterans Administration
NOTICES
Environmental statements; availability, etc.:
17155 Pittsburgh, Pa.; construction of new laundry

White House Fellowships, President's Commission
NOTICES
17139 Meetings

CFR PARTS AFFECTED IN THIS ISSUE

A cumulative list of the parts affected this month can be found in the Reader Aids section at the end of this issue.

3 CFR

Executive Orders:

December 12, 1917 (Revoked in part by PLO 6238) 17060

7 CFR

418 17033
991 17034
1106 17035
1139 17036

9 CFR

Proposed Rules:

151 17068

10 CFR

500 17037
501 17037
504 17037

12 CFR

Proposed Rules:

31 17069
337 17264

17 CFR

240 17046
249 17046

18 CFR

271 17054

Proposed Rules:

271 17070

19 CFR

Proposed Rules:

123 17072

24 CFR

300 17055

25 CFR

Proposed Rules:

43d 17072

30 CFR

Proposed Rules:

221 17076
732 17268
840 17268
843 17268
910 17240

34 CFR

205 17246
631 17252
632 17252
633 17252
634 17252
635 17252
683 17200

36 CFR

Proposed Rules:

7 17077

40 CFR

123 17055
180 (3 documents) 17056-17058
256 17059

Proposed Rules:

180 17078

43 CFR

Public Land Orders:

6238 17060
6239 17061

44 CFR

70 (6 documents) 17061-17064

Proposed Rules:

67 (6 documents) 17078-17082

47 CFR

73 17065

Proposed Rules:

Ch. I 17083

50 CFR

26 17066

Proposed Rules:

285 17086

Rules and Regulations

Federal Register
Vol. 47, No. 77
Wednesday, April 21, 1982

This section of the FEDERAL REGISTER contains regulatory documents having general applicability and legal effect, most of which are keyed to and codified in the Code of Federal Regulations, which is published under 50 titles pursuant to 44 U.S.C. 1510.
The Code of Federal Regulations is sold by the Superintendent of Documents. Prices of new books are listed in the first FEDERAL REGISTER issue of each month.

DEPARTMENT OF AGRICULTURE

Federal Crop Insurance Corporation

7 CFR Part 418

[Amdt. 1]

Wheat Crop Insurance Regulations

AGENCY: Federal Crop Insurance Corporation, USDA.

ACTION: Interim rule.

SUMMARY: The Federal Crop Insurance Corporation (FCIC) hereby amends the Wheat Crop Insurance Regulations (7 CFR Part 418), effective with the 1983 crop year by (1) adding a section 11 to the Appendix to the Policy to prescribe procedures in cases of loss by fire, (2) prescribing interest rates to be charged when premium payments are not made within a certain time, and (3) adding a provision to require the insured to file a notice of probable loss when the crop is damaged to the extent that a loss is probable and require that a representative sample of unharvested crop be left intact for a certain period of time. The intended effect of this amendment is to restore a provision in the regulations regarding losses from fire, improve the debt management practices of the Corporation, and revise the system of reporting damage or loss to insured crops to make administration more effective. This interim rule is promulgated under the authority contained in the Federal Crop Insurance Act, as amended.

DATES: Effective Date: April 21, 1982.

Comment Date: Written comments, data, and opinions on this proposed rule must be submitted by not later than June 21, 1982, to be sure of consideration.

ADDRESS: Written comments on this interim rule should be sent to the Office of the Chairman, Federal Crop Insurance Corporation, U.S. Department of Agriculture, Washington, D.C. 20250.

FOR FURTHER INFORMATION CONTACT: Peter F. Cole, Secretary, Federal Crop Insurance Corporation, U.S. Department of Agriculture, Washington, D.C. 20250, telephone 202-447-3325.

The Impact Statement describing the options considered in developing this interim rule and the impact of implementing each option is available upon request from Peter F. Cole.

SUPPLEMENTARY INFORMATION: Information collection requirements contained in the regulations to which this amendment applies (7 CFR Part 418) have been approved by the Office of Management and Budget (OMB) under the provisions of 44 U.S.C. Chapter 35 and have been assigned OMB #0563-0001, 0563-0003, and 0563-0007.

This action has been reviewed under USDA procedures established in Secretary's Memorandum No. 1512-1 (June 11, 1981).

Melvin E. Sims, Chairman, FCIC, has determined that an emergency situation exists which warrants immediate implementation of this rule without allowing for the normal 60-day comment period because the Wheat Crop Insurance Regulations (7 CFR Part 418) provide that any amendments to the regulations must be placed on file 15 days prior to the cancellation date in order to allow policyholders sufficient time to decide on their insurance plans.

The earliest cancellation date reflected in this rule is April 30; therefore, under the provisions of the Wheat Crop Insurance Regulations, any amendments must be placed on file by April 15 in order to be effective for the 1983 crop year. It has been determined that there would not be sufficient time to permit a comment period and still conform with the requirements of the regulations with respect to placing the amended regulations on file 15 days prior to the cancellation date.

Pursuant to the administrative provisions in 5 U.S.C. 553, it is found upon good cause that notice and other public procedure with respect to this interim rule are impracticable and contrary to the public interest.

However, comments are solicited for 60 days after the pubication of this document, and this interim action will be scheduled for review so that a final document discussing comments received and any amendments required can be published in the **Federal Register** as soon as possible.

Melvin E. Sims, Chairman, FCIC, has also determined that (1) this action is not a major rule as defined by Executive Order No. 12291 (February 17, 1981), (2) this action does not increase the Federal paperwork burden for individuals, small businesses, and other persons, and (3) this action conforms to the Federal Crop Insurance Act, as amended (7 U.S.C. 1501 *et seq.*), and other applicable law.

The title and number of the Federal Assistance Program to which this amendment applies are: Title—Crop Insurance; Number 10.450.

This action will not have a significant impact specifically on area and community development; therefore, review as established in OMB Circular A-95 was not used to assure that units of local government are informed of this action.

It has been determined that this action is exempt from the provisions of the Regulatory Flexibility Act; therefore, no Regulatory Impact Statement was prepared.

It has also been determined that this action does not constitute a review as to the need, currency, clarity, and effectiveness of these regulations under the provisions of Secretary's Memorandum No. 1512-1 (June 11, 1981). That review will be completed prior to the sunset review date of February 8, 1987.

In reviewing these regulations, it was determined that the following changes should be made:

1. Restore section 11 to the Appendix to the crop insurance Policy to provide that if the insured has other insurance against fire losses, the Corporation shall be liable for loss due to fire only for the smaller of the amount of indemnity determined on the contract with FCIC, or the amount by which the loss from fire exceeds the indemnity paid or payable under such other insurance.
2. Amend Section (d) of 7 CFR 418.7(d) Terms and Conditions to provide that, for the 1983 and succeeding crop years, unpaid premiums will bear interest in the amount of 1½ percent simple interest per month or part thereof, starting with the first day of the month following the month in which the acreage reporting date for the crop in the county occurred.
3. Add to paragraph 7 of 7 CFR 418.7(d) Terms and Conditions to

provide that if a crop is damaged to the extent that a loss is probable, the insured is required to give written notice of damage at least 15 days prior to the beginning of harvest. If a probable loss is not determined until less than 15 days prior to harvest, the insured is required to give such notice immediately and leave a representative sample of unharvested crop intact for 15 days after the date of the notice.

All written comments made pursuant to this notice will be available for public inspection in the Office of the Chairman during regular business hours, Monday through Friday.

List of Subjects in 7 CFR Part 418

Crop insurance, Wheat.

Interim Rule

PART 418—WHEAT CROP INSURANCE

Accordingly, pursuant to the authority contained in the Federal Crop Insurance Act, as amended (7 U.S.C. 1501 *et seq.*), the Federal Crop Insurance Corporation hereby amends the Wheat Crop Insurance Regulations (7 CFR Part 418), effective with the 1983 and succeeding crop years, in the following instances:

1. The authority for 7 CFR Part 418 is:

Authority: Secs. 506, 516, Pub. L. 75-430, 52 Stat. 72, as amended (7 U.S.C. 1506, 1516).

2. Paragraph 5(d) of the Terms and Conditions section of the Policy as found in 7 CFR 418.7(d) is amended to read as follows:

§ 418.7 The application and policy.

* * * * *

(d) * * *

Wheat Crop Insurance Policy

Terms and Conditions

* * * * *

5. *Annual Premium.*

* * * * *

(d) Interest will accrue at the rate of one and a half percent (1½%) simple interest per calendar month or any part thereof on any, unpaid premium balance following the month in which the acreage reporting date for the crop occurs (see Section 3): *Provided,* That such interest will not be charged if the full amount of the premium is received by the Corporation within 30 days from the date of the first premium billing.

3. Paragraph 7 of the Terms and Conditions section of the Policy as found in 7 CFR 418.7(d) is amended by redesignating subparagraphs (d) and (e) as subparagraphs (e) and (f) and by revising subparagraph (c) and adding a new subparagraph (d) to read as follows:

§ 418.7 The application and policy.

* * * * *

(d) * * *

Terms and Conditions

* * * * *

(c) Notice shall be given at least 15 days prior to the beginning of harvest if the wheat on any unit is damaged to the extent that a loss is probable. If probable loss is not determined until less than 15 days prior to the beginning of harvest on a unit, notice shall be given immediately and a representative sample of the unharvested wheat (at least 10 feet wide and the entire length of the field) shall remain intact for a period of 15 days from the date of the notice, unless the Corporation gives written consent to the insured to harvest the representative sample.

(d) In addition to the notices required in paragraphs (b) and (c) of this section, if a loss is to be claimed on any unit, the insured shall give written notice thereof to the Corporation at the office for the county not later than *30 days* after the earliest of (1) the date harvest is completed on the unit, (2) the calendar date for the end of the insurance period, or (3) the date the entire wheat crop on the unit is destroyed, as determined by the Corporation. The Corporation reserves the right to provide additional time if there are extenuating circumstances.

4. The Appendix to the Wheat Insurance Policy (Additional Terms and Conditions) as found in 7 CFR 418.7 is hereby amended by adding section 11 to read as follows:

§ 418.7 The application and policy.

* * * * *

(d) * * *

Wheat Crop Insurance Policy

* * * * *

Appendix to the Wheat Insurance Policy—(Additional Terms and Conditions)

* * * * *

11. Other Insurance Against Fire. If the insured has other insurance against damage by fire during the insurance period, the Corporation shall be liable for loss due to fire only for the smaller of (a) the amount of indemnity determined by the Corporation under the policy with the Corporation or (b) the amount by which the loss from fire exceeds the indemnity paid or payable under such other insurance. For the purposes of this section, the amount of loss from fire shall be the difference between the fair market value of the production on the unit before the fire and after the fire, as determined by the Corporation from appraisals made by the Corporation.

Approved by the Board of Directors on February 4, 1982.

Peter F. Cole,

Secretary, Federal Crop Insurance Corporation.

Dated: April 12, 1982.

Approved by:

Merritt W. Sprague,

Acting Chairman.

[FR Doc. 82-10884 Filed 4-20-82; 8:45 am]

BILLING CODE 3410-08-M

Agricultural Marketing Service

7 CFR Part 991

Hops of Domestic Production; Amendment of Administrative Rules and Regulations

AGENCY: Agricultural Marketing Service, USDA.

ACTION: Emergency final rule.

SUMMARY: (1) The April 1 deadline date for the transfer of base has passed; and (2) unless the date extension is made promptly, producers will be unable to transfer base under the provisions of the order and make their plans for growing 1982 crop hops. This action is based on the recommendation of the Hop Administrative Committee in accordance with provisions of Marketing Order No. 991, as amended (7 CFR Part 991), regulating the handling of hops of domestic production. This final rule extends the cut-off date to April 30 for producers to transfer all or part of their allotment bases to other producers under the marketing order for domestic hops. This change would apply only in calendar year 1982. This will enable growers to transfer base under the order and complete their growing plans for the 1982-83 marketing year.

EFFECTIVE DATE: April 21, 1982.

FOR FURTHER INFORMATION CONTACT: J. S. Miller, Chief, Specialty Crops Branch, Fruit and Vegetable Division, AMS, U.S. Department of Agriculture, Washington, D.C. 20250 (202) 447-5697.

SUPPLEMENTARY INFORMATION: This final rule has been reviewed under USDA guidelines implementing Executive Order 12291 and Secretary's Memorandum No. 1512-1 and has been classified a "non-major" rule under criteria contained therein.

William T. Manley, Acting Administrator, Agricultural Marketing Service, has determined that this action will not have a significant economic impact on a substantial number of small entities because it would result in only minimal costs being incurred by the regulated nine handlers.

It is found that it is impractical, unnecessary, and contrary to the public interest to give preliminary notice, engage in public rulemaking and delay the effective date of this action until 30 days after publication in the **Federal**

Register (5 U.S.C. 553) because: (1) The April 1 deadline date for the transfer of base has passed; and (2) unless the date extension is made promptly, producers will be unable to transfer base under the provisions of the order and make their plans for growing 1982 crop hops.

This action is based on the recommendation of the Hop Administrative Committee in accordance with provisions of Marketing Order No. 991, as amended (7 CFR Part 991), regulating the handling of hops of domestic production. The order is effective under the Agricultural Marketing Agreement Act of 1937, as amended (7 U.S.C. 601-674).

Section 991.146(c) of Subpart—Administrative Rules and Regulations (7 CFR 991.130-991.160; 46 FR 60177) currently provides that a producer can transfer all or part of his allotment base to another producer only if the transfer is effective prior to the issuance of an annual allotment to the transferor or prior to April 1, whichever is earlier. The late establishment of the salable quantity and allotment percentage for the 1982-83 marketing year makes it necessary to extend the April 1 cut-off date to April for the 1982 calendar year. This will enable growers to transfer base under the order and complete their growing plans for the 1982-83 marketing year.

After consideration of all relevant matter presented, including the information and recommendation submitted by the Committee, and other available information it is further found that to change the cut-off date for producers to transfer allotment bases to other producers in calendar year 1982 to April, as hereinafter set forth, will tend to effectuate the declared policy of the act.

List of Subjects in 7 CFR Part 991

Marketing Agreements and Orders, Hops.

PART 991—HOPS OF DOMESTIC PRODUCTION

Therefore, this final rule would revise § 991.146(c) of Subpart—Administrative Rules and Regulations (7 CFR 991.130-991.162; 46 FR 60177) to read as follows:

§ 991.146 Transfer of allotment base.

* * * * *

(c) Whenever a producer transfers all or part of his allotment base to another producer, the annual allotment referable to such transferred allotment base, or part thereof, shall be issued to the transferee only if the transfer is effective prior to the issuance of an annual allotment to the transferor or prior to April 1, whichever is the earlier: *Provided,* That for the 1982 calendar year that date shall be April 30 instead of April 1.

(Secs. 1-19, 48 Stat. 31, as amended (7 U.S.C. 601-674))

Dated: April 15, 1982.

D. S. Kuryloski,

Deputy Director, Fruit and Vegetable Division.

[FR Doc. 82-10874 Filed 4-21-82; 8:45 am]

BILLING CODE 3410-02-M

---

7 CFR Part 1106

[Milk Order No. 106]

Milk in the Oklahoma Metropolitan Marketing Area; Order Suspending Certain Provisions

AGENCY: Agricultural Marketing Service, USDA.

ACTION: Suspension of rules.

SUMMARY: This action suspends certain provisions of the Oklahoma Metropolitan Federal milk order that relate to the shipping requirements for supply plants and the limits on the amount of milk from individual producers that may be diverted directly to nonpool plants and still be priced under the order. For the months of April and May 1982, the suspension reduces the amount of milk that a supply plant must ship to pool distributing plants in order to qualify as a pool plant. Also, the amount of milk that may be moved directly from farms to nonpool plants for manufacturing would be increased. The suspension was requested by two producer cooperative associations because of increased milk production relative to fluid milk sales. Comments received in response to the proposed action asserted that the suspension is needed to assure the efficient disposition of reserve milk supplies and to assure that dairy farmers who have regularly supplied the fluid milk needs of the market will continue to have their milk pooled and priced under the order.

EFFECTIVE DATE: April 21, 1982.

FOR FURTHER INFORMATION CONTACT: Robert F. Groene, Marketing Specialist, Dairy Division, Agricultural Marketing Service, U. S. Department of Agriculture, Washington, D.C. 20250, (202) 447-4824.

SUPPLEMENTARY INFORMATION: Prior documents in this proceeding: Notice of Proposed Suspension: Issued March 25, 1982; published March 31, 1982 (47 FR 13532).

It has been determined that this action is not a major rule under the criteria set forth in Executive Order 12291.

It has also been determined that any need for suspending certain provisions of the order on an emergency basis precludes following certain review procedures set forth in Executive Order 12291. Such procedures would require that this document be submitted for review to the Office of Management and Budget at least 10 days prior to its publication in the **Federal Register.** However, this would not permit the issuance of the suspension on a timely enough basis since it is necessary that it be effective for the month of April 1982. In this instance, the initial request for this action was received on March 23, 1982. A notice of proposed suspension was issued on March 25, 1982, inviting interested parties to submit comments on the proposed action on or before April 7, 1982.

It has also been determined that this action will not have a significant economic impact on a substantial number of small entities. This action lessens the regulatory impact of the order on certain milk handlers and tends to ensure that dairy farmers will continue to have their milk priced under the order and thereby receive the benefits that accrue from such pricing.

This order of suspension is issued pursuant to the provisions of the Agricultural Marketing Agreement Act of 1937, as amended (7 U.S.C. 601 *et seq.*), and of the order regulating the handling of milk in the Oklahoma Metropolitan marketing area.

Notice of proposed rulemaking was published in the **Federal Register** (47 FR 13532) concerning a proposed suspension of certain provisions of the order. Interested persons were afforded opportunity to file written data, views, and arguments thereon.

PART 1106—MILK IN OKLAHOMA METROPOLITAN MARKETING AREA

After consideration of all relevant material, including the proposal set forth in the aforesaid notice, data, views, and arguments filed thereon, and other available information, it is hereby found and determined that for the months of April and May 1982 the following provisions of the order do not tend to effectuate the declared policy of the Act:

1. In § 1106.7(b), that part of the provisions that reads "until any month of such period in which less than 20 percent of the plant receipts and diverted milk specified previously herein is transferred to plants described in paragraph (a) of this section. A plant not meeting such 20 percent requirement in any month of such January-August period shall be qualified under this

paragraph in any remaining month of the year only if transfers of fluid milk products (except filled milk) from the plant during the month to plant(s) described in paragraph (a) of this section are at least 50 percent of the plant receipts and diverted milk specified previously herein".

2. In § 1106.13(e)(1), that part of the provisions that reads ", subject to the conditions of paragraph (e)(3) of this section, a total quantity of milk not in excess of total" and "received at all pool plants during the month. Diversions in excess of such quantity shall not be eligible under this section and the diverting cooperative shall specify the dairy farmers whose diverted milk is not so eligible. If the cooperative association fails to designate such person, status under this section shall be forfeited with respect to all milk diverted by such cooperative association".

3. In § 1106.13(e)(2), that part of the provisions that reads ", subject to the conditions of paragraph (e)(3) of this section," and ", in a total quantity not in excess of the milk of producers not members of such cooperative association received at such pool plant(s) during the month. Milk diverted in excess of such quantity shall not be eligible under this section and the diverting handler shall specify the dairy farmers whose diverted milk is not so eligible. If a handler fails to designate such persons, status under this section shall be forfeited with respect to all milk diverted by such handler".

4. In § 1106.13, paragraph (e)(3).

Statement of Consideration

This action reduces the amount of milk that supply plants must ship to pool distributing plants to attain pool plant status under the order. Under the suspension, a supply plant would need to make but one shipment to a pool distributing plant to qualify as a pool plant.

The action also increases the amount of milk that may be moved directly from farms to nonpool manufacturing plants and still be priced under the order. Without the suspension, diversions would be limited to producers who deliver not less than 15 percent of their producer milk to pool plants. In addition, diversions to nonpool plants by proprietary handlers and cooperatives could not exceed the quantity of milk received at pool plants.

The suspension was requested by two cooperative associations representing producers supplying the market.

The suspension is necessary because increased production by producers over year earlier levels has not been matched by a corresponding increase in fluid milk sales. Also, milk production is increasing seasonally and is expected to peak in April and May. At the same time, fluid milk sales are expected to decline as schools begin closing in May. Because of this imbalance between fluid milk sales and production, continuation of the current order provisions would require unnecessary shipments from supply plants and would inhibit the efficient movement of milk directly from farms to manufacturing plants for surplus disposal. In the absence of the suspension costly and inefficient movements of milk would have to be made by the cooperatives and a supply plant operator solely for the purpose of assuring that the milk of dairy farmers who have regularly supplied the fluid milk needs of the market would continue to be pooled under the order.

Interested parties were given an opportunity to submit written data, views, or arguments concerning the suspension. No views in opposition to the suspension were received. One of the cooperative associations that originally requested the action and the operator of proprietary pool supply plant filed comments in support of the suspension action. Each of the parties pointed out, however, that the provisions that were proposed to be suspended with respect to the supply plant shipping standards would not accomplish the objectives intended. These parties urged that the suspension action be broadened to fully accomplish the stated objective of requiring a supply plant that qualified as a pool plant during each of the preceding months of September through January to make but one shipment to a pool distributing plant to qualify as a pool plant. On the basis of these comments, the scope of the suspension action regarding supply plant shipping standards has been expanded slightly from that reflected in the notice of proposed rulemaking.

It is hereby found and determined that thirty days' notice of the effective date hereof is impractical, unnecessary and contrary to the public interest in that:

(a) This suspension is necessary to reflect current marketing conditions and to maintain orderly marketing conditions in the marketing area in that without this action uneconomic movements of milk would be made solely for the purpose of pooling the milk of dairy farmers who have regularly been associated with the market;

(b) This suspension does not require of persons affected substantial or extensive preparation prior to the effective date; and

(c) Notice of proposed rulemaking was given interested parties and they were afforded opportunity to file written data, views or arguments concerning this suspension.

Therefore, good cause exists for making this order effective upon publication in the **Federal Register.**

List of Subjects in 7 CFR Part 1106

Milk marketing orders.

It is therefore ordered, That the aforesaid provisions of the order are hereby suspended for the months of April and May 1982.

(Secs. 1-19, 48 Stat. 31, as amended (7 U.S.C. 601-674))

Effective date: April 21, 1982.

Signed at Washington, D.C. on April 15, 1982.

C. W. McMillan,

Assistant Secretary, Marketing and Inspection Services.

[FR Doc. 82-10931 Filed 4-20-82; 8:45 am]

BILLING CODE 3410-02-M

---

7 CFR Part 1139

Milk in the Lake Mead Marketing Area; Order Suspending Certain Provisions

AGENCY: Agricultural Marketing Service, USDA.

ACTION: Suspension of rule.

SUMMARY: This action suspends certain order provisions relating to how much milk not needed for fluid (bottling) use may be moved directly from farms to manufacturing plants and still be priced under the order. The suspension removes the limit on such movements of milk for April 1982 through August 1982. The suspension was requested by a cooperative association to assure the efficient disposition of milk not needed for fluid use and still maintain producer status under the order for its dairy farmer members regularly associated with the market.

EFFECTIVE DATE: April 21, 1982.

FOR FURTHER INFORMATION CONTACT: Maurice M. Martin, Marketing Specialist, Dairy Division, Agricultural Marketing Service, U.S. Department of Agriculture, Washington, D.C. 20250, (202) 447-7183.

SUPPLEMENTARY INFORMATION: Prior documents in this proceeding: Notice of Proposed Suspension: Issued March 24, 1982, published March 30, 1982 (47 FR 13368)

It has been determined that this action is not a major rule under the criteria set forth in Executive Order 12291. It also has been determined that the need for suspending certain provisions of the

order on an emergency basis precludes following certain review procedures set forth in Executive Order 12291. Such procedures would require that this document be submitted for review to the Office of Management and Budget at least 10 days prior to its publication in the Federal Register. However, this would not permit the issuance of the suspension on the timely basis necessary to include April 1982 in the suspension period.

William T. Manley, Acting Administrator, Agricultural Marketing Service, has determined that this action would not have a significant economic impact on a substantial number of small entities. This action lessens the regulatory impact of the order on certain milk handlers and tends to ensure that dairy farmers will continue to have their milk priced under the order and thereby receive the benefits that accrue from such pricing.

This order of suspension is issued pursuant to the provisions of the Agricultural Marketing Agreement Act of 1937, as amended (7 U.S.C. 601 *et seq.*), and of the order regulating the handling of milk in the Lake Mead marketing area.

Notice of proposed rulemaking was published in the **Federal Register** (47 FR 13368) concerning a proposed suspension of certain provisions of the order. Interested persons were afforded opportunity to file written data, views, and arguments thereon. The proponent of the suspension and another cooperative association filed written comments supporting the suspension.

After considering all relevant material, including the proposal in the notice, the comments received from the proponent and the other cooperative association and other available information, it is hereby found and determined that for the months of April through August 1982 the following provisions of the order do not tend to effectuate the declared policy of the Act:

1. In § 1139.13(d)(2), the sentence "The total quantity of milk so diverted may not exceed 30 percent in the months of March through July and 20 percent in other months of the producer milk which the association causes to be delivered to pool plants during the month".

2. In § 1139.13(d)(3), the sentence "The total quantity of milk so diverted may not exceed 30 percent in the months of March through July and 20 percent in other months of the milk received at such pool plant from producers and for which the operator of such plant is the handler during the month".

Statement of Consideration

This action removes the limit on the amount of producer milk that a cooperative association or other handlers may divert from pool plants to nonpool plants during the months of April 1982 through August 1982. The order now provides that a cooperative association may divert up to 30 percent of its total member milk received at all pool plants or diverted therefrom during the months of March through July and 20 percent during all other months. Similarly, the operator of a pool plant may divert up to 30 percent of its receipts of producer milk (for which the operator of such plant is the handler during the month) during the months of March through July and 20 percent during all other months.

The suspension was requested by a cooperative association that supplies the market with a substantial part of its fluid milk needs and handles all of the market's reserve milk supplies. The basis for the action is that current marketing conditions require the association to handle an increasing quantity of reserve milk supplies during April–August 1982 because of increased milk production by the market's producers. The cooperative stated that milk production by producers on the market is approximately 5 percent above last year. It indicated that this situation is aggravated by the fact that sales to its fluid outlets this year have been declining because of decreases in the market's Class I sales. Also, the cooperative stated that in February some of the milk of its members had to be depooled since the current diversion limitations did not accommodate the market situation at that time.

In view of this, it is expected that the cooperative's reserve milk supplies during April through August 1982 will exceed the quantity of producer milk that may be diverted to nonpool manufacturing plants under the order's present diversion limitations. Without the suspension, it is likely that some of the milk of its member producers who have regularly supplied the fluid market would have to be moved uneconomically, first to pool plants and then to nonpool manufacturing plants, in order to continue producer status for such milk during April through August 1982. The suspension will permit these producers to continue sharing in the proceeds of the marketwide pool.

It is hereby found and determined that 30 days' notice of the effective date hereof is impractical, unnecessary, and contrary to the public interest in that:

(a) This suspension is necessary to reflect current marketing conditions and to maintain orderly marketing conditions in the marketing area in that the most efficient method of handling milk not needed for the fluid market is by direct movements from producers' farms to manufacturing outlets. This suspension allows for such economical movements of milk while the dairy farmers involved retain producer status.

(b) This suspension does not require of persons affected substantial or extensive preparations prior to the effective date; and

(c) Notice of proposed rulemaking was given interested parties and they were afforded opportunity to file written data, views, or arguments concerning this suspension. No views were received in opposition to the proposed suspension.

Therefore, good cause exists for making this order effective upon publication in the **Federal Register**.

List of Subjects in 7 CFR 1139

Milk marketing orders.

It is therefore ordered, That the aforesaid provisions of the order are hereby suspended for April 1982 through August 1982.

(Secs. 1–19, 48 Stat. 31, as amended, 7 U.S.C. 601–674)

Effective Date: April 21, 1982.

Signed at Washington, D.C., on April 15, 1982.

C. W. McMillan,

Assistant Secretary, Marketing and Inspection Services.

[FR Doc. 82-10882 Filed 4-20-82; 8:45 am]

BILLING CODE 3410-02-M

DEPARTMENT OF ENERGY

Economic Regulatory Administration

10 CFR Parts 500, 501, and 504

Implementation of Powerplant and Industrial Fuel Use Act of 1978

AGENCY: Economic Regulatory Administration, Energy.

ACTION: Final rules.

SUMMARY: The Economic Regulatory Administration (ERA) in the Department of Energy (DOE) is revising its final rules implementing the Powerplant and Industrial Fuel Use Act of 1978 (FUA). This revision implements certain amendments made by the Omnibus Budget Reconciliation Act of 1981 (OBRA). OBRA eliminated DOE's authority to issue prohibition orders for the involuntary conversion of existing electric powerplants to coal or another alternate fuel. OBRA now allows DOE to issue prohibition orders *only* where

the owner or operator of a powerplant commences the proceeding by filing an affirmative certification as to both the technical capability and financial feasibility of the conversion.

ERA is also amending the requirements relating to the financial feasibility finding that ERA must make in order to issue a prohibition order. This amendment is being adopted in response to public comment but was not specifically proposed in the November 27, 1981 Notice of Proposed Rulemaking. ERA is therefore issuing § 504.6 as an interim final rule so as to provide the public with an additional opportunity to submit comments on this section.

EFFECTIVE DATE: May 21, 1982. Comment date: Written comments on the interim final rule (§ 504.6) are due by May 21, 1982.

ADDRESSES: All comments should be addressed to Public Hearing Management, Docket No. ERA-R-81-14, Department of Energy, Room 7146, 12th Street and Pennsylvania Avenue, NW, Washington, D.C. 20461.

FOR FURTHER INFORMATION CONTACT:

Henry K. Garson, Office of the General Counsel, Department of Energy, Room 6B-178, Forrestal Building, 1000 Independence Avenue, SW, Washington, D.C. 20585, (202) 252-2967;

Constance Buckley, Fuels Conversion Division, Fuels Program, Economic Regulatory Administration, Department of Energy, Room GA-093, RG-62, Forrestal Building, 1000 Independence Avenue, SW, Washington, D.C. 20585, (202) 252-1774;

Jack Vandenberg, Office of Public Information, Economic Regulatory Administration, Room 7120, 12th Street and Pennsylvania Ave., NW, Washington, D.C. 20461, (202) 633-9451.

SUPPLEMENTARY INFORMATION: OMB Control Number: #1903-0077

Paperwork Reduction Act

Information collection requirements contained in this regulation have been approved by the Office of Management and Budget (OMB) under the provisions of the Paperwork Reduction Act of 1980 (Pub. L. 96-511) and have been assigned OMB #1903-0077.

I. Background
II. Comments
III. Procedural Matters

I. Background

On November 27, 1981, ERA issued a Notice of Proposed Rulemaking (NOPR) (46 FR 58051) to revise the rules implementing the Powerplant and Industrial Fuel Use Act of 1978 (FUA) in order to account for certain amendments to Title III of FUA (existing facilities) made by the Omnibus Budget Reconciliation Act of 1981 (OBRA). Section 1021 of OBRA provides the authority to the Secretary of Energy to prohibit the use of petroleum or natural gas as a primary energy source in certain existing electric powerplants only where the owner or operator commences a proceeding by filing an affirmative certification, which must be concurred in by ERA. Under former law (section 301 of FUA and section 2 of the Energy Supply and Environmental Coordination Act of 1974, as amended, 15 U.S.C. 791 *et seq.* (ESECA)), the Secretary could order the involuntary conversion of such powerplants. The certification relates to the powerplant's technical capability of using coal or another alternate fuel and the financial feasibility of using coal or another alternate fuel as a primary energy source in the powerplant. These final rules provide a substantially revised prohibition order process for existing electric powerplants that wish to make the certification provided for under section 301 of FUA, as amended by OBRA (certifying powerplants).

The prohibition order procedures for major fuel burning installations (MFBIs) (see 10 CFR 501.51) and for powerplants that elected continued coverage under former section 301 of FUA (electing powerplants), pursuant to the election provisions found at 46 FR 48118 (October 1, 1981), have not been changed. The prohibition order procedures for facilities that make the election for continued coverage under section 2 of ESECA are found at 10 CFR Parts 303 and 305.

ERA is issuing § 504.6 in interim rule form since that section now contains amendments to the substantive criteria for ERA's issuance of a prohibition order, particularly as it relates to the financial feasibility standard. In the case of electing powerplants and MFBIs, ERA must make findings regarding these criteria before it can impose a prohibition order against the use of natural gas or petroleum as a primary energy source. Similarly, certifying powerplants must submit information to support these criteria.

The following section summarizes the comments received by ERA in response to the NOPR.

II. Comments

The comments to the NOPR enthusiastically supported the proposed revisions, and, in general, they merely requested clarification of ERA's intent behind the new prohibition order process for certifying powerplants. Above all, the comments sought as much flexibility in the applicable procedures and standards as feasible within the statutory framework, particularly with regard to the owner or operator's ability to amend a certification due to changed circumstances and the amendment's impact on the effective date of the prohibition order.

Section 301(d) of FUA, as amended by OBRA, provides that a certification may be amended at any time "in order to take into account changes in relevant facts and circumstances; except that no such amendment to such a certification may be made after the date of any final prohibition * * *." This provision of the statute makes clear that a certification, and an order issued in response thereto, may be amended prior to the date of any "final prohibition". The term "final prohibition", as used in section 301(d) of FUA, as amended, means the date on which the prohibitions contained in a final order become effective by operation of the terms and conditions set forth in the final order. It is anticipated that terms and conditions in the compliance schedule, attached to any final prohibition order under § 504.5(d), will include conditions precedent to the prohibitions contained in the final order becoming effective. For example, the certifying owner or operator of the powerplant may include a condition in the compliance schedule that provides for the determination by a regulatory body that certain pollution control equipment will not be required as a result of the conversion. If such a condition is included in the compliance schedule, the prohibitions contained in the final order do not become effective or become final prohibitions until such determination is made. If the determination is never made, the prohibitions in the order never become effective.

This procedure enables the Department to issue final prohibition orders which will become subject to judicial review as well as provide a mechanism to account for "changes in relevant facts and circumstances" between the issuance of the final prohibition order and the final effectiveness of the prohibitions contained therein. Further, should there be a change in relevant facts and circumstances that is not provided for in the compliance schedule, such change could also serve as the basis for amendment of the prohibition order itself pursuant to Section 301(d) of FUA.

A. General Definitions

Under § 500.2, ERA defines OBRA to mean the Omnibus Budget Reconciliation Act of 1981, Pub. L. 97-35, and ESECA to mean the Energy Supply and Environmental Coordination Act of 1974, as amended, 15 U.S.C. 791 *et seq.*, in order to correct a definitional oversight in the NOPR.

B. Prohibition Order Procedures

1. *Public comment in proceeding.* One commenter requested a period of 60 days be provided for submission of written comments after a Notice of Acceptance of a certification and ERA's proposed prohibition order are published in the **Federal Register** instead of the 45 day period currently proposed. In addition, the commenter sought a 30 day period instead of a 14 day period in which additional written comments could be submitted following a public hearing. ERA believes that the 45 day and 14 day periods, the same as currently in effect, are sufficent to enable the public to file comments and still enable ERA to keep the proceeding within a manageable time period.

2. *Prohibition order procedures for certifying powerplants.* Some commenters expressed concern about the distinction between a "final prohibition order" and a "final prohibition" under § 501.52(c). The commenters believe that the provision of § 501.52(d) permits a certification to be amended prior to the effective date of a prohibition order and this implies that the order may not become final prior to the effective date of the prohibitions. They state that this implication conflicts with the different dates for the order's finality and effectiveness as described in § 501.52(c)(2). The commenters desire language that indicates a prohibition order becomes final on the effective date of the prohibitions. The commenters are concerned that if the order is final before the effective date of the prohibitions contained in the order, their statutory option to amend the certification will be foreclosed through classification of the order as "final", before the fulfillment of conditions or other contingencies (as may be contained in the prohibition order compliance schedule) occurring subsequent to ERA's prohibition order proceeding. The commenters also sought clarification on the relationship between the effective date of the order and the various contingencies in the prohibition order compliance schedule, which if not favorably satisfied, may result in the utility's withdrawal or amendment of its certification.

In its proposal, ERA did not intend to limit a utility's opportunity, and statutory option, to amend its certification at any time prior to the effectiveness of the prohibitions contained in a final prohibition order. A utility continues to have the option to amend its certification in order to take into account changes in relevant facts and circumstances, such as the conditions and circumstances, occurring subsequent to the ERA proceeding, including those contained in the prohibition order compliance schedule submitted by the utility.

ERA believes that it is important to preserve the divergent dates for the final order and the effectiveness of the prohibitions contained in the order. As proposed by ERA, a prohibition order will be final for purposes of the judicial review provisions of section 702 of FUA. However, a utility may amend its certification prior to the effective date of the prohibitions contained in the order under section 301(d) of FUA, as amended. Note that the effective date of the prohibitions contained in the order is derived from the date the utility believes all its contingencies and conditions outlined in its prohibition order compliance schedule will be satisfied. Note also that as part of the certification, the prohibition order compliance schedule itself may be amended to account for any other contingencies that may become apparent after the submission of the original compliance schedule. ERA has clarified § 501.52(c)(2) to state that the *prohibitions* in the prohibition order shall not become effective (at which point a utility can no longer amend its certification) until the date of the prohibitions stated in the order, or, where the order is subject to conditions occurring subsequent to ERA's proceeding, until all conditions of the compliance schedule are met.

One commenter perceived certain ambiguities in the language of § 501.52(b)(5) and (b)(3) in regard to the proposed order recipient's ability to recertify a powerplant as coal capable after a termination of a prior proceeding or after issuance of a final Notice of Non-Concurrence. Section 501.52(b)(5), as proposed, provided that ERA may terminate a prohibition order proceeding prior to the date upon which a prohibition order becomes final whenever ERA believes that the proposed order recipient's certification "is in some way deficient". For objectivity and clarity, ERA has eliminated the quoted phrase and referenced the standard for termination to specified regulations. In order to avoid unnecessary confusion as to the meaning of the effective date of the final prohibition order, ERA is deleting the effective date language; instead ERA must terminate a proceeding prior to the issuance of a final prohibition order. In addition, ERA also clarifies its intent that a final Notice of Non-Concurrence does not have any relevance to a utility's ability to make a later certification under section 301 of FUA.

One commenter requested clarification of whether § 501.52(b)(5) is merely an expansion of § 501.52(b)(2) or stands as an independent provision. ERA believes § 501.(b)(5) is necessary to highlight ERA's ability to terminate a prohibition order proceeding at any time prior to the issuance of a final prohibition order if the requirements of specified regulations are not satisfied.

One commenter requested the addition of a provision for the preparation of a Tentative Staff Analysis (TSA) in certification proceedings so that it could serve as a working document for public comment in the proceeding. ERA believes there is no need for the costly and time-consuming preparation of a TSA in these proceedings since all information, which might serve as a basis for public comment, may be obtained from the certification submitted by the utility, the Notice of Acceptance of the certification and the proposed prohibition order which states the reasons for the order, or, if ERA cannot accept the certification, from the Notice of Proposed Non-Concurrence. In addition, a statement containing the basis upon which the final order is issued will be contained in the final order.

3. *Interim Rule—Substantive criteria for prohibition orders.* One commenter advised that ERA should segregate the regulations governing the substantive criteria of § 504.6 for prohibition orders for certifying powerplants and electing powerplants and those for MFBIs. The commenter maintains that Congress amended the substantive criteria for only powerplants in OBRA, while it retained the criteria for MFBIs; therefore, the prohibition criteria should not necessarily be the same for these two categories. However, according to the commenter, the substantive criteria should be the same for orders to be issued to electing powerplants and certifying powerplants but should be amended to better account for the voluntary nature of the proceeding. In the commenter's view the proceeding is voluntary in the case of certifying powerplants in that they initiate the process by filing their certifications and prohibition order compliance schedules,

while in the case of electing powerplants, they have effectively consented to the proposed conversion in that they have elected to continue under the former section 301 of FUA.

In comparing the statutory prohibition criteria under former section 301 of FUA, which applies not only to existing MFBIs but also to electing powerplants under section 1022 of OBRA, with the criteria under section 301, as amended by OBRA, which applies to certifying powerplants, ERA believes the statutory criteria in each case are the same. Therefore, the standards for ERA's findings in the case of MFBIs and electing powerplants, and the required certification in the case of certifying powerplants should be essentially the same.

OBRA merely changed the involuntary conversion proceeding for certain powerplants into a voluntarily initiated proceeding. Therefore, in the case of certifying powerplants, ERA has attempted in these final rules to streamline the procedures and allow a utility the opportunity to amend its certification to the fullest extent permitted under FUA, as amended. ERA has decided to adopt several changes to the substantive criteria as suggested by the commenters, as described below.

In response to proposed § 504.6(c) on the technical capability criterion, one commenter suggested that the regulation should be streamlined to eliminate all other technical capability considerations except the ability of the unit, from the point of fuel intake, to physically sustain combustion of a given fuel and to maintain heat transfer. ERA has adopted the spirit of this suggestion and revised a footnote to encompass several of the more minor considerations which may still be relevant.

In regard to the substantial physical modification criterion of § 504.6(d), one commenter suggested that footnote illustrations of what is regarded as a substantial modification warranted by the complexity of the engineering project or by the impact upon operations at the site should be more directly focused on significant alterations of the boiler itself. ERA agrees that alterations to the boiler were its major concern in the currently effective regulation in 45 FR 53681 (August 12, 1980) (1980 rules) and accordingly, ERA has revised the illustrations.

For the substantial reduction in rated capacity criterion contained in § 504.6(e)(4), ERA has adopted one commenter's suggestion that the environmental requirements that will be considered in the appraisal of the impact of a reduction in rated capacity are those requirements expected to be applicable at the date the prohibitions contained in the final prohibition order become effective. This change offers greater certainty as to which environmental requirements are to govern in the test and also affords greater flexibility in the case of statutory or administrative amendment of the environmental requirements in the course of the prohibition order proceeding.

Some commenters advised that the financial feasibility criterion of § 504.6(f) should no longer be equated with the general cost calculation described in § 504.12. Under the 1980 rules, if the company's cost of using an alternate fuel does not substantially exceed the cost of using imported petroleum as calculated under § 504.12, ERA presumes that the financial feasibility test is satisfied. The proposed order recipient may then seek to rebut this presumption utilizing an inadequacy of capital test. The commenters maintain that the cost calculation test, which attempts to measure the economic desirability of a conversion project, is not appropriate to evaluate the company's actual ability to obtain sufficient capital to finance a conversion. In addition, commenters consider the current presumption as inappropriate in the new voluntary proceedings for certifying powerplants. The commenters offered a test that would gauge a firm's ability to obtain sufficient financing for its project.

ERA has decided to adopt this suggestion and make the new elements of the financial feasibility criterion applicable to all existing powerplants and MFBIs. ERA will deem a conversion to be financially feasible where the firm has the actual ability to obtain sufficient capital to finance the conversion, including all necessary land, coal and ash handling equipment, and pollution control equipment, without violating legal restrictions on its ability to raise debt or equity capital. Because of the capital-intensive nature of coal conversions, their long-term financial feasibility may involve, in part, projected cost-of-generation savings relative to continued operation on petroleum or natural gas. Therefore, where helpful in clarifying the long-term financial feasibility of a conversion, ERA may analyze the economic benefits anticipated from operation of the converted unit or units using coal or other alternate fuel relative to those from continued operation using petroleum or natural gas. ERA believes that this revised test will provide more flexibility in the prohibition order process.

C. Environmental requirements and procedures for certifying powerplants

One commenter urged ERA to either eliminate § 504.9, as proposed, or structure its procedure as an option to the traditional agency review of an environmental document prepared by a proposed order recipient. The commenter viewed § 504.9 as unnecessarily restrictive in that its independent party approach could interfere with a utility's ability to use the environmental documents prepared in the early planning of projects to support the environmental review. Another commenter was concerned primarily about securing a significant role for the powerplant's owner or operator in the preparation of any environmental documents and studies required in the environmental process.

The comments also objected to the absorption of all the costs by the utility that may arise from the preparation of environmental documents, while ERA maintains the exclusive supervisory and approval authority for the process. One commenter suggested that if § 504.9 were adopted as an option to the traditional environmental process, it would not object to a reasonable fee, not to exceed $50,000, payable to ERA to cover costs for the preparation of environmental documents based on the utility's environmental studies. This fee payment would accompany a certification seeking a prohibition order.

One commenter suggested that a financial hardship exception be made to the NEPA procedures under § 504.9 for those certifying powerplants whose conversion to an alternate fuel would be financially infeasible if the powerplant owner or operator were required to bear the costs of the NEPA process.

ERA has decided to adopt § 504.9 substantially as proposed, incorporating a financial hardship exception as above described. Since the prohibition order proceeding for certifying powerplants is purely voluntary in that the owner or operator of a powerplant may commence and discontinue the proceeding at any time by simply filing a certification or withdrawing it under section 301 of FUA, as amended, and since the proceeding benefits the owner or operator of the powerplant and its ratepayers, the government generally should not be responsible for the costs arising from the environmental compliance process necessary to issue the prohibition order. However, ERA foresees that, in a limited number of conversions, the costs associated with NEPA compliance may make the conversion financially infeasible if the

powerplant owner or operator were required to bear these costs. In such cases, to assist a conversion that is in the public interest, ERA may, within its discretionary authority, waive the requirements for payment of the costs of NEPA compliance by the powerplant owner or operator under § 504.9 (a) and (b) and conduct the environmental review at ERA's cost.

In addition, before a prohibition order may be issued under FUA, ERA has the ultimate responsibility to comply with the National Environmental Policy Act of 1969, 42 U.S.C. 4321 *et seq.* (NEPA). Therefore, to fulfill its responsibility, ERA must retain exclusive authority for supervision and final approval of the necessary documents. However, the proposed order recipient may produce and submit its own environmental documents prepared during the planning of its project to the independent party conducting the environmental review. These documents may, of course, be used as the basis or support for any environmental documents, such as an Environmental Impact Statement (EIS) or Environmental Assessment (EA) which must be prepared.

III. Procedural Matters

A. Section 102 of the National Environmental Policy Act (NEPA)

DOE has determined that these final regulations will not constitute a major Federal action significantly affecting the quality of the human environment within the meaning of section 102(2)(C) of NEPA since there are no significant substantive alterations to the existing program. The changes in these regulations reflect the elimination of the DOE's authority to issue prohibition orders involuntarily to certifying powerplants. The environmental impacts resulting from initial implementation of FUA are addressed in a final Environmental Impact Statement prepared pursuant to the requirements of NEPA (DOE/EIS 0038, April 1979). In view of the foregoing, the preparation of an EIS is not required.

B. Regulatory Flexibility Act

In accordance with the statutory provisions of the OBRA amendments to FUA, ERA cannot institute a prohibition order proceeding against an existing powerplant unless the powerplant's owner or operator voluntarily submits the required certification. Very few small entities within the meaning of the Regulatory Flexibility Act will be affected in any manner by these proposals. DOE hereby certifies that these final regulations are not likely to have a significant economic impact on a substantial number of small entities within the meaning of the Regulatory Flexibility Act. Therefore, DOE is not required to publish a final regulatory flexibility analysis under section 603 of that Act.

C. Executive Order No. 12291

DOE has determined that these final regulations are not a major rule under Executive Order No. 12291, which requires the preparation of a Regulatory Impact Analysis for major regulations. These final rules will not be likely to result in an annual effect on the economy of $100 million or more. DOE foresees no major increase in costs or prices for consumers, industries, geographic regions, or Federal, State or local government agencies. DOE does not consider it likely that the rules will result in significant adverse effects on competition, employment investment, or productivity. Therefore, no Regulatory Impact Analysis is required.

The proposals were submitted to the Office of Management and Budget for review at least 10 days prior to publication in the **Federal Register.**

List of Subjects

10 CFR Part 500

Business and industry, Electric power plants, Energy conservation, Natural gas, Petroleum.

10 CFR Part 501

Administrative practice and procedure, Business and industry, Electric power plants, Energy conservation, Environmental impact statements, Investigations, Natural gas, Petroleum.

10 CFR Part 504

Business and industry, Electric power plants, Energy conservation, Environmental impact statements, Natural gas, Petroleum.

(Department of Energy Organization Act, Pub. L. 95-91, 42 U.S.C. 7101 *et seq.;* Energy Supply and Environmental Coordination Act of 1974, Pub. L. 93-319, as amended by Pub. L. 94-163, Pub. L. 95-70, and Pub. L. 95-620, (15 U.S.C. 719 *et seq.);* Powerplant and Industrial Fuel Use Act of 1978, Pub. L. 95-620, as amended by Pub. L. 97-35 (42 U.S.C. 8301 *et seq.*); Omnibus Budget Reconciliation Act of 1981, Pub. L. 97-35)

In consideration of the foregoing, Parts 500, 501 and 504, Subchapter E, "Alternate Fuels" of Chapter II, Title 10 of the Code of Federal Regulations, are amended as set forth below.

Issued in Washington, D.C., on the 7th day of April 1982.

Rayburn Hanzlik,

Administrator, Economic Regulatory Administration.

PART 500—DEFINITIONS

For reasons set out in the preamble, Part 500 of Chapter II, Title 10 of the Code of Federal Regulations is amended as shown.

§ 500.2 [Amended]

By adding definitions of "certifying powerplant", "electing powerplant", "ESECA", and "OBRA" to § 500.2 to read as follows:

* * * * *

"Certifying powerplant" means an existing powerplant whose owner or operator seeks to obtain a prohibition order against the use of natural gas or petroleum either totally or in a mixture with coal or an alternate fuel by filing a certification as to both the technical capability and financial feasibility of conversion to coal or another alternate fuel pursuant to section 301 of FUA, as amended.

* * * * *

"Electing powerplant" means an existing powerplant, which (1) has been issued a proposed prohibition order under former section 301 (b) or (c) of FUA prior to August 13, 1981, the date of enactment of the Omnibus Budget Reconciliation Act of 1981, Pub. L. 97-35 (OBRA); and (2) files an election to continue the current prohibition order proceeding under provisions of the former section 301 of FUA, rather than under amended section 301 of FUA.[1] Under the election provisions, an existing powerplant which has an order pending against it under section 2 of the Energy Supply and Environmental Coordination Act of 1974, as amended, 15 U.S.C. 791 *et seq.* (ESECA), as of August 13, 1981, may also elect to continue the current proceeding under section 2 of ESECA. Electing powerplants under ESECA are not included in the FUA definition of "electing powerplant". Relevant regulations governing ESECA proceedings are found at 10 CFR Part 303 and 305. These elections must have been filed with DOE by November 30, 1981 in the case of FUA orders and by January 14, 1982 in the case of ESECA orders.

* * * * *

[1] The election provisions are published at 46 FR 48118 (October 1, 1981) and will not be codified in the Code of Federal Regulations.

"ESECA" means the Energy Supply and Environmental Coordination Act of 1974, as amended, 15 U.S.C. 791 *et seq.*

* * * * *

"OBRA" means the Omnibus Budget Reconciliation Act of 1981, Pub. L. 97-35.

PART 501—ADMINISTRATIVE PROCEDURES AND SANCTIONS

For the reasons set out in the preamble, Part 501 of Chapter II, Title 10 of the Code of Federal Regulations is amended as shown.

1. By revising the heading for § 501.51 and by an entry for § 501.52 in the table of contents of Part 501 as follows:

* * * * *

Subpart E—Prohibition Rules and Orders

* * * * *

Sec.
501.51 Prohibitions by order—existing major fuel-burning installations and electing powerplants.
501.52 Prohibitions by order—certifying powerplants.

* * * * *

Authority: Department of Energy Organization Act, Pub. L. 95-91 (42 U.S.C. 7101 *et seq.*); Energy Supply and Environmental Coordination Act of 1974, Pub. L 93-319, as amended by Pub. L. 94-163, Pub. L. 95-70, and Pub. L. 95-620, (15 U.S.C. 719 *et seq.*); Powerplant and Industrial Fuel Use Act of 1978, Pub. L. 95-620, as amended by Pub. L. 97-35, (42 U.S.C. 8301 *et seq.*); Omnibus Budget Reconciliation Act of 1981, Pub. L. 97-35.

2. By revising §§ 501.31(b) and 501.33(b) to read as follows:

§ 501.31 Written comments.

* * * * *

(b) *Existing facilities.* Except as may be provided elsewhere in these regulations, ERA shall provide a period of at least 45 days for submission of written comments concerning a proposed prohibition rule or order or a petition for an exemption or permit. This period shall commence, in the case of a petition for a noncertification exemption, on the day after publication of the Notice of Acceptance, and in the case of a certification exemption, on the day after publication of the Notice of Acceptance and Availability of Certification in the Federal Register, in accordance with §501.63(a). In the case of proposed prohibition rules or orders to be issued to existing major fuel-burning installations (MFBIs) and electing powerplants, ERA shall also provide for a period of at least 45 days for submission of written comments concerning a Tentative Staff Analysis. This period shall commence on the day after publication of the Notice of Availability of the Tentative Staff Analysis in the Federal Register. In the case of prohibition order proceedings for certifying powerplants under section 301 of FUA, as amended, ERA shall provide a period of at least 45 days, beginning the day after the Notice of Acceptance of certification or Notice of Proposed Non-Concurrence is published, for submission of written comments concerning the certification and ERA's proposed prohibition order, and requests for public hearings. Prohibition order proceedings under section 301, as amended by OBRA, will have only one period of 45 days, since no Tentative Staff Analysis will be prepared. The comment period may be extended by ERA in accordance with § 501.7. See §§ 501.51(b) and 501.52(b) of this Part with respect to the comment periods applicable to prohibitions by order to existing facilities and the extension of such comment periods. Written comments shall be filed in accordance with § 501.7.

§ 501.33 Requests for a public hearing.

* * * * *

(b) *Existing facilities.* In the case of a petition for an exemption from a prohibition imposed either by the Act or by a final rule or order issued by ERA to an existing facility under former sections of Title III of FUA or Title III of FUA, as amended, or a petition for an exemption or permit, if applicable, any interested person may submit a written request that ERA convene a public hearing in accordance with section 701 of FUA within 45 days after the notice of the filing of a petition is published in the Federal Register. In the case of a proposed prohibition rule or order to be issued to existing MFBIs, and electing powerplants under former section 301, the 45 day period in which to request a public hearing shall commence upon the publication of the Notice of Availability of the Tentative Staff Analysis. In the case of a proposed prohibition order to be issued to certifying powerplants under section 301 to FUA, as amended, the 45 day period in which to request a public hearing commences upon publication of the Notice of Acceptance of certification. This time limit may be extended at the discretion of ERA in accordance with § 501.7.

3. By amending § 501.51 by revising the section heading and the introductory text of paragraph (a) and paragraphs (b)(2), (3) and (d)(2)(ii) to read as follows:

§ 501.51 Prohibitions by order—Existing major fuel-burning installations and electing powerplants.

(a) ERA may prohibit by order the use of petroleum or natural gas as a primary energy source or in amounts in excess of the minimum amount necessary to maintain reliability of operation consistent with reasonable fuel efficiency in an existing MFBI, or electing powerplant, if:

* * * * *

(b) * * *

(2) Pursuant to section 701 of FUA, prior to the issuance of a final order to an existing MFBI or electing powerplant, ERA shall publish a proposed order in the Federal Register together with a statement of the reasons for the order. In the case of a proposed order that would prohibit the use of petroleum or natural gas as a primary energy source, the finding required by former section 301(b)(1) of the Act in the case of a powerplant or the finding required by section 302(a)(1) of the Act in the case of an installation shall be published with such proposed order.

(3) ERA shall provide a period for the submission of written comments of at least three months after the date of the proposed order. During this period, the recipient of the proposed order and any other interested person must submit any evidence that they have determined at that time to support their respective positions as to each of the findings that ERA is required to make under former section 301(b) of the Act in the case of a powerplant, or the findings required by section 302(a) of the Act in the case of an installation. A proposed order recipient may submit additional new evidence at any time prior to the close of the public comment period which follows publication of the Tentative Staff Analysis or prior to the close of the record of any public hearing, whichever occurs later. A request by the proposed order recipient for an extension of the three month period may be granted at ERA's discretion.

* * * * *

(d) * * *

(2) * * *

(ii) Sufficient information for ERA to make the findings required by former section 301(b) of FUA in the case of a powerplant and section 302(b) of FUA in the case of an installation.

* * * * *

4. By adding a new § 501.52 to read as follows:

§ 501.52 Prohibitions by order—certifying powerplants.

(a) ERA may prohibit by order the use of petroleum or natural gas as a primary energy source or in amounts in excess of the minimum amount necessary to maintain reliability of operation consistent with maintaining reasonable

fuel efficiency in an existing powerplant if the owner or operator of the powerplant certifies, and ERA concurs in such certification in accordance with the requirements of §§ 504.5, 504.6 and 504.8.

(b) *Notice of order and participation.* (1) ERA may hold a conference with the proposed order recipient, at the recipient's election, prior to issuing the proposed order. The conference may resolve any questions regarding the certification required by section 301 of the Act, as amended, and §§ 504.5, 504.6, and 504.8, and ERA's review and concurrence therein.

(2) Pursuant to section 701(b) of FUA, prior to the issuance of a final order to a certifying powerplant owner or operator, ERA must publish in the **Federal Register**, a proposed prohibition order stating the reasons for such order. ERA will review all of the information submitted by a proposed order recipient within 60 days after receipt by ERA. If the certification is complete, ERA will, within 30 days after the end of the 60 day review period, publish in the **Federal Register** a Notice of Acceptance of certification together with a proposed prohibition order stating therein the reasons for such order. This commences the prohibition order proceeding. If ERA does not believe it is able to concur in the certification, ERA shall notify the proposed order recipient and shall publish a Notice of Proposed Non-Concurrence in the **Federal Register** within 30 days after the end of the 60 day review period. If ERA finds that the certification with compliance schedule is incomplete, ERA will notify the proposed prohibition order recipient as to the deficiencies, and provide an additional period of 30 days for the certification to be amended and resubmitted. If a complete certification is not submitted within this period, the proceeding shall be terminated in accordance with § 501.52(b)(5). ERA will notify the proposed order recipient and other parties to the proceeding of the termination and publish a notice in the **Federal Register.** ERA, on its own motion, may extend any period of time by publishing a notice to that effect in the **Federal Register.**

(3) The publication of the Notice of Acceptance or Notice of Proposed Non-Concurrence commences a period of 45 days during which interested persons may submit written comments or request a public hearing. During this period, the recipient of the proposed order and any other interested person may submit any evidence that they have available relating to the proposed order, the certification or the concurrence that ERA must make. A proposed order recipient may submit additional new evidence at any time prior to the close of the public comment period which follows the commencement of the proceeding or prior to the close of the record of any public hearing, whichever occurs later. A request for an extension of the 45 day period may be granted at ERA's discretion. In the case of a Notice of Acceptance, as set forth in § 504.9, no final prohibition order can be issued until any necessary environmental review pursuant to the National Environmental Policy Act of 1969, 42 U.S.C 4321 *et seq.* (NEPA) has been completed. Upon completion of the NEPA review and unless ERA determines on the basis of the record of the proceeding that the certification fails to meet the requirements of §§ 504.5, 504.6, and 504.8, ERA shall publish a final prohibition order, together with the information required by paragraph (c) of this section. In the case of a Notice of Proposed Non-Concurrence, at the end of the 45 day comment period, ERA will notify the proposed order recipient and parties to the proceeding and publish a final Notice of Non-Concurrence in the **Federal Register**, if ERA determines it cannot concur in the certification based upon additional information submitted during the proceeding. If, at the end of the 45 day period, ERA believes it can concur in the certification, ERA will notify the proposed order recipient and parties to the proceeding and publish a Notice of Acceptance followed by a new 45 day comment period.

(4) If a hearing has been requested, ERA shall provide interested persons with an opportunity to present oral data, views and agruments at a public hearing held in accordance with Subpart C of this part. The hearing may consider, among other matters, the sufficiency of the certification of the owner or operator of the powerplant required by section 301 of FUA, as amended, and §§ 504.5, 504.6, and 504.8 of these regulations.

(5) ERA may terminate a prohibition order proceeding at any time prior to the date upon which a final prohibition order is issued whenever ERA believes, from any information contained in the record of the proceeding, that the certification does not meet the requirements of section 301 of the Act, as amended, or §§ 504.5, 504.6, and 504.8 of these regulations. If ERA terminates the proceeding or publishes a final Notice of Non-Concurrence, or the proposed order recipient fails to submit a complete certification, ERA will notify the proposed order recipient and other parties to the proceeding and publish a notice in the Federal Register. In such event, the proposed order recipient may submit a new certification under any provision of section 301 of the Act, as amended, at a later date. Specifically, a Notice of Non-Concurrence under either section 301(b) or 301(c) shall not affect a proposed order recipient's ability to make a certification under the other subsection.

(c) *Record and decision to issue a final order.* (1) ERA will base its determination to issue an order on consideration of the whole record or those parts thereof cited by a party and supported by and in accordance with reliable, probative and substantial evidence.

(2) ERA shall include in the final order a written statement of the basis upon which the final order is issued, and its concurrence in the required certification. A copy of the final order and a summary of the basis therefor will be published in the **Federal Register.** While the prohibition order is final for purposes of judicial review under section 702 of FUA, the prohibitions contained in the final order shall not become effective for purposes of amendment under section 301(d) of FUA, as amended, and § 501.52(d) of these regulations until the effective date of the prohibitions stated in the order, or, where the order is subject to one or more conditions subsequent listed in the prohibition order compliance schedule, until all its conditions are met.

(d) *Amendment to certifications under §§ 504.5 and 504.6.* The proposed prohibition order recipient may amend its compliance schedule under § 504.5(d), or its certification under section 301 of FUA, as amended, and §§ 504.5, 504.6 and 504.8 of these regulations in order to take into account changes in relevant facts and circumstances at any time prior to the effective date of the prohibitions contained in the final prohibition order.

(e) *Rescission of prohibition orders.* The rescission or modification of final prohibition orders issued to existing electric powerplants will be governed by the procedure in § 501.101 of these regulations. OMB Control Number: 1903-0077.

PART 504—EXISTING FACILITIES

For reasons set out in the Preamble, Part 504 of Chapter II, Title 10 of the Code of Federal Regulations is amended as shown.

1. By revising the table of contents for Subpart B as follows:

* * * * *

Subpart B—Prohibitions

Sec.
504.2 Purpose and scope.
504.3–504.4 [Reserved]
504.5 Prohibitions by order (certifying powerplants under amended section 301 of FUA).
504.6 Prohibitions by order (case-by-case).
504.7 Prohibitions against excessive use of petroleum or natural gas in mixtures—electing powerplants and major fuel-burning installations.
504.8 Prohibitions against excessive use of petroleum or natural gas in mixtures—certifying powerplants.
504.9 Environmental requirements for certifying powerplants.

* * * * *

Authority: Department of Energy Organization Act, Pub. L. 95-91, 42 U.S.C. 7101 *et seq.*; Energy Supply and Environmental Coordination Act of 1974, Pub. L. 93-319, as amended by Pub. L. 94-163, Pub. L. 95-70, and Pub. L. 95-620, 15 U.S.C. 719 *et seq.*; Powerplant and Industrial Fuel Use Act of 1978, Pub. L. 95-620, as amended by Pub. L. 97-35, 42 U.S.C. 8301 *et seq.;* Omnibus Budget Reconciliation Act of 1981, Pub. L. 97-35.

2. By revising § 504.2 to read as follows:

§ 504.2 Purpose and scope.

(a) Sections 504.5, 504.6, and 504.8, below, set forth the prohibitions that ERA, pursuant to section 301 of the Act, as amended, may impose upon existing powerplants after a review of the certification and prohibition order compliance schedule submitted by the owner or operator of a powerplant. Sections 504.5, and 504.8 are explanatory sections, and § 504.6 provides the informational requirements necessary to support the certification.

(b) Sections 504.6 and 504.7, below, also set forth the prohibitions that ERA may impose upon existing MFBIs, pursuant to sections 302 and 303 of the Act, and certain electing powerplants, pursuant to former section 301 (b) and (c) of FUA, where ERA can make the findings as to the unit's technical capability and financial feasibility to use coal or another alternate fuel as a primary energy source. The prohibitions may be made to apply to existing MFBIs and electing electric powerplants unless an exemption is granted by ERA under Subparts D and E of this part. Any person who owns, controls, rents or leases an existing installation or electing powerplant may be subject to the prohibitions imposed by and the sanctions provided for in the Act or these regulations, if ERA can make the findings required by former section 301 (b) and (c) of FUA and section 302 (a) and (b) of the Act, as amended.

§ 504.3 [Removed and reserved]

3. By removing and reserving § 504.3.

4. By revising § 504.5 to read as follows:

§ 504.5 Prohibitions by order (certifying powerplants under section 301 of FUA, as amended).

(a) In the case of existing powerplants, ERA may prohibit, in accordance with section 301 of the Act, as amended, the use of petroleum or natural gas as a primary energy source where the owner or operator of the powerplant presents a complete certification concurred in by ERA. The certification, which may be presented at any time, pertains to the unit's technical capability and financial feasibility to use coal or another alternate fuel as a primary energy source in the unit. The informational requirements necessary to support a certification are contained in § 504.6 of these regulations. A prohibition compliance schedule which meets the requirements of § 504.5(d) shall also be submitted.

(b) If ERA concurs with the certification, a prohibition order on the powerplant's use of petroleum or natural gas will be issued following the procedure outlined in § 501.52 of these regulations.

(c) The petitioner may amend its certification at any time prior to the effective date of the prohibitions contained in the final prohibition order in order to take into account changes in relevant facts and circumstances by following the procedure contained in § 501.52(d).

(d) *Prohibition order compliance schedule.* The certification described above, which forms the basis for the issuance of a prohibition order to a powerplant, shall include a prohibition order compliance schedule. The compliance schedule should contain the following:

(1) A schedule of progressive events involved in the conversion project, including construction of any facilities for the production of fuel or fuel handling equipment, and contracts for the purchase of alternate fuels, and estimated date of compliance with the applicable prohibitions of the Act; and

(2) A schedule indicating estimated dates for obtaining necessary federal, state, and local permits and approvals. Any prohibition order issued under the certification provisions of §§ 504.5, 504.6, and 504.8 will be subject to appropriate conditions subsequent so as to delay the effectiveness of the prohibitions contained in the final prohibition order until the above events or permits have occurred or been obtained.

(OMB Control Number: 1903-0077)

5. By amending § 504.6 by revising the introductory text of paragraph (a), revising paragraphs (b), (c)(1), (d), (e) (introductory text on (e)(4)), and (f) (retain all footnotes) to read as follows:

§ 504.6 Prohibitions by order (case-by-case).

(a) ERA may prohibit, by order, the use of natural gas or petroleum as a primary energy source in existing MFBIs and existing powerplants under certain circumstances. In the case of certifying powerplants under section 301 of the Act, as amended, the petitioner must present evidence to support the certification, required by § 504.6 (c), (d), (e), and (f). In the case of electing powerplants and in the case of MFBIs, ERA must make the following findings required by § 504.6 (c), (d), (e), and (f), in order to issue a prohibition order to the unit, pursuant to former section 301 (b) or (c), and section 302, respectively:

* * * * *

(b) In the case of MFBIs and electing powerplants, ERA must make a proposed finding regarding the technical capability of a unit to use alternate fuel as identified in paragraph (a) (1) of this section prior to the date of publication of the notice of the proposed prohibition. ERA will publish this finding in the Federal Register along with the notice of the proposed prohibition.

(c) *Technical capability.* (1) In the case of MFBIs, and electing and certifying powerplants, ERA will consider "technical capability" on a case-by-case basis in order to make the required finding. In the case of a certifying powerplant, the powerplant should present information to support the certification relevant to the considerations set forth below. ERA will consider the ability of the unit, from the point of fuel intake to physically sustain combustion of a given fuel and to maintain heat transfer.[2]

* * * * *

(d) *Substantial physical modifications.* In the case of existing MFBIs, and electing and certifying powerplants, ERA will make its determination on whether a physical modification to a unit is "substantial" on a case-by-case basis. In the case of certifying powerplants, ERA will

[2] ERA will not ordinarily consider the nature or absence of appurtenances outside the unit. For example, ERA will examine the furnace configuration and ash removal capability but will not normally consider the need to install pollution control equipment as a measure of technical capability. Furthermore, ERA will not normally conclude that the absence of fuel handling equipment, such as conveyor belts, pulverizers, or unloading facilities, bears on the issue of a unit's "technical capability" to burn an alternate fuel.

consider the following factors set forth below for the purpose of concurrence in the certification. ERA will consider physical modifications made to the unit as "substantial" where warranted by the magnitude and complexity of the engineering task or where the modification would impact severely upon operations at the site.[3] ERA will not, however, assess physical modification on the basis of cost.

(e) *Substantial reduction in rated capacity.* In the case of existing MFBIs, and electing and certifying powerplants, ERA will make this determination on the basis of the following factors. A certifying powerplant should present information to support its certification regarding these factors in order for ERA to make its review for concurrence. * * *

(4) In assessing whether a unit's derating is not substantial, ERA will consider the impact of a reduction in rated capacity of the unit taking into consideration all necessary appurtenances such as air pollution control equipment required to burn an alternate fuel in compliance with environmental requirements expected to be applicable at the date the prohibitions contained in the final prohibition order become effective. However, the potential order recipient may raise the following impacts on derating in rebuttal, if under paragraph (e)(2) of this section, or case-by-case, if under paragraph (e)(3) of this section.

* * * * *

(f) *Financial feasibility.* In the case of existing MFBIs, and certifying and electing powerplants, ERA will make this finding based on the following considerations. A certifying powerplant should present information to support its certification relevant to these factors in order for ERA to make its review for concurrence. Conversion of a unit to burn coal or an alternate fuel shall be deemed financially feasible if the firm has the ability to obtain sufficient capital to finance the conversions, including all necessary land, coal and ash handling equipment, pollution control equipment, and all other necessary conversion expenditures, without violating legal restrictions on its ability to raise debt or equity capital. ERA will consider any economic or financial factors presented by the proposed order recipient in determining the firm's ability or inability to finance the conversion including, but not limited to, the following:

(1) The required coverage ratios on the firm's debt and preferred stock;

(2) The firm's investment program; and

(3) The financial impact of the conversion, including other conversions which are or may be undertaken voluntarily by the proposed order recipient or imposed upon the recipient's system by the Act, and including pending or planned construction or reconstruction of alternate-fuel-fired plants and plants exempt from FUA prohibitions.[4] Where helpful in clarifying the long-term financial feasibility of a conversion, DOE may analyze the economic benefits anticipated from operation of the converted unit or units using coal or other alternate fuel relative to those from continued operation using petroleum or natural gas.

(OMB Control Number: 1903-0077)

6. By revising § 504.7 (a) and (b) to read as follows:

§ 504.7. Prohibitions againts excessive use of petroleum or natural gas in mixtures—electing powerplants and major fuel-burning installations.

(a) In the case of existing MFBIs and electing powerplants, if ERA finds that it is technically and financially feasible for a unit to use a mixture of petroleum or natural gas and an alternate fuel as its primary energy source, ERA may prohibit, by order, the use in that unit of petroleum or natural gas, or both, in amounts exceeding the minimum amount necessary to maintain reliability of operation consistent with maintaining reasonable fuel efficiency of the mixture. For installations, such minimum amount determined by ERA shall not be less than 25 percent.

(b) In making the technical feasibility finding required by former section 301 (b) and (c) of the Act and paragraph (a) of this section, ERA may weigh "physical modification" or "derating of the unit," but these considerations, by themselves, will not control the technical feasibility finding. A technical feasibility finding might be made notwithstanding the need for substantial physical modification. The economic consequences of a substantial physical modification are taken into account in determining financial feasibility.

* * * * *

7. By adding a new § 504.8 to read as follows:

§ 504.8. Prohibitions against excessive use of petroleum or natural gas in mixtures—certifying powerplants.

(a) In the case of certifying powerplants, ERA may prohibit the use of petroleum or natural gas in such powerplant in amounts exceeding the minimum amount necessary to maintain reliability of operation consistent with maintaining the reasonable fuel efficiency of the mixture. This authority is contained in section 301(c) of the Act, as amended. The owner or operator of the powerplant may certify at any time to ERA that it is technically capable and financially feasible for the unit to use a mixture of petroleum or natural gas and coal or another alternate fuel as a primary energy source. In assessing whether the unit is technically capable of using a mixture of petroleum or natural gas and coal or another alternate fuel as a primary energy source, for purposes of this section, the extent of any physical modification necessary to convert the unit and any concomitant reduction in rated capacity are not relevant factors. So long as a unit as proposed to be modified would be technically capable of using the mixture as a primary energy source under § 504.6(c), this certification requirement shall be deemed met. The criteria for certification of financial feasibility are found at § 504.6(f). In addition, the powerplant's owner or operator must submit a prohibition compliance schedule, which meets the requirements of § 504.5(d).

(b) If ERA concurs with the certification, a prohibition order against the unit's excessive use of petroleum or natural gas in the mixture will be issued following the procedure outlined in § 501.52 of these regulations.

(c) The petitioner may seek to amend its certification in order to take into account changes in relevant facts and circumstances by following the procedure contained in § 501.52(d).

Note.—The authority of ERA implemented under this section should not be confused with the other two fuel mixture provisions of these regulations. One is the general requirement that petitioners for permanent exemptions demonstrate that the use of a mixture of natural gas or petroleum and an alternate fuel is not economically or technically feasible (See § 504.15). The second is the permanent fuel mixtures exemption itself (See § 504.56).

(OMB Control Number: 1903-0077)

[3] Generally, modification of a unit to burn coal or an alternate fuel will be considered insubstantial if significant alterations to the boiler, such as a change to the furnace configuration or a complete respacing of the tubes, are not required. Minor alterations such as replacement of burners or additions of soot blowers, and additions or alterations outside the boiler, shall not cause the modification to be substantial.

[4] ERA will not require the proposed order recipient to cancel or defer construction or reconstruction of any alternate-fuel-fired facility, or any facility exempt from the prohibitions of the Act, for which a decision to finance such facility has been made by the appropriate company official before the publication of the prohibition order. The proposed order recipient may choose to cancel or defer any such facility.

8. By adding § 504.9 to read as follows:

§ 504.9 Environmental requirements for certifying powerplants.

Under §§ 501.52, 504.5 and 504.6 of these regulations, ERA may prohibit, in accordance with section 301 and section 303 (a) or (b) of FUA, as amended, the use of natural gas or petroleum, or both, as a primary energy source in any certifying powerplant. Under sections 301(c) and 303(a) of FUA, as amended, and §§ 501.52, 504.6, and 504.8 of these regulations, ERA may prohibit the excessive use of natural gas or petroleum in a mixture with an alternate fuel as a primary energy source in a certifying powerplant.

(a) *NEPA Compliance.* Except as provided in paragraph (c) of this section, where the owner or operator of a powerplant seeks to obtain an ERA prohibition order through the certification procedure, and did not hold either a proposed prohibition order under former section 301 of FUA or pending order under section 2 of ESECA, it will be responsible for the costs of preparing any necessary Environmental Assessment (EA) or Environmental Impact Statement (EIS) arising from ERA's obligation to comply with NEPA. The powerplant owner or operator shall enter into a contract with an independent party selected by ERA, who is qualified to conduct an environmental review and prepare an EA or EIS, as appropriate, and who does not have a financial or other interest in the outcome of the proceedings, under the supervision of ERA. The NEPA process must be completed and approved before ERA will issue a final prohibition order based on the certification.

(b) *Environmental review procedure.* Except as provided in paragraph (c) of this section, environmental documents, including the EA and EIS, where necessary, will be prepared utilizing the process set forth above. ERA, the powerplant owner or operator and the independent third party shall enter into an agreement for the owner or operator to engage and pay directly for the services of the qualified third party to prepare the necessary documents. The third party will execute an ERA prepared disclosure document stating that he does not have any conflict of interest, financial or otherwise, in the outcome of either the environmental process or the prohibition order proceeding. The agreement shall outline the responsibilities of each party and his relationship to the other two parties regarding the work to be done or supervised. ERA shall approve the information to be developed and supervise the gathering, analysis and presentation of the information. In addition, ERA will have the authority to approve and modify any statement, analysis, and conclusion contained in the third party prepared environmental documents.

(c) *Financial hardship.* Whenever the bona fide estimate of the costs associated with NEPA compliance, if borne by the powerplant owner or operator, would make the conversion financially infeasible, ERA may waive the requirement set forth in paragraphs (a) and (b) of this section and perform the necessary environmental review.

(OMB Control Number: 1903-0077)

[FR Doc. 82-11046 Filed 4-20-82; 8:45 am]

BILLING CODE 6450-01-M

SECURITIES AND EXCHANGE COMMISSION

17 CFR Parts 240 and 249

[Release No. 34-18647; IC-12375]

System of Classification for Purposes of Exempting Smaller Issuers From Certain Reporting and Other Requirements

AGENCY: Securities and Exchange Commission.

ACTION: Final rules.

SUMMARY: The Commission today announced the adoption of a new rule and rule amendments which exempt a class of smaller issuers from the registration and reporting requirements under the Securities Exchange Act of 1934. The new provisions represent an effort by the Commission to alleviate the burdens imposed on small business in complying with the Securities Exchange Act of 1934 to the greatest extent possible consistent with the public interest and the protection of investors.

EFFECTIVE DATE: April 15, 1982. Rule 12g-1 is effective with respect to fiscal years ending on or after December 20, 1981. Thus, a calendar year issuer who exceeded the Section 12(g) criteria on December 31, 1981, but whose total assets were below $3 million on that date, is not required to register under that section.

FOR FURTHER INFORMATION CONTACT: William E. Toomey, (202) 272-2573, Office of Chief Counsel, Division of Corporation Finance, Securities and Exchange Commission, 500 North Capitol Street, Washington, D.C. 20549.

SUPPLEMENTARY INFORMATION: On October 27, 1981, the Commission published for comment a proposed rule and amendments to rules to establish a system of classifying small issuers for purposes of exempting certain of them from reporting and other obligations under the Securities Exchange Act of 1934 (the "Exchange Act") (15 U.S.C. 78a *et seq.*).[1] The proposals were designed to provide a rational adjustment to the criteria for entry into or exit from the Exchange Act reporting system and to eliminate the costs to the smallest issuers of complying with the registration and reporting provisions under that Act. In light of the comments received and the Commission's experience, the proposals have been adopted substantially as proposed.[2]

This release first will summarize the statutory registration and reporting system under the Exchange Act, then discuss the classification system and how it will operate with the statutory system, and, finally, discuss the original proposals and revisions made in response to the commentators. Appendix A to the release sets forth certain examples of how the new classification system operates and Appendix B is an algorithm intended to further assist readers in understanding the system. The release will also describe the procedure for withdrawal of Form 10 registration statements (17 CFR 249.210) filed after December 20, 1981. Attention is directed to the text of the rules and forms for a more complete understanding.

I. Discussion

A. The Registration and Reporting System

There are three provisions of the Exchange Act by which a class of securities of an issuer becomes subject

---

[1] Release No. 34-18189 (October 27, 1981) (46 FR 52382) ("Proposing Release"). Prior to the proposals, the Commission published an advance notice of proposed rulemaking announcing that it was considering the advisability of classifying companies under the Exchange Act so that defined classes of smaller issuers might have modified reporting and other requirements. Release No. 34-16866 (June 13, 1980) (45 FR 40145).

[2] The Commission notes that the rules governing the National Association of Securities Dealers Automated Quotation System ("NASDAQ"), an automated inter-dealer quotation system for over-the-counter securities, require that an issuer of securities registered for trading on NASDAQ must be a reporting company under the Exchange Act. Thus, a company which seeks to have its shares traded by means of NASDAQ must comply with the Exchange Act, regardless of whether the company may otherwise be exempt under the classification rules adopted herein. Presently, NASDAQ registration standards require that companies must have total assets of $2 million. *See* Release No. 34-18054 (August 21, 1981) (46 FR 4334). Thus, certain issuers with assets exceeding $2 million but less than $3 million may be eligible for NASDAQ registration even though they may be exempted from Exchange Act reporting requirements under the classification rules.

to the registration, continuous reporting and other requirements of that Act. Under Section 12(b), an issuer may register a security on a national securities exchange. Under Section 12(g), if an issuer on the last day of its fiscal year has total assets exceeding $1 million and a class of securities held of record by 500 or more persons, the issuer is required to file a registration statement of that class of securities. Section 15(d) applies to any issuer that has filed a registration statement which has become effective pursuant to the Securities Act of 1933 (15 U.S.C. 77a *et seq.*) (the "Securities Act"). The issuer's duty to file reports under Section 15(d) is automatically suspended if and so long as any class of the issuer's securities are registered pursuant to Section 12.

A company with a class of securities registered under Sections 12(b) or 12(g), as well as a company subject to Section 15(d), is required to file annual, quarterly and current reports pursuant to Section 13(a). In addition, the proxy provisions of Section 14 (a) and (c), the Williams Act amendments and the short swing profit provisions of Section 16 apply to classes of securities registered under Section 12.

Registration under Section 12(g) may be terminated whenever the class of equity securities is held of record by less than 300 persons. Under Section 15(d), an issuer is required to file reports for the fiscal year in which its registration statement becomes effective, but is suspended from that obligation during any subsequent year in which there are less than 300 record holders of the class of securities to which the registration statement relates on the first day of the fiscal year.

B. The New Classification System

In general, the new classification system will change the existing system in three ways.

First, a company will not have to register under Section 12(g) until it has 500 or more record holders of a class of equity securities and it has total assets of $3 million or more, this being an increase from the present total assets criterion of $1 million.

Second, once a company's class of securities is registered under Section 12(g), it may deregister at any time that it has fewer than 500 record holders of the class and the company has had total assets of less than $3 million at the end of each of its last three fiscal years. This represents an addition to the present provision which permits a company to terminate its registration at any time it has fewer than 300 shareholders.

Third, a company that is subject to Section 15(d) will generally have its obligation to file reports under that section suspended with respect to any fiscal year in which on the first day of such fiscal year it has fewer than 500 security holders and has had total assets under $3 million at the end of its three most recent fiscal years. The new provision for suspending the reporting obligation under Section 15(d) is not available to an issuer for any year in which it has a registration statement become effective or in the two succeeding years. Under the present system the duty to file reports under Section 15(d) is only suspended if the number of security holders is less than 300.

C. Public Comments on the Proposed Amendments and Principal Revisions

1. *Public Comments.* In the Proposing Release, the Commission proposed new Rule 12g–1 (17 CFR 240.12g–1),[3] amendments to Rule 12g–4 (17 CFR 240.12g–4) and an amendment to Rule 15d–6 (17 CFR 240.15d–6).[4] Under proposed Rule 12g–1, issuers with less than $3 million in total assets would be exempt from the registration provisions of the Exchange Act. Under proposed Rules 12g–4 and 15d–6, issuers would have been required (i) to have had assets under $3 million at the end of each of their last three fiscal years and (ii) to have filed annual reports with the Commission for each of those last three fiscal years before registration could be terminated under Section 12(g) or reporting could be suspended under Section 15(d).

Sixteen comment letters were received, twelve of which supported the comcept of classifying small issuers and exempting certain of them from Exchange Act reporting and other obligations.[5] The commentators offered general views on the concept of classification and specific suggestions on the proposals.

Several commentators believed that criteria other than asset size and number of shareholders should be used for granting an exemption from the reporting requirements. The principal alternative criteria suggested were trading volume and market value of securities. A number of other commentators stated that the shareholder number was generally indicative of investor interest in a company's securities and, thus, of the need for continuous disclosure, and that an asset test was a simple and functional test of an issuer's size in relation to the costs of complying with Exchange Act reporting requirements. While the suggested alternatives may have merit, the Commission believes that a classification system based on asset size and shareholder number is consistent with the present statutory framework and avoids confusion and complex regulations.

Some commentators favored a higher total asset level, while others favored raising or eliminating the shareholder number criterion. The Commission continues to believe that the $3 million total asset figure represents an appropriate adjustment to that criterion to account for the effects of inflation since Congressional enactment of the Exchange Act amendments in 1964. According to data analyzed by the Commission's Directorate of Economic and Policy Analysis ("DEPA"), it would take a nominal dollar amount of nearly $3 million to match the purchasing power in 1964, adjusting for inflation, of $1 million in constant 1964 dollars.[6] The Commission expects to monitor inflation measures and will consider further amendments to the classification rules, if necessary. Moreover, the Commission believes that the 500 shareholders of record criterion is an appropriate indicator of investor interest, one which balances the concerns of cost of

[3] The Commission also proposed, in conjunction with proposed Rule 12g–1, to rescind current Rule 12g–1 (17 CFR 240.12g–1) which was promulgated in order to provide an orderly transition for affected companies to the amendments to the Exchange Act as enacted in the 1964 Securities Acts Amendments. *See* Release No. 34–7429 (September 30, 1964) (29 FR 13462).

[4] Footnote 11 of the Proposing Release stated that if the proposals were adopted, the deferral for small insurance companies of the effective date of the quarterly reporting requirements of Part I of Form 10–Q (17 CFR 240.308a) as provided by Rules 13a–13(c)(1) (17 CFR 240.13a–13(c)(1)) and 15d–13(g)(1) (17 CFR 240.15d–13(g)(1)) would not have to be extended. Since the proposals were not adopted prior to the expiration of the existing deferral at the end of 1981, the Commission published Release No. 33–6370 (December 31, 1981) (46 FR 6354) to extend the deferral through calendar year 1982. Consequently, smaller life insurance companies not required to comply with Part 1 of Form 10–Q will be required to file full Form 10–Q reports beginning in calendar year 1983 unless they are able to terminate or suspend their reporting obligations under the amendments adopted today.

[5] The comment letters and a Summary of Comments (File No. S7–910) are available for public inspection and copying at the Commission's Public Reference Room, 1100 L Street, NW., Washington, D.C. 20549.

[6] According to the DEPA calculations using Gross National Product price deflators, the general price level of the Gross National Product would indicate a $2.74 million 1981 figure for $1 million in 1964 dollars; the increase in the price level associated with the total fixed private, domestic investment would indicate a $2.94 million 1981 figure for $1 million in 1964 dollars; and the increase in the price level associated with total fixed private, domestic investment, excluding residential investment, would indicate a $2.83 million 1981 figure for $1 million in 1964 dollars.

compliance with adequate investor protection. It should be noted also that the amendments as adopted will permit approximately eight percent of those issuers currently reporting under Sections 12(g) or 15(d), or 5.6 percent of all reporting companies, to exit, if they so elect.[7]

2. *Principal Revisions From Proposals.* The system adopted today differs from the one proposed for comment principally in that it does not require annual reports to have been filed for each of the last three fiscal years in order to terminate or suspend the reporting obligations under either Section 12(g) or Section 15(d).

As proposed, once an issuer registered under Section 12(g), it would have had to continue reporting for a minimum of three years, in order to meet the requirement that three annual reports had been filed. While this will still be true for most Section 12(g) registrants, it is now possible for an issuer who had voluntarily registered under Section 12(g), with less than $3 million of assets, to terminate its registration before it has filed three annual reports, if it has had assets under $3 million for its last three fiscal years.

The proposed system would have affected an issuer reporting pursuant to Section 15(d) in a slightly different manner. If a company had a Section 15(d) obligation suspended by the statute for several years because it had less than 300 shareholders and then had an increase in the number of its shareholders to between 300 and 500, it would have had to file annual reports for three years even though its assets had never exceeded $3 million in order to meet the requirement to file three annual reports. Under the system adopted today, such a company's obligation to file reports will continue to be suspended so long as it has less than 500 shareholders and has had less than $3 million of assets at the end of each of its last three fiscal years.

In the above two situations, the Commission agrees with the commentators that the requirement to file three annual reports represented an unnecessary burden on small issuers and was not necessary for the protection of investors. The Commission, however, continues to believe that if a company files a registration statement under the Securities Act it should be required to file reports with the Commission for three years after the effective date of the registration statement unless such obligation is suspended by the statutory provision of having less than 300 security holders. This belief is based on the premise that if a company goes to the public to raise capital, its public shareholders should have some assurance that information about the company will be publicly available for at least some period of time. The statute itself provides that an annual report must be filed for the year in which the registration statement becomes effective and this provision is retained. Thus, the system being adopted today will specifically provide that an issuer cannot be relieved of its obligation to report under Section 15(d) for the year in which a registration statement becomes effective and the two succeeding years unless it meets the statutory test of having fewer than 300 shareholders on the first day of the fiscal year following the year in which the registration statement became effective under the Securities Act.

3. *Revisions to Rule 12g-2 for Business Development Companies.* Rule 12g-2 has been amended to provide certain relief for business development companies. A business development company is defined as: a domestic, closed-end investment company that is operated for the purpose of making investments in small and developing businesses and financially troubled businesses; that makes available significant managerial assistance to its portfolio companies; and that has notified the Commission of its election to be subject to the system of regulation established by Sections 55 through 65 of the Investment Company Act of 1940 (15 U.S.C. 80a *et seq.*) (the "1940 Act").[8] In order to elect to be regulated as a business development company, a company must have a class of equity securities registered under Section 12 of the Exchange Act or have filed a registration statement under that section.[9]

Generally, registration under the Exchange Act may be effected either before or simultaneously with the filing of the company's Notice of Election[10] to be regulated as a business development company. However, as more fully explained in Release No. IC-11703 (March 26, 1981),[11] the automatic registration provisions of Rule 12g-2 have the effect of permitting certain registered investment companies to file a Notice of Election without filing a registration statement under the 1934 Act. As also explained in Release No. IC-11703, investment companies with securities held of record by fewer than 300 persons did not have the benefit of the relief provided by Rule 12g-2. The proposed technical change to Rule 12g-2 would have had the effect of raising this threshold number to 500. As adopted, the amendments to Rule 12g-2 make the provision available to all registered investment companies electing to become business development companies.

4. *Other Revisions.* Certain other changes have been made to other rules to conform them to the classification rules as follows:

(a) Changes have been made to Rule 12h-4 (17 CFR 240.12h-4), which has been redesignated Rule 12h-3, to reflect the adoption of amendments to Rule 12g-4. The changes provide that an issuer relying on Rule 12g-4(a)(2) will not have its Section 15(d) reporting obligation automatically suspended unless it has filed annual reports with the Commission with respect to its three most recent fiscal years.

(b) Changes have been made to the descriptions of the joint Form 12(g)-4/15d-6 (17 CFR 249.323, 333) to conform them to the revisions to Rule 12g-4 and Rule 15d-6 discussed above.

(c) Form 12g-4/15d-6 has been amended to require that issuers specify which exemptive provision is being claimed for termination of registration or suspension of reporting obligations. Thus, issuers must check a box indicating whether the new rules or the statutory exemptive provisions apply.

II. Withdrawal of Certain Form 10's Filed After December 20, 1981

The Commission recognizes that some issuers may have filed a Form 10 under Section 12(g) relating to a fiscal year ending on or after December 20, 1981, prior to the effective date of new Rule 12g-1 adopted herein. Some of these issuers, although they exceeded the Section 12(g) criteria, may not have exceeded the criteria established by new Rule 12g-1 at the end of their fiscal years. The Commission will permit any issuer who filed a Form 10 relating to a fiscal year ending on or after December 20, 1981, who would otherwise be exempt from filing such form by new Rule 12g-1, to withdraw the Form 10 up to the time the form becomes effective. Issuers wishing to withdraw a non-effective Form 10 should write a letter to the Commission requesting that the Form 10 be withdrawn. If a Form 10 relating to a fiscal year ending on or after December 20, 1981, which would

[7] DEPA reviewed data in connection with issuers filing reports in 1979 and estimated that approximately 500 issuers would have fewer than 500 shareholders and less than $3 million in total assets.

[8] Section 2(a)(48) of the 1940 Act.

[9] Section 54(a) of the 1940 Act (15 U.S.C. 80a-53(a)).

[10] Form N-54A (17 CFR 274.53).

[11] (46 FR 19459).

not have been required to be filed under new Rule 12g-1, becomes effective prior to June 1, 1982, the Commission will permit the issuer to terminate its registration by filing a Form 12g-4/15d-6, noting on that form the special use being made of the form. The Form 12g-4/15d-6 must be filed, however, prior to the earlier of the date the first periodic report is required to be filed or July 1, 1982.

III. Availability of Final Regulatory Flexibility Analysis

The Commission has prepared a Final Regulatory Flexibility Analysis in accordance with 5 U.S.C. 603 regarding Rule 12g-1 and the associated amendments adopted herein. A summary of the corresponding Initial Regulatory Flexibility Analysis was included in the Proposing Release at 46 FR 52387. Members of the public who wish to obtain copies of the Final Regulatory Flexibility Analysis should contact Suzanne S. Brannan, Securities and Exchange Commission, 500 North Capitol Street, Washington, D.C. 20549 (202/272-2644).

IV. Certain Findings and Statutory Authority

As required by Section 23(a) of the Exchange Act, the Commission has specifically considered the impact that the rulemaking actions revising 17 CFR Parts 240 and 249, taken pursuant to the various provisions of the Exchange Act would have on competition and has concluded that they would not impose a significant burden on competition not necessary or appropriate in furtherance of the purposes of the Exchange Act.

For the reasons stated above and pursuant to Section 553(d) of the Administrative Procedure Act (5 U.S.C. 553(d)), the Commission finds that there is good cause for making the new rule and related amendments effective immediately in view of the fact that they provide an exemption from the reporting and other obligations imposed by the Exchange Act.

List of Subjects in 17 CFR Part 240

Brokers, Fraud, Investment companies, Municipal securities dealers, Reporting requirements, Securities (bonds, business development companies, exempt securities, government securities, institutional disclosure, investment companies, investments, stocks), Securities associations, Securities exchanges.

List of Subjects in 17 CFR Part 249

Brokers, Investment companies, Municipal securities dealers, Securities (business development companies, institutional disclosure), Securities associations, Securities exchanges.

The Commission hereby adopts the following rule, rule amendments, and form amendments pursuant to sections 12(h) and 23(a) of the Exchange Act (Secs. 12(h), 23(a), 48 Stat. 892, 901; sec. 203(a), 49 Stat. 704; sec. 8, 49 Stat. 1379; sec. 18, 89 Stat. 155; 15 U.S.C. 78*l*(h), 78w(a)). Accordingly, 17 CFR 240.12g-1, 240.12g-2, 240.12g-4, 240.12h-4, 240.15d-6, 249.323 and 333 are revised as set forth below.

By the Commission.

George A. Fitzsimmons,

Secretary.

April 15, 1982.

Appendix A

The following examples illustrate the operation of the classification scheme under Section 12(g):

1. Company A sells securities under Section 4(2) of the Securities Act every year for three years. It eventually has total assets of $10 million and has 450 shareholders. Company A does not have to register its securities under the prior system or the new classification system since the number of shareholders does not exceed 500.

2. Company B sells securities under Section 4(2) of the Securities Act every year for three years. Eventually it has total assets of $2.8 million and has 700 shareholders. Pursuant to Rule 12g-1, Company B would not have to register its securities since its assets do not exceed $3 million. However, under the prior system, Company B would have been required to register its securities since its assets exceeded $1 million and it had more than 500 shareholders.

3. Company C has been registered and reporting under Section 12(g) for four years, and has consistently had $2.8 million in total assets and 450 shareholders. Company C files a certification pursuant to the amendments to Rule 12g-4 that it has fewer than 500 shareholders and has had total assets not exceeding $3 million on the last day of each of its last three fiscal years. Its registration is terminated and no further reports are made by Company C. Under the prior system, the company would have had to continue to be a reporting company since the number of shareholders exceeded 300 persons.

4. Company D has been registered and reporting under Section 12(g) for four years and has consistently had $2.8 million in assets and 450 shareholders. Company D does not file a certification regarding its asset size and shareholder number when Rule 12g-1 and amendments to Rule 12g-4 become effective. Instead it continues to file reports, due two months after the effective date of the rule and amendments. Shortly before the end of its fiscal year, the company determines that it does not wish to continue as a reporting company. It files a certification under the amendments to Rule 12g-4 and its registration is terminated, and it is relieved of its further reporting obligations.

5. Company E has been registered and reporting under Section 12(g) for two years. At the end of the second year, it had $2.8 million in assets and 450 shareholders. In prior years, Company E had less than $1 million in assets. Rule 12g-1 and amendments to Rule 12g-4 become effective. Company E has fewer than 500 shareholders and has had total assets not exceeding $3 million at the end of each of its last three fiscal years and, therefore, may file a Form 12g-4/15d-6 and terminate its registration.

The following examples illustrate the operation of the classification system under Section 15(d):

1. The amendment to Rule 15d-6 becomes effective. Company A registers securities on Form S-18. Pursuant to Section 15(d), Company A is required to file a Form 10-K for the fiscal year in which the registration statement was declared effective. At the beginning of its next fiscal year, Company A has more than 300 shareholders and, thus, must continue to be a reporting company. If Company A files three annual reports reflecting that the company has had under $3 million in assets for three consecutive fiscal years and Company A has under 500 shareholders at the beginning of its next fiscal year, then Company A could file Form 12g-4/15d-6 and its reporting duty under Section 15(d) would be suspended, under the prior system, Company A's reporting duty would not be suspended, since the number of shareholders exceeded 300 persons.

2. The amendment to Rule 15d-6 becomes effective. Company B registers securities on Form S-18. Company B files a Form 10-K for the fiscal year in which its Form S-18 become effective. On the first day of its next fiscal year Company B has 290 shareholders and had $2.8 million in assets for its latest fiscal year. Company B's duty to report under the Exchange Act is suspended. At the beginning of its next year, Company B has $2.8 million assets and 301 shareholders. Company B must resume reporting. If on the first day of the following fiscal year Company B has under 500 shareholders and has continued to have total assets under $3 million it would be eligible to file a Form

12g–4/15d–6 and its duty to report would be suspended under Rule 15d–6(a)(2).

3. The amendment to Rule 15d–6 becomes effective. Company C registers securities on Form S–18. Company C files a Form 10–K for the fiscal year in which its Form S–18 became effective. On the last day of that fiscal year, it has $4 million in total assets and 600 shareholders. Company C must register under Section 12(g). If Company C subsequently has assets below $3 million for three consecutive fiscal years and has fewer then 500 shareholders, Company C could terminate its registration under Section 12(g) pursuant to Rule 12g–4(a)(2) and its reporting obligation also would be suspended under Section 15(d) pursuant to Rule 15d–6(a)(2).

BILLING CODE 8010–01–M

Appendix B

Termination or Suspension of Exchange Act Reporting: An Algorithm

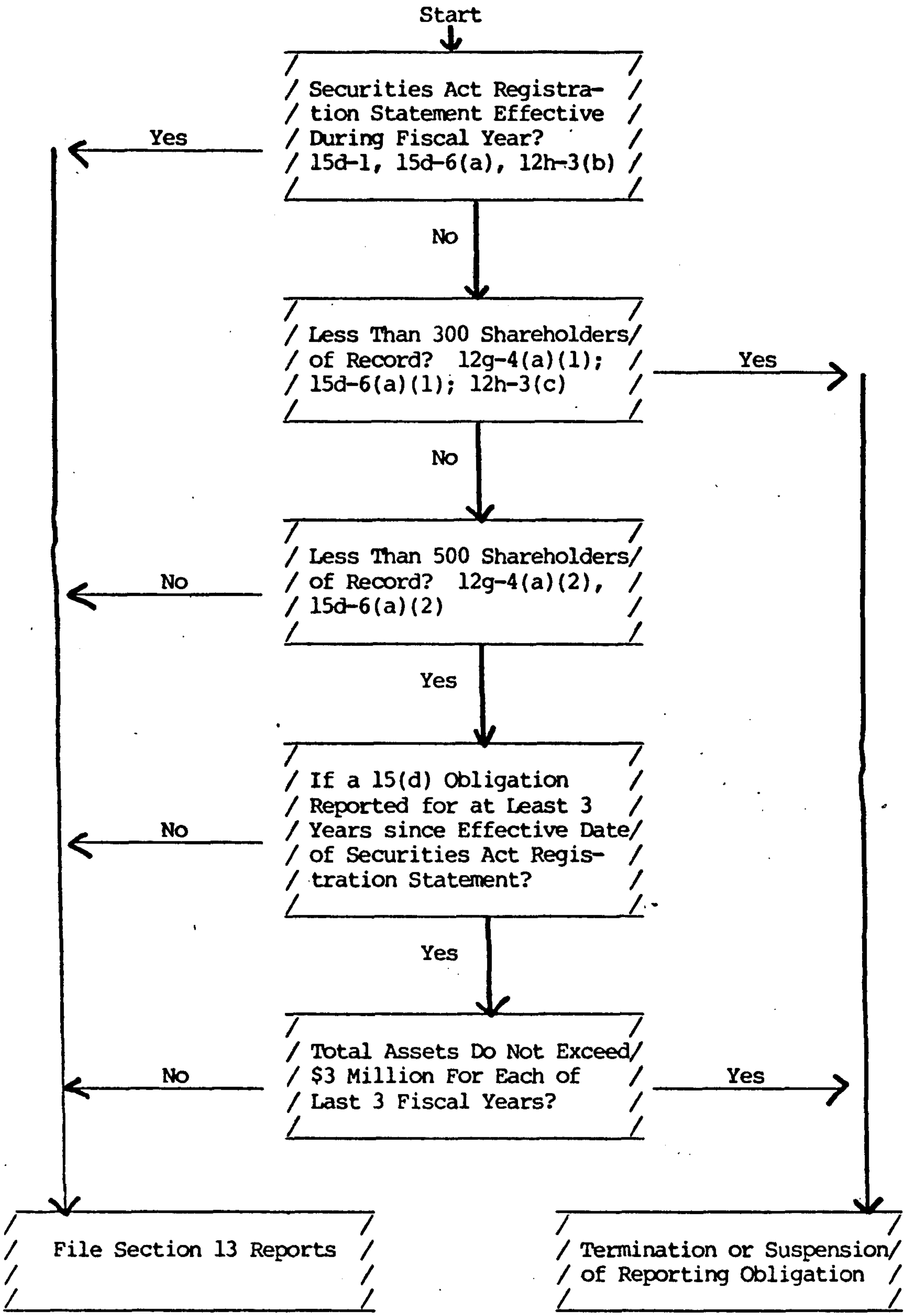

BILLING CODE 8010-01-C

Text of Amendments

17 CFR Chapter II is amended as follows:

PART 240—GENERAL RULES AND REGULATIONS, SECURITIES EXCHANGE ACT OF 1934

1. By revising § 240.12g-1 to read as follows:

§ 240.12g-1 Exemption from section 12(g).

An issuer shall be exempt from the requirement to register any class of equity securities pursuant to section 12(g)(1) if on the last day of its most recent fiscal year the issuer had total assets not exceeding $3,000,000.

2. By revising § 240.12g-2 to read as follows:

§ 240.12g-2 Securities deemed to be registered pursuant to section 12(g)(1) upon termination of exemption pursuant to section 12(g)(2)(A) or (B).

Any class of securities which would have been required to be registered pursuant to section 12(g)(1) of the Act except for the fact that it was exempt from such registration by section 12(g)(2)(A) because it was listed and registered on a national securities exchange, or by section 12(g)(2)(B) because it was issued by an investment company registered pursuant to section 8 of the Investment Company Act of 1940, shall upon the termination of the listing and registration of such class or the termination of the registration of such company and without the filing of an additional registration statement be deemed to be registered pursuant to said section 12(g)(1) if at the time of such termination (a) the issuer of such class of securities has elected to be regulated as a business development company pursuant to sections 55 through 65 of the Investment Company Act of 1940 and such election has not been withdrawn, or (b) securities of the class are not exempt from such registration pursuant to section 12 or rules thereunder delete "or" and all securities of such class are held of record by 300 or more persons.

3. By revising § 240.12g-4 to read as follows:

§ 240.12g-4 Certifications of termination of registration under section 12(g).

(a) Termination of registration of a class of securities shall take effect in 90 days, or such shorter period as the Commission may determine, after the issuer certifies to the Commission on Form 12g-4/15d-6 that: (1) the number of holders of record of such class of securities is reduced to less than 300 persons; or (2) the number of holders of record of such class of securities is reduced to less than 500 persons and the total assets of the issuer have not exceeded $3,000,000 on the last day of each of the issuer's three most recent fiscal years. (b) The issuer's duty to file any reports required under section 13(a) shall be suspended immediately upon certification on Form 12g-4/15d-6, *Provided, however,* That if the certification on Form 12g-4/15d-6 is subsequently withdrawn or denied, the registrant shall, within 60 days after the date of such withdrawal or denial, file with the Commission all reports which would have been required to be filed had the certification on Form 12g-4/15d-6 not been filed. If the issuer had merged into, or consolidated with another issuer or issuers, the form shall be filed by the successor issuer. The form shall be filed in addition to any other report required to be filed with the Commission in connection with the number of holders of record.

§ 240.12h-1 [Removed]

4. By removing § 240.12h-1.

§ 240.12h-2 [Redesignated as § 240.12h-1]

5. By redesignating § 240.12h-2 as § 240.12h-1.

§ 240.12h-3 [Redesignated as § 240.12h-2]

6. By redesignating § 240.12h-3 as § 240.12h-2.

§ 240.12h-4 [Redesignated as § 240.12h-3 and Revised]

7. By redesignating § 240.12h-4 as § 240.12h-3 and revising it to read as follows:

§ 240.12h-3 Partial exemption from section 15(d) upon termination of registration under section 12.

(a) Except as provided under paragraph (b) of this section, the duty to file reports required by section 13(a) of the Act as to any class of securities pursuant to section 15(d) shall be immediately suspended as to such class of securities upon either: (1) The deregistration of such class of securities pursuant to section 12(d) if such class of securities would not thereupon be deemed registered pursuant to section 12(g), or the rules or regulations thereunder; or (2) the filing of a certification on Form 12g-4/15d-6 with respect to such class of securities: *Provided, however,* That if the certification on Form 12g-4/15d-6 is subsequently withdrawn or denied, the registrant shall, within 60 days after the date of such withdrawal or denial, file with the Commission all reports which would have been required to be filed had the certification on Form 12g-4/15d-6 not been filed.

(b) Notwithstanding paragraph (a) of this section, the duty to file reports required by section 13(a) of the Act as to any class of securities pursuant to section 15(d) shall not be automatically suspended unless the issuer has filed reports with the Commission with respect to its most recent three fiscal years.

(c) The suspension shall not be in effect for any subsequent fiscal year at the beginning of which securities of such class are held of record by 300 or more persons and are not exempt pursuant to Rule 15d-6(a)(2).

(d) All duties arising under section 15(d), other than the duty to file the reports required by section 13(a), shall remain in effect under paragraphs (b) and (c) of this section unless otherwise suspended under section 15(d).

8. By revising § 240.15d-1 to read as follows:

§ 240.15d-1 Requirement of annual reports.

Every registrant under the Securities Act of 1933 shall file an annual report, on the appropriate form authorized or prescribed therefor, for the fiscal year in which the registration statement under the Securities Act of 1933 became effective and for each fiscal year thereafter, unless the registrant is exempt from such filing by section 15(d) of the Act or rules thereunder. Annual reports shall be filed within the period specified in the appropriate report form. At the time of filing the annual report, the registrant other than a person registered under the Public Utility Holding Company Act of 1935 or the Investment Company Act of 1940, shall pay to the Commission a fee of $250, no part of which shall be refunded.

9. By revising § 240.15d-6 to read as follows:

§ 240.15d-6 Suspension of duty to file reports.

(a) The duty of any issuer to file reports pursuant to section 15(d) of the Act for any fiscal year shall be suspended if,

(1) At the beginning of such fiscal year, other than the fiscal year within which such registration statement under the Securities Act of 1933 became effective, all securities of each class registered under the Securities Act of 1933 are held of record by less than 300 persons; or

(2) At the beginning of such fiscal year, other than the fiscal year within which a registration statement under the Securities Act of 1933 became effective and the succeeding two fiscal years, all securities of each class registered under the Securities Act of 1933 are held of record by less than 500 persons and on

the last day of each of the issuer's three most recent fiscal years total assets of the issuer have not exceeded $3,000,000.

(b) If the duty of any issuer to file reports pursuant to section 15(d) of the Act as to any fiscal year is suspended in accordance with paragraph (a) (1) or (2) of this section, such issuer shall, within 30 days after the beginning of the first such fiscal year, file a notice on Form 15d-6 informing the Commission of such suspension. If the suspension resulted from the issuer's merger into, or consolidation with, another issuer or issuers, the notice shall be filed by the successor issuer. The notice shall be filed in addition to any other report required to be filed with the Commission in connection with the transaction or event giving rise to such suspension.

PART 249—FORMS, SECURITIES EXCHANGE ACT OF 1934

10. By revising § 249.323 to read as follows:

§ 249.323 Form 12g-4, certification of termination of registration of a class of security under section 12(g) of the Act.

This form shall be filed by each issuer to certify that the number of holders of record of a class of security registered under section 12(g) of the Act is reduced to less than 300 persons, or that the number of holders of record of a class of security registered under section 12(g) of the Act is reduced to less than 500 persons and the total assets of the issuer have not exceeded $3,000,000 on the last day of each of the issuer's three most recent fiscal years. Registration terminates 90 days after the filing of the certificate or within such shorter time as the Commission may direct. Copies of this form may be obtained from the Commission on request.

Note.—A binomial form for use also in reporting on Form 15d-6.

11. By revising § 249.333 to read as follows:

§ 249.333 Form 15d-6, for suspension of duty to file reports pursuant to section 15(d) of the Act.

This form shall be filed by each issuer required to file reports pursuant to section 15(d) of the Act, as a notification that the duty to file such reports is suspended because at the beginning of the fiscal year in which such reports would be required all securities of each class of such issuer registered under the Securities Act of 1933 are held of record by less than 300 persons or because all securities of each class registered under the Securities Act of 1933 are held of record by less than 500 persons and the total assets of the issuer have not exceeded $3,000,000 on the last day of each of the issuer's three most recent fiscal years. This form shall be filed within 30 days after the beginning of such fiscal year to which it pertains. Copies of this form may be obtained from the Commission on request.

Note.—A binomial form for use also in reporting on Form 12g-4.

12. Part 249—Forms, Securities Exchange Act of 1934, by revising the text of Form 12g-4/15d-6 to read as follows:

Sec File Number

U.S. Securities and Exchange Commission

Washington, D.C. 20549

Certification Pursuant to Rule 12g-4 or Notice Pursuant to Rule 15d-6 Under the Securities Exchange Act of 1934

(See General Instructions on Reverse Side of Form. Please Print or Type.)

Full name of registrant

Address of principal executive office (street, and number, city, State, ZIP code)

Please place an X in the appropriate box and furnish all information required by the form:

☐ Form 12g-4—Certification to terminate registration of a class of security under Sec. 12(g)

Title of Security covered by this certification ______

Approximate number of holders of record as of the certification date:

(Record Holders) ______

(Date) ______

Will registration under Sec. 12(g) remain in effect for any other class of equity security? ☐ Yes ☐ No

Will Section 15(d), subject to Rule 12h-4, become applicable because this class of securities has been offered pursuant to an effective Securities Act registration and the tests of Section 15(d) are met? ☐ Yes ☐ No

Please check the box corresponding to the provision under which this Form 12g-4 is being filed.

Holders of record below 300 persons. ☐

Holders of record below 500 persons and total assets within the Rule 12g-4(a)(2) limits. ☐

☐ Form 15d-6—Notice of suspension of duty to file reports pursuant to Sec. 15(d)

Title of Security covered by this notice ______

Approximate number of holders of record as of the beginning of the current fiscal year for which duty to file reports has been suspended:

(Record Holders) ______

(Date) ______

Please check the box corresponding to the provision under which this Form 15d-6 is being filed.

Holders of record below 300 persons. ☐

Holders of record below 500 persons and total assets within the Rule 15d-6(a)(2) limits. ☐

State below the date and nature of the transaction or event requiring or enabling the filing of this Form, including the name, address and fiscal year of any successor issuer:

Pursuant to the requirements of the Securities Exchange Act of 1934,

---

(Name of Registrant as Specified in Charter) has caused this certification/notice to be signed on its behalf by the undersigned duly authorized person.

Date: ______

By: ______

Instruction. The form may be signed by an officer of the registrant, by counsel or by any other duly authorized person. The name and title of the person signing the form shall be typed or printed under the signature.

1593 (4-82)

U.S. Securities and Exchange Commission

Washington, D.C. 20549

General Instructions

1. This Form is required by Rule 12g-4 and Rule 15d-6 of the General Rules and Regulations under the Securities Exchange Act of 1934, which state:

Rule 12g-4 Certifications of Termination of Registration Under Section 12(g)

(a) Termination of registration of a class of securities shall take effect in 90 days, or such shorter period as the Commission may determine, after the issuer certifies to the Commission on Form 12g-4/15d-6 that: (1) The number of holders of record of such class of securities is reduced to less than 300 persons; or (2) the number of holders or record of such class of securities is reduced to less than 500 persons and the total assets of the issuer have not exceeded $3,000,000 on the last day of each of the issuer's three most recent fiscal years.

(b) The issuer's duty to file any reports required under section 13(a) shall be suspended immediately upon certification on Form 12g-4/15d-6, *Provided, however*, That if the certification on Form 12g-4/15d-6 is subsequently withdrawn or denied, the registrant shall, within 60 days after the date of such withdrawal or denial, file with the Commission all reports which would have been required to be filed had the certification on Form 12g-4/15d-6 not been filed. If the issuer had merged into, or consolidated with another issuer or issuers, the form shall be filed by the successor issuer. The form shall be filed in addition to any other report required to be filed with the Commission in connection with the number of holders of record.

Rule 15d-6 Suspension of Duty To File Reports

(a) The duty of any issuer to file reports pursuant to section 15(d) of the Act for any fiscal year shall be suspended if,

(1) At the beginning of such fiscal year, other than the fiscal year within which such registration statement under the Securities Act of 1933 became effective, all securities of each class registered under the Securities Act of 1933 are held of record by less than 300 persons; or

(2) At the beginning of such fiscal year, other than the fiscal year within which a

registration statement under the Securities Act of 1933 became effective and the succeeding two fiscal years, all securities of each class registered under the Securities Act of 1933 are held of record by less than 500 persons and on the last day of each of the issuer's three most recent fiscal years total assets of the issuer have not exceeded $3,000,000.

(b) If the duty of any issuer to file reports pursuant to section 15(d) of the Act as to any fiscal year is suspended in accordance with paragraph (a)(1) or (2) of this section, such issuer shall, within 30 days after the beginning of the first such fiscal year, file a notice on Form 15d-6 informing the Commission of such suspension. If the suspension resulted from the issuer's merger into, or consolidation with, another issuer or issuers, the notice shall be filed by the successor issuer. The notice shall be filed in addition to any other report required to be filed with the Commission in connection with the transaction or event giving rise to such suspension.

2. Three copies of the form shall be filed with the Commission, one of which shall be manually signed. Copies not manually signed shall bear typed or printed signatures.

3. If the space provided on the form for setting forth the details of the transaction or event involving the decrease in the number of holders of record is insufficient, the complete answer should be prepared on a separate sheet and attached to the form. Reference thereto must be made on the form in the space provided for such details.

4. It should be noted that a company is required to file a notice on Form 15d-6 only for the first fiscal year in which the suspension is operative and not for each continuously successive fiscal year during which the suspension of the reporting requirements continues.

5. Any request for acceleration of the effective date of termination of registration under Section 12(g) and Rule 12g-4 shall be made in writing and filed with the Commission as a separate document and shall briefly describe the reasons therefore. Each request is considered on the basis of the facts and circumstances relevant to it.

[FR Doc. 82-10943 Filed 4-20-82; 8:45 am]
BILLING CODE 8010-01-M

DEPARTMENT OF ENERGY

Federal Energy Regulatory Commission

18 CFR Part 271

[Docket No. RM79-76-000 (Colorado-22); Order No. 223]

High-Cost Gas Produced From Tight Formations, Colorado; Final Rule

AGENCY: Federal Energy Regulatory Commission, DOE.

ACTION: Final rule.

SUMMARY: The Federal Energy Regulatory Commission is authorized by section 107(c)(5) of the Natural Gas Policy Act of 1978 to designated certain types of natural gas as high-cost gas where the Commission determines that the gas is produced under conditions which present extraordinary risks or costs. Under section 107(c)(5), the Commission issued a final regulation designating natural gas produced from tight formations as high-cost gas which may receive an incentive price (18 C.F.R. § 271.703). This rule established procedures for jurisdictional agencies to submit to the Commission recommendations of areas for designation as tight formations. This final order adopts the recommendation of the Colorado Oil and Gas Conservation Commission that the Mesaverde Formation, including the Rollins Member, and the Cozzette and Corcoran Members of the Upper Mancos Formation be designated as a tight formation under § 271.703(d).

EFFECTIVE DATE: This rule is effective April 15, 1982.

FOR FURTHER INFORMATION CONTACT: Leslie Lawner, (202) 357-8511 or Victor Zabel, (202) 357-8616.

SUPPLEMENTARY INFORMATION:

High-Cost Gas Produced From Tight Formations

Issued: April 15, 1982.

The Commission hereby amends § 271.703(d) of its regulations to include the Mesaverde Formation, including the Rollins Member and the Cozzette and Corcoran Members of the Upper Mancos Formation (hereinafter referred to as the Mesaverde Formation) located in Garfield, Mesa, Delta, Pitkin and Gunnison Counties, Colorado as a designated tight formation eligible for incentive pricing under § 271.703. The amendment was proposed in a Notice of Proposed Rulemaking by Director, OPPR, issued January 29, 1982 (47 FR 5237, February 4, 1982) [1] based on a recommendation by the Colorado Oil and Gas Conservation Commission (Colorado) in accordance with § 271.703(c) that the Mesaverde Formation, as described above, be designated as a tight formation.

Evidence submitted by Colorado supports the assertion that the Mesaverde Formation meets the guidelines contained in § 271.703(c)(2). Colorado reported in its submission that certain portions of the recommended area have been recommended as tight formations in previous applications submitted by Colorado, and that certain areas have been excluded from the instant recommendation because of infill drilling and gas storage utilization.

Furthermore, in accordance with Commission Order No. 205, issued January 22, 1982 in Docket No. RM79-76 (Colorado-17), the Naval Oil Shale Leaseholds are deleted from the area designated as a tight formation. These lands are under the jurisdiction of the U.S. Department of Energy, not Colorado or the Minerals Management Service of the U.S. Department of the Interior. This exclusion in no way forecloses the Department of the Energy from recommending this area as a tight formation under § 271.703. (See Appendix for Areas Excluded from Designation.) With the modifications thus listed, the Commission hereby adopts the Colorado recommendation.

This amendment shall become effective immediately. The Commission has found that the public interest dictates that new natural supplies be developed on an expedited basis, and, therefore, incentive prices should be made available as soon as possible. The need to make incentive prices available establishes good cause to waive the thrity-day publication period.

(Dept. of Energy Org. Act, 42 U.S.C. 7101 *et seq.;* Natural Gas Policy Act of 1978, 15 U.S.C. 3301-3432; Administrative Procedure Act, 5 U.S.C. 553)

List of Subjects in 18 CFR Part 271

Natural gas, incentive price, Tight formations.

In consideration of the foregoing, Part 271 of Subchapter H, Chapter I, Title 18, Code of Federal Regulations, is amended as set forth below, effective April 15, 1982.

By the Commission.

Kenneth F. Plumb,
Secretary.

PART 271—CEILING PRICES

Section 271.703 is amended by adding new paragraphs (d) (81), (82), and (83) to read as follows:

§ 271.703 Tight formations.

* * * * *

(d) *Designated tight formations.*

* * * * *

(81) *Mesaverde Formation (including the Rollins Member) in Colorado.* RM79-76 (Colorado—22).

(i) *Delineation of formation.* The Mesaverde Formation is located in the southeast portion of the Piceance Basin in Garfield, Mesa, Delta, Pitkin and Gunnison Counties, Colorado, approximately 15 miles northeast of the city of Grand Junction, Colorado. The

[1] Comments were invited and no comments were received. No party requested a hearing and no hearing was held.

Mesaverde Formation underlies Townships 6 through 11 South, Ranges 89 through 97 West, 6th P.M., with certain specified exclusions.

(ii) *Depth.* The Mesaverde Formation varies in thickness from zero to approximately 6,000 feet with its base defined as the bottom of the Rollins Members. The average depth to the top of the Mesaverde Formation is 5,641 feet.

(82) *Cozzette Formation in Colorado.* RM79-76 (Colorado—22).

(i) *Delineation of formation.* The Cozzette Formation is located in the southeast portion of the Piceance Basin in Garfield, Mesa, Delta, Pitkin and Gunnison Counties, Colorado, approximately 15 miles northeast of the city of Grand Junction, Colorado. The Cozzette Formation underlies Townships 6 through 11 South, Ranges 89 through 97 West, 6th P.M., with certain specified exclusions.

(ii) *Depth.* The Cozzette Formation is a member of the Upper Mancos Formation. The average depth to the top of the Cozzette Formation is 6,869 feet.

(83) *Corcoran Formation in Colorado.* RM79-76 (Colorado—22).

(i) *Delineation of formation.* The Corcoran Formation is located in the southeast portion of the Piceance Basin in Garfield, Mesa, Delta, Pitkin and Gunnison Counties, Colorado, approximately 15 miles from the city of Grand Junction, Colorado. The Corcoran Formation underlies Townships 6 through 11 South, Ranges 89 through 97, West, 6th P.M., with certain specified exclusions.

(ii) *Depth.* The Corcoran Formation is a member of the Upper Mancos Formation. The average depth to the top of the Corcoran Formation is 7,069 feet.

Appendix—Areas Not Covered by the Tight Formation Designation in Docket No. RM79-76 (Colorado—22)

(1) The area designated by the Commission as a tight formation in Docket No. RM79-76 (Colorado—5), Order No. 148, under § 271.703 for the Rollins, Corcoran and Cozzette Formation.

(2) Township 10 South, Range 95 West, 6th P.M., Sections 17, 18, 19, 30, and Township 10 South, Range 96 West, 6th P.M., Sections 12, 13, 23 through 28, 33m for the Rollins, Cozzette and Corcoran Formations. (Infill Drilling)

(3) Township 9 South, Range 97 West, 6th P.M., Sections 1 through 24, 26 through 35, and Township 10 South, Range 97 West, 6th P.M. Sections 2 through 11, for the Cozzette and Corcoran. (Designated in Order No. 151, Docket No. RM79-76 (Colorado—9))

(4) The area designated in Docket No. RM79-76 (Colorado—12) Order No. 156, under § 271.703 for the Cozzette, and Corcoran.

(5) Township 6 South, Range 93 West, 6th P.M., Sections 5, 6, 7, 8, 17, 18, 19, 20, and Township 6 South, Range 94 West, 6th P.M. Sections 1, 2, 3, 8 through 17, 19 through 24, 27 through 33, for the Mesaverde Formation. (Designated in Order No. 205, Docket No. RM79-76 (Colorado—17))

(6) The Wolf Creek Unit area. (Storage)

[FR Doc. 82-10891 Filed 4-20-82; 8:45 am]
BILLING CODE 6717-01-M

DEPARTMENT OF HOUSING AND URBAN DEVELOPMENT

Government National Mortgage Association

24 CFR Part 300

[Docket No. R-82-985]

List of Attorneys-in-Fact

AGENCY: Government National Mortgage Association, HUD.

ACTION: Final rule.

SUMMARY: Attorneys-in-fact are authorized to act for the Association by executing documents in its name in conjunction with servicing GNMA's mortgage purchase programs. This amendment updates the current list of attorneys-in-fact.

EFFECTIVE DATE: May 22, 1982.

ADDRESS: Rules Docket Clerk, Office of General Counsel, Room 5218, Department of Housing and Urban Development, 451 7th Street, SW., Washington, D.C. 20410.

FOR FURTHER INFORMATION CONTACT: Mr. William J. Linane, Office of General Counsel, on (202) 755-7186.

SUPPLEMENTARY INFORMATION: Notice and public procedure on this amendment are unnecessary and impracticable because of the large volume of legal documents that must be executed on behalf of the Association.

PART 300—GENERAL

§ 300.11 [Amended]

1. Paragraph (c) of § 300.11 is amended by adding the following names to the current list of attorneys-in-fact:

Name and Region

Stuart J. Jaffee, Philadelphia, PA
Edward H. Erickson, Chicago, IL
Victoria L. Arrington, Chicago, IL
Edward E. Czubernat, Chicago, IL
Nancy L. Webster, Chicago, IL
Carlton T. Foster, Jr., Atlanta, GA
Cynthia C. Estle, Atlanta, GA
R. Douglas Ezzell, Atlanta, GA

* * * * *

2. Paragraph (c) of § 300.11 is amended by removing the following names from the current list of attorneys-in-fact:

Name and Region

John Valentio, Philadelphia, PA
Ida M. Behling, Chicago, IL
Elaine Benes, Chicago, IL
Fred J. Jankowski, Chicago, IL
Francine A. Koch, Chicago, IL
Virginia K. O'Rourke, Chicago, IL
Cletus C. Parker, Chicago, IL
Florence Snuukst, Chicago, IL
Ada P. Tilford, Chicago, IL
Howard S. Carnes, Atlanta, GA
D. Keith Gettman, Atlanta, GA
Cynthia C. Anderson, Atlanta, GA

* * * * *

(Sec. 309(d), National Housing Act, 12 U.S.C. 1723a(d), and sec. 7(d), Department of Housing and Urban Development Act, 42 U.S.C. 3535(d))

Dated: April 7, 1982.

Warren A. Lasko,
Executive Vice President, Government National Mortgage Association.

[FR Doc. 82-10974 Filed 4-20-82; 8:45 am]
BILLING CODE 4210-01-M

ENVIRONMENTAL PROTECTION AGENCY

40 CFR Part 123

[SW-1-FRL—2107-2]

Hazardous Waste Management Program; Phase I Interim Authorization for Connecticut

AGENCY: Environmental Protection Agency (EPA).

ACTION: Authorization of State hazardous waste program.

SUMMARY: The State of Connecticut has applied for interim authorization of its hazardous waste program under Subtitle C of the Resource Conservation and Recovery Act and EPA guidelines for the approval of State hazardous waste programs (40 CFR Part 123). EPA has determined that the State's program meets all applicable statutory and regulatory requirements and is granting Phase I interim authorization to Connecticut to operate a hazardous waste program in its jurisdiction in lieu of Phase I of the Federal hazardous waste program.

EFFECTIVE DATE: April 21, 1982.

FOR FURTHER INFORMATION CONTACT: William R. Torrey, III, Waste Management Branch, U.S.E.P.A., Region

I, John F. Kennedy Federal Building, Boston, Massachusetts 02203; telephone (617) 223-5775.

SUPPLEMENTARY INFORMATION:

I. Introduction

Subtitle C of the Resource Conservation and Recovery Act of 1976, as amended, (RCRA) requires EPA to establish a comprehensive Federal program to assure the safe management of hazardous waste. Once a Federal program is established, EPA is authorized under Section 3006 of RCRA to approve State hazardous waste programs to operate in lieu of the Federal program in their jurisdictions.

Two types of State program approvals are authorized under RCRA. The first, "final authorization," is a permanent approval which may be granted to States whose programs are "equivalent to" and "consistent with" the Federal program and provide adequate enforcement. The second, "interim authorization," is a temporary approval for States which cannot meet the requirements of final authorization but whose programs are "substantially equivalent" to the Federal program. RCRA contemplates that States receiving interim authorization will use the interim authorization period to make the changes in their regulations and statutes necessary to qualify for final authorization.

On May 19, 1980, EPA published the first phase of the Federal hazardous waste program (40 CFR Parts 260-263 and 265) and guidelines for authorizing State hazardous waste programs under Section 3006 of RCRA (40 CFR Part 123). These guidelines set forth the requirements for interim authorization and the procedures which EPA will follow in acting on State applications for interim authorization. They also provide that EPA will grant interim authorization in two major phases (Phase I and Phase II), corresponding to the two major phases of the Federal program.

The State of Connecticut submitted a draft application for Phase I interim authorization on May 27, 1981. In its comments on the draft application, EPA identified several issues in the State's program description, memorandum of agreement, and Attorney General's statement which required further clarification or amendment. Most of these issues were addressed in the final application submitted to EPA on October 16, 1981, and the application was determined to be complete on that date.

In accordance with EPA guidelines, EPA provided public notice of receipt of the State's application and opportunity for comment on it. [46 FR 56464 (November 17, 1981)]. A hearing was held on the application on December 18, 1981, at Room 221, State Office Building in Hartford, Connecticut. No public comments on Connecticut's application were presented at the hearing or in writing by the close of the comment period on December 30, 1981.

II. Major Issues

No issues were raised by commenters on Connecticut's application.

III. Decision

EPA has reviewed the State of Connecticut's complete application for Phase I interim authorization and determined that the State program is "substantially equivalent" to the Phase I Federal program as defined in 40 CFR Part 123. In accordance with Section 3006(c) of RCRA, the State of Connecticut is hereby granted interim authorization to operate a hazardous waste program in lieu of Phase I of the Federal hazardous waste program. The practical effect of this decision is that generators, transporters, and owners and operators of existing hazardous waste management facilities in Connecticut will be subject to the State of Connecticut's hazardous waste management program and will not again be subject to the Phase I Federal program unless (1) the State fails to obtain final authorization by the deadline specified in section 3006(c) of RCRA and implementing regulations or (2) authorization is withdrawn for cause by EPA.

IV. Compliance with Executive Order 12291

The Office of Management and Budget has exempted this rule from the requirements of Section 3 of Executive Order 12291.

V. Certification under the Regulatory Flexibility Act

Subject: Connecticut Application for Interim Authorization, Certification Under the Regulatory Flexibility Act.

Pursuant to the provisions of 5 U.S.C. 605(b), I hereby certify that this authorization will not have a significant economic impact on a substantial number of small entities. The authorization suspends the applicability of certain Federal regulations in favor of the State program, thereby eliminating duplicative requirements for handlers of hazardous wastes in the State. It does not impose any new burdens on small entities. This rule, therefore, does not require a regulatory flexibility analysis.

VI. Authority

This notice is issued under the authority of Sections 2002(a), 3006, and 7004(b) of the Solid Waste Disposal Act, as amended by the Resource Conservation and Recovery Act of 1976, as amended, 42 U.S.C. 6912(a), 6926, and 6974(b).

List of Subjects in 40 CFR Part 123

Hazardous materials, Indians—lands, Reporting and recordkeeping requirements, Waste treatment and disposal, Water pollution control, Water supply, Intergovernmental relations, Penalties, Confidential business information.

Dated: March 30, 1982.

Lester A. Sutton,
Regional Administrator.

[FR Doc. 82-10855 Filed 4-20-82; 8:45 am]
BILLING CODE 6560-50-M

---

40 CFR Part 180

[PP 2F2586/R411; PH-FRL-2105-5]

Tolerances and Exemptions From Tolerances for Pesticide Chemicals in or on Raw Agricultural Commodities; Cyano(3-Phenoxyphenyl)Methyl 4-Chloro-Alpha-(1-Methyl-ethyl)Benzeneacetate

AGENCY: Environmental Protection Agency (EPA).

ACTION: Final rule.

SUMMARY: This rule establishes tolerances for residues of the insecticide cyano(3-phenoxyphenyl)methyl 4-chloro-alpha-(1-methylethyl)benzene-acetate in or on the raw agricultural commodities cucumbers and summer squash. This regulation to establish the maximum permissible level for the insecticide in or on the commodities was requested by Shell Oil Company.

EFFECTIVE DATE: April 21, 1982.

ADDRESS: Written objections may be submitted to the: Hearing Clerk (A-110), Environmental Protection Agency, Rm. 3708, 401 M St., SW., Washington, DC 20460.

FOR FURTHER INFORMATION CONTACT: Franklin D. R. Gee, Product Manager (PM) 17, Registration Division (TS-767C), Office of Pesticide Programs, Environmental Protection Agency, Rm. 207, CM#2, 1921 Jefferson Davis Highway, Arlington, VA 22202, (703-557-2690).

SUPPLEMENTARY INFORMATION: EPA issued a notice in the Federal Register of December 16, 1981 (46 FR 61331) which announced that Shell Oil Co., 1025

Connecticut Ave., NW., Suite 200, Washington, DC 20036, had filed a pesticide petition (PP 2F2586) with the EPA. The petition proposed that 40 CFR 180.379 be amended by establishing tolerances for residues of the insecticide cyano (3-phenoxyphenyl)methyl 4-chloro-alpha-(1-methylethyl)-benzeneacetate in or on the raw agricultural commodities cucumbers and summer squash at 0.5 part per million (ppm).

No comments were received in response to this notice of filing.

The data submitted in the petition and other relevant material have been evaluated. The toxicological data considered in support of the proposed tolerances included: An acute oral rat toxicity study with median lethal dose (LD_{50}) of 1–3 grams (g)/kilogram (kg) of body weight (bw) (water vehicle) and 450 milligrams (mg)/kg of bw (dimethylsulfoxide (DMSO) vehicle); a 90-day dog feeding study with a no-observed-effect level (NOEL) of 500 ppm (highest dose tested); a 90-day rat feeding study with a NOEL of 125 ppm; an 18-month mouse feeding study with a NOEL of less than 100 ppm with no oncogenic effects at the highest level fed (3,000 ppm); a 24-month mouse feeding study with a NOEL of 10–50 ppm for males and 50–250 ppm for females (no oncogenic effects were noted at 1,250 ppm, the highest dose tested); a 24-month rat feeding study that demonstrated no oncogenic effects at 1,000 ppm (only level tested—significantly decreased body weight was observed at this dose level); a 2-year rat feeding study with a NOEL of 250 ppm (highest level fed)—no oncogenic effects were observed; a 3-generation rat reproduction study with a NOEL of 250 ppm (highest level fed); teratology studies (in mice and rabbits, both negative at the highest dose of 50 mg/kg of bw/day); and the following mutagenicity studies: mouse dominant lethal (negative at 100 mg/kg of bw, which was the highest level fed); mouse host-mediated bioassay (negative at 50 mg/kg of bw, which was the highest level fed); AMES test in vitro (negative); and bone marrow cytogenic study in the Chinese hamster (negative at 25 mg/kg of bw). The following studies assessing neurological effects were performed: A hen study negative at 1.0 g/kg of bw for 5 days, repeated at 21 days; a rat (8-day) acute study with a NOEL of 200 mg/kg of bw; a 15-month rat feeding study which resulted in a systemic NOEL of 500 ppm and a NOEL of 1,500 ppm with respect to nerve damage.

The acceptable daily intake (ADI) is calculated to be 0.1250 mg/kg/day based on the 2-year rat feeding study and using a 100-fold safety factor. The maximum permissible intake (MPI) has been calculated to be 7.5000 mg/day (60 kg). Approval of the tolerances for cucumbers and summer squash would result in a theoretical maximum residue contribution (TMRC) of 1.4959 mg/day (1.5 kg) and utilize 19.95 percent of the ADI.

The metabolism of the insecticide is adequately understood for this use, and an adequate analytical method (gas chromatography) is available for enforcement purposes. There are currently no regulatory actions pending against the continued registration of this insecticide. Since no feed items are involved with this use there will be no problem of secondary residues in meat, milk, poultry, and eggs.

The pesticide is considered useful for the purpose for which the tolerances are sought, and it is concluded that establishment of the tolerances will protect the public health. Therefore, the tolerances are established as set forth below.

Any person adversely affected by this regulation may, on or before May 21, 1982, file written objections with the Hearing Clerk, at the address given above. Such objections should be submitted in quintuplicate and specify the provisions of the regulation deemed objectionable and the grounds for the objections. If a hearing is requested, the objections must state the issues for the hearing and the grounds for the objections. A hearing will be granted if the objections are supported by grounds legally sufficient to justify the relief sought.

The Office of Management and Budget has exempted this rule from the requirements of section 3 of Executive Order 12291.

Pursuant to the requirements of the Regulatory Flexibility Act (Pub. L. 96–534, 94 Stat. 1164, 5 U.S.C. 601–612), the Administrator has determined that regulations establishing new tolerances or raising tolerance levels or establishing exemptions from tolerance requirements do not have a significant economic impact on a substantial number of small entities. A certification statement to this effect was published in the **Federal Register** of May 4, 1981 (46 FR 24950).

Effective on: April 21, 1982.

(Sec. 408(d)(2), 68 Stat. 512 (21 U.S.C. 346a(d)(2)))

List of Subjects in 40 CFR Part 180

Administrative practice and procedure, Raw agricultural commodities, Pesticides and pests.

Dated: April 8, 1982.

Edwin L. Johnson,

Director, Office of Pesticide Programs.

PART 180—TOLERANCES AND EXEMPTIONS FROM TOLERANCES FOR PESTICIDE CHEMICALS IN OR ON RAW AGRICULTURAL COMMODITIES

Therefore, 40 CFR 180.379 is amended by adding and alphabetically inserting the raw agricultural commodities cucumbers and summer squash to read as follows:

§ 180.379 Cyano(3-phenoxyphenyl)methyl 4-chloro-alpha-(1-methylethyl)benzene-acetate; tolerances for residues.

* * * * *

Commodities	Parts per million
Cucumbers	0.5
* * * * *	
Summer squash	0.5
* * * * *	

[FR Doc. 82-10687 Filed 4-20-82; 8:45 am]

BILLING CODE 6560-50-M

40 CFR Part 180

[PP 9F2190/R424; PH-FRL-2105-4]

Norflurazon; Tolerances for Residues

AGENCY: Environmental Protection Agency (EPA).

ACTION: Final rule.

SUMMARY: This rule establishes tolerances for residues of the herbicide norflurazon in or on rotational and follow-up crops and for other indirect or inadvertent residues for norflurazon and its metabolite in or on certain agricultural follow-up crops from direct application to cotton. This regulation to establish the maximum permissible level for residues of the herbicide in or on the commodities was requested by Sandoz, Inc.

EFFECTIVE DATE: April 21, 1982.

ADDRESS: Written objections may be submitted to the: Hearing Clerk (A-110), Environmental Protection Agency, Rm. 3708, 401 M St., SW., Washington, DC 20460.

FOR FURTHER INFORMATION CONTACT: Richard F. Mountfort, Product Manager (PM) 23, Registration Division (TS-767C), Office of Pesticide Programs, Environmental Protection Agency, Rm. 237, CM#2, 1921 Jefferson Davis Highway, Arlington, VA 22202, (703-557-1830).

SUPPLEMENTARY INFORMATION: EPA issued a notice of proposed rulemaking published in the **Federal Register** of March 3, 1982 (47 FR 9025) which announced that Sandoz, Inc., 480 Camino del Rio South, Suite 204, San Diego, CA 92108, had submitted pesticide petition PP 9F2190 to the EPA proposing that tolerances be established for residues of the herbicide norflurazon [4-chloro-5-(methylamino)-2-(alpha, alpha, alpha-trifluoro-*m*-tolyl)-3(2*H*)-pyridazinone] and its desmethyl metabolite [4-chloro-5-amino-2-(alpha, alpha, alpha-trifluoro-*m*-tolyl)-3(2*H*)-pyridazinone) in or on peanuts at 0.2 part per million (ppm); and peanut hay, peanut hulls, and peanut vines at 0.5 ppm. These tolerances would cover residues resulting in peanuts as a follow-up crop when initial planting to cotton results in crop failure or a poor crop stand. No comments or requests for referral to an advisory committee were received in response to this notice of proposed rulemaking.

The data submitted in the petition and all other relevant material have been evaluated and discussed in the notice of proposed rulemaking (47 FR 9025, March 3, 1982). It is concluded that the establishment of these tolerances will protect the public health. Therefore, the tolerances are established as set forth below.

There are no regulatory actions pending against the chemical. The metabolism of norflurazon in plants and animals is adequately understood, and an analytical method, gas chromatography using an electron-capture detector, is available for enforcement purposes. The tolerances for various raw agricultural commodities, pesticide petition PP 9F2177 (47 FR 6894, February 17, 1982), will be adequate to cover residues that would result in meat, milk, or poultry as delineated in 40 CFR 180.6(a)(2).

Any person adversely affected by this regulation may, on or before May 21, 1982, file written objections with the Hearing Clerk, at the address given above. Such objections should be submitted in quintuplicate and specify the provisions of the regulation deemed objectionable and the grounds for the objections. If a hearing is requested, the objections must state the issues for the hearing and the grounds for the objections. A hearing will be granted if the objections are supported by grounds legally sufficient to justify the relief sought.

The Office of Management and Budget has exempted this rule from the requirements of section 3 of Executive Order 12291.

Effective on: April 21, 1982.

(Sec. 408(e), 68 Stat. 514 (21 U.S.C. 346(a)(e)))

List of Subjects in 40 CFR Part 180

Administrative practice and procedures, Agricultural commodities, Pesticides and pests.

Dated: April 7, 1982.

Edwin L. Johnson,

Director, Office of Pesticide Programs.

PART 180—TOLERANCES AND EXEMPTIONS FROM TOLERANCES FOR PESTICIDE CHEMICALS IN OR ON RAW AGRICULTURAL COMMODITIES

Therefore, 40 CFR 180.356 is amended by designating the existing text as paragraph (a) under the heading "Specific tolerances" and adding a new paragraph (b) under the heading "Indirect or inadvertent tolerances" to read as follows:

§ 180.356 Norflurazon; tolerances for residues.

(a) *Specific tolerances.* * * *

(b) *Indirect or inadvertent tolerances.* Tolerances are established for indirect residues of norflurazon in or on the following raw agricultural commodities when present therein as a result of its application to the growing of cotton as follows:

Commodities	Parts per million
Peanuts	0.2
Peanut, hay	0.5
Peanut, hulls	0.5
Peanut, vines	0.5

[FR Doc. 82-10889 Filed 4-20-82; 8:45 am]

BILLING CODE 6560-50-M

40 CFR Part 180

[PP 1F2540/R425; PH-FRL-2105-6]

Tolerances and Exemptions From Tolerances for Pesticide Chemicals in or on Raw Agricultural Commodities; Terbufos

AGENCY: Environmental Protection Agency (EPA).

ACTION: Final rule.

SUMMARY: This rule establishes tolerances for the combined residues of the insecticide terbufos and its cholinesterase-inhibiting metabolites in or on sorghum fodder, sorghum forage, and sorghum grain. This regulation to establish the maximum permissible level for the combined residues of the insecticide in or on the commodities was requested by American Cyanamide Co.

EFFECTIVE DATE: April 21, 1982.

ADDRESS: Written objections may be submitted to the: Hearing Clerk (A-110), Environmental Protection Agency, Rm. 3708, 401 M St., SW., Washington, D.C. 20460.

FOR FURTHER INFORMATION CONTACT: William H. Miller, Product Manager (PM) 16, Registration Division (TS-767C), Office of Pesticide Programs, Environmental Protection Agency, Rm. 211, CM#2, 1921 Jefferson Davis Highway, Arlington, VA 22202, (703-557-2600).

SUPPLEMENTARY INFORMATION: EPA issued a notice in the **Federal Register** of October 2, 1981 (46 FR 48755) which announced that American Cyanamide Co., PO Box 400, Princeton, NJ 08540, had filed a pesticide petition (PP 1F2540) with the EPA. The petition proposed that 40 CFR 180.352 be amended by establishing tolerances for the combined residues of the insecticide terbufos (*S*-[[1,1-dimethylethyl)thio]methyl]*O,O*-diethyl phosphorodithioate and its cholinesterase-inhibiting metabolites in or on the raw agricultural commodities sorghum fodder and sorghum forage at 0.5 part per million (ppm) and sorghum grain at 0.05 ppm. No comments were received in response to this notice of filing.

The data submitted in the petition and all other relevant material have been evaluated. The toxicology data considered in support of the proposed tolerances were: A 6-month dog feeding study with a no-observed-effect level (NOEL) of 0.0025 milligrams (mg)/kilogram (kg)/day (0.1 ppm) for cholinesterase inhibition and systemic toxicity observed at 0.04 mg/kg/day (1.6 ppm); a 90-day oral rat feeding study with a NOEL of 0.25 ppm for effects on red blood count and brain cholinesterase; a delayed neurotoxicity study in chickens which was negative at 0.01 mg/kg; a teratogenicity study in rats which was negative at 0.15 mg/kg; a 3-generation reproduction study in rats with effects observed at 1.0 ppm; and a mutagenicity Ames test including metabolic activation which was negative.

Desirable data that are currently lacking include: a teratology study in a second species; a 2-year chronic rat feeding/oncogenicity study; and an 18-month or longer mouse oncogenicity study; and mutagenicity studies [rat hepatocytes DNA repair test (HPC-UDS) and mammalian cell transformation assay].

Although there are significant data gaps for the chemical, the available toxicity data are adequate to support the

proposed tolerances because the proposed use will result in an insignificant increase (0.4348 percent) in the theoretical maximum residue contribution (TMRC) to the human diet. As stated in the Federal Register of May 11, 1979 (44 FR 27932-27936), the Agency will generally consider as insignificant an increase in the TMRC of 1.0 percent or less.

No secondary residues are likely to occur in eggs, milk, meat, and poultry as delineated in § 180.6(a)(3). An adequate analytical method (gas chromatographic procedure equipped with a flame photometric detector in the phosphorous mode) is available for enforcement purposes. The nature of the residues is adequately understood.

Tolerances have previously been established for the combined residues of terbufos and its cholinesterase-inhibiting metabolites in or on sugar beets (roots at 0.05 ppm and tops at 0.1 ppm); on the forage and fodder of field, pop, and sweet corn at 0.5 ppm; and in or on grain corn and sweet corn at 0.05 ppm (40 CFR 180.352). There are no regulatory actions pending against continued registration of the pesticide and there are no other considerations involved in establishing the tolerances.

The pesticide is considered useful for the purpose for which the tolerances are sought and it is concluded that establishment of the tolerances will protect the public health. Therefore, 40 CFR 180.352 is amended as set forth below.

Any person adversely affected by this regulation may, on or before May 21, 1982, file written objections with the Hearing Clerk, at the address given above. Such objections should be submitted in quintuplicate and specify the provisions of the regulation deemed objectionable and the grounds for the objections. If a hearing is requested, the objections must state the issues for the hearing and the grounds for the objections. A hearing will be granted if the objections are supported by grounds legally sufficient to justify the relief sought.

The Office of Management and Budget has exempted this rule from the requirements of section 3 of Executive Order 12291.

Pursuant to the requirements of the Regulatory Flexibility Act (Pub. L. 96-534, 94 Stat. 1164, 5 U.S.C. 601-612), the Administrator has determined that regulations establishing new tolerances or raising tolerance levels or establishing exemptions from tolerance requirements do not have a significant economic impact on a substantial number of small entities. A certification statement to this effect was published in the Federal Register of May 4, 1981 (46 FR 24950).

Effective on: April 21, 1982.

(Sec. 408(d)(2), 68 Stat. 512 (21 U.S.C. 346a(d)(2)))

List of Subjects in 40 CFR Part 180

Administrative practice and procedure, Agricultural commodities, Pesticides and pests.

Dated: April 9, 1982.

Edwin L. Johnson,

Director, Office of Pesticide Programs.

PART 180—TOLERANCES AND EXEMPTIONS FROM TOLERANCES FOR PESTICIDE CHEMICALS IN OR ON RAW AGRICULTURAL COMMODITIES

Therefore, 40 CFR 180.352 is amended by adding and alphabetically inserting the raw agricultural commodities sorghum fodder, sorghum forage, and sorghum grain to read as follows:

§ 180.352 Terbufos; tolerances for residues.

* * * * *

Commodities	Parts per million
* * * * *	*
Sorghum, fodder	0.5
Sorghum, forage	0.5
Sorghum, grain	0.05
* * * * *	

[FR Doc. 82-10686 Filed 4-20-82; 8:45 am]

BILLING CODE 6560-50-M

40 CFR Part 256

[SW-1-FRL—2106-8]

Approval of Massachusetts Solid Waste Management Plan

AGENCY: Environmental Protection Agency.

ACTION: Final rule.

SUMMARY: As provided by the Solid Waste Disposal Act, as amended by the Resource Conservation and Recovery Act of 1976 (RCRA), the State of Massachusetts has received Federal financial assistance for development of a State solid waste management plan. The State of Massachusetts has submitted to the U.S. Environmental Protection Agency (EPA or the Agency) its adopted State solid waste management plan. Today, EPA is announcing its approval of the Massachusetts solid waste management plan. Approval of the plan indicates that it meets the requirements set forth in RCRA, which provides for the identification of State, local and regional responsibilities for solid waste management; the encouragement of resource conservation and recovery; and the development and application of State controls to provide for environmentally sound solid waste disposal practices.

The purpose of this notice is to inform the public that the Agency is approving the Massachusetts solid waste management plan.

EFFECTIVE DATE: April 21, 1982.

FOR FURTHER INFORMATION CONTACT: Conrad O. Desrosiers, Chief, Solid Waste Section, U.S. EPA, Region I, JFK Federal Building, Boston, MA 02203 (617) 223-5775.

SUPPLEMENTARY INFORMATION:

Background

On July 31, 1979, (44 FR 45066) EPA published Guidelines for the Development and Implementation of State Solid Waste Management Plans. These guidelines were required by section 4002(b) of the Solid Waste Disposal Act, as amended by the Resource Conservation and Recovery Act of 1976 (RCRA).

The guidelines reflect the minimum statutory requirements for the State plans and recommend methods and procedures to meet those requirements. Under section 4007 of RCRA, the Administrator approves State plans which meet the requirements of paragraphs (1), (2), (3) and (5) of section 4003 of RCRA and which contain provisions for revision. Briefly, these requirements are:

1. The plan shall identify the responsibilities of State, local and regional authorities in the implementation of the State plan; the distribution of Federal funds to the authorities responsible for development and implementation of the State plan; and the means for coordinating regional planning and implementation under the State plan;

2. The plan shall prohibit the establishment of new open dumps within the State and contain requirements that all solid waste be utilized for resource recovery or disposed of in sanitary landfills, as defined by section 4004(a) of RCRA, or otherwise disposed of in an environmentally sound manner. The State prohibition must be effective as of the date on which EPA approves the plan;

3. The plan shall provide for the closing or upgrading of all existing open dumps within the State;

4. The plan shall provide that no local government within the State shall be prohibited under State or local law from entering into long term contracts for supply of solid waste to resource recovery facilities; and

5. The plan must contain specific provisions for revision.

The guidelines also addressed section 4005 of RCRA which requires a mechanism in the State plan for establishment of compliance schedules for entities engaged in the prohibited act of open dumping. The plan must provide that, in attempting to obtain such compliance schedules, entities must demonstrate their inability to utilize other public or private alternatives to comply with the prohibition.

Response to Comments

On November 4, 1981 at (46 FR 54772) the Massachusetts solid waste management plan was noticed for public review and comment. No substantive comments were received during the 30 day review period.

Finding

I have reviewed the solid waste management plan submitted by the State of Massachusetts for approval.

I find that the plan meets the requirements of RCRA for approval. Under authority of Section 4007 of RCRA, I approve the Massachusetts solid waste management plan.

The plan prohibits the establishment of open dumps, and the state prohibition was in effect on April 21, 1982.

Also, the plan provides for compliance schedules for entities engaged in open dumping where those entities can demonstrate that they are unable to utilize other public or private alternatives for solid waste management to comply with the RCRA prohibition of open dumping. As of this date entities engaged in open dumping may, pursuant to the plan, approach the State for further information on compliance schedules and necessary demonstrations. Parties that receive compliance schedules satisfying section 4005 from EPA approved States and that are in compliance with these schedules are not in violation of the open dumping prohibition in Section 4005. Such compliance schedules cannot extend beyond September 13, 1984.

Compliance With Executive Order 12291

The Office of Management and Budget has exempted this rule from the requirements of Section 3 of Executive Order 12291.

Certification Under the Regulatory Flexibility Act

I certify under 5 U.S.C. 605(b) that the approval of the Massachusetts solid waste management plan will not have a significant economic impact on a substantial number of small entities. This Approval will reduce burdens on small entities by establishing a mechanism to insulate them from citizen suits to enforce the open dumping prohibition. This rule, therefore, does not require a regulatory flexibility analysis.

List of Subjects in 40 CFR Part 256

Grant programs—environmental protection, Waste treatment and disposal.

(Sec. 4007(a), Pub. L. 94-580, 90 Stat. 2817 (42 U.S.C. 6947))

Dated: April 12, 1982.

Leslie Carothers,

Acting Regional Administrator.

[FR Doc. 82-10856 Filed 4-20-82; 8:45 am]

BILLING CODE 6560-50-M

DEPARTMENT OF THE INTERIOR

Bureau of Land Management

43 CFR Public Land Order 6238

[OR-19014, OR-19113, OR-19115, OR-19116]

Oregon; Powersite Restoration Nos. 662, 718, 747, and 756; Partial Revocation of Powersite Reserve Nos. 659, 661, and 662; Partial Revocation of Water Power Designation No. 14

AGENCY: Bureau of Land Management, Interior.

ACTION: Public Land Order.

SUMMARY: This order revokes an Executive order and a Secretarial order in part as to 4,132.47 acres of land withdrawn for three powersite reserves and a water power designation. This action will restore 3,844.74 acres of land to operation of the public land laws generally. The balance of 287.73 acres remains segregated by existing withdrawals.

EFFECTIVE DATE: May 18, 1982.

FOR FURTHER INFORMATION CONTACT: Champ C. Vaughan, Jr., Oregon State Office, 503-231-6905.

SUPPLEMENTARY INFORMATION: By virtue of the authority vested in the Secretary of the Interior by Section 204 of the Federal Land Policy and Management Act of 1976, 90 Stat. 2751; 43 U.S.C. 1714, and pursuant to the determination by the Federal Energy Regulatory Commission in DA-540-Oregon, DA-543-Oregon, DA-557-Oregon, and DA-563-Oregon, it is ordered as follows:

1. The Executive Order of December 12, 1917, which created Powersite Reserve Nos. 659, 661, and 662, and the Secretarial Order of December 12, 1917, which created Water Power Designation No. 14, are hereby revoked insofar as they affect the following described lands:

Willamette Meridian

Powersite Reserve No. 662

T. 16 S., R. 7 W.,
 Sec. 18, Lot 2.
T. 18 S., R. 8 W.,
 Sec. 28, SE¼SE¼.

Water Power Designation No. 14

T. 2 S., R. 6 E.,
 Sec. 25, N½NE¼.
T. 2 S., R. 7 E.,
 Sec. 31, N½NE¼ and NE¼NW¼.

Siuslaw National Forest

T. 16 S., R. 8 W.,
 Sec. 7, Lots 2 and 4, and NW¼NE¼;
 Sec. 19, Lot 3 and NE¼NW¼.

Water Power Designation No. 14

Powersite Reserve No. 659

T. 19 S., R. 6 W.,
 Sec. 17, NE¼NE¼.
T. 16 S., R. 7 W.,
 Sec. 31, Lot 3.
T. 16 S., R. 8 W.,
 Sec. 13, Lots 15 and 16.
T. 17 S., R. 8 W.,
 Sec. 1, SW¼NW¼.

Revested Oregon and California Railroad Grant Land

Powersite Reserve No. 659; Water Power Designation No. 14

T. 19 S., R. 6 W.,
 Sec. 7, S½SE¼;
 Sec. 17, NW¼NE¼ and N½NW¼;
 Sec. 31, SE¼SW¼.
T. 20 S., R. 6 W.,
 Sec. 1, W½SE¼;
 Sec. 5, SW¼NW¼;
 Sec. 9, Lot 9;
 Sec. 11, E½NW¼.
T. 16 S., R. 7 W.,
 Sec. 7, Lots 3 and 4, E½SW¼, W½SE¼, and SE¼SE¼;
 Sec. 19, NE¼SW¼;
 Sec. 21, Lots 4 and 5;
 Sec. 29, NW¼NW¼;
 Sec. 31, NW¼NE¼.
T. 19 S., R. 7 W.,
 Sec. 5, Lot 1 and SE¼NE¼;
 Sec. 7, NE¼NE¼;
 Sec. 19, Lot 1.
T. 20 S., R. 7 W.,
 Sec. 3, Lots 10, 11, and 12.
T. 16 S., R. 8 W.,
 Sec. 13, Lot 9;
 Sec. 25, Lots 25, 31, and 32;
 Sec. 35, Lots 1 and 2, SE¼NE¼, NW¼NW¼, and NW¼SE¼.
T. 17 S., R. 8 W.,
 Sec. 1, NW¼SW¼;

Sec. 3, S½SW¼ and SW¼SE¼;
Sec. 5, SE¼SE¼;
Sec. 17, SW¼NW¼.
T. 18 S., R. 8 W.,
Sec. 9, SE¼SW¼ and SW¼SE¼;
Sec. 17, N½SW¼;
Sec. 21, W½SW¼.
T. 19 S., R. 8 W.,
Sec. 3, Lot 10;
Sec. 11, SE¼SE¼.
T. 30 S., R. 9 W.,
Sec. 9, S½SE¼;
Sec. 17, S½NW¼.

Water Power Designation No. 14

Powersite Reserve No. 661

T. 18 S., R. 1 W.,
Sec. 25, SE¼SE¼.
T. 16, S., R. 8 W.,
Sec. 27, Lots 17, 18, 23, 24, 25, 26, 31, and 32.
T. 18 S., R. 1 E.,
Sec. 19, N½S½ and S½SE¼;
Sec. 21, N½NE¼, SW¼NE¼, and N½SW¼;
Sec. 25, NW¼SE¼ and S½SE¼.

Water Power Designation No. 14

T. 2 S., R. 6 E.,
Sec. 21, SE¼SW¼ and S½SE¼.
T. 2 S., R. 7 E.,
Sec. 31, S½NE¼.
The areas described aggregate 4,132.47 acres in Coos, Douglas, and Lane Counties.

2. The State of Oregon has waived its preference right for highway rights-of-way or material sites as provided by the Federal Power Act of June 10, 1920, 16 U.S.C. 818.

3. The land in the S½SE¼, Section 9, T. 30 S., R. 9 W., is withdrawn as a recreation area and remains segregated from operation of the public land laws, including the United States mining laws.

4. The lands in Lots 2, Section 18, T. 16 S., R. 7 W., Lots 15 and 16, Section 12, T. 16 S., R. 8 W., SW¼NW¼, Section 1, T. 17 S., R. 8 W., and SE¼SE¼, Section 28, T. 18 S., R. 8 W., are withdrawn for multiple use management by Public Land Order No. 5490 of February 12, 1975, and therefore remain segregated from operation of the public land laws generally.

5. At 10 a.m., on May 18, 1982, subject to valid existing rights, the provisions of existing withdrawls, and the requirements of applicable law, the above described lands, except as provided in paragraphs 3 and 4, will be open to such forms of disposition as may by law be made of National Forest lands and Revested Oregon and California Railroad Grant Land.

6. The above described lands, except as provided in paragraph 3, have been open to applications and offers under the mineral leasing laws and to location under the United States mining laws subject to the provisions of the Act of August 11, 1955 (69 Stat. 682; 30 U.S.C. 621).

Inquiries concerning the lands should be addressed to the State Director, Bureau of Land Management, P.O. Box 2965, Portland, Oregon 97208.

Garrey E. Carruthers,
Assistant Secretary of the Interior.
April 12, 1982.
[FR Doc. 82-10938 Filed 4-20-82; 8:45 am]
BILLING CODE 4310-84-M

---

43 CFR Public Land Order 6239

[AA-2135]

Alaska; Opening of Lands Subject to Section 24 of the Federal Power Act

AGENCY: Bureau of Land Management, Interior.

ACTION: Public land order.

SUMMARY: This public land order will restore an area aggregating 120 acres of land near Haines, Alaska, within Powersite Classification No. 439 of July 10, 1957, pursuant to the determination issued August 29, 1967, by the Federal Power Commission and pursuant to Final Judgment entered on October 30, 1980, in the United States District Court for the District of Alaska in "Reeves v. Andrus", Civ. No. A158-73.

EFFECTIVE DATE: April 21, 1982.

FOR FURTHER INFORMATION CONTACT: Beau McClure, Washington, D.C., 202-343-6511, or Robert Sorenson, Bureau of Land Management, 701 C Street, Box 13, Anchorage, Alaska 99513, 907-271-5060.

SUPPLEMENTARY INFORMATION: By virtue of the authority contained in Section 24 of the Federal Power Act of June 10, 1920, 41 Stat. 1075, as amended, 16 U.S.C. 818, and in accordance with the authority vested in the Secretary of the Interior by Section 204 of the Federal Land Policy and Management Act of 1976, 90 Stat. 2751; 43 U.S.C. 1714, it is ordered as follows:

1. In DA-91-Alaska, the Federal Power Commission determined that the power values of the following described lands, withdrawn in Powersite Classification No. 439 of July 10, 1957, will not be injured or destroyed by restoration to location, entry, or selection under appropriate public land laws, subject to the provisions of Section 24 of the Federal Power Act:

Beginning at Corner No. 1, in what when surveyed will be approximately sec. 4, T. 29 S., R. 58 E., *which* is submerged point in the center of Chilkoot River at the point on said Chilkoot River where a certain unnamed creek enters said Chilkoot River from the west, and from which point that a certain cabin on the head of Chilkoot Lake known as the "Company 'F'—32nd Infantry Cabin," bears approximately S. 20° E., a distance of approximately 6,380 ft., and from which that certain mountain in sec. 32, T. 28 S., R. 58 E., with an elevation of 4,950 ft., bears approximately E. 20° N., at a distance of approximately 2½ miles thence W. 2,640 ft., to Corner No. 2; thence S. 1,980 ft., to Corner No. 3; thence E. 1,320 ft., to Corner No. 4; thence S. 1,320 ft., to Corner No. 5; thence E. 1,320 ft., to said Corner No. 6; thence N. 3,300 ft., to said Corner No. 1, the point of beginning.

Which, if surveyed, would probably be described as follows:

Copper River Meridian

T. 29 S., R. 58 E.,
Sec. 4, NE¼SW¼, W½SE¼.
The area described aggregates 120 acres near Haines, Alaska.

2. The lands are hereby restored to operation of the public land laws generally, subject to the provisions of Section 24 of the Federal Power Act, *supra,* the requirements of applicable law, and valid existing rights, including but not limited to the valid homestead settlement location of Henry Reeves and all rights thereunto accruing under the Final Judgment entered on October 30, 1980, in "Reeves v. Andrus", Civ. No. A158-73 (D. Alas. Oct. 30, 1980).

3. The State of Alaska has waived the preference right of selection granted to it by the Act of July 7, 1958, 72 Stat. 339-352.

The lands have been open to applications and offers under the mineral leasing laws, and to location under the United States mining laws subject to the provisions of the Act of August 11, 1955, 69 Stat. 682; 30 U.S.C. 621.

Inquiries concerning the lands should be addressed to the Bureau of Land Management, 701 C Street, Box 13, Anchorage, Alaska 99513.

Garrey E. Carruthers,
Assistant Secretary of the Interior.
April 12, 1982.
[FR Doc. 82-10942 Filed 4-20-82; 8:45 am]
BILLING CODE 4310-84-M

FEDERAL EMERGENCY MANAGEMENT AGENCY

44 CFR Part 70

[Docket No. FEMA-5909]

Letter of Map Amendment for Santa Barbara County, Calif., Under National Flood Insurance Program

AGENCY: Federal Emergency Management Agency.

ACTION: Final rule.

SUMMARY: The Federal Emergency Management Agency (FEMA) published a list of communities for which maps

identifying Special Flood Hazard Areas have been published. This list included Santa Barbara County. It has been determined by the Associate Director, State and Local Programs and Support, after acquiring additional flood information and after further technical review of the Flood Insurance Rate Map for Santa Barbara County, that certain property is not within the Special Flood Hazard Area.

This map amendment, by establishing that the subject property is not within the Special Flood Hazard Area, removes the requirement to purchase flood insurance for that property as a condition of Federal or federally-related financial assistance for construction or acquisition purposes.

EFFECTIVE DATE: April 21, 1982.

FOR FURTHER INFORMATION CONTACT: Mr. Robert G. Chappell, P.E., Chief, Engineering Branch, Natural Hazards Division, Federal Emergency Management Agency, Washington, DC 20472, (202) 287-0230.

SUPPLEMENTARY INFORMATION: If a property owner was required to purchase flood insurance as a condition of Federal or federally-related financial assistance for construction or acquisition purposes, and the lender now agrees to waive the property owner from maintaining flood insurance coverage on the basis of this map amendment, the property owner may obtain a full refund of the premium paid for the current policy year, provided that no claim is pending or has been paid on the policy in question during the same policy year. The premium refund may be obtained through the insurance agent or broker who sold the policy, or from the National Flood Insurance Program (NFIP) at: P.O. Box 34294, Bethesda, Maryland 20034, Telephone: (800) 638-6620.

The map amendments listed below are in accordance with S 70.7(b):

Map No. H & I 060331 Panel 0755B, published on October 6, 1980, in 45 FR 66119, indicates that portions of Lots 185 and 186, Outside Pueblo Land, Santa Barbara County, California, recorded as Instrument Number 79-58348, in the Office of the Clerk and Recorder, Santa Barbara County, California, is within the Special Flood Hazard Area.

Map No. H & I 060331 Panel 0755B is hereby corrected to reflect that the existing structure located on the above mentioned property is not within the Special Flood Hazard Area identified on March 15, 1979. This structure is in Zone C.

Pursuant to the provisions of 5 U.S.C. 605(b), the Associate Director, State and Local Programs and Support to whom authority has been delegated by the Director, Federal Emergency Management Agency, hereby certifies that this rule if promulgated will not have a significant economic impact on a substantial number of small entities. This rule provides routine legal notice of technical amendments made to designated special flood hazard areas on the basis of updated information and imposes no new requirements or regulations on participating communities.

List of Subjects in 44 CFR Part 70

Flood insurance, Floodplains.

(National Flood Insurance Act of 1968 (Title XIII of Housing and Urban Development Act of 1968), effective January 28, 1969 (33 FR 17804, November 28, 1968), as amended; 42 U.S.C. 4001-4128; Executive Order 12127, 44 FR 19367; delegation of authority to Associate Director, State and Local Programs and Support)

Issued: March 23, 1982.

Lee M. Thomas,

Associate Director, State and Local Programs and Support.

[FR Doc. 82-10894 Filed 4-20-82; 8:45 am]

BILLING CODE 6718-03-M

---

44 CFR Part 70

[Docket No. FEMA-5909]

Letter of Map Amendment for the City of Waveland, Miss., Under National Flood Insurance Program

AGENCY: Federal Emergency Management Agency.

ACTION: Final rule.

SUMMARY: The Federal Emergency Management Agency published a list of communities for which maps identifying Special Flood Hazard Areas have been published. This list included the City of Waveland, Mississippi. It has been determined by the Associate Director, State and Local Programs and Support after acquiring additional flood information and after further technical review of the Flood Insurance Rate Map for the City of Waveland, Mississippi, that certain property is not within the Special Flood Hazard Area.

This map amendment, by establishing that the subject property is not within the Special Flood Hazard Area, removes the requirement to purchase flood insurance for that property as a condition of Federal or federally-related financial assistance for construction or acquisition purposes.

EFFECTIVE DATE: April 21, 1982.

FOR FURTHER INFORMATION CONTACT: Mr. Robert G. Chappell, Chief, Engineering Branch, Natural Hazards Division, Federal Emergency Management Agency, Washington, D.C. 20472, (202) 287-0230.

SUPPLEMENTARY INFORMATION: If a property owner was required to purchase flood insurance as a condition of Federal or federally-related financial assistance for construction or acquisition purposes, and the lender now agrees to waive the property owner from maintaining flood insurance coverage on the basis of this map amendment, the property owner may obtain a full refund of the premium paid for the current policy year, provided that no claim is pending or has been paid on the policy in question during the same policy year. The premium refund may be obtained through the insurance agent or broker who sold the policy, or from the National Flood Insurance Program (NFIP): P.O. Box 34294, Bethesda, Maryland 20034, Phone: (800) 638-6620.

The map amendments listed below are in accordance with § 70.7(b):

Map Number H & I 285262 A, Panel 04 published on October 6, 1980 in 45 FR 66066 indicates that the southern 4.97 acres of a parcel of land located in the City of Waveland, Mississippi, owned by the Donegan Development Corporation of which Joseph J. Kirkland has an option to purchase, being recorded in Deed Book AA 51, Pages 261, 262, 632 and 633 in the Chancery Court Clerk's Office in Hancock County, Mississippi are located within the Special Flood Hazard Area.

Map Number H & I 285262 A, Panel 04 is hereby corrected to reflect that the above-mentioned property is not located within the Special Flood Hazard Area as identified on April 16, 1976. The property is located in Zone B.

Pursuant to the provisions of 5 U.S.C. 605(b), the Associate Director, State and Local Programs and Support, to whom authority has been delegated by the Director, Federal Emergency Management Agency, hereby certifies that this rule if promulgated will not have a significant economic impact on a substantial number of small entities. This rule provides routine legal notice of technical amendments made to designated special flood hazard areas on the basis of updated information and imposes no new requirements or regulations on participating communities.

List of Subjects in 44 CFR Part 70

Flood insurance, Floodplains.

(National Flood Insurance Act of 1968 (Title XIII of Housing and Urban Development Act of 1968), effective January 28, 1969 (33 FR 17804, November 28, 1968), as amended; 42 U.S.C. 4001-4128; Executive Order 12127, 44

FR 19367; delegation of authority to Associate Director, State and Local Programs and Support)

Issued: March 22, 1982.

Lee M. Thomas,

Associate Director, State and Local Programs and Support.

[FR Doc. 82-10899 Filed 4-20-82; 8:45 am]

BILLING CODE 6718-03-M

44 CFR Part 70

[Docket No. FEMA-5909]

Letter of Map Amendment for the Township of Chatham, N.J., Under National Flood Insurance Program

AGENCY: Federal Emergency Management Agency.

ACTION: Final rule.

SUMMARY: The Federal Emergency Management Agency published a list of communities for which maps identifying Special Flood Hazard Areas have been published. This list included the Township of Chatham, New Jersey. It has been determined by the Associate Director, State and Local Programs and Support after acquiring additional flood information and after further technical review of the Flood Insurance Rate Map for the Township of Chatham, New Jersey, that certain property is and certain property is not within the Special Flood Hazard Area.

This map amendment, by establishing that the subject property is and is not within the Special Flood Hazard Area, removes and enforces the requirement to purchase flood insurance for that property as a condition of Federal or federally-related financial assistance for construction or acquisition purposes.

EFFECTIVE DATE: April 21, 1982.

FOR FURTHER INFORMATION CONTACT: Mr. Robert G. Chappell, Chief, Engineering Branch, Natural Hazards Division, Federal Emergency Management Agency, Washington, D.C. 20472, (202) 287-0230.

SUPPLEMENTARY INFORMATION: If a property owner was required to purchase flood insurance as a condition of Federal or federally-related financial assistance for construction or acquisition purposes, and the lender now agrees to waive the property owner from maintaining flood insurance coverage on the basis of this map amendment, the property owner may obtain a full refund of the premium paid for the current policy year, provided that no claim is pending or has been paid on the policy in question during the same policy year. The premium refund may be obtained through the insurance agent or broker who sold the policy, or from the National Flood Insurance Program (NFIP): P.O. Box 34294, Bethesda, Maryland 20034, Phone: (800) 638-6620.

The map amendments listed below are in accordance with § 70.7(b):

Map Number H & I 340504, Panel 0004 B published on October 6, 1980 in FR 66027 indicates that the structures on Lots 6, 8, and 9, Block 48-0, Stonewyck at Chatham, in the Township of Chatham, New Jersey, and recorded as Map Number 3353 in the Office of the Clerk of Morris County, New Jersey, are located within the Special Flood Hazard Area.

In addition, the above-mentioned Map and Panel Number indicates that the structure located on Lot 5, of the above-mentioned property is located in Zone C.

Map Number H & I 340504, Panel 0004 B is hereby corrected to reflect that the structures located on Lots 6, 8, and 9 are not within the Special Flood Hazard Area identified on May 15, 1980. The structures are in Zone C. However, portions of the property would still be inundated by a flood having a one-percent chance of occurrence in any given year (base flood).

Furthermore, the above-mentioned Map and Panel Number is hereby corrected to reflect that the structure located on Lot 5 is in Zone A.

Pursuant to the provisions of 5 U.S.C. 605(b), the Associate Director, State and Local Programs and Support, to whom authority has been delegated by the Director, Federal Emergency Management Agency, hereby certifies that this rule if promulgated will not have a significant economic impact on a substantial number of small entities. This rule provides routine legal notice of technical amendments made to designated special flood hazard areas on the basis of updated information and imposes no new requirements or regulations on participating communities.

List of Subjects in 44 CFR Part 70

Flood insurance, Floodplains.

(National Flood Insurance Act of 1968 (Title XIII of Housing and Urban Development Act of 1968), effective January 28, 1969 (33 FR 17804, November 28, 1968), as amended; 42 U.S.C. 4001-4128; Executive Order 12127, 44 FR 19367; delegation of authority to Associate Director, State and Local Programs and Support)

Issued: March 23, 1982.

Lee M. Thomas,

Associate Director, State and Local Programs and Support.

[FR Doc. 82-10895 Filed 4-20-82; 8:45 am]

BILLING CODE 6718-03-M

44 CFR Part 70

[Docket No. FEMA-5952]

Letter of Map Amendment for the City of Forest Acres, S.C. Under National Flood Insurance Program

AGENCY: Federal Emergency Management Agency.

ACTION: Final rule.

SUMMARY: The Federal Emergency Management Agency published a list of communities for which maps identifying Special Flood Hazard Areas have been published. This list included the City of Forest Acres, South Carolina. It has been determined by the Associate Director, State and Local Programs and Support after acquiring additional flood information and after further technical review of the Flood Insurance Rate Map for the City of Forest Acres, South Carolina, that certain property is not within the Special Flood Hazard Area.

This map amendment, by establishing that the subject property is not within the Special Flood Hazard Area, removes the requirement to purchase flood insurance for that property as a condition of Federal or federally-related financial assistance for construction or acquisition purposes.

EFFECTIVE DATE: April 21, 1982.

FOR FURTHER INFORMATION CONTACT: Mr. Robert G. Chappell, Chief, Engineering Branch, Natural Hazards Division, Federal Emergency Management Agency, Washington, D.C. 20472, (202) 287-0230.

SUPPLEMENTARY INFORMATION: If a property owner was required to purchase flood insurance as a condition of Federal or federally-related financial assistance for construction or acquisition purposes, and the lender now agrees to waive the property owner from maintaining flood insurance coverage on the basis of this map amendment, the property owner may obtain a full refund of the premium paid for the current policy year, provided that no claim is pending or has been paid on the policy in question during the same policy year. The premium refund may be obtained through the insurance agent or broker who sold the policy, or from the National Flood Insurance Program (NFIP): P.O. Box 34294, Bethesda, Maryland 20034, Phone: (800) 638-6620.

The map amendments listed below are in accordance with § 70.7(b):

Map Number H & I 450174, Panel 0005 B published on December 15, 1980 in 46 FR 82260 indicates that portions of Parcels A and B of the Trenholm Road Shopping Center, Inc., in the City of

Forest Acres, South Carolina, as recorded on Plat Map 14–204 in the Office of the Clerk of Richland County, South Carolina are located within the Special Flood Hazard Area.

Map Number H & I 450174, Panel 0005 B is hereby corrected to reflect that the existing buildings numbered 2B, 2C, 2D, 3 through 6 and 15 are not within the Special Flood Hazard Area identified on November 5, 1980. The buildings numbered 3 through 6 and 15 are located in Zone B. The existing buildings numbered 2B, 2C, and 2D are located in Zone C.

Pursuant to the provisions of 5 U.S.C. 605(b), the Associate Director, State and Local Programs and Support, to whom authority has been delegated by the Director, Federal Emergency Management Agency, hereby certifies that this rule if promulgated will not have a significant economic impact on a substantial number of small entities. This rule provides routine legal notice of technical amendments made to designated special flood hazard areas on the basis of updated information and imposes no new requirements or regulations on participating communities.

List of Subjects in 44 CFR Part 70

Flood insurance, Floodplains.

(National Flood Insurance Act of 1968 (Title XIII of Housing and Urban Development Act of 1968), effective January 28, 1969 (33 FR 17804, November 28, 1968), as amended; 42 U.S.C. 4001–4128; Executive Order 12127, 44 FR 19367; delegation of authority to Associate Director, State and Local Programs and Support)

Issued: March 22, 1982.

Lee M. Thomas,

Associate Director, State and Local Programs and Support.

[FR Doc. 82-10900 Filed 4-20-82; 8:45 am]

BILLING CODE 6718-03-M

---

44 CFR Part 70

[Docket No. FEMA-5909]

Letter of Map Amendment for Harris County, TX, Under National Flood Insurance Program

AGENCY: Federal Emergency Management Agency.

ACTION: Final rule.

SUMMARY: The Federal Emergency Management Agency (FEMA) published a list of communities for which maps identifying Special Flood Hazard Areas have been published. This list included Harris County, Texas. It has been determined by the Associate Director, State and Local Programs and Support, after acquiring additional flood information and after further technical review of the Flood Insurance Rate Map for Harris County, Texas, that certain property is not within the Special Flood Hazard Area.

This map amendment, by establishing that the subject property is not within the Special Flood Hazard Area, removes the requirement to purchase flood insurance for that property as a condition of Federal or federally-related financial assistance for construction or acquisition purposes.

EFFECTIVE DATE: April 21, 1982.

FOR FURTHER INFORMATION CONTACT: Mr. Robert G. Chappell, P.E., Chief, Engineering Branch, Natural Hazards Division, Federal Emergency Management Agency, Washington, D.C. 20472, (202) 287-0230.

SUPPLEMENTARY INFORMATION: If a property owner was required to purchase flood insurance as a condition of Federal or federally-related financial assistance for construction or acquisition purposes, and the lender now agrees to waive the property owner from maintaining flood insurance coverage on the basis of this map amendment, the property owner may obtain a full refund of the premium paid for the current policy year, provided that no claim is pending or has been paid on the policy in question during the same policy year. The premium refund may be obtained through the insurance agent or broker who sold the policy, or from the National Flood Insurance program (NFIP) at: P.O. Box 34294, Bethesda, Maryland 20034, Telephone: (800) 638-6620.

The map amendments listed below are in accordance with § 70.7(b):

Map No. H & I 480287 Panel 0175C, published on October 6, 1980, in 45 FR 66098, indicates that Lots 92 through 95, Block 5; Lots 1 through 3, Block 9; Lots 17 through 20 and 33 through 36, Block 10; and Lots 12 through 15, 20 through 23, and 27 through 30, Block 11, Oak Cliff Place, Section Two, Harris County, Texas, recorded as Instrument No. H040252 in Volume 303, page 93 of Map Records, in the Office of the Clerk, Harris County, Texas, are within the Special Flood Hazard Area.

Map No. H&I 480287 Panel 0175C is hereby corrected to reflect that the above mentioned lots are not within the Special Flood Hazard Area identified on February 24, 1981. These lots are in Zone C.

Pursuant to the provisions of 5 U.S.C. 605(b), the Associate Director, State and Local Programs and Support to whom authority has been delegated by the Director, Federal Emergency Management Agency, hereby certifies that this rule if promulgated will not have a significant economic impact on a substantial number of small entities. This rule provides routine legal notice of technical amendments made to designated special flood hazard areas on the basis of updated information and imposes no new requirements or regulations on participating communities.

List of Subjects in 44 CFR Part 70

Flood insurance, Floodplains.

(National Flood Insurance Act of 1968 (Title XIII of Housing and Urban Development Act of 1968), effective January 28, 1969 (33 FR 17804, November 28, 1968), as amended; 42 U.S.C. 4001–4128; Executive Order 12127, 44 FR 19367; delegation of authority to Associate Director, State and Local Programs and Support)

Issued: March 23, 1982.

Lee M. Thomas,

Associate Director, State and Local Programs and Support.

[FR Doc. 82-10902 Filed 4-20-82; 8:45 am]

BILLING CODE 6718-03-M

---

44 CFR Part 70

[Docket No. FEMA-6116]

Letter of Map Amendment for Martin County, Florida, Under National Flood Insurance Program

AGENCY: Federal Emergency Management Agency.

ACTION: Final rule.

SUMMARY: The Federal Emergency Management Agency published a list of communities for which maps identifying Special Flood Hazard Areas have been published. This list included Martin County, Florida. It has been determined by the Associate Director, State and Local Programs and Support after acquiring additional flood information and after further technical review of the Flood Insurance Rate Map for Martin County, Florida, that certain property is not within the Special Flood Hazard Area.

This map amendment, by establishing that the subject property is not within the Special Flood Hazard Area, removes the requirement to purchase flood insurance for that property as a condition of Federal or federally-related financial assistance for construction or acquisition purposes.

EFFECTIVE DATE: April 21, 1982.

FOR FURTHER INFORMATION CONTACT: Mr. Robert G. Chappell, Chief, Engineering Branch, Natural Hazards Division, Federal Emergency

Management Agency, Washington, D.C. 20472, (202) 287-0230.

SUPPLEMENTARY INFORMATION: If a property owner was required to purchase flood insurance as a condition of Federal or federally related financial assistance for construction or acquisition purposes, and the lender now agrees to waive the property owner from maintaining flood insurance coverage on the basis of this map amendment, the property owner may obtain a full refund of the premium paid for the current policy year, provided that no claim is pending or has been paid on the policy in question during the same policy year. The premium refund may be obtained through the insurance agent or broker who sold the policy, or from the National Flood Insurance Program (NFIP): P.O. Box 34294, Bethesda, Maryland 20034, Phone: (800) 638-6620.

The map amendments listed below are in accordance with § 70.7(b):

Map Number H & I 120161, Panel 0170 B published on July 22, 1981 in 46 FR 37654 indicates that portions of Plat Numbers 1, 1A, 2, and 3 of Sailfish Point Subdivision, as recorded in Plat Book 8, Pages, 10, 47, 48, and 49, respectively, Public Records of Martin County, Florida, are located within the Special Flood Hazard Area.

Map Number H & I 120161, Panel 0170 B is hereby corrected to reflect that the existing structures on Lots 3, 5, 7 through 9, 14 through 16, 18, 21, and the guard house, all on Plat No. 1, the water treatment buildings on Parcel C-1, the golf course maintenance building on Parcel C-2, both on Plat No. 1A, the ship's chandlery building on Parcel E of Plat No. 2, and the golf starter building on Parcel G of Plat No. 3, are not within the Special Flood Hazard Area identified on June 15, 1981. These structures are located in Zone B. Portions of the property would still be inundated by a flood having a one-percent chance of occurrence in any given year (base flood).

Pursuant to the provisions of 5 U.S.C. 605(b), the Associate Director, State and Local Programs and Support, to whom authority has been delegated by the Director, Federal Emergency Management Agency, hereby certifies that this rule if promulgated will not have a significant economic impact on a substantial number of small entities. This rule provides routine legal notice of technical amendments made to designated special flood hazard areas on the basis of updated information and imposes no new requirements or regulations on participating communities.

List of Subjects in 44 CFR Part 70

Flood insurance, Floodplains.

(National Flood Insurance Act of 1968 (Title XIII of Housing and Urban Development Act of 1968), effective January 28, 1969 (33 FR 17804, November 28, 1968), as amended (42 U.S.C. 4001-4128); Executive Order 12127, 44 FR 19367; delegation of authority to Associate Director, State and Local Programs and Support)

Issued: March 22, 1982.

Lee M. Thomas,

Associate Director, State and Local Programs and Support.

[FR Doc. 82-10893 Filed 4-20-82; 8:45 am]

BILLING CODE 6718-03-M

FEDERAL COMMUNICATIONS COMMISSION

47 CFR Part 73

[FCC 82-171]

Radio Broadcast Services; Amendment of the Commission's Rules To Eliminate the Provision That Applicants Submit Proof of Publication of Local Notice

AGENCY: Federal Communications Commission.

ACTION: Final rule.

SUMMARY: This action revises § 73.3580(h) of the Commission's rules to delete the requirement that applicants for broadcast contruction permits submit actual proof of compliance with the local notice requirements contained in § 73.3580 of the rules. This submission requirement has already been eliminated by other Commission actions and the present action is necessary to make § 73.3580(h) consistent with those actions.

EFFECTIVE DATE: April 23, 1982.

ADDRESS: Federal Communications Commission, 1919 M Street, NW., Washington, D.C.

FOR FURTHER INFORMATION CONTACT: Robert Hayne, Broadcast Bureau, (202) 632-6485.

SUPPLEMENTARY INFORMATION:

Adopted: April 1, 1982.

Released: April 9, 1982.

In re matter of amendment of § 73.3580 of the rules.

1. The Commission, on its own motion, is hereby amending § 73.3580(h) of the Rules to eliminate the provision that applicants submit proof of publication of local notice.[1] Instead, we will require certification of compliance. This action reflects our previous actions with respect to the broadcast construction permit application (Form 301) and the "short form" renewal application (Form 303-S). As a result of this action, certification will be sufficient for the other applications, and amendments thereto, in which a local notice is required by § 73.3580 of the Rules.

2. On June 16, 1981, we adopted a revised Form 301 (FCC 81-278). The revised Form 301 merely requires the construction permit applicant to certify compliance with § 73.3580 of the Rules. Formerly, the publication occurred following the tendering of the application. This necessitated an amendment to the pending application within 7 days of the completion of the local notice requirement. See § 73.3580(h) of the Rules. Similarly, on May 11, 1981, we adopted a "short form" renewal application (Form 303-S) which also deleted the submission requirement by merely requiring that certification of compliance along with the dates, times and text of the local notice be associated with the renewal application in the public file. (FCC 81-146). Section 73.3580(h) is not now applicable to the construction permit application filed on Form 301 and the renewal application filed on Form 303-S.

3. We continue to believe that local notice is a valuable administrative tool in informing the public of the pendency of an application. However, requiring any applicant to amend an application to submit actual proof of compliance is an unnecessary inconvenience to the applicant and delays our processing. As indicated earlier, we have already determined that applicants filing Forms 301 and 303-S, and major amendments thereto, need not submit a separate statement of compliance with § 73.3580 of the Rules. The rationale for these changes applies with equal force to the other broadcast applications within the public notice requirement of § 73.3580. Therefore, these applications will be revised to specifically provide for certification of compliance. In the meantime, we are amending § 73.3580(h) of the Rules to substitute a certification requirement in lieu of the requirement

---

[1] Section 73.3580(h) provides in pertinent part, as follows:

"(h) Within 7 days of the last day of publication in a newspaper or broadcast of the notice required by paragraphs (c), (d) or (g) of this Section, the applicant shall file a statement with the FCC in triplicate if filed pursuant to paragraph (c) or (d); original only, if filed pursuant to paragraph (g), setting forth the dates on which the notice was published, the newspaper in which the notice was published, the text of notice, and/or, where applicable, the dates and times that the notice was broadcast and the text thereof * * *"

that actual proof of local notice be submitted within 7 days.

4. The Administrative Procedure Act exempts rules of procedure and practice from its notice and comment rulemaking requirements, 5 U.S.C. 553(b)(A). For that reason, because this proceeding draws upon actual experience in application processing, and because of the urgency of expedited application processing, we will not solicit comments on the matters discussed herein. Furthermore, we will make the changes effective immediately upon publication in the Federal Register. The Appendix contains the rule change adopted herein.

5. Authority for adoption of the amendments set out in the attached Appendix is contained in sections 4(i) and 303(r) of the Communications Act of 1934, as amended, 47 U.S.C. 154(i) and 303(r). For the reasons recited in paragraph 4 above, the prior notice and effective date provisions of 5 U.S.C. 553 are inapplicable.

6. Accordingly, it is ordered, that Part 73 of the Rules is amended as set out in the Appendix hereto, April 23, 1982.

List of Subjects in 47 CFR Part 73

Radio broadcasting.

(Secs. 4, 303, 307, 48 Stat., as amended, 1066, 1082, 1083; 47 U.S.C. 154, 303, 307)

Federal Communications Commission.

William J. Tricarico.

Secretary.

Appendix

PART 73—RADIO BROADCAST SERVICES

Section 73.3580(h) of the Commission's rules is revised to read as follows:

§ 73.3580 Local public notice of filing of broadcast applications.

* * * * *

(h) The applicant may certify in the appropriate application that it has or will comply with the public notice requirements contained in paragraphs (c), (d) or (g) of this Section. However, an applicant for renewal of license shall, within 7 days of the last day of broadcast of the required publication announcements, place in its public inspection file a statement certifying compliance with Section 73.3580 along with the dates and times that the pre-filing and post-filing notices were broadcast and the text thereof. This certification need not be filed with the Commission but shall be retained in the public inspection file for as long as the application to which it refers.

[FR Doc. 82-10858 Filed 4-20-82; 8:45 am]

BILLING CODE 6712-01-M

DEPARTMENT OF THE INTERIOR

Fish and Wildlife Service

50 CFR Part 26

Special Regulations, Public Access and Use; Wichita Mountains Wildlife Refuge, Oklahoma

AGENCY: Fish and Wildlife Service, Interior.

ACTION: Extension of expiration of special regulations.

SUMMARY: It has been determined that the opening to public access, use, and recreation of the Wichita Mountains Wildlife Refuge, Oklahoma, is compatible with the objectives for which the area was established and will provide additional recreational opportunity to the public. This document extends the duration of the regulations published on January 27, 1981, to December 31, 1982, and provides the Office of Management Budget clearance number for information collection. The duration of these rules is extended to December 31, 1982, so that orderly management of the refuge will not be compromised.

EFFECTIVE DATES: April 21, 1982 through December 31, 1982.

FOR FURTHER INFORMATION CONTACT: Robert A. Karges, Refuge Manager, Wichita Mountains Wildlife Refuge, Route 1, Box 448, Indiahoma, Oklahoma 73552 (Telephone 405-429-3221).

SUPPLEMENTARY INFORMATION: The author of this document is Ronald L. Fowler, Division of Refuge Management, U.S. Fish and Wildlife Service, 18th & C Streets, NW, Washington, D.C. 20240 (Telephone 202-343-4305).

General

On January 27, 1981, public access and use regulations for the Wichita Mountains Wildlife Refuge were published in the Federal Register (46 FR 8525) in accordance with provisions of 50 CFR 26.24. These regulations were effective through December 31, 1981. The duration of these rules is extended to December 31, 1982, so that the orderly management of the refuge will not be compromised. In addition, the Office of Management and Budget clearance number for information collections for permits for stays exceeding 7 days and back-country camping permits is given.

The Refuge Recreation Act of 1962 (16 U.S.C. 460k) authorizes the Secretary of the Interior to administer refuge areas for public recreation as an appropriate incidental or secondary use only to the extent that it is practicable and not inconsistent with the primary objectives for which the area was established. In addition, the Refuge Recreation Act requires (1) that any recreational use permitted will not interfere with the primary purpose for which the area was established; and (2) that funds are available for the development, operation, and maintenance of the permitted forms of recreation. The recreational use authorized by these regulations will not interfere with the primary purposes for which these National Wildlife Refuges were established. This determination is based upon consideration of, among other things, the Service's Final Environmental Statement on the Operation of the National Wildlife Refuge System published in November 1976. Funds are available for the administration of the recreational activities permitted by these regulations.

The Department of the Interior has determined that this rulemaking is not a "major rule" within the meaning of Executive Order 12291 (46 FR 13193), and that the rulemaking would not have "significant economic effect on a substantial number of small entities" within the meaning of the Regulatory Flexibility Act (Pub. L. 96-354).

List of Subjects in 50 CFR Part 26

National wildlife refuge system, Recreation, Wildlife refuges.

PART 26—PUBLIC ENTRY AND USE

Accordingly, the special regulations for public access and use published at 50 CFR 26.34 for the Wichita Mountains Wildlife Refuge remain in effect until December 31, 1982, and the regulations are amended by the addition of the following paragraph (17):

§ 26.34 Special regulations concerning public access, use, and recreation for individual National Wildlife Refuges.

Oklahoma

Wichita Mountains Wildlife Refuge

* * * * *

(17) *Information collection.* The information collection requirements contained in paragraphs (7) and (8) of these regulations have been approved by the Office of Management and Budget under 44 U.S.C. 3507 and assigned clearance number 1018-0043. The information is being collected to solicit information necessary for the

Refuge Manager to issue permits and other benefits. The information will be used to grant administrative benefits. In both sections the obligations to respond is required to obtain a benefit.

(16 U.S.C. 460k, 668dd. Paperwork Reduction Act of 1980, 94 Stat. 2812. Pub. L. 96-511)

Dated: March 30, 1982.

J. Craig Potter,

Acting Assistant Secretary for Fish and Wildlife and Parks.

[FR Doc. 82-10860 Filed 4-20-82; 8:45 am]

BILLING CODE 4310-55-M

Proposed Rules

Federal Register
Vol. 47, No. 77
Wednesday, April 21, 1982

This section of the FEDERAL REGISTER contains notices to the public of the proposed issuance of rules and regulations. The purpose of these notices is to give interested persons an opportunity to participate in the rule making prior to the adoption of the final rules.

DEPARTMENT OF AGRICULTURE

Animal and Plant Health Inspection Service

9 CFR Part 151

[Docket No. 82-025]

Recognized Breeds and Books of Record

AGENCY: Animal and Plant Health Inspection Service, USDA.

ACTION: Proposed rule.

SUMMARY: This document proposes to amend the regulations by adding the Russian Arabian Stud Book of Moscow to the list of "Recognized breeds and books of records." A breed of animal and its book of record must be approved by the Animal and Plant Health Inspection Service (Service) and added to the list contained in the regulations to entitle the owner or agent free entry of the animal into the United States. The effect of this action would be the addition of a book of record to the current list, thereby providing for duty-free entry of certain Arabian horses which are registered in the "Russian Arabian Stud Book of Moscow."

DATE: Comments must be received on or before June 21, 1982.

ADDRESS: Comments to Deputy Administrator, USDA, APHIS, VS, Federal Building, Room 870, 6505 Belcrest Road, Hyattsville, MD 20782.

FOR FURTHER INFORMATION CONTACT: Dr. D. E. Herrick, USDA, APHIS, VS, Federal Building, Room 821, Hyattsville, MD 20782, 301-436-8530.

SUPPLEMENTARY INFORMATION:

Executive Order 12291

This proposed rule has been reviewed in conformance with Executive Order 12291 and has been determined to be not a "major rule." Based on information compiled by the Department, it has been determined that this action would have an annual effect on the economy of less than one hundred million dollars; would not cause a major increase in costs or prices for consumers, individual industries, Federal, State of local government agencies, or geographic regions; would not have a significant adverse effect on comptetiton, employments, investment, productivity, innovation, or the ability of United States-based enterprises to compete with foreign-based enterprises in domestic or export markets.

Certification Under the Regulatory Flexibility Act

Dr. Harry C. Mussman, Administrator of the Animal and Plant Health Inspection Service, has determined that this action will not have a significant economic impact on a substantial number of small entities. This is because this action would only affect those importers interested in importing Arabian horses registered in the Russian Arabian Stud Book of Moscow. It is anticipated that annually no more than six Arabian horses registered in the Russian Arabian Stud Book of Moscow would seek the duty-free entry into the United States which this amendment would permit.

Notice is hereby given in accordance with the administrative procedure provisions in 5 U.S.C 553, that pursuant to the provisions of Sec. 101, 76 Stat. 72, Item 100.01, Title I, Tariff Act of 1930, as amended (19 U.S.C 1202, Item 100.01); 37 FR 28464, 28477; 38 FR 19141, the Animal and Plant Health Inspection Service is considering amending Part 151, Title 9, Code of Federal Regulations.

Alternatives

The following alternatives were considered:

1. Not amend the regulations.
2. Amend the regulations to add the USSR Book of Record for the Arabian Horse to the present list.

Alternative No. 1 was not selected because this would place an unnecessary restriction on Arabian horses registered in the Russian Arabian Stud Book of Moscow.

Alternative No. 2 was selected because the Russian Arabian Stud Book of Moscow has been examined by a representative of the Service and is acceptable.

Background

A book of record for a breed of animal must be examined and approved by the Service before it is eligible to be added to the list contained in § 151.9 of the regulations (9 CFR 151.9). If the breed of animal or book of record of the animals required to pay duties are not included in this part, the owner or agent must pay such duties when the animal is presented for entry into the United States.

To become eligible as a recognized breed, the animal must be the progeny of three complete generations of known and recorded purebred animals of the particular breed involved.

The book of record must be a printed book or an approved microfilm record sponsored by a registry association and containing breeding data relative to a large number of registered purebred animals used as a basis for the issuance of pedigree certificates.

A representative of the Service has examined the Russian Arabian Stud Book of Moscow and has determined that the book of record meets all the requirements necessary to be added to the list in § 151.9. Therefore, the Department is proposing to amend Title 9, Code of Federal Regulations, § 151.9, by adding the Russian Arabian Stud Book of Moscow to the list of "Recognized breeds and books of record."

List of Subjects in 9 CFR Part 151

Animal pedigree, Animals, Imports, Purebred animals.

PART 151—RECOGNITION OF BREEDS AND BOOKS OF RECORD OF PUREBRED ANIMALS

Accordingly, in § 151.9, the chart in paragraph (a) would be amended by adding the following between Code 2208, The Arabian Stud Book of the Arabian breed, and Code 2101, Stud-Book des Chevaux de Trait Belges of the Belgian breed under the heading "Horses."

§ 151.9 Recognized breeds and books of record.

Code	Name of breed	Book of record	By whom published
* * *	* *	* *	* *
2304	Arabian	Russian Arabian Stud Book of Moscow, Union of Soviet Socialist Republics.	Ministry of Agriculture USSR, All-Union Research Institute of Horsebreeding.
* * *	* *	* *	* *

All written submissions made pursuant to this notice will be made available for public inspection at the Federal Building, 6505 Belcrest Road, Room 870, Hyattsville, Maryland, during regular hours of business (8 a.m. to 4:30 p.m., Monday to Friday, except holidays) in a manner convenient to the public business (7 CFR 1.27(b)).

Comments submitted should bear a reference to the date and page number of this issue in the Federal Register.

Done at Washington, D.C., this 14th day of April 1982.

J. K. Atwell,

Deputy Administrator, Veterinary Services.

[FR Doc. 82-10799 Filed 4-20-82; 8:45 am]

BILLING CODE 3410-34-M

DEPARTMENT OF THE TREASURY

Comptroller of the Currency

12 CFR Part 31

[Docket No. 82-4]

Accrual Accounting; Proposed Requirement

AGENCY: Comptroller of the Currency, Treasury.

ACTION: Notice of proposed rulemaking.

SUMMARY: The Office of the Comptroller of the Currency (Office) is proposing to require the adoption of accrual accounting by all national banks as recommended by the Federal Financial Institutions Examination Council (Council).

The regulation would become effective on January 1, 1983, for national banks with assets of more than $10 million, and on January 1, 1985, for all other national banks. The purpose of this regulation is to assure that the financial data they provide to federal regulators and make available to bank shareholders, customers, and the general public are timely, consistent, and fairly present their financial condition, income and expenses.

Notice is being published at this time in order that banks not now using accrual accounting may have ample time to convert to that procedure.

DATES: Written comments should be received no later than June 21, 1982. It is proposed that the regulation will become effective on January 1, 1983, for national banks with assets of more than $10 million and on January 1, 1985, for all other National banks.

ADDRESS: Comments should be directed to: Docket No. 82-4 Communications Division, Third Floor, Office of the Comptroller of the Currency, 490 L'Enfant Plaza, East, S.W., Washington, D.C. 20219, Attention: Marie T. Giblin, (202) 447-1800. Comments will be available for inspection and photocopying.

FOR FURTHER INFORMATION CONTACT: Rhoger H. Pugh, National Bank Examiner, Program Analysis Division, Office of the Comptroller of the Currency, Washington, D.C. 20219, (202) 447-1723.

SUPPLEMENTARY INFORMATION:

Special Studies

No Regulatory Flexibility Analysis or Regulatory Impact Analysis has been prepared for this proposal. A Regulatory Flexibility Analysis was waived by the Secretary of the Treasury who determined that none was required in this instance. An informal survey of accounting practitioners undertaken by the Council's Reports Task Force and a formal survey of banks with assets of less than $25 million conducted by the Federal Deposit Insurance Corporation under the auspices of the Council in August of 1981 yielded estimates of the costs to convert to the accrual basis and maintain such an accounting system in subsequent years. The cost of conversion is expected to be approximately $2,000 per institution and annual operating costs about $1,000.

Publication of the proposed accrual accounting guideline by the Council on August 14, 1981 (46 FR 41204) generated additional information. During the comment period, 271 letters were received. Many bankers predicted their costs would be higher than those listed above. Agency estimates are, however, predicated upon the actual costs reported by banks which had converted to the accrual system within three years prior to the August 1981 survey. Presuming that the average asset size of an affected bank is $12 million, and the cost of conversion for such a bank proved to be twice what the survey showed, its first-year expense still would be only $6,000 or .05 percent of its total resources. Such an impact does not reach the level of significance contemplated by the Regulatory Flexibility Act.

In an effort to minimize the burden on smaller institutions, the banking agencies have allowed an extended lead time, until January 1, 1985, for banks with assets of $10 million or less to convert to the accrual basis.

A Regulatory Impact Analysis is not required because the OCC has determined that the proposal is not a "major rule" as defined by Executive Order 12291. It will not have an annual effect on the economy of $100 million or more, will not result in a major increase in costs or prices to consumers, individual industries, Federal, State or local government agencies, or geographic regions, nor will it have significant adverse effects on competition, employment, investment, productivity, innovation, or on the ability of United States-based enterprises to compete with foreign-based enterprises.

Drafting Information

The principal drafter of this document is Rhoger H. Pugh, National Bank Examiner, Program Analysis Division, Office of the Comptroller of the Currency, (202) 447-1723.

1. *Scope of proposed regulation.* On November 5, 1981, the Council directed, pursuant to section 1006(c) of Pub. L. 95-630, 12 U.S.C. 3305(c), the banking agencies to require all insured commercial and mutual savings banks to file their Reports of Condition and Income on the accrual basis. Subject to OMB approval, this requirement is to become effective January 1, 1983, for banks with resources of more than $10 million and January 1, 1985, for banks with resources of $10 million or less. The only banks directly affected by this reporting standard are those with assets of less than $25 million. Banks with resources of more than $25 million have been required since 1970 by the instructions for preparation of reports of condition and income to prepare those reports on the accrual basis of accounting.

At the same time, pursuant to section 1006(b) of Pub. L. 95-630, 12 U.S.C. 3305(b), the Council recommended that federal supervisors of insured commercial and mutual savings banks require those institutions to convert their books to the accrual basis of accounting, on the same time schedule suggested for accrual reporting. In accordance with that recommendation, two constituent agencies of the Council, the Federal Deposit Insurance Corporation and the Office of the Comptroller of the Currency, are proposing identical regulations.

OCC does not know of any national banks with assets of more than $25 million which do not maintain their books on an accrual basis. However, there might be a few. Such banks would

be affected by this regulation in the same manner as many under $25 million in assets.

Banks with assets of less than $25 million previously had the option of filing their Reports of Condition and Income on a modified cash or an accrual basis. Those using the former system will be affected by this regulation.

The August 1981 Survey, mentioned previously, indicated that of the 4,676 insured commercial banks with assets of between $10 and $25 million, 2,617 had already adopted accrual accounting, and of the 2,830 insured commercial banks with assets of under $10 million, 1,102 had already adopted accrual accounting. Thus, only about half of the banks with assets under $25 million would have to convert to accrual accounting under the proposed regulation.

For banks that must change accounting methods, the shift will not impose entirely new procedures. All banks have been subject to some accrual reporting standards. Since 1970, banks, regardless of their size, have been required to report four items on the accrual basis. These items are: income from installment loans; depreciation of fixed assets; bond premium amortization; and income taxes. Furthermore, the proposed regulation and related reporting standards affect only material accounts. It is one of the tenets of accrual accounting that amounts which are immaterial need not be accrued. Finally, the agencies are not proposing that accruals be posted daily, but rather that the bank management adopt an accrual frequency which is appropriate to the nature of each specific account or transaction.

Pursuant to Internal Revenue Service Ruling 68–83, the proposed requirement does not preclude a national bank from filing its federal income tax return on the cash receipts and disbursements method, as long as the bank's permanent books and records reconcile the two methods of accounting.

2. *Purpose of proposed regulation.* In times of deregulation, volatile interest rates, and rapidly changing banking practice and portfolio patterns, it is extremely difficult to estimate the true financial position of banks which account for material components of income and expense on a cash basis. Cash basis accounting is particularly likely to misrepresent the results of operation in times of change. In a cash basis accounting system, income and expenses can more easily be shifted from one period to another, and income and expenses may not be reported in the period in which they were earned or incurred. Thus, it is also difficult to make comprehensive judgments about the performance of an individual bank or group of banks when some banks report on the accrual basis and others do not.

Such judgments will become increasingly important as the banking agencies attempt to modernize their supervisory techniques to deal with changes in the banking system, budgetary constraints, and the need to lessen the direct burden of on-site examinations. One technique calls for increasing reliance on the use of monitoring reports filed by depository institutions to make preliminary determinations about their condition. Such monitoring can be used to detect problem institutions at an early stage and to more carefully target the scope and frequency of on-site examinations. It also permits reductions in both the frequency and length of examinations of institutions without apparent problems. Uniform use of accrual accounting is necessary for the efficient functioning of such off-site monitoring.

The Office is in favor of greater deregulation of the banking industry and believes that regulations which unduly restrict bank management's ability to serve the banking needs of the public should be eliminated. For a bank to survive in a less regulated environment, its management must have sufficient information to make appropriate choices on matters upon which, prior to deregulation, it had no choices. A vital component of that necessary information is an accurate picture of the financial condition of the bank and the results of its operation. Thus, although the imposition of accrual accounting may increase the operating expenses of a bank not already using an accrual accounting system, such an imposition is a necessary precondition for the Government, bank regulatory agencies, and the banking industry to implement a permissive but prudent course toward significant deregulation.

List of Subjects in 12 CFR Part 31

Accounting, National banks.

For the reasons set out in the preamble, the Office proposes to add a new Part 31 to Chapter 1 of Title 12 of the Code of Federal Regulations as set forth below.

PART 31—ACCRUAL ACCOUNTING

Sec.
31.1 Purpose, Scope and Effective dates.
31.2 Accrual Accounting Requirement.

Authority: R.S. 324 et seq., as amended (12 U.S.C. 1 et seq.).

§ 31.1 Purpose, scope, and effective dates.

(a) *Purpose.* This part is issued to assure that financial data provided or available to federal regulators, bank shareholders, customers, and the general public are timely, consistent, and fairly present the results of the operations and financial condition of the bank.

(b) *Scope.* This part applies to all national banks.

(c) *Effective dates.* This part is effective upon the following dates: January 1, 1983 for all national banks with assets of more than $10 million as of December 31, 1981; and January 1, 1985, for all national banks.

§ 31.2 Accrual Accounting Requirement.

As of the effective dates specified in § 31.1(c), banks shall maintain their books and records on the accrual basis of accounting. Banks shall make all material accrual adjustments to assure that periodic revenues and expenses are matched for recording purposes to the specific periods of time, such as the day or month, in which they are earned or incurred, without regard to the actual date of receipt or payment of cash. The posting frequency for accruals shall be appropriate to (1) the specific transaction involved, (2) the volatility of the underlying asset or liability, and (3) the schedule of reporting for internal bank management; and, normally, frequency of posting will be at least monthly. This requirement in no way prohibits banks from maintaining such cash basis records or other records as are necessary for tax or other purposes in accordance with Federal or other statutes or regulations.

Dated: March 25, 1982.

C. T. Conover,

Comptroller of the Currency.

[FR Doc. 82-10835 Filed 4-20-82; 8:45 am]

BILLING CODE 4810-33-M

DEPARTMENT OF ENERGY

Federal Energy Regulatory Commission

18 CFR Part 271

[Docket No. RM79-76 (Wyoming-13)]

Wyoming; High-Cost Gas Produced From Tight Formations

April 15 1982.

AGENCY: Federal Energy Regulatory Commission, DOE.

ACTION: Notice of Proposed Rulemaking.

SUMMARY: The Federal Energy Regulatory Commission is authorized by section 107(c)(5) of the Natural Gas Policy Act of 1978 to designate certain types of natural gas as high-cost gas where the Commission determines that the gas is produced under conditions which present extraordinary risks or costs. Under section 107(c)(5), the Commission issued a final regulation designating natural gas produced from tight formations as high-cost gas which may receive an incentive price (18 CFR 271.703). This rule established procedures for jurisdictional agencies to submit to the Commission recommendations of areas for designation as tight formations. This Notice of Proposed Rulemaking by the Director of the Office of Pipeline and Producer Regulation contains the recommendation of the State of Wyoming that the Lance, Meeteetse, and Mesaverde Formations each be designated as a tight formation under § 271.703(d).

DATE: Comments on the proposed rule are due on May 17, 1982.

Public Hearing. No public hearing is scheduled in this docket as yet. Written requests for a public hearing are due on April 30, 1982.

ADDRESS: Comments and requests for hearing must be filed with the Office of the Secretary, 825 North Capitol Street, NE., Washington, D.C. 20426.

FOR FURTHER INFORMATION CONTACT: Leslie Lawner, (202) 357-8511, or Victor Zabel, (202) 357-8616.

SUPPLEMENTARY INFORMATION:

I. Background

On April 2, 1982, the State of Wyoming Oil and Gas Conservation Commission (Wyoming) submitted to the Commission a recommendation, in accordance with § 271.703 of the Commission's regulations (45 FR 56034, August 22, 1980), that the Lance, Meeteetse, and Mesaverde Formations located in Fremont and Natrona Counties, Wyoming, each be designated as a tight formation. Pursuant to § 271.703(c)(4) of the regulations, this Notice of Proposed Rulemaking is hereby issued to determine whether Wyoming's recommendation that the Lance, Meeteetse, and Mesaverde Formations each be designated a tight formation should be adopted. The United States Department of the Interior, Minerals Management Service (formerly the U.S. Geological Survey) concurs with Wyoming's recommendation. Wyoming's recommendation and supporting data are on file with the Commission and are avaliable for public inspection.

II. Description of Recommendation

The recommended formations underlie a portion of Fremont and Natrona Counties in the Wind River Basin of central Wyoming. The area encompasses portions of Townships 38 and 39 North, Ranges 88 through 92 West. The Lance Formation lies between the base of the Lower Fort Union Formation and the top of the Meeteetse Formation. The average depth to the top of the Lance Formation if 8,915 feet. The Meeteetse Formation lies between the base of the Lance Formation and the top of the Mesaverde Formation. The average depth to the top of the Meeteetse Formation is 14, 319 feet. The Mesaverde Formation lies between the base of the Meeteetse Formation and the top of the Cody Shale Formation. The average depth to the top of the Mesaverde Formation is 15,396 feet.

III. Discussion of Recommendation

Wyoming claims in its submission that evidence gathered through information and testimony presented at a public hearing in Cause No. 2, Order No. 1, and Cause No. 3, Order No. 1, Docket No. 238-81 convened by Wyoming on this matter demonstrates that:

(1) The average *in situ* gas permeability throughout the pay sections of the proposed area is not expected to exceed 0.1 millidarcy;

(2) The stabilized production rate, against atmospheric pressure, of wells completed for production from the recommended formations, without stimulation, is not expoected to exceed the maximum, allowable production rate set out in § 271.703(c)(2)(i)(B); and

(3) No well drilled into the recommended formations is expected to produce more than five (5) barrels of oil per day.

Wyoming further asserts that existing State and Federal Regulations assure that development of these formations will not adversely affect any fresh water aquifers.

Accordingly, pursuant to the authority delegated to the Director of the Office of Pipeline and Producer Regulation by Commission Order No. 97, issued in Docket No. RM80-68 (45 *Fed. Reg.* 53456, August 12, 1980), notice is hereby given of the proposal submitted by Wyoming that the Lance, Meeteetse, and Mesaverde Formatins, as described and delineated in Wyoming's recommendation as filed with the Commission, each be designated as a tight formation pursuant to § 271.703.

IV. Public Comment Procedures

Interested persons may comment on this proposed rulemaking by submitting written data, views or arguments to the Office of the Secretary, Federal Energy Regulatory Commission, 825 North Capitol Street, N.E., Washington, D.C. 20426, on or before May 17, 1982. Each person submitting a comment should indicate that the comment is being submitted in Docket No. RM 79-76 (Wyoming-13), and should give reasons including supporting data for any recommendations. Comments should include the name, title, mailing address, and telephone number of one person to whom communications concerning the proposal may be addressed. An original and 14 conformed copies should be filed with the Secretary of the Commission. Written comments will be available for public inspection at the Commission's Division of Public Information, Room 1000, 825 North Capitol Street, NE., Washington, D.C. during business hours.

Any person wishing to present testimony, view, data, or otherwise participate at a pulbic hearing should notify the Commission in writing that they wish to make an oral presentation and therefore request a public hearing. Such request shall specify the amount of time requested at the hearing. Requests should be filed with the Secretary of the Commission no later than April 30, 1982.

List of Subjects in 18 CFR Part 271

Natural gas, Incentive price, Tight formations.

(Natural Gas Policy Act of 1978, 15 U.S.C. 3301-3432.)

PART 271—CEILING PRICES

Accordingly, the Commission proposes to amend the regulations in Part 271, Subchapter H, Chapter I, Title 18, Code of Federal Regulations, as set forth below, in the event Wyoming's recommendation is adopted.

Kenneth A. Williams,

Director, Office of Pipeline and Producer Regulation.

Section 271.703(d) is revised by adding new subparagraphs (109), (110), and (111) to read as follows:

§ 271.703 Tight formations.

* * * * *

(d) *Designated tight formations.*

* * * * *

(78) through (108) [Reserved]

(109) *Lance Formation in Wyoming.* RM79-76 (Wyoming-13).

(i) *Delineation of formation.* The Lance Formation is located in Fremont and Natrona Counties, Wyoming, in Township 38 North, Range 88 West,

Sections 3 through 11, 13 through 36; Township 38 North, Range 89 West; Township 38 North, Range 90 West, Sections 1 through 3, 6 through 36; Township 38 North, Range 91 West; Township 38 North, Range 92 West, Sections 1, 2, 11 through 14, 23, 24, 25; Township 39 North, Range 88 West, Sections 19, 29 through 33; Township 39 North, Range 89 West, Sections 4 through 10, 13 through 36; Township 39 North, Range 90 West, Sections 1 through 32, 36; Township 39 North, Range 91 West; Township 39 North, Range 92 West, Sections 12, 13, 14, 23 through 26, 35, 36.

(ii) *Depth.* The Lance Formation lies between the base of the Lower Fort Union Formation and the top of the Meeteetse Formation. The average depth to the top of the Lance Formation is 6,915 feet.

(110) *Meeteetse Formation in Wyoming.* RM79-76 (Wyoming-13).

(i) *Delineation of formation.* The Meeteetse Formation is located in Fremont and Natrona Counties, Wyoming, in Township 38 North, Range 88 West, Sections 3 through 11, 13 through 36; Township 38 North, Ranges 89, 90, and 91 West; Township 38 North, Range 92 West, Sections 1, 2, 11 through 14, 23, 24, 25; Township 39 North, Range 88 West, Sections 19, 29 through 33; Township 39 North, Range 89 West, Sections 4 through 10, 13 through 36; Township 39 North, Ranges 90 and 91 West; Township 39 North, Range 92 West, Sections 12, 13, 14, 23 through 26, 35, 36.

(111) *Mesaverde Formation in Wyoming.* RM79-76 (Wyoming-13).

(i) *Delineation of formation.* The Mesaverde Formation is located in Fremont and Natrona Counties, Wyoming, in Township 38 North, Range 89 West, Sections 6, 7; Township 38 North, Range 90 West, Sections 1 through 12, 15 through 18; Township 38 North, Range 91 West, Sections 2, 3, 4, 9 through 12; Township 39 North, Range 90 West, Sections 28 through 36; Township 39 North, Range 91 West, Sections 25, 26, 34, 35, 36.

(iii) *Depth.* The Mesaverde Formation lies between the base of the Meeteetse Formation and the top of the Cody Shale Formation. The average depth to the top of the Meeteetse Formation is 15,396 feet.

[FR Doc. 82-10940 Filed 4-20-82; 8:45 am]
BILLING CODE 6717-01-M

DEPARTMENT OF THE TREASURY

Customs Service

19 CFR Part 123

Customs Form 7533; Invitation to the Public To Comment

AGENCY: Customs Service, Treasury.

ACTION: Invitation to the public to comment.

SUMMARY: This document gives notice that Customs is inviting comments from interested members of the public concerning Customs proposal to eliminate Customs Form 7533 (Inward Cargo Manifest For Vessel Under Five Tons, Ferry, Train, Car, Vehicle, Etc.) and develop a new standardized form to be used servicewide. Presently there are about 200 versions of Customs Form 7533 in use by various carriers. This has presented problems for exporters who wish to computerize their operations. The development of a standardized form will eliminate these problems and facilitate entry of merchandise arriving from contiguous countries.

DATES: Comments (preferably in triplicate) must be received on or before June 21, 1982.

ADDRESS: Comments should be addressed to the Commissioner of Customs, Attention: Regulations Control Branch, 1301 Constitution Avenue, NW., Room 2426, Washington, D.C. 20229.

FOR FURTHER INFORMATION CONTACT: Patricia Anson, Cargo Processing Division, Office of Inspection, U.S. Customs Service, 1301 Constitution Avenue, NW., Washington, D.C. 20229 (202-566-5354).

SUPPLEMENTARY INFORMATION:

Background

Section 123.3, Customs Regulations (19 CFR 123.3), requires that baggage or other merchandise carried on a vehicle or on a vessel of less than 5 net tons arriving otherwise than by sea from Canada or Mexico be listed on a manifest as prescribed by § 123.4, Customs Regulations (19 CFR 123.4). With certain exceptions, § 123.4 provides that the inward foreign manifest required by § 123.3 for a vehicle or vessel of less than 5 net tons arriving in the United States from Canada or Mexico otherwise than by sea with baggage or merchandise, shall be on Custons Form 7533.

For many years Customs has permitted individual carriers to develop their own format of Customs Form 7533 recognizing that each mode of transportation has specific documentation requirements. As a result, there are about 200 versions of this form in use so that two shipments from the same exporter to the same importer but shipped by different carriers generally would not be on the same documents. This has presented problems for exporters who wish to computerize their operations, as well as for Customs in processing these entries. Accordingly, Customs is considering eliminating the Customs Form 7533 and developing a new standardized form to be used in its place. To ensure that a new form meets the special needs of each type of carrier, can be easily computerized, and yet cause the least disruption to industry, Customs would welcome public comments.

Comments Invited

No format has been designed for the proposed form. Public comment is invited on its suggested content and format, and the effect of the elimination of the present form. Consideration will be given to any written comments timely submitted to the Commissioner of Customs. Comments submitted will be available for public inspection in accordance with § 103.11(b), Customs Regulations (19 CFR 103.11(b)), on regular business days between the hours of 9:00 a.m. and 4:30 p.m. at the Regulations Control Branch, Headquarters, U.S. Customs Service, 1301 Constitution Avenue, NW., Room 2426, Washington, D.C. 20229.

Drafting Information

The principal author of this notice was Jesse V. Vitello, Regulations Control Branch, U.S. Customs Service. However, personnel from other Customs offices participated in its development.

Dated: April 13, 1982.

William von Raab,
Commissioner of Customs.

[FR Doc. 82-10889 Filed 4-20-82; 8:45 am]
BILLING CODE 4820-02-M

DEPARTMENT OF THE INTERIOR

Bureau of Indian Affairs

25 CFR Part 43d

Preparation of a Membership Roll of the Pribilof Islands Aleut Communities of St. Paul and St. George

April 2, 1982.

AGENCY: Bureau of Indian Affairs, Interior.

ACTION: Proposed rule.

SUMMARY: The Bureau of Indian Affairs proposes to add a new part to its

regulations to establish procedures to govern the preparation of a membership roll of the Pribilof Islands Aleut Communities of St. Paul and St. George. The roll to be prepared will serve as a basis for a per capita distribution of a portion of judgment funds awarded the Pribilof Islands by the U.S. Court of Claims.

DATE: Comments must be received on or before May 21, 1982.

ADDRESS: Written comments should be directed to the Chief, Division of Tribal Government Services, Bureau of Indian Affairs, 1951 Constitution Avenue, N. W., Washington, D. C. 20245.

FOR FURTHER INFORMATION CONTACT: Kathleen L. Slover, Branch of Tribal Enrollment Services, Division of Tribal Government Services, Bureau of Indian Affairs, telephone number (703) 235-8275.

SUPPLEMENTARY INFORMATION: This proposed rule is published in exercise of authority delegated by the Secretary of the Interior to the Assistant Secretary—Indian Affairs by 209 DM 8.

The U. S. Court of Claims awarded a judgment in a compromise settlement to the Aleut Community of St. Paul Island and the Aleut Community of St. George Island in Dockets 352 and 369-A originally filed with the Indian Claims Commission. Funds to satisfy the award were appropriated by Congress.

A plan for the use and distribution of the funds was prepared pursuant to the Judgment Funds Distribution Act of October 19, 1973 (87 Stat. 466), and became effective on June 22, 1980. The plan provides for eighty (80) percent of the award, less attorney fees and litigation expenses, and including all interest and investment income accrued, to be distributed by the Secretary in the form of per capita payments to all persons duly enrolled as members of the Pribilof Islands Aleut Communities of St. Paul and St. George living on the effective date of the plan. In order to determine who is duly enrolled and, thus, eligible to share in the distribution of judgment funds, it will be necessary for the Bureau to prepare a roll as of June 22, 1980, of persons who meet the membership requirements of the Communities. The rule proposed in this part provides procedures to govern the preparation of that roll. Persons who desire to be enrolled and who believe they meet the requirements for enrollment must file or have filed for them completed application forms before the deadline specified in the regulations in order to be eligible to share in the judgment funds. It is intended, however, that the qualifications for enrollment proposed in this part otherwise reflect the membership requirements of the Communities.

Individuals can establish eligibility for membership in the Communities under various substantive requirements which are contained in more than one document. In the interest of clarity, rather than just citing the original documents in the text of the regulations, the various requirements for membership have been stated as one set of definitive enrollment requirements. Certain ambiguities in the membership requirements of the Communities, however, had to be resolved in the process of consolidating and simplifying the enrollment requirements for the purpose of preparing this roll. There is reference in the Communities' membership requirements to the class of "Original Members" as persons who were members of the Communities at the time an amendment to the constitution, amalgamating the two communities into one entity, was ratified. Because leaving the Communities, intending not to return, is grounds for loss of membership, "Original Members" would still have to reside or be presumed to reside on St. Paul or St. George Island in order to be duly enrolled as current members now. Consequently, specific reference to "Original Members" who have continued to reside or are presumed to reside in the Communities is omitted under the qualifications for enrollment in the proposed rule. Also, establishing eligibility on the basis of being a child of a member is qualified as minor children, who are less than eighteen years of age, in the proposed regulations. Children of persons who establish eligibility for membership, who were 18 years of age or older on June 22, 1980, the effective date of the plan, will have to otherwise qualify for enrollment in order to establish their eligibility. In addition, only minor children of those persons who establish eligibility on the basis that they resided or were presumed to have resided on either St. Paul or St. George Island can so qualify. It is not intended that minor children of persons who qualify for membership because they were born on either St. Paul or St. George Island be enrolled merely on the basis of being children of members. It should be noted, however, that minors are not precluded from establishing eligibility on the basis that they resided or are presumed to have resided on either St. Paul or St. George Island if they otherwise meet the requirements for enrollment. In other words, persons less than eighteen years of age on June 22, 1980, may establish eligibility on a basis other than being children of members.

The proposed regulations provide for the Area Director to send notices to certain persons who applied for enrollment pursuant to the Alaska Native Claims Settlement Act of December 18, 1971, Pub. L. 92-203, 85 Stat. 688, as amended and supplemented by the Act of January 2, 1976, Pub. L. 94-204, 89 Stat. 1145. This is to provide actual notice of the preparation of the roll and the procedures to be followed, including the deadline for filing applications, to as many potentially eligible participants as possible.

The information collection requirement contained in § 43d.4(b) has been submitted to the Office of Management and Budget for approval as required by 44 U.S.C. 3507. The collection of this information will not be required until it has been approved by the Office of Management and Budget.

The policy of the Department of the Interior is, whenever practical, to afford the public an opportunity to participate in the rulemaking process. Accordingly, interested persons may submit written comments, suggestions, or objections regarding this proposed rule.

The primary author of this document is Kathleen L. Slover, Branch of Tribal Enrollment Services, Division of Tribal Government Services, Bureau of Indian Affairs, telephone number (703) 235-8275.

The Department of the Interior has determined that this document is not a major rule under the criteria established by Executive Order 12291 and does not have a significant economic effect on a substantial number of small entities under the criteria established by the Regulatory Flexibility Act.

List of Subjects in 25 CFR Part 43d

Indians—claims, Indians—enrollment.

Subchapter F of Chapter I of Title 25 of the Code of Federal Regulations is hereby amended by the addition of a new part to read as follows:

PART 43d—PREPARATION OF A MEMBERSHIP ROLL OF THE PRIBILOF ISLANDS ALEUT COMMUNITIES OF ST. PAUL AND ST. GEORGE

Sec.
43d.1 Definitions.
43d.2 Purpose
43d.3 Information Collection.
43d.4 Qualifications for enrollment and the deadline for filing applications.
43d.5 Notices.
43d.6 Application forms.
43d.7 Burden of proof.
43d.8 Action by the Communities.
43d.9 Action by the Superintendent.

Sec.
43d.10 Appeals.
43d.11 Decision of the Secretary on appeals.
43d.12 Preparation of roll.
43d.13 Certification and approval of the roll.
43d.14 Special instructions.

Authority: 5 U.S.C 301; 25 U.S.C. 2 and 9, and 87 Stat. 466.

§ 43d.1 Definitions.

As used in these regulations:

(a) "Adopted person" means a person whose natural parents' parental rights have been terminated by court order and given to others to exercise.

(b) "ANCSA" means the Alaska Native Claims Settlement Act of December 18, 1971, Pub. L. 92-203, 85 Stat. 688, as amended and supplemented by the Act of January 2, 1976, Pub. L. 94-204, 89 Stat. 114.

(c) "Assistant Secretary" means the Assistant Secretary of the Interior for Indian Affairs or his/her authorized representative.

(d) "Communities" means the Pribilof Islands Aleut Communities of St. Paul and St. George.

(e) "Community Council" means the governing body of the Aleut Community of St. Paul Island or the governing body of the Aleut Community of St. George Island.

(f) "Director" means the Area Director, Juneau Area office, Bureau of Indian Affairs or his/her authorized representative acting under delegated authority.

(g) "Enrollment Committee " means the committee for the Aleut Community of St. Paul Island or the Aleut Community of St. George Island appointed by the Community Councils to perform duties relating to the enrollment of members.

(h) "Living" means born on or prior to and living on June 22, 1980.

(i) "Minor Children" means persons who are less than eighteen years of age on the date specified.

(j) "Plan" means the plan for the use and distribution of Pribilof Islands judgment funds awarded in Docket 352 and 369-A before the U.S. Court of Claims, prepared pursuant to the Act of October 19, 1973, Pub. L. 93-134, 87 Stat. 466 and effective June 22, 1980.

(k) "St. George Tanaq" means the village corporation created pursuant to ANCSA, comprised of those Alaska Natives who were determined eligible and whose Permanent Residence as of April 1, 1970, for the purposes of ANCSA, was St. George, Alaska.

(l) "Secretary" means the Secretary of the Interior on his/her authorized representative.

(m) "Sponsor" means a parent, recognized guardian, next friend, next of kin, spouse, executor or administrator of estate, the Superintendent, or other person who files an application for enrollment on behalf of another person.

(n) "Superintendent" means the Superintendent, Anchorage Agency, Bureau of Indian Affairs of his/her authorized representative acting under delegated authority.

(o) "Tanadgusix" means the village corporation created pursuant to ANCSA, comprised of those Alaska Natives who were determine eligible and whose Permanent Residence as of April 1, 1970, for the purposes of ANCSA, was St. Paul, Alaska.

§ 43d.2 Purpose.

The regulations in this part are to govern the compilation of a roll of members of the Pribilof Islands Aleut Communities of St. Paul and St. George eligible to share in the distribution of judgment funds awarded the Pribilof Islands by the U.S. Court of Claims in Dockets 352 and 369-A.

§ 43d.3 Information collection.

The information collection requirement contained in § 43d.4(b) has been submitted to the Office of Management and Budget for approval as required by 44 U.S.C. 3507. The collection of this information will not be required until it has been approved by the Office of Management and Budget. The information will be collected in order to prepare a roll of members of the Pribilof Islands Aleut Communities of St. Paul and St. George living on June 22, 1980. The information will be used to determine the eligibility of each applicant for enrollment. The obligation to respond is voluntary, but is a requirement in order to be eligible to share in the distribution of judgment funds awarded the Pribilof Islands by the U.S. Court of Claims.

§ 43d.4 Qualifications for enrollment and the deadline for filing applications.

The roll shall contain the names of persons living on June 22, 1980, who meet the following requirements:

(a) They are of Aleut descent; and:

(1) They resided on St. Paul or St. George Island on June 22, 1980; *Provided that,* any person of Aleut descent enrolled to Tanadfusix Corporation or St. George Tanaq Corporation shall be presumed to have resided in the Communities, and all persons of Aleut descent who were absent from the Communities for purposes of their own education, service in the United States armed forces, or personal health shall be presumed to have resided in the Communities on June 22, 1980; or

(2) They were minor children on June 22, 1980, of persons who qualify for enrollment under paragraph (a) of this section (1), or

(3) They were born on either St. Paul of St. George Island on or before December 31, 1946; and

(b) They file or have filed on their behalf an application with the Superintendent, Anchorage Agency, Bureau of Indian Affairs, P.O. Box 120, Anchorage, Alaska 99510. Application forms must be received by the Superintendent no later than close of business on (210 days from date of publication). Applications received after that date will be rejected for inclusion on the roll being prepared for failure to file on time regardless of whether the applicant otherwise meets the requirements for enrollment. If the filing deadline falls on a Saturday, Sunday, legal holiday, or other nonbusiness day, the deadline will be the next working day thereafter.

§ 43d.5 Notices

(a) The Director shall mail a notice to each person who applied for enrollment under ANCSA at the last address of record and whose application on which his/her determination of eligibility was based indicated that he/she was of Aleut descent.

(b) The notice shall advise of the preparation of a membership roll pursuant to the Plan and the relevant procedures to be followed including the requirements for enrollment, and the need to file or have filed on their behalf a completed application form before the deadline specified in § 43d.4(b) in order to be eligible to share in the distribution of judgment funds. The notice shall also state how and where application forms may be obtained as well as the name, address, and telephone number of a person who may be contacted for further information.

§ 43d.6 Application forms.

(a) Application forms to be filed by or for applicants for enrollment will be furnished by the Superintendent, or other designated persons, upon written or oral request. Each person furnishing application forms shall keep a record of the names of individuals to whom forms are given, as well as the control numbers of the forms and the date furnished. Instructions for completing and filing applications shall be furnished with each form. The form shall indicate prominently the deadline for filing applications.

(b) Among other information, each applications form shall contain:

(1) Certification as to whether application is for a natural child or an adopted child of the parent through whom eligibility is claimed.

(2) If the application is filed by a sponsor, the name and address of sponsor and relationship to applicant.

(3) A control number for the purpose of keeping a record of forms furnished interested individuals.

(c) Application forms may be filed by sponsors on behalf of other persons.

(d) Every applicant or sponsor shall furnish the applicant's mailing address on the application form. Thereafter, he/she shall promptly notify the Superintendent of any change in address, giving appropriate identification of the application, otherwise the mailing address as stated on the form shall be acceptable as the proper address for all purposes under the regulations in this part.

(e) Criminal penalties of a $10,000 fine for five(5) years in prison, or both, are provided by statute for knowingly filing false information in such applications (18 U.S.C. 1001).

§ 43d.7 Burden of proof.

The burden of proof of eligibility for enrollment rests upon the applicant. Documentary evidence such as birth certificates, death certificates, baptismal records, adoption records, copies of probate findings, affidavits, medical records, Armed Forces service records, or other records acceptable to the Secretary, must be used to support claims for enrollment. Records of the Bureau of Indian Affairs or other U.S. Government Agencies may also be used to establish eligibility.

§ 43d.8 Action by the Communities.

The Superintendent shall submit copies of all applications received to the appropriate Enrollment Committee for review and determination; except that, in cases of adopted persons where the Bureau of Indian Affairs has assured confidentiality in order to obtain the information necessary to determine the eligibility for enrollment of the individual, such confidential information will not be released to the Communities, but the Superintendent shall certify as to the eligibility for membership of such applicant to the Communities. The appropriate Enrollment Committee shall review all applications and make their decision in writing stating the reason(s) for approval or rejection of the applicant for membership in the Communities. The Community Council, by resolution, will make the final decision for the Communities. If the Community Council's decision reverses the Enrollment Committee's decision, the reason(s) for approval or rejection of the applicant for membership in the Communities must be stated in writing. The application shall then be returned to the Superintendent with the decision and any additional evidence used in determining eligibility for membership in the Communities. Approval of the applicant for membership in the Communities by the Community Council does not insure eligibility to share in the distribution of judgment funds.

§ 43d.9 Action by the Superintendent.

(a) The Superintendent shall consider each application, all documentation, and the Community Council's decision. The Superintendent shall accept the decision of the Community Council unless the decision is clearly erroneous. If the Superintendent overrules the Community Council's decision, he/she shall notify the Community Council of his/her actions and the reasons therefore. The determination of the Superintendent shall only affect the applicant's eligibility to share in the distribution of the judgment funds.

(b) Upon determining an applicant's eligibility, the Superintendent shall notify the applicant or sponsor, as applicable, in writing of his/her decision. If the decision is favorable, the name of the applicant shall be placed on the roll. If the Superintendent decides the applicant is not eligible, he/she shall notify the applicant or sponsor, as applicable, in writing by certified mail, to be received by the addressee only, return receipt requested, and shall explain fully the reasons for rejection and of the right to appeal to the Secretary. If correspondence is sent out of the United States, it may be necessary to use registered mail. If an individual files applications on behalf of more than one person, one notice of eligibility or rejection may be addressed to the person who filed the applications. However, said notice must list the name of each person involved. If a certified or registered notice is returned as "Unclaimed" the Superintendent shall remail the notice by regular mail together with an acknowledgement of receipt form to be completed by the addressee and returned to the Superintendent. If the acknowledgement of receipt is not returned, computation of the appeal period shall begin on the date the notice was remailed. Certified or registered notices returned for any reason other than "Unclaimed" need not be remailed.

(c) A notice of rejection is considered to have been made on the date:

(1) Of delivery indicated on the return receipt,

(2) Of acknowledgement of receipt,

(3) Of personal delivery, or

(4) Of the return by the post office of an undelivered certified or registered letter.

(d) In all cases where an applicant is represented by an attorney, such attorney will be recognized as fully controlling the case on behalf of his/her client and service on the attorney of any document relating to the application shall be considered to be service on the applicant he/she represents. Where an applicant is represented by more than one attorney, service upon one of the attorneys shall be sufficient.

(e) To avoid hardship or gross injustice, the Superintendent may waive technical deficiencies in applications or other submissions. Failure to file by a deadline does not constitute a technical deficiency.

§ 43d.10 Appeals.

Appeals from rejected applicants must be in writing and must be filed pursuant to Part 42 of this subchapter, a copy of which shall be furnished with each notice of rejection.

§ 43d.11 Decision of the Secretary on appeals.

The decision of the Secretary on an appeal shall be final and conclusive and written notice of the decision shall be given to the applicant or sponsor. When so directed by the Secretary, the Assistant Secretary shall cause to be entered on the roll the name of any person whose appeal has been sustained.

§ 43d.12 Preparation of roll.

The Superintendent shall prepare a minimum of five (5) copies of the roll of those persons determined to be eligible for enrollment, including those who appeals were sustained. In addition to other information which may be shown, the complete roll shall contain for each person an identification number, full name, address, sex, date of birth, date of death (if applicable), and the authority for enrollment.

§ 43d.13 Certification and approval of the roll.

A certificate shall be attached to the roll by the Superintendent certifying that to the best of his/her knowledge and belief the roll contains only the names of those persons who were determined to meet the requirements for enrollment. The Director shall approve the roll.

§ 43d.14 Special instructions.

To facilitate the work of the Superintendent the Assistant Secretary may issue special instructions not

inconsistent with the regulations in this Part.

John W. Fritz,

Deputy Assistant Secretary—Indian Affairs (Operations)

[FR Doc. 82-10859 Filed 4-20-82; 8:45 am]

BILLING CODE 4310-02-M

Geological Survey

30 CFR Part 221

Site Security; Onshore Federal and Indian Oil and Gas Leases

AGENCY: Minerals Management Service, Interior.

ACTION: Notice of intent to propose rulemaking.

SUMMARY: The Minerals Management Service (MMS), in order to reduce the circumstances which could facilitate the theft or mishandling of crude oil, intends to develop minimum site security requirements to be followed by the private sector when operating on, or for the benefit of, onshore Federal and Indian (except OSAGE) oil and gas leases which are subject to the regulations contained in Title 30 CFR Part 221. Interested parties are invited to submit comments and recommendations to assist the MMS in development of such site security requirements and related enforcement actions.

DATE: Comments must be received by May 21, 1982.

ADDRESS: Send comments to Director, Minerals Management Service, 12203 Sunrise Valley Drive, Mail Stop 656, Reston, Virginia 22091.

FOR FURTHER INFORMATION CONTACT: Mr. Gerald R. Daniels, (703) 860-7535, (FTS) 928-7535; or Mr. Cecil Feeney, (703) 860-6259, (FTS) 928-6259.

SUPPLEMENTARY INFORMATION: There is evidence that crude oil and condensate (crude oil) is being stolen and mishandled at onshore sites throughout the United States, including sites on onshore Federal and Indian lands. It has been determined that the opportunities for theft or mishandling or crude oil are related directly to the quality of the site security provided at operational sites where crude oil is produced, separated, treated, stored, and marketed. The results of the inspections conducted by the MMS over the past 18 months and the recently completed study by the Commission on Fiscal Accountability of the Nation's Energy Resources (Commission) both are indicative that security at operational sites on onshore Federal and Indian leases frequently is lax and, thus, offers an open invitation to theft or mishandling of crude oil. The Commission concluded that site security is the primary responsibility of those who operate the onshore Federal and Indian oil and gas leases. The Secretary of the Interior (Secretary) concurred in that conclusion, and the Secretary subsequently issued a notice to that effect to all lessees and operators of onshore Federal and Indian leases and to the major national and regional oil and gas associations.

The Secretary, in implementing the Commission's recommendations for improving site security, has directed the MMS to develop regulations that (1) establish the minimum site security requirements for onshore Federal and Indian oil and gas leases, (2) provide for the development and submission of a site security plan by each individual operator, and (3) specify the range of enforcement actions to be taken in the event of a failure to comply with such site security requirements or an operator's more stringent site security plan.

The MMS is seeking information from interested parties to assist in the development of these regulatory requirements prior to the publication of proposed rulemaking. Specific comments are sought on the following:

1. Do you currently have a site security plan or plans in effect? If so, please submit with your comments a copy of your plan(s) or provide a synopsis of the major components. Plans or synopses thereof which are submitted will be held confidential on request.

2. Are you experiencing problems with theft or mishandling of crude oil from the Federal and Indian oil and gas leases which you operate? If so, what is the present magnitude of these problems and has there been an increase recently?

3. How do you react to a suspected or confirmed incident of theft or mishandling of crude oil which occurs on Federal and Indian leases that you operate? For example, do you report these incidents to local, State, and Federal law enforcement agencies and to the MMS, alter equipment and/or procedures, increase surveillance, etc.?

4. Based on your past experience and knowledge of the operating environment, which points in the production, separation, treating, storage, and marketing phases do you believe to be the most vulnerable to the theft or mishandling of crude oil?

5. On the basis of your past experience and views as to vulnerability, what minimum site security requirements do you consider as necessary to effectively deter theft or mishandling of crude oil in a cost-effective manner.

6. Once the final MMS regulations for site security requirements have been issued, what is a reasonable period of time for an operator to (a) develop and submit a site security plan and (b) bring all of its facilities into compliance with such requirements?

7. Where an individual operator has a site security plan that exceeds the minimum requirements, should the MMS inspect for compliance with the total plan or only as to the minimum requirements of MMS?

8. Are there circumstances that might justify an approved deviation from the site security requirements ultimately adopted by the MMS? If so, cite examples.

9. Should MMS hold the site security plans filed with it confidential on the basis that indiscriminate disclosure could abet those intent on the theft or mishandling of crude oil?

10. In addition to the inspections carried out by the MMS, should the operators be required to conduct a periodic, self-inspection program for compliance with the site security requirements and report the results to the MMS?

11. When MMS identifies a violation of the site security requirements at a facility, how much time should the operator be given to correct the situation?

12. What range of enforcement actions should be provided for violations of the site security requirements? For example, citation and opportunity to correct within a reasonable period of time for first violation; shut-in for failure to timely correct or second violation of the same requirement at the same facility or at other similar facilities in the area; shut-in and the assessment of penalties for repeated violations or a continuing failure to exercise due care and diligence; or lease cancellation when all previous enforcement actions fail to result in compliance.

List of Subjects in 30 CFR Part 221

Government contracts, Minerals royalties, Oil and gas exploration, Public lands mineral resources, Reporting and recordkeeping requirements.

Dated: April 9, 1982.

William P. Pendley,

Acting Assistant Secretary of the Interior.

[FR Doc. 82-10852 Filed 4-20-82; 8:45 am]

BILLING CODE 4310-MR-M

National Park Service

36 CFR Part 7

Olympic National Park; Snowmobile Regulations

AGENCY: National Park Service, Interior.

ACTION: Proposed rule.

SUMMARY: The proposed regulation set forth below is necessary to designate locations within Olympic National Park where snowmobiles may be used for recreational purposes when portions of motor roads are covered by snow and closed to use by normal motor vehicle traffic. It is the objective of this proposed regulation to provide for the preservation and enjoyment of Olympic National Park in a way that is consistent with both the snowmobile policy of the National Park Service and off-road vehicle policy of the Department of the Interior.

DATES: Written comments, suggestions or objections will be accepted until May 21, 1982.

ADDRESS: Comments should be directed to: Superintendent, Olympic National Park, 600 East Park Avenue, Port Angeles, Washington 98362.

FOR FURTHER INFORMATION CONTACT: Roger J. Contor, Superintendent, Olympic National Park, Telephone: (206) 452-4501.

SUPPLEMENTARY INFORMATION:

Background

Executive Order 11644 (Use of Off-Road Vehicles on the Public Lands) directed Federal land managing agencies to develop unified regulations and to designate areas of use for off-road vehicles. Such areas must meet criteria which minimize resource damage, harassment of wildlife, disruption of wildlife habitat, and, in the case of national parks, not adversely affect scenic, natural and aesthetic values.

In response to Executive Order 11644, the Secretary of the Interior issued a Departmental memorandum on May 5, 1972, to assure full compliance with the Order and to provide policies and procedures for its implementation. The National Park Service, as required by the above directive, promulgated 36 CFR 2.34 (c) on April 1, 1974, which closed all National Park System areas to snowmobile use except those specifically designated as open by **Federal Register** notice or special regulation.

In order to comply with the requirements of Executive Order 11644 and 36 CFR 2.34, the National Park Service developed a Servicewide policy revision which was published in the **Federal Register** on August 13, 1979 (44 FR 4712). This policy provides for the use of snowmobiles in units of the National Park System as a mode of transportation to provide the opportunity for visitors to see, sense and enjoy the special qualities of the park in the winter. The snowmobile use must be consistent with the park's natural, cultural, scenic and aesthetic values; safety considerations; park management objectives; and not disturb the wildlife or damage other park resources.

The policy further provides that, where permitted, snowmobile use shall be confined to properly designated routes and water surfaces which are used by motorized vehicles or motorboats during other seasons. Routes and water surfaces to be designated for snowmobile use shall be promulgated as special regulations in the Code of Federal Regulations.

This proposed regulation is necessary to comply with Servicewide policy. Its promulgation also is a liberalization of the present *no* snowmobiling activity and allows additional recreational opportunities along designated motor road surfaces within Olympic National Park when snow conditions are such that the motor road is closed to public automobile travel. The designated routes for snowmobiles will be that portion of Staircase Road from the park boundary to the Staircase Ranger Station; Whiskey Bend Road from the junction of Elwha Road to the Whiskey Bend trailhead; Boulder Creek Road from Glines Canyon Dam to end of road; North Fork Quinault Road from the end of the plowed portion to the North Fork Ranger Station; South Shore Road from the end of the plowed portion to the Graves Creek Ranger Station.

Public Participation

The policy of the Department of the Interior is, here whenever practical, to afford the public an opportunity to participate in the rulemaking process. Accordingly, interested persons may submit written comments, suggestions or objections regarding the proposed regulation to the address noted at the beginning of the rulemaking.

Drafting Information

The following persons participated in the writing of this regulation: Gordon D. Boyd, Chief Ranger; Paul Crawford, Resource Management Specialist; Robert C. Marriott, Park Ranger; all of Olympic National Park.

Compliance With Other Laws

The Department of the Interior has determined that this document is not a "major rule" within the meaning of Executive Order 12291 (46 FR 13193; February 19, 1981) and that this rulemaking will not have a "significant economic effect on a substantial number of small entities" that will require the preparation of a regulatory analysis within the meaning of the Regulatory Flexibility Act, 94 Stat. 1164, 5 U.S.C. 601 *et seq.*

Pursuant to the National Environmental Policy Act (42 U.S.C. 4332) the Service has prepared an environmental assessment on this proposed regulation which is available at the address noted above.

This rule does not contain an information collection or recordkeeping requirement as defined in the Paperwork Reduction Act, 94 Stat. 2812, 44 U.S.C. 3501 *et seq.*

(Sec. 3 of the Act of August 25, 1916 (39 Stat. 535, as amended; 16 U.S.C. 3))

List of Subjects in 36 CFR Part 7

National parks.

PART 7—SPECIAL REGULATIONS, AREAS OF THE NATIONAL PARK SYSTEM

In consideration of the foregoing, it is proposed to amend 36 CFR 7.28 by adding a new paragraph (f) as follows:

§ 7.28 Olympic National Park.

* * * * *

(f) *Snowmobile use* (1) The use of snowmobiles is prohibited except in areas and on routes designated by the superintendent by the posting of appropriate signs or by marking on a map available at the office of the superintendent, or both. The following routes have been designated for snowmobile use within Olympic National Park:

(i) Staircase Road from the park boundary to the Staircase Ranger Station.

(ii) Whiskey Bend Road from the junction of Elwha road to the Whiskey Bend trailhead.

(iii) Boulder Creek Road from Glines Canyon Dam to the end of the road.

(iv) North Fork Quinault Road from the end of the plowed portion to the North Fork Ranger Station.

(v) South Shore Road from the end of the plowed portion to the Graves Creek Ranger Station.

(2) On roads designated for snowmobile use, only that portion of the roadway intended for wheeled vehicle use may be used by snowmobiles. Such roadway is available for snowmobile use only when the designated roadway

is closed to all other public motor vehicle use by snow.

J. Craig Potter,

Acting Assistant Secretary for Fish and Wildlife and Parks.

March 26, 1982.

[FR Doc. 82-10876 Filed 4-20-82; 8:45 am]

BILLING CODE 4310-70-M

ENVIRONMENTAL PROTECTION AGENCY

40 CFR Part 180

[PP 2F2642/P222; PH-FRL-2105-7]

Potassium Sorbate; Proposed Technical Amendment

AGENCY: Environmental Protection Agency (EPA).

ACTION: Proposed rule.

SUMMARY: This notice of proposed rulemaking adds potassium sorbate to the chemicals listed in 40 CFR 180.2(a) as a pesticide chemical generally recognized as safe when used as a pre- or postharvest fungicide for purposes of section 408(a) of the Federal Food, Drug, and Cosmetic Act. This amendment is being proposed upon the Agency's own initiative.

DATE: Written comments must be received on or before May 21, 1982.

ADDRESS: Written comments to: Henry M. Jacoby, Product Manager (PM) 21, Registration Division (TS-767C), Environmental Protection Agency, 401 M St., SW., Washington, DC 20460.

FOR FURTHER INFORMATION CONTACT: Henry M. Jacoby (703-557-1900).

SUPPLEMENTARY INFORMATION: Residues of potassium sorbate (potassium salt or sorbic acid) are exempt from the requirement of a tolerance when used in accordance with good agricultural practices as an inert ingredient as a preservative in pesticide formulations applied to growing crops or to raw agricultural commodities after harvest (§ 180.1001(c)). The presence of potassium sorbate is a common daily occurrence in the human diet in the form of a preservative in dairy, baked goods, and fruit and vegetable products. Concentrations generally range from 0.05 percent (500 parts per million (ppm)) to 0.3 percent (3,000 ppm) by weight in such common food items as cake, margarine, and cheese. The parent compound sorbic acid was isolated in 1859 from the berries of the mountain ash as parasorbic acid; its antimicrobial activity was discovered in 1939; and since 1950 it has been used in many countries as a food preservative.

The Food and Drug Administration lists potassium sorbate as generally recognized as safe (GRAS) when used in good manufacturing practice as a direct food additive (21 CFR 182.3640).

Considering the extensive use, history and scientific investigation supporting the relative safety of potassium sorbate, it is reasonable to conclude that the proposed inclusion of potassium sorbate to 40 CFR 180.2(a) as a pesticide chemical considered safe for use will protect the public health.

The subject material is considered useful for the purpose for which this proposed regulation is sought and it is concluded that it will protect the public health. It is proposed, therefore, that the regulation be established as set forth below.

Any person who has registered or submitted an application for registration of a pesticide, under the Federal Insecticide, Fungicide, and Rodenticide Act (FIFRA) as amended, which contains any of the ingredients listed herein, may request on or before May 21, 1982 that this rulemaking proposal be referred to an Advisory Committee in accordance with section 408(e) of the Federal Food, Drug, and Cosmetic Act.

Interested persons are invited to submit written comments on the proposed regulation. Comments must bear a notation indicating the document control number, "[PP 2F2642/P222]". All written comments filed in response to this proposed regulation will be available for public inspection in the office of Henry Jacoby at the above address from 8:00 a.m. to 4:00 p.m., Monday through Friday, except legal holidays.

The Office of Management and Budget has exempted this rule from the requirements of section 3 of Executive Order 12291.

Pursuant to the requirements of the Regulatory Flexibility Act (Pub. L. 96-534, 94 Stat. 1164, 5 U.S.C. 601-612), the Administrator has determined that regulations establishing new tolerances or raising tolerance levels or establishing exemptions from tolerance requirements do not have a significant economic impact on a substantial number of small entities. A certification statement to this effect was published in the **Federal Register** of May 4, 1981 (46 FR 24950).

(Sec. 408(e), 68 Stat. 514 (21 U.S.C. 346a(e)))

List of Subjects in 40 CFR Part 180

Administrative practice and procedure, Agricultural commodities, Pesticides and pests.

Dated: April 7, 1982.

Edwin L. Johnson,

Director, Office of Pesticide Programs.

PART 180—TOLERANCES AND EXEMPTIONS FROM TOLERANCES FOR PESTICIDE CHEMICALS IN OR ON RAW AGRICULTURAL COMMODITIES

Therefore, it is proposed that 40 CFR 180.2(a) be revised to read as follows:

§ 180.2 Pesticide chemicals considered safe.

(a) As a general rule, pesticide chemicals other than benzaldehyde (when used as a bee repellant in the harvesting of honey), ferrous sulfate, lime, lime-sulfur, potassium polysulfide, potassium sorbate, sodium carbonate, sodium chloride, sodium hypochlorite, sodium polysulfide, and sulfur, and, when used as postharvest fungicides, citric acid, fumaric acid, oil of lemon, oil of orange, sodium benzoate, and sodium propionate are not for the purposes of section 408(a) of the Act generally recognized as safe for use.

* * * * *

[FR Doc. 82-10688 Filed 4-20-82; 8:45 am]

BILLING CODE 6560-50-M

FEDERAL EMERGENCY MANAGEMENT AGENCY

44 CFR Part 67

[Docket No. FEMA-5938]

Proposed Flood Elevation Determinations

AGENCY: Federal Emergency Management Agency, FEMA.

ACTION: Proposed rule; revision.

SUMMARY: Technical information or comments are solicited on the proposed base (100-year) flood elevations listed below for selected locations in the City of Huntington Beach, Orange County, California.

Due to recent engineering analysis, this proposed rule revises the proposed determinations of base (100-year) flood elevations published in 45 FR 71825 on October 30, 1980 and in the *Huntington Beach Independent,* published on or about October 16, 1980, and October 23, 1980, and hence supersedes those previously published rules for the areas cited below.

DATES: The period for comment will be ninety (90) days following the second publication of this notice in a newspaper of local circulation in the above-named community.

ADDRESSES: Maps and other information showing the detailed outlines of the flood-prone areas and the proposed flood elevations are available for review at City Hall, Huntington Beach, California.

Send comments to: the Honorable Ruth Bailey, 2000 Main Street, Huntington Beach, California 92648.

FOR FURTHER INFORMATION CONTACT: Mr. Robert G. Chappell, National Flood Insurance Program, (202) 287-0230, Federal Emergency Management Agency, Washington, D.C. 20472.

SUPPLEMENTARY INFORMATION: Proposed base (100-year) flood elevations are listed below for selected locations in the City of Huntington Beach, Orange County, California, in accordance with section 110 of the Flood Disaster Protection Act of 1973 (Pub. L. 93-234), 87 Stat. 980, which added section 1363 to the National Flood Insurance Act of 1968 (Title XIII of the Housing and Urban Development Act of 1968 (Pub. L. 90-448), 42 U.S.C. 4001-4128, and 44 CFR 67.4(a)).

These base (100-year) flood elevations are the basis for the flood plain management measures that the community is required to either adopt or show evidence of being already in effect in order to qualify or remain qualified for participation in the National Flood Insurance Program (NFIP).

These modified elevations will also be used to calculate the appropriate flood insurance premium rates for new buildings and their contents and for the second layer of insurance on existing buildings and their contents.

Pursuant to the provisions of 5 U.S.C. 605(b), the Associate Director, to whom authority has been delegated by the Director, Federal Emergency Management Agency, hereby certifies that the proposed flood elevation determinations, if promulgated, will not have a significant economic impact on a substantial number of small entities. A flood elevation determination under section 1363 forms the basis for new local ordinances, which, if adopted by a local community, will govern future construction within the floodplain area. The elevation determinations, however, impose no restriction unless and until the local community voluntarily adopts floodplain ordinances in accord with these elevations. Even if ordinances are adopted in compliance with Federal standards, the elevations prescribe how high to build in the floodplain and do not proscribe development. Thus, this action only forms the basis for future local actions. It imposes no new requirement; of itself it has no economic impact.

List of Subjects in 44 CFR Part 67

Flood insurance, floodplains.

The proposed base (100-year) flood elevations are:

Source of flooding	Location	#Depth in feet above ground *Elevation in feet (NGVD)
Santa Ana River	Intersection of Edwards Street and Warner Avenue	#1
	Intersection of Padua Drive and Misty Lane	*1
	Intersection of Garfield Avenue and Ward Street	#2
	Intersection of Heil Avenue and Rhone Lane	#3
	Intersection of Atlanta Avenue and Magnolia Street	*11
	Intersection of Holburn Lane and Ramsgate Lane	*14
Pacific Ocean	Centerline of Edgewater Lane 500 feet west from the intersection of Edgewater and Waverly Lanes.	*5
	850 feet southwest from the intersection of Pacific Coast Highway and Warner Avenue.	*6
	650 feet southwest from the intersection of Pacific Coast Highway and Warner Avenue.	*13

(National Flood Insurance Act of 1968 (Title XIII of Housing and Urban Development Act of 1968), effective January 28, 1969 (33 FR 17804, November 28, 1968), as amended; 42 U.S.C. 4001-4128; Executive Order 12127, 44 FR 19367; and delegation of authority to the Associate Director)

Issued: April 6, 1982.

Lee M. Thomas,

Associate Director State and Local Programs and Support.

[FR Doc. 82-10865 Filed 4-20-82; 8:45 am]

BILLING CODE 6718-03-M

---

44 CFR Part 67

[Docket No. FEMA-6277]

National Flood Insurance Program, Proposed Zone Designation, Determinations for the City of Plano, Kendall County, Illinois

AGENCY: Federal Emergency Management Agency.

ACTION: Proposed rule.

SUMMARY: Technical information or comments are solicited on the proposed zone designations described below.

The proposed zone designations are the basis for the flood plain management measures that the community is required to either adopt or show evidence of being already in effect in order to qualify or remain qualified for participation in the National Flood Insurance Program (NFIP).

DATES: The period for comment will be ninety days following the second publication of this proposed rule in the newspaper of local circulation in the above-named community.

ADDRESSES: Maps and other information showing the detailed outlines of the flood-prone areas and the proposed zone designations are available for review at 101 West Main Street, Plano, Illinois.

Send comments to: Honorable Verlin Akers, City of Plano, 101 West Main Street, Plano, Illinois 60545.

FOR FURTHER INFORMATION CONTACT: Robert G. Chappell, P.E., Chief, Engineering Branch, Natural Hazards Division, Federal Emergency Management Agency, Washington, D.C. 20472, (202) 287-0230.

SUPPLEMENTARY INFORMATION: The Associate Director, State and Local Programs and Support gives notice of the proposed zone designations for the City of Plano, Illinois, in accordance with section 110 of the Flood Disaster Protection Act of 1973 (Pub. L. 93-234), 87 Stat. 980, which added Section 1363 to the National Flood Insurance Act of 1968 (Title XIII of the Housing and Urban Development Act of 1968 (Pub. L. 90-448)), 42 U.S.C. 4001-4128, and 44 CFR Part 67.

These zone designations and base (100-year) flood elevations, together with the flood plain management measures required by § 60.3 of the program regulations, are the minimum that are required. They should not be construed to mean the community must change any existing ordinances that are more stringent in their flood plain management requirements. The community may at any time enact stricter requirements on its own, or pursuant to policies established by other Federal, State, or regional entities. The proposed zone designations will also be used to calculate the appropriate flood insurance premium rates for new buildings and their contents.

The proposed zone designations are as follows:

Zone A and Zone C along the reach of Little Rock Creek that is within the corporate

limits, and along Big Rock Creek in portions of the community lying east of a line which proceeds from the southern corporate limits north along Hale Street to South Street, from thence continues east along South Street to a point approximately 900 feet west of Big Rock Creek, and from there goes north, following East Street for part of the way, to Big Rock Avenue, which it follows to the eastern corporate limits.

Pursuant to the provisions of 5 U.S.C. 605(b), the Associate Director, State and Local Programs and Support, to whom authority has been delegated by the Director, Federal Emergency Management Agency, hereby certifies that this rule if promulgated will not have a significant economic impact on a substantial number of small entities. This rule provides routine legal notice of technical amendments made to designated special flood hazard areas on the basis of updated information and imposes no new requirements or regulations on participating communities.

List of Subjects in 44 CFR Part 67

Flood insurance, Floodplains.

(National Flood Insurance Act of 1968 (Title XIII, Housing and Urban Development Act of 1968), effective January 28, 1969 (33 FR 17804, November 28, 1968), as amended; 42 U.S.C. 4001–4128; Executive Order 12127, 44 FR 19367; and delegation of authority to Associate Director, State and Local Programs and Support)

Issued: March 23, 1982.

Lee M. Thomas,

Associate Director, State and Local Programs and Support.

[FR Doc. 82-10854 Filed 4-20-82; 8:45 am]

BILLING CODE 6718-03-M

44 CFR Part 67

[Docket No. FEMA-6276]

National Flood Insurance Program; Proposed Zone Designation and Base Flood Elevation Determinations for the Village of South Roxana, Madison County, Illinois

AGENCY: Federal Emergency Management Agency.

ACTION: Proposed rule.

SUMMARY: Technical information or comments are solicited on the proposed base flood elevations and zone designations described below.

The proposed base flood elevations and zone designations are the basis for the flood plain management measures that the community is required to either adopt or show evidence of being already in effect in order to qualify or remain qualified for participation in the National Flood Insurance Program (NFIP).

DATES: The period for comment will be ninety-days following the second publication of this proposed rule in the newspaper of local circulation in the above-named community.

ADDRESSES: Maps and other information showing the detailed outlines of the flood-prone areas and the proposed base flood elevations and zone designations are available for review at 211 Sinclair Avenue, South Roxana, Illinois. Send Comments to: Monte R. Anderson, Village President, Village of South Roxana, P.O. Box 107, South Roxana, Illinois 62087.

FOR FURTHER INFORMATION CONTACT: Robert G. Chappell, P.E., Chief, Engineering Branch, Natural Hazards Division, Federal Emergency Management Agency, Washington, D.C. 20472, (202) 287-0230.

SUPPLEMENTARY INFORMATION: The Associate Director, State and Local Programs and Support gives notice of the proposed base flood elevations and zone designations for the Village of South Roxana, Illinois, in accordance with section 110 of the Flood Disaster Protection Act of 1973 (Pub. L. 93-234), 87 Stat. 980, which added section 1363 to the National Flood Insurance Act of 1968 (Title XIII of the Housing and Urban Development Act of 1968 (Pub. L. 90-448)), 42 U.S.C. 4001-4128, and 44 CFR Part 67.

These zone designations and base (100-year) flood elevations, together with the flood plain management measures required by § 60.3 of the program regulations, are the minimum that are required. They should not be construed to mean the community must change any existing ordinances that are more stringent in their flood plain management requirements. The community may at any time enact stricter requirements on its own, or pursuant to policies established by other Federal, State, or regional entities. The proposed base flood elevations and zone designations will also be used to calculate the appropriate flood insurance premium rates for new buildings and their contents.

List of Subjects in 44 CFR Part 67

Flood insurance, Floodplains.

The proposed zone designations and base flood elevations are as follows:

Source of flooding	location	Elevation (NGVD) (feet)	Zone designation
Local runoff and ponding	Portions of the area bounded on the north by a line parallel to and approximately 100 feet south of Park Street, on the east by a line parallel to and approximately 250 feet west of Smith Avenue, and on the south and west by the corporate limits.	428	A4
	Portions of the area bounded on the north by Daniel Boone Trail, on the east by a line which is first directed south from Daniel Boone Trail along a line parallel to and approximately 150 feet west of Sinclair Avenue until it reaches a point approximately 2,150 feet south of Daniel Boone Trail, there the line turns west until it reaches State Highway 111, where it is again directed south and proceeds to the southern corporate limits, and on the south and west by the corporate limits.	428	A4

Pursuant to the provisions of 5 U.S.C. 605(b), the Associate Director, State and Local Programs and Support, to whom authority has been delegated by the Director, Federal Emergency Management Agency, hereby certifies that this rule if promulgated will not have a significant economic impact on a substantial number of small entities. This rule provides routine legal notice of technical amendments made to designated special flood hazard areas on the basis of updated information and imposes no new requirements of regulations on participating communities.

(National Flood Insurance Act of 1968 (Title XIII of Housing and Urban Development Act of 1968), effective January 28, 1969 (33 FR 17804, November 28, 1968), as amended; 42 U.S.C. 4001–4128; Executive Order 12127, 44 FR 19367; and delegation of authority to Associate Director, State and Local Programs and Support)

Issued: March 23, 1982.

Lee M. Thomas,

Associate Director, State and Local Programs and Support.

[FR Doc. 82-10933 Filed 4-20-82; 8:45 am]

BILLING CODE 6718-03-M

44 CFR Part 67

[Docket No. FEMA-6275]

National Flood Insurance Program; Proposed Zone and Base Flood Elevation Determinations for the City of Sedgwick, Harvey, and Sedgwick Counties, Kansas

AGENCY: Federal Emergency Management Agency.

ACTION: Proposed rule.

SUMMARY: Technical information or comments are solicited on the proposed zones and base flood elevations as described below.

The proposed zones and base flood elevations are the basis for the flood plain management measures that the community is required to either adopt or show evidence of being already in effect in order to qualify or remain qualified for participation in the National Flood Insurance Program (NFIP).

DATES: The period for comment will be ninety (90) days following the second publication of this proposed rule in the newspaper of local circulation in the above-named community.

ADDRESSES: Maps and other information showing the detailed outlines of the flood-prone areas and the proposed zones and base flood elevations are available for review at the Office of the City Clerk, City Hall, 511 North Commercial, Sedgwick, Kansas.

Send comments to: Honorable Tom Trower, Mayor, City of Sedgwick, P.O. Box 131, Sedgwick, Kansas 67135.

FOR FURTHER INFORMATION CONTACT: Mr. Robert G. Chappell, P.E. Chief, Engineering Branch, Natural Hazards Division, Office of Natural and Technological Hazards, Federal Emergency Management Agency, Washington, DC 20472, (202) 287-0216.

SUPPLEMENTARY INFORMATION: The Associate Director, State and Local Programs and Support, gives notice of the proposed zones and base flood elevations for the City of Sedgwick, Kansas, in accordance with Section 110 of the Flood Disaster protection Act of 1973 (Pub. L. 93-234), 87 Stat. 980, which added section 1363 to the National Flood Insurance Act of 1968 (Title XIII of the Housing and Urban Development Act of 1968, Pub. L. 90-448), 42 U.S.C. 4001-4128, and 44 CFR Part 67.

These zones and base flood elevations, together with the flood plain management measures required by § 60.3 of the program regulations, are the minimum that are required. It should not be construed to mean the community must change any existing ordinances that are more stringent in their flood plain management requirements. The community may at any time enact stricter requirements on its own, or pursuant to policies established by other Federal, State, or regional entities. The proposed zones and base flood elevations will also be used to calculate the appropriate flood insurance premium rates for new buildings and their contents and for the second layer of insurance on existing buildings and their contents.

List of Subjects in 44 CFR Part 67

Flood insurance, Floodplains.

The proposed base (100-year) flood elevations and zone designations for selected locations are:

Source of flooding	Location	Elevation (NGVD) (feet)	Zone designation
Little Arkansas River	At the southernmost corporate limits	1374	A4.
	Just downstream of Sixth Street	1375	A4.
Sand Creek	Just downstream of Madison Avenue	1376	A4.
	Just upstream of Madison Avenue	1377	A3.
	At the northernmost corporate limits	1379	A3.
Sedgwick Ditch	At First Street	1377	AH.
	Just north of Fourth Street	1378	AH.
	At the northernmost corporate limits	1380	AH.

Pursuant to the provisions of 5 U.S.C. 605(b), the Associate Director, State and Local Programs and Support, to whom authority has been delegated by the Director, Federal Emergency Management Agency, hereby certifies that this rule if promulgated will not have a significant economic impact on a substantial number of small entities. This rule provides routine legal notice of technical amendments made to designated special flood hazard areas on the basis of updated information and imposes no new requirements or regulations on participating communities.

(National Flood Insurance Act of 1968 (Title XIII of Housing and Urban Development Act of 1968), effective January 28, 1969 (33 FR 17804, November 28, 1968), as amended; 42 U.S.C. 4001-4128; Executive Order 12127, 44 FR 19367; and delegation of authority to the Associated Director, State and Local Programs and Support)

Issued: March 22, 1982.

Lee M. Thomas,

Associate Director, State and Local Programs and Support.

[FR Doc. 82-10934 Filed 4-20-82; 8:45 am]

BILLING CODE 6718-03-M

44 CFR Part 67

[Docket No. FEMA-6274]

National Flood Insurance Program; Proposed Base Flood Elevations and Zone Designations for the City of Lafayette, Lafayette Parish, Louisiana

AGENCY: Federal Emergency Management Agency.

ACTION: Proposed rule.

SUMMARY: Technical information or comments are solicited on the proposed base flood elevations and zone designations as described below.

The proposed base flood elevations and zone designations are the basis for the flood plain management measures that the community is required to either adopt or show evidence of being already in effect in order to qualify or remain qualified for participation in the National Flood Insurance Program (NFIP).

DATES: The period for comment will be ninety (90) days following the second publication of this proposed rule in the newspaper of local circulation in the above-named community.

ADDRESSES: Maps and other information showing the detailed outlines of the flood-prone areas and the proposed base flood elevations and zone designations are available for review at the Office of the Flood Plain Management Administrator, 707 West University Avenue, Lafayette, Louisiana.

Send comments to: Honorable Dud Lastrapes, Mayor, City of Lafayette, P.O. Box 4017-C, Lafayette, Louisiana 70502.

FOR FURTHER INFORMATION CONTACT: Mr. Robert G. Chappell, P.E., Chief, Engineering Branch, Natural Hazards Division, Office of Natural and Technological Hazards, Federal Emergency Management Agency, Washington, D.C. 20472, (202) 287-0216.

SUPPLEMENTARY INFORMATION: The Associate Director, State and Local Programs and Support, gives notice of the proposed base flood elevations and zone designations for the City of Lafayette, Louisiana in accordance with Section 110 of the Flood Disaster Protection Act of 1973 (Pub. L. 93-234), 87 Stat. 980, which added section 1363 to the National Flood Insurance Act of 1968 (Title XIII of the Housing and Urban Development Act of 1968, Pub. L.

90-448), 42 U.S.C. 4001-4128, and 44 CFR Part 67.

These base flood elevations and zone designations, together with the flood plain management measures required by § 60.3 of the program regulations, are the minimum that are required. It should not be construed to mean the community must change any existing ordinances that are more stringent in their flood plain management requirements. The community may at any time enact stricter requirements on its own, or pursuant to policies established by other Federal, State, or regional entities. The proposed base flood elevations and zone designations will also be used to calculate the appropriate flood insurance premium rates for new buildings and their contents and for the second layer of insurance on existing buildings and their contents.

List of Subjects in 44 CFR Part 67

Flood insurance, Floodplains.

The proposed base flood elevations and zone designations are as follows:

Source of flooding and location	Elevation (feet) national geodetic vertical datum	Zone designation
Coulee Bend: In the northwestern portion of the city.	40	A2.
Coulee Mine: North of the Southern Pacific Railroad.	35	A3.
Coulee Des Poches:		
Approximately 3,800 feet downstream of the limit of detailed study.	26	A2.
Approximately 1,100 feet downstream of the limit of detailed study.	29	A2.
At the limit of detailed study.....	30	A1.

The remaining annexed areas throughout the City have been identified as Zones A, B and C. In addition, the flood boundaries were adjusted throughout the City to coincide with additional contour information. The proposed floodway has also been added along the above tabulated portions of Coulee Bend and Coulee Des Poches.

Pursuant to the provisions of 5 U.S.C. 605(b), the Associate Director, State and Local Programs and Support, to whom authority has been delegated by the Director, Federal Emergency Management Agency, hereby certifies that this rule if promulgated will not have a significant economic impact on a substantial number of small entities. This rule provides routine legal notice of technical amendments made to designated special flood hazard areas on the basis of updated information and imposes no new requirements or regulations on participating communities.

(National Flood Insurance Act of 1968 (Title XIII of Housing and Urban Development Act of 1968), effective January 28, 1969 (33 FR 17804, November 28, 1968), as amended; 42 U.S.C. 4001-4128; Executive Order 12127, 44 FR 19367; and delegation of authority to the Associate Director, State and Local Programs and Support)

Issued: March 22, 1982.

Lee M. Thomas,

Associate Director, State and Local Programs and Support.

[FR Doc. 82-10932 Filed 4-20-82; 8:45 am]

BILLING CODE 6718-03-M

---

44 CFR Part 67

[Docket No. FEMA-6273]

National Flood Insurance Program; Proposed Base Flood Elevation Determinations for the Village of Arlington, Washington County, Nebraska

AGENCY: Federal Emergency Management Agency.

ACTION: Proposed rule.

SUMMARY: Technical information or comments are solicited on the proposed base flood elevations as described below.

The proposed base flood elevations are the basis for the flood plain management measures that the community is required to either adopt or show evidence of being already in effect in order to qualify or remain qualified for participation in the National Flood Insurance Program (NFIP).

DATES: The period for comment will be ninety (90) days following the second publication of this proposed rule in the newspaper of local circulation in the above-named community.

ADDRESSES: Maps and other information showing the detailed outlines of the flood-prone areas and the proposed base flood elevations are available for review at the Office of the City Clerk, 245 North Second Street, Arlington, Nebraska.

Send comments to: Mr. Donald Brown, Chairman, Village of Arlington, P.O. Box 39, Arlington, Nebraska 68002.

FOR FURTHER INFORMATION CONTACT: Mr. Robert G. Chappell, P.E., Chief, Engineering Branch, Natural Hazards Division, Office of Natural and Technological Hazards, Federal Emergency Management Agency, Washington, D.C. 20472, (202) 287-0216.

SUPPLEMENTARY INFORMATION: The Associate Director, State and Local Programs and Support, gives notice of the proposed base flood elevations for the Village of Arlington, Nebraska in accordance with Section 110 of the Flood Disaster Protection Act of 1973 (Pub. L. 93-234), 87 Stat. 980, which added section 1363 to the National Flood Insurance Act of 1968 (Title XIII of the Housing and Urban Development Act of 1968, Pub. L. 90-448), 42 U.S.C. 4001-4128, and 44 CFR Part 67.

These base flood elevations, together with the flood plain management measures required by § 60.3 of the program regulations, are the minimum that are required. It should not be construed to mean the community must change any existing ordinances that are more stringent in their flood plain management requirements. The community may at any time enact stricter requirements on its own, or pursuant to policies established by other Federal, State, or regional entities. The proposed base flood elevations will also be used to calculate the appropriate flood insurance premium rates for new buildings and their contents and for the second layer of insurance on existing buildings and their contents.

List of Subjects in 44 CFR Part 67

Flood insurance, Floodplains.

The proposed base flood elevations are as follows:

Source of flooding and location	Elevation (feet) national geodetic vertical datum	Zone designation
Elkhorn River:		
Just downstream of the Chicago and Northwestern Railroad.	1,161	A6.
Just upstream of the Chicago and Northwestern Railroad..	1,164	A6.
At U.S. Route 30..........................	1,165	A6.
Approximately 1,400 feet upstream of U.S. Route 30.	1,166	A6.

Pursuant to the provisions of 5 U.S.C. 605(b), the Associate Director, State and Local Programs and Support, to whom authority has been delegated by the Director, Federal Emergency Management Agency, hereby certifies

that this rule if promulgated will not have a significant economic impact on a substantial number of small entities. This rule provides routine legal notice of technical amendments made to designated special flood hazard areas on the basis of updated information and imposes no new requirements or regulations on participating communities.

(National Flood Insurance Act of 1968 (Title XIII of Housing and Urban Development Act of 1968), effective January 28, 1969 (33 FR 17804, November 28, 1968), as amended; 42 U.S.C. 4001-4128; Executive Order 12127, 44 FR 19367; and delegation of authority to the Associate Director, State and Local Programs and Support)

Issued: March 24, 1982.

Lee M. Thomas,

Associate Director, State and Local Programs and Support.

[FR Doc. 82-10835 Filed 4-20-82; 8:45 am]

BILLING CODE 6718-03-M

FEDERAL COMMUNICATIONS COMMISSION

47 CFR Ch. I

[CC Docket No. 81-893; FCC 81-576]

Procedures for Implementing the Detariffing of Customer Premises Equipment and Enhanced Services (Second Computer Inquiry)

AGENCY: Federal Communications Commission.

ACTION: Notice of inquiry.

SUMMARY: In order to implement its Computer II decision to deregulate carrier provided customer premises equipment, the Commission must develop procedures for valuing and detariffing embedded customer premises equipment. Changes in the carriers' accounting procedures must also be made. This Notice of Inquiry explains the tasks which need to be done to implement the Computer II decisions and requests proposals from interested parties on how these tasks can be accomplished.

DATES: Comments must be received on or before June 4, 1982, and Reply Comments must be received on or before July 9, 1982.

ADDRESS: Secretary, Room 222, Federal Communications Commission, Washington, D.C. 20554.

FOR FURTHER INFORMATION CONTACT: William S. Reece, (202) 632-4715.

SUPPLEMENTARY INFORMATION:

Adopted: December 17, 1981.

Released: April 13, 1982.

In the matter of procedures for implementing the detariffing of customer premises equipment and enhanced services (Second Computer Inquiry); notice of inquiry.

I. Introduction

1. In the Second Computer Inquiry or Computer II, Final Decision, 77 FCC 2d 384 (May 1980), Reconsideration Order, 84 FCC 2d 50 (December 1980), Further Reconsideration Order, 88 FCC 2d 512 (October 1981), the Commission determined that "enhanced services" [1] and the provision of customer premises equipment (CPE) [2] were not communications common carriage and should be offered separate and apart from a carrier's tariffed services. Customer premises equipment, however, has traditionally been provided by telephone companies, under tariff, as part of regular utility service. To accomplish the separation between utility and non-utility services required under *Computer II*, AT&T will be permitted to offer enhanced services only through a separate corporate entity which is not engaged in common carriage. Other carriers will be permitted to offer non-utility services so long as they are segregated from tariffed services by appropriate accounting procedures.

2. In order to achieve the dichotomy envisioned by *Computer II*, it will be necessary for carriers to terminate existing CPE offerings, to detariff CPE and to remove the costs associated with CPE from utility revenue requirements. On Further Reconsideration, the Commission affirmed that the detariffing of CPE was to be accomplished through a bifurcated transition plan which distinguished between "new" and "embedded" CPE.[3] The plan requires that only new CPE be detariffed on or before January 1, 1983; embedded CPE is to be detariffed after the completion of a separate "implementation proceeding" which "will address issues of capital recovery and asset valuation, alternative mechanisms by which transition to an unregulated CPE environment may be achieved, and the appropriate time period for removal of embedded CPE investment from separations and a carrier's rate base." Reconsideration, para. 55.

3. In our *Computer II* orders we have erected a framework for detariffing CPE. The problem at this point is no longer where we are going but how to get there. We are keenly aware of the difficulties presented by the tasks before us. There is, for example, the need to remove—or at least consider the means for removing—over $20 billion in CPE and other assets currently in the rate bases of regulated carriers. As we noted on Further Reconsideration, our efforts to detariff must be regarded in large measure as an "organic process" which "will continue to evolve as we gain experience." Further Reconsideration, para. 107. We intend to move carefully, with due caution, and to seek the advice of state regulators and other concerned parties where practicable. To this end, we intend, from time-to-time, as appropriate, to issue notices requesting their comments.

4. Several steps which bear upon and are important to implementation have already been taken. First, on February 24, 1982, we adopted a plan recommended by a Joint Board convened in Docket 80-286 (the so-called "Popenoe Plan") for phasing CPE out of the jurisdictional separations process. Amendment of Part 67 of the Commission's Rules and Establishment of a Joint Board, FCC 82-98. This phase out will occur over a period of five years and will begin concurrently with the effective date of *Computer II*, January 1, 1983. The phase-out approach adopted by the Commission should substantially alleviate concerns that detariffing of CPE will result in a sudden or harmful shift of revenue requirements to the various state jurisdictions.

5. Equally important, on January 21, 1982, we prescribed remaining life depreciation rates for terminal equipment held by carriers subject to our depreciation prescription process.[4]

---

[1] "Enhanced services" are defined in *Computer II* to include all services offered over common carrier transmission facilities used in interstate communications, which employ computer processing applications that act on the format, content, code, protocol or similar aspects of the subscriber's transmitted information; provide the subscriber additional, different, or restructured information; or involve subscriber interaction with stored information. Reconsideration Order, Appendix, p. 1 (Rule 64.702(a)).

[2] Customer-premises equipment includes all equipment provided by common carriers and located on customer premises except overvoltage protection equipment, inside wiring, coin-operated or pay telephones, and multiplexing equipment used to deliver multiple channels to the customer. Reconsideration Order, para 34, n. 10.

[3] Upon Further Reconsideration, however, the Commission modified the definitions of "new" and "embedded" CPE. Under the bifurcation plan adopted, embedded CPE is defined as "that equipment or inventory which is tariffed or otherwise subject to the jurisdictional separations process as of the bifurcation date (January 1, 1983)." New CPE is defined as "[a]ny other CPE which is acquired by a carrier or manufactured by an affiliated entity after (the bifurcation date)." Further Reconsideration, para. 45.

[4] See *Orders* FCC-82-39, FCC-82-40, FCC-82-41, FCC-82-42, released January 28, 1982.

The remaining life rates adopted will, we believe, reduce the amount of any underdepreciation for "embedded" CPE which may now exist and will facilitate the sale, transfer or other disposition of that CPE.

Options for CPE Detariffing

6. In our *Computer II* decisions, we made clear that the various state jurisdictions might independently take steps to remove embedded CPE from tariffed services. We noted that various states were currently examining the option of affording subscribers the ability to purchase the CPE they currently use and that California, in particular, had decided to vigorously pursue the sale of existing CPE (*Reconsideration* at paras. 31–35). We continue to believe that the sale of embedded CPE, under state auspices, to subscribers now using such equipment is an appropriate means of reducing the amount of CPE subject to tariffing and will be helpful in easing the transition.

7. In this phase of the implementation proceeding, we will consider possible procedures for removal of the remaining CPE—i.e., that portion of embedded CPE not sold under state auspices to individual subscribers—from tariffed service. As we view it, there are four possible options which may be used to detariff existing customer premises equipment:

1. The sale of CPE to the subscriber using it.
2. The transfer of CPE to the carrier's untariffed service or, in the case of AT&T, its sale to a separate subsidiary.
3. The sale of CPE to a third party.
4. Allowing the equipment to remain in tariffed service until it is fully retired.

Although these different options could conceivably be used by themselves, they might also be used in tandem. There are obviously a large number of possibilities to choose from here. Rather than speculate as to possible plans at this time we would first ask the parties to comment upon and present their views as to how the various alternatives listed above should be used—either separately or in combination—and what the appropriate time period for detariffing might be under a given option or set of options.

Valuation

8. If remaining embedded CPE is to be sold or transferred, it will be necessary to develop procedures for the valuation of such CPE. The sale or transfer of CPE would have the advantage of eliminating, or at least shortening the substantial delay that would be entailed in waiting for full retirement. In any case, it will be necessary to develop procedures for the valuation of other assets which might be needed to support CPE operations and which a carrier might wish to transfer out of tariffed service. It is our tentative view that the transfer of equipment to untariffed service or its sale to a separate subsidiary or a third party should be at a price equal to its "economic value," that is, the maximum amount the carrier would be willing to pay for that equipment if, instead of owning it, the carrier had the opportunity to buy it. We believe that this concept of economic value flows naturally from standard capital budgeting procedures which are familiar to corporate management.[5] The logic of this approach to valuation follows from the fact that a carrier would consider buying the assets in order to earn some expected flow of net revenues in the future. The discounted expected flow of net revenues to be earned from using the assets, adjusted in someway for the uncertainty of future returns, would determine the maximum amount the firm would be willing to spend to acquire those assets.

9. The capital budgeting approach we propose for defining economic value is not the only possible approach. Another possible concept is that of opportunity cost, which in this context would mean that the economic value of the assets would be defined to be the maximum amount the carrier could get for the assets by selling them to another firm rather then using them itself. We tentatively reject this approach since it seems likely that the value of the CPE to the carrier itself would greatly exceed the value to other firms, so that a transfer at opportunity cost would allow the carrier more than full capital recovery. That is, the carrier would continue to earn the allowed rate of return and recover depreciation expenses on the amount of the difference between the economic values calculated by the two approaches, and also achieve an internal rate of return on the assets greatly in excess of the appropriate discount rate. To see this, note that the capital budgeting definition of economic value is the asset price which would equate the internal rate of return and the relevant discount rate (cost of capital), so that a smaller asset price would raise the internal rate of return above the discount rate.

10. We ask parties to comment on our tentative definition of economic value and the use of the economic value standard as a basis for determining the sale or transfer prices of CPE. We invite parties to propose other valuation concepts to be used in setting sale or transfer prices for remaining embedded CPE.

11. In addition to defining economic value we must also develop a method for its measurement. The transfer of embedded CPE from tariffed to untariffed accounts is unique, and the economic value to be determined is likely to be little more than hypothetical. We do not expect that any kind of workable market exists for the sale of the large quantities of CPE which might be transferred in the transition to detariffing. It may therefore be necessary to develop some form of surrogate which can be used to measure economic value. There are a number of different valuation methods, which might be used alone or in combination. For example, we could attempt to imitate the process the firm would pursue in its capital budgeting process by estimating future net revenues to be earned from the assets and discounting these flows to the present, in some manner adjusting for the uncertainty of future returns. There are two primary difficulties we would face in trying to implement this procedure. The first is trying to estimate the future net revenues. The second is in adjusting for the uncertainty of these revenues. Of course, we expect that the carriers themselves will be performing these calculations internally; so that one possibility is to have the carriers report these results to the Commission. We could then review these studies and either accept the findings as providing measures of economic value or reject them if found to be seriously flawed.

12. Using net book value as a proxy for economic value is another alternative. It has the advantage of extreme simplicity, and it may be the most prudent approach in some cases given the practical difficulties of implementing other alternatives. These alternatives include having the assets appraised by independent appraisers or, as NTIA has suggested, conducting auctions.[6]

13. The means selected for measuring economic value is of critical importance. The amounts involved are enormous, and there could be a substantial adverse impact upon monopoly ratepayers as a result of any underpayment to the regulated entity. Thus, we seek the comments of all interested parties on the valuation question. We ask that parties address their comments both to the

[5] See, *e.g.*, the discussion of valuation in Marshall R. Colberg, Dascomb R. Forbush and Gilbert R. Whitaker, Jr., Business Economcs: Principles and Cases, p. 209, *et seq.*

[6] See, Petition for Reconsideration, filed by NTIA after the adoption of the Final Decision, at p. 16.

appropriate concept of economic value and to the practical means that might be used to measure it. We also ask that, in addition to commenting on our preliminary views expressed above, parties present detailed alternative proposals of their own for our consideration.[7]

Possible Impact of the 1982 AT&T Consent Decree

14. On January 8, 1982, the United States and the American Telephone and Telegraph Company filed in the United States District Court for the District of New Jersey an agreed upon modification to the 1956 consent decree entered in United States v. Western Electric Co., et al. (CA No. 17-49). The parties also filed a motion to transfer that case to the United States District Court for the District of Columbia. Simultaneously, the parties filed in the District Court for the District of Columbia a Stipulation for Voluntary Dismissal of the government's pending antitrust suit, United States v. American Telephone and Telegraph Company, et al. (CA No. 74-1698).

15. The proposed settlement would vacate the 1956 judgment in its entirety and substitute therefore a plan which would encompass far-reaching structural changes for Bell system. The modified Consent Decree would require, inter alia, that AT&T divest itself of its 22 majority-owned operating companies (BOCs), "by means of a spin-off of stock of the separated BOCs to the shareholders of AT&T, or by other disposition." Section I(A)(4) of the Decree. Moreover, the divested BOCs would be limited to providing exchange and exchange access and other natural monopoly services which are actually regulated by tariff; they would not be permitted to provide interexchange services, information services or customer premises equipment. Within 6 months after entry of the decree by the District Court AT&T must submit a reorganization plan for approval by the Department of Justice and is required to complete the plan within 18 months after the decree is entered.

16. The decree requires that AT&T take certain actions not required under *Computer II* but which affect its provision of embedded CPE. Because of the requirement that provision of CPE be separated from the provision of local exchange service, CPE may not be owned by the same Bell entity that provides local exchange service. This will necessitate a change in the way CPE traditionally has been offered.[8] For example, embedded CPE and associated installation and maintenance will have to be unbundled from charges associated with local exchange service and offered by a different entity separate and apart from local exchange service. For the most part, this could be accomplished through CPE tariffs filed at the state level by AT&T even though it would no longer provide local exchange service. Tariffing of embedded CPE would still remain because the decree, while it separates the provision of CPE from local exchange service, does not require the detariffing of any CPE.

17. The decree also requires AT&T to separate installation and maintenance personnel associated with the provision of embedded CPE from carrier personnel associated with the maintenance of local exchange service. Thus, after divestiture AT&T would no longer be able to continue to combine exchange service maintenance with the maintenance of embedded CPE. Under the Commission's bifurcated approach to detariffing CPE, AT&T's operations with respect to these two activities are unaffected.[9] If AT&T is to continue providing installation and maintenance services for embedded CPE, the decree forces a separation of such services at the time of divestiture which goes beyond that separation considered in *Computer II*. We conclude, therefore, that it would be appropriate for interested parties (including affected state regulatory agencies) to comment on what further regulatory action, if any, should be considered to facilitate a smooth integrated transition under *Computer II* and the decree.

18. In this regard it might prove helpful to note that the Commission has already indicated various options available to AT&T to alleviate potential operational concerns that might arise from a fragmentation of its installation and maintenance services. In our order on Further Reconsideration, we indicated that AT&T could endeavor to have a single installation and maintenance force by pursuing the deregulation of maintenance for embedded CPE, or even keeping maintenance of embedded CPE tariffed but contracting out the actual work to the separate subsidiary. See 88 FCC 2d at 532. These options may find appeal in a post-decree environment if the decree were to result in an undue fragmentation of the maintenance function. Accordingly, interested parties may comment on these matters and on the extent to which the decree requirements affecting CPE impact the various options discussed above for detariffing any remaining embedded CPE.

Accounting Problems

19. Under our *Computer II* decision various carriers may, for the first time, engage in detariffed activities without federally imposed structural separation requirements. As a result, there is increased reliance on accounting mechanisms to separate costs for tariffed and untariffed activities. Guidelines must be adopted to ensure that costs attributable to the provision of enhanced services and CPE are not reflected in accounts for tariffed services and are not borne by the communications ratepayers.

20. In addressing accounting separation there are two different categories of carriers to consider—those carriers which traditionally have been subject to this Commission's system of accounts and those carriers which have not. As to those carriers not governed by our Uniform System of Accounts (USOA), we envision that state regulatory commissions will oversee the appropriate accounting separation for those carriers subject to their jurisdiction. With respect to those carriers which are subject to the USOA certain guidelines must be established to ensure that direct and indirect costs attributable to the provision of enhanced services and CPE are not reflected in accounts for tariffed services. Even though carriers other than AT&T are not subject to the structural separation requirement, they still have to account for and allocate costs between tariffed and non-tariffed activities. Costs associated with shared personnel, services, and network facilities, for example, must be appropriately allocated so that the ratepayer is not burdened by a carrier's detariffed activities. We anticipate that separate accounts and subaccounts will have to be set up so that the cost and revenue flows inherent in any sharing can be readily tracked. We solicit proposals from carriers and other interested parties on the methodology to

[7] We recognize that in deciding upon and carrying out an appropriate plan for removal of embedded CPE from regulated service it may be necessary to employ outside consultants to assist the staff in their efforts. Parties filing comments may assume that, where required, outside help for specific valuation problems will be available to the staff.

[8] Moreover, since toll settlements based on CPE have been a source of subsidy for local rates (See FCC 82-98, at paragraph 38), the transfer of ownership of CPE from AT&T's local exchange carriers raises separations related issues.

[9] For new CPE offered after January 1, 1983, however, AT&T's subsidiary must use separate installation and maintenance personnel for residential CPE, but may share carrier personnel for installation and maintenance associated with business CPE for an 18 month transition period.

be used to allocate such costs as well as the appropriate accounting procedures.[10]

21. In our recent First Report and Order in CC Docket 79-105, 85 FCC 2d 818, 834-39 (1981), we addressed the need for changes in the Uniform System of Accounts for Class A and Class B telephone companies regarding sales of CPE. We ordered that all revenues and expenses associated with the sale of new terminal equipment be recorded in account 316, "Miscellaneous income." We also established a new account, account 124, "Merchandise and material held for sale," to record the cost of new merchandise, including new terminal equipment, held for sale or for new material for use in repair of CPE. We required that account 327, "Other non-operating taxes," be used to record applicable taxes related to account 124. *Id.* at 838. We deferred consideration of accounting issues related to the sale of embedded CPE to the instant proceeding.

22. Thus, we solicit from the affected carriers and all interested parties detailed proposals concerning the procedures that should be adopted to account for the sale of embedded CPE and related plant. In particular, separate accounts or memorandum records may have to be created to reflect either any shortfalls, if CPE or other assets are sold or transferred at less than net book value, or any gains, if assets are transferred or sold at more than net book value. The general rule is that a regulated utility is entitled to recover the full value of assets prudently invested in public utility service unless it has otherwise been compensated for the risk of loss and that consumers have the superior claim to capital gains achieved on depreciable and nondepreciable assets, Democratic Central Committee of D.C. v. Washington Metropolitan Area Transit Comm'n, 485 F. 2d 786 (D.C. Cir. 1973). We are, of course, not faced at this time with the problem of determining the treatment of any gains or losses on the sale of assets in connection with *Computer II.*[11] Our point is simply that any shortfall or any gain relative to net book value should at least be recognized in a separate account so that any refunds or adjustments to revenue requirements would be possible if this Commission, in an interstate rate case, or a state commission, in a state rate case, finds that such action is appropriate.

23. While we have already made changes in Part 31 of our Rules and Regulations to account for sale of new terminal equipment by Class A and Class B telephone companies, see paragraph 21, supra, we have not done so with regard to international record carriers or Western Union. The Uniform System of Accounts for these carriers are specified in Parts 34 and 35 of our Rules and Regulations.[12] To remedy this situation, we will in the near future release a Notice of Proposed Rulemaking to revise Parts 34 and 35.

24. Finally, the Commission will issue, within the next several months, a Notice of Proposed Rulemaking addressing specific accounting problems which should be resolved prior to the provision of new CPE on a detariffed basis. These problems include establishing separate accounts for coin telephones and for telephones, teletypewriters, and large and small private branch exchanges used for telephone company business; separating tariffed and untariffed cables and wires associated with large private branch exchanges (the demarcation problem); establishing item listings for all new CPE accounts as well as the appropriate adjustments to item listings and retirement units for existing accounts; and, reclassifying of certain miscellaneous equipment.

Conclusion

25. Many complex issues must be resolved before our decisions in *Computer II* can be fully implemented. In resolving these questions we will, as already noted, issue a series of Notices requesting the participation of interested parties. We encourage all interested parties to submit their views, in detail, on the issues raised in this Notice. Comments should be filed on or before June 4, 1982. Reply comments will be due on or before July 9, 1982.

26. Accordingly, it is ordered, pursuant to Section 4(i), 4(j), 201-205, 403 and 404 of the Communications Act of 1934, as amended, that an inquiry into the matters described herein be instituted.[13]

27. it is further ordered, That the Secretary shall cause a copy of this Notice of Inquiry to be published in the Federal Register.

Federal Communications Commission.

William J. Tricarico,

Secretary.

[FR Doc. 82-10564 Filed 4-20-82; 8:45 am]

BILLING CODE 6712-01-M

[10] We required AT&T to submit within seventy-five days of the release of our order on Further Reconsideration a detailed description of any administrative services which it will share with its subsidiary, along with an explanation as to how the costs of any shared services are to allocated between the two entities. Id. at para. 104. AT&T filed its response to this requirement on January 13, 1982. (Report on Services To Be Shared Between Fully Separated Subsidiary and Affiliated Companies, and Associated Costing Methodology). On February 1, the Common Carrier Bureau issued a Public Notice requesting parties to file comments on AT&T's report. Comments were received on February 19, 1982 and AT&T replied on March 11, 1982. The development of appropriate accounting mechanisms to reflect the cost of shared services, personnel, etc. between AT&T's subsidiary and affiliated entities has proceeded independently of this proceeding. Any inconsistencies between the separate accounting mechanisms adopted for AT&T and the accounting changes considered herein will be reconciled at the conclusion of this proceeding.

[11] See, however, our Phase II Final Decision and Order in Docket 19129, 64 FCC 2d 1 (1977), at paras. 170-175.

[12] Currently there are no Class C telephone companies subject to our jurisdiction. Thus, we are not at this time addressing Part 33, which specifies the Uniform System of Accounts for Class C telephone companies.

[13] This Notice of Inquiry reflects events subsequent to the adoption date of December 17, 1981. These changes have been approved by vote of the Commission on circulation. Section 0.5(d) of our Rules, 47 CFR 0.5(f)(d).

DEPARTMENT OF COMMERCE

National Oceanic and Atmospheric Administration

50 CFR Part 285

Atlantic Tuna Fisheries

AGENCY: National Oceanic and Atmospheric Administration (NOAA), Commerce.

ACTION: Proposed rule.

SUMMARY: NOAA proposes to implement the recommendations of the International Commission for the Conservation of Atlantic Tunas (ICCAT or the Commission) to limit fishing for Atlantic bluefin tuna for two years in the western Atlantic Ocean to a small catch needed to monitor the status of the stock. Implementation of this recommendation will reduce substantially the annual quota for U.S. fishermen. Changes in existing regulations are proposed to provide a reasonable mechanism for managing the fishery consistent with the ICCAT recommendations and the significantly reduced quota.

DATES: Comments must be received on or before May 21, 1982.

See supplementary information section for dates of the public hearings on this proposed rulemaking.

ADDRESSES: Send comments on this proposed rulemaking to: National Marine Fisheries Service, Northeast Region, Management Division, State Fish Pier, Gloucester, Massachusetts 01930-3097. Clearly mark "Comments on bluefin tuna regulations" on the outside of the envelope. Copies of the draft Environmental Impact Statement, Regulatory Impact Review, and

Regulatory Flexibility Analysis are available from: National Marine Fisheries Service, Northeast Region, Services Division, P.O. Box 1109, Gloucester, Massachusetts 01930.

See "Supplementary Information" for addresses of public hearings on this proposed rulemaking.

FOR FURTHER INFORMATION CONTACT: Mr. William C. Jerome, Jr., 617-281-3600, extension 325 or David S. Crestin, 617-281-3600, extension 253.

SUPPLEMENTARY INFORMATION:

Background

The United States is a signatory nation to the International Convention for the Conservation of Atlantic Tunas (the Convention, 20 UST 2887, TIAS 6767). The Convention's provisions entered into force for the United States on March 21, 1969. The United States' obligation under the Convention are implemented by the Atlantic Tunas Convention of Act of 1975 (16 U.S.C. 971-971h) (the Act). Both the Convention and the Act are directed towards "maintaining the populations of Atlantic bluefin tuna at levels which will permit the maximum sustainable catch of food and other purposes" (Preamble to the Convention). This Act directs the Secretary of Commerce to promulgate regulations necessary to implement recommendations adopted by ICCAT and to carry out the purposes and objectives of the Convention.

The Secretary, through the National Marine Fisheries Service (NMFS) of the NOAA, has continually monitored the stock levels of Atlantic bluefin tuna to fulfill the international treaty obligations of the United States.

Regulations presently governing the U.S. fishery (45 FR 40118, 45 FR 48636, 46 FR 8012) were designed to implement the following recommendations of ICCAT made in 1974: (1) To prohibit any taking and landing of Atlantic bluefin tuna weighing less than 6.4 kilograms (kg) (14 pounds (lbs)) except for a 15 percent incidental catch allowance; and (2) to limit fishing mortality to recent levels. Until last fall, these were the only recommendations which ICCAT had made pertinent to Atlantic bluefin tuna.

At its Seventh Regular Meeting in Tenerife, Canary Islands, November 11-17, 1981, the Commission adopted a Standing Committee on Research and Statistics (SCRS) recommendation that harvest levels of Atlantic bluefin tuna in the western Atlantic Ocean be as near zero as feasible for two years, since information indicates this stock is depleted. The Commission recommended that the nations actively fishing for bluefin tuna in the western Atlantic Ocean (United States, Canada, and Japan and to a lesser extent Brazil and Cuba) consult and conclude consultations before February 15, 1982, to develop conditions under which fishing by their nationals would be carried out. The Commission further recommended, until such conditions were developed, that direct and incidental catches be limited to an annual level of 800 metric tons (mt) (882 short tons (st)) to enable ongoing scientifc studies to continue.

The required consultations were held on February 8-12, 1982, in Miami, Florida, among officials representing the governments of Brazil, Canada, Japan, and the United States. Officials of the participating governments shared their concerns on the status of the Atlantic bluefin tuna stock and the need to ensure a stablization or increase in stock size. During the Miami consultations, the status of the Atlantic bluefin tuna stock was re-examined and, with additional input of the latest Japanese catch data, estimates of surplus production were revised slightly upward. Consideration was given to the annual catch needed to allow ongoing scientific studies to continue to monitor the abundance of the stock. Preliminary estimates of economic impacts of the different Atlantic bluefin tuna fisheries also were reviewed. Officials agreed to recommend to their governments the following measures for 1982 and 1983 to implement the ICCAT recommendations on Atlantic bluefin tuna management in the western Atlantic Ocean:

(1) The annual catch be limited to 1,160 mt (1,279 st);

(2) The annual catch be allocated as follows:

Canada	250 mt (276 st)
Japan	305 mt (336 st)
United States	605 mt (667 st)

(3) The catch from the developing tuna fisheries of Brazil and Cuba, which currently take less than 50 mt (55 st) of Atlantic bluefin tuna annually, not be included in the total quota of 1,160 mt (1,279 st).

(4) There be no directed fishery on the spawning stock of Atlantic bluefin tuna in the Gulf of Mexico.

Officials also agreed to recommend to their governments that they immediately initiate steps necessary to implement the management measures agreed upon in these consultations. These proposed regulations implement the ICCAT recommendations from the November 1981 Tenerife meeting and the recommended management measures from the February 1982 consultative process.

The recent action taken by ICCAT to prohibit the capture of Atlantic bluefin tuna in the western Atlantic Ocean has been the result of monitoring this fishery over a long period of time. Measures taken in 1974 were designed to arrest the decline in the abundance of the stocks and, with favorable recruitment in subsequent years, ICCAT hoped that rebuilding of the stocks would occur. The annual assessments have shown instead a continuing long-term decline which led to the ICCAT recommendation during its November 1981 meeting.

Concern for the conservation of Atlantic bluefin tuna surfaced in the early 1970's when reports of the SCRS highlighted two disturbing trends regarding the stock of Atlantic bluefin tuna: The very sharp decline in catches of large bluefin tuna and the substantial catches of very small bluefin tuna. In subsequent years, the SCRS noted the decreasing number of giant fish, the continuing absence of medium-size fish, and the increasing average size of the giants; these trends indicate low recruitment to the stock. As noted above, in 1974, the Commission responded to these reports and adopted a minumum size regulation of 6.4 kg (14 lbs) and a limit on fishing mortality for bluefin tuna which was intended to prevent a further increase in catches.

Since 1974, the SCRS reports on the status of bluefin tuna stocks have revealed troublesome trends. In 1976, the SCRS pointed out that the levels of fishing mortality were high for the long-lived bluefin tuna, with fishing mortality on the small and medium fish affecting recruitment to the large size group. The following year, the SCRS noted that the spawning stock size was small and that further reductions would soon bring the spawning stock to a level at which recruitment to the stock would be affected. The SCRS also indicated, however, that while the abundance of giant Atlantic bluefin tuna in the western Atlantic Ocean continued to decrease, more fish were reaching 4 or 5 years of age and that these fish could be expected to make a significant contribution to the spawning stock if their exploitation could be held to reasonable levels.

In 1978, the SCRS, in its appraisal of the western Atlantic bluefin tuna stock, found that although the minimum size regulations and mortality limitations implemented by ICCAT appeared to have reduced mortality on young Atlantic bluefin tuna and thus should increase escapement out of the surface fishery and into the spawning group, the condition of the stock of bluefin tuna

age 6 and above did not appear to have improved. The average size for giant fish in the western north Atlantic Ocean continued to increase in 1978 in all fisheries except the Japanese longline fishery in the Gulf of Mexico. The SCRS in 1978 repeated its concerns of 1977 that unless controls were placed on the harvest of immature bluefin tuna, expected increases in the spawning stocks might not materialize. The SCRS warned that if controls are not placed on catches of young mature fish, then spawning stock size might decrease, and recruitment failure might then occur.

In 1980, the SCRS continued to note that the stock structure of bluefin tuna remained uncertain although evidence at the time was towards the hypothesis of separate eastern and western Atlantic stocks, with only a small and variable interchange between these two stocks. An analysis of the bluefin tuna stock under the single stock assumption showed that if the current catch level, either in weight or numbers, was maintained, the adult stock (age 6 and over) could decline considerably by 1985; however, if the current fishing mortality rate was maintained the decline would not be as severe. Analysis of the bluefin tuna stocks in the western Atlantic Ocean under the assumption of two separate stocks indicated that adult stock abundance in the western Atlantic decreased 75 percent between the early 1960's and 1978, then increased with recruitment of the abundant 1973 year-class to the adult stock in 1979. Juvenile stock size decreased 75 percent from 1960 to 1973. The strong 1973 year-class accounted for higher levels of juvenile abundance from 1974 until 1979. Recruitment levels since 1977 have been below average.

The SCRS reports, described above, have, over the years, been of concern to the United States. In 1977, the United States attempted unsuccessfully to improve the measures concerning bluefin tuna adopted by the Commission in 1975 by proposing that member governments actively fishing for bluefin tuna limit the fishing mortality of bluefin tuna in each of the areas of their fishing operations to the average mortality occurring in these areas during 1970–1974. In subsequent years, the United States continued to express its concern over the status of the bluefin tuna stocks.

At the 1981 meeting of ICCAT, one of the SCRS proposed management regimes was based on the assumption of separate stocks. Under this assumption, the SCRS analysis indicated a significant difference in the abundance estimates of the two stocks. While stock abundance in the eastern Atlantic appears to be stabilizing or even slightly increasing, current levels of juvenile and large fish in the Western Atlantic are very low. Recruitment levels have dropped to 12 percent of the 1960 level. Stock-recruitment analysis indicates that recruitment abundances will continue to be very low. The 1973 year-class has been reduced greatly by heavy exploitation and cannot be expected to contribute significantly to the western Atlantic spawning stock. Since there is no indication that spawning stock abundance will increase in the immediate future, probability of an abundant year-class in the near future appears very low. At current abundance levels, there will be little, if any, 1982 surplus production of adult fish.

Proposed changes to regulations

1. Change in Nature of U.S. Fishery

ICCAT recommended that the individual country allocations of the recommended total quota should be used to the maximum extent practicable to allow collection of scientific information necessary for assessing the status of the Atlantic bluefin tuna stock and to enable ongoing scientific studies to continue. This requires the fisheries of the involved nations, including the U.S., to undergo significant changes. The table below shows the quotas that have been in effect since the 1980 fishing season for various segments of the U.S. fishery.

1980–81 [1]

	Short tons	Metric tons
Total quota	2,393	(2,171)
1. Purse seine	950	(862)
a. School tuna	650	(590)
b. Giant tuna	300	(272)
2. Handgear fishery	1,218	(1,105)
a. Northern area	1,128	(1,023)
i. General category	876	(795)
ii. Charter boat	102	(92)
ii. Harpoon boat	150	(136)
b. Southern area	90	(82)
3. Research	225	(104)

[1] Present regulations specify quotas in short tons. Conversions to metric tons are approximations.

These proposed regulations would eliminate specific allocations for several sectors of the fishery; vessels in these sectors would be fishing under different or more general quotas. The reasons for this are: (1) The small quantity of Atlantic bluefin tuna available does not make it feasible or practicable to continue such an elaborate allocation scheme; (2) stock assessment information is needed; and (3) the unavoidable catch of Atlantic bluefin tuna taken incidentally to fishery operations directed at other species is part of the quota.

The first consideration for a monitoring program involves the availability of timely catch and fishing effort information. It is important that the catch information broadly represent each age group in the stock. The bluefin tuna stock assessment calculations require that recent catch and effort data be available for each age/size group represented in the fishery. The monitoring scheme also should give priority to those sectors of the fishery which provide timely fishing effort information that is related to the fishing mortality rate.

Second, the catch must be available for taking specimens for conducting stock structure research. As broad a sample as possible over the age groups is required.

Third, some allowance for those fisheries which take Atlantic bluefin tuna incidentally to the harvest of other fish species must be considered.

To summarize, the following five criteria were used to develop measures for the U.S. fisheries consistent with the ICCAT recommendation: (1) Minimal catch sufficient for scientific purposes; (2) catch should broadly cover as many age groups as possible; (3) fishing effort that generates the catch should be related to the fishing mortality rate; (4) the catch must be available for scientific sampling; and (5) minimal bycatch should be allowed. The quotas proposed are as follows:

1982–83 (PROPOSED) [1]

	Short tons	Metric tons
Total quota	667	(605)
1. Directed fisheries:		
a. Handgear fishery	353	(320)
b. Recreational angling	99	(90)
2. Incidental fisheries:		
a. Swordfish longline	44	(40)
b. Purse seine vessels	171	(155)

[1] Proposed regulations specify quotas in short tons. Conversions to metric tons are approximations.

2. Elimination of the Directed Purse-Seine Fishery

The existing regulations provide for a total quota of 950 st of Atlantic bluefin tuna for a directed purse-seine fishery subdivided into 650 st for school sized fish and 300 st for giants. The purse-seine fishery for school fish takes place off the northeastern coast of the United States. This fishery operates within a narrow belt that extends about 140 miles (225 km) from the coastline primarily between Cape Charles, Virginia and Cape Cod, Massachusetts. These fish range primarily between 20 and 48 inches (51 and 122 cm) fork length and between two and five years of age. In 1981, five vessels successfully

participated in this fishery. Catches in 1981 amounted to 728 st of school tuna and 157 st of giant tuna. Average catches for the five year period 1977–1981 were 944 st of school fish and 201 st of giant tuna.

In general, effort information on purse-seine fishing is not suited for indexing the fishing mortality rate in small, localized fisheries. This U.S. purse-seine fishery is no exception in that only a relatively few vessels are active in the fishery. In addition, if the catch were restricted to 100–200 st, then it becomes more likely that the entire amount could be taken at one point in space and time. Thus, the catches likely could consist of only a few schools comprising one or two age groups. It is unlikely, therefore, that purse-seine catch data will represent the range of ages of small and medium bluefin; thus, the effort as an index of the fishing mortality rate will not be sufficiently useful for developing stock assessments for bluefin of these ages.

In some years, the domestic purse-seine vessels which have been involved actively in the Atlantic bluefin tuna fishery also direct effort at skipjack and yellowfin tunas and may catch substantial amounts of these species. The vessels direct fishing effort at these tropical tuna species in years when they are available. This occurred in 1963 and 1964 in which 33 and 49 percent of the catch was skipjack tuna, respectively. Similar circumstances occurred in 1981, when skipjack tuna catches were relatively high and bluefin tuna catches were relatively low.

Information obtained from the 1981 directed skipjack tuna fishery indicates that bluefin tuna are taken as an unavoidable incidental catch. Percentages of bluefin tuna in some opf the skipjack tuna catches ranged from approximately 8 to 17 percent. Also, catches of bluefin tuna may occur from errors in identification of the species of tuna in a school before making a purse-seine set. To address the incidental catch in this fishery, the NMFS proposes an allowable incidental catch of bluefin tuna not to exceed 10 percent by weight of all other tuna species onboard a vessel at the end of a trip. Purse-seine vessels participating in the herring, mackerel, and menhaden fisheries will continue to be allowed an incidental catch of Atlantic bluefin tuna not to exceed two percent by weight of all other fish onboard the vessel at the end of a trip. Further, the catch under these incidental catch allowances would be limited to a quota of 171 st of bluefin tuna by purse-seine vessels regardless of the directed fishery. These measures are deemed necessary to prevent catches from exceeding the total quota of 667 st available for all segments of the U.S. fishery. If the 1982 catch of skipjack tuna equals or exceeds the 1981 catch of 2,400 st, the incidential catch quota of 171 st may not be sufficient. The data base, however, for these tropical tunas over the past two decades shows that their appearance in the fishing area described above is intermittent.

3. Swordfish Longline Incidental Catch Quota

A U.S. longline fishery for swordfish has existed in the Gulf of Mexico since the 1960's with minimal incidental catch of bluefin tuna. In recent years, however, there has been a gear change (to wire leaders or heavier monofilament leaders) and the incidental catch of bluefin tuna has increased substantially. The maximum annual incidental catch of bluefin tuna, before the gear change in 1974, was estimated at approximately two to three short tons. During the 1980 winter-spring fishery in the Gulf of Mexico, U.S. longline vessels fishing for swordfish began to land increasing quantities of giant Atlantic bluefin tuna. A swordfish longline fishery also has occurred for some time in the Atlantic Ocean. Here, too, the incidental catch of giant Atlantic bluefin tuna increased but not nearly as rapidly as in the Gulf of Mexico longline fishery. These events signaled the potential development of a new directed fishery for Atlantic bluefin tuna, which would be contrary to our ICCAT commitments. To prevent this from happening, the NMFS promulgated incidental catch provisions in January 1981 to apply to all vessels using longline gear (45 FR 8102)

The NMFS permit application files show 173 longline vessels had incidental catch permits for Atlantic bluefin tuna in 1981. Prior to the 1981 regulatory change which prohibited longline vessels from conducting a directed fishery, approximately 350 vessels reported using longline gear for harvesting bluefin tuna. This suggests approximately 175 longline vessels may not have the proper permit. Operators of these vessels have been notified of the potential need to reapply for a proper permit depending on the nature of their current fishing operations.

Under these incidental catch restrictions, the longline fishery in 1981 reportedly took approximately 90 st of giant Atlantic bluefin tuna, 80 st in the Gulf of Mexico and 10 st in the Atlantic Ocean. The NMFS believes the 80 st harvested in the Gulf of Mexico in 1981 is more than the unavoidable incidental catch needs of the swordfish fishery. Data from the 1982 swordfish fishery in the Gulf of Mexico show that the landings to date are approximately 30 st with a significant portion of the season completed. Considering the traditional incidental catch of between two to three short tons and the 1982 incidental catch of approximately 30 st, the NMFS believes that the proposed quota of 44 st will address adequately the needs of the present swordfish longline fishery throughout the regulatory area and will provide adequately for the unavoidable incidental catch of giant Atlantic bluefin tuna in this fishery.

The NMFS recognizes, however, that if the Atlantic and Gulf of Mexico longline fisheries for swordfish expand, the incidental catch quota of 44 st may not be sufficient for unavoidable incidental catch of bluefin tuna in this fishery. It is important to prevent the incidental catch from exceeding this amount as the bluefin tuna taken with longline gear in the Gulf of Mexico are spawning adults. Minimizing their capture, therefore, may contribute to increasing stock size. The information which could be gained from this fishery may not be particularly useful for stock assessment purposes and may not meet the five monitoring criteria mentioned above. In addition, the ICCAT recommendation specifically directs that there be no directed fishery on spawning bluefin tuna stocks in the Gulf of Mexico.

4. The Recreational Fishery Quota

The proposed regulations provide for a recreational fishery quota for young school, school, and medium bluefin tuna of 99 st. In the past, this sector of the fishery had no specific quota. As a result of the ICCAT recommendation that the catches from all directed and incidental Atlantic bluefin tuna fisheries be counted, quotas are proposed for all gear segments of the U.S. fishery.

The U.S. recreational fishery for young school, school, and medium tuna occurs from June through October from the mid-Atlantic states to Cape Cod. Sampled length frequencies from the fishery show a wide range of age groups in the catch. Ages 1 through 5 constitute the majority of the catch but samples frequently show ages 6–10 and some age 0 fish also are taken. This range of ages encompasses all of the small and medium age groups, which makes the recreational fishery unique in this respect. Additionally, an annual survey of catch and effort data has been conducted since 1976. This combination of age distribution sampling, the availability of catch and effort data, and the length of the fishing season and area

covered makes the recreational fishery well suited for monitoring of all age groups of young school, school, and medium tuna. Stock assessment scientists recommend this fishery be used as the primary monitoring mechanism. The proposed quota of 99 st will not unduly restrict this fishery as the 1981 catch was 93 st and the five-year average was 105 st.

5. Buy Boats

The proposed regulations eliminate the use of buy boats in the Atlantic bluefin tuna fishery. Existing regulations (§§ 285.22(m) and 285.23(b)) provide for the buy boat system primarily because of the multiple daily catch limit provisions presently in effect. Some fishermen found it convenient to sell or transfer their fish at sea to enable them to continue fishing. The buy boats provided a service which included assistance in transporting multiple catches and also aided in the proper handling of the fish to protect product quality.

The enforceability of the regulations on buy boats has been controversial since the first year they were allowed to operate in the fishery. High value giant tuna provide incentive for potential violators and monitoring tuna transfers at sea is much more difficult than monitoring such activities in port.

The NMFS believes that the proposed elimination of the existing, multiple daily catch limits and the increased importance of improving the NMFS ability to monitor the fishery, make the present regulations allowing transfer of giant Atlantic bluefin tuna at sea contrary to the best interests of the fishery.

6. Change in Commencement Date of Handgear Fishery

The proposed regulations change the commencement date for the giant bluefin tuna handgear fishery (handline, harpoon, and rod and reel) from January 1 to July 15 of each year. This proposed change would eliminate the directed handgear fishery before July 15 in the regulatory area, including the Gulf of Mexico and adjacent areas. Giant bluefin spawn in the Gulf of Mexico during the winter and spring months. The January 1 commencement date, in conjunction with the present allowable catch limit of five giant tuna per vessel per week in the southern area (§ 285.32(c)), provides the opportunity for a sizeable directed handgear fishery to develop. The NMFS believes that this proposed change is necessary to prevent this from occurring on spawning Atlantic bluefin tuna in the Gulf of Mexico. At present low stock levels, such a fishery could have deleterious effects on an already declining stock. This proposed change complies with the recommendation in the February 1982 resolution developed in Miami to prohibit directed fishing on spawning bluefin tuna in the Gulf of Mexico.

A relatively small traditional rod and reel fishery has existed in the Gulf of Mexico for many years. The reported catch from this fishery was one giant in 1981; none were reported taken in 1980. NMFS believes this fishery should continue only on a tag and release basis.

The proposed change in the commencement date from January 1 to July 15 reduces the length of the fishing season for giants in the area off the mid-Atlantic and New England states. Normally, the fishery in these areas begins in early June. This proposal, in conjunction with the proposed change in the allowable catch limit, will aid in preventing an early closure of the handgear fishery.

The proposed regulations restrict the handgear fishery throughout the regulatory area. Anglers may continue to engage in the traditional tag and release program under § 285.33. Tag and release fishing enables the NMFS to obtain at little cost information on Atlantic bluefin tuna which otherwise might be lost.

7. Establishing a Single Catch Rate for Harvesting Giant Atlantic Bluefin Tuna

The existing regulations in §285.32 contain a complex set of catch limits for the giant bluefin tuna fisheries in the northern and southern management areas. The northern area fishery has a variable catch limit of from one to four giant tuna per vessel, per day, depending on the percentage of the quota harvested as the season progresses. The southern area fishery has a set catch limit of five giant tuna per vessel, per week. The NMFS established these differing catch limits for the handgear fisheries in the northern and southern areas to provide for a more equitable distribution of available fish to the fishermen, and to extend the fishing seasons as long as possible. Such a system provided the greatest number of user-days, which in turn generated greater regional coastal community income and recreational opportunties.

Allowable catch limits of this magnitude were possible because of the size of the quotas available for the northern and southern areas: 1,128 st and 90 st, respectively. Under the proposed handgear quota of 353 st of giants deemed appropriate for monitoring the status of the stock, reducing allowable catch limits may help avoid an early closure of the fishery.

The NMFS proposes a single allowable catch limit for the entire regulatory area of one giant bluefin tuna per vessel per week to apply to the handgear fishery. Based on available catch data for 1979–1981, this catch limit will provide for the longest season with a reasonable chance of achieving the quota of 353 st. Depending on average weight, this will allow the harvesting of approximately 900–1,000 giant bluefin tuna during the season.

If a uniform catch limit for the entire regulatory area for the handgear fishery is established, a major reason for separate northern and southern management areas no longer exists. The handgear fishery operating in the southern area reportedly harvested 101.3 st of giant Atlantic bluefin tuna, or approximately 113 percent of its 1981 quota of 90 st. The handgear fishery in the northern area harvested approximately 46 percent (512.5 st) of its 1981 quota. The 1981 harvest exceeds the 353 st available for the total handgear fishery under these proposed regulations. Reductions for each management area proportionate to the 1981 allocation of the total quota would result in an extremely small quota for the southern area (26 st).

The NMFS proposes to eliminate the distinction of separate northern and southern areas for management purposes for vessels permitted in the general category. However, the NMFS proposes to reserve 26 st of the total handgear quota of 353 st for the period following September 1 in the area south and west of Gay Head, Martha's Vineyard, to take into consideration the normally late fishery in this area.

8. Elimination of Separate Specific Quotas for Harpoon Boats and Charter Boats Within the General Category

The existing regulations (§285.30) contain within the general category quota of 1128 st for vessels fishing in the northern area a harpoon boat quota of 150 st, and a charter boat quota of 102 st; the charter boat quota is further subdivided into monthly sub-quotas. Under §285.32(b)(2), vessels registered in the charter boat category have an allowable catch limit of one giant bluefin tuna per vessel, per day. Sixteen vessels were registered in the charter boat category in 1980 and again in 1981. Reported catches for these vessels amounted to 11 giants in 1980 and 8 giants in 1981, against a quota equal to approximately 280 giants annually.

Applying the percentage of the general category quota allocated to

charter boats in the present regulations (8.4 percent) to the proposed handgear fishery quota of 353 st, the charter boat quota would be approximately 30 st. Using the average weight of giant fish harvested by charter boats in 1981, a quota of 30 st would amount to about 108 giants. The NMFS proposes to eliminate the separate quota for charter boats. The extremely low harvests in 1980 and 1981 do not warrant continuation of the separate quota for this category.

Section 285.30 of the existing regulations contains a quota of 150 st for the harpoon boat catgegory within the general category quota for the northern area. Harpoon boats in this category are allowed an unlimited daily catch limit of giant Atlantic bluefin tuna under existing §285.32(b)(3). In 1980 and 1981, 38 vessels were registered in the harpoon boat category. These regulations recognized a unique aspect of harpoon fishing versus other sectors of the handgear fishery. Unlike rod and reel and handline fishermen which can fish under a variety of weather conditions, harpoon fishermen require weather conditions conductive to spotting giant bluefin tuna at or near the water's surface to fish successfully. The existing regulations allowed harpoon fishermen to take maximum advantage of those days with relatively calm ocean surfaces to harvest giant bluefin tuna.

Fishing with harpoons is one of the traditional bluefin tuna fisheries off the New England coast. This fishery, however, does not provide the same potential for stock monitoring purposes as other handgear fishing methods. While the NMFS will utilize any catch and effort data generated by the harpoon fishery, it is not considered one of the primary fisheries for stock monitoring purposes.

The existing quota for the harpoon boat category of 150 st is 12.3 percent of the total general category quota of 1,218 st. Applying this same percentage to the proposed handgear quota of 353 st, the harpoon boat quota would be approximately 43 st. Based on the number of vessels registered in this category (38), and the average weight of the giant tuna harvested in 1981 (841.3), a 43 st quota would provide an average of only 2.7 giant bluefin tuna per boat, per season. Such a small separate quota would reduce severly the fishing opportunity for the harpoon boats.

The NMFS proposes to eliminate the harpoon boat category with its separate quota and separate catch limit. These boats would fish under the general category quota of 353 st and the catch limit of one giant tuna per vessel per week.

9. Reduction in the Catch Limit for Anglers Fishing for Young School, School, and Medium Atlantic Bluefin Tuna

The present regulations do not provide a specific quota for this sector of the Atlantic bluefin tuna fishery. Anglers are restricted, however, to a daily catch limit of no more than four young school, school, or medium Atlantic bluefin tuna, provided that only one of the four fish may be a medium tuna.

As previously discussed, NMFS proposes to establish a specific quota for anglers of 99 st purposes for young school, school, and medium tuna. While in most years, the present catch limits have resulted in a total estimated harvest by anglers of around 100 st, the harvest does have the potential for being significantly higher. For example, in 1979 anglers harvested approximately 132 st. Depending on the availability of these size classes of bluefin tuna to the recreational fishery, the number of anglers varies from 5,000 to 40,000 annually. If the present catch limit were maintained, there would be a greater potential for the quota to be exceeded. The NMFS proposes, therefore, to reduce the catch limit from four to two fish per angler per day. This will reduce the potential for exceeding the quota. Further, this reduction in catch limit will allow the benefits from this resource to be spread among the greatest number of participants in this fishery.

10. Miscellaneous Changes to the Regulations

The NMFS has rearranged several of the sections of Subpart A to improve the overall structure and readability of these proposed regulations. The relationship of each section of Subpart A in the proposed regulations to the sections in the present regulations is shown in the following derivation table.

DERIVATION TABLE

New section	Old section
§285.1	§285.2, §285.7, and §285.9.
§285.2	§285.1.
§285.3	§285.3.
§285.4	§285.4.
§285.5	§285.5.
§285.6	§286.6.
§285.7	§285.8.

NMFS proposes to make a number of technical changes and clarifications to the present regulations. The following descriptions explain the proposed changes.

Section 285.1

This section is new as written. It combines material pertaining to the purpose and scope of the regulations which now is distributed unnecessarily throughout the present regulations. Much of the historical discussion of ICCAT contained in § 285.2 of the existing regulations has been deleted as it was inappropriate as regulatory text.

NOAA/NMFS has exercised authority under the Act to make these regulations effective within the boundaries of certain States bordering the ICCAT Convention area, after determining that the affected States did not have regulations in place implementing the ICCAT recommendations then in force or had regulations which were less restrictive or not effectively enforced. This determination was codified as § 285.9. On June 22, 1979, NOAA/NMFS published an interpretation of this section, clarifying that the application of Federal regulations in State waters was not intended to prevent the concurrent application of State regulations which do not conflict with Federal regulations, and are not inconsistent with conservation and management of Atlantic bluefin tuna under ICCAT recommendations.

The text of § 285.1 continues the application of Federal bluefin tuna regulations within the boundaries of the States covered by earlier determinations. As the regulations proposed in this notice would affect fishing within the boundaries of additional States, particularly those bordering the Convention area in the Gulf of Mexico, the Assistant Administrator will consult with such States and make appropriate determinations under § 285.7.

Contain provisions of the Federal regulations were not made effective in the territorial sea adjacent to the State of Maine. Those provisions established quotas for the purse-seine fishery on school size and giant bluefin, incidental allowances of other sizes of bluefin, and a daily catch limit of four school-size fish per day in fisheries other that the purse seine fishery. Until further notice determination, the corresponding provisions of the proposed regulations (§ 285.30(a)(1) and (a)(4), § 285.31.(a), and § 285.32(b)) would not apply within the territorial sea of the United States adjacent to the State of Maine.

Section 285.2

The definitions of "authorized officer" and "Secretary" have been conformed with recent NMFS usage in other sets of fishery regulations. Definitions have

been added for "fork length", "handgear", "metric ton", and "short ton." The definitions of "Council", "quota", and "regulatory area" have been deleted as unnecessary.

Section 285.6

The citation for the procedures for assessing civil penalties was changed from 50 CFR Part 218 to 15 CFR Part 904. This change reflects NOAA's consolidation of a number of different procedural regulations concerning civil penalties into a new single set of regulations. Section 285.6(d) is proposed to be deleted as it no longer reflects current enforcement procedures.

Section 285.21

This section was rewritten extensively to reflect the proposed changes in allowable fisheries. The proposed regulations reduce the number of permit categories from five to two, and specify which purse-seine vessels operating in other tuna fisheries may be issued an incidental catch permit for Atlantic bluefin tuna.

Section 285.22

Paragraphs (d) and (e) were combined. Paragraph (m) of this section was deleted as it applied to buy boats which under the proposed regulations would be prohibited. The citation for the procedures for assessing civil penalties was changed from 50 CFR Part 621 to 15 CFR Part 904

Section 285.23

This section was rearranged to place the requirements for submitting daily reports first, and to correct several inconsistencies in the present regulations. Paragraph (b) of this section was deleted as it applied to buy boats. The requirement for dealers to submit negative weekly reports has been eliminated.

Section 285.25

Paragraphs (e), (g), (h), (q), and (z) of the present regulations were deleted as they applied to vessels engaged in a directed purse-seine fishery, which under the proposed regulations, would be prohibited. Paragraphs (r), (u), and (y) of the present regulations were deleted as unnecessary since these prohibitions are covered adequately in either §285.3, §285.21, or §285.22.

Section 285.28

Paragraphs (a) and (b) of this section were deleted as they pertained to a directed purse-seine fishery. Paragraph (c) of the present regulations was rewritten to delete the requirement for inspection of a vessel prior to commencement of fishing and inspection of fishing gear.

Sections 285.29 through 285.32

These sections have undergone major revisions to reflect the changes of the entire nature of the U.S. fishery as fully described above.

Section 285.33

Paragraph (a) of this section was revised for clarity,

Public hearings

The NMFS will hold public hearings to receive comment on these proposed regulations. The notice announcing the public hearings was published in the **Federal Register** on April 5, 1982 (47 FR 14501). The dates, locations, and times of each hearing are repeated here for informational purposes.

Date	Location	Time
April 19	Quality Inn—Lake Wright, 6280 North Hampton Blvd., Norfolk, VA 23502, Tel: 804/461-6251.	7 p.m.-9:30 p.m.
April 19	Madiera Beach City Hall, 300 Municipal Drive, Madiera Beach, FL 33708, Tel: 813/391-9951.	7 p.m.-9:30 p.m.
April 20	Best Western Exec Inn, 3224 South US1, Ft. Pierce, FL 33450, Tel: 305/465-7000.	7 p.m.-9:30 p.m.
April 21	Best Western International Hotel, 2610 Williams Blvd., Kenner, LA 70062, Tel: 504/466-1401.	7 p.m.-9:30 p.m.
April 22	Texas A&M University, Agricultural Research Center, Agricultural & Extension Serv., Highway 44, 6 miles east of Corpus Christi, TX, Tel: 512/265-9203.	7 p.m.-9:30 p.m.
April 26	Holiday Inn, Route 36, West Long Branch, NJ 07764, Tel: 201/229-9000.	7 p.m.-9:30 p.m.
April 27	Holiday Inn of Riverhead, Route 25, Riverhead, Long Island, NY 11901, Tel: 516/369-2200.	7 p.m.-9:30 p.m.
April 28	Holiday Inn, U.S. Route 1, 1 Newbury Street, Peabody, MA 01960, Tel: 617/535-4600.	7 p.m.-9:30 p.m.
April 29	Holiday Inn—West, 81 Riverside Street, Portland, ME 04103, Tel: 207/774-5601.	7 p.m.-9:30 p.m.
May 3	Governor Carver Motor Inn, 25 Summer Street, Plymouth, MA 02360, Tel: 617/746-7100.	7 p.m.-9:30 p.m.

Classification

The NOAA Administrator has determined that these regulations are not major under Executive Order 12291. A Regulatory Impact Review has been prepared which describes the expected benefits and costs of the proposed regulatory action. The review provides the basic for the Administration's determination.

The NOAA Administrator also has determined that these regulations will have a significant economic impact upon a substantial number of small entities under the Regulatory Flexibility Act. An initial Regulatory Flexibility Analysis has been prepared and is available to the public as a combined Environmental Impact Statement/Regulatory Impact Review/Initial Regulatory Flexibility Analysis.

Appendix B of the 1980 Final Environmental Impact Statement discussed the participants who will be affected by this action. Since 1980, the number of applications (Federal Fisheries Permit, OMB 0648–0097) for permits in the giant bluefish tuna fishery (all categories) has increased by approximately 30 persons. The number of recreational anglers in the school-size fishery has increased by an estimated three percent.

These proposed regulations continue existing data reporting requirements essential for fishery management. There is a minimum amount of mandatory record keeping and data reporting which constitute "collection of information" under the Paperwork Reduction Act. The present requirement for fish dealers to report all transactions involving Atlantic bluefin tuna is continued (OMB form 0648–0013).

The draft Environmental Impact Statement (DEIS) has been prepared under Sections 102(2)(c) and 702(d) of the National Environmental Policy Act of 1969. The notice of intent to prepare an Environmental Impact Statement was published in the **Federal Register** on March 23, 1982 (47 FR 12367). The DEIS was filed with the Environmental Protection Agency (EPA) and made available to the public on April 12, 1982. The combined DEIS/RIR/RFA is available from the NMFS, Northeast Regional Office (see address above). Comments on the DEIS/EIR/RFA will be recieved until May 19, 1982. Comments received will be revised and summarized in the final EIS/RIR/RFA which will be filed with EPA prior to the promulgation of final rulemaking. Copies of all comments will be open for inspection at the NMFS, Northeast Region office (address above).

The Administrator, NOAA, has found good cause for this rulemaking to be exempt from section (3)(c)(3) of E.O. 12291 under section 8(a) of that Order. Transmitting this proposed rule to the Director of the Office of Management and Budget (OMB) at least 10 days before publication would cause a delay in implementing final regulations necessary to insure resource conservation and to meet an international commitment. A copy of this notice is being transmitted to OMB

simultaneously with publication in the **Federal Register.**

List of Subjects in 50 CFR Part 285

Fish, Fisheries, Fishing.

Robert K. Crowell,

Deputy Executive Director, National Marine Fisheries Service.

PART 285—ATLANTIC TUNA FISHERIES

1. The authority citation for 50 CFR Part 285 is:

Authority: 16 U.S.C. 971–971h.

2. It is proposed to revise Subparts A and B of 50 CFR Part 285 to read as follows:

Subpart A—General

Sec.
285.1 Purpose and scope.
285.2 Definitions.
285.3 Prohibitions.
285.4 Civil penalties.
285.5 Enforcement.
285.6 Civil procedures.
285.7 Relationship to other laws and regulations.

Subpart B—Atlantic Bluefin Tuna (Thunnus thynnus thynnus)

285.20 Effective period of regulations.
285.21 Vessel permits.
285.22 Dealer licenses.
285.23 Dealer recordkeeping and reporting.
285.24 Metal tags.
285.25 Prohibitions.
285.26 Presumptions.
285.27 Penalties.
285.28 Purse-seine vessel inspection.
285.29 Permitted fishing.
285.30 Quotas.
285.31 Incidental catch.
285.32 Catch limits.
285.33 Tag and release program.

Authority: 16 U.S.C. 971–971h.

Subpart A—General

§ 285.1 Purpose and scope.

(a) The Atlantic Tunas Convention Act of 1975 (16 U.S.C. 971–971h) authorizes the Secretary to implement the recommendations of the International Commission for the Conservation of Atlantic Tunas (ICCAT). The Secretary's authority under the Act has been delegated to the Assistant Administrator.

(b) These regulations implement the ICCAT recommendations for persons and vessels subject to the jurisdiction of the United States.

(c) This part does not apply to any person or vessel authorized by the Commission, the Regional Director, the Director of the Southeast Fisheries Center, or any State upon approval by the Regional Director, to engage in fishing for research purposes.

(d) Under section 9(d) of the Act and § 285.7, the Assistant Administrator has determined that the provisions of this part apply within the territorial sea of the United States adjacent to the States of Florida, Georgia, South Carolina, North Carolina, Virginia, Maryland, Delaware, New Jersey, New York, Massachusetts, New Hampshire, and the Commonwealths of Puerto Rico and the Virgin Islands, and, with the exceptions of § 285.30(a)(1) and (a)(4), § 285.31(a), and § 285.32(b), within the territorial sea of the United States adjacent to the State of Maine.

§ 285.2 Definitions.

In addition to the meaning given by the Act, if any, the following terms as used in this part shall mean:

"Act" means the Atlantic Tunas Convention Act of 1975, 16 U.S.C. 971–971h.

"Albacore" means the fish species *Thunnus alalunga.*

"Angling" means fishing for or catching, or the attempted fishing for or catching, fish by any person (angler) with a hook attached to a line which is hand held or by rod and reel designed or manufactured for this purposes.

"Assistant Administrator" means the Assistant Administrator for Fisheries, National Oceanic and Atmospheric Administration.

"Atlantic bluefin tuna" means the fish species *Thunnus thynnus thynnus.* Class sizes for Atlantic bluefin tuna are defined in § 285.26.

"Atlantic bonito" means the fish species *Sarda chiliensis* or *Sarda sarda.*

"Authorized Officer" means

(a) Any commissioned, warrant, or petty officer of the United States Coast Guard;

(b) Any special agent of the NMFS;

(c) Any officer designated by the head of any Federal or State agency which has entered into an agreement with the Secretary and the Commandant of the U.S. Coast Guard to enforce the provisions of the Act; or

(d) Any U.S. Coast Guard personnel accompanying and acting under the direction of any person described in paragraph (a) of this definition.

"Bigeye tuna" means the fish species *Thunnus obesus.*

"Buy boat" means any vessel used by a dealer in purchasing or receiving Atlantic bluefin tuna from any person or fishing vessel engaged in fishing for such tuna.

"Cargo vessel" means any fishing vessel used for transporting fish or fish products.

"Commercial activity" means any activity, other than fishing, or industry, trade, and commerce including but not limited to the buying or selling of a regulated species and activities conducted for the purpose of facilitating such buying and selling.

"Commission" means the International Commission for the Conservation of Atlantic Tunas established under Article III of the Convention.

"Convention" means the International Convention for the Conservation of Atlantic Tunas, signed at Rio de Janeiro May 14, 1966, 20 U.S.T. 2887, TIAS 6767, including any amendments or protocols thereto, which are binding upon the United States.

"Dealer" means any person who engages in a commercial activity with respect to a regulated species or parts thereof.

"Dressed weight" means the weight of a fish after it has been gilled, gutted, beheaded, and definned.

"Fish" or "fishing" means the catching or fishing for, or the attempted catching or fishing for, any species of fish covered by the Convention, or any activities in support of fishing.

"Fishing trip" means the time period between when a fishing vessel departs from port to carry out fishing operations and the time such vessel returns to port or offloads any of its catch.

"Fishing vessel" means any vessel engaged in fishing, processing, or transporting fish loaded on the high seas, or any vessel outfitted for such activities.

"Fishing week" means a period of time beginning at 0001 hours local time on Sunday, and ending at 2400 hours local time on the following Saturday.

"Fork length" means a measurement of the length of Atlantic bluefin tuna taken in a straight line along the middle of the lateral surface from the tip of the snout to the fork of the tail.

"Handgear" means handline, harpoon, or rod and reel.

"Handline" or "handline gear" means fishing gear which is released by hand and consists of one main line of variable length to which is attached one or two leaders and hooks. Handlines are retrieved only by hand, and not by mechanical means.

"Longline" or "Longline gear" means fishing gear which is set horizontally, either anchored, floating, or attached to a vessel, which consists of a main or groundline of three or more gangions and hooks. A longline may be retrieved by hand or mechanical means.

"Metal tag" means the flexible, selflocking ribbon of metal issued by the NMFS for the identification of Atlantic bluefin tuna required under § 285.24.

"Metric ton" (mt) means 2204.6 pounds (1000 kilograms).

"NMFS" means the National Marine Fisheries Service, National Oceanic and Atmospheric Administration.

"Person" means any individual, partnership, corporation, or association subject to the jurisdiction of the United States.

"Plastic tag" means the plastic or combination plastic and metal marker which is issued with a tag and release permit under § 285.33(b).

"Purse seining" means fishing for or catching a regulated species by means of an encircling net and associated gear.

"Regional Director" means:

(a) For purposes of Atlantic bluefin tuna, the Regional Director, Northeast Region, National Marine Fisheries Service, Federal Building, 14 Elm Street, Gloucester, Massachusetts 01930–3799; and

(b) For purposes of yellowfin tuna, the Regional Director, Southwest Region, National Marine Fisheries Service, 300 South Ferry Street, Terminal Island, California 90731.

"Regulated species" means Atlantic bluefin tuna, yellowfin tuna, skipjack tuna, albacore, or bigeye tuna.

"Reporting week" means a period of time beginning at 0001 hours local time on Sunday, and ending at 2400 hours local time the following Saturday.

"Round weight" means the weight of a fish before gilling, gutting, beheading, and definning.

"Secretary" means the Secretary of Commerce or an individual to whom appropriate authority has been delegated.

"Short ton" (st) means 2,000 pounds (907 kilograms).

"Skipjack tuna" means the fish species *Katsuwonus pelamis.*

"State" means the States of the United States, the District of Columbia, the Commonwealths of Puerto Rico and the Virgin Islands, and territories and possessions of the United States.

"Tuna" means Atlantic bluefin tuna, yellowfin tuna, skipjack tuna, albacore, or bigeye tuna.

"Yellowfin tuna" means the fish species *Thunnus albacares.*

§ 285.3 Prohibitions.

It is unlawful—

(a) For any person in charge of a fishing vessel or for any fishing vessel subject to the jurisdiction of the United States to engage in fishing or to land any tuna in violation of these regulations.

(b) For any person to land, transship, ship, transport, purchase, sell, offer for sale, import, export, or have in custody, possession, or control any fish which the person knows, or should have known, was taken or retained contrary to this part, without regard to the citizenship of the person or registry of the fishing vessel which took the fish.

(c) For a dealer or any person in charge of any fishing vessel subject to the jurisdiction of the United States to fail to make, keep, or furnish reports required by this part.

(d) For a dealer or any person in charge of any fishing vessel subject to the jurisdiction of the United States to obstruct or to refuse to allow any authorized officer to enter the dealer's premises or to board the vessel in order to search or inspect its catch, equipment, books, documents, records, or other articles, or to question the persons onboard under the provisions of this part.

(e) For any person to import from any country any regulated species in any form subject to regulation under a recommendation of the Commission, or any fish in any form not under regulation but under investigation by the Commission, during the period such fish have been denied entry under this part.

§ 285.4 Civil penalties.

Any person who—

(a) Violates any provision of § 285.3 (a) or (b) shall be assessed a civil penalty of not more than $25,000 and for any subsequent violation of said section shall be assessed a civil penalty of not more than $50,000;

(b) Violates any provision of § 285.3 (c) or (d) shall be assessed a penalty of not more than $1,000 and for any subsequent violation of said subsection shall be assessed a civil penalty of not more than $5,000; or

(c) Violates any provision of § 285.3(e) shall be assessed a civil penalty of not more than $100,000.

§ 285.5 Enforcement.

(a) This part and the provisions of the Act shall be enforced jointly by the Secretary, the Secretary of the Department in which the United States Coast Guard is operating, and the United States Customs Service.

(b) Enforcement Agents of the National Marine Fisheries Service shall enforce provisions of this part and the Act on behalf of the Secretary and may take any actions authorized under this Part and the Act with respect to enforcement. By agreement, the Secretary may utilize the personnel, services, and facilities of any other Federal Agency to enforce the Act and, also may designate personnel of a State to enforce the Act.

§ 285.6 Civil procedures.

(a) The method for assessment of civil penalties for violation of these regulations or the Act shall be in accordance with the rules and procedures set forth in 15 CFR Part 904.

(b) In view of the perishable nature of tuna when not processed otherwise than by chilling or freezing, authorized officers may cause to be sold, for not less than its reasonable market value, unchilled or unfrozen tunas that may be seized and forfeited under the Act and this part.

(c) The proceeds of any sale made under paragraph (b) of this section shall be remitted by the purchaser to the Regional Director. The Regional Director shall deposit and retain the proceeds in the Suspense Account of the NMFS (Account No. 14x6875(17)) after deducting the reasonable cost of the sale, if any, pending judgment of the court or other disposition of the case.

§ 285.7 Relationship to other laws and regulations.

(a) These regulations apply within the boundaries of any State bordering the convention area if the Assistant Administrator determines, and so notifies the State, that the State:

(1) Has not enacted laws or promulgated regulations which implement the recommendations of the Commission within its waters within 30 days after the promulgation of these regulations; or

(2) Has enacted such laws or promulgated such regulations, but such laws or regulations are less restrictive than the regulations promlugated under the Act to implement such recommendations of the Commission, or are not being enforced effectively.

(b) To ensure that the purposes of this section are carried out, the Assistant Administrator shall review continually the relevant laws and regulations of the pertinent States and the extent to which such laws and regulations are enforced.

(c) Upon notice of a determination adverse to its interests under § 285.7(a), a State may request a hearing on the record.

Subpart B—Atlantic Bluefin Tuna (Thunnus thynnus thynnus)

§ 285.20 Effective period of regulations.

This subpart shall remain in effect until superseded, amended, or otherwise suspended.

§ 285.21 Vessel permits.

(a) *Permit requirements.* Any vessel which fishes for or takes Atlantic bluefin tuna, except vessels being used by anglers fishing for young school, school, or medium Atlantic bluefin tuna under § 285.32(b), shall have an

appropriate permit issued under this section.

(b) *Categories of permits.* Each vessel shall be permitted in only one of the following categories: General (handgear), or Incidental Catch (purse seine, longline, traps, or fixed gear). A vessel shall fish only under the quota for the category in which it is permitted, and shall use only gear appropriate to that category. Only those purse seine vessels, or their replacements, which were granted allocations and landed Atlantic bluefin tuna in the directed fishery for Atlantic bluefin tuna during the period 1980 through 1981 shall be permitted to take Atlantic bluefin tuna incidentally as described in § 285.31(a). Any vessel which its owners retired from the fishery and replaced with another vessel during the period 1980 through 1981 shall not be permitted to take Atlantic bluefin tuna under § 285.31(a).

(c) *Registration procedure.* All permit applications under this section shall be submitted and signed by the vessel owner on an appropriate form obtained from the Regional Director. The application shall be submitted to the Regional Director at least 30 days before the date on which the applicant desires to have the permit made effective. The application shall include the name and address of the vessel owner, the name of the vessel, the port where the vessel is docked, the official registration or documentation number, the length of the vessel, the tonnage (if known), the area to be fished, and the category in which the vessel is to be registered.

(d) *Issuance.* (1) The Regional Director shall issue a permit within 30 days of receipt of a completed application.

(2) The Regional Director shall notify the applicant of any deficiency in the application. If the applicant fails to correct the deficiency within 15 days following the date of notification, the application shall be considered abandoned.

(e) *Duration.* A permit issued under this section shall remain valid until it expires or is revoked. Permits issued under this section expire when the owner or name of the vessel changes.

(f) *Alteration.* Any permit issued under this section which is substantially altered, erased, or mutilated is invalid.

(g) *Replacement.* Replacement permits may be issued by the Regional Director. An aplication for a replacement permit shall not be considered a new application.

(h) *Transfer.* Permits issued under this section are not transferable or assignable. A permit is valid only for the vessel and owner for which it is issued.

(i) *Display.* Any permit issued under this section shall be carried on board the vessell at all times. The permit shall be displayed for inspection upon request of any Authorized Officer or any employee of the NMFS designated by the Regional Director for such purpose.

(j) *Modification.* Permits issued under this section may be modified, suspended, or revoked by the Assistant Administrator for violations of the Act, or any of the Act's implementing regulations. Any modification, suspension, or revocation, shall follow the procedures of 15 CFR Part 904.

(k) *Fees.* No fee is required for any permit issued under this section.

(l) *Change in application information.* Any change in the information contained in an application submitted for a permit under this section shall be reported in writing to the Regional Director within 15 days of the change.

§ 285.22 Dealer licenses.

(a) *General.* Any person purchasing or receiving Atlantic bluefin tuna for a commercial purpose from any person or vessel fishing for such tuna shall have a valid license required under this section.

(b) *Application.* An application for a dealer license shall be made in writing, signed by the applicant, and submitted to the Regional Director at least 30 days before the date upon which the applicant desires to have the license made effective. Applications shall contain the following information:

(1) The name, mailing address, and telephone number of the applicant; and

(2) The expected disposition of all Atlantic bluefin tuna purchased (e.g., foreign or domestic markets).

(c) *Issuance.* (1) The Regional Director shall issue a license within 30 days of receipt of a completed application.

(2) The Regional Director shall notify the applicant of any deficiency in the application. If the applicant fails to correct the deficiency within 15 days following the date of notification, the application shall be considered abandoned.

(d) *Duation.* Any license issued under this section shall remain valid until December 31 of the year for which it is issued, or until it is suspended or revoked.

(e) *Alteration.* Any license which is substantially altered, erased, or mutilated is invalid.

(f) *Replacement.* Replacement licenses may be issued by the Regional Director. An application for a replacement license shall not be considered a new application.

(g) *Transfer.* Licenses issued under this section are not transferable or assignable. A license shall be valid only for the dealer to whom it issued.

(h) *Inspection.* A license issued under this section shall be kept at the principal place of business of the dealer. The license shall be displayed for inspection upon request of any Authorized Officer, or any employee of the NMFS designated by the Regional Director for that purpose.

(i) *Modification.* Licenses issued under this section may be modified, suspended, or revoked by the Assistant Administrator for violations of the Act, or any of the Act's implementing regulations. Any modification, suspension, or revocation shall follow the procedures of 15 CFR Part 904.

(j) *Fees.* No fee is required for any license issued under this section.

(k) *Change in application information.* Any change in the information specified in this section shall be reported to the Regional Director within 15 days of the change.

§ 285.23 Dealer recordkeeping and reporting.

Any person issued a dealer license under § 285.22:

(a) Shall submit to the Regional Director a daily report on a reporting card provided by the NMFS, within 24 hours of the purchase of each giant Atlantic bluefin tuna. Each card shall show the Atlantic bluefin tuna vessel permit number, metal tag number affixed to the fish by the dealer or assigned by an Authorized Officer, the date landed, the port where landed, the round or dressed weight, the fork length, gear used, and area where caught. This report will be signed by the vessel permit holder or vessel operator.

(b) Shall submit to the Regional Director a weekly report on forms supplied by the NMFS, within two days after the end of each reporting week in which Atlantic bluefin tuna were purchased or received. Each report shall specify accurately and completely: The number of tuna purchased or received; location where the tuna was caught; the disposition of the tuna (names, addresses and, where applicable, country of destination); the source of the tuna (names and addresses); date; metal tag numbers (where applicable); round or dressed weight and fork length (by individual fish or giant tuna); and any other information requested by the Regional Director.

(c) Shall allow an Authorized Officer, or any employee of the NMFS designated by the Regional Director for this purpose, to inspect any records of transfers, purchases, or receipts of Atlantic bluefin tuna.

(d) Shall retain in his/her possession a copy of each weekly report for a period of two years from the date on wihch it was submitted to the Regional Director.

§ 285.24 Metal tags.

(a) *Issuance of tags.* Any person receiving a dealer's license under the provisions of § 285.22 shall be issued numbered metal tags by the NMFS.

(b) *Transfer of tags.* Tags issued under paragraph (a) of this section are not transferable.

(c) *Affixing tags.* (1) Giant Atlantic bluefin tuna shall be tagged immediately by a dealer or his agent upon offloading from a vessel. The tag, issued under paragraph (a) of this section, shall be affixed to the tuna between the fifth dorsal finlet and the keel.

(2) Any person who catches a giant Atlantic bluefin tuna and does not transfer it to a licensed dealer shall contact the nearest NMFS enforcement office within 24 hours of landing such tuna to make the tuna available for inspection and attachment of the metal tag.

(d) *Removal of tags.* A metal tag affixed to any giant Atlantic bluefin tuna shall remain on the tuna until the tuna is either cut into portions for personal consumption or for commercial purposes, or sold for export from the United States. If the tag is removed from the tuna for commercial or export purposes, the tag shall be attached to the outside of the container and the tag number must be written legibly and indelibly on the outside of any package or container which holds the tuna or tuna parts and which is being exported or transported domestically for commercial use. If a giant Atlantic bluefin tuna is packed for export, the tag shall also be attached to the container until it is shipped from the United States.

§ 285.25 Prohibitions.

It shall be unlawful for any person or vessel subject to the jurisdiction of the United States:

(a) To fish for or catch Atlantic bluefin tuna without a valid permit required under § 285.21 and carried onboard the vessel;

(b) To fish for or catch Atlantic bluefin tuna after fishing has been closed or before fishing has commenced under § 285.29, except under the provisions of § 285.31 or § 285.33;

(c) To fish for or catch Atlantic bluefin tuna in excess of the quotas specified in § 285.30 except under the provisions of § 285.31 or § 285.33;

(d) To fish for or catch Atlantic bluefin tuna in excess of the catch limits specified in § 285.32;

(e) To fish for or catch Atlantic bluefin tuna within 91.5 meters (100 yards) of the cork line of a purse seine net used by any vessel conducting scientific research operations authorized by the NMFS;

(f) To catch and retain Atlantic bluefin tuna in excess of the incidental catch allowed in § 285.31;

(g) To land any Atlantic bluefin tuna in forms other than round, or eviscerated with the head removed;

(h) To retain any Atlantic bluefin tuna caught under a tag and release permit issued under § 285.33;

(i) To purchase, receive, or transfer any Atlantic bluefin tuna at sea from a person or vessel engaged in fishing for such tuna;

(j) To sell, offer for sale, or transfer any Atlantic bluefin tuna to any person other than a licensed dealer under § 285.22;

(k) To engage in fishing with a vessel holding a permit under § 285.21(a) unless the vessel travels to and from the area where it will be fishing under its own power and the person operating that vessel brings under control any Atlantic bluefin tuna (secured to the catching vessel or boated) with no assistance from other vessels, except in circumstances where the safety of the vessel of its crew is jeopardized or due to other circumstances beyond the control of the operator;

(l) To fail to release immediately with a minimum of injury any Atlantic bluefin tuna which will not be retained;

(m) To fail to affix immediately to any giant Atlantic bluefin tuna, between the fifth dorsal finlet and the keel, an individually numbered metal tag when the tuna has been received or purchased by that person for a commercial purpose from any person or vessel having caught such tuna;

(n) To remove any metal tag affixed to an Atlantic bluefin tuna pursuant to § 285.24 before removal is allowed under that section, or fail to write the tag number on the shipping package or container as prescribed by that section;

(o) To begin off-loading from any purse-seine vessel to which a permit has been issued under § 285.21 any Atlantic bluefin tuna without first requesting an inspection of the vessel by a Special Agent of the NMFS in accordance with § 285.28;

(p) To fail to report the taking of any Atlantic bluefin tuna to which a plastic tag has been affixed under a bona fide tag and release program conducted by the NMFS or any other recognized scientific organization;

(q) To falsify or fail to make, keep, maintain, or submit any reports, or other record required by this subpart;

(r) To refuse to allow an Authorized Officer to make inspections for the purpose of checking any records relating to the catching, harvesting, landing, purchase or sale of any Atlantic bluefin tuna required by this subpart;

(s) To make any false statement, oral or written, to an Authorized Officer concerning the catching, harvesting, landing, purchase, sale, or transfer of any Atlantic bluefin tuna;

(t) To fish for or catch giant Atlantic bluefin tuna with longline gear except as provided in § 285.31(d);

(u) To fish for or catch giant Atlantic bluefin tuna with longline gear, or while having longline gear on board, if the vessel is registered in the general category pursuant to § 285.21(b);

(v) To purchase or transport with a buyboat any Atlantic bluefin tuna;

(w) To violate any other provision of this subpart, the Act, or any other regulations promulgated under the Act.

§ 285.26 Presumptions.

For any Atlantic bluefin tuna which is landed eviscerated with the head removed, there shall be a rebuttable presumption for purposes of this subpart that the tuna when caught fell into a size class in accordance with the following table. For this purpose, all measurements must be taken in a straight line along the midde of the lateral surface from the forward most part of the beheaded fish to the fork of the tail. Approximate weights are given for illustrative purposes only.

Original size class (Fork length)	Approximate weights (whole fish)	Length with head off
Young school tuna: Less than 26 inches (66 cm).	Less than 14 pounds.	Less than 18 inches (46 cm).
School tuna: Equal to 26 inches (66 cm) but less than 57 inches (145 cm).	From 14 pounds but less than 135 pounds.	18 inches (46 cm) but less than 40 inches (102 cm).
Medium tuna: Equal to 57 inches (145 cm) but less than 77 inches (196 cm).	From 135 pounds but less than 310 pounds.	40 inches (102 cm) but less than 54 inches (137 cm).
Giant tuna: 77 inches (196 cm) or more.	Larger than 310 pounds.	54 inches (137 cm) and over.

§ 285.27 Penalties.

(a) Any person who violates paragraphs (a) through (p) inclusive, or paragraphs (u) through (y), inclusive, of § 285.25 shall be assessed a civil penalty of not more than $25,000; and for a subsequent violation shall be assessed a civil penalty of not more than $50,000.

(b) Any person who violates paragraphs (q) through (t), inclusive, of § 285.25 shall be assessed a civil penalty

of not more than $1,000, and for a subsequent violation shall be assessed a civil penalty of not more than $5,000.

(c) Any person who violates paragraph (z) of § 285.25 shall be assessed a civil penalty in accordance with the criteria set forth in 16 U.S.C. 971e.

§ 285.28 Purse-seine vessel inspection.

Any owner or operator of a purse seine vessel with a permit issued under § 285.21 shall request an inspection of the vessel by a Special Agent of the NMFS before offloading any Atlantic bluefin tuna. Requests for such inspection shall be made by calling (617) 281-3600 extension 252; or (617) 992-7711. Requests shall be made at least 24 hours before offloading.

§ 285.29 Permitted fishing.

(a) *School and giant Atlantic bluefin tuna.*—(1) *Commencement.* (i) Vessels permitted in the Incidental Catch category may fish in the regulatory area beginning on January 1 of each year. Vessels permitted in the general category may fish in the regulatory area beginning on July 15 of each year.

(ii) Consistent with the Convention, the Act and this part, the Assistant Administrator may change the commencement date for fishing under this section for any size class of Atlantic bluefin tuna for any area and vessel gear type if the Assistant Administrator determines that such date will enable scientific research on the status of the stock to be conducted more effectively and will not prevent the quotas for the affected fishery from being caught, based upon historical catch data or other relevant information. The Assistant Administrator shall publish a notice in the **Federal Register** of any change in the commencement date(s) for fishing under this section at least 60 days before commencement of the affected fishery. Nothing in this paragraph shall be construed to invalidate any more restrictive commencement date established by any State in waters under its jurisdiction.

(2) *Closure.* (i) The Assistant Administrator shall monitor catch and landing statistics of Atlantic bluefin tuna. On the basis of these statistics, the Assistant Administrator shall project a date when the total catch of Atlantic bluefin tuna will exhaust any quota under § 285.30, and shall publish a notice in the **Federal Register** stating that the catching and retaining of the Atlantic bluefin tuna by the type of vessels subject to the quota must cease on that date at a specified hour.

(ii) *Angling.* Following such a closure, anglers may continue to fish for Atlantic bluefin tuna under a tag and release program under § 285.33.

(b) *Young School Atlantic Bluefin Tuna.* Fishing for or catching young school Atlantic bluefin tuna by vessels using purse seines or gear other than purse seines in the regulatory area shall be limited at all times during the year to an incidental catch under § 285.31, except that anglers may fish for or catch young school Atlantic bluefin tuna at any time under the limitations specified in § 285.32(b).

§ 285.30 Quotas.

(a) *Total quota.* The total amount of Atlantic bluefin tuna which may be caught and retained in the regulatory area is 667 st (605 mt).

(1) The total amount of giant Atlantic bluefin tuna which may be caught and retained in the regulatory area by vessels permitted in the general category under § 285.21(b) is 353 st (320 mt). No more than 327 st (296 mt) shall be taken before September 1 east and north of a line drawn north and south through Gay Head, Martha's Vineyard (700°50' W. longitude).

(2) The total amount of young school, school, and medium Atlantic bluefin tuna which may be caught and retained in the regulatory area by angling is 99 st (90 mt).

(3) The total amount of Atlantic bluefin tuna which may be caught and retained by longline vessels permitted in the incidental-catch category under § 285.21(b) is 44 st (40 mt)

(4) The total amount of Atlantic bluefin tuna which may be caught and retained by purse seine vessels permitted in the incidental-catch category under § 285.21(b) is 171 st (155 mt).

(b) *Incidental catches.* Any Atlantic bluefin tuna taken incidentally to fishing operations for other species shall be included in the quotas and subquotas of this section.

§ 285.31 Incidental catch.

(a) Purse-seine vessels fishing for tunas other than Atlantic bluefin tuna and possessing an Atlantic bluefin tuna incidental-catch permit under § 285.21 may catch, during any fishing trip, Atlantic bluefin tuna of any size class; provided that the total amount of Atlantic bluefin tuna taken does not exceed 10 percent by weight of all other tuna species onboard the vessel at the end of each fishing trip.

(b) *Herring, mackerel, and menhaden purse seine vessels and vessels using fixed gear other than traps (pounds, weirs, and gill-nets) or longlines.* Any person operating a vessel fishing principally for species of fish other than tuna and possessing an Atlantic bluefin tuna incidental-catch permit under § 285.21 may catch, during any fishing trip, Atlantic bluefin tuna of any size class: *Provided* That, the total amount of Atlantic bluefin tuna taken does not exceed 2 percent, by weight, of all other fish onboard the vessel at the end of each fishing trip.

(c) *Traps.* Any person operating a vessel with an incidental-catch permit issued under § 285.21 which catches Atlantic bluefin tuna incidentally while fishing with traps, may retain Atlantic bluefin tuna: Provided, that such tuna do not exceed 2 percent, by weight, of the total amount of all other fish species caught within the preceding 30-day period.

(d) *Longlines.* Any person operating a vessel using longline gear with an incidental-catch permit issued under § 285.21 may land giant Atlantic bluefin tuna as an incidental catch. The amount of Atlantic bluefin tuna shall not exceed:

(1) Two fish per vessel, per trip, south of 36° N. latitude, and

(2) Two percent by weight of all other fish on board at the end of each fishing trip, north of 36° N. latitude.

§ 285.32 Catch limits.

(a) Vessels registered in the general category may catch and retain only one giant Atlantic bluefin tuna per week, per vessel with a possession limit of only one tuna at any time.

(b) *Young school, school, and medium tuna.* Persons angling in the regulatory area may catch and retain no more than two young school, school, or medium Atlantic bluefin tuna each day, only one of which may be a medium.

§ 285.33 Tag and release program.

(a) *Tag and release permits.* Any angler who wishes to tag and release Atlantic bluefin tuna shall obtain a valid tag and release permit, and tags issued by the NMFS under paragraph (b) of this section. A tag and release permit and tags may be obtained by sending an application to the Atlantic Bluefin Tuna

Tagging Program, Southeast Fisheries Center, NMFS, 75 Virginia Beach Drive, Miami, Florida 33149. The application shall include the name of the vessel, official United States Coast Guard or State number(s), name(s) of the owner and master, and the general area(s) in which the tag and release activity will be carried out. The Center Director shall issue a tag and release permit and tags within 30 days of receipt.

(b) *Plastic tags.* Anglers receiving a tag and release permit under paragraph (a) of this section also shall receive plastic tags and detailed instructions of use of these tags. All Atlantic bluefin tuna caught under a tag and release permit shall be tagged before they are released. Such tuna shall be released immediately with a minimum of injury.

[FR Doc. 82-10941 Filed 4-20-82; 8:45 am]
BILLING CODE 3510-22-M

Notices

Federal Register
Vol. 47, No. 77
Wednesday, April 21, 1982

This section of the FEDERAL REGISTER contains documents other than rules or proposed rules that are applicable to the public. Notices of hearings and investigations, committee meetings, agency decisions and rulings, delegations of authority, filing of petitions and applications and agency statements of organization and functions are examples of documents appearing in this section.

CIVIL AERONAUTICS BOARD

Application of Avia International Airlines, Inc. for Transfer and Reissuance of Certificates

AGENCY: Civil Aeronautics Board.

ACTION: Notice of Order to Show Cause, 82-4-84, Dockets 39898, 40019.

SUMMARY: The Board has tentatively decided to transfer the certificates of Challenge Air Transport, Inc. to Avia International Airlines, Inc., and reissue them in the name of Challenge Air Transport, Inc., and to amend the carrier's section 401 certificate to authorize it to engage in worldwide charter air transportation of cargo.

OBJECTIONS: All interested persons having objections to the Board's tentative findings and conclusions that this action be taken, as described in the order cited above, shall no later than May 14, 1982, file a statement of such objections with the Civil Aeronautics Board (20 copies, addressed to Dockets 39898 and 40019, Dockets Section, Civil Aeronautics Board, Washington, D.C. 20428) and mail copies to Avia International Airlines, Inc., Challenge Air Transport, Inc., the Departments of State and Transportation, and the Attorney General. A statement of objections must cite the docket number and must include a summary of testimony, statistical data or other such supporting evidence.

If no objections are filed, the Secretary of the Board will enter an order whicl. ill make final the Board's tentative findings and conclusions, transfer and reissue the certificates, and subject to the disapproval of the President under section 801(a) of the Act, amend the carrier's certificate to authorize it to engage in worldwide charter air transportation of cargo.

To get a copy of the complete order, request it from the Civil Aeronautics Board, Distribution Section, Room 100, 1825 Connecticut Avenue, N.W., Washington, D.C. 20428. Persons outside the Washington Metropolitan area may send a postcard request.

FOR FURTHER INFORMATION CONTACT: Rhonda Starck, (202) 673-5035, Legal Division, Bureau of International Aviation, Civil Aeronautics Board, Washington, D.C. 20428.

By the Civil Aeronautics Board: April 15, 1982.

Phyllis T. Kaylor,
Secretary.

[FR Doc. 82-10870 Filed 4-20-82; 8:45 am]
BILLING CODE 6320-01-M

[Order 82-4-62; Docket 40132]

Flying Tiger Line Inc., et al.; Order

Adopted by the Civil Aeronautics Board at its office in Washington, D.C. on the 12th day of April, 1982.

On October 14, 1981, The Flying Tiger Line Inc. (Flying Tiger) filed a complaint against the Canadian Transport Commission (CTC) and Air Canada, the Canadian flag carrier. The complaint states that the CTC has denied Flying Tiger the necessary authority to provide scheduled all-cargo service between California and Toronto/Montreal via Chicago and that this action violates Exchange of Notes, dated January 17, 1966, between the governments of the United States and Canada which addresses scheduled all-cargo operations between the two countries. Flying Tiger stated that the decision of the CTC warrants remedial action under section 2 of the International Air Transportation Fair Competitive Practices Act of 1979, as amended, (IATFCPA).

We have received comments and answers from the Department of Transportation, Air Canada, and several other Canadian carriers. While DOT supports Flying Tiger's position, all of the Canadian carriers urge the Board to deny the complaint.[1]

On March 29, 1982, Flying Tiger filed a motion with the Board requesting dismissal of its complaint without prejudice to refiling if it is unable to obtain all-cargo operating authority from the Canadian Government. The carrier states that a dismissal of its complaint "at this point in time will better facilitate its long-sought entry to Canada than would commercial retaliatory action."

We have decided to grant Flying Tiger's request. We find that this action is consistent with the public interest because it will afford the United States and Canada a further opportunity to resolve this matter through negotiations. In this connection we note that aviation discussions between the two countries are scheduled to resume next month and that the question of additional all-cargo service in the United States-Canada market is among the issues that will be discussed.

Accordingly,

1. We dismiss the complaint filed by The Flying Tiger Line Inc. in Docket 40132 without prejudice;
2. We shall serve this order upon The Flying Tiger Line Inc., Air Canada, Wardair, CP Air, Quebecair, Pacific Western Airlines, Ltd., the Ambassador of Canada in Washington, D.C., and the Departments of State and Transportation; and
3. We may amend, modify or revoke this order at our discretion without a hearing.

We shall publish this order in the **Federal Register.**

By the Civil Aeronautics Board:[2]

Phyllis T. Kaylor,
Secretary.

[FR Doc. 82-10869 Filed 4-20-82; 8:45 am]
BILLING CODE 6320-01-M

[1] We have previously extended the date for Board action on the complaint pending aviation discussions between the United States and Canada. See Orders 82-2-61 and 82-3-61.

[2] All members concurred.

DEPARTMENT OF COMMERCE

International Trade Administration

Export Promotion Subcommittee of the President's Export Council; Open Meeting

AGENCY: International Trade Administration, Commerce.

SUMMARY: The President's Export Council was initially established by Executive Order 11753 of December 20, 1973. The Council was reconstituted by Executive Order 12131 of May 4, 1979, and continued by Executive Order 12258 of December 31, 1980. The Council's purpose is to advise the President on matters relating to United States export trade. The Export Promotion

Subcommittee was formed by the Council to study ways to promote U.S. export trade.

TIME AND PLACE: May 6, 1982, from 2:00 p.m.–5:00 p.m. The meeting will be held at the Department of Commerce, 14th and Constitution Avenue, NW., Room 4830, Washington, D.C. 20230.

AGENDA:

Opening remarks and comments on World Trade Week
Task Force Reports on:
U.S. Competitiveness and Technological Development
Foreign Market Opportunities and the Foreign Commercial Service
Export Goals and Domestic Promotion Programs
Status Report on Export Trading Company Legislation
Formulation of Recommendations for the Full President's Export Council Meeting
Public Comment and Discussion

PUBLIC PARTICIPATION: The meeting will be open for public observation and a limited number of seats will be available. To the extent time permits, members of the public may present oral statements to the Subcommittee. Written statements may be submitted at any time before or after the meeting.

FOR FURTHER INFORMATION OR COPIES OF THE MINUTES CONTACT: Ms. Wendy Haimes, Executive Secretary, President's Export Council, Room 2128, U.S. Department of Commerce, Washington, D.C. 20230; Telephone: (202) 377–1124.

Dated: April 16, 1982.

Henry P. Misisco,

Acting Director.

[FR Doc. 82–10906 Filed 4–20–82; 8:45 am]

BILLING CODE 3510-25-M

Petitions by Producing Firms for Determinations of Eligibility To Apply for Trade Adjustment Assistance

Petitions have been accepted for filing from the following firms: (1) Century Industries, Inc., 765 Conger Street, Eugene, Oregon 97402, producer of stoves, safes and security vaults (accepted March 19, 1982); (2) Pilgrim Fireplace Equipment Company, 720 Harbour Way South, Richmond, California 94804, producer of fireplace accessories (accepted March 25, 1982); (3) The Pesses Company, 29605 Hall Street, Solon, Ohio 44139, producer of ferroalloys (accepted March 26, 1982); (4) LSA Scientific Corporation, 109 Beaver Court, Cockeysville, Maryland 21030, producer of navigation equipment (accepted March 26, 1982); (5) CODI Corporation, 350 Hurst Street, Linden, New Jersey 07036, producer of semiconductors (accepted March 26, 1982); (6) Laudelino Alonzo & Company, Inc., P.O. Box F, Juncos, Puerto Rico 00666–1705, producer of men's and women's slacks (accepted March 29, 1982); (7) Springfield Electrical Specialties, Inc., 18 Furler Street, Totowa, New Jersey 07512, producer of automotive ignition parts (accepted March 30, 1982); (8) Pennshire Shirt Corporation, 10 East 34th Street, New York, New York 10016, producer of men's shirts (accepted March 30, 1982); (9) Sportmaster, Inc., P.O. Box 70, Decatur, Illinois 62521, producer of roller skates (accepted April 1, 1982); (10) Vue-More Manufacturing Corporation, 184 Franklin Avenue, Nutley, New Jersey 07110, producer of merchandising displays and lighting systems (accepted April 1, 1982); (11) Oscar of California, Inc., 3051 South Alameda Street, Los Angeles, California 90058, producer of men's shirts (accepted April 1, 1982); (12) Gale Industries, Inc., P.O. Box 725, Warrensburg, Missouri 64093, producer of headwear (accepted April 2, 1982); (13) Luhr-Jensen and Sons, Inc., P.O. Box 297, Hood River, Oregon 97031, producer of fishing tacle and other sporting goods (accepted April 5, 1982); and (14) Morrow Electronics, Inc., 4740 Ridge Drive, N.E., Salem Oregon 97303, producer of electronic navigation and communication equipment (accepted April 5, 1982).

The petitions were submitted pursuant to Sections 251 of the Trade Act of 1974 (Pub. L. 93–618) and Section 315.23 of the Trade Adjustment Assistance Regulations for Firms and Communities (13 CFR Part 315). Consequently, the United States Department of Commerce has initiated separate investigations to determine whether increased imports into the United States of articles like or directly competitive with those produced by each firm contributed importantly to total or partial separation of the firm's workers, or threat thereof, and to a decrease in sales or production of each petitioning firm.

Any party having a substantial interest in the proceedings may request a public hearing on the matter. A request for a hearing must be received by the Director, Cerification Division, Office of Trade Adjustment Assistance, International Trade Administration, U.S. Department of Commerce, Washington D.C. 20230, no later than the close of business of the tenth calendar day following the publication of this notice.

The Catalog of Federal Domestic Assistance official program number and title of the program under which these petitions are submitted is 11.309, Trade Adustment Assistance. Inasfar as this notice involves petitons for determination of eligibility under the Trade Act of 1974, the requirements of Office of Management and Budget Circular No. A-95 regarding review by clearinghouse do not apply.

Jack W. Osburn, Jr.,

Director, Certification Division, Office of Trade Adjustment Assistance.

[FR Doc. 82–10905 Filed 4–20–82; 8:45 am]

BILLING CODE 3510-25-M

Renewal of Advisory Committees

AGENCY: International Trade Administration, Commerce.

SUMMARY: Subsection 135(c) of the Trade Act of 1974, 19 U.S.C. 2155; as amended by the Trade Agreements Act of 1979, Pub. L. 96–39, gives the President authority to establish advisory committees to provide general policy advice on trade. This authority has been delegated to the United States Trade Representative (the USTR), acting in conjunction with the Secretary of Commerce (the Secretary), according to Executive Order 11846 of March 27, 1975. The USTR and the Secretary renewed the advisory committees listed below which were established on March 21, 1980. This action was taken in accordance with the provisions of the Federal Advisory Committee Act (5 U.S.C. App. (1976)) and OMB Circular A–63 (revised) of March 1974.

Industry Policy Advisory Committee for Trade Policy Matters
Committee of Chairmen of Industry Advisory Committees for Trade Policy Matters
Industry Functional Advisory Committee on Customs Matters for Trade Policy Matters
Industry Functional Advisory Committee on Standards for Trade Policy Matters
Industry Sector Advisory Committees for Trade Policy Matters:
(ISAC 1)—Aerospace Equipment
(ISAC 2)—Capital Goods (e.g., turbines, generators, lifting equipment, industrial machinery, electric transmission equipment, other electrical equipment)
(ISAC 3)—Chemicals and Allied Products (e.g., chemicals and related products except footwear)—4
(ISAC 4)—Consumer Goods (e.g., food and kindred products, tobacco manufactures, household furniture and appliances, published materials, kitchenware)
(ISAC 5)—Electronics and Instrumentation

(ISAC 6)—Energy (e.g., coal mining, oil and gas extraction, refining, pipelines, electric services, gas production and distribution, utility services)
(ISAC 7)—Ferrous Ores and Metals
(ISAC 8)—Footwear, Leather, and Leather Products
(ISAC 9)—Industrial and Construction Material and Supplies
(ISAC 10)—Lumber and Wood Products
(ISAC 11)—Nonferrous Ores and Metals
(ISAC 12)—Paper and Paper Products
(ISAC 13)—Services (e.g., air, water, ground and rail transportation, banking, insurance, hotels, legal services, engineering, construction)
(ISAC 14)—Small and Minority Business
(ISAC 15)—Textiles and Apparel
(ISAC 16)—Transportation, Construction, and Agricultural Equipment
(ISAC 17)—Wholesaling and Retailing

The committees provide technical and policy advice and information to the USTR and the Secretary on trade negotiations, including factors relevant to U.S. positions in such negotiations, and on other matters arising in connection with the operation of trade agreements and the administration of U.S. trade policy. Members of each committee are appointed by and serve at the discretion of the USTR and the Secretary. Each committee functions solely as an advisory body in compliance with the provisions of the Federal Advisory Committee Act. The International Trade Administration (ITA) of the Department of Commerce provides staff support.

Copies of the committees' revised charters were filed with appropriate committees of the Congress and a copy forwarded to the Library of Congress.

EFFECTIVE DATE: Committees were renewed March 11, 1982.

MEMBERSHIP: Representatives from industry or industry associations wishing to be considered for appointment to serve on these committees are requested to make application in writing to the Trade Advisory Center, Room 3036, U.S. Department of Commerce, International Trade Administration, Washington, D.C. 20230.

FOR FURTHER INFORMATION CONTACT: Helen Burroughs or Clare Soponis of the Trade Advisory Center, telephone (202) 377-3268.

Dated: April 15, 1982.

Stephen B. Strauss,

Acting Deputy Assistant Secretary for Trade Information and Analysis.

[FR Doc. 82-10904 Filed 4-20-82; 8:45 am]

BILLING CODE 3510-25-M

The Johns Hopkins University; Notice of Decision on Application for Duty-Free Entry of Scientific Article

The following is a decision on an application for duty-free entry of a scientific article pursuant to Section 6(c) of the Educational, Scientific, and Cultural Materials Importation Act of 1966 (Pub. L. 89-651, 80 Stat. 897) and the regulations issued thereunder as amended (15 CFR Part 301).

A copy of the record pertaining to this decision is available for public review between 8:30 a.m. and 5:00 p.m. in Room 2097 of the Department of Commerce Building, 14th and Constitution Avenue, NW., Washington, D.C. 20230.

Docket No.: 81-00382. Applicant: The Johns Hopkins University, Charles and 34th Streets, Baltimore, MD 21218. Article: Micromanipulator. Manufacturer: Micro Instruments, Ltd., United Kingdom. Intended use of article: See Notice on page 51627 in the **Federal Register** of October 21, 1981.

Comments: No comments have been received with respect to this application. Decision: Application approved. No instrument or apparatus of equivalent scientific value to the foreign article, for such purposes as this article is intended to be used, is being manufactured in the United States. Reasons: This application is a resubmission of Docket No. 81-00011 which was denied without prejudice to resubmission on June 24, 1981 for informational deficiencies. The foreign article provides a single control for all movements and fineness of movement plus a choice of coarse or fine movement. The Department of Health and Human Services advises in its memorandum dated January 19, 1982 that (1) the capabilities of the foreign article described above are pertinent to the applicant's intended purpose and (2) it knows of no domestic instrument or apparatus of equivalent scientific value to the foreign article for the applicant's intended use.

The Department of Commerce knows of no other instrument or apparatus of equivalent scientific value to the foreign article, for such purposes as this article is intended to be used, which is being manufactured in the United States.

(Catalog of Federal Domestic Assistance Program No. 11.105, Importation of Duty-Free Educational and Scientific Materials)

Frank W. Creel,

Acting Director, Statutory Import Programs Staff.

[FR Doc. 82-10907 Filed 4-20-82; 8:45 am]

BILLING CODE 3510-25-M

University of Louisville; Notice of Decision on Application for Duty-Free Entry of Scientific Article

The following is a decision on an application for duty-free entry of a scientific article pursuant to Section 6(c) of the Educational, Scientific, and Cultural Materials Importation Act of 1966 (Pub. L. 89-651, 80 Stat. 897) and the regulations issued thereunder as amended (15 CFR Part 301).

A copy of the record pertaining to this decision is available for public review between 8:30 a.m. and 5:00 p.m. in Room 2097 of the Department of Commerce Building, 14th and Constitution Avenue, NW., Washington, D.C. 20230.

Docket No.: 81-00246. Applicant: University of Louisville, c/o University Counsel, Louisville, Kentucky 40292. Article: Therac 20/Saturne Linear Accelerator. Manufacturer: Atomic Energy of Canada, Ltd., Canada. Intended use of article: See Notice on page 37302 in the **Federal Register** of July 20, 1981.

Comments: Comments dated July 28, 1981 have been received from Varian with respect to this application. Decision: Application denied. Reasons: An instrument or apparatus of equivalent scientific value to the foreign article, for such purposes as this article is intended to be used, is being manufactured in the United States. This application is a resubmission of Docket Number 78-00321 and 79-00231 which were denied without prejudice to resubmission on December 21, 1978 and December 21, 1979, respectively, for informational deficiencies. Discussion: In response to Question 8 of the application form, the applicant admits that there was a domestic instrument (the Varian Clinac 35) of equivalent scientific value to the foreign article at the time the article was ordered (April 26, 1976), but states that (1) the Clinac 35 could not be accommodated in the available space (24'×15' with 4' thick concrete control walls) because of shielding requirements, present architectural configuration, and real estate available, and (2) the Clinac 35 electron beam employs a beam flattener to achieve a uniform treatment field, whereas the foreign article uses an electron scanning roster for this

purpose, thus minimizing x-ray contamination of electron beams at higher levels and permitting safe operation within the shielding configuration available. Section 301.2(m) of the regulations defines "most closely comparable instrument" as the domestic instrument most closely fulfilling the applicant's technical requirements "without regard to differences in cost, design or structural characteristics." Section 301.2(n) defines "pertinent specifications" as those characteristics "necessary for the accomplishment of the (applicant's) purposes," excluding such characteristics as "size, durability, complexity, ease of operation, ease of maintenance and versatility (and) * * * cost differences between the domestic and foreign instrument." The applicant has failed to justify duty-free treatment for this foreign article on grounds allowable under the provisions. We find that the factors cited by the applicant, however important they may have been in its purchasing decision, are directly or indirectly cost-related.

Based on the foregoing consideration as well as on other factual information in our possession (specifications, textbooks, etc.), we find that the Varian Model Clinac 35 was of equivalent scientific value to the foreign article, at the time the foreign article was ordered, for such purposes as this article is intended to be used.

(Catalog of Federal Domestic Assistance Program No. 11.105, Importation of Duty-Free Educational and Scientific Materials)

Frank W. Creel,

Acting Director, Statutory Import Programs Staff.

[FR Doc. 82-10909 Filed 4-20-82; 8:45 am]

BILLING CODE 3510-25-M

Resumption of Monitoring Stainless Steel Round Wire and Round Stainless Steel Drawn Bar Imports; Correction

AGENCY: International Trade Administration, Commerce.

ACTION: This document corrects a document that was published April 20, 1982 at titled, "Resumption of Monitoring of Round Stainless Steel Wire Imports."

SUMMARY: The first sentence of the summary is corrected to read: "This notice is to advise the public that the Department of Commerce will resume monitoring imports of stainless steel round wire and round stainless steel drawn bars in sizes under 0.703" diameter." The third sentence is corrected to read: "The Department will monitor imports of stainless steel round wire and round stainless steel drawn bars in sizes under 0.703" diameter that are exported after April 14, 1982 and are not subject to a binding fixed price contract entered into before April 14, 1982, or that are exported after May 29, 1982.

The seventh sentence of the Supplementary Information is corrected to read: "The Department will resume trigger price monitoring of imports of stainless steel round wire and round stainless steel drawn bars in sizes under 0.703" diameter that are exported after April 14, 1982 and are not subject to a binding fixed price contract entered into before April 14, 1982, or that are exported to the United States after May 29, 1982." On page 16820, column 3, line 28, insert" and round stainless steel drawn bars" after the words "procedures for stainless steel round wire." On page 16820, column 3, line 31, insert "and round stainless steel drawn bars" after the words "stainless steel round wire." On page 16820, column 3, line 34, insert "and 12-6" after the words "pages 16-20 to 16-30." On page 16820, column 3, line 38, delete "(TSUSA 609.4510 and 609.4540)." After the series of tables ending on page 16823, Column 1 add a new table, "Round Stainless Steel *Drawn* Bar in Sizes Under 0.703" Diameter" as follows:

Page 12-6, Rev. Nov. 1981

ROUND STAINLESS STEEL DRAWN BARS IN SIZES UNDER 0.703₆₆ DIAMETER, AISI CATEGORY 12

[Base prices per metric ton—use pages 16-20 to 16-23]

Charges to CIF	Ocean freight	Handling	Interest (percent)
Pacific Coast	$102	$9	3.6
Gulf Coast	124	5	4.6
Atlantic Coast	124	4	4.6
Great Lakes	161	4	5.7

Interest charge equals F.O.B. trigger base price including size extra times interest factor.

Insurance 1% of base price + extras + ocean freight.

Extras ($/M.T.): Use pages 16-20 to 16-30.

Note.—This coverage applies to stainless steel bar produced by drawing. Bar, in these sizes, if produced by hot rolling is not covered by published prices.

F. Lynn Holec,

Director, Agreements Compliance Division, ITA.

[FR Doc. 82-11074 Filed 4-20-82; 8:45 am]

BILLING CODE 3510-25-M

Minority Business Development Agency

Financial Assistance Application Announcement; New York Region Project; Ponce, Puerto Rico SMSA

AGENCY: Minority Business Development Agency, Commerce.

ACTION: Notice.

SUMMARY: The Minority Business Development Agency (MBDA) announces that it is soliciting applications for one New York Region project as follows:

1. One cooperative agreement under its Business Development Center (BDC) program to operate a pilot project for a 12-month period beginning August 1, 1982 in the Ponce, Puerto Rico SMSA. The cost of the project is estimated to be $225,000. The maximum federal participation amount is $225,000. The minimum amount required for non-federal participation is $25,000. The project number is 02-10-82008-01.

Applicants shall be required to contribute at least 10% of the total program costs through non-federal funds. Cost sharing contributions can be in the form of cash contributions, fee for services or in-kind contributions.

CLOSING DATE: May 21, 1982; OMB No. 0640-0006.

ADDRESS: New York Regional Office, Minority Business Development Agency, U.S. Department of Commerce, 26 Federal Plaza, Room 36-116, New York, New York 10278

FOR FURTHER INFORMATION CONTACT: R. Allen Walls, Chief, Enterprise Development; telephone (212) 264-4742.

SUPPLEMENTARY INFORMATION:

A. Scope and Purpose of this Announcement

Executive Order 11625 authorizes MBDA to fund projects which will provide technical and management assistance to eligible clients in areas related to the establishment and operation of businesses. The BDC program is specifically designed to assist those minority businesses that have the highest potential for success. In order to accomplish this, MBDA offers Cooperative Agreements that can: coordinate and broker public and private sector resources on behalf of minority individuals and firms; offer them a full range of management and technical assistance; and serve as a conduit through which and from which information and assistance to and about minority businesses are funneled.

B. Eligible Applicants

Awards shall be open to all individuals, non-profit organizations, for-profit firms, local and state governments, American Indian tribes and educational institutions.

C. Evaluation Process

All proposals received as a result of this announcement will be evaluated by a MBDA review panel.

D. Evaluation Criteria for Business Development Center Application.

The evaluation criteria is designed to facilitate an objective evaluation of competive applications for the Business Development Center program.

MBDA reserves the right to reject any or all applications, including the application receiving the highest evaluation, and will exercise this right when it is determined that it is in the best interest of the Government to do so (e.g., the apparent successful applicant has serious unresolved audit issues from current or previous grants, contracts or cooperative agreements with an agency of the Federal Government).

Evaluation of proposals will employ the following criteria:

I. *Capability and Experience of Firm/ Staff.*—Provide information that demonstrates the organization's capabilities and prior experiences in addressing the needs of minority business individuals and firms. Provide information that demonstrates the staff's capabilities and prior experiences in providing management and technical assistance to minority individuals and firms. Indicate previous experience in MBE community to be served in terms of: inventorying resources and opportunities; the brokering thereof; and providing management and technical assistance.

The following are key factors to be considered in this section:

Firm:

—The organization's receptivity in the MBE community to be served, i.e., business contacts in the public and private sector; leadership responsibilities; and experience in assisting MBE business persons and firms. (references from clients assisted are pertinent.)

—Background credentials and references for the owners of the organization and a capability statement of what the organization can do.

—Knowledge of the geographic area to be served in terms of the needs of minority businesses and past ongoing relationships with local public and private entities—that can possibly enhance the BDC program effort—i.e., Chambers of Commerce, trade associations, venture capital organizations, banks, SBA, HUD, state, city and county government agencies, etc.

Staff:

—List personnel to be used. Indicate their salaries, educational level and previous experiences. Provide resumes for all professional staff personnel.

—Demonstrate competence among staff to effectuate mergers, acquisitions, spin-offs and joint-ventures.

—Provide organization chart, job descriptions and qualification standards involving all professional staff persons to be utilized on the project.

—If any contractors are to be utilized, identify and indicate areas and level of experience. *Primary consideration will be given to inhouse capability.*

Note.—All contracting proposed should be in accordance with procurement standards in Attachment O of OMB Circulars A-110 or A-102.

II. *Techniques and Methodology.*—specify plans for achieving the goals and objectives of the project. This section should be developed by using the outline of the Work Requirements and the BDC responsibilities as *guides* and will become part of the award document. Include start-up plan and example of work plan format. Fully explain the procedures for: outreach, screening, assisting and monitoring clients; developing and maintaining the profile inventory of minority business; and brokering of new business ownership, market and capital opportunities. In summary, address how, when and where work will be done and by whom. Include level of performance.

III. *Resources.*—address technical and administrative resources, i.e. computer facilities, voluntary staff time and space; and financial resources in terms of meeting MBDA's 10% cost sharing requirement to include a fee for services for assistance provided clients. The fee for services will be 10% for firms with gross sales of $500,000 or less and 25% for firms with gross sales of over $500,000.

Cost sharing is that portion of project costs not borne by the Federal Government. The composition and amount of cost sharing are key factors that will be considered in determining the merit of this section. The cost sharing requirement can be met through the following order of priority: 1. cash contributions; 2. fee for services; and 3. in-kind contributions.

A. *Cash contribution.*—means cash that is contributed or donated by the recipient, by other non-federal, public agencies and institutions, private organizations, corporations and individuals.

B. *Fee for services.*—are charges to the client for assistance provided by BDC.

C. *In-kind contribution.*—represent the value of non-cash contributions provided by the recipient and non-federal parties. The order of priority for in-kind contributions are: high technology systems to be utilized to achieve program objectives; top level staff personnel and real and personal property donated by other public agencies, institutions and private organizations. Property purchased with Federal funds will not be considered as the recipient's in-kind contribution.

IV. *Costs.*—demonstrate in narrative format that costs being proposed will give the minority business client and the government the most effective program possible in terms of quality, quantity, timeliness and efficiency.

Include the principal costs involved for achieving work plan under Cooperative Agreement by completing Part III—the Budget Information Section of the Request for Application.

Provide cost sharing plan information in terms of methodology and format for billing the cost of management and technical assistance to clients.

Total project costs will be evaluated in terms of:

—Clear explanations of all expenditures proposed, and

—The extent to which the applicant can leverage federal program funds and operate with *economy* and *efficiency.*

In conclusion, the applicant's schedule for start of BDC operation should be included in Part Two. Part Two will be known as the applicant's plan of operation and will be incorporated into the Cooperative Agreement award.

A detailed justification for all proposed costs is required for Part Four and each item must be fully explained.

The failure to supply information in any given category of the criteria will result in the application being considered nonresponsive and consequently, dropped from competition.

All information submitted is subject to verification by MBDA.

E. Disposition of Proposals

Notification of awards will be made by the Grants Officer. Organizations whose proposals are unsuccessful will be advised by the Regional Director.

F. Proposal Instructions and Forms

Questions concerning the preceding information, copies of application forms, and applicable regulations can be obtained at the above address.

Nothing in this solicitation shall be construed as committing MBDA to divide available funds among all qualified applicants. The program is subject to OMB Circular A-95 requirements.

G. A Pre-Application Conference

To assist all interested applicants who will be held at the above address on May 5, 1982, at 2:00 p.m. in Room 36-116.

(Catalog of Federal Domestic Assistance 11:800 Minority Business Developmen)t

Dated: April 13, 1982.

Ralph J. Pérez,

Regional Director.

[FR Doc. 82-10830 Filed 4-20-82; 8:45 am]

BILLING CODE 3510-21-M

National Bureau of Standards

National Voluntary Laboratory Accreditation Program (NVLAP); Carpet Testing, Public Workshop

AGENCY: National Bureau of Standards, Commerce.

ACTION: Notice of public workshop to discuss the technical aspects of the laboratory accreditation program for carpet testing laboratories.

SUMMARY: The National Bureau of Standards (NBS), in cooperation with the Carpet and Rug Institute (CRI), hereby announces that an informal public workshop will be held to provide interested parties an opportunity to discuss the technical aspects of the carpet laboratory accreditation program (Carpet LAP).

DATE: The workshop will be held on May 26, 1982, from 10:00 a.m. to 4:00 p.m.

ADDRESS: The workshop will take place in Atlanta, Georgia at the Marriott Perimeter Center Hotel, 246 Perimeter Center Parkway, NE (located at the intersection of I-75 and I-285), telephone: (404) 394-6500.

FOR FURTHER INFORMATION CONTACT:

1. Diana Kirkpatrick, NVLAP Project Leader, National Bureau of Standards, Technology Bldg., Room B06, Washington, DC 20234, telephone: (301) 921-2427, or

2. Barry Torrence, Director of Technical Services, The Carpet and Rug Institute, P.O. Box 2048, Dalton, GA 30720, telephone: (404) 278-3176.

Persons who wish to attend the workshop should inform Dr. Kirkpatrick or Mr. Torrence not later than May 21, 1982.

SUPPLEMENTARY INFORMATION: The Carpet LAP was formally established in 1980 in response to a request from the Department of Housing and Urban Development (HUD) under the Procedures of the National Voluntary Laboratory Accreditation Program (NVLAP), 15 CFR Part 7b.

Since this LAP was established, various questions about test procedures have arisen and changes have occurred in the test methods. Issues to be discussed at this informal workshop include, but are not limited to:

1. Changes in the test methods currently in the LAP.
2. Interest in adding test methods to the LAP.
3. The current proficiency testing requirements.
4. Sources of assessors.
5. Suggestions from improving the program.

Representatives from accredited laboratories and from laboratories which may seek accreditation in the future for testing carpet are particularly encouraged to attend. The workshop will be hosted by CRI. Those who represent organizations which are not members of CRI will be asked to pay a $15 registration fee. This fee, payable at the door, is to defray the specific costs associated with their attendance.

Dated: April 16, 1982.

Ernest Ambler,

Director, National Bureau of Standards.

[FR Doc. 82-10807 Filed 4-20-82; 8:45 am]

BILLING CODE 3510-13-M

National Oceanic and Atmospheric Administration

New England Fishery Management Council's Scientific and Statistical Committee; Meeting Amendment

AGENCY: National Oceanic and Atmospheric Administration, Commerce.

ACTION: Notice of change in meeting date for the New England Fishery Management Council's Scientific and Statistical Committee.

SUMMARY: The scheduled public meeting of the New England Fishery Management Council's Scientific and Statistical Committee as published in the **Federal Register**, March 25, 1982, (47 FR 12842), has been changed as follows:

From—Convening on Monday, April 19, 1982, at approximately 10 a.m., and adjourning at approximately 5 p.m.

To—Convening on Monday, April 26, 1982, at approximately 10 a.m., and adjourning at approximately 5 p.m.

All other information remains unchanged.

FOR FURTHER INFORMATION CONTACT: New England Fishery Management Council, Suntaug Office Park, Five Broadway, Route One, Saugus, Massachusetts 01906; Telephone: (617) 231-0422.

Dated: April 16, 1982.

Jack L. Falls,

Chief, Administrative Support Staff, National Marine Fisheries Service.

[FR Doc. 82-10927 Filed 4-20-82; 8:45 am]

BILLING CODE 3510-22-M

COMMITTEE FOR THE IMPLEMENTATION OF TEXTILE AGREEMENTS

Summarizing Provisions of the Visa and Exempt Certification Arrangement Concerning Certain Cotton, Wool and Man-Made Fiber Textile and Apparel Products Produced or Manufactured in the Republic of Korea

April 14, 1982.

On May 25, 1972, a letter dated May 19, 1972 from the Chairman of the Committee for the Implementation of Textile Agreements to the Commissioner of Customs was published in the Federal Register (37 FR 10605), which established an export visa requirement for certain specified textile and apparel products, produced or manufactured in the Republic of Korea and exported to the United States, pursuant to the bilateral cotton, wool and man-made fiber textile agreement between the two governments. During the ensuing years, the original visa arrangement has been amended numerous times to include, among other things, a certification to exempt certain products from the ceilings of the agreement.

By an exchange of letters dated October 29, 1981 and January 18, 1982, the Governments of the United States and the Republic of Korea have agreed to consolidate the existing provisions of the visa system into a single document in an effort to clarify and facilitate implementation of the current requirements. A summary of those requirements follows this notice along with facsimiles of the authorized visa and exempt certification stamps.

Under the terms of the Bilateral Cotton, Wool and Man-Made Fiber Textile Agreement between the Governments of the United States and the Republic of Korea, the Chairman of

the Committee for the Implementation of Textile Agreements has directed the Commissioner of Customs, effective on June 24, 1972 and until further notice, to prohibit entry, or withdrawal from warehouse, for consumption in the United States of cotton, wool and man-made fiber textile products in Categories 300–369, 400–469 and 600–669, produced or manufactured in the Republic of Korea, for which the Government of the Republic of Korea has not issued an appropriate export visa. The only exception is merchandise which is accompanied by a properly executed exempt certification.

Down and feather-filled jackets, coats and vests in Categories 353, 354, 653 and 654 and shipments for the non-commercial, personal use of the importer, regardless of value, are exempt from both the export visa and exempt certification requirements.

The visa is an original, oval-shaped, stamped marking on the front of the invoice (Special Customs Invoice Form 5515, successor document, or commercial invoice when such form is used). The visa number and date of issuance are to be indicated on the stamp, as well as the correct category number, the quantity, unit of measure and the signature of the official authorized to issue visas. Although the visa stamp must be an original, the stamp may be on a copy of the invoice.

The category on the export visa must agree with that determined by the U.S. Customs Service, or entry will be denied, except that merchandise in the following categories may be permitted entry if the accompanying visa includes the combination of categories, or one or more of the constituent categories in the combination:

333/334/335	433/434	638/669
338/339	445/446	643/644
347/348	633/634/635	645/646

In the case of man-made fiber woven shirts in Category 640, the export visa must show the correct category plus the letter "D" or "O", as follows:

640–D (only T.S.U.S.A. numbers 379.3130, 379.3334, 379.9535, 379.9540 and 379.9639)

640–O (all T.S.U.S.A. nmbers in Category 640 except 379.3130, 379.3334, 379.9535, 379.9540 and 379.9639)

Absence of the correct letter identifier will result in denial of entry.

The foregoing provisions notwithstanding, if the quantity indicated on the export visa is more than that of the shipment, entry will be permitted.

Merchandise which is exempt from the quantitative levels of the bilateral agreement with the Republic of Korea is to be certified for exemption by the Government of the Republic of Korea prior to exportation. The basis for exemption will be stated on the certification by the use of a description, such as, "$250 or less", or "Taekwondo and Judosuits", or will include the name of a particular traditional folklore/handicraft textile product identified in the agreement, or subsequently added by mutual agreement between the two governments. At this time the following products have been specifically identified for exemption:

1. Chima—The long, formless and ample skirt portion of the traditional Korean Chima-Chogori dress set.
2. Chogori—The short, halter-type blouse or top portion of the traditional Korean Chima-Chogori dress set.
3. Bosun—An ankle boot-type article, wholly of cloth, worn by Korean women indoors.
4. Fabrics not exceeding 24 x 48 inches in size, containing hand-embroidered or handpainted Korean scenes.
5. Handmade carpets in which the pile was inserted or knotted by hand and classified by the U.S. Customs Service in T.S.U.S.A. numbers 360.0600, 360.7800, 360.1050 and 360.1550.
6. Judo and Karate and Taekwondo Uniforms classified by the U.S. Customs Service in T.S.U.S.A. numbers 379.0830, 379.3330, 379.6300, 379.9635, 383.0850 and 383.2345.
7. Korean-style handbags and other flat goods of the type considered by the U.S. Customs Service to be classified as luggage: women's and children's handbags and billfolds, card cases, coin purses, eyeglass cases and similar flat goods.

Merchandise covered by an invoice which has an exempt certification but contains both exempt and non-exempt items will be denied entry by the U.S. Customs Service.

At this time Jang Wooh Noh (Noh, J. W.) is the only official authorized by the Government of the Republic of Korea to sign export visas and certifications for exemption.

Any changes in the foregoing arrangements which may be effected in the future by agreement between the Governments of the United States and the Republic of Korea will be published in the **Federal Register**.

Paul T. O'Day,

Chairman, Committee for the Implementation of Textile Agreements.

BILLING CODE 3510-25-M

DEPARTMENT OF THE TREASURY
UNITED STATES CUSTOMS SERVICE
19 U.S.C. 1481, 1482, 1484

SPECIAL CUSTOMS INVOICE

(Use separate invoice for purchased and non-purchased goods.)

Form Approved.
O.M.B. No. 48-RO342

1. SELLER

2. DOCUMENT NR.*

3. INVOICE NR. AND DATE*

4 REFERENCES *

5. CONSIGNEE

6. BUYER *(if other than consignee)*

7. ORIGIN OF GOODS

8. NOTIFY PARTY *

9. TERMS OF SALE, PAYMENT, AND DISCOUNT

MINISTRY OF COMMERCE & INDUSTRY
TEXTILE EXPORT VISA
NO ______ DATE OF
CAT. NO ______ ISSUE ______
QUANTITY ______ (UNIT:)
[illegible] JANG WOO [illegible]
DIRECTOR, EXPORT DIVISION I
REPUBLIC OF KOREA

10. ADDITIONAL TRANSPORTATION INFORMATION*

11. CURRENCY USED

12. [illegible]

13. DATE ORDER ACCEPTED

14. MARKS AND NUMBERS ON SHIPPING PACKAGES	15. NUMBER OF PACKAGES	16. FULL DESCRIPTION OF GOODS	17. QUANTITY	UNIT PRICE 18. HOME MARKET	UNIT PRICE 19. INVOICE	20. INVOICE TOTALS
		SAMPLE				

21. ☐ If the production of these goods involved furnishing goods or services to the seller *(e.g., assists such as dies, molds, tools, engineering work)* and the value is not included in the invoice price, check box (21) and explain below.

22. PACKING COSTS

27. DECLARATION OF SELLER/SHIPPER (OR AGENT)

I declare:

(A) ☐ If there are any rebates, drawbacks or bounties allowed upon the exportation of goods, I have checked box (A) and itemized separately below.

(B) ☐ If the goods were not sold or agreed to be sold, I have checked box (B) and have indicated in column 19 the price I would be willing to receive.

I further declare that there is no other invoice differing from this one (unless otherwise described below) and that all statements contained in this invoice and declaration are true and correct.

(C) SIGNATURE OF SELLER/SHIPPER (OR AGENT):

23. OCEAN OR INTERNATIONAL FREIGHT

24. DOMESTIC FREIGHT CHARGES

25. INSURANCE COSTS

26. OTHER COSTS *(Specify Below)*

28. THIS SPACE FOR CONTINUING ANSWERS

THIS FORM OF INVOICE REQUIRED GENERALLY IF RATE OF DUTY BASED UPON OR REGULATED BY VALUE OF GOODS AND PURCHASE PRICE OR VALUE OF SHIPMENT EXCEEDS $500. OTHERWISE USE COMMERCIAL INVOICE

*Not necessary for U.S. Customs purposes.

Customs Form 5515 (12-20-76)

DEPARTMENT OF THE TREASURY
UNITED STATES CUSTOMS SERVICE
19 U.S.C. 1481, 1482, 1484

SPECIAL CUSTOMS INVOICE

(Use separate invoice for purchased and non-purchased goods.)

Form Approved.
O.M.B. No. 48-RO342

1. SELLER

2. DOCUMENT NR.*

3. INVOICE NR. AND DATE*

4. REFERENCES*

5. CONSIGNEE

6. BUYER (If other than consignee)

7. ORIGIN OF GOODS

8. NOTIFY PARTY*

9. TERMS OF SALE, PAYMENT, AND DISCOUNT

10. ADDITIONAL TRANSPORTATION INFORMATION*

11. CURRENCY USED

12. EXCH. RATE (If fixed or agreed)

13. DATE ORDER ACCEPTED

MINISTRY OF COMMERCE AND INDUSTRY
KOREAN ITEM
DATE:
ITEM:
QUANTITY:
NON-QUOTAITEM
No.
NOH JANG WOON
DIRECTOR

14. MARKS AND NUMBERS ON SHIPPING PACKAGES	15. NUMBER OF PACKAGES	16. FULL DESCRIPTION OF GOODS	17. QUANTITY	UNIT PRICE 18. HOME MARKET	UNIT PRICE 19. INVOICE	20. INVOICE TOTALS
		SAMPLE				

21. ☐ If the production of these goods involved furnishing goods or services to the seller (*e.g., assists such as dies, molds, tools, engineering work*) and the value is not included in the invoice price, check box (21) and explain below.

22. PACKING COSTS

27. DECLARATION OF SELLER/SHIPPER (OR AGENT)

23. OCEAN OR INTERNATIONAL FREIGHT

I declare:

(A) ☐ If there are any rebates, drawbacks or bounties allowed upon the exportation of goods, I have checked box (A) and itemized separately below.

(B) ☐ If the goods were not sold or agreed to be sold, I have checked box (B) and have indicated in column 19 the price I would be willing to receive.

24. DOMESTIC FREIGHT CHARGES

25. INSURANCE COSTS

I further declare that there is no other invoice differing from this one (unless otherwise described below) and that all statements contained in this invoice and declaration are true and correct.

(C) SIGNATURE OF SELLER/SHIPPER (OR AGENT):

26. OTHER COSTS (Specify Below)

28. THIS SPACE FOR CONTINUING ANSWERS

THIS FORM OF INVOICE REQUIRED GENERALLY IF RATE OF DUTY BASED UPON OR REGULATED BY VALUE OF GOODS AND PURCHASE PRICE OR VALUE OF SHIPMENT EXCEEDS $500. OTHERWISE USE COMMERCIAL INVOICE.

*Not necessary for U.S. Customs purposes.

Customs Form 5515 (12-20-76)

[FR Doc. 82-10908 Filed 4-20-82; 8:45 am]

BILLING CODE 3510-25-C

DEPARTMENT OF DEFENSE

Department of the Army

Medical Research and Development Advisory Committee, Medical Defense Against Chemical Agents Subcommittee; Partially Closed Meeting

In accordance with Section 10(a)(2) of the Federal Advisory Committee Act (Pub. L. 92–463), announcement is made of the following Subcommittee meeting:

Name of Committee: United States Army Medical Research and Development Advisory Committee, Subcommittee on Medical Defense Against Chemical Agents.

Date of Meeting: May 12, 1982.

Time and Place: 0900 hrs, Room 14, US Army Medical Research Institute of Chemical Defense, Aberdeen Proving Ground, MD.

Proposed Agenda: This meeting will be open to the public from 0900 to 1000 hrs for the administrative review and discussion of the scientific research program of the U.S. Army Medical Research Institute of Chemical Defense. Attendance by the public at open sessions will be limited to space available.

In accordance with the provisions set forth in Section 552b(c)(6), United State Code, Title 5 and Section 10(d) of Pub. L. 92–463, the meeting will be closed to the public from 1000 to 1630 hrs for the review, discussion and evaluation of individual programs and projects conducted by the U.S. Army Medical Research and Development Command, including consideration of personnel qualifications and performance, the competence of individual investigators, medical files of individual research subjects, and similar items, the disclosure of which would constitute a clearly unwarranted invasion of personal privacy.

Dr. Richard Lindstrom, U.S. Army Medical Research Institute of Chemical Defense, Aberdeen Proving Ground, MD 21010 (301/671–2833) will furnish summary minutes, roster of Subcommittee members and substantive program information.

Harry G. Dangerfield,

Colonel, MC, Deputy Commander.

[FR Doc. 82–10918 Filed 4–20–82; 8:45 am]

BILLING CODE 3710–08–M

Military Traffic Management Command, Military Personal Property Symposium; Open Meeting

Announcement is made of a meeting of the Military Personal Property Symposium. This meeting will be held on May 13, 1982 at the Quality Inn, Pentagon City, 300 Army Navy Drive, Arlington, VA, and will convene at 0900 hours and adjourn at approximately 1500 hours.

Proposed Agenda: The purpose of the Symposium is to provide an open discussion and free exchange of ideas with the public on procedural changes to the Personal Property Traffic Management Regulation (DOD 4500.34–R), and the handling of other matters of mutual interest relating to the movement and/or storage of household goods and unaccompanied baggage, as well as proposed changes and innovations in the Department of Defense Personal Property Movement and Storage Program.

All interested persons desiring to submit topics to be discussed should submit them in writing to the Commander, Military Traffic Management Command, ATTN: MT–PPM, Washington, DC 20315. Topics to be discussed should be received on or before May 3, 1982.

Dated: April 13, 1982.

Nathan R. Berkley,

Colonel, GS, Director of Personal Property.

[FR Doc. 82–10917 Filed 4–20–82; 8:45 am]

BILLING CODE 3710–08–M

DEPARTMENT OF EDUCATION

Office of Elementary and Secondary Education

School Construction in Areas Affected by Federal Activity; Cutoff Date Notice for the Transmittal of Applications for Fiscal Year 1982

Applications are invited for new projects under the School Construction Program. Authority for this program is contained in Pub. L. 81–815, school construction in areas affected by Federal activities.

(20 U.S.C. 631–647)

Notice is given that the Secretary of Education has established a cutoff date for the transmittal of applications for increase periods ending June 1982 or June 1983 for assistance under Sections 5 and 9 of Pub. L. 81–815. (An increase period is a period of four consecutive regular school years during w iich a school district has experienced a substantial increase in school membership as a result of new or increased Federal activities.) This cutoff date also applies to applications for new projects under Section 14 of Pub. L. 81–815, and to new applications for supplemental assistance under Section 8 of Pub. L. 81–815. (Section 14 authorizes assistance for certain school districts which serve children residing on Indian lands, or which are significantly burdened by the presence of nontaxable Federal property. Section 8 authorizes assistance that supplements certain awards made under Sections 5, 9, and 14 of Pub. L. 81–815.)

Approval of these applications is subject to availability of funds.

Cutoff Date for Transmittal of Applications

Applications must be mailed or hand delivered to the U.S. Secretary of Education from the State educational agencies on or before June 30, 1982.

Applications Delivered by Mail

Applications sent by mail must be addressed to the Secretary of Education, U.S. Department of Education, 400 Maryland Avenue, S.W., Room 2107A, Washington, D.C. 20202.

An applicant must show proof of mailing consisting of one of the following:

(1) A legible dated U.S. Postal Service postmark.

(2) A legible mail receipt with the date of mailing stamped by the U.S. Postal Service.

(3) A dated shipping label, invoice, or receipt from a commercial carrier.

(4) Any other evidence of mailing acceptable to the U.S. Secretary of Education.

If an application is sent through the U.S. Postal Service, the Secretary does not accept either of the following as proof of mailing:

(1) A private metered postmark.

(2) A mail receipt that is not dated by the U.S. Postal Service.

An applicant should note that the U.S. Postal Service does not uniformly provide a dated postmark. Before relying on this method, an applicant should check with its local post office.

An applicant is encouraged to use registered or at least first class mail. Each late applicant for a new project will be notified that its application will not be considered.

Applications Delivered by Hand

An application that is hand delivered must be taken to the U.S. Department of Education, 400 Maryland Avenue, S.W., Room 2107A, Washington, D.C.

Hand delivered applications will be accepted between 8:00 a.m. and 4:30 p.m. (Washington, D.C., time) daily, except Saturdays, Sundays, and Federal holidays. Hand delivered applications will not be accepted after 4:30 p.m. on the cutoffdate.

Program Information

Federal funds are provided for the construction or temporary provision of urgently needed minimum school facilities in school districts which have had substantial increases in school membership as a result of new or increased Federal activities. Funds are also provided for construction of needed minimum school facilities in school districts which serve children residing

on Indian lands, or which are significantly burdened by the presence of nontaxable Federal property.

Application Forms

Application forms and instructions may be obtained from the appropriate State educational agency which serves the applicant local educational agency.

Applications must be prepared and submitted in accordance with the regulations, instructions, and forms included in the program application.

Applicable Regulations

The regulations applicable to this program are: (a) Regulations governing the School Construction Program (34 CFR Part 221) published in the **Federal Register** on April 8, 1975.

Note.—Proposed regulations governing the School Construction Program (34 CFR Part 221) were published in the **Federal Register** on June 29, 1979. When these regulations are published in final, they will supersede all other regulations.

(b) The Education Department General Administrative Regulations (EDGAR).

(34 CFR Parts 75 and 77)

(20 U.S.C. 633)

(Catalog of Federal Domestic Assistance No. 84.040, School Assistance in Federally Affected Areas-Construction

D. Jean Benish,

Acting Assistant Secretary for Elementary and Secondary Education.

[FR Doc. 82-10890 Filed 4-20-82; 8:45 am]

BILLING CODE 4000-01-M

Office of Postsecondary Education

Cooperative Education Program; Application Notice for New Awards for Fiscal Year 1982

Applications are invited for new awards for administration, demonstration, research, and training projects under the Cooperative Education Program. Applications are not invited for exploration projects under this program.

The Cooperative Education Program provides Federal financial assistance to help institutions of higher education offer students paid work experiences closely related to their academic and career pursuits, and to provide for specialized training and research to improve the effectiveness of programs of Cooperative Education.

Authority for this program is contained in Title VIII of the Higher Education Act of 1965, as amended by Pub. L. 96-374.

(20 U.S.C. 1133-1133b)

Closing Date for Transmittal of Applications

Applications for administration, demonstration, research, and training grants must be mailed or hand-delivered by June 7, 1982.

Applications Delivered by Mail

An application sent by mail must be addressed to the U.S. Department of Education, Application Control Center, Attention: 84.055A (for administration projects), or 84.055B (for demonstration projects), 84.055C (for research projects), or 84.055D (for training projects), Washington, D.C. 20202.

An applicant must show proof of mailing consisting of one of the following:

(1) A legibly dated U.S. Postal Service postmark.

(2) A legible mail receipt with the date of mailing stamped by the U.S. Postal Service.

(3) A dated shipping label, invoice, or receipt from a commercial carrier.

(4) Any other proof of mailing acceptable to the U.S. Secretary of Education.

If an application is sent through the U.S. Postal Service, the Secretary does not accept either of the following as proof of mailing: (1) A private metered postmark, or (2) a mail receipt that is not dated by the U.S. Postal Service.

An applicant should note that the U.S. Postal Service does not uniformly provide a dated postmark. Before relying on this method, an applicant should check with its local post office.

An applicant is encouraged to use registered or at least first class mail. Each late applicant will be notified that its application will not be considered.

Applications Delivered by Hand

An application that is hand-delivered must be taken to the U.S. Application Control Center, Room 5673, Regional Office Building 3, 7th and D Streets, S.W., Washington, D.C.

The Application Control Center will accept a hand-delivered application between 8:00 a.m. and 4:30 p.m. (Washington, D.C. time) daily, except Saturdays, Sundays, and Federal holidays.

An application that is hand-delivered will not be accepted after 4:30 p.m. on the closing date.

Program Information

Regulations for the Cooperative Education Program are published in this issue of the **Federal Register**. Applicants should be guided by the provisions or requirements of the regulations in developing their applications.

Applicants are encouraged to be specific in their responses to the operative selection criteria, inasmuch as the Secretary will not give further consideration for funding to any application that receives an average score of 50 points or less in the evaluation process conducted in accordance with 34 CFR 75.217.

Administration Projects

To provide opportunities for a greater number of students to participate in Cooperative Education projects, the Secretary strongly encourages institutions of higher education to apply for funds for more than one eligible unit, as that term is defined in § 631.3 of the regulations.

As provided in the statute, in any fiscal year, an institution of higher education applying for an administration grant individually is eligible for an award of up to $325,000; an institution applying for an administration grant as a member of a consortium is eligible for an award of up to $250,000.

The Secretary will give awards for multi-year projects to successful applicants who have never before received Federal funds to support a program of Cooperative Education. Awards for multi-year projects will be made out of succeeding years' appropriations and in accordance with 34 CFR 75.253. The Secretary will give awards for single-year projects to all other successful applicants.

In awarding administration grants, the Secretary, in accordance with the statute, will place an emphasis on funding institutions of higher education that show the greatest promise of success because of—

(1) The extent to which programs of Cooperative Education in the academic disciplines, with respect to which the application is made, have had a favorable reception by employers; and

(2) The commitment of the institution of higher education to Cooperative Education, as demonstrated by the plans which the institution has made to continue Cooperative Education after the termination of Federal financial assistance.

Demonstration Projects

Applicants may apply for a demonstration grant to conduct a comprehensive Cooperative Education project, as that phrase is defined in § 631.3 of the regulations. Successful applicants may be given a multi-year grant out of the fiscal year 1982 appropriation to cover a project period of up to three years.

Research Projects

The Secretary will award research grants for studies that assess the impact of factors that hinder or enhance student participation, faculty participation, or employer participation in programs of Cooperative Education.

The Secretary will give awards for single-year projects to successful applicants.

Training Projects

The Secretary will make awards for training projects designed to meet the needs of eligible individuals who participate or wish to participate in the planning, establishment, administration, or coordination of Cooperative Education projects conducted by institutions of higher education.

In preparing the application, applicants are encouraged to work jointly with employers in planning training projects.

The Secretary will give awards for single-year or multi-year projects to successful applicants. Awards for multi-year projects will be made out of succeeding years' appropriations and in accordance with 34 CFR 75.253.

Available Funds

There is $14,400,000 appropriated for awards under the Cooperative Education Program for fiscal year 1982. However, the President has proposed budget rescissions to the Congress that may reduce the funds for this program. The deadline in this notice will not be extended, and applicants should prepare and submit applications pending further notification. Applications must be submitted to the Application Control Center at the address in this notice.

Of the $14,400,000 appropriation, approximately $596,000 has been committed for noncompeting continuation grants for 8 administration projects, and approximately $519,000 has been committed for noncompeting continuation grants for 4 training projects.

The $7,404,000 available for administration grants will support approximately 121 projects, with awards averaging $62,000.

The $5,470,000 available for demonstration grants will support 6 to 8 comprehensive Cooperative Education projects, with awards averaging approximately $684,000.

The $130,000 available for research grants will support 4 research projects, with awards averaging $32,500.

The $281,000 available for training grants will support 3 training projects, with awards averaging approximately $93,000.

If the appropriation is reduced, there would be $4,268,000 to support administration projects only. From the available funds, $596,000 will be used to honor commitments made to grantees that received multi-year awards in fiscal year 1981. The remaining $3,672,000 will be used to fund new administration projects. It is anticipated that approximately 58 grants will be awarded, averaging $62,000.

These estimates do not bind the U.S. Department of Education, except as may be required by the applicable statute and regulations.

Application Forms

Application forms and program information packages are expected to be mailed to all eligible institutions of higher education by April 21, 1982. They may also be obtained after April 26, 1982, from the Cooperative Education Branch, U.S. Department of Education (Room 3053, Regional Office Building 3), 7th and D Streets, S.W., Washington, D.C. 20202. Telephone: (202) 245-2146.

Applications must be prepared and submitted in accordance with the regulations, instructions, and forms included in the program information package. The Secretary strongly urges that the narrative portion of the application not exceed 25 pages in length for administration projects, 30 pages for demonstration projects, 15 pages for research projects and 20 pages for training projects. The Secretary further urges that applicants not submit information that is not requested.

Applicable Regulations

Regulations applicable to this program include the following:

(a) Regulations governing the Cooperative Education Program (34 CFR Parts 631, 632, 633, 634, and 635), published in this issue of the **Federal Register**; and

(b) The Education Department General Administrative Regulations (EDGAR) (34 CFR Parts 74, 75, 77 and 78). (These parts were previously codified as 45 CFR Parts 74, 100a, 100c, and 100d respectively).

Further Information

For further information, contact Mr. Morris L. Brown, Chief, Cooperative Education Branch, U.S. Department of Education, Division of Institutional and State Incentive Programs, Office of Institutional Support Programs, (Room 3053, ROB-3), 400 Maryland Avenue, S.W., Washington, D.C. 20202. Telephone: (202) 245-2146.

(20 U.S.C. 1133-1133b)

(Catalog of Federal Domestic Assistance No. 84.055: Cooperative Education Program)

Dated: April 16, 1982.

T. H. Bell,

Secretary of Education.

[FR Doc. 82-10863 Filed 4-20-82; 8:45 am]

BILLING CODE 4000-01-M

DEPARTMENT OF ENERGY

Pricing Policy Change for Sale of Uranium Depleted in Isotope U-235

AGENCY: Energy Department.

ACTION: Notice of pricing policy change for sale of uranium depleted in the isotope U-235.

SUMMARY: Having received no comment on the proposed pricing policy change for sale of uranium depleted in the isotope U-235 (tails) as published in the **Federal Register** on January 7, 1982, (47 FR 846) and (47 FR 847), the Department hereby gives the following notice.

SUPPLEMENTARY INFORMATION: Since this regulation clearly will not have a significant effect on the environment, DOE has determined that C.E.Q. Regulations (40 CFR Part 1501) do not require an environmental impact statement or an environmental assessment.

DOE has determined that initial and final regulatory flexibility analyses required by sections 603 and 604 of the Regulatory Flexibility Act need not be prepared for these regulations since they would not exert a significant economic impact on a substantial number of small entities.

DOE has determined that these regulations are not "major rules" as defined in section 1(b) of Executive Order 12291 because they are not likely to result in an annual effect on the economy of $100 million or more and would not result in a major increase in costs for individual industries.

Uranium Hexaflouride; Base Charges, Use Charges, Special Charges, Table of Enriching Services, Specifications and Packaging: Revisions

The Department of Energy (DOE) hereby announces revisions to the notice entitled, "Uranium Hexaflouride: Base Charges, Use Charges, Special Charges, Table of Enriching Services, Specification and Packaging," as published in the **Federal Register** on November 29, 1967, 32 FR 16289, as amended in 34 FR 2626, February 26, 1969; 34 FR 14039, September 4, 1969; 35 FR 13457, August 25, 1971; 36 FR 4563, March 9, 1971; 36 FR 11877, June 22, 1971; 38 FR 4432, February 14, 1973; 38 FR 13593, May 23, 1973; 38 FR 21518, August 9, 1973; 38 FR 22908, August 27, 1973; 38

FR 27962, October 10, 1973; 39 FR 22182, June 20, 1974; 40 FR 1117, January 16, 1975; 40 FR 17070, April 16, 1975; 40 FR 26060 and 26061, June 20, 1975; 41 FR 8414 and 8415, February 26, 1976; 41 FR 18914 and 18915, May 7, 1976; 41 FR 31942 and 31943, July 30, 1976; and 42 FR 51635, September 29, 1977; (referred to herein as "the Notice").

Delete paragraph 3 of the Notice in its entirety and substitute the following:

3. Standard table of enriching services charges per kilogram unit of separative work, base charges and standard processing loss. (a) DOE's standard table of enriching services is set forth in Table 1 of this Notice. (b) The charges per kilogram unit of separative work furnished by DOE and the base charge ($/kg U) for the sale of government-owned natural uranium will be published by separate notice. (c) The base charge ($/kg U) for uranium enriched in the isotope U–235 and in the form of UF_6 is determined by summing (i) the number opposite the desired assay in the Feed component column of Table 1 multiplied by the then applicable current base charge ($/kg U) for the sale of government-owned natural uranium in the form of UF_6, and (ii) the number opposite the desired assay in the Separative Work Component column of Table 1 multiplied by the current charge per kilogram unit of separative work furnished pursuant to other than requirements-type contracts. The calculated base charge is rounded up to the nearest $0.01. For assays not shown in Table 1, the feed component and separative work component are first determined by linear interpolation before calculation of the base charge. (d) The price for uranium depleted in the isotope U–235 (tails) will be established in consideration of the market value of the material at the time of sale, or on a negotiated basis if market value cannot reasonably be determined. (e) The standard processing loss factor to be applied to toll enricher's acquisition of tails material is 0.05 percent.

Dated: April 14, 1982.

Shelby T. Brewer,

Assistant Secretary for Nuclear Energy.

[FR Doc. 82–10871 Filed 4–20–82; 8:45 am]

BILLING CODE 6450-01-M

Office of Assistant Secretary for International Affairs

Proposed Subsequent Arrangement; European Atomic Energy Community (EURATOM)

Pursuant to section 131 of the Atomic Energy Act of 1954, as amended (42 U.S.C. 2160) notice is hereby given of a proposed "subsequent arrangement" under the Additional Agreement for Cooperation Between the Government and the United States of America and the European Atomic Energy Community (EURATOM) Concerning Civil Uses of Atomic Energy, as amended.

The subsequent arrangement to be carried out under the above mentioned agreement involves U.S. approval for the use of 57.8 kilograms of fissile plutonium recovered from U.S. origin special nuclear material in either the French Super Phenix or the West German Kalkar fast breeder reactors. The plutonium is to be recovered from spent fuel assemblies from the Swiss Muehleberg nuclear power plant, and the spent fuel is now located at the Cogema reprocessing facility at Cap-La-Hague, France.

In accordance with section 131 of the Atomic Energy of 1954, as amended, it has been determined that this subsequent arrangement will not be inimical to the common defense and security.

This subsequent arrangement will take effect no sooner than fifteen days after the date of publication of this notice and after fifteen days of continuous session of the Congress, beginning the day after the date on which the reports required by Section 131 of the Atomic Energy Act of 1954, as amended (42 U.S.C. 2160) are submitted to the Committee on Foreign Affairs of the House of Representatives and the Committee on Foreign Relations of the Senate. The two time periods referred to above shall run concurrently.

For the Department of Energy.

Dated: April 16, 1982.

Harold D. Bengelsdorf,

Director, Office of International Nuclear and Non-Proliferation Policy.

[FR Doc. 82–10808 Filed 4–20–82; 8:45 am]

BILLING CODE 6450-01-M

Office of Defense Programs

Dose Assessment Advisory Group; Open Meeting

Pursuant to the provisions of the Federal Advisory Committee Act (Pub. L. 92–463, 86 Stat. 770), notice is hereby given of the following advisory committee meeting.

Name: Dose Assessment Advisory Group.

Date and Time: Thursday, May 6, 1982—8:30 a.m.–5:00 p.m. Friday, May 7, 1982—8:30–4:00 p.m.

Place: U.S. Department of Energy, Nevada Operations Office Auditorium, 2753 South Highland Drive, Las Vegas, Nevada

Contact: Marshall Page, Jr., Deputy Project Manager, Off-Site Radiation Exposure Review Project, Nevada Operations Office, U.S. Department of Energy, Box 14100, Las Vegas, Nevada 89114, Telephone: 702–734–3181

Purpose of Group

To provide the Secretary of Energy and the Manager, Nevada Operations Office (NV), with advice and recommendations pertaining to the Off-Site Radiation Exposure Review Project (ORERP). This project concerns the evaluation and assessment of the amount of radiation received by members of the offsite population surrounding the Nevada Test Site (NTS) as a result of the nuclear test operations conducted at the NTS.

Tentative Agenda

May 6, 1982

- Welcome and Introduction
- Overview of Work Since Meeting of December 2–3, 1981
- Fallout Verification Report
- Progress Reports:
  —Data Collection
  —Fallout Pattern Analyses
  —Statistical Analyses
  —Pathways Analysis
  —Internal and External Doses
- Time of Arrival Position Paper
- Dose Estimates for 5 Litigants
- Discussion and Comments by DAAG Members
- Public Comments and Questions (5 minute rule)

May 7, 1982

- Environmental Sampling Update
- Strategy for Project Completion
- Introduction to Sheep Discussions
- Sheep Deaths in Utah and Nevada Following the 1953 Nuclear Tests
- Dose Assessment for Sheep Exposed to Fallout From Nuclear Test Nancy
- Preliminary Assessment of Radiation Dose to Sheep Wintering in the Vicinity of the Nevada Test Site in 1953
- Discussion and Recommendations by DAAG to ORERP
- Public Comments and Questions (5 minute rule)

Public Participation

The meeting is open to the public. The Chairperson of the Group is empowered to conduct the meeting in a fashion that will, in his judgment, facilitate the orderly conduct of business. Any member of the public who wishes to file a written statement with the Group will be permitted to do so, either before or after the meeting. Members of the public who wish to make oral statements

pertaining to agenda items should contact Marshall Page at the address or telephone number listed above.

Transcripts

Available for public review and copying at the Public Reading Room, Room 1E-190, Forrestal Building, 1000 Independence Avenue, SW, Washington, DC, between 8:00 a.m. and 4:00 p.m., Monday through Friday, except Federal holidays.

Issued in Washington, DC on April 13, 1982.

Howard H. Raiken,

Deputy Advisory Committee Management Officer.

[FR Doc. 82-11090 Filed 4-20-82; 11:54 am]

BILLING CODE 6450-01-M

Office of the Secretary

Intent to Grant Exclusive Patent License; Atom Sciences, Inc., Oak Ridge, Tenn.

Notice is hereby given of an intent to grant to Atom Sciences, Inc., of Oak Ridge, Tennessee, an exclusive license to manufacture, use, and sell in the United States, the invention described in U.S. Patent No. 3,987,302, entitled "Resonance Ionization For Analytical Spectroscopy." The patent is owned by the United States of America, as represented by the Department of Energy (DOE).

This is a republican of the notice appearing in the Federal Register (47 FR 6362, February 11, 1982), which identifed an incorrect patent number. Accordingly, the prior notice is hereby cancelled.

The proposed license will contain terms and conditions in accordance with 35 U.S.C. 209. DOE intends to grant the license, upon a final determination in accordance with 35 U.S.C. 209(c), unless within 60 days of this notice the Assistant General Counsel for Patents, Department of Energy, Washington, D.C. 20585, receives in writing any of the following, together with supporting documents:

(i) A statement from any person setting forth reasons why it would not be in the best interest of the United States to grant the proposed license, or

(ii) An application for a nonexclusive license to manufacture, use, and/or sell the invention in the United States, in which applicant states that he has already brought the invention to practical application or is likely to bring the invention to practical application expeditiously.

The Assistant General Counsel for Patents will review all written responses to this notice, and will grant the license if, after expiration of the 60-day notice period, and after consideration of written responses to this notice, a determination is made, in accordance with 35 accordance with 35 U.S.C. 209(c), that the license grant is in the public interest.

Signed at Washington, D.C., on this 14th day of April 1982.

Department of Energy.

R. Tenney Johnson,

General Counsel.

[FR Doc. 82-10809 Filed 4-20-82; 8:45 am]

BILLING CODE 6450-01-M

Office of Energy Research

High Energy Physics Advisory Panel; Open Meeting

Pursuant to the provisions of the Federal Advisory Committee Act (Public Law 92-463, 86 Stat. 770), notice is hereby given of the following advisory committee meeting:

Name: High Energy Physics Advisory Panel.

Date and time: Monday, May 10, 1982—9:00 a.m.-6:00 p.m.; Tuesday, May 11, 1982—9:00 a.m.-4:00 p.m.

Place: U.S. Department of Energy, Room A-410, Germantown, MD.

Contact: Dr. P. K. Williams, Secretary, High Energy Physics Advisory Panel, U.S. Department of Energy, Mail Stop J-309, Washington, D.C. 20545, Telephone: 301-353-3367.

Purpose of committee: To provide advice and guidance on a continuing basis with respect to the high energy physics research program.

Tentative agenda:

- Discussion of the status of DOE and NSF FY 83 budgets for High Energy Physics.
- Discussion of the final report of the Technical Assessment Panel.
- Brief status reports from the accelerator laboratories.
- Discussion of the Tevatron-I project.
- Schedule of HEPAP accelerator laboratory program reviews.
- Public Comment (10 minute rule).

Public participation: The meeting is open to the public. The Chairperson of the Committee is empowered to conduct the meeting in a fashion that will, in his judgment, facilitate the orderly conduct of business. Any member of the public who wishes to file a written statement with the Committee will be permitted to do so either before or after the meeting. Members of the public who wish to make oral statements pertaining to agenda items should contact Gloria Decker at 202-252-8990. Requests must be received at least 5 days prior to the meeting and reasonable provision will be made to include the presentation on the agenda.

Minutes: Available for public review and copying at the Public Reading Room, Room 1E-190, Forrestal Building, 1000 Independence Avenue SW., Washington, D.C. between 8:00 a.m. and 4:00 p.m., Monday through Friday, except Federal holidays.

Issued at Washington, D.C., on April 16, 1982.

Howard H. Raiken,

Deputy Advisory Committee Management Officer.

[FR Doc. 82-10810 Filed 4-20-82; 8:45 am]

BILLING CODE 6450-01-M

ENVIRONMENTAL PROTECTION AGENCY

[PF-268; PH-FRL-2105-3]

Certain Companies; Pesticide Petitions

AGENCY: Environmental Protection Agency (EPA).

ACTION: Notice.

SUMMARY: EPA has received pesticide petitions relating to the establishment and amendment of tolerances for residues of certain pesticide chemicals in or on certain raw agricultural commodities.

ADDRESS: Written comments to the product manager (PM) cited in each specific petition at the address below: Registration Division (TS-767C), Office of Pesticide Programs, Envionmental Protection Agency, 401 M St., SW., Washington, DC 20460.

Written comments may be submitted while the petitions are pending before the Agency. The comments are to be identified by the document control number "[PF-268]" and the specific petition number. All written comments filed in response to this notice will be available for public inspection in the product manager's office from 8:00 a.m. to 4:00 p.m., Monday through Friday, except holidays.

FOR FURTHER INFORMATION CONTACT: The product manager cited in each petition at the telephone number provided.

SUPPLEMENTARY INFORMATION: EPA gives notice that the Agency has received the following pesticide petitions relating to the establishment and amendment of tolerances for residues of certain pesticide chemicals in or on certain agricultural commodities in accordance with the Federal Food, Drug, and Cosmetic Act. The analytical method for determining residues, where required, is given in each petition.

FAP 1H5287. ICI Americas, Inc., Concord Pike & New Murphy Rd., Wilmington, DE 19897. Proposed amending 21 CFR Part 193 by establishing a regulation permitting residues of the insecticide (±) alpha-cyano-(3-phenoxyphenyl)methyl (±) *cis, trans*-3-(2,2-dichloroethnyl)-2,2-dimethylcyclopropanecarboxylate in connection with an experimental use program involving the application of the insecticide in the growing of cotton with a tolerance limitation of 5.0 parts per million (ppm) in cottonseed oil. (PM-17, Franklin D.R. Gee, 703-577-2690).

FAP 1H5304. Zoecon Corporation, 975 California Avenue, Palo Alto, CA 94304. Proposes amending 21 CFR Part 193 by establishing a regulation permitting residues of the insecticide *N*-[2-chloro-4-(trifluoromethyl) phenyl]-*d*-valine (±) *a*-cyano (3-phenoxyphenyl)methyl ester in connection with an experimental use program involving the application of the insecticide in the growing of cotton with a tolerance limitation of 0.5 ppm in cottonseed oil. (PM-17, Franklin D.R. Gee, 703-577-2690).

FAP 2H5340. Shell Oil Company, Suite 200, 1025 Connecticut Ave. NW., Washington, DC 20036. Proposes amending 21 CFR Part 561 by establishing a regulation permitting residues of the insecticide cyano (3-phenoxyphenylmethyl-4-chloro-alpha-(1-methylethyl) benzeneacetate with a tolerance limitation of 30.0 ppm on dried grape pomace. (PM-17, Franklin D.R. Gee, 703-557-2690).

PP 2F2657. Shell Oil Co. Proposes amending 40 CFR 180.379 by establishing tolerances for residues of the above mentioned insecticide in or on the raw agricultural commodities raisins and grapes at 4.0 ppm. Proposed analytical method for determining residues is by gas chromatography. (PM-17, Franklin D.R. Gee, 703-557-2690).

PP 2F2588. Dow Chemical Co., PO Box 1706, Midland MI 48640. In the **Federal Register** of December 16, 1981 (46 FR 61330), the EPA announced that the Dow Chemical Co. submitted a pesticide petition (PP 2F2588) proposing to amend 40 CFR 180.342 by establishing a tolerance for residues of the insecticide chlorpyrifos (*O,O*-diethyl *O*-(3,5,6-trichloro-2-pyridyl) phosphorothiate)] and its metabolite 3,5,6-trichloro-2-pyridinol in or on the raw agricultural commodity sunflower seeds at 0.2 ppm.

The Dow Chemical Co. has amended this petition by increasing the tolerance level of 0.25 ppm. The analytical method for determing residues is gas chromatography using a hydrogen flame ionization detector. (PM-12, J. Ellenberger, 703-557-7238).

(Sec. 408(d)(1), 68 Stat. 512, (7 U.S.C. 136))

Dated: April 8, 1982.

Douglas D. Campt.,

Director, Registration Division, Office of Pesticide Programs.

[FR Doc. 82-10683 Filed 4-20-82; 8:45 am]

BILLING CODE 6560-50-M

[PP 9G2168/T361; PH-FRL-2105-8]

Chlorpyrifos; Extension of Temporary Tolerances

AGENCY: Environmental Protection Agency (EPA).

ACTION: Notice.

SUMMARY: EPA has extended temporary tolerances for the combined residues of the insecticide chlorpyrifos and its metabolites in or on the raw agricultural commodities lemons and oranges.

DATE: These temporary tolerances expire June 30, 1983.

FOR FURTHER INFORMATION CONTACT: Jay Ellenberger, Product Manager (PM) 12, Registration Division (TS-767C), Office of Pesticide Programs, Environmental Protection Agency, Rm. 202, CM#2, 1921 Jefferson Davis Highway, Arlington, VA 22202, (703-557-2386).

SUPPLEMENTARY INFORMATION: EPA has extended temporary tolerances for the combined residues of the insecticide chlorpyrifos [*O,O*-diethyl *O*-(3,5,6-trichloro-2-pyridyl phosphorothioate] and its metabolite 3,5,6-trichloro-2-pyridinol in or on the raw agricultural commodities lemons and oranges at 2.5 parts per million (ppm). These temporary tolerances had been established in response to pesticide petition (PP 9G2168), submitted by Dow Chemical Co., P.O. Box 1706, Midland, MI 48640. A food additive regulation has also been extended in or on dried citrus pulp at 15.0 ppm.

The company requested a one-year extension of the temporary tolerances to permit the continued marketing of the raw agricultural commodities named above when treated in accordance with the provisions of experimental use permit (464-EUP-56), which is being extended under the Federal Insecticide, Fungicide, and Rodenticide Act (FIFRA) as amended, (92 Stat. 819; 7 U.S.C. 136).

The scientific data reported and all other relevant material were evaluated, and it was determined that the extension of these temporary tolerances will protect the public health. Therefore, the temporary tolerances have been extended on the condition that the pesticide be used in accordance with the experimental use permit and with the following provisions:

1. The total amount of the active ingredient to be used must not exceed the quality authorized by the experimental use permit.
2. Dow Chemical U.S.A. must immediately notify the EPA of any findings from the experimental use that have a bearing on safety. The company must also keep records of production, distribution, and performance and on request make the records available to any authorized officer or employee of the EPA or the Food and Drug Administration.

These tolerances expire June 30, 1983. Residues not in excess of this amount remaining in or on the raw agricultural commodities after this expiration date will not be considered actionable if the pesticide is legally applied during the term of, and in accordance with, the provisions of the experimental use permit and temporary tolerances. These tolerances may be revoked if the experimental use permit is revoked or if any experience or scientific data with this pesticide indicate that such revocation is necessary to protect the public health.

The Office of Management and Budget has exempted this notice from the requirements of section 3 of Executive Order 12291.

Pursuant to the requirements of the Regulatory Flexibility Act (Pub. L. 96-534, 94 Stat. 1164, 5 U.S.C. 601-612), the Administrator has determined that regulations establishing new tolerances or raising tolerance levels or establishing exemptions from tolerance requirements do not have a significant economic impact on a substantial number of small entities. A certification statement to this effect was published in the **Federal Register** of May 4, 1981 (46 FR 24950).

(Sec. 408(j), 68 Stat. 516, (21 U.S.C. 346a(j)))

Dated: April 7, 1982.

Douglas D. Campt.

Director, Registration Division, Office of Pesticide Programs.

[FR Doc. 82-10681 Filed 4-20-82; 8:45 am]

BILLING CODE 6560-50-M

[PF-269; PH-FRL-2104-8]

Diamond Shamrock Corp.; Food Additive Petition

AGENCY: Environmental Protection Agency (EPA).

ACTION: Notice.

SUMMARY: Diamond Shamrock Corp. has submitted a food additive petition proposing the establishment of a regulation permitting the combined

residues of the fungicide chlorothalonil and its metabolite in citrus oil.

ADDRESS: Written comments to: Henry Jacoby, Product Manager (PM-21), Registration Division (TS-767C), Office of Pesticide Programs, Environmental Protection Agency, 401 M St., SW., Washington, DC 20460.

Written comments may be submitted while the petition is pending before the Agency. The comments are to be identified by the document control number "[PF-269]" and the petition number. All written comments filed in response to this notice will be available for public inspection in the product manager's office from 8:00 a.m. to 4:00 p.m., Monday through Friday, except legal holidays.

FOR FURTHER INFORMATION CONTACT: Henry Jacoby, (703-557-1900).

SUPPLEMENTARY INFORMATION: EPA gives notice that the Diamond Shamrock Corporation, 1100 Superior Avenue, Cleveland, OH 44114, has submitted a food additive petition (FAP 1H5278) to EPA proposing that 21 CFR Part 193 be amended by establishing a regulation permitting the combined residues of the fungicide chlorothalonil (2,4,5,6-tetrachloroisophthalonitrile) and its metabolite 4-hydroxy-2,5,6-trichloroisophthalonitrile in citrus oil at 10 parts per million (ppm) resulting from the application of the fungicide as preharvest application to citrus in connection with an experimental use program in accordance with the Federal Food, Drug, and Cosmetic Act.

(Sec. 409(b)(5), 72 Stat. 1786, (21 U.S.C. 348))

Dated: April 12, 1982.

Douglas D. Campt,

Director, Registration Division, Office of Pesticide Programs.

[FR Doc. 82-10684 Filed 4-20-82; 8:45 am]

BILLING CODE 6560-50-M

[PP 2G2613/T362; PH-FRL-2106-1]

Dow Chemical U.S.A.; Establishment of Temporary Tolerances

AGENCY: Environmental Protection Agency (EPA).

ACTION: Notice.

SUMMARY: EPA has established temporary tolerances for the combined residues of the fungicide 2-chloro-6-(2-furanylmethoxy)-4-trichloromethyl) pyridine and its metabolites in or on the raw agricultural commodities beans (succulent) and peas (succulent). These temporary tolerances were requested by Dow Chemical U.S.A.

DATS: These temporary tolerances expire February 1, 1983.

FOR FURTHER INFORMATION CONTACT: Henry Jacoby, Product Manager (PM) 21, Registration Division (TS-767C), Office of Pesticide Programs, Environmental Protection Agency Rm. 277, CM#2, 1921 Jefferson Davis Highway, Arlington, VA 22202, (703-557-1900).

SUPPLEMENTARY INFORMATION: Dow Chemical U.S.A., P.O. Box 1706, Midland, MI 48640, has requested, in pesticide petition PP 2G2613, the establishment of temporary tolerances for the combined residues of the fungicide 2-chloro-6-(2-furanylmethoxy)-4-trichloromethyl) pyridine and its metabolites convertible to 2-chloro-6-hydroxyisonicotinic acid (calculated as the parent compound) in or on the raw agricultural commodities beans (succulent) and peas (succulent) at 0.05 part per million (ppm).

These temporary tolerances will permit the marketing of the above raw agricultural commodities when treated in accordance with the provisions of the experimental use permit 464-EUP-72 which is being issued under the Federal Insecticide, Fungicide, and Rodenticide Act (FIFRA) as amended, (92 Stat. 819; 7 U.S.C. 136).

The scientific data reported and all other relevant material were evaluated, and it was determined that establishment of the temporary tolerances will protect the public health. Therefore, the temporary tolerances have been established on the condition that the pesticide be used in accordance with the experimental use permit and with the following provisions:

1. The total amount of the active ingredient to be used must not exceed the quantity authorized by the experimental use permit.

2. Dow Chemical U.S.A. must immediately notify the EPA of any findings from the experimental use that have a bearing on safety. The company must also keep records of production, distribution, and performance and on request make the records available to any authorized officer or employee of the EPA or the Food and Drug Administration.

These tolerances expire February 1, 1983. Residues not in excess of these amounts remaining in or on the raw agricultural commodities after this expiration date will not be considered actionable if the pesticide is legally applied during the term of, and in accordance with, the provisions of the experimental use permit and temporary tolerances. These tolerances may be revoked if the experimental use permit is revoked or if any experience or scientific data with this pesticide indicate that such revocation is necessary to protect the public health.

The Office of Management and Budget has exempted this notice from the requirements of section 3 of Executive Order 12291.

Pursuant to the requirements of the Regulatory Flexibility Act (Pub. L. 96-534, 94 Stat. 1164, 5 U.S.C. 610-612), the Administrator has determined that regulations establishing new tolerances or raising tolerance levels or establishing exemptions from tolerance requirements do not have a significant economic impact on a substantial number of small entities. A certification statement to this effect was published in the **Federal Register** of May 4, 1981 (46 FR 24950).

(Sec. 408(j), 68 Stat. 516, (21 U.S.C. 346a(j)))

Dated: April 9, 1982.

Douglas D. Campt.

Director, Registration Division, Office of Pesticide Programs.

[FR Doc. 82-10682 Filed 4-20-82; 8:45 am]

BILLING CODE 6560-50-M

[OPP-50538A; PH-FRL-2104-7]

Issuance of Experimental Use Permits; Correction

AGENCY: Environmental Protection Agency (EPA).

ACTION: Correction notice.

SUMMARY: This notice corrects an experimental use permit, issued to The Upjohn Company, No. 1023-EUP-43, that published in the **Federal Register** of July 8, 1981 (46 FR 35351). In FR Doc. 81-19968, on page 35552, second column, line 30, the total acreage was inaccurate. It is corrected to read "total of 4,409.9 acres, 2,204.95 acres treated both years of the program, are involved * * * "

FOR FURTHER INFORMATION CONTACT: Henry Jacoby, Registration Division (TS-767C), Office of Pesticide Programs, Environmental Protection Agency, Rm. 227, CM#2, 1921 Jefferson Davis Highway, Arlington, VA 22202, (703-557-1900).

Dated: April 9, 1982.

Douglas D. Campt,

Director, Registration Division, Office of Pesticide Programs.

[FR Doc. 82-10811 Filed 4-20-82; 8:45 am]

BILLING CODE 6560-50-M

FEDERAL EMERGENCY MANAGEMENT AGENCY

[FEMA-3084-EM]

Mississippi; Emergency and Related Determinations

AGENCY: Federal Emergency Management Agency.

ACTION: Notice.

SUMMARY: This is a notice of the Presidential declaration of an emergency for the State of Mississippi (FEMA-3084-EM), dated April 10. 1982, and related determinations.

DATED: April 10. 1982.

FOR FURTHER INFORMATION CONTACT: Sewall H. E. Johnson, Disaster Assistance Programs, Federal Emergency Management Agency, Washington, D.C. 20472, (202) 287-0501.

Notice

Pursuant to the authority vested in the Director of the Federal Emergency Management Agency by the President under Executive Order 12148, effective July 15, 1979, and delegated to me by the Director under Federal Emergency Management Agency Delegation of Authority, and by virtue of the Act of May 22, 1974, entitled "Disaster Relief Act of 1974" (88 Stat. 143); notice is hereby given that, in a letter of April 10. 1982, the President declared an emergency as follows:

I have determined that the damage in certain areas of the State of Mississippi resulting from severe storms and tornadoes, on April 3, 1982, is of sufficient severity and magnitude to warrant an emergency declaration under Public Law 93-288. I therefore declare that such an emergency exists in the State of Mississippi.

In order to provide Federal assistance, you are hereby authorized to loan or donate government-owned mobile homes to the State of Mississippi for the purpose of providing temporary housing under the provisions of Section 404 of Pub. L. 93-288. You are further authorized to allocate, from funds available for these purposes, such amounts as you find necessary for administrative expenses.

Notice is hereby given that pursuant to the authority vested in the Director of Federal Emergency Management Agency under Executive Order 12148, and delegated to me by the Director under the Federal Emergency Agency Delegation of Authority, I hereby appoint Mr. R. Jackson Ingram of the Federal Emergency Management Agency to act as the Federal Coordinating Officer for this declared emergency.

I do hereby determine the following areas of the State of Mississippi to have been affected adversely by this declared emergency.

The Counties of Kemper, Leake and Neshoba for assistance as authorized by the President's declaration.

(Catalog of Federal Domestic Assistance No. 83-300, Disaster Assistance)

Lee M. Thomas,

Associate Director, State and Local Programs and Support, Federal Emergency Management Agency.

[FR Doc. 82-10813 Filed 4-20-82; 8:45 am]

BILLING CODE 6718-02-M

[FEMA-653-DR]

Ohio; Amendment to Notice of Major Disaster Declaration

AGENCY: Federal Emergency Management Agency.

ACTION: Notice.

SUMMARY: This notice amends the notice of a major disaster for the State of Ohio (FEMA-653-DR), dated March 26, 1982, and related determinations.

DATED: April 2, 1982.

FOR FURTHER INFORMATION CONTACT: Sewall H. E. Johnson, Disaster Assistance Programs, Federal Emergency Management Agency, Washington, D.C. 20472, (202) 0501.

Notice

The notice of a major disaster for the State of Ohio dated March 26, 1982, is hereby amended to include the following areas among those areas determined to have been adversely affected by the catastrophe declared a major disaster by the President in his declaration of March 26, 1982:

For Individual Assistance only:

The Village of Scott in Van Wert County.

That portion of the City of Toledo south of the boundary commencing at the corporate limits easterly along Airport Highway to the Conrail right of way, continuing east on the Conrail right of way to Route 2 and continuing east on Route 2 to the Maumee River.

(Catalog of Federal Domestic Assistance No. 83.300, Disaster Assistance.)

Lee M. Thomas,

Associate Director, State and Local Programs and Support, Federal Emergency Management Agency.

[FR Doc. 82-10814 Filed 4-20-82; 8:45 am]

BILLING CODE 6718-02-M

[FEMA-653-DR]

Ohio; Amendment to Notice of Major Disaster Declaration

AGENCY: Federal Emergency Management Agency.

ACTION: Notice.

SUMMARY: This notice amends the notice of a major disaster for the State of Ohio (FEMA-653-DR), dated March 26, 1982, and related determinations.

DATED: April 6, 1982.

FOR FURTHER INFORMATION CONTACT: Sewall H. E. Johnson, Disaster Assistance Programs, Federal Emergency Management Agency, Washington, D.C. 20472, telephone (202) 287-0501.

Notice

The notice of a major disaster for the State of Ohio dated March 26, 1982, is hereby amended to include the following areas among those areas determined to have been adversely affected by the catastrophe declared a major disaster by the President in his declaration of March 26, 1982:

For Individual Assistance only:

Williams County
Fulton County

(Catalog of Federal Domestic Assistance No. 83.300, Disaster Assistance.)

Lee M. Thomas,

Associate Director, State and Local Programs and Support, Federal Emergency Management Agency.

[FR Doc. 82-10815 Filed 4-20-82; 8:45 am]

BILLING CODE 6718-02-M

[FEMA-655-DR]

Texas; Major Disaster and Related Determinations

AGENCY: Federal Emergency Management Agency.

ACTION: Notice.

SUMMARY: This is a notice of the Presidential declaration of a major disaster for the State of Texas (FEMA-655-DR), dated April 8, 1982, and related determinations.

DATE: April 8, 1982.

FOR FURTHER INFORMATION CONTACT: Sewall H. E. Johnson, Disaster Assistance Programs, SLPS, Federal Emergency Management Agency, Washington, D.C. 20472, telephone No. (202) 287-0501.

Notice

Pursuant to the authority vested in the

Director of the Federal Emergency Management Agency by the President under Executive Order 12148, effective July 15, 1979, and delegated to me by the Director under Federal Emergency Management Agency delegation of authority, and by virtue of the Act of May 22, 1974, entitled "Disaster Relief Act of 1974" (** Stat. 143); notice is hereby given that, in a letter of April 8, 1982, the President declared a major disaster as follows:

I have determined that the damage in certain areas of the State of Texas resulting from severe storms and tornadoes beginning on April 2, 1982, is of sufficient severity and magnitude to warrant a major disaster declaration under Pub. L. 93-288. I therefore declare that such a major disaster exists in the State of Texas.

In order to provide Federal assistance, you are hereby authorized to allocate, from funds available for these purposes, such amounts as you find necessary for Federal disaster assistance and administrative expenses. Consistent with the requirement that Federal assistance be supplemental, the Federal funds provided under Pub. L. 93-288 for public assistance will be limited to 75 percent of total eligible costs in the designated area except for technical assistance which will be funded at 100 percent.

The time period prescribed for the implementation of Section 313(a), priority to certain applications for public facility and public housing assistance, shall be for a period not to exceed six months after the date of this declaration.

Notice is hereby given that pursuant to the authority vested in the Director of the Federal Emergency Management Agency under Executive Order 12148, and delegated to me by the Director under the Federal Emergency Management Agency delegation of authority, I hereby appoint Mr. William C. Tidball of the Federal Emergency Management Agency to act as the Federal Coordinating Officer for this declared major disaster.

I do hereby determine the following area of the State of Texas to have been affected adversely by this declared major disaster.

Lamar County for Individual Assistance and Public Assistance.

(Catalog of Federal Domestic Assistance No. 83-300, Disaster Assistance)

Lee M. Thomas,

Associate Director, State and Local Programs and Support, Federal Emergency Management Agency.

[FR Doc. 82-10816 Filed 4-20-82; 8:45 am]

BILLING CODE 6718-02-M

DEPARTMENT OF HEALTH AND HUMAN SERVICES

Health Services Administration

Scholarships for First-year Students of Exceptional Financial Need Program; Availability of Awards for the 1982-83 School Year for Medical, Osteopathy and Dental Schools

Section 758 of the Public Health Service (PHS) Act (42 U.S.C. 294z) authorizes the Secretary of Health and Human Services to make grants to public or private non-profit schools of medicine, osteopathy, dentistry, optometry, pharmacy, podiatry, or veterinary medicine for scholarships to be awarded by the individual schools to full-time first-year students of exceptional financial need. The statute requires that preference in funding be given to schools of medicine, osteopathy, and dentistry.

Public Law 97-35, enacted August 13, 1981, amended section 758 of the PHS Act by removing the requirement that grants be distributed among all eligible health professions schools.

Notice is hereby given that, for the school year 1982-83, due to the limited availability of funds and in keeping with the statutory requirement that preference in funding be given to schools of medicine, osteopathy and dentistry, grants under the Scholarships for First-Year Students of Exceptional Financial Need Program will be made only to schools of medicine, osteopathy, and dentistry.

FOR ADDITIONAL INFORMATION CONTACT: Michael Heningburg, Director, Division of Student Services, Bureau of Health Personnel Development and Service, Health Services Administration, Center Building, Room 9-50, 3700 East-West Highway, Hyattsville, Maryland 20782, Telephone: (301) 436-7227.

Dated: April 12, 1982.

John H. Kelso,

Acting Administrator.

[FR Doc. 82-10817 Filed 4-20-82; 8:45 am]

BILLING CODE 4160-16-M

DEPARTMENT OF THE INTERIOR

Bureau of Land Management

[INT DEIS-82-15]

Draft Environmental Impact Statement, Preliminary Wilderness Recommendations for Tonapah Resource Area, Battle Mountain District, Nevada

AGENCY: Bureau of Land Management, Interior.

ACTION: Notice of availability of the Draft Tonopah Wilderness Environmental Impact Statement.

SUMMARY: The draft environmental impact statement (DEIS), containing the preliminary wilderness recommendations for the Wilderness Study Areas in the Battle Mountain District's Tonopah Resource Area, is available for public review. The Wilderness Study Areas (WSAs) being considered for possible inclusion into the National Wilderness Preservation System are located in northeastern Nye County, Nevada. They are:

WSA name	Number	Acreage
Kawich	NV-060-019	54,320
Rawhide Mountain	NV-060-059	64,360
Fandango	NV-060-190	40,940
Morey	NV-060-191	20,120
Palisade Mesa	NV-060-142/162	99,550
The Wall	NV-060-163	38,000
South Reveille	NV-060-112	106,200
Blue Eagle	NV-060-158/199	59,560
Grant Range	NV-060-166	5,840

The wilderness study in the Tonopah Resource Area is being conducted as an amendment to the existing land use plan. The DEIS analyzes the effects of implementing such a planning amendment.

The DEIS has been prepared in accordance with the Council On Environmental Quality regulations for the implementation of the National Environmental Policy Act.

DATES: The public is invited to submit their views on the preliminary wilderness recommendations during public hearings to be held on June 5th at the Tonopah Convention Center, 301 Brougher, Tonopah, Nevada from 1-3 P.M. and on June 7th at the Pioneer Hotel, 221 S. Virginia, Reno, Nevada from 7-9 P.M. The 90-day public comment period on the DEIS ends on July 9th.

AVAILABILITY: The DEIS will be available for review at all BLM offices in Nevada as well as at public libraries in Battle Mountain, Carson City, Ely, Las Vegas , and Tonopah, Nevada. Copies will also be placed in the University of Nevada libraries at Las Vegas and Reno.

FOR FURTHER INFORMATION CONTACT: Comments and requests for copies of the DEIS should be sent to Mr. Leslie A. Monroe, Tonopah Resource Area Manager at Building 102 Military Circle, P.O. Box 911, Tonopah, Nevada 89049 or call (702) 482-6214.

SUPPLEMENTARY INFORMATION: Five alternatives, including a preferred alternative, are analyzed in the DEIS. They are: All Wilderness, Emphasis On

Manageability, Emphasis On Resource Development, No action or No Wilderness, and the Preferred Alternative. The Preferred Alternative represents the BLM's preliminary wilderness recommendations for the Wilderness Study Areas in the Tonopah Resource Area.

Mineral resources survey reports will be prepared by the U.S. Geological Survey and the U.S. Bureau of Mines on all areas with a preliminary recommendation as suitable for wilderness designation. The mineral resources survey reports and the results of public participation will be considered by the Secretary of the Interior in making the final recommendations on wilderness suitability. Congress will make the final decision on wilderness suitability and nonsuitability.

Dated: April 7, 1982.

H. James Fox,

Battle Mountain District Manager.

[FR Doc. 82-10866 Filed 4-20-82; 8:45 am]

BILLING CODE 4310-84-M

[CA 6829]

Contra Costa County, Calif.; Conveyance of Public Land

April 13, 1982.

Notice is hereby given that pursuant to the Act of October 21, 1976 (90 Stat. 2743; 43 U.S.C. 1713, 1719), the Pacific Gas and Electric Company, 77 Beale Street, San Francisco, California 94106, has purchased by noncompetitive sale public land in Contra Costa County, California, described as:

Mount Diablo Meridian, California

T. 2 N., R. 1 W.,
Sec. 17, Lots 9 and 10.
Containing 0.24 acre.

The purpose of this notice is to inform the public and interested state and local governmental officals of the issuance of the conveyance document to the Pacific Gas and Electric Company.

Harold R. Dietz,

Acting Chief, Lands Section, Branch of Lands and Minerals Operations.

[FR Doc. 82-10831 Filed 4-20-82; 8:45 am]

BILLING CODE 4310-84-M

[CA 9295]

San Diego County, Calif.; Conveyance of Public Land

April 12, 1982.

Notice is hereby given that pursuant to the Act of October 21, 1976 (90 Stat. 2743; 43 U.S.C. 1713), Donald E. and Miriam T. Viol, 3592 E. Hillhaven Drive, West Covina, California 91791, have purchased by noncompetitive sale public land in San Diego County, California described as:

San Bernardino Meridian, California

T. 11 S., R. 3 E.,
Sec. 22, Lot 9.
Containing 5.59 acres.

The purpose of this notice is to inform the public and interested state and local governmental officials of the issuance of the conveyance document to Mr. and Mrs. Viol.

Harold R. Dietz,

Acting Chief, Lands Section, Branch of Lands and Minerals Operations.

[FR Doc. 82-10832 Filed 4-20-82; 8:45 am]

BILLING CODE 4310-84-M

Glenwood Springs Resource Area (Colorado) Resource Management Plan; Public Workshop

ACTION: Public Workshop Schedule for Glenwood Springs Resource Area Resource Management Plan (RMP).

SUMMARY: The Glenwood Springs Resource Area is currently preparing a Resource Management Plan (RMP) to analyze alternative proposals for management of the public lands within the Resource Area.

The RMP is a land use plan that takes into account all resources and all uses of those resources, both potential and realized. It is being prepared in accordance with the Federal Land Policy and Management Act of 1976. Public issues and management concerns have been identified, field inventories and background data have been collected, and an analysis of the existing management situation is complete. Draft alternatives for land management have been formulated and are available for public review and comment.

Public participation is essential to the decision making process. Six public workshops will be held during May to present these draft alternatives to the public and to provide for public input. The meetings are scheduled:

Location	Date	Place and time
Rifle	May 3	Assembly of God Church, 550 East Fifth, 2-5 and 7-9 p.m.
Eagle	May 4	School Dist. Board Room, 757 East Third Street, 6-9 p.m.
McCoy	May 5	McCoy School (Classroom), 0026 McCoy Road, 6-9 p.m.
Glenwood Springs.	May 6	Colorado Room, Hotel Colorado, 526 Pine, 2-5 and 7-9 p.m.
Grand Junction.	May 10	BLM Conference Room, 764 Horizon Drive, 2-5 and 7-9 p.m.
Denver	May 12	Ramada Inn Foothills, 11595 West Sixth Avenue, 2-5 and 7-9 p.m.

FOR FURTHER INFORMATION CONTACT: Interested parties wishing to discuss or review the RMP documents or data available to date may visit or call Dave Mensing, Team Leader, BLM Glenwood Springs Resource Area Office, 50629 Highway 6 & 24, P.O. Box 1009, Glenwood Springs, CO 81602; telephone (303) 945-2341.

Richard Freel,

Associate District Manager.

[FR Doc. 82-10818 Filed 4-20-82; 8:45 am]

BILLING CODE 4310-84-M

[U-4342]

Utah; Termination of Classification for Multiple-Use Management and Termination of Mineral Segregation

1. Pursuant to the authority delegated by Bureau Order No. 701 dated July 23, 1964 (29 FR 10526), the Bureau of Land Management Multiple-Use Classification Order dated March 27, 1968 and published in the **Federal Register** March 28, 1968, Vol. 33, No. 61, Page 5110 and modification dated December 1, 1970 published in the **Federal Register** December 2, 1970 pages 18336-7 is hereby terminated.

The public lands involved aggregate 1,365,340 acres in Juab County.

2. The public lands described in the notice were classified for Multiple-Use Management and segregated from appropriation under the Agricultural Land Laws (43 U.S.C. Parts 7 and 9; 25 U.S.C. Sec. 334), and from sales under section 2455 of the Revised Statutes as amended (43 U.S.C. 1171). The notices also segregated 4,926.13 acres of the lands from all forms of appropriation, entry, location or selection under the public land laws, including the general mining laws, and from surface use and occupancy under the mineral leasing laws.

3. The lands described in this paragraph are not affected by this termination order, and will remain subject to the terms of the original classification order listed in paragraphs 1 and 2 above, including segregation from mineral location.

Salt Lake Meridian, Utah

Fish Springs Pony Express Station

T. 11 S., R. 14 W.,
Sec. 23, NW¼NW¼.

Black Rock Pony Express Station

T. 11 S., R. 13 W.,
Sec 11, SW¼NE¼.

Dugway Pony Express Station

T. 11 S., R. 11 W.,
Sec. 13, SE¼SW¼.

Boyd Pony Express Station

T. 11 S., R. 15 W.,
Sec. 21, SW¼NE¼SW¼, SE¼NW¼SW¼, NE¼SW¼SW¼, NW¼SE¼SW¼.

Thoms Creek

T. 11 S., R. 17 W.,
Sec. 20, N½NE¼;
Sec. 21, N½NW¼.

Geode Beds

T. 11 S., R. 11 W.,
Sec. 6, all;
Sec. 7, N½.
T. 11 S., R. 12 W.,
Sec. 1, all;
Sec. 12, N½.

Topaz Mountain

T. 13 S., R. 11 W.,
Sec. 8, E½;
Sec. 9, all;
Sec. 15, W½;
Sec. 17, E½.

Paul Banyon Wood Pile

T. 12 S., R. 2½ W.,
Sec. 13, S½ of lots 4 and 5.

Baker Springs

T. 14 S., R. 8 W.,
Sec. 10, NW¼SE¼.

Aggregating 4074 acres.

4. Pursuant to the regulations set forth in (43 CFR 2461.5(c)(2)), the above classification, except for lands in paragraph 3, is hereby terminated. At 10:00 a.m., on May 15, 1982 the lands described in said Notices, except for lands in paragraph 3 above, will be open to operation of the public land laws generally, subject to valid existing rights, the provisions of existing withdrawals, and the requirement of applicable law. All valid applications received at or prior to 10:00 a.m., on May 15, 1982 shall be considered as simultaneously filed at that time. Those received thereafter will be considered in the order of filing.

5. The lands described in the above notices, except for lands in paragraph 3, will also be open to location under the United States Mining Laws at 10:00 a.m. on May 15, 1982.

Inquiries concerning these lands should be addressed to the Chief, Branch of Lands and Minerals Operations, Bureau of Land Management, 136 East South Temple, Salt Lake City, Utah 84111.

Dated: April 12, 1982.

Roland G. Robison,

State Director.

[FR Doc. 82-10819 Filed 4-20-82; 8:45 am]

BILLING CODE 4310-84-M

Bureau of Reclamation

Deaver Irrigation District, Shoshone Project, Wyoming; Intent To Negotiate a Rehabilitation and Betterment Program Loan Repayment Contract

The Department of the Interior, through the Bureau of Reclamation, intends to begin contract negotiations with the Deaver Irrigation District (ID) of Deaver, Wyoming, for repayment of a loan covering the cost of a rehabilitation and betterment (R&B) program. The Deaver ID, which encompasses the Frannie Division of the Shoshone Project, is located in Park and Bighorn counties of northwest Wyoming. The proposed R&B contract with Deaver ID would be negotiated pursuant to the Act of October 7, 1949 (63 Stat. 724), and the Act of June 7, 1902 (32 Stat. 388), as amended and supplemented.

The lateral system for the district was constructed between 1917 and 1921 in very permeable subsoils. The system has a history of excessive seepage and water erosion problems and many of the control structures and measuring devices are obsolete and deteriorated. Major objectives of the proposed R&B program include conserving water, lowering the ground water table, reclaiming wet areas adjacent to the existing waterways, reducing risk of failure, and improving service to water users.

The proposed R&B program would replace approximately 180 control structures and water measuring devices, and replace 28.5 miles of open earth laterals with 12 miles of concrete pipe and 16.5 miles of slip-form concrete lining. The district is seeking authorization of a loan for $1.6 million to perform this work.

Approval of the loan is ultimately dependent upon approval of the form of the proposed contract by the Secretary of Interior and completion of a congressional review of the Secretary's findings regarding ability of the district to repay the loan. Certain committees of the Congress have a 60-day period to respond in writing if there are objections to the loan or there are reasons to approve the loan earlier.

The public may observe any contract negotiation session. Advance notice of such sessions will be furnished to interested parties who have provided a timely written request identifying the proposed contract with Deaver ID. Requests should be addressed to Regional Director, Bureau of Reclamation, Attention Code UM-440, P.O. Box 2553, Billings, Montana 59103.

All written correspondence concerning the proposed contracts will be made available to the general public pursuant to the terms and procedures of the Freedom of Information Act (80 Stat. 383), as amended.

The proposed draft contracts will be made available for public review and receipt of written comments for a 30-day period following completion of contract negotiations. Unless significant interest is evidenced during the negotiations, the availability of the contracts for public review and comment will not be publicized further. The proposed contract form may be obtained by contacting Mr. William Crosby, Chief, Economics and Repayment Branch, Division of Water and Land, at the address stated above or by telephone (406) 657-6413.

Dated: April 13, 1982.

R. N. Broadbent,

Commissioner of Reclamation.

[FR Doc. 82-10821 Filed 4-20-82; 8:45am]

BILLING CODE 4310-09-M

Fish and Wildlife Service

Endangered Species Permits Issued for the Month of March 1982

Notice is hereby given that the U.S. Fish and Wildlife Service has taken the following action with regard to permit applications duly received according to Section 10 of the Endangered Species Act of 1973, as amended, 16 U.S.C. 1539. Each permit listed was issued only after it was determined that it was applied for in good faith, and that by granting the permit it will not be to the advantage of the endangered species; and that it will be consistent with the purposes and policy set forth in the Endangered Species Act of 1973 as amended.

Additional information on these permit actions may be requested by contacting the Federal Wildlife Permit Office, Box 3654, Arlington, VA 22203, telephone (703/235-1903) or by appearing in person at the Federal Wildlife Permit Office, 1000 N. Glebe Road, Room 605, Arlington, VA, between the hours of 9:00 a.m. and 3:00 p.m. weekdays.

International Animal Exchange	X8745	3-01-82
Wayne D. Dubuc	X8713	3-03-82
San Francisco Zoological Park	X8743	3-03-82
University of North Carolina	X8742	3-08-82
San Diego Wild Animal Park	X8647	3-10-82
International Animal Exchange	X8715	3-11-82
Kenneth K. Kalencik	X8766	3-19-82
New York Zoological Society	X8817	3-19-82

Dated: April 16, 1982.

R. K. Robinson,

Chief, Branch of Permits, Federal Wildlife Permit Office.

[FR Doc. 82-10867 Filed 4-20-82; 8:45 am]

BILLING CODE 4310-55-M

Endangered Species Permit; Receipt of Application

Applicant: National Zoological Park, Washington, DC.

The applicant requests a permit to import six (6) captive-reared gharial *(Gavialis gangeticus)* from the Gharial Breeding Centre, Nepal, for enhancement of propagation.

Humane care and treatment during transport has been indicated by the applicant.

Documents and other information submitted with this application are available to the public during normal business hours in Room 601, 1000 N. Glebe Road, Arlington, Virginia, or by writing to the U.S. Fish and Wildlife Service, Federal Wildlife Permit Office, P.O. Box 3654, Arlington, VA 22203.

This application has been assigned file number PRT 2-9029. Interested persons may comment on this application until May 21, 1982 by submitting written data, views, or arguments to the above address. Please refer to the file number when submitting comments.

Dated: April 16, 1982.

R. K. Robinson,

Chief, Branch of Permits, Federal Wildlife Permit Office.

[FR Doc. 82-10868 Filed 4-20-82; 8:45 am]

BILLING CODE 4310-55-M

National Park Service

Grant Village, Yellowstone National Park

AGENCY: National Park Service, Interior.

ACTION: Finding of no significant impact for development concept plan.

SUMMARY: The National Park Service announces the Finding of No Significant Impact, Approved February 8, 1982, for Grant Village Development Concept Plan, Yellowstone National Park.

The selected plan will implement the earlier decision to reduce impacts at environmentally sensitive areas by substituting accommodations at Grant Village. The plan includes the proposal to construct approximately 700 visitor lodging units with 2.5 to 3 acres of additional parking, a registration office, a restaurant complex including a gift shop and a store featuring camping supplies. Support facilities proposed include: dormitories for 275 concessioner employees, 50 additional trailer sites for concessioner employees, housing for 40 YACC members, housing for National Park Service employees and a multi-purpose building containing recreation and possibly food-service facilities for employees. Construction of trails among new facilities, rerouting trails north of the visitor center, and reengineering of two road intersections to eliminate traffic circulation problems are also included.

Proposed improvements to existing utilities include: construction of a water treatment plant; improvement of water intake, water tanks, and distribution system; construction of an additional 250,000 gpd sewage system; installation of a 2,500 kilowatt standby generator; construction of a solid waste compactor and transfer station; and installation of additional telephone lines.

Several areas are proposed as development sites for additional visitor accommodations. These sites include: 15 acres in the southeast quadrant of the lodging area and about 80 acres south and east of the dump.

The Grant Village area has been distrubted previously by the existing development. The 1974 park master plan includes expansion of visitor use facilities at Grant Village in order to reduce the environmental impact on primary park resources by removing overnight visitor accommodations at Old Faithful and Fishing Bridge.

The following are other alternatives considered:

No Action Alternative

The National Park Service would not change existing patterns of visitor use. Development would not be removed from environmentally sensitive areas (Old Faithful, West Thumb, Fishing Bridge, and Pelican Valley.)

Allow Unlimited Development

The National Park Service would allow unlimited expansion of facilities throughout Yellowstone National Park. Grant Village and other areas would be developed.

Remove All Facilities and Restrict Visitor Use

Grant Village would not be developed. Development would be removed from environmentally sensitive areas.

Develop Facilities Outside the Park

Remove overnight accommodations from environmentally sensitive areas and encourage the development of replacement facilities outside the park boundaries.

Reduce Facilities

Reduce the kinds and quantities of visitor facilities, services, and activities, in Yellowstone National Park.

The public review period for the environment assessment and preferred alternative took place from July 1, 1981, through July 31, 1981. Notice of availability of these documents was sent to over 100 media representatives throughout the Yellowstone National Park region. A public response form was prepared that sought comments on the various planning issues.

All practical means to avoid and/or minimize environmental harm has been adopted from alternatives considered.

FOR FURTHER INFORMATION CONTACT: A copy of the assessment of alternatives and preferred alternative along with the record of decision and finding of no significant impact is available from the Superintendent, Yellowstone National Park, Wyoming, 82190, and at the Rocky Mountain Regional Office, National Park Service, 655 Parfet Street, Post Office Box 25287, Lakewood, Colorado 80225.

Dated: April 1, 1982.

James B. Thompson,

Acting Regional Director, Rocky Mountain Region.

[FR Doc. 82-10885 Filed 4-20-82; 8:45 am]

BILLING CODE 4310-70-M

Kalaupapa National Historical Park Advisory Commission; Meeting

Notice is hereby given in accordance with the Federal Advisory Committee Act that a meeting of the Kalaupapa National Historical Park Advisory Commission will be held at 9:00 a.m. on Thursday, May 13, 1982, at Paschoal Community Hall, Kalaupapa, Molokai, Hawaii.

The Advisory Commission was established by Public Law 95-565 to provide advice with respect to park development, operations, public visitation, and employee training.

Members of the Commission are as follows:

Rev. David K. Kaupu, Chairman
Mr. Clifford K. Andeson
Mr. Robert L. Barrel
Mrs. Kuulei Bell
Mr. James Brede
Mr. Shoichi Hamai
Mr. Paul Harada
Mr. Isaac Keao
Mr. Richard Marks
Mr. Ralston Nagata
Mr. Bernard Punikaia

This will be the initial meeting of the newly appointed commission, and as such, will be devoted largely to organizational matters and a review of

the new park's plans and programs to date.

The meetings are open to the public. Any member of the public may file with the Commission a written statement concerning the matters to be discussed.

Persons wishing to receive further informatin on this meeting or who wish to submit written statements may contact Mr. Bryan Harry, Pacific Area Director, National Park Service, 300 Ala Moana Boulevard, Box 50165, Honolulu, Hawaii 96850; telephone (808) 546-7584.

Minutes of the meeting will be available for public inspection by June 12, 1982, in the Office of the Pacific Area Director, National Park Service, 300 Ala Moana Boulevard, Room 6305, Honolulu, Hawaii 96850.

Dated: April 6, 1982.

W. Lowell White,

Acting Regional Director, Western Region.

[FR Doc. 82-10880 Filed 4-20-82; 8:45 am]

BILLING CODE 4310-70-M

Potential 1982 U.S. World Heritage Nominations

AGENCY: National Park Service, Interior.

ACTION: Notice and request for public comment.

SUMMARY: On January 8, 1982, the Department of the Interior, through the National Park Service, set forth in a public notice the process and schedule that will be used in calendar year 1982 to identify and prepare U.S. nominations to the World Heritage List (47 FR 1034). In addition, the January 8 notice identified the criteria and requirements that U.S. properties must satisfy before nomination for World Heritage status, and solicited public comments and suggestions regarding cultural and natural properties that should be considered as potential U.S. nominations this year. This notice announces and invites comment on the four cultural and six natural properties described below that have been identified as potential 1982 U.S. World Heritage nominations.

DATES: Written comments or recommendations regarding any property listed herein as a potential 1982 U.S. World Heritage nomination must be received by May 21, 1982 to ensure full consideration. The final list of proposed 1982 nominations will be selected from among the potential nominations, and will be published in the **Federal Register** by July 1, 1982. A draft nomination document will be prepared for any property selected as a proposed nomination. In November 1982, the Federal Interagency Panel for World Heritage will review the accuracy and completeness of the draft 1982 nomination(s) and will make recommendations to the Assistant Secretary of the Interior for Fish and Wildlife and Parks. The Assistant Secretary subsequently transmits any approved nomination(s) on behalf of the United States to the United Nations Educational, Scientific, and Cultural Organization, through the Department of State, by December 15 for evaluation by the World Heritage Committee in a process that could lead to inscription on the World Heritage List by fall 1983. Notice of transmittal of U.S. nominations will be published in the **Federal Register.**

ADDRESS: Written comments or recommendations should be sent to the Director, National Park Service, U.S. Department of the Interior, Washington, D.C. 20240. Attention: World Heritage Convention—773.

FOR FURTHER INFORMATION CONTACT: Mr. Robert A. Ritsch, Associate Director, Recreation Resources, National Park Service, U.S. Department of the Interior, Washington, D.C. 20240 (202/343-4462).

SUPPLEMENTARY INFORMATION: The Convention Concerning the Protection of the World Cultural and Natural Heritage, now ratified by the United States and 62 other countries, has established a system of international cooperation through which cultural and natural properties of outstanding universal value to mankind may be recognized and protected. The Convention seeks to put into place an orderly approach for coordinated and consistent heritage resource protection and enhancement throughout the world.

Participating nations identify and nominate their sites for inclusion on the World Heritage List, which currently includes 112 cultural and natural properties. The World Heritage Committee judges all nominations against established criteria. Under the Convention, each participating nation assumes responsibility for taking appropriate legal, scientific, technical, administrative, and financial measures necessary for the identification, protection, conservation, and rehabilitation of World Heritage properties situated within its borders.

In the United States, the Department of the Interior is responsible for directing and coordinating U.S. participation in the World Heritage Convention. The Department implements its responsibilities under the Convention in accordance with the statutory mandate contained in Title IV of the National Historic Preservation Act Amendments of 1980 (Pub. L. 96-515; 16 U.S.C. 470a-1, a-2). On October 20, 1981, the Interior Department published in the **Federal Register** the policies and procedures which it proposed to use to carry out this legislative mandate (46 FR 51557). The proposed rules contain additional information on the Convention and its implementation in the United States, and identify the specific requirements that U.S. properties must satisfy before they can be nominated for World Heritage status, i.e., the property must have previously been determined to be of national significance, its owner must concur in writing to its nomination, and its nomination must include evidence of such legal protections as may be necessary to ensure preservation of the property and its environment.

The Federal Interagency Panel for World Heritage assists the Department in implementing the Convention by making recommendations on U.S. World Heritage policy, procedures, and nominations. The Panel is chaired by the Assistant Secretary for Fish and Wildlife and Parks, and includes representatives from the Office of the Assistant Secretary for Fish and Wildlife and Parks, the National Park Service, and the U.S. Fish and Wildlife Service within the Department of the Interior; the President's Council on Environmental Quality; the Smithsonian Institution; the Advisory Council on Historic Preservation; National Oceanic and Atmospheric Administration, Department of Commerce; and the Department of State.

Potential 1982 U.S. World Heritage Nominations

The Department of the Interior, through the National Park Service, has identified the following four cultural and six natural properties as potential 1982 U.S. nominations to the World Heritage List. With one exception, each of these properties was included on the draft inventory of potential future U.S. World Heritage nominations, published in the **Federal Register** on September 1, 1981 (46 FR 43892). Wright Brothers National Memorial was not included on the inventory as it was formally nominated for World Heritage status in December 1980. It was subsequently withdrawn from consideration in May 1981 after questions were raised regarding its international significance and integrity; it is now being considered for possible renomination. A brief description is provided for each property, along with the World Heritage criteria that it appears to satisfy. The final list of proposed 1982 U.S. nominations to the World Heritage List will be selected

from among the potential nominations included herein. Identification of a property as a potential 1982 nomination does not confer World Heritage status on it. A draft nomination document will be prepared for each property that is ultimately selected as a proposed 1982 nomination. The Department encourages all interested parties to comment and make recommendations on the potential nominations, as these comments and additional evaluation will serye as the basis for identifying proposed 1982 nominations.

The following cultural properties, arranged alphabetically by theme, and natural properties, arranged alphabetically by natural region, have been identified as potential 1982 U.S. World Heritage nominations:

I. Cultural Properties

Archaeology

Chaco Culture National Historical Park, New Mexico. From 600 to 1200 A.D., this area was inhabited by a group of Pueblo Indians distinguished for the massiveness of the buildings they constructed and the excellence of their masonry—achievements that were realized to the fullest in the 11th and 12th centuries.

Among the largest of the pueblos was Pueblo Bonito, built to a height of 5 stories on a floor plan exceeding 3 acres and capable of housing roughly 1200 persons in about 800 rooms. Criteria: (ii) Exerted great influence over a span of time and within a cultural area of the world on developments in town-planning; and (iii) bears a unique testimony to a civilization which has disappeared.

European Exploration and Colonial Settlement

La Fortaleza-San Juan National Historic Site, Puerto Rico. Spanish defenses at San Juan guarded their sea lanes to the Caribbean; at this site they founded one of their earliest colonies in the Americas. La Fortaleza, the first fortification of San Juan (built 1533–40), has been the residence of the island's governors since the 1620s. The massive masonry citadel of El Morro was begun in 1591. A comparative study of similar resources will be undertaken to assess the relative importance of this complex. *Criteria:* (iv) An outstanding example of a type of structure which illustrates a significant stage in history; and (vi) directly and tangibly associated with events of outstanding universal significance.

Modern U.S. Architecture

Wainwright Building, St. Louis, Missouri. Significant prototype of the modern office building, constructed in 1890–91. It was Sullivan's first commission involving the use of complete iron and steel framing. This building represents a deliberate attempt to create an ahistorical form expressive of the new mass of the multistory office block. For Sullivan, the potential aesthetic quality of the tall building lay in its unusual height. To emphasize this height to the maximum degree, he devised a system of closely ranked, pierlike verticals that give the street elevations their forceful thrust. *Criteria:* (i) Represents a unique artistic achievement, a masterpiece of the creative genius; and (ii) has exerted great influence, over a span of time, and within a cultural area of the world, on developments in architecture.

Comments on the relative significance of the Wainwright Building in comparison with other works of Louis Sullivan and its subsequent impact on developments in architecture and city planning are especially encouraged.

Science and Industry

Wright Brothers National Memorial/First Powered Flight, Kitty Hawk, North Carolina. This site is directly and tangibly associated with the event of the flight of the world's first powered and controlled airplane, which was completed by Wilbur and Orville Wright on December 17, 1903. This event revolutionized travel patterns and modes of warfare throughout the world. The invention of the airplane has changed the course of human history, as the pace and scope of life has quickened and human endeavor broadened. *Critiera:* (vi) Directly and tangibly associated with an event of outstanding universal significance. As noted, Wright Brothers National Memorial was previously nominated in 1980 and subsequently withdrawn, and is now being considered for possible renomination.

II. Natural Properties

Appalachian Ranges

Great Smoky Mountains National Park, Tennessee/North Carolina. This tract, which includes one of the oldest uplands on earth, has a diversity of lush vegetation associated with its varied topography, including spruce-fir, hemlock, deciduous, and mixed forests. The area has been designated a Biosphere Reserve. *Criteria:* (ii) An outstanding example of biological evolution, and (iii) contains superlative natural phenomena and areas of exceptional natural beauty.

Cascade Range

Crater Lake National Park, Oregon. This unique, deep blue lake lies at the center of Mount Mazama, an ancient volcanic peak that collapsed centuries ago. The lake is bounded by multicolored lava walls extending 500 to 2,000 feet above the lake's waters. *Criteria:* (ii) An outstanding example of significant geological processes, and (iii) contains superlative natural phenomena, formations, and areas of exceptional natural beauty.

Chihuahuan Desert

Carlsbad Caverns National Park, New Mexico/Guadalupe Mountains National Park, Texas. Rising abruptly from the surrounding desert, the mountain mass known as the Guadalupe Escarpment located within Guadalupe Mountains National Park contains portions of the world's most extensive and significant Permian limestone fossil reef. A tremendous earth fault and unusual flora and fauna are also found here. Carlsbad Caverns National Park, another portion of the Guadalupe Escarpment, includes the largest underground chambers yet discovered, and has many magnificent and curious cave formations, including an array of speleothems. *Criteria:* (i) An outstanding example illustrating a major stage of the earth's evolutionary history, (ii) an outstanding example of significant geological processes, and (iii) contains superlative natural phenomena, formations, and areas of exceptional natural beauty.

Hawaiian Islands

Hawaii Volcanoes National Park, Hawaii. This site contains outstanding examples of active and recent volcanism, along with luxuriant vegetational development at its lower elevations. The area has been designated a Biosphere Reserve. *Criteria:* (i) An outstanding example illustrating the earth's evolutionary history, (ii) an outstanding example of significant geological processes, and (iii) contains superlative natural phenomena, formations, and areas of exceptional natural beauty.

Pacific Mountain System

Katmai National Park, Alaska. This area's interior wilderness includes the Valley of 10,000 Smokes, the result of the 1912 volcanic eruption of Mt. Katmai. The eruption produced countless fumaroles, a few of which are still active. *Criteria:* (ii) An outstanding

example of significant geological processes, and (iii) contains superlative natural phenomena, formations, and areas of exceptional natural beauty.

Sierra Nevada

Yosemite National Park, California. Granite peaks and domes rise high above broad meadows in the heart of the Sierra Nevada, along with groves of sequoias and related tree species. Mountains, lakes, and waterfalls, including the nation's highest, are found here. *Criteria:* (ii) An outstanding example of significant geological processes and biological evolution, and (iii) contains superlative natural phenomena, formations and areas of exceptional natural beauty.

Dated: April 15, 1982.

G. Ray Arnett,

Assistant Secretary for Fish and Wildlife and Parks.

[FR Doc. 82-10878 Filed 4-20-82; 8:45 am]

BILLING CODE 4310-70-M

Grand Canyon National Park, Arizona; Adjacent Lands Study; Notice of Availability

Notice is hereby given that the final Adjacent Lands Study for Grand Canyon National Park, prepared by the National Park Service and the Bureau of Land Management, Department of Interior and the U.S. Forest Service, Department of Agriculture, is available for public review.

This study was done in response to the Grand Canyon National Park Enlargement Act, Public Law 93-620 of January 3, 1975, and accompanying Joint Statement of Committee Conference Report #93-1611. The Committee of Conference directed the Secretary of the Interior to study certain lands adjacent to the enlarged Grand Canyon National Park and determine if they qualified for national park designation.

The final report describes the study area and evaluates resource significance and land use practices. Land management options are discussed and conclusions reported.

The study is available for distribution to all interested individuals, organizations, and government agencies.

Anyone wanting copies of the Adjacent Land Study may write to the National Park Service, Western Regional Office, 450 Golden Gate Avenue, Box 36063, San Francisco, California 94102, Attention: Grand Canyon Adjacent Lands Study.

Dated: April 8, 1982.

Howard H. Chapman,

Regional Director, Western Region, National Park Service.

[FR Doc. 82-10879 Filed 4-20-82; 8:45 am]

BILLING CODE 4310-70-M

INTERSTATE COMMERCE COMMISSION

Motor Carriers; Permanent Authority Decisions; Decision-Notice

The following applications, filed on or after February 9, 1981, ar governed by Special Rule of the Commission's Rules of Practice, see 49 CFR 1100.251. Special Rule 251 was published in the Federal Register of December 31, 1980, at 45 FR 86771. For compliance procedures, refer to the **Federal Register** issue of December 3, 1980, at 45 FR 80109.

Persons wishing to oppose an application must follow the rules under 49 CFR 1100.252. A copy of any application, including all supporting evidence, can be obtained from applicant's representative upon requst and payment to applicant's representative of $10.00.

Amendments to the request for authority are not allowed. Some of the applications may have been modified prior to publication to conform to the Commission's policy of simplifying grants of operating authority.

Findings

With the exception of those applications involving duly noted problems (e.g., unresolved common control, fitness, water carrier dual operations, or jurisdictional questions) we find, preliminarily, that each applicant has demonstrated a public need for the proposed operations and that it is fit, willing, and able to perform the service proposed, and to conform to the requirements of Title 49, Subtitle IV, United States Code, and the Commission's regulations. This presumption shall not be deemed to exist where the application is opposed. Except where noted, this decision is neither a major Federal action significantly affecting the quality of the human environment nor a major regulatory action under the Energy Policy and Conservation Act of 1975.

In the absence of legally sufficient opposition in the form of verified statements filed on or before 45 days from date of publication, (or, if the application later becomes unopposed) appropriate authorizing documents will be issued to applicants with regulated operations (except those with duly noted problems) and will remain in full effect only as long as the applicant maintains appropriate compliance. The unopposed applications involving new entrants will be subject to the issuance of an effective notice setting forth the compliance requirements which must be satisfied before the authority will be issued. Once this compliance is met, the authority will be issued.

Within 60 days after publication an applicant may file a verified statement in rebuttal to any statement in opposition.

To the extent that any of the authority granted may duplicate an applicant's other authority, the duplication shall be contrued as conferring only a single operating right.

Note.—All applications are for authority to operate as a motor common carrier in interstate or foreign commerce over irregular routes, unless noted otherwise. Applications for motor contract carrier authority are those where service is for a named shipper "under contract".

Please direct status inquiries to the Ombudsman's Office, (202) 275-7326.

Volume No. OP3-062

Decided: April 15, 1982.

By the Commission, Review Board Number 2, members Carleton, Fisher and Williams Member Fisher not participating.

MC 99775 (Sub-5), filed April 7, 1982. Applicant: TRIANGLE EXPRESS, INC., 1015 S.W. 2nd, Oklahoma City, OK 73109. Representative: William P. Parker, P.O. Box 54657, Oklahoma City, OK 73154, (405) 424-3301. Transporting *general commodities* (except classes A and B explosives), between Maize, KS and point in OK.

MC 114555 (Sub-1), filed April 9, 1982. Applicant: EUREKA-REDDING STAGES, INC., d.b.a. REDWOOD EMPIRE LINES, Post office Box 3790, Eureka, CA 95501. Representative: Eldon M. Johnson, 650 California Street, Suite 2808, San Francisco, CA 94108, (415) 986-8696. Transporting *passengers and their baggage,* in the same vehicle with passengers, in special and charter operations, beginning and ending at points in Del Norte, Humboldt, Lassen, Modoc, Shasta, Siskiyou, Tehama and Trinity Counties, CA, and Coos, Curry, Douglas, Jackson, Josephine, Klamath and Lake Counties, OR, and extending to points in the U.S. (except HI).

MC 121504 (Sub-2), filed March 16, 1982. Applicant: KEN'S TRANSFER, INC., P.O. Box 534, Milbank SD 57252. Represenative: James E. Ballenthin, 630 Osborn Bldg., St. Paul, MN 55102, (612) 227-7731. Over regular routes, transporting *general commodities* (except classes A and B explosives, household goods, and commodities in

bulk), between Watertown and Milbank, SD, serving all intermediate points and serving off-route points in Grant County, SD, from Watertown over U.S. Hwy 81 to junction SD Hwy 20, then over SD Hwy 20 to junction U.S. Hwy 77, then over U.S. Hwy 77 to Milbank, and return over the same route, and (2) over irregular routes, transporting (a) *building materials,* between Houston, TX, points in Woodford County, IL and Larimer County, CO, on the one hand, and, on the other, points in MN, MT, NE, ND, and SD, and (b) *agricultural feed systems, components and parts,* between ports of entry on the International boundary line, between the U.S. and Canada, on the one hand, and, on the other, points in MN, ND, SD, MT, NE, and IA. Condition: Coincidental cancellation of Certificate of Registration No. MC-121504 Sub-1, issued June 24, 1966, which applicant has requested in writing.

MC 121835 (Sub-1), filed April 2, 1982. Applicant: VIKING FREIGHT SYSTEM, INC., 3405 Victor Square, Santa Clara, CA 95050. Represenative: Thomas M. Loughram, 100 Bush St., 21st Fl., San Francisco, CA 94104, (415) 986-5778. Over regular routes, transporting *general commodities* (except classes A and B explosives, household goods and commodities in bulk), (1) between San Clemente, CA and junction U.S. Hwy 101 and CA Hwy 1 near Leggett, CA, over CA 1; (2) between Brookings, OR and Los Angeles, CA, over U.S. Hwy 101; (3) between the CA/NM State line and Medford, OR over Interstate Hwy 5; (4) between junction CA Hwy 99 and Interstate Hwy 5 near Mettler, CA and Red Bluff, CA, over CA Hwy 99; (5) between junction Interstate Hwy 15 and U.S. Hwy 395 near Hesperia, CA and Lakeview, OR over U.S. Hwy 395; (6) between junction CA Hwys 49 and 70 at or near Vinton, CA and Oakhurst, CA, over CA Hwy 49; (7) between junction CA Hwy 89 and Interstate Hwy 5 near Mount Shasta, CA and junction CA Hwy 89 and U.S. Hwy 395 near Topaz, CA, over Hwy 89; (8) between junction CA Hwys 29 and 20 near Upper Lake, CA and Vallejo, CA over CA Hwy 29; (9) between junction CA Hwy 70 and Interstate Hwy 5 near Sacramento, CA and junction CA Hwy 70 and U.S. Hwy 395 near Chilcoot, CA, over CA Hwy 70; (10) between Santa Cruz, CA and San Rafael, CA, over CA Hwy 17; (11) between San Jose, CA and junction Interstate Hwys 680 and 80 near Cordelia, CA, over Interstate Hwy 680; (12) between Yosemite Village, CA and Fresno, CA, over CA Hwy 41; (13) between junction CA Hwy 99 and 65 near Bakersfield, CA and junction CA Hwys 65 and 198 near Visalia, CA, over CA Hwy 65; (14) between junction CA Hwy 14 and Interstate Hwy 5 near San Fernando, CA and junction CA Hwy 14 and U.S. Hwy 395 near Little Lake, CA, over CA Hwy 14; (15) between San Diego, CA and Las Vegas, NV over Interstate Hwy 15; (16) between Calexico, CA and junction Interstate Hwy 10 and CA Hwy 86 near Indio, CA, over CA Hwy 86; (17) between San Diego, CA and junction Interstate Hwy 8 and Interstate Hwy 10 near Casa Grande, AZ, over Interstate Hwy 8; (18) between Santa Monica, CA and Tucson, AZ, over Interstate Hwy 10 and AZ Hwy 85; (19) between junction Interstate Hwys 15 and 40 and Topock, AZ, over Interstate Hwy 40; (20) between junction CA Hwy 198 and U.S. Hwy 101 near San Lucas and Three Rivers, CA, over CA Hwy 198; (21) between Watsonville, CA and junction CA Hwys 152 and 99 near Fairmead, over CA Hwy 152; (22) between Hayward, CA and junction Interstate Hwys 205 and 5 near Banta, CA, from Hayward over Interstate Hwy 580 to junction Interstate Hwy 205, then over Interstate Hwy 205 to junction Interstate Hwy 5, and return over the same route; (23) between junction Interstate Hwy 5 and CA Hwy 120 near Banta, CA and junction CA Hwys 120 and 140 near Yosemite Village, CA over CA Hwy 120; (24) between Hercules, CA and junction CA Hwys 4 and 89 near Markleeville, CA, over CA Hwy 4; (25) between San Francisco, CA and junction Interstate Hwy 80 and U.S. Hwy 50 Alt. near Fernley, NV, over Interstate Hwy 80; (26) between Sacramento, CA and Fallon, NV, over U.S. Hwy 50; (27) between Noyo, CA and junction CA Hwy 20 and Interstate Hwy 80 near Emigrant Gap, CA, over CA Hwy 20; (28) between Arcata, CA and Alturas, CA, over CA Hwy 299; (29) between Las Vegas, NV and Fallon, NV, over U.S. Hwy 95; (30) between junction U.S. Hwys 50 and Alt. near Fallon, NV and junction U.S. Hwy 50 Alt. and Interstate Hwy 80 near Fernley, NV, over U.S. Hwy 50 Alt.; serving all intermediate points on routes (1) through (30) above, and serving points in CA as off-route points, over irregular routes, transporting *general commodities* (except classes A and B explosives, household goods and commodities in bulk), between points in AZ, CA, CO, ID, MT, NV, NM, OR, TX, UT, WA and WY.

Note.—Coincidental cancellation of Certificate of Registration No. MC-121835, served May 27, 1981, which applicant has requested in the application.

MC 124965 (Sub-8), filed April 6, 1982. Applicant: OIL TRANSPORT, INC., 4419 Bainbridge Boulevard, Chesapeake, VA 23320. Representative: Blair P. Wakefield, Suite 1001, First and Merchants National Bank Bldg., Norfolk, VA 23510. Transporting *petroleum and petroleum products,* between points in NJ and PA, on the one hand, and, on the other, points in AL, DE, FL, GA, KY, MD, MS, NC, NJ, OH, PA, SC, TN, VA, WV and DC.

MC 138184 (Sub-4), filed April 9, 1982. Applicant: WALLACE TRUCKING COMPANY, Route 4, Box A-71, Laurinburg, NC 28352. Representative: F. Kent Burns, P.O. Box 2479, Raleigh, NC 27602, (919) 828-2421. Transporting *natural and synthetic yarn fiber and textile mill products including finished yarn products,* between points in Alamance, Anson, Suncombe, Chatham, Cleveland, Cumberland, Gaston, Guilford, Lenoir, Mecklenburg, Montgomery and Scotland Counties, NC, on the one hand, and, on the other, points in AL, AR, CA, CT, FL, GA, IL, IN, IA, KY, MD, MS, NJ, NC, OH, PA, SC, TN, TX, VA and WV.

MC 141914 (Sub-108), filed April 9, 1982. Applicant: FRANKS AND SON, INC., Route 1, Box 108A, Big Cabin, OK 74332. Representative: Kathrena J. Franks (same address as applicant), (918) 783-5180. Transporting *general commodities* (except classes A and B explosives, household goods, and commodities in bulk), between the facilities of Borden, Inc., at points in the U.S. (except AK and HI), on the one hand, and, on the other, points in the U.S. (except AK and HI).

MC 144645 (Sub-13), filed April 9, 1982. Applicant: ROBERT HANSEN TRUCKING, INC., Rt. 2, Box 125, Delavan, WI 53115. Representative: Daniel R. Dineen, 710 No. Plankinton Ave., Milwaukee, WI 53203, (414) 273-7410. Transporting *food and related products,* between points in the U.S. (except AK and HI), under continuing contract(s) with Merkt Enterprises, Inc. of Salem, WI.

MC146605 (Sub-9), filed April 6, 1982. Applicant: EVENSON BROS., INC., P.O. Box 328, Pelican Rapids, MN 56572. Representative: Thomas J. Van Osdel, 15 Broadway-Suite 502, Fargo, ND 58102, (701) 235-4487. Transporting (1) *metal, plastic, stone, clay, glass and concrete products, machinery and drainage systems,* between points in MN, MT, ND AND SD, on the one hand, and, on the other, point in the U.S. (except AK and HI) and (2) *metal products, hardware, automotive parts, recycled metal and construciton materials,* (a) between points in ID, MT, OR, UT, WA and WY and (b) between points in ID, MT, OR,

UT, WA, and WY, on the one hand, and, on the other, in the U. S. (except AK and HI).

MC151655 (Sub-12), filed April 8, 1982. Applicant: FRANK BROS. TRUCKING CO., P.O. Box 241 349 Abbott Ave., Hillsboro, TX 76645. Representative: Charles E. Munson, 500 W. Sixteen St., P.O. Box 1945, Austin TX 78767, (512) 478-9808. Transporting *transportation equipment*, between points in McLennan County, TX, on the one hand, and, on the other, points in the U. S. (except AK and HI).

MC153664 (Sub-1), filed April 9, 1982. Applicant: DAVID A. LUNDEEN, d.b.a. FARGO FREIGHT TERMINAL & WAREHOUSE, 1445 5th Ave., North, P.O. Box 1828, Fargo, ND 58102. Representative: Richard P. Anderson, P.O. Box 2581, Fargo, ND 58108, (701) 235-3300. Transporting *general commodities* (except classes A and B explosives, household goods, and commodities in bulk), between points in the U. S. (except AK and HI), under continuing contract(s) with Dry Storage Corporation, of Chicago, IL.

MC161415, filed April 8, 1982. Applicant: F. M. TRANSPORT, INC., 1781 Schiller Rd., Portage, IN 46368. Representative: Joel H. Steiner, 29 South LaSalle St., Suite 905, Chicago, IL 60603, (312) 236-9375. Transporting *metal products*, between points in MI, IL, IN, and OH.

MC161424, filed April 9, 1982. Applicant: PARCEL DELIVERY COMPANY, INC., 2020 Warehouse Rd., Normal, IL 61761. Representative: Guy H. Postell, Suite 675, 3384 Peachtree Rd., N.E., Atlanta, GA 303026, (404) 237-6472. Transporting *general commodities* (except classes A and B explosives, household goods, and commodities in bulk), between points in the U. S. (except AK and HI), under continuing contract(s) with Avon Products, Inc., of New York, NY.

Volume No. OP4-134

Decided: April 13, 1982.

By the Commission, Review Board No. 2, Members Carleton, Fisher and Williams. Member Fisher not participating.

MC 70267 (Sub-22), filed April 6, 1982. Applicant: ECKERT TRUCKING, INC., 1090 E. Springettsbury Ave., York, PA 17405. Representative: David Zimmerman (same address as applicant), (717) 843-0995. Transporting *clay, concrete, glass or stone products*, between Baltimore, MD, Chesapeake, VA, and points in Logan County, KY and Sandusky County, OH, on the one hand, and, on the other those points in the U.S. in and east of WI, IL, KY, TN, and MS.

MC 139457 (Sub-31), filed April 2, 1982. Applicant: G. L. SKIDMORE d.b.a. JELLY SKIDMORE TRUCKING CO., P.O. Box 38, Paris, TX 75460. Representative: Paul D. Angenend, P.O. Box 2207, Austin, TX 78768, (512) 476-6391. Transporting *food and related products*, between points in the U.S., under continuing contract(s) with Campbell Soup Company of Camden, NJ.

MC 139457 (Sub-32), filed April 2, 1982. Applicant: G. L. SKIDMORE d.b.a. JELLY SKIDMORE TRUCKING CO., P.O. Box 38, Paris, TX 75460. Representative: Paul D. Angenend, P.O. Box 2207, 1806 Rio Grande, Austin, TX 78768, (512) 476-6391. Transporting *food and related products*, between points in the U.S. under continuing contract(s) with Anderson Clayton Foods of Houston, TX.

MC 148657 (Sub-2), filed April 6, 1982. Applicant: RAYMOND & HIGGINS TRANSPORTATION CO., INC., 78/80 Judith St., Providence, RI 02909. Representative: Robert B. Walker, 915 Pennsylvania Bldg., 425 13th St., NW., Washington, DC 20004, (202) 737-1030. Transporting (1) *petroleum and petroleum products*, and (2) *chemicals*, between points in NJ, on the one hand, and, on the other those points in MA, CT, and RI.

MC 149497 (Sub-25), filed April 6, 1982. Applicant: HAUPT CONTRACT CARRIERS, INC., P.O. Box 1023, Wausau, WI 54401. Representative: Robert A. Wagman (same address as applicant), (715) 359-2907. Transporting *general commodities* (except classes A and B explosives, household goods, and commodities in bulk), between points in the U.S., under continuing contract(s) with Harnischfeger Corporation of Milwaukee, WI.

MC 150017 (Sub-4), filed April 2, 1982. Applicant: DELICIOUS FOODS CARRIERS, INC., P.O. Box 730, Grand Island, NE 68801 Representative: Jack L. Shultz, P.O. Box 82028, Lincoln, NE 68501, (402) 475-6761. Transporting *general commodities* (except classes A and B explosives, household goods, and commodities in bulk), between points in the U.S., under continuing contract(s) with Wetterau, Inc., of Hazelwood, MO, and its subsidiaries of Golden Dipt, Division of DCA Foods, Inc., of East St Louis IL, Roland Industries, Inc., of St. Louis, MO, Tasty-Toppings, Inc., of Columbus, NE, Keystone Mushroom Sales, of Avondale, PA, and Hanover Brands, Inc., of Hanover, PA.

MC 150907, filed March 30, 1982. Applicant: BERNARD C. WESSELING d.b.a. BERNHAVEN SOD SUPPLY, R.R. 2, Bowmanville, Ontario, CN L1C 3K3. Representative: Robert D. Gunderman, Can-Am Bldg., 101 Niagara St, Buffalo, NY 14202, (716) 854-5870. Transporting *fertilizer and chemicals and related products* between points in the U.S., under continuing contract(s) with Chipman, Inc., of Stoney Creek, Ontario, Canada.

MC 152277 (Sub-1), filed April 5, 1982. Applicant: LONG MILE RUBBER COMPANY, 6820 Forest Park, Exchange Park, Dallas, TX 75245. Representative: D. Paul Stafford, P.O. Box 45538, Dallas, TX 75245. Transporting *paper and paper products*, between points in the U.S., under continuing contract(s) with Sonoco Products Company, of Hartsville, SC.

MC 160017 (Sub-1), filed April 5, 1982. Applicant: QUALITY SUPPLIER TRUCKING, INC., Keyser Industrial Park, Keyser, WV 26726. Representative: Dixie C. Newhouse, 1329 Pennsylvania Ave., P.O. Box 1417, Hagerstown, MD 21740, (301) 797-6060. Transporting *recyclable materials, and paper and paper products*, between points in MD, DE, NY, ME, PA, OH, VA, WV, NC, MA, and DC.

MC 161347, filed April 1, 1982. Applicant: TRI-ME TRANSPORTATION, a Division, of Triangle Metallurgical, Inc., 1559 A State, P.O. Box 45, Granite City, IL 62040. Representative: Michael W. O'Hara, 300 Reisch Bldg., Springfield, IL 62701. (217) 544-5468.Transporting *metal and metal products*, between points in the U.S., under continuing contract(s) with L. C. Metals, Inc., of Granite City, IL, and Chemetco, Inc., of Hartford, IL.

MC 161377, filed April 6, 1982. Applicant: JAG TRUCKING, INC., 6250 N.W. 74th Ave., Miami, FL 33166. Representative: Jeffrey W. Kohlman, 3390 Peachtree Rd., N.E., Suite 520, Atlanta, GA 30326, (404) 262-7855. Transporting *general commodities* (except classes A and B explosives, household goods, and commodities in bulk), between Atlanta, GA, on the one hand, and, on the other, points in the U.S. (except AK and HI).

MC 161387, filed April 6, 1982. Applicant: DOUGLAS C. ROYCRAFT, d.b.a. ROYCRAFT TRANSIT & STORAGE, 1800 Vernon St., Eau Claire, WI 54701. Representative: Robert S. Lee, 1600 TCF Tower, 121 S. 8th St., Minneapolis, MN 55402, (612) 333-1341. Transporting *food and related products, and matches*, between Chicago, IL, and Memphis, TN, and points in Stark County, OH, and Gibson County, TN, and points in WI, on the one hand, and, on the other, points in CA, CO, FL, GA,

IL, LA, MN, MO, NE, NJ, NC, OH, PA, TN, TX, and WI.

Volume No. OP4-137

Decided: April 15, 1982.

By the Commission, Review Board Number 2, Members Carleton, Fisher and Williams. Members Fisher not participating.

MC 152376 (Sub-2), filed April 7, 1982. Applicant: TABLOID SHIPPERS, INC., 1101 Tonnele Ave., N. Bergen, NJ 07047. Representative: William J. Augello, 120 Main St., Huntington, NY 11743, (516) 427-0100. Transporting *printed matter*, between points in NH, NJ and WI, on the one hand, and, on the other, points in the U.S. (except AK and HI).

MC 152706 (Sub-4), filed April 5, 1982. Applicant: MIDWEST OIL TRANSIT, INC., P.O. Box 68083, Indianapolis, IN 46268. Representative: Robert B. Hebert, 777 Chamber of Commerce Bldg., 320 N. Meridian St., Indianapolis, IN 46204, (317) 639-4511. Transporting *petroleum products and coal products*, between points in Crawford County, IL, on the one hand, and, on the other, points in Vigo, Putnam, and Hendricks Counties, IN, and Indianapolis, IN.

MC 154346 (Sub-2), filed April 6, 1982. Applicant: ERNEST RYLIE, JR., d.b.a. RYLIE TRUCKING COMPANY, 3105 N. Hwy. 75, Corsicana, TX 75110. Representative: J. Michael Alexander, 5801 Marvin D. Love Freeway, Suite 301, Dallas, TX 75237-2385, (214) 339-4108. Transporting *clay, concrete, glass or stone products*, between in TX, OK, NM, AR, LA, KS, MO, TN, MS, and AL.

MC 159826, filed March 23, 1982. Applicant: PACELLA TRUCKING EXPRESS, 3521 S. Morgan St., Chicago, IL 60609. Representative: Gilbert J. Green, 5957 S. Rutherford Ave., Chicago, IL 60638, (312) 254-4500. Transporting *general commodities* (except classes A and B explosives, household goods, and commodities in bulk), between Chicago, IL, on the one hand, and, on the other points in IL.

MC 161346, filed April 2, 1982. Applicant: FTL COMPANY, INC., 4632 Sheila St., City of Commerce, CA 90040. Representative: Ronald N. Cobert, 1730 M St., NW, Suite 501, Washington, DC 20036, (202) 296-2900. Transporting *general commodities* (except classes A and B explosives, household goods, and commodities in bulk), between points in AZ, CA, and NV.

MC 161106, filed April 5, 1982. Applicant: POWERS TRANSPORT, LTD., 937 Fleury St., Regina, Saskatchewan, CD S4N 4W7. Representative: Daniel O. Hands, 205 W. Touhy Ave., Suite 200A, Park Ridge, IL 60068, (312) 698-2235. Transporting *food and related products*, between Baltimore, MD and Philadelphia, PA, on the one hand, and, on the other, those points on the International Boundary line between the U.S. and Canada located at points in MT, ND, and MN.

MC 161356, filed April 5, 1982. Applicant: DES-LAN TRUCKING, INC., 67911 State Road 23, N Liberty, IN 46544. Representative: Donald W. Smith, P.O. Box 40248, Indianapolis, IN 46240, (317) 846-6655. Transporting *building materials*, between points in St. Joseph and LaPorte Counties, IN, on the one hand, and, on the other, points in the U.S. in and east of ND, SD, NE, CO, OK and TX.

MC 161376, filed April 5, 1982. Applicant: TRUCK TRANSFER SERVICE, INC., 8013 Horse Chestnut Lane, Charlotte, NC 28230. Representative: Frank A. Graham, Jr., P.O. Box 11864, Columbia, SC 29211, (803) 799-9122. Transporting *motor vehicles*, in driveaway service, between points in Allen County, IN and Clark County, OH, on the one hand, and, on the other, points in NC and SC.

MC 161396, filed April 6, 1982. Applicant: BLAINE WEBB, d.b.a. GOLDEN WEST LIVESTOCK, P.O. Box 271, Heber City, UT 84032. Representative: Juanita Webb (same address as applicant), (801) 654-2364. Transporting *such commodities* as are dealt in by grocery and food business houses, between points in UT, NV, AZ, CA, and ID.

Agatha L. Mergenovich,

Secretary.

[FR Doc. 82-10840 Filed 4-20-82; 8:45 am]

BILLING CODE 7035-01-M

Motor Carriers; Permanent Authority Decisions; Decision-Notice

The following applications, filed on or after February 9, 1981, are governed by Special Rule of the Commission's Rules of Practice, see 49 CFR 1100.251. Special Rule 251 was published in the **Federal Register** on December 31, 1980, at 45 FR 86771. For compliance procedures, refer to the **Federal Register** issue of December 3, 1980, at 45 FR 80109.

Persons wishing to oppose an application must follow the rules under 49 CFR 1100.252. Applications may be protested *only* on the grounds that applicant is not fit, willing, and able to provide the transportation service or to comply with the appropriate statutes and Commission regulations. A copy of any application, including all supporting evidence, can be obtained from applicant's representative upon request and payment to applicant's representative of $10.00.

Amendments to the request for authority are not allowed. Some of the applications may have been modified prior to publication to conform to the Commission's policy of simplifying grants of operating authority.

Findings

With the exception of those applications involving duly noted problems (e.g., unresolved common control, fitness, water carrier dual operations, or jurisdictional questions) we find, preliminarily, that each applicant has demonstrated a public need for the proposed operations and that it is fit, willing, and able to perform the service proposed, and to conform to the requirements of Title 49, Subtitle IV, United States Code, and the Commission's regulations. This presumption shall not be deemed to exist where the application is opposed. Except where noted, this decision is neither a major Federal action significantly affecting the quality of the human environment nor a major regulatory action under the Energy Policy and Conservation Act of 1975.

In the absence of legally sufficient opposition in the form of verified statements filed on or before 45 days from date of publication (or, if the application later become unopposed), appropriate authorizing documents will be issued to applicants with regulated operations (except those with duly noted problems) and will remain in full effect only as long as the applicant maintains appropriate compliance. The unopposed applications involving new entrants will be subject to the issuance of an effective notice setting forth the compliance requirements which must be satisfied before the authority will be issued. Once this compliance is met, the authority will be issued.

Within 60 days after publication an applicant may file a verified statement in rebuttal to any statement in opposition.

To the extent that any of the authority granted may duplicate an applicant's other authority, the duplication shall be construed as conferring only a single operating right.

Note.—All applications are for authority to operate as a motor common carrier in interstate or foreign commerce over irregular routes, unless noted otherwise. Applications for motor contract carrier authority are those where service is for a named shipper "under contract".

Please direct status inquiries to the Ombudsman's Office, (202) 275-7326.

Volume No. OPI–64

Decided: April 14, 1982.

By the Commission, Review Board Number 1, Members Parker, Chandler and Fortier.

MC 159701 (Sub-2), filed April 2, 1982. Applicant: TRU-MORE TRUCKING CO., INC., P.O, Box 365, Roosevelt, NY 11575. Representative: Jack L. Schiller, 123–60 83rd Ave., Kew Gardens, NY 11415, (212) 263–2078. Transporting (1) for or on behalf of the United States Government, *general commodities* (except used household goods, hazardous or secret materials, and sensitive weapons and munitions); and (2) *used household goods* for the account of the United States Government incident to the performance of a pack-and-crate service on behalf of the Department of Defense, between points in the U.S. (except AK and HI).

MC 161311, filed April 1, 1982. Applicant: DEAN ALLEN, 12382 South Mulino Rd, Canby, OR 97013. Representative: Dean Allen (same address as applicant). Transporting *food and other edible products and byproducts intended for human consumption* (except alcoholic beverages and drugs), *agricultural limestone and fertilizers, and other soil conditioners* by the owner of the motor vehicle in such vehicle, between points in the U.S. (except AK and HI).

MC 161320, filed April 1, 1982. Applicant: MICHAEL P. TUTTLE, d.b.a. T & T TRUCKING, P.O. Box 77, North Bend, WA 98045. Representative: Jim Pitzer, 15 S. Grady Way, Suite 321, Renton, WA 98055–3273, (206) 235–1111. Transporting *food and other edible products and byproducts intended for human consumption* (except alcoholic beverages and drugs), *agricultural limestone and fertilizers and other soil conditioners* by the owner of the motor vehicle in such vehicle, between points in the U.S. (except AK and HI).

MC 161321, filed April 1, 1982. Applicant: KEN JUNEAU, INC., Route 1, Box 533, Simmesport, LA 71369. Representative: Andrew V. Baylor, 337 East Elm Street, Phoenix, AZ 85012, (602) 274–5146. Transporting *food and other edible products and byproducts intended for human consumption* (except alcoholic beverages and drugs), *agricultural limestone and fertilizers, and other soil conditioners* by the owner of the motor vehicle in such vehicle, between points in the U.S. (except AK and HI).

Volume No. OP2–73

Decided: April 8, 1982.

By the Commission, Review Board Number 1, Members Parker, Chandler and Fortier.

MC 161203, filed March 24, 1982. Applicant: ROBERT E. GUNTER, d.b.a. B. G. TRUCKING, 7748 Trails End Dr. SE., Olympia, WA 98501. Representative: Robert E. Gunter, (same address as applicant), (206) 943–3329. Transporting *food and other edible products and byproducts intended for human consumption* (except alcoholic beverages and drugs), *agricultural limestone and fertilizers, and other soil conditioners* by the owner of the motor vehicle in such vehicle, between points in the U.S. (except AK and HI).

MC 161233, filed March 29, 1982. Applicant: SIMON BROS., INC., 468 N. Maple, Box 116, Fowler, MI 48835. Representative: Dennis J. Simon, 534 N. Walnut St., Fowler, MI 48835, (517) 593–3288. Transporting *food and other edible products and byproducts intended for human consumption,* (except alcoholic beverages and drugs), *agricultural limestone and fertilizers, and other soil conditioners* by the owner of the motor vehicle in such vehicle, between points in the U.S. (except AK and HI).

Volume No. OP3–059

Decided: April 14, 1982.

By the Commission, Review Board Number 2, Members Carleton, Fisher and Williams. Member Fisher not participating.

MC 161304, filed March 31, 1982. Applicant: SUPERIOR DELIVERY & MESSENGER SERVICE, INC., 247 Hope St., Stamford, CT 06906. Representative: John D. Heffner, 1776 K St., NW., Suite 700, Washington, D. C. 20006, (202) 296–0600. Transporting *shipments weighing 100 pounds or less* if transported in a motor vehicle in which no one package exceeds 100 pounds, between points in the U. S. (except AK and HI).

MC 161354, filed April 5, 1982. Applicant: INTERNATIONAL FORWARDERS, INC., 1122 Morrison Drive, P.O. Box 550, Charleston, SC 29402. Representative: A. N. Manucy, Jr., (same address as applicant), (803) 722–2731. As a *broker of general commodities* (except household goods), between points in the U. S. (except AK and HI).

MC 161355, filed April 5, 1982. Applicant: JOHN E. LANE d.b.a. LANE TRUCKING, 6200 Edinger Ave., No. 508, Huntington Beach, CA 92647. Representative: John E. Lane (same address as applicant), (714) 840–9430 Transporting *food and other edible products and byproducts intended for human consumption* (except alcoholic beverages and drugs), *agricultural limestone and fertilizers,* and *other soil conditioners,* by the owner of the motor vehicle in such vehicle, between points in the U.S.

Volume No. OP4–135

Decided: April 13, 1982.

By the Commission, Review Board Number 2, Members Carleton, Fisher and Williams. Member Fisher not participating.

MC 161327, filed April 2, 1982. Applicant: DAVID F. YOUNGBLOOD, d.b.a. NEWS TRANSPORT, 10810 Heeter Rd., Brookville, OH 45309. Representative: Eric Meierhoefer, Suite 1000, 1029 Vermont Ave., NW., Washington, DC 20005, (202) 347–9332. Transporting (1) for or on behalf of the United States Government, *general commodities* (except used household goods, hazardous or secret materials, and sensitive weapons and munitions), between points in the U. S. (except AK and HI), and (2) as a *broker of general commodities* (except household goods), between points in the U. S. (except AK and HI).

Volume No. OP4–136

Decided: April 15, 1982.

By the Commission, Review Board Number 2, Members Carleton, Fisher and Williams. Member Fisher not participating.

MC 158286 (Sub-7), filed April 5, 1982. Applicant: M. T. TRUCK LINE, INC., 4947 W. 173rd St., Country Club Hills, IL 60477. Representative: James C. Hardman, 33 N. LaSalle St., Chicago, IL 60602, (312) 236–5944. Transporting *general commodities* (except classes A and B explosives, household goods, and commodities in bulk), between Truro and Whitehall, OH, on the one hand, and, on the other, points in the U. S. (except AK and HI). Condition: Issuance of a certificate in this proceeding is conditioned upon applicant certifying to the Commission, prior to commencing operations, that all rail service has actually terminated at specified points. The certification should be sent to the Deputy Director, Section of Operating Rights, Interstate Commerce Commission, Washington, DC 20423.

MC 161286, filed March 30, 1982. Applicant: FRONTIER TRANSPORTATION INC., 2422 Highway 2 West, Kalispell, MT 59901. Representative: Kenneth P. Doty (same address as applicant), (406) 755–6565. As a *broker of general commodities* (except household goods), between points in the U.S. (except AK and HI).

Volume No. OP5–84

Decided: April 13, 1982.

By the Commission, Review Board No. 3, Members Krock, Joyce, and Dowell.

MC 140898 (Sub-13), filed April 1, 1982. Applicant: KENDRICK TRUCKING CORP., 728 Upsliner Rd., Louisville, KY

40213. Representative: Fred F. Bradley, P.O. Box 773, Frankfort, KY 40602, (502) 227-2254. Transporting (1) *shipments weighing 100 pounds or less* if transported in a motor vehicle in which no one package exceeds 100 pounds, and (2) for or on behalf of the United States Government, *general commodities* (except used household goods, hazardous or secret materials, and sensitive weapons and munitions), between points in the U.S. (except AK and HI).

MC 161228, filed March 26, 1982. Applicant: LINE HAUL SERVICES, INC., 699 Hwy 203, East St. Louis, IL 62201. Representative: Lawrence Marquette, P.O. Box 629, Carmel Valley, CA 93924, 408-625-2031. (1) Transporting for or on behalf of the United States Government, *general commodities* (except used household goods, hazardous or secret materials, and sensitive weapons and munitions), between points in the U.S. (except AK and HI), and (2) as a *broker of general commodities* (except household goods), between points in the U.S. (except AK and HI).

MC 161249, filed March 29, 1982. Applicant: NIEDERER TRUCKING, RT. 3 Box 237, Rexburg, ID 83440. Representative: Terrel Niederer (same address as applicant), 208-356-6318. Transporting *food and other edible products and byproducts intended for human consumption* (except alcoholic beverages and drugs), *agricultural limestone and fertilizers, and other soil conditioners*, by the owner of the motor vehicle in such vehicle, between points in the U.S. (including AK but excluding HI).

MC 161328, filed April 1, 1982. Applicant: PORT JERSEY DISTRIBUTION SERVICES, INC., 2 Colony Rd., Jersey City, NJ 07305. Representative: Charles J. Williams, P.O. Box 186, Scotch Plains, NJ 07076, (201) 322-5030. To operate as a *broker* of *general commodities* (except household goods), between points in the U.S., (including AK, but excluding HI).

[FR Doc. 82-10841 Filed 4-20-82; 8:45 am]

BILLING CODE 7035-01-M

Motor Carrier Temporary Authority Applications

The following are notices of filing of applications for temporary authority under Section 10928 of the Interstate Commerce Act and in accordance with the provisions of 49 CFR 1131.3. These rules provide that an original and two (2) copies of protest to an application may be filed with the Regional Office named in the **Federal Register** publication no later than the 15th calendar day after the date the notice of the filing of the application is published in the **Federal Register**. One copy of the protest must be served on the applicant, or its authorized representative, if any, and the protestant must certify that such service has been made. The protest must indentify the operating authority upon which it is predicated, specifying the "MC" docket and "Sub" number and quoting the particular portion of authority upon which it relies. Also, the protestant shall specify the service it can and will provide and the amount and type of equipment it will make available for use in connection with the service contemplated by the TA application. The weight accorded a protest shall be governed by the completeness and pertinence of the protestant's information.

Except as otherwise specifically noted, each applicant states that there will be no significant effect on the quality of the human evironment resulting from approval of its application.

A copy of the application is on file, and can be examined at the ICC Regional Office to which protest are to be transmitted.

Note.—All applications seek authority to operate as a common carrier over irregular routes except as otherwise noted.

Motor Carrier of Property

Notice No. F-164

The following applications were filed in Region I: Send protests to: Interstate Commerce Commission, Regional Authority Center, 150 Causeway Street, Room 501, Boston, MA 02114.

MC 134806 (Sub-1-28TA), filed April 8, 1982. Applicant: B-D-R TRANSPORT, INC., Vernon Drive, P.O. Box 1277, Brattleboro, VT 05301. Representative: Edward T. Love, 4401 East Highway, Suite 404, Bethesda, MD 20814. *Contract carrier:* irregular routes: *Maple syrup, syrup products, and supplies* from Jacksonville and Brattleboro, VT to points in AZ, CA, CO, ID, MT, NM, NV, OR, UT, WA and WY, under continuing contact(s) with Coombs Maple Products, Inc., Jacksonville, VT. Supporting shipper: Coombs Maple Products, Inc., Jacksonville, VT 05342.

MC 153279 (Sub-1-4TA), filed April 9, 1982. Applicant: BONWAY SERVICE TRANSPORT, INC., 54 Fulton Street, Buffalo, NY 14204. Representative: Anthony J. Zaleski, (same as applicant). *Food and related products,* between points in CT, IL IN, IA, MA, MI, MN, NJ, NY, OH, PA, RI, and WI. Supporting shipper: Otto Brehm Inc., 75 Tuckahoe Road, Yonkers, NY 10710.

MC 144598 (Sub-1-8TA), filed April 12, 1982. Applicant: C & J TRANSPORT, INC., P.O. Box 42, Rt. 32, N. Vassalboro, ME 04962. Representative: Chester A. Zyblut, 366 Executive Bldg., 1030 15th Street, N.W., Washington, D.C. 20005. *Building materials,* between Broward County, FL, on the one hand, and, on the other, points in the U.S. located in and east of MN, IA, MO, AR, and LA. Supporting shipper: Kraco, Inc., 1020 Southwest 69th Ave., Miami, FL 33144.

MC 161435 (Sub-1-1TA), filed April 9, 1982. Applicant: CABLE TRANSPORT CORPORATION, 421 Ridge Street, Rome, NY 13440. Representative: Eugene D. Anderson, 1001 Connecticut Avenue, N.W., Suite 838, Washington, D.C. 20036. *Contract carrier:* irregular routes: *(1) Cable,* from Rome, NY to points in and east of AR, IL, LA, MO; *(2) Materials and supplies used in the manufacture of cable,* from CA, KY, NH, NJ, NY, TX to Rome, NY, under continuing contract(s) with Rome Cable Corporation, Rome, NY. Supporting shipper: Rome Cable Corporation, 421 Ridge Street, Rome, NY. 13440.

MC 161491 (Sub-1-1TA), filed April 13, 1982. Applicant: DATA SECURITIES COURIER CORP., 526 Revere Beach Blvd., Revere, MA 02151. Representative: James R. Fitzpatrick, 516 Revere Beach Blvd., Revere, MA 02151. *Contract carrier:* irregular routes: *Data processing forms and magnetic tapes,* from Boston, MA to points in CT, NY and NH, under continuing contract(s) with State Street Bank & Trust Co., Boston, MA. Supporting shipper: State Street Bank & Trust Co., 225 Franklin Street, Boston, MA.

MC 144424 (Sub-1-1TA), filed April 8, 1982. Applicant: ROBERT M. DEJONGE, R.D. #1, Beaver Hill Road, Wellsville, NY 14895. Representative: Raymond A. Richards, 35 Curtice Park, Webster, NY 14580. *Such commodities as are dealt in by grocery and food business houses, and agricultural products, not otherwise exempt, (except commodities in bulk)* between points in the U.S. in and east of MN, IA, MO, AR and TX. Supporting shipper(s): Olean Wholesale Grocery Co-op, Inc., Haskell Road, Olean, NY 14760; Castle & Cooke Foods, Inc., 330 Vanderbilt Motor Parkway, Hauppauge, NY 11788.

MC 150559 (Sub-1-4TA), filed April 9, 1982. Applicant: EMERSON EXPRESS CO., INC., 545 Lyell Avenue, P.O. Box 8008, Rochester, NY 14606. Representative: Raymond A. Richards, 35 Curtice Park Webster, NY 14580. *Electric storage batteries, spent,* from points in OH, VA, WV, MD, DE, PA, NJ, CT, RI, MA, VT, and NC to Middletown

(Orange County), NY. Supporting shipper: RSR Corporation, 1111 W. Mockingbird Lane, Dallas, TX 75247.

MC 139123 (Sub-1-1TA), filed April 8, 1982. Applicant: GLOUCESTER DISPATCH, INC., R.F.D. #2, Rt. 114, P.O. Box 124, Weare, NH 03281. Representative: Robert L. Cope; Suite 501, 1730 M Street, NW., Washington, D.C. 20036. *General commodities (except classes A and B explosives, household goods and commodities in bulk and hazardous waste)*, between Rockingham, Hillsborough and Strafford Counties, NH, Essex, Norfolk and Suffolk Counties, MA, and York County, ME, on the one hand, and, on the other, Los Angeles, San Francisco, CA; Denver, CO; Chicago, IL; New Orleans, LA; St. Louis, MO; Portland, OR; Dallas and Houston, TX; Seattle, WA; Miami, FL; Birmingham, AL; Cleveland, OH; and Atlanta, GA. Supporting shipper(s): There are 11 statements of support attached to this application which may be examined at the Regional Office of the I.C.C. in Boston, MA.

MC 16111 (Sub-1-1TA), filed April 12, 1982. Applicant: GROSS TRANSPORTATION CORPORATION, 201 Bay Avenue, Elizabeth, NJ 07201. Representative: Charles J. Williams, P.O. Box 186, Scotch Plains, NJ 07076. *Contract carrier:* irregular routes: *Such commodities as are dealt in or used by manufacturers or distributors of hospital and health care products*, between points in the U.S. under continuing contract(s) with C. R. Bard, Inc., of Murray Hill, NJ. Supporting shipper: C. R. Bard, Inc., 731 Central Avenue, Murray Hill, NJ 07971.

MC 99273 (Sub-1-3TA), filed April 8, 1982. Applicant: KINDLE TRUCKING CO., INC., 449 Silver Street, P.O. Box 311, Agawam, MA 01001. Representative: David M. Marshall, Marshall and Marshall, 101 State Street—Suite 304, Springfield, MA 01103. *Contract carrier:* irregular routes: *General commodities (except Classes A and B explosives, hazardous wastes, commodities in bulk and household goods)*, between Agawam, MA and Enfield, CT, on the one hand, and, on the other, points in AL, CA, GA, FL, IL, KS, MS, NV, NC, SC, TN, MO and TX, under continuing contract(s) with United Consolidation, Inc., of Agawam, MA. Supporting shipper: United Consolidation, Inc., 449 Silver Street, Agawam, MA 01001.

MC 147101 (Sub-1-1TA), filed April 13, 1982. Applicant: LDF, INC., 30 Enterprise Avenue, Secaucus, NJ 07094. Representative: Thomas B. Hill, 1010 Jorie Blvd., Suite 200, Oak Brook, IL 60521. *Contract carrier:* irregular routes: *Foodstuffs in vehicles equipped with mechanical refrigeration* from Atlanta, GA to Charlotte, NJ, under continuing contract(s) with Atlanta Bonded Warehouse Corp., Atlanta, GA. Supporting shipper: Atlanta Bonded Warehouse Corp., 1500 Southland Circle, N.W., Atlanta, GA 30325.

MC 161449 (Sub-1-1TA), filed April 12, 1982. Applicant: GEORGE LOGAN, d.b.a. GEORGE LOGAN'S TOWING SERVICE, Route 130, North Brunswick, NJ 08902. Representative: Robert B. Pepper, 168 Woodbridge Avenue, Highland Park, NJ 08904. *Disabled and wrecked vehicles* between NJ, on the one hand, and, on the other, points in CT, DE, DC, ME, MD, MA, NH, NY, NC, OH, PA, RI, VT, VA and WV. Supporting shipper(s): Rimar Transport, 827 Ridgewood Ave., North Brunswick, NJ; ACF Selover Trans., 393 Turnpike, South River, NJ; Hermann Forwarding Company, P.O. Box 1, North Brunswick, NJ 08902; Rutgers Express, 6 Commertz Road, East Brunswick, NJ.

MC 161448 (Sub-1-1TA), filed April 12, 1982. Applicant: NACKAWIC MECHANICAL, LTD., Industrial Park, P.O. Box 588, Nackawic, New Brunswick, CD E0H 1P0. Representative: John C. Lightbody, Esq., 30 Exchange Street, Portland, ME 04101. *Contract carrier:* irregular routes: *Rough and finished lumber* between points on the U.S./CD border at Houlton, ME, on the one hand, and, on the other, Old Town, ME, under continuing contract(s) with Old Town Lumber Company, Bennoch Road, Old Town, ME.

MC 155236 (Sub-1-4TA), filed April 13, 1982. Applicant: POTTLE'S TRANSPORTATION, INC., Odlin Road, Bangor, ME 04401. Representative: Clifton E. Pottle, P.O. Box 164, Carmel, ME 04419. *Contract carrier:* irregular routes: *Malt beverages, wine and liquors, rim, wheels, brakes and parts*, between points in the U.S. (excluding AK and HI) under continuing contract(s) with McLaughlin & Moran, Inc., Providence, RI, and New England Wheel & Rim, Boston, MA. Supporting shipper: McLaughlin & Moran, Inc., P.O. Box 6086, Providence, RI 02940; New England Wheel & Rim, 290 No. Beacon Street, Boston, MA.

MC 142114 (Sub-1-11TA), filed April 12, 1982. Applicant: RETAIL EXPRESS, INC., 9 Stuart Road, Chelmsford, MA 01824. Representative: Frank M. Cushman, 36 South Main Street, Sharon, MA 02067. *Contract carrier:* irregular routes: *Bakery supplies, animal and bird feed, industrial salt products*, between all points in the U.S., under continuing contract(s) with Davis Grande, Inc., No. Bergen, NJ. Supporting shipper: Davis-Grande, Inc., 4401 Dell Avenue, North Bergen, NJ 07047.

MC 161450 (Sub-1-1TA), filed April 12, 1982. Applicant: ROBERTSON FACTORIES, INC., 33 Chandler Avenue, Taunton, MA 02780. Representative: Charles E. Southwell (Same as applicant). *Contract carrier:* irregular routes: *General commodities* between points in the U.S., under continuing contract(s) with Cameo Curtain Company, New Bedford, MA; Mt. Hope Finishing Co., Butner, NC; Polylok Corp., New York, NY. Supporting shipper(s): Cameo Curtain Company, P.O. Box D910, New Bedford, MA 02742; Mt. Hope Finishing Company, Butner, NC 27509; Polylok Corporation, 31 W. 54th Street, New York, NY 10019.

MC 129874 (Sub-1-1TA), filed April 13, 1982. Applicant: TYLER TRANSPORT LIMITED, 379 Queen Street East, Acton, Ontario, CD L7J 2M6. Representative: E. Tyler (Same as applicant). *Contract carrier:* irregular routes: *Lumber and lumber products*, from the U.S./CD International Boundary at Detroit, St. Clair, Niagara and St. Lawrence Rivers to points in DE, IN, KY, MD, MI, NJ, NY, OH, PA, VA, WV, RI, CT, NH, VT, TN, GA, AL, NC, SC, FL, and return of rejected shipments, under continuing contract(s) with Arbre Forest Products, Division of Taiga Wood Products Limited, of Milton, Ontario, CD. Supporting shipper: Arbre Forest Products, Division of Taiga Wood Products Limited, 570 Harrop Drive, Milton, Ontario, CD, L9T 3H2.

The following applications were filed in Region 6. Send protests to: Interstate Commerce Commission, Region 6 Motor Carrier Board, P.O. Box 7413, San Francisco, CA 94120.

MC 79737 (Sub-6-2TA), filed April 9, 1982. Applicant: BERTA BROS. TRANSPORTATION, INC., 3365 E. Hwy. 50, Canon City, CO 81212. Representative: Jack B. Wolfe, 601 E. 18th Ave. #107, Denver, CO 80203. *Contract Carrier*, irregular route, *ores and minerals*, between Grant County, NM on the one hand, and, on the other, Fremont County, CO, for the account of Dorchester Coal Company of Florence, CO, for 270 days. An underlying ETA seeks authority for 120 days. Supporting shipper: Dorchester Coal Company, 1190 County Rd. 92, Florence, CO 81226.

MC 161447 (Sub-6-G-1TA), filed April 12, 1982. Applicant: CARA LINES, 500 Devon Ct., Rio Rancho, NM 87124. Representative: Veronica F. DiZinno (same as applicant). *Contract Carriers* Irregular routes, *lumber, lumber products and roofing materials*, between NM, TX and OK., for 270 days. An ETA

seeks 120 authority. Supporting shipper: Sagebrush Sales Co., P.O. Box 25606, Albuquerque, NM 87125.

MC 160923 (Sub-6-6TA), filed April 8, 1982. Applicant: DIANE P. FRYE, d.b.a. HUSKY TRANSPORTATION, 8527 Emerald, Anchorage, AK 99502. Representative: James A. Frye (same as applicant). *General commodities* (except Class A and B explosives and household goods) restricted to traffic having prior or subsequent movement by water, between Anchorage, AK and all points in AK, for 270 days. An underlying ETA seeks 120 days authority. Supporting shipper: Coastal Alaska Lines, Inc., 1031 W. Ewing St., Seattle, WA 98119.

MC 161428 (Sub-6-1TA), filed April 9, 1982. Applicant: J. B. TRUCKING, 4555 E. Hwy. 40, Vernal, UT 84078. Representative: Gerald D. Baumer (same as applicant). *Materials and supplies used in and around oil fields,* between the states of CA, CO, ID, MT, NE, NV, NM, ND, KS, OK, SD, TX, UT, and WY for 270 days. An underlying ETA seeks 120 days authority. Supporting shippers: Petro-Chem, Inc., 6500 S.E. Hwy. 40, Vernal, UT; Saddleback Drilling, Inc., 1348 South 1500 East, #9, Vernal, UT 84078; and Northern Rig & Reel, 393 S. 400 W., Vernal, UT 84078.

MC 153758 (Sub-6-5TA), filed April 7, 1982. Applicant: LAMPMAN BROKERAGE, INC., d.b.a. MASTRO ENTERPRISES, 4233 West Sierra Madre, Fresno, CA 93711. Representative: James A. Spiegel, Olde Towne Office Park, 6333 Odana Road, Madison, WI 53719. *Contract,* irregular, *food and related products,* between Santa Cruz County, CA, on the one hand, and on the other hand, points in the U.S., for 270 days. Restriction, restricted to shipments performed under continuing contract(s) with Valley Packing Service. Supporting shipper: Valley Packing Service, P.O. Box 108, Watsonville, CA 95077.

MC 143515 (Sub-6-1-TA), filed April 9, 1982. Applicant: P & W CHARTER SERVICE, INC., 1810 S. 11th Street, Union Gap, WA 98903. Representative: R. E. Ammerman (same as applicant). *Contract Carrier,* Irregular routes: *Passenger and Their Baggage* in the same vehicle in Charter Operations from points in WA and OR to points in the U.S. including AK but excluding HI for the account of Senior Citizens Roaming Around the Map, Inc., for 180 days. An underlying ETA seeks 90 days authority. Supporting shipper: Senior Citizens Roaming Around the Map, Inc. P.O. Box 1602, Pendleton, OR 97801.

MC 147727 (Sub-6-4-TA), filed April 8, 1982. Applicant: SCOTT DAVIS TRANSPORT, INC., 611 N. Front St., Yakima, WA 98901. Representative: Jerry R. Woods, 101 SW Main St., Rm. 1600, Portland, OR 97204. *Vinegar, in bulk,* between points in Pierce County, WA on the one hand, and, on the other points in OR, for 270 days. Supporting shipper: Nabisco Brands, Inc., 921 98th Ave., Oakland, CA 94603.

MC 161410 (Sub-6-1-TA), filed April 7, 1982. Applicant: A. B. TIBBITTS, 113 East Main St., Teton City, ID 83451. Representative: J. D. Hancock, P.O. Box 427, Rexburg, ID 83440. (1) *Coal* from and to all points in ID, UT, MT, WY, and WA; and (2) *fertilizer* from and to all points in ID, UT, MT, WY, and WA for 270 days. An underlying ETA seeks 120 days authority. Supporting shipper(s): There are five supporting shippers. Their statements may be examined at the Regional Office listed.

MC 110325 (Sub-6-57-TA), filed April 7, 1982. Applicant: TRANSCON LINES, P.O. Box 92220, Los Angeles, CA 90009. Representative: Jerome Biniasz, P.O. Box 92220, Los Angeles, CA 90009. *Contract carrier,* irregular routes, *General commodities* (except Class A and B explosives, household goods and commodities in bulk) between points in the U.S. (except AK and HI), under continuing contract(s) with Ford Motor Company and subsidiaries for 270 days. Supporting shipper: Ford Motor Company, One Parkland Blvd., Parklane Towers, East, Suite 200, Dearborn, MI 48126.

MC 161254 (Sub-6-1-TA), filed April 8, 1982. Applicant: EMMETT VIRES, 24 150th SW, Lynnwood, WA 98036. Representative: Lawrence V. Smart, Jr., 419 NW 23rd Ave, Portland, OR 97210. *Malt beverages,* from Seattle, WA and Los Angeles, CA to Portland, OR including points in their commercial zones, for 270 days. Supporting shipper: Manchester's Distributors, 620 NE Kelly, Gresham, OR 97030.

Agatha L. Mergenovich,

Secretary.

[FR Doc. 82-10842 Filed 4-20-82; 8:45 am]

BILLING CODE 7035-01-M

[Ex Parte No. 311 (Sub-4)]

Modification of Motor Carrier Fuel Surcharge Program

AGENCY: Interstate Commerce Commission.

ACTION: Change in owner-operator fuel reimbursement figure.

SUMMARY: Due to a decrease in the nationwide average cost of diesel fuel, owner-operator reimbursement has decreased from 13 to 12.5 cents per mile.

EFFECTIVE DATE: This decision will be effective on May 4, 1982.

FOR FURTHER INFORMATION CONTACT:
Lee Alexander, (202) 275-7723
Ted Kalick, (202) 274-6446
Alan Rothenberg, (202) 275-7597
Richard Shullaw, (202) 275-7639

SUPPLEMENTARY INFORMATION: In a decision served March 26, 1982 (47 FR 13421, March 30, 1982) the Commission established owner-operator reimbursement as 13 cents per mile for all carrier-related business miles. This change became effective April 13, 1982. As noted in the October 8, 1981 decision (46 FR 50070, October 9, 1981), the mileage payment will change when the price of fuel in conjunction with the reimbursement formula causes the figure to rise or decline by .5 cents per mile.

As of April 12, 1982, the current price of diesel fuel was 121.7 cents per gallon. The reimbursement figure is 12.3. Ten working days after publication of the notice in the **Federal Register** (on May 4, 1982) carriers shall reimburse owner-operators at a minimum of 12.5 cents per mile.

During this 10-day period or after, if they choose, carriers may adjust their rates to reflect the downward change in owner-operator reimbursement by using the 10-day notice provisions of Special Permission No. 81-2500 (see Part 2 of Appendix B and Appendix C to the October 8 decision). All other normal ratemaking avenues are also available.

Notice shall be given to the general public by mailing a copy of this decision to the Governor of each State having jurisdictional over transportation by depositing a copy in the Office of the Secretary, Interstate Commerce Commission, Washington, D.C., for public inspection and by depositing a copy with the Director, Office of the Federal Register, for publication.

Decided: April 14, 1982.

By the Commission, Chairman Taylor, Vice Chairman Gilliam, Commissioners Gresham, Sterrett, and Andre.

Agatha L. Mergenovich,

Secretary.

April 12, 1982

Appendix A—Diesel Fuel—Prices and Required Mileage Escalation

Date	Price including taxes (cents)	Increased fuel cost		Required[2] amount (cents)
		(cents) total	(over 63.5) per mile[1]	
(1)	(2)	(3)	(4)	(5)
01/01/79	63.5	0	0	0
11/09/81	130.5	67.0	13.4	14.2
11/16/81	130.5	67.0	13.4	14.2
11/23/81	130.5	67.0	13.4	14.2
11/30/81	131.0	67.5	13.5	14.3
12/07/81	131.0	67.5	13.5	14.3
12/14/81	131.0	67.5	13.5	14.3
12/21/81	131.1	67.6	13.5	14.3

APPENDIX A—DIESEL FUEL—PRICES AND REQUIRED MILEAGE ESCALATION—Continued

Date	Price including taxes (cents)	Increased fuel cost		Required[2] amount (cents)
		(cents) total	(over 63.5) per mile[1]	
(1)	(2)	(3)	(4)	(5)
12/28/81	131.1	67.6	13.5	14.3
01/04/82	131.4	67.9	13.6	14.4
01/11/82	131.3	67.8	13.6	14.4
01/18/82	131.2	67.7	13.5	14.3
01/25/82	131.0	67.5	13.5	14.3
02/01/82	130.8	67.3	13.5	14.3
02/08/82	130.3	66.8	13.4	14.2
02/16/82	129.8	66.3	13.3	14.1
02/22/82	129.3	65.8	13.2	14.0
03/01/82	128.8	65.3	13.1	13.9
03/08/82	126.7	63.2	12.6	13.4
03/15/82	125.5	62.0	12.4	13.1
03/22/82	124.4	60.9	12.2	12.9
03/29/82	123.4	59.9	12.0	12.7
04/05/82	121.9	58.4	11.7	12.4
04/12/82	121.7	58.2	11.6	12.3

[1] In cents per mile assuming 5.0 miles per gallon (column 3 divided by 5.0 miles per gallon).
[2] Cost per mile increased by 6 percent for circuity (column 4 multiplied by 1.06).

[FR Doc. 82-10643 Filed 4-20-82; 8:45 am]

BILLING CODE 7035-01-M

[Ex Parte No. 387 (Sub-No. 114)]

Rail Carriers; Missouri Pacific Railroad Co. and Union Pacific Railroad Co.; Exemption for Contract Tariff ICC-UP-C-0022

AGENCY: Interstate Commerce Commission.

ACTION: Notice of provisional exemption.

SUMMARY: Petitioners are granted a provisional exemption under 49 U.S.C. 10505 from the notice requirements of 49 U.S.C. 10713(e). The contract tariff to be filed may become effective on one day's notice. This exemption may be revoked if protests are filed within 15 days of publication in the **Federal Register.**

FOR FURTHER INFORMATION CONTACT: Donald J. Shaw, Jr. or Jane F. Mackall (202) 275-7656.

SUPPLEMENTARY INFORMATION: Missouri Pacific Railroad Company and Union Pacific Railroad Company filed a petition on April 1, 1982, seeking an exemption under 49 U.S.C. 10505 from the statutory notice provisions of 49 U.S.C. 10713(e). Petitioners request that we permit contract tariff ICC-UP-C-0022 to become effective on one day's notice. The tariff was filed to become effective on May 1, 1982. The tariff involves transportation of imported pipe from Freeport, TX, to points in Colorado and Wyoming for use in constructing a pipeline.

Under 49 U.S.C. 10713(e), contracts must be filed on not less than 30 days' notice. There is no provision for waiving this requirement. Cf. former section 10762(d)(1). However, the Commission has granted relief under our section 10505 exemption authority in exceptional situations.

The petition shall be granted. The pipe to be moved under the transportation contract was due to be ready for rail transportation by the end of March. The 30 day notice requirement of section 10713 would delay movement of this pipe. Since the pipe is required immediately at the pipeline construction sites, any delay would create great difficulties and unnecessary expense for the shipper and the pipeline construction company. We find this to be the type of exceptional circumstance which warrants a provisional exemption.

Petitioners' contract ICC-UP-C-0022 may become effective on one day's notice. We will apply the following conditions which have been imposed in similar exemption proceedings:

> If the Commission permits the contract to become effective on one day's notice, this fact neither shall be construed to mean that this is a Commission approved contract for purposes of 49 U.S.C. 10713(g) nor shall it serve to deprive the Commission of jurisdiction to institute a proceeding on its own initiative or on complaint, to review this contract and to disapprove it.

Subject to compliance with these conditions, under 49 U.S.C. 10505(a) we find that the 30 day notice requirement in these instances is not necessary to carry out the transportation policy of 49 U.S.C. 10101a and is not needed to protect shippers from abuse of market power. Further, we will consider revoking this exemption under 49 USC 10505(c) if protests are filed within 15 days of publication in the **Federal Register.**

This action will not significantly affect the quality of the human environment or conservation of energy resources.

(49 U.S.C. 10505)

Dated: April 14, 1982.

By the Commission, Division 2, Commissioners Gresham, Gilliam, and Taylor. Commissioner Gresham did not participate.

Agatha L. Mergenovich,

Secretary.

[FR Doc. 82-10843 Filed 4-20-82; 8:45 am]

BILLING CODE 7035-01-M

[Ex Parte No. 387 (Sub-No. 116)]

Rail Carriers; Richmond, Fredericksburg and Potomac Railroad Co.; Exemption for Contract Tariff ICC-RFP-C-0012

AGENCY: Interstate Commerce Commission.

ACTION: Notice of provisional exemption.

SUMMARY: Petitioner is granted a provisional exemption under 49 U.S.C. 10505 from the notice requirements of 49 U.S.C. 10713(e). The contract tariff to be filed may become effective on one day's notice. This exemption may be revoked if protests are filed within 15 days of publication in the **Federal Register.**

FOR FURTHER INFORMATION CONTACT: Donald J. Shaw, Jr., or Jane F. Mackall, (202) 275-7656.

SUPPLEMENTARY INFORMATION: The Richmond, Fredericksburg and Potomac Railroad Company (RF&P) filed a petition on April 2, 1982, seeking an exemption under 49 U.S.C. 10505 from the statutory notice provisions of 49 U.S.C. 10713(e). Petitioner requests that we permit contract tariff ICC-RFP-C-0012 to become effective on 1 day's notice. The tariff was filed to become effective on April 26, 1982. The tariff provides for the track storage of paper and paper products in equipment carrying RF&P markings.

Under 49 U.S.C. 10713(e), contracts must be filed on not less than 30 days' notice. There is no provision for waiving this requirement. Cf. former section 10762(d)(1). However, the Commission has granted relief under our section 10505 exemption authority in exceptional situations.

The petition shall be granted. Due to the economic downturn, the RF&P has excess equipment, and the paper industry has excess production. The contract allows the shippers to store their shipments in boxcars until needed at destination. This eliminates the need for making a shipment to a storage warehouse and reshipping when the product is needed. It also permits use of idle boxcars. One shipper has developed a production problem and requires immediate storage. Advancement of the effective date would permit immediate use of the boxcars. We find this to be the type of exceptional circumstances which warrants a provisional exemption.

Petitioner's contract tariff ICC-RPF-C-0012 may become effective on one day's notice. We will apply the following conditions which have been

imposed in similar exemption proceedings:

If the Commission permits the contract to become effective on one day's notice, this fact neither shall be construed to mean that this is a Commission approved contract for purposes of 49 U.S.C. 10713(g) nor shall it serve to deprive the Commission of jurisdiction to institute a proceeding on its own initiative or on complaint, to review this contract and to disapprove it.

Subject to compliance with these conditions, under 49 U.S.C. 10505(a) we find that the 30 day notice requirements in these instances is not necessary to carry out the transportation policy of 49 U.S.C. 10101a and is not needed to protect shippers from abuse of market power. Further, we will consider revoking this exemption under 49 U.S.C. 10505(c) if protests are filed within 15 days of publication in the **Federal Register.**

This action will not significantly affect the quality of the human environment or conservation of energy resources.

(49 U.S.C. 10505)

Dated: April 14, 1982.

By the Commission, Division 2, Commissioners Gresham, Gilliam and Taylor.

Agatha L. Mergenovich,

Secretary.

[FR Doc. 82-10844 Filed 4-20-82; 8:45 am]

BILLING CODE 7035-01-M

[Finance Docket No. 29861]

Rail Carriers; Southern Railway Company—Exemption—Abandonment and Trackage Rights Over Seaboard Coast Line Railroad Company in Brunswick, GA

AGENCY: Interstate Commerce Commission.

ACTION: Notice of exemption.

SUMMARY: The Commission exempts from the requirements of prior approval under 49 U.S.C. 10903 the abandonment by Southern Railway Company (Southern) of its mainline track in Brunswick, GA, MP 425.8-H to MP 430.0-H, a distance of approximately 4.2 miles, subject to provisions for the protection of railway labor. Southern will continue to serve shippers over 4.1 miles of parallel Seaboard Coast Line Railroad Company (SCL) track. The trackage rights agreement of Southern and SCL comes within the exemption described at 49 CFR 1111.5(e) (formerly 1111.5(c)(5)) for joint projects involving the relocation of a line of railroad which does not disrupt service to shippers. As a condition to the use of the exemption any Southern employee affected by the trackage rights agreement shall be protected by appropriate labor conditions.

DATES: This exemption is effective on April 21, 1982. Petitions to reopen must be filed within 20 days following publication.

ADDRESSES: Send petitions for reopening to:

(1) Section of Finance, Room 5414, Interstate Commerce Commission, Washington, D.C. 20423; and
(2) Nancy S. Fleischman, Southern Railway Company, P.O. Box 1808, Washington, D.C. 20013. Pleadings should refer to Finance Docket No. 29861.

FOR FURTHER INFORMATION CONTACT: Louis E. Gitomer (202) 275-7245.

SUPPLEMENTARY INFORMATION: The decision served by the Commission contains further information. The decision may be obtained from the Commission's Secretary. For copies of the full decision write to: Interstate Commerce Commission, Room 2227, Washington, DC 20423 or call toll free (800) 424-5403.

Decided: April 14, 1982.

By the Commission, Chairman Taylor, Vice Chairman Gilliam, Commissioners Greshman, Sterrett, and Andre.

Agatha L. Mergenovich,

Secretary.

[FR Doc. 82-10845 Filed 4-20-82; 8:45 am]

BILLING CODE 7035-01-M

[Docket No. AB-167 (Sub-No. 188N)]

Rail Carriers; Conrail Abandonment Between Ellendale and Milton, DE.; Notice of Findings

Notice is hereby given pursuant to Section 308(e) of the Regional Rail Reorganization Act of 1973 that the Commission, review Board Number 1 has issued a certificate authorizing the Consolidated Rail Corporation to abandon its rail line between Ellendale and Milton in the County of Sussex, DE, a total distance of 6.8 miles effective on March 26, 1982.

The net liquidation value of this line is $153,676. If, within 120 days from the date of this publication, Conrail receives a bona fide offer for the sale, for 75 percent of the net liquidation value, of this line it shall sell such line and the Commission shall, unless the parties otherwise agree, establish an equitable division of joint rates for through routes over such lines.

Agatha L. Mergenovich,

Secretary.

[FR Doc. 82-10848 Filed 4-20-82; 8:45 am]

BILLING CODE 7035-01-M

[Docket No. AB-167 (Sub-415N)]

Rail Carriers; Conrail Abandonment Between Crawfordsville and Olin, IN; Notice of Findings

Notice is hereby given pursuant to Section 308(e) of the Regional Rail Reorganization Act of 1973 that the Commission, Review Board Number 3 has issued a certificate authorizing the Consolidated Rail Corporation to abandon its rail line between Crawfordsville and Olin in the County of Washington, IN, a total distance of 28.0 miles effective on March 11, 1982.

The net liquidation value of this line is $1,176,783. If, within 120 days from the date of this publication, Conrail receives a bona fide offer for the sale, for 75 percent of the net liquidation value, of this line it shall sell such line and the Commission shall, unless the parties otherwise agree, establish an equitable division of joint rates for through routes over such lines.

Agatha L. Mergenovich,

Secretary.

[FR Doc. 82-10849 Filed 4-20-82; 8:45 am]

BILLING CODE 7035-01-M

[Docket No. AB-167 (Sub-No. 177N)]

Rail Carriers; Conrail Abandonment Between Frankford Creek and Delaware Expressway, PA; Notice of Findings

Notice is hereby given pursuant to Section 308(e) of the Regional Rail Reorganization Act of 1973 that the Commission, Review Board No. 1 has issued a certificate authorizing the Consolidated Rail Corporation to abandon its rail line between Frankford Creek and Delaware Expressway in the County of Philadelphia, PA, a total distance of 1.9 miles effective on March 11, 1982.

The net liquidation value of this line is $130,560. If, within 120 days from the date of this publication, Conrail receives a bona fide offer for the sale, for 75 percent of the net liquidation value, of this line it shall sell such line and the Commission shall, unless the parties otherwise agree, establish an equitable division of joint rates for through routes over such lines.

Agatha L. Mergenovich,

Secretary.

[FR Doc. 82-10847 Filed 4-20-82; 8:45 am]

BILLING CODE 7035-01-M

[Docket No. AB-167 (Sub-No. 111N)]

Rail Carriers; Conrail Abandonment Between Mill Creek Junction and St. Clair, PA; Notice of Findings

Notice is hereby given pursuant to Section 308(e) of the Regional Rail Reorganization Act of 1973 that the Commission, Review Board Number 2 has issued a certificate authorizing the Consolidated Rail Corporation to abandon its rail line between Mill Creek Junction and St. Clair in the County of Schuylkill, PA, a total distance of 2.6 miles effective on March 15, 1982.

The net liquidation value of this line is $185,395. If, within 120 days from the date of this publication, Conrail receives a bona fide offer for the sale, for 75 percent of the net liquidation value of this line it shall sell such line and the Commission shall, unless the parties otherwise agree, establish an equitable division of joint rates for through routes over such lines.

Agatha L. Mergenovich,

Secretary.

[FR Doc. 82-10846 Filed 4-20-82; 8:45 am]

BILLING CODE 7035-01-M

INTERNATIONAL TRADE COMMISSION

[Investigation No. 337-TA-105]

Certain Coin-Operated Audiovisual Games and Components Thereof (viz. Rally-X and Pac Man); Commission Hearing on the Presiding Officer's Recommendation and on Relief, Bonding, and the Public Interest, and the Schedule for Filing Written Submissions

AGENCY: International Trade Commission.

ACTION: The scheduling of a public hearing and written submissions in investigation No. 337-TA-105, Certain Coin-Operated Audiovisual Games and Components Thereof (viz. Rally-X and Pac Man).

Notice is hereby given that the presiding officer has issued a recommended determination that there is a violation of section 337 of the Tariff Act of 1930, 19 U.S.C. 1337, in the unauthorized importation into the United States and sale of certain coin-operated audiovisual games and components thereof that are the subject of the Commission's investigation. Accordingly, the recommended determination and the record of the hearing have been certified to the Commission for review and a Commission determination. Interested persons may obtain copies of the nonconfidential version of the presiding officer's recommendation (and all other public documents on the record of the investigation) by contacting the Office of the Secretary, U.S. International Trade Commission, 701 E Street NW., Room 161, Washington, D.C. 20436, telephone 202-523-0161.

COMMISSION HEARING: The Commission will hold a public hearing on May 24, 1982, in the Commission's Hearing Room, 701 E Street NW., Washington, D.C. 20436, beginning at 10:00 a.m. The hearing will be divided into two parts. First, the Commission will hear oral arguments on the presiding officer's recommended determination that a violation of section 337 of the Tariff Act of 1930 exists. Second, the Commission will hear presentations concerning appropriate relief, the effect that such relief would have upon the public interest, and the proper amount of the bond during the Presidential review period in the event that the Commission determines that there is a violation of section 337 and that relief should be granted. These matters will be heard on the same day in order to facilitate the completion of this investigation within time limits established under law and to minimize the burden of this hearing upon the parties.

ORAL ARGUMENTS: Any party to the Commission's investigation or any interested Government agency may present an oral argument concerning the presiding officer's recommended determination. That portion of a party's or an agency's total time allocated to oral argument may be used in any way the party or agency making argument sees fit, i.e., a portion of the time may be reserved for rebuttal or devoted to summation. The oral arguments will be held in the following order: complainant, respondents, Government agencies, and the Commission investigative attorney. Any rebuttals will be held in this order: respondents, complainant, Government agencies, and the Commission investigative attorney. Persons making oral argument are reminded that such argument must be based upon the evidentiary record certified to the Commission by the presiding officer.

ORAL PRESENTATIONS ON RELIEF, BONDING, AND THE PUBLIC INTEREST: Following the oral arguments on the presiding officer's recommendation, parties to the investigation, Government agencies, public-interest groups, and interested members of the public may make oral presentations on the issues of relief, bonding, and the public interest. This portion of the hearing is quasi-legislative in nature; presentations need not be confined to the evidentiary record certified to the Commission by the presiding officer, and may include the testimony of witnesses. Oral presentations on relief, bonding, and the public interest will be heard in this order: complainant, respondents, Government agencies, the Commission investigative attorney, public-interest groups, and interested members of the public.

If the Commission finds that a violation of section 337 has occurred, it may issue (1) an order which could result in the exclusion of the subject articles from entry into the United States and/or (2) an order which could result in one or more respondents being required to cease and desist from engaging in unfair methods of competition or unfair acts in the importation and sale of such articles. Accordingly, the Commission is interested in hearing presentations which address the form of relief, if any, which should be ordered.

If the Commission concludes that a violation of section 337 has occurred and contemplates some form of relief, it must consider the effect of that relief upon the public interest. The factors which the Commission will consider include the effect that an exclusion order and/or a cease and desist order would have upon (1) the public health and welfare, (2) competitive conditions in the U.S. economy, (3) the U.S. production of articles which are like or directly competitive with those which are the subject of the investigation, and (4) U.S. consumers.

If the Commission finds that a violation of section 337 has occurred and orders some form of relief, the President has 60 days to approve or disapprove the Commission's action. During this period, the subject articles would be entitled to enter the United States under a bond in an amount determined by the Commission and prescribed by the Secretary of the Treasury. The Commission is therefore interested in hearing presentations concerning the amount of the bond, if any, which should be imposed.

TIME LIMIT FOR ORAL ARGUMENT AND ORAL PRESENTATION: Parties and Government agencies will be limited to a total of 30 minutes (exclusive of time consumed by questions from the Commission or its advisory staff) for making both oral argument on violation and oral presentations on remedy, bonding, and the public interest. Persons making only oral presentations on remedy, bonding, and the public interest will be limited to 10 minutes (exclusive of time consumed by questions from the Commission and its advisory staff). The

Commission may in in its discretion expand the aforementioned time limits upon receipt of a timely request to do so.

WRITTEN SUBMISSIONS: In order to give greater focus to the hearing, the parties to the investigation and interested Government agencies are encouraged to file briefs on the issues of violation (to the extent they have not already briefed that issue in their written exceptions to the presiding officer's recommended determination), remedy, bonding, and the public interest. The complainant and the Commission investigative attorney are also requested to submit a proposed exclusion order and/or proposed cease and desist orders for the Commission's consideration. Persons other than the parties and Government agencies may file written submissions addressing the issues of remedy, bonding, and the public interest. Written submissions on the question of violation must be filed not later than the close of business on April 29, 1982; written submissions on the questions of remedy, bonding, and the public interest must be filed not later than the close of business on May 6, 1982. During the course of the hearing, the parties may be asked to file posthearing briefs.

NOTICE OF APPEARANCE: Written requests to appear at the Commission hearing must be filed with the Office of The Secretary by May 17, 1982.

ADDITIONAL INFORMATION: The original and 14 true copies of all briefs on violation must be filed with the Office of the Secretary not later than April 29, 1982; the original copy and 14 true copies of all briefs on remedy, bonding, and the public interest must be filed with the Office of the Secretary not later than May 6, 1982. Any person desiring to discuss confidential information, or to submit a document (or a portion thereof) to the Commission in confidence, must request in camera treatment unless the information has already been granted such treatment by the presiding officer. All such requests should be directed to the Secretary of the Commission and must include a full statement of the reasons why the Commission should grant such treatment. Documents or arguments containing confidential information approved by the Commission for in camera treatment will be treated accordingly. All nonconfidential written submissions will be available for public inspection at the Secretary's Office.

Notice of this investigation was published in the Federal Register of July 1, 1981, 46 FR 34436.

FOR FURTHER INFORMATION CONTACT: Scott M. Daniels, Esq., Office of the General Counsel, U.S. International Trade Commission, telephone 202-523-0074.

By order of the Commission.

Issued: April 14, 1982.

Kenneth R. Mason,

Secretary.

[FR Doc. 82-10920 Filed 4-20-82; 8:45 am]

BILLING CODE 7020-02-M

[Investigation No. 337-TA-110]

Certain Methods for Extruding Plastic Tubing; Termination of Respondent

AGENCY: International Trade Commission.

ACTION: Termination of investigation as to respondent Logo-Paris, Inc.

SUMMARY: The Commission has terminated the above-captioned investigation as to respondent Logo-Paris, Inc. (Logo-Paris) on the basis of a motion filed by Logo-Paris. The complainant has not opposed the motion. The Commission investigative attorney does not oppose it.

SUPPLEMENTARY INFORMATION: This investigation is being conducted under section 337 and section 337a) of the Tariff Act of 1930 (19 U.S.C. 1337 and 1337a and concerns alleged unfair trade practices in the importation into and sale in the United States of certain plastic bags allegedly made abroad in accordance with the claims of a method patent owned by the complainant in thiss proceeding, Minigrip, Inc. The motion to terminate the investigation as to Logo-Paris included an affidavit by Claude LePage, the president of Logo-Paris. In the affidavit, Mr. LePage states that the plastic bags Logo-Paris sought to import were to be used as packaging for eyeglass frames which it manufactures and sells, but that importation was blocked by the U.S. Customs Service pursuant to the exclusion order issued by the Commission at the conclusion of investigation No. 337-TA-22.

Copies of the Commission's Action and Order and all other nonconfidential documents filed in connection with this investigation are available for inspection during official business hours (8:45 a.m. to 5:15 p.m.) in the Office of the Secretary, U.S. International Trade Commission, 701 E Street NW., Washington, D.C. 20436, telephone 202-523-0161.

FOR FURTHER INFORMATION CONTACT: Jeffrey S. Neeley, Esq., Office of the General Counsel, telephone 202-523-0079.

By order of the Commission.

Issued: April 13, 1982.

Kenneth R. Mason,

Secretary.

[FR Doc. 82-10922 Filed 4-20-82; 8:45 am]

BILLING CODE 7020-02-M

[Investigation No. 337-TA-109]

Certain Multi-Sequential Coded Radio Pagers; Termination of Investigation

AGENCY: International Trade Commission.

ACTION: Termination of investigation based on settlement agreement.

SUMMARY: On January 8, 1982, all parties to *Certain Multi-Sequential Coded Radio Pagers,* Inv. No. 337-TA-109, filed a joint motion to terminate the investigation based on a settlement agreement entered into as of January 18, 1982, by complainant Motorola, Inc., of Schaumburg, Ill. (Motorola) and respondent Nippon Electric Co., Ltd., of Tokyo, Japan (NEC Japan). The Commission published notice containing a summary of the proposed settlement agreement in the Federal Register on February 24, 1982, and requested comments from the public. No comments adverse to termination were received. Having reviewed the record in this investigation, the Commission has voted to grant the parties' joint motion to terminate (Motion 109-2) and is ordering the termination of investigation No. 337-TA-109, *Certain Multi-Sequential Coded Radio Pagers.*

SUPPLEMENTARY INFORMATION: Notice of the institution of this investigation was published in the Federal Register on October 28, 1981 (46 FR 54658). Copies of the Commission's Action and Order and any other public documents in this investigation are available for inspection during official working hours (8:45 a.m. to 5:15 p.m.) in the Office of the Secretary, U.S. International Trade Commission, 701 E Street NW., Washington, D.C. 20436; telephone 202-523-0161. The settlement agreement entered into by the parties contains confidential business information subject to a protective order and is not available for public examination.

FOR FURTHER INFORMATION CONTACT: Jane Albrecht, Esq., Office of the General Counsel, U.S. International Trade Commission, 701 E Street NW., Washington, D.C. 20436; telephone 202-523-1627.

By order of the Commission.

Issued: April 13, 1982.

Kenneth R. Mason,

Secretary.

[FR Doc. 82-10923 Filed 4-20-82; 8:45 am]

BILLING CODE 7020-02-M

[Investigation No. 337-TA-120]

Certain Silica-Coated Lead Chromate Pigments; Investigation

AGENCY: International Trade Commission.

ACTION: Institution of investigation pursuant to 19 U.S.C. 1337.

SUMMARY: Notice is hereby given that a complaint was filed with the U.S. International Trade Commission on March 18, 1982, under section 337 of the Tariff Act of 1930 (19 U.S.C. 1337), on behalf of E.I. du Pont de Nemours and Co., 1007 Market Street, Wilmington, Delaware 19898. An amendment to the complaint was filed on March 31, 1982. The amended complaint (hereinafter the complaint) alleges unfair methods of competition and unfair acts in the importation of certain silicia-coated lead chromate pigments into the United States, or in their sale, by reason of alleged direct infringement of the claims of U.S. Letters Patent 3,639,133. The complaint further alleges that the effect or tendency of the unfair methods of competition and unfair acts is to destroy or substantially injure an industry, efficiently and economically operated, in the United States.

The complainant requests the Commission to institute an investigation and, after a full investigation, to issue an order excluding said pigments from entry into the United States for the life of the patent in issue.

AUTHORITY: The authority for institution of this investigation is contained in section 337 of the Tariff Act of 1930 and in § 210.12 of the Commission's rules of practice and procedure (19 CFR 210.12).

SCOPE OF INVESTIGATION: Having considered the complaint, the U.S. International Trade Commission, on April 14, 1982, ordered that—

(1) Pursuant to subsection (b) of section 337 of the Tariff Act of 1930, an investigation be instituted to determine whether there is a violation of subsection (a) of section 337 in the unlawful importation of certain silica-coated lead chromate pigments into the United States, or in their sale, by reason of alleged direct infringement of the claims of U.S. Letters Patent 3,639,133, the effect or tendency of which is to destroy or substantially injure an industry, efficiently and economically operated, in the United States;

(2) For the purpose of the investigation so instituted, the following are hereby named as parties upon which this notice of investigation shall be served:

(a) The complainant is—E.I. du Pont de Nemours and Co., 1007 Market Street, Wilmington, Delaware 19898.

(b) The respondents are the following companies, alleged to be in violation of section 337, and are the parties upon which the complaint is to be served:

Toho Ganryo Kogyo KK, No. 36-5, 3-Chome Sakashita, Itabashi-ku, Tokyo, Japan 174;

Japan Cotton Co., 630 Houston Natural Gas Building, Houston, Texas 77002;

Synergistic Pigments of Massachusetts, Inc., 765 Quequechan Street, Fall River, Massachusetts 02722;

C. Withington Co., Inc., 16 Pelham Parkway, Pelham Manor, New York 10803.

(c) Oreste Russ Pirfo, Unfair Import Investigations Division, U.S. International Trade Commission, 701 E Street NW., Room 124, Washington, D.C. 20436, shall be the Commission Investigative Attorney, a party to this investigation; and

(3) For the investigation so instituted, Donald K. Duvall, Chief Administrative Law Judge, U.S. International Trade Commission, 701 E Street NW., Washington, D.C. 20436, shall designate the presiding officer.

Responses must be submitted by the named respondents in accordance with § 210.21 of the Commission's rules of practice and procedure (19 CFR 210.21). Pursuant to §§ 201.16(d) and 210.21(a) of the rules, such responses will be considered by the Commission if received not later than 20 days after the date of service of the complaint. Extensions of time for submitting a response will not be granted unless good cause therefor is shown.

Failure of a respondent to file a timely response to each allegation in the complaint and in this notice may be deemed to constitute a waiver of the right to appear and contest the allegations of the complaint and this notice, and to authorize the presiding officer and the Commission, without further notice to the respondent, to find the facts to be as alleged in the complaint and this notice and to enter both a recommended determination and a final determination containing such findings.

The complaint, except for any confidential information contained therein is available for inspection during official business hours (8:45 a.m. to 5:15 p.m.) in the Office of the Secretary, U.S. International Trade Commission, 701 E Street NW., Room 156, Washington, D.C. 20436, telephone 202-523-0471.

FOR FURTHER INFORMATION CONTACT: Oreste Russ Pirfo, Unfair Import Investigations Division, U.S. International Trade Commission, telephone 202-523-4693.

By order of the Commission.

Issued: April 14, 1982.

Kenneth R. Mason,

Secretary.

[FR Doc. 82-10924 Filed 4-20-82; 8:45 am]

BILLING CODE 7020-02-M

[Investigation No. 337-TA-100]

Certain Thermal Conductivity Sensing Gem Testers and Components Thereof; Termination of Investigation Based on a Settlement Agreement

AGENCY: International Trade Commission.

ACTION: Termination of the investigation based on a settlement agreement.

SUPPLEMENTARY INFORMATION: Complainants Ceres Electronics Corp., Adams-Smith, Inc. and MSB Industries, Inc., respondents Brunit Trading AB, Presidium Diamonds Pte Ltd., (Singapore), Presidium, Inc., Gem Instruments Corp., and Gemological Institute of America, and the Commission investigative attorney moved to terminate this investigation on the basis of a settlement agreement.

On February 10, 1982, the Commission published a notice in the **Federal Register** requesting comment from the public and interested Federal agencies on the settlement agreement (47 FR 6118). No comments were received.

On April 8, 1982, the Commission terminated this investigation on the basis of the settlement agreement. The Commission concluded that such termination would not adversely affect the public interest.

Notice of the institution of this investigation was published in the **Federal Register** of May 20, 1981 (46 FR 27586).

Copies of the Commission's Action and Order and all other nonconfidential documents filed in connection with this investigation are available for inspection during official business hours (8:45 a.m. to 5:15 p.m.) in the Office of the Secretary, U.S. International Trade Commission, 701 E Street NW., Washington, D.C. 20436, telephone 202-523-0161.

FOR FURTHER INFORMATION CONTACT: Scott Daniels, Esq., U.S. International Trade Commission, 701 E Street NW.,

Washington, D.C. 20436, telephone 202-523-0074.

By order of the Commission.

Issued: April 16, 1982.

Kenneth R. Mason,

Secretary.

[FR Doc. 82-10928 Filed 4-20-82; 8:45 am]

BILLING CODE 7020-02-M

[Investigation No. 337-TA-3]

Doxycycline; Denial of Motion To Modify Exclusion Order

AGENCY: International Trade Commission.

ACTION: Denial of motion to modify exclusion order.

SUPPLEMENTARY INFORMATION: On April 12, 1979, the Commission issued an order prohibiting the importation into the United States of doxycycline falling within claim 10 of U.S. Letters Patent 3,200,149 for the remaining term of the patent except under license. That order is now in force.

On September 2, 1981, Agvar Chemicalsm Inc. filed a motion with the Commission seeking modification of the doxycycline exclusion order to permit the importation of small quantities of doxycycline to be used for the purpose of obtaining Food and Drug Administration certification under 21 U.S.C. 357, and not for sale to the consuming public.

On April 12, 1982, the Commission denied the motion of Agvar Chemicals, Inc. to modify the doxycycline exclusion order.

FOR FURTHER INFORMATION CONTACT: Lairold M. Street, Esq., Office of the General Counsel, U.S. International Trade Commission, 701 E Street NW., Washington, D.C. 20436; telephone 202-523-0124.

By order of the Commission.

Issued: April 16, 1982.

Kenneth R. Mason,

Secretary.

[FR Doc. 82-10931 Filed 4-20-82; 8:45 am]

BILLING CODE 7020-02-M

[332-73]

Release for Public Comment of Provisionally Adopted Chapters of the Harmonized Commodity Description and Coding System

AGENCY: International Trade Commission.

ACTION: Release for public comment, pursuant to Commission investigation No. 332-73, under the authority of section 332(g) of the Tariff Act of 1930, as amended, of drafts of Explanatory Notes to the following chapters of the Harmonized Commodity Description and Coding System (Harmonized System) as provisionally adopted by the Harmonized System Committee and the Nomenclature Committee of the Customs Cooperation Council.

Volume 3

Chapter 15: Animal and vegetable fats and oils and their clevage products; animal and vegetable waxes
Chapter 20: Preparations of vegetables, fruit, nuts, or other parts of plants
Chapter 21: Miscellaneous edible preparations
Chapter 22: Beverages, spirits and vinegar

Volume 4

Chapter 25: Salt; sulphur; earth and stone; plastering materials; lime and cement
Chapter 27: Mineral fuels, mineral oils and products of their distillation; bituminous substances; mineral waxes
Chapter 30: Pharmaceutical products
Chapter 34: Soap, organic surface-active agents, washing preparations, lubricating preparations, artificial waxes, prepared waxes, polishing and scouring preparations, candles and similar articles, modelling pastes and "dental waxes"
Chapter 45: Cork and articles of cork
Chapter 47: Pulp of wood or of other fibrous cellulosic, material; waste and scrap of paper or paperboard
Chapter 49: Printed books, newspapers, pictures and other products of the printing industry; manuscripts, typescripts and plans

Volume 5

Chapter 50: Silk
Chapter 51: Wool, fine or coarse animal hair; horsehair yarn and woven fabric
Chapter 52: Cotton
Chapter 53: Other vegetable textile fibers; paper yarn and woven fabrics of paper yarn
Chapter 54: Man-made filaments

WRITTEN SUBMISSIONS: Parties wishing to submit written comments should do so by filing them with the Secretary of the Commission at his office in Washington, D.C. no later than the close of business on April 30, 1982.

COPIES OF DOCUMENTS: Copies of Explanatory Notes which are the subject of this notice are available for public inspection at the offices of the Commission, 701 E Street, NW., Washington, D.C. 20436. The Secretary will also send copies to interested parties upon request.

FOR FURTHER INFORMATION CONTACT: Eugene A. Rosengarden, Director, or Holm Kappler, Deputy Director, Office of Tariff Affairs, U.S. International Trade Commission, 701 E Street, NW., Washington, D.C. 20436; Telephone: (202) 523-0370 or 0362.

SUPPLEMENTARY INFORMATION: In its public notices of February 8, 1980 (45 FR 9828 of February 13, 1980), March 21, 1980 (45 FR 19696 of March 26, 1980), August 15, 1980 (45 FR 55449 of August 20, 1980), June 24, 1981 (46 FR 34439 of July 11, 1981), the Commission identified the 97 chapters of the Harmonized System for which texts had been provisionally adopted by the Harmonized System and Nomenclature Committees of the Customs Cooperation Council. Views and comments of interested parties with respect to the nomenclature structure formulated in the 97 chapters were sought by those notices.

This notice is being issued pursuant to Commission investigation No. 332-73, instituted on January 31, 1975 (40 FR 6329), under section 332(g) of the Tariff Act of 1930. The public notice of July 17, 1981 (46 FR 37824) set forth the basis of the Commission's investigation in order to participate in technical work on, and described the structure and development of, the Harmonized System.

The draft chapters identified by the Commission contain the headings of the nomenclature as provisionally adopted. Legal notes, which have the same binding force as the headings and as the rules of interpretation of the nomenclature, are provided to define the scope of a heading or the meaning of terms, to list articles covered by a heading or group of headings, and to list excluded articles.

Explanatory Notes, which do not form a part of the nomenclature, contain the official interpretation of the nomenclature ultimately to be adopted by the Customs Cooperation Council. The notes are arranged in the scope of each heading, including the products included and excluded, technical product descriptions, a guide for product identification, and the appearance, properties, uses, and methods of production of the products concerned. The public notice of February 16, 1982 (47 FR 8108) identified chapters for which Explanatory Notes were available and requested public comment. The notice sought views on Explanatory Notes for Chapters 1 through 14, 16 through 19, 23, 24, 26, 31, 35 through 37, 41 through 43, and 46.

Drafts of the above Explanatory Notes are being finally reviewed by both the Harmonized System Committee and the Nomenclature Committee. As texts of further Explanatory Notes are adopted, the Commission will issue future notices requesting public comment.

By order of the Commission.

Issued: April 14, 1982.

Kenneth R. Mason,

Secretary.

[FR Doc. 82-10919 Filed 4-20-82; 8:45 am]

BILLING CODE 7020-02-M

[Investigation No. 731-TA-91 (Preliminary)]

Sodium Nitrate From Chile; Preliminary Antidumping Investigation

AGENCY: International Trade Commission.

ACTION: Institution of preliminary antidumping investigation and scheduling of a conference to be held in connection therewith.

SUMMARY: The U.S. International Trade Commission hereby gives notice of the institution of investigation No. 731-TA-91 (Preliminary) under section 733(a) of the Tariff Act (19 U.S.C. 1673b(a)) to determine whether there is a reasonable indication that an industry in the United States is materially injured, or is threatened with material injury, or the establishment of an industry in the United States is materially retarded, by reason of imports from Chile of sodium nitrate, provided for in item 480.25 of the Tariff Schedules of the United States, which are allegedly being sold in the United States at less than fair value (LTFV).

EFFECTIVE DATE: April 12, 1982.

FOR FURTHER INFORMATION CONTACT: Mr. Woodley Timberlake, Office of Investigations, U.S. International Trade Commission; telephone 202-523-4618.

SUPPLEMENTARY INFORMATION:

Background

This investigation is being instituted in response to a petition filed April 12, 1982, on behalf of Olin Corporation. The Commission must make its determination in the investigation within 45 days after the date of the filing of the petition, or in this case by May 27, 1982. The investigation will be subject to the provisions of part 207, Subpart B, of the Commission's Rules of Practice and Procedure (19 CFR Part 207).

Written Submissions

Any person may submit to the Commission on or before May 10, 1982, a written statement of information pertinent to the subject matter of this investigation. A signed original and fourteen (14) copies of such statements must be submitted.

Any business information which a submitter desires the Commission to treat as confidential shall be submitted separately, and each sheet must be clearly marked at the top "Confidential Business Data." Confidential submissions must conform with the requirements of § 201.6 of the Commission's rules of practice and procedure (19 CFR 201.6). All written submissions, except for confidential business data will be available for public inspection.

Conference

The Director of Operations of the Commission has scheduled a conference in connection with this investigation for 10:00 a.m., on May 4, 1982, at the U.S. International Trade Commission Building, 701 E Street, NW., Washington, D.C. Parties wishing to participate in the conference should contact the investigator for this investigation, Mr. Woodley Timberlake, telephone 202-523-4618, not later than April 27, 1982, to arrange for their appearance.

Inspection of the Petition

The petition filed in this case is available for public inspection at the Office of the Secretary, U.S. International Trade Commission.

This notice is published pursuant to § 207.12 of the Commission's rules of practice and procedure (19 CFR 207.12).

By order of the Commission.

Issued: April 16, 1982.

Kenneth R. Mason,

Secretary.

[FR Doc. 82-10925 Filed 4-20-82; 8:45 am]

BILLING CODE 7020-02-M

NATIONAL FOUNDATION ON THE ARTS AND THE HUMANITIES

Humanities Panel Meeting

AGENCY: National Endowment for the Humanities, NFAH.

ACTION: Notice of meeting.

SUMMARY: Pursuant to the provision of the Federal Advisory Committee Act (Public Law 92-463, as amended), notice is hereby given that the following meeting of the Humanities Panel will be held at 806 15th Street, N.W., Washington, D.C. 20506.

Date: May 24-25, 1982

Time: 8:30 a.m. to 5:30 p.m.

Room: 807

Program: This meeting will review applications submitted by state humanities committees, Division of State Programs, for activity beginning after November 1, 1982.

The proposed meeting is for the purpose of Panel review, discussion, evaluation and recommendation on applications for financial assistance under the National Foundation on the Arts and the Humanities Act of 1965, as amended, including discussion of information given in confidence to the agency by grant applicants. Because the proposed meeting will consider information that is likely to disclose:

(1) Trade secrets and commercial or financial information obtained from a person and privileged or confidential;

(2) Information of a personal nature the disclosure of which would constitute a clearly unwarranted invasion of personal privacy; and

(3) Information the disclosure of which would significantly frustrate implementation of proposed agency action;

pursuant to authority granted me by the Chairman's Delegation of Authority to Close Advisory Committee Meetings, dated January 15, 1978, I have determined that this meeting will be closed to the public pursuant to subsections (c)(4), (6) and (9)(B) of section 552b of Title 5, United States Code.

Further information about this meeting can be obtained from Mr. Stephen J. McCleary, Advisory Committee Management Officer, National Endowment for the Humanities, D.C. 20506 or call (202) 724-0367.

Stephen J. McCleary,

Advisory Committee Management Officer.

[FR Doc. 82-10888 Filed 4-20-82; 8:45 am]

BILLING CODE 7536-01-M

Music Advisory Panel (Composers Prescreening); Meeting

Pursuant to section 10 (a)(2) of the Federal Advisory Committee Act (Pub. L. 92-463), as amended, notice is hereby given that a meeting of the Music Advisory Panel (Composers Prescreening) to the National Council on the Arts will be held April 29-30, 1982, from 9:00 a.m.-5:30 p.m., in the 12 the floor screening of the Columbia Plaza Office Complex, 2401 E Street, N.W., Washington, D.C., 20506.

This meeting is for the purpose of Panel review, discussion, evaluation, and recommendation on applications for financial assistance under the National foundation on the Arts and the Humanities Act of 1965, as amended, including discussion of information given in confidence to the agency by grant applicants. In accordance with the determination of the Chairman published in the Federal Register of February 13, 1980, these sessions will be closed to the public pursuant to subsections (c) (4), (6) and 9(b) of section 552b of Title 5, United States Code.

Further information with reference to this meeting can be obtained from Mr. John H. Clark, Advisory Committee Management Officer, National Endowment for the Arts, Washington, D.C. 20506, or call (202) 634-6070.

John H. Clark,

Director, Office of Council and Panel Operations, National Endowment for the Arts.

April 14, 1982.

[FR Doc. 82-10822 Filed 4-20-82; 8:45 am]

BILLING CODE 7537-01-M

NATIONAL SCIENCE FOUNDATION

Advisory Council; Meeting

In accordance with the Federal Advisory Committee Act, Pub. L. 92-463, the National Science Foundation announces the following meeting:

Name: NSF Advisory Council
Place: Room 540, National Science Foundation, 1800 G Street, N.W., Washington, D.C. 20550
Date: Thursday & Friday, May 6 &7 1982
Time: 9:00 a.m. till 5:00 p.m. both days
Type of Meeting: Open
Contact person: Ms. Jeanne Hudson, Executive Secretary, NSF Advisory Council, National Science Foundation, Rm. 518, 1800 G Street, N.W., Washington, D.C. 20550. Telephone: 202/357-9419
Purpose of advisory council: The purpose of the NSF Advisory Council is to provide advice and counsel to the NSF Director and principal members of his staff on Foundation-wide issues which require the expertise of the many and varied disciplines and program interests represented in the Foundation.
Summary minutes: May be obtained from the contact person at above stated address.
Agenda: To review progress by the three task groups of NSF Advisory Council and to meet with the Director and Deputy Director and NSF Staff.

M. Rebecca Winkler,

Committee Management Coordinator.

April 15, 1982.

[FR Doc. 82-10823 Filed 4-20-82; 8:45 am]

BILLING CODE 7555-01-M

NUCLEAR REGULATORY COMMISSION

[Docket Nos. STN-454 and STN 50-455]

Availability of Final Environment Statement for the Byron Station, Units 1 and 2, Commonwealth Edison Co.

Notice is hereby given that the Final Environmental Statement (NUREG-0848) has been prepared by the Commission's Office of Nuclear Reactor Regulation related to the proposed operation of the Byron Station, Units 1 and 2 by Commonwealth Edison Company. The station is located in Rockvale Township, Ogle County, Illinois.

The Final Environmental Statement NUREG-0848) is available for inspection by the public in the Commission's Public Document Room at 1717 H Street NW., Washington, D.C. 20555 and in the Rockford Public Library, 215 N. Wyman Street, Rockford, Illinois. The Final Environmental Statement is also being made available at the State Clearinghouse, Bureau of the Budget, Lincoln Tower Plaza, 5245 Second Street, Springfield, Illinois 62706.

The notice of availability of the Draft Environmental Statement (DES) for the Byron Station, Units 1 and 2, and request for comments was published in the **Federal Register** on December 4, 1981 (46 FR 59350). The comments received from Federal, State and local agencies, and interested members of the public have been included as appendices to the Final Environmental Statement.

Copies of the Final Environmental Statement (NUREG-0848) may be purchased at current rates from the National Technical Information Service, Department of Commerce, 5285 Port Royal Road, Springfield, Virginia 22161, and from the Sales Office, U.S. Nuclear Regulatory Commission, Washington, D.C. 20555.

For the Nuclear Regulatory Commission.

William F. Kane,

Acting Branch Chief, Licensing Branch No. 1, Division of Licensing.

[FR Doc. 82-10872 Filed 4-20-82; 8:45 am]

BILLING CODE 7590-01-M

[Docket No. 50-255]

Consumers Power Co.; Issuance of Amendment to Provisional Operating License

The U.S. Nuclear Regulatory Commission (the Commission) has issued Amendment No. 70 to Provisional Operating License No. DPR-20, issued to Consumers Power Company (the licensee), which revised the Technical Specifications for operation of the Palisades Plant (facility) located in Van Buren County, MI. This amendment is effective as of its date of issuance.

The amendment approves the change which clarifies the implementation of the Appendix A Technical Specifications with regard to shutdown margin requirements with less than four primary coolant pumps in operation.

The application for amendment complies with the standards and requirements of the Atomic Energy Act of 1954, as amended (the Act), and the Commission's rules and regulations. The Commission has made appropriate findings as required by the Act and the Commission's rules and regulations in 10 CFR Chapter I, which are set forth in the license amendment. Prior public notice of this amendment was not required since the amendment does not involve a significant hazards consideration.

The Commission has determined that the issuance of this amendment will not result in any significant environmental impact and that pursuant to 10 CFR § 51.5(d)(4) an environmental impact statement or negative declaration and environmental impact appraisal need not be prepared in connection with issuance of this amendment.

For further details with respect to this action, see (1) the application for amendment dated May 5, 1980, (2) Amendment No. 70 to License No. DPR-20, and (3) the Commission's related Safety Evaluation. These items are available for public inspection at the Commission's Public Document Room, 1717 H Street, N.W., Washington, D.C. and at the Kalamazoo Public Library, 315 South Rose Street, Kalamazoo, Michigan 49006.

A single copy of items (2) and (3) may be obtained by request addressed to the U.S. Nuclear Regulatory Commission, Washington, D.C. 20555, Attention: Director, Division of Licensing.

Dated at Bethesda, Maryland, this 14th day of April 1982.

For the Nuclear Regulatory Commission.

Dennis M. Crutchfield,

Chief, Operating Reactors Branch No. 5, Division of Licensing.

[FR Doc. 82-10873 Filed 4-20-82; 8:45 am]

BILLING CODE 7590-01-M

PENSION BENEFIT GUARANTY CORPORATION

Pendency of Request for Exemption From Bond Escrow and Sale-Contract Requirements Relating to Sale of Assets by an Employer That Contributes to a Multiemployer Plan; RGZ, Inc., et al.

AGENCY: Pension Benefit Guaranty Corporation.

ACTION: Notice of consideration of class exemption.

SUMMARY: This notice advises interested persons that, on the basis of a joint request from RGZ, Inc./Gulf Elevator & Transfer Company, Inc. and Cooper Stevedoring Co., Inc. for an exemption from the bond/excrow and sale-contract requirements of section 4204(a)(1)(B) and (C) of the Employee Retirement Income Security Act of 1974, as

amended, the Pension Benefit Guaranty Corporation is considering granting a class exemption for certain transactions occurring prior to and soon after enactment of the Multiemployer Pension Plan Amendments Act of 1980. Section 4204(a)(1) provides that the sale of assets by an employer that contributes to a multiemployer pension plan will not constitute a complete or partial withdrawal from the plan if certain conditions are met. One of these conditions is that the purchaser post a bond or deposit money in escrow for five plan years beginning after the sale. Another condition is that the sales agreement provide that the seller will be secondarily liable for its withdrawal liability if the purchaser withdraws within the first five plan years after the sale and fails to pay withdrawal liability. The PBGC is authorized to grant individual and class exemptions from these requirements. Prior to granting an exemption, the PBGC is required to give interested persons an opportunity to comment on the exemption request. The effect of this notice is to solicit the views of interested persons on this exemption request and the PBGC's proposal to grant a class exemption.

DATES: Comments must be submited on or before June 7, 1982.

ADDRESSES: All written comments (at least three copies) should be addressed to: Assistant Executive Director for Policy and Planning (140), Pension Benefit Guaranty Corporation, 2020 K Street, NW., Washington, D.C. 20006. The request for exemption and the comments received will be available for public inspection at the PBGC Public Affairs Office, Suite 7100, at the above address, between the hours of 9:00 a.m. and 4:00 p.m.

FOR FURTHER INFORMATION CONTACT: James M. Graham, Office of the Executive Director, Policy and Planning (140), Pension Benefit Guaranty Corporation, 2020 K Street, NW., Washington, D.C. 20006; (202) 254-4862. [This is not a toll-free number.]

SUPPLEMENTARY INFORMATION:

Background

The Multiemployer Pension Plan Amendments Act of 1980, Pub. L. 96-364, 94 Stat. 1208 (the "Multiemployer Act") became law on September 26, 1980 and amended the Employee Retirement Income Security Act of 1974 ("ERISA"), 29 U.S.C. 1001 *et seq.* As a result of the Multiemployer Act, an employer that withdraws, or partially withdraws, from a multiemployer pension plan covered under Title IV of ERISA may be liable to the plan for a portion of the plan's unfunded vested benefits.

Section 4204 of ERISA, 29 U.S.C. 1384, provides that a bona fide arm's-length sale of assets of a contributing employer to an unrelated party will not be considered a withdrawal if three conditions are met. These conditions, enumerated in section 4204(a)(1)(A)-(C), are that—

(A) The purchaser has an obligation to contribute to the plan for substantially the same number of contribution base units for which the seller was obligated to contribute;

(B) The purchaser obtains a bond or places an amount in escrow, for a period of five plan years after the sale, in an amount equal to the greater of the seller's average required annual contribution to the plan for the three plan years preceding the year in which the sale occurred or the seller's required annual contribution for the plan year preceding the year in which the sale occurred; and

(C) The contract of sale provides that if the purchaser withdraws from the plan within the first five plan years beginning after the sale and fails to pay any of its liability to the plan, the seller shall be secondarily liable for the liability it (the seller) would have had but for section 4202.

The bond or escrow described above would be paid to the plan if the purchaser withdraws from the plan or fails to make any required contributions to the plan within the first five plan years beginning after the sale.

When section 4204 applies to a transaction, both purchaser and seller assume certain responsibilities. The purchaser assumes the contribution record of the seller for the plan year in which the sale occurred and the preceding four plan years (section 4204(b)(1)). The seller becomes secondarily liable under section 4204(a)(2) if, within five plan years after the sale, the purchaser withdraws and fails to make a withdrawal liability payment when due. Both of these obligations occur as a matter of law if the sale is covered by section 4204.

All of the withdrawal liability provisions of the Multiemployer Act—sections 4201 through 4225—are effective as of April 29, 1980. Since Congress made section 4204 effective as of April 29, 1980, it is apparent that Congress intended that provision to be available with respect to transactions occurring after that date that would otherwise constitute withdrawals.

Section 4204(c) of ERISA authorizes the Pension Benefit Guaranty Corporation ("PBGC") to grant individual or class variances or exemptions from the purchaser's bond/escrow requirement of section 4204(a)(1)(B) and the sale-contract requirement of section 4204(a)(1)(C) when warranted. The legislative history of section 4204 indicates a Congressional intent that the sales rules be administered in a manner that assures protection of the plan with the least practicable intrusion into normal business transactions. The granting of an exemption or variance from the requirements of section 4204(a)(1)(B) or (C) does not constitute a finding by PBGC that a particular transaction satisfies the other requirements of section 4204(a)(1).

The Request

The PBGC has received a joint request from the seller, RGZ, Inc./Gulf Elevator & Transfer Company, Inc. ("RGZ/GETCO"), and the purchaser, Cooper Stevedoring Co., Inc. ("Cooper"), (collectively referred to as the "Parties") for an exemption from the requirements of section 4204(a)(1)(B) and (C) of ERISA. In the request, the Parties represent, among other things, that:

1. On October 5, 1980, Cooper purchased certain assets of RGZ/GETCO.

2. Cooper has assumed RGZ/GETCO's responsibilities under a collective bargaining agreement with the International Longshoremen's Association Local No. 3033, which obligated RGZ/GETCO to contribute to the New Orleans Steamship Association, International Longshoremen's Association, AFL-CIO, Pension Plan (the "Plan"). According to the Plan, RGZ/GETCO's potential withdrawal liability to the Plan had been calculated to be $196,821.

3. The amount of the bond or escrow required under section 4204(a)(1)(B) is $221,285 (the annual contribution required to be made by RGZ/GETCO for the 1980 plan year, the plan year preceding the sale).

4. On November 23, 1981, RGZ/GETCO and Cooper entered into an agreement whereby RGZ/GETCO agreed, if section 4204 was applicable to the sale, that it would be secondarily liable under section 4204(a)(1)(C).

5. An exemption should be granted from the requirements of section 4204(a)(1)(B) and (C), because the sale was consummated only nine days after the enactment of the Multiemployer Act. To not do so, in view of the fact that the

parties could not realistically have been aware of these requirements at the time the sale was consummated, "would be unjust, harsh and detrimental to RGZ/GETCO and Cooper."

6. A complete copy of this request has been sent by the Parties to the Plan and the collective bargaining representative of the seller's former employees.

No financial information on the purchaser was submitted as part of this request.

Issue Under Consideration

This request asserts that relief is warranted under section 4204(c) because the sale was consummated only nine days after the enactment of the Multiemployer Act, when many persons were unaware of the Act's provisions, and the parties want their sale to be covered by section 4204 and have indicated their agreement to assume the responsibilities they would incur if section 4204 applies. PBGC believes that if it concludes that these facts do warrant the granting of an exemption under section 4204(c), it follows that this relief would be warranted in all other similar sale of assets cases. Accordingly, on the basis of the instant request, PBGC is considering granting a class exemption from the requirements of section 4204(a)(1)(B) and (C) for sales that were consummated before or soon after enactment of the Multiemployer Act, where the parties jointly indicate an intention for their sale to be covered by section 4204. Further, PBGC has tentatively decided that January 1, 1981 would be an appropriate cut-off for the exemption. Thus, if granted, the exemption would apply to all sales consummated prior to January 1, 1981, if the parties jointly notify the affected plan of their intention that their transaction be covered by section 4204.

Comment

All interested persons are invited to submit written comments on the pending class exemption to the above address, on or before June 7, 1982. All comments will be made a part of the record. Comments received, as well as the application for exemption, will be available for public inspection at the address set forth above.

Issued at Washington, D.C., on this 16th day of April, 1982.

Robert E. Nagle,

Executive Director, Pension Benefit Guaranty Corporation.

[FR Doc. 82-10824 Filed 4-20-82; 8:45 am]

BILLING CODE 7708-01-M

PRESIDENT'S COMMISSION ON WHITE HOUSE FELLOWSHIPS

Meeting

Pursuant to section 10(a)(2) of Pub. L. 92-463, the Federal Advisory Committee Act, notice is hereby given that the Annual Selection Meeting of the President's Commission on White House Fellowships will be held at The Homestead, Hot Springs, Virginia, on June 3-6, 1982.

The Annual Selection Meeting is part of the screening process of the White House Fellowships program. During this three-day meeting the thirty-three national finalists will be interviewed by the members of the Presidential Commission. At the conclusion of this meeting the Commission recommends to the President fourteen to twenty persons to serve as White House Fellows.

It has been determined by the Director of the Office of Personnel Management that, because of the very nature of the screening process where personnel records and confidential character references must be used which, if revealed to the public, would constitute a clear invasion of an applicant's privacy, the content of this meeting falls within the provisions of Section 552b(c)(6) of title 5 of the United States Code and that this meeting will be closed to the public.

James C. Roberts,

Director.

Determination to Close the Final Selection Meeting of the President's Commission on White House Fellowships

The final selection meeting of the President's Commission on White House Fellowships is part of the screening process leading to the selection of individuals for White House Fellowships. In this meeting, national finalists of the program are interviewed by the Commission members. The Commission also reviews confidential files and character references which we believe are exempt from disclosure under the provisions of the Freedom of Information and Privacy Acts. At the conclusion of the interviews, the Commission recommends to the President those individuals who should serve as White House Fellows.

This meeting is covered by the provisions of the Federal Advisory Committee act; Pub. L. 92-463. That act provides that meetings may be closed to the public only as provided for by subsection (c) of section 552b of title 5, United States Code. That section contains the ten exemptions to the open meeting requirements of the "Government in the Sunshine Act." Exemption (6) permits closing of meetings where they would "disclose information of a personal nature when disclosure would constitute a clearly unwarranted invasion of personal privacy:"

It is hereby determined that the final selection meeting of the President's Commission on White House Fellowships is concerned with personal information as described in exemption (6) above. Accordingly, the final selection meeting of the Commission to be held on June 3-6, 1982, will be closed to the public.

Donald J. Devine,

Director, Office of Personnel Management.

[FR Doc. 82-10833 Filed 4-20-82; 8:45 am]

BILLING CODE 6325-01-M

SECURITIES AND EXCHANGE COMMISSION

[Rel. No. 12371; 812-4796]

Allied Capital Corp., et al.; Filing of Application

April 14, 1982.

Notice is hereby given that Allied Capital Corporation ("Allied Capital"), Allied Investment Corporation ("Allied Investment"), Allied Lending Corporation ("Allied Lending"), all registered under the Investment Company Act of 1940 ("Act") as closed-end, non-diversified management investment companies; and Allied Development Corporation ("Allied Development"), a District of Columbia corporation, 1625 I Street, N.W., Washington, D.C. 20006, which intends to register under the Act as a closed-end, non-diversified management investment company (collectively referred to herein as the "Funds", "Applicants" or the "Allied Group"), filed an application on January 5, 1982, and amendments thereto on February 17, and March 24, 1982, requesting an order of the Commission pursuant to section 6(c) of the Act exempting Applicants from the provisions of sections 8(b), 12(e), 17(a), 17(d), 18(a), 18(c), 30(a) and 30(d) of the Act and Rules 8b-16, 17d-1, 30a-1 and 30d-1 thereunder to the extent requested; and requesting an order of the Commission amending in the manner described below an earlier order of the Commission dated November 24, 1976 (Investment Company Act Release No. 9540). All interested persons are referred to the application on file with the Commission for a statement of the representations contained therein, which are summarized below.

Allied Capital was organized in 1958 and commenced business as a "venture capital" company. Until 1977 Allied Capital was licensed by the Small Business Administration ("SBA") as a small business investment company ("SBIC") under the Small Business Investment Act of 1958. According to the application, Allied Capital became a holding company having, in addition to an existing wholly-owned subsidiary, Allied Advisory, Inc. ("Allied Advisory"), two newly formed wholly-owned subsidiaries, Allied Investment and Allied Lending, pursuant to a reorganization which was effective April 1, 1977. Pursuant to this reorganization, Allied Capital transferred its SBIC license to Allied Investment.

Allied Investment, a District of Columbia corporation, is licensed by the SBA as a SBIC and is principally engaged in furnishing financial assistance to small business concerns through loans, guarantees and equity investments. Allied Lending, a District of Columbia corporation, is recognized by the SBA as a participating lender under the small business lending company program and is primarily engaged in making loans to qualified small businesses, which loans are guaranteed as to principal and interest up to 90% by the SBA. Allied Advisory, a District of Columbia corporation, is enagaged in the business of providing loan packaging services, financial analysis and limited corporate consulting.

Allied Development is a District of Columbia corporation newly organized by Allied Capital. According to the application, Allied Development, which has not commenced business, proposes to register as a non-diversifed, closed-end, investment company and to participate in government guaranteed lending programs other than the SBA program such as the Business and Industrial Loan Program administered by the Farmers Home Administration ("FMHA") of the Department of Agriculture or the guaranteed loan program of the Economic Development Administration ("EDA") of the Department of Commerce. Applicants state that the FMHA and EDA may guarantee up to 90% of loans made under their programs, but that the continuation of such programs is dependent on their obtaining adequate fundings. Applicants further state that Allied Development has been approved as an eligible lender by both the FMHA and EDA. Allied Capital proposes to fund Allied Development by acquiring its common stock for an aggregate purchase price of $100,000 and a direct loan of $100,000.

Applicants state that in connection with the 1977 reorganization of Allied Capital, Allied Capital received an order of the Commission pursuant to section 6(c) of the Act granting exemptions from sections 12(e), 17(a) and 17(d) of the Act and Rule 17d-1 thereunder to the extent necessary to permit it to acquire all of the issued and outstanding common stock of Allied Investment and Allied Lending and to permit Allied Capital and its wholly-owned subsidiaries to engage in certain affiliated and joint transactions subject to conditions, which order was dated November 24, 1976 (Investment Company Act Release No. 9540) ("1976 Order"). The present application seeks comparable exemptive relief from the Act with respect to Allied Development. In addition, the present apaplication seeks exemptive relief from the provisions of section 12(e) of the Act to permit Allied Capital to make further acquisitions of securities (including debt securities) of Allied Investment, Allied Lending and Allied Development. The present application also seeks exemptive relief from the provisions of sections 18(a) and 18(c) of the Act to permit Allied Capital and each of its wholly-owned investment company subsidiaries to borrow from banks, insurance companies and other financial institutions (including in the case of such subsidiaries from Allied Capital) on a secured or unsecured basis and to permit Allied Capital to guarantee such borrowings. Finally, the present application seeks exemptive relief from the provisons of sections 8(b), 30(a) and 30(d) of the Act and Rules 8b-16, 30a-1 and 30d-1 thereunder to permit Allied Capital to file with the Commission on behalf of itself and its three wholly-owned investment company subsidiaries annual reports, and amendments to its registration statement and to transmit to its shareholders reports containing financial statements of Allied Capital and its investment company subsidiaries on a consolidated basis only.

Section 12(d)(1)(A) of the Act, as here pertinent, prohibits a registered investment company from acquiring more than three percent of the total outstanding voting stock of any other investment company, and section 12(d)(1)(C) of the Act prohibits an investment company from acquiring more than 10 percent of the total outstanding voting stock of a registered closed-end investment company. Section 12(e) of the Act excludes from the provisions of section 12(d)(1) any purchase or acquisition by a registered investment company of any security issued by any one corporation engaged or proposing to engage in the business of underwriting, furnishing capital to industry, financing promotional enterprises, purchasing securities of issuers for which no ready market is in existence, and reorganizing companies or similar activities provided, among other things, that the aggregate cost of the securities of such other corporation purchased by such registered investment company does not exceed five percent of the value of the total assets of such registered company at the time of any purchase or acquisition of such securities and that the securities issued by such corporation (other than short-term paper and securities representing bank loans) consist solely of one class of common stock. Because the proposed investment by Allied Capital in Allied Development will consist of the issuance of common stock and the creation of a debt security, the transaction will involve the issuance by Allied Development of securities other than one class of common stock. In addition, Applicants seek exemptive relief from the provisions of section 12(e) to permit future acquisitions by Allied Capital of the common stock of Allied Investment, Allied Lending and Allied Development which purchases might cause Allied Capital's investment therein, taken at cost, to exceed five percent of the value of its total assets at the time of any such purchase. Furthermore, Applicants seek exemptive relief from the provisions of section 12(e) to permit Allied Capital, banks, insurance companies and other financial institutions to acquire notes or other evidences of indebtedness issued by Allied Investment, Allied Lending or Allied Development.

Section 17(a) of the Act, as here pertinent, provides that it shall be unlawful for any affiliated person of a registered investment company, or any affiliated person of such person, (1) to sell any security or other property to such registered investment company or to any company controlled by any such registered investment company except securities of which the buyer is the issuer of securities of which the seller is the issuer and which are part of a general offering to holders of a class of its securities, (2) to purchase from a registered investment company, any security or other property (except securities of which the seller is the issuer), or (3) to borrow money or other property from such registered company, with certain exceptions. Section 2(a)(3) of the Act, in pertinent part, defines affiliated person to include: (i) Any person directly or indirectly owning,

controlling, or holding with power to vote 5 per centum or more of the outstanding voting securities of such other person; (ii) any person 5 per centum or more of whose outstanding voting securities are directly or indirectly owned, controlled, or held with power to vote, by such other person; and (iii) any person directly of indirectly controlling, controlled by, or under common control with, such other person. Allied Capital is or will be an affiliated person of Allied lending, Allied Investment, Allied Development by reason of its ownership (or proposed ownership) of all the common stock of Allied Lending, Allied Investment, and Allied Development and its control of those companies. In addition, Allied Capital, Allied Lending, and Allied Development, and Allied Investment are affiliated persons of one another. Accordingly, any exchange of securities or property or any borrowings between Allied Capital and Allied Development would constitute an affiliated transaction prohibited by section 17(a). Therefore, Applicants request exemptive relief to permit transfers of property and securities or the borrowing of money or other property between Allied Capital and Allied Development. Applicants further seek to amend the 1976 Order to permit, subject to condition (7) as hereinafter stated, any person to which loans may be made by Allied Development and which may become an affiliated person of the Applicants, to borrow from, or sell securities issued by it to Allied Capital, Allied Investment, Allied Lending or Allied Development. Applicants state that the modification of the 1976 Order is necessary because Allied Development's proposed lending activites are not limited to persons that are "small business concerns".

Section 17(d) of the Act and Rule 17d-1 thereunder, taken together, provide, among other things, that it shall be unlawful, with certain exceptions, not applicable here, for an affiliated person of any registered investment company, or an affiliated person of such a person, acting as principal, to participate in or effect any transaction in connection with any joint enterprise or arrangement in which any such registered company or a company controlled by such registered company is a participant unless an application regarding such transaction has been granted by the Commission. Applicants state that the 1976 Order granted an exemption from the provisions of section 17(d) of the Act and Rule 17d-1 thereunder to the extent necessary to permit Allied Capital, Allied Investment and Allied Lending to participate in any joint enterprise or joint arrangement involving other participants. Applicants seek to extend such relief to include Allied Development.

Applicants state that the before-mentioned exemptive relief is necessary or appropriate in the public interest and consistent with the protection of investors and the purposes of the Act. Applicants state that in order to protect investors any order of the Commission that may be issued pursuant to this notice of filing of application may be conditioned upon Applicants' compliance with all undertakings as herein stated. Applicants further state that the exemptive relief requested for Allied Development is necessary because the SBA does not permit either Allied Investment or Allied Lending to make loans which are not part of SBA programs.

Applicants have also requested relief from those provisions of the Act governing capital structure. Section 18(a) of the Act makes it unlawful for any registered closed-end investment company to issue or to sell any class of senior security of which it is the issuer, unless the company complies with the asset coverage requirements as set forth in that section. Under the provisions of section 18 applicable to closed-end investment companies, senior securities representing indebtedness must have an asset coverage of 300 percent immediately after their issuance or sale. Senior securities representing stock must have an asset coverage of 200 percent. Section 18(c) of the Act, in pertinent part, makes it unlawful for any registered closed-end investment company to issue or sell any senior security representing indebedness if immediately thereafter such company will have outstanding more than one class of senior security representing indebtedness.

According to the application, the 1976 Order prohibits Allied Capital, Allied Investment and Allied Lending from issuing any senior securities except that: (i) Allied Capital may borrow from either banks or insurance companies, but not both, on the basis of unsecured promissory notes or other unsecured evidences of indebtedness; (ii) Allied Investment may borrow only from the SBA, which borrowing may not be guaranteed by Allied Capital, and from Allied Capital; and (iii) Allied Lending may borrow only from Allied Capital. Applicants state that the 1976 Order also requires that Allied Capital individually, and Allied Capital, Allied Investment and Allied Lending on a consolidated basis, meet the 300 percent asset coverage requirement of section 18(a) immediately after the issuance of any senior securities.

Applicants seek to modify the 1976 Order to permit Allied Capital, Allied Investment, Allied Lending and Allied Development to borrow from banks, insurance companies and other financial institutions, including in the case of the wholly-owned investment company subsidiaries of Allied Capital from Allied Capital, on a secured or unsecured basis and to permit Allied Capital to guarantee the borrowings of such subsidiaries. Applicants represent that all borrowings by Allied capital, Allied Lending and Allied Development except borrowings by Allied Lending, Allied Investment and Allied Development from Allied Capital, will be subject to the asset coverage requirements of section 18(a) of the Act as applied on an individual basis to each company and on a consolidated basis to Allied Captial and its subsidiaries. In addition, any guarantee by Allied Capital of borrowings by its subsidiaries shall not be deemed a senior security and not subject to the asset coverage requirements of section 18(a) provided that 90 percent of Allied Capital's assets are represented by its investments in Allied Lending, Allied Investment and Allied Development or in securities similar to those in which such subsidiaries invest. Furthermore, Applicants state that any borrowings by Allied Invesment are not subject to the section 18(a) asset coverage requirement by reason of the section 18(k) exclusion for SBICs. However, Applicants represent that in applying the section 18(a) asset coverage test on a consolidated basis an amount of assets equal to any such borrowings by Allied Investment shall be excluded. In addition, Applicants request an exemption from the provisions of section 18(c) of the Act to permit Allied Capital and its investment company subsidiaries to have more than one class of senior security representing indebtedness outstanding.

In support of their exemptive request, Applicants state that requiring Allied Capital to borrow and then reloan such funds to its subsidiaries involves considerable duplication of effort. Applicants further state that restricting Allied Capital to borrow only from banks or insurance companies, but not both, and only on an unsecured basis effectively limits the sources of credit available to the Allied Group and hinders it from seeking or obtaining credit on competitive terms. Applicants state that permitting direct borrowings by Allied Lending, Allied Investment and Allied Development will be

consistent with the purposes of and protections of the Act since Allied Capital, Allied Lending, and Allied Development individually, and Allied Capital and its subsidiaries on a consolidated basis, as adjusted for the borrowings of Allied Investment, will still have to meet the 300 percent asset coverage requirement of section 18(a) subject to the above noted exclusions. Applicants state that by excluding an amount of assets equal to the borrowings of Allied Investment for purposes of calculating the asset coverage required by section 18(a), investors are protected from the adverse effects of leveraging. Finally, Applicants contend that the exclusion from the asset coverage requirements of section 18(a) of borrowings from Allied Capital by its investment company subsidiaries and Allied Capital's guarantee of its subsidiaries' borrowings does not impair the protections of section 18 since neither type of senior security (as defined in the Act) increases the aggregate indebtedness incurred by the Allied Group.

Applicants have also requested relief from certain provisions of the Act regarding filings with the Commission and transmittal of reports to shareholders. Section 8(b) of the Act, in pertinent part, requires every registered investment company to file with the Commission a registration statement containing such information and documents as the Commission shall prescribe. Rule 8b-16 thereunder, in part, requires every registered management investment company filing annual reports on Form N-1R to amend its registration statement filed pursuant to section 8(b) not more than 120 days after the close of each fiscal year. Section 30(a) of the Act, requires a registered investment company to file annually with the Commission such information, documents and reports as is required to investment companies subject to section 13(a) of the Securities Exchange Act of 1934. Rule 30a-1 thereunder, requires every registered investment company to file an annual report with the Commission not more than 120 days after the close of each fiscal year. Section 30(d) of the Act, in part, requires every registered investment company to transmit to its stockholders, at least semiannually, reports containing such information and financial statements or their equivalent as the Commission may prescribe. Rule 30d-1 thereunder, in part, requires every registered management investment company to transmit to its stockholders, at least semiannually, a report containing the financial statements required to be included in such reports by the Commission's registration statement from under the Act and prescribes the time within which such report must be transmitted.

Applicants request an exemption from section 8(b) of the Act and Rule 8b-16 thereunder to the extent necessary to permit Allied Capital to file on behalf of itself and Allied Lending and Allied Development amendments to its registration statement filed under the Act containining information with respect to and financial statements of Allied Capital and its subsidiaries on a consolidated basis only. In addition, Applicants request an exemption from the provisions of section 30(a) of the Act and Rule 30a-1 thereunder to the extent necessary to permit Allied Capital to file on behalf of itself and Allied Investment, Allied Lending and Allied Development annual reports on Form N-1R, or appropriate successor form, containing information with respect to Allied Capital and its subsidiaries on a consolidated basis only and a copy of the financial report of Allied Investment filed with the SBA on SBA Form 468. Applicants request that such consolidated forms and amendments shall be in lieu of the separate filing obligations of Allied Investments shall be in lieu of the separate filing obligations of Allied Investment, Allied Lending and Allied Development pursuant to section 30(a) of the act and Rules 8b-16 and 30a-1 thereunder. Applicants also request an exemption from section 30(d) of the act and Rule 30d-1 thereunder to the extent necessary to permit Allied Capital to transmit to its shareholders semiannually reports containing financial information and statements prescribed on a consolidated basis for allied Capital and its subsidiaries. Applicants request that such consolidated report be in lieu of the separate reporting obligations of Allied Development, Allied Investment and Allied Lending.

Applicants agree that separate financial statements will not be required in any amendment filed with the Commission pursuant to Rule 8b-16 under the Act and in any semiannual report to shareholders so long as the amount of Allied Capital's total assets on a consolidated basis invested in assets other than securities of its three investment company subsidiaries do not equal or exceed 10 percent. Applicants further state that in the event 10 percent or more of Allied Capital's total assets on a consolidated basis should be invested in securities othe than those of its three investment company subsidiaries, additional financial statements will be required; namely, combined financial statements of Allied Capital's three investment company subsidiaries and separate financial statements of any other subsidiary in which Allied Capital's investment equals or exceeds 10 percent of its total assets on a consolidated basis.

Applicants state that separate filings for each is burdensome and there is a question as to whether multiple filings provide a convenient source of information to investors. Similarly, Applicants contend that transmitting semiannual reports containing consolidated financial statements will not lessen investors' understanding of the financial position or operations of the Allied Group. In this regard, Applicants state that since Allied Capital and its subsidiaries operate essentially as a single economic unit, consolidated financial statements present the most meaningful financial information for financial reporting purposes.

Section 6(c) of the Act provides, in part, that the Commission upon application, may conditionally or unconditionally exempt any person, security, or transaction, or any class or classes of persons, securities, or transactions, from any provision or provisions of the Act or of any rule or regulation thereunder, if and to the extent that such exemption is necessary or appropriate in the public interest and consistent with the protection of investors and the purposes fairly intended by the policy and provisions of the Act.

Applicants have agreed that any order granted by the Commission on their application may be subject to the following conditions:

1. At all times Allied Capital will own and hold, beneficially and of record, all of the outstanding capital stock of Allied Investment, Allied Lending and Allied Development;

2. Allied Capital will not cause or permit Allied Investment, Allied Lending or Allied Development to change any of their fundamental investment policies, or take any other action referred to in section 13(a) of the Act, unless such action shall have been authorized by Allied Capital after approval of such action by a vote of a majority (as defined in the Act) of the outstanding voting securities of Allied Capital;

3. Allied Capital will not cause or permit Allied Investment, Allied Lending or Allied Development to enter into, renew or perform any investment advisory or underwriting contract or agreement, written or oral, as

contemplated by section 15 of the act, unless the terms of such contracts or agreements and any renewal thereof shall have been approved in compliance with said section 15; and where any vote of the stockholders of Allied Investment, Allied Lending or Allied Development would be required by said section 15 unless the stockholders of Allied Capital also shall have approved the same by vote by a majority (as defined in the Act) of the outstanding voting securities of Allied Capital, or where any action of the directors of Allied Investment, Allied Lending or Allied Development would be required by said section 15, unless the Board of Directors of Allied Capital, including a majority of those directors who are not parties to any such contract or agreement or interested persons of any such party, also shall have approved the same;

4. Allied Capital will not itself, and Allied Capital will not cause or permit Allied Investment, Allied Lending or Allied Development, to issue any security or sell any senior security of which Allied Capital, Allied Investment, Allied Lending or Allied Development is the issuer except as hereinafter set forth:

(a) Allied Capital and each of its investment company subsidiaries may issue and sell to banks, insurance companies and other financial institutions their secured or unsecured promissory notes or other evidences of indebtedness in consideration of any loan, or any extension or renewal thereof made by private arrangement, provided the following conditions are met: (i) such notes or evidences of indebtedness are not intended to be publicly distributed, (ii) such notes or evidences of indebtedness are not convertible into, exchangeable for or accompanied by any options to acquire any equity security, and (iii) Allied Capital and its subsidiaries on a consolidated basis, and Allied Capital, Allied Lending, and Allied Development individually, shall have the asset coverage required by Section 18(a) of the Act immediately after the issuance or sale of any such notes or evidences of indebtedness by any of them, except that, in determining whether Allied Capital and its subsidiaries on a consolidated basis have the asset coverage required by section 18(a), any borrowings by Allied Investment shall not be considered senior securities and, for purposes of the definition of "asset coverage" in section 18(h), shall be treated as indebtedness not represented by senior securities;

(b) In addition, (i) Allied Investment may borrow from the SBA on such basis as the SBA from time to time may lend to SBICs, (ii) Allied Lending, Allied Investment and Allied Development may borrow from Allied Capital, and (iii) Allied Capital may guarantee any borrowings by its subsidiaries, provided that 90% of the total assets of Allied Capital on a consolidated basis are represented by Allied Capital's investments in Allied Lending, Allied Investment and Allied Development or in securities similar to those in which such subsidiaries invest. None of the borrowings or other arrangements permitted by this sub-paragraph (b) shall be deemed senior securities for purposes of this order or section 18 of the Act.

5. Allied Capital will cause to be elected as directors of Allied Investment, Allied Lending and Allied Development only persons who are directors of Allied Capital, elected in compliance with section 16(a) of the Act, and at all times officers of Allied Capital will also be officers of Allied Investment, Allied Lending and Allied Development;

6. Allied Capital will file with the Commission pursuant to Rule 8b-16 amendments to its registration statement pursuant to section 8(b) of the Act on behalf of itself and Allied Lending and Allied Development containing information with respect to and financial statements of Allied Capital and its subsidiaries on a consolidated basis only, such amendments to be in lieu of and in satisfaction of the separate filing obligations of Allied Lending and Allied Development pursuant to Rule 8b-16. Allied Capital will file with the Commission pursuant to section 30(a) of the Act annual reports on Form N-1R, or appropriate successor form, on behalf of itself and Allied Investment, Allied Lending and Allied Development containing information with respect to Allied Capital and its subsidiaries on a consolidated basis only and a copy of the financial report of Allied Investment filed with the SBA on SBA Form 468, or appropriate successor form, such consolidated annual reports to be in lieu of and in satisfaction of the separate filing obligations of Allied Investment, Allied Lending and Allied Development pursuant to section 30(a). Allied Capital will transmit to its stockholders semi-annually pursuant to section 30(d) of the Act reports containing the financial information and statements prescribed and required by such section for Allied Capital and its subsidiaries on a consolidated basis only, which reports shall be in lieu of and in satisfaction of the separate reporting obligations of Allied Investment, Allied Lending and Allied Development pursuant to section 30(d); provided, however, that if 10 percent or more of Allied Capital's total assets on a consolidated basis are invested in assets other than securities issued by Allied Lending, Allied Investment, or Allied Development or securities similar to those in which such subsidiaries invest, then, in addition to the consolidated financial statements of Allied Capital and its subsidiaries, there shall be included in such reports combined financial statements of Allied Lending, Allied Investment, and Allied Development and separate financial statements of any subsidiary other than the aforementioned subsidiaries if Allied Capital's investment in such subsidiary amounts to 10 percent or more of Allied Capital's total assets on a consolidated basis. Allied Capital will also cause Allied Investment and Allied Lending to file with the Commission copies of all reports which they are required to file with the SBA. Any independent public accountant who signs a financial statement filed by Allied Capital, Allied Investment, Allied Lending or Allied Development with the Commission shall be selected and approved in compliance with section 32(a) of the Act by holders of a majority (as defined in the Act) of Allied Capital's outstanding voting securities;

7. Any small business concern or other concern to which loans may be made Allied Capital, Allied Investment, Allied Lending or Allied Development which concern may become an affiliated person of the Allied Group may borrow from, or sell securities issued by it to, Allied Capital, Allied Investment, Allied Lending or Allied Development, provided that such transaction meets the requirements for an exemption pursuant to Rule 17a-6 promulgated pursuant to the Act, except to the extent that it fails to meet the requirements of such Rule solely because another member of the Allied Group is also a party to the transaction or has, or within six months prior to the transaction had, or pursuant to an arrangement will acquire, a direct of indirect financial interest in the small business or other concern;

8. The Allied Group, or any member thereof, may participate in any joint enterprise or joint arrangement involving other participants, provided that such transaction meets the requirements for an exemption pursuant to Rule 17d-1 except to the extent it fails to meet the requirements of such Rule solely because other members of the Allied Group are, or propose to be, participants in the joint enterprise or joint arrangement.

Notice is further given that any interested person may, not later than May 10, at 5:30 p.m., submit to the Commission in writing a request for a hearing on the application accompanied by a statement as to the nature of his interest, the reason for such request, and the issues, if any, of fact or law proposed to be controverted, or he may request that he be notified if the Commission shall order a hearing thereon. Any such communication should be addressed: Secretary, Securities and Exchange Commission, Washington, D.C. 20549. A copy of such request shall be served personally or by mail upon Applicants at the address stated above. Proof of such service (by affidavit or, in the case of an attorney-at-law, by certificate) shall be filed contemporaneously with the request. As provided by Rule 0-5 of the rules and regulations promulgated under the Act, an order disposing of the application will be issued as of course following said date unless the Commission thereafter orders a hearing upon request or upon the Commission's own motion. Persons who request a hearing, or advice as to whether a hearing is ordered, will receive any notices and orders issued in this matter, including the date of the hearing (if ordered) and any postponements thereof.

For the Commission, by the Division of Investment Management, pursuant to delegated authority.

George A. Fitzsimmons,

Secretary.

[FR Doc. 82-10825 Filed 4-20-82; 8:45 am]

BILLING CODE 8010-01-M

[Rel. No. 12372; 812-5101]

Chancellor Cash Fund, Inc., et al.; Filing of Application

April 14, 1982.

Notice is hereby given that Chancellor Cash Fund, Inc. ("Cash Fund"), Chancellor Equity Fund, Inc. ("Equity Fund") and Chancellor Quality Income Fund, Inc. ("Income Fund") (collectively, the "Funds"), each of which is registered under the Investment Company Act of 1940 ("Act") as a diversified, open-end, management investment company, and Bache Halsey Stuart Shields Incorporated ("Bache"), 100 Gold Street, New York, NY 10292, a wholly-owned subsidiary of the Prudential Insurance Company of America ("Prudential") and the proposed principal underwriter of the Funds, filed an application on February 3, 1982, and amendments thereto on March 4 and April 13, 1982, for an order pursuant to section 6(c) of the Act declaring that the Honorable Terry Sanford, a proposed director of each Fund, shall not, solely by reason of his status as a director of the International Telephone & Telegraph Corporation ("ITT"), be deemed to be an "interested person" within the meaning of section 2(a)(19) of the Act of any of the Funds, Prudential, Bache, or of any other investment company for which Prudential or Bache acts or may act as investment adviser or principal underwriter and on whose board of directors Mr. Sanford may serve. All interested persons are referred to the application on file with the Commission for a statement of the representations contained therein, which are summarized below.

According to the application, Cash Fund previously operated under the name, Asset Reserves Inc., as a "money market" fund from May 19 to August 4, 1980, when it terminated operations. Cash Fund states that on that date, substantially all of its assets were transferred to another money market fund as part of an offer of exchange of shares made to the shareholders of Cash Fund which left Bache as its sole remaining shareholder. Cash Fund further states that on January 25, 1982, Bache, as sole shareholder of Cash Fund, elected a new board of directors of Cash Fund, consisting of five members, including two members who were interested persons and three members who were not interested persons, including Mr. Sanford. Cash Fund further represents that it has filed with the Commission a post-effective amendment to its Registration Statement on Form N-1, upon the effectiveness of which Cash Fund intends to recommence operations as a money market fund. On January 25, 1982, Bache, as sole shareholder of Cash Fund, and Cash Fund's directors approved a proposed investment advisory agreement to be entered into with Prudential and a proposed distribution agreement to be entered into with Bache.

Equity Fund and Income Fund state that they were incorporated in the State of Maryland and that they have filed separate Registration Statements on Form N-1 with the Commission as open-end, diversified, management investment companies. Equity Fund and Income Fund Further state that on January 25, 1982, their original boards of directors voted to increase and change the composition of their respective boards to consist of five members, including two members who were interested persons and three members were not interested persons, including Mr. Sanford as a person who was not an interested person. Thereafter, on the same date, Bache, as the sole shareholder of both Equity Fund and Income Fund and the newly constituted boards of directors for Equity Fund and Income Fund approved separate investment advisory agreements and distribution agreements to be entered into with Prudential and Bache, respectively.

The Funds further state that Mr. Sanford, who is the President of Duke University and a former Governor of North Carolina, is also a director of ITT. According to the application, ITT has three indirect subsidiaries, namely, ITT Financial Securities, Inc. ("Financial"), Hartford Securities Co., Inc. ("Hartford") and Hartford Equity Sales Co., Inc. ("Equity Sales") (collectively the "Broker-Dealer Subsidiaries"), which are special purpose broker-dealers registered under the Securities Exchange Act of 1934. Financial, a third tier subsidiary of ITT, acts solely as dealer for commercial paper issued by ITT and by ITT Financial Corp., a second-tier subsidiary of ITT. Hartford, a second-tier subsidiary of ITT, acts as broker-dealer exclusively for securities transactions executed for the accounts of the Hartford group of insurance companies and is a member of regional securities exchanges. Equity Sales, a sixth-tier subsidiary of ITT, sells only variable annuity accounts in Hartford Fund, Inc., an investment company registered under the Act, and fixed annuities. The Funds and Bache further state that except as described above, none of ITT's Broker-Dealer Subsidiaries are members of securities exchanges, make markets in securities, execute or clear securities transactions or are otherwise engaged in the securities business. According to the application, for the year 1981, the net income of all three Broker-Dealer Subsidiaries represented less than 1 percent of the consolidated net income of ITT. The Fund's further state that Mr. Sanford is not a director of any direct or indirect ITT subsidiary and owns a de minimis amount of ITT stock.

The Funds and Bache state that on February 23, 1982, Mr. Sanford tendered his resignation as a director of each of the Funds and was replaced by a new director. The Funds and Bache further state that Mr. Sanford has consented to become a sixth director of each of the Funds upon receipt of an order of the Commission declaring that Mr. Sanford is not an "interested person" as defined in section 2(a)(19) of the Act. Mr. Sanford may also serve as a director of other investment companies for which Prudential or Bache may act as

investment adviser or principal underwriter ("Other Sponsored Funds").

Section 2(a)(19)(A)(v) and (B)(v) of the Act define an "interested person" of an investment company, investment adviser or principal underwriter, to include any broker or dealer registered under the Securities Exchange Act of 1934 or any affiliated person of such broker or dealer. Section 2(a)(3) of the Act defines an "affiliated person" of another person to include, *inter alia*, any director, officer or employee of such other person. The Funds and Bache submit that by virtue of his status as a director of ITT, Mr. Sanford is an affiliated person of ITT which, in turn, is an affiliated person of its Broker-Dealer Subsidiaries by virtue of controlling 5 percent or more of their outstanding voting securities. Without admitting that Mr. Sanford is an interested person of the Funds, Prudential or Bache by virtue of his indirect connection with ITT's Broker-Dealer Subsidiaries, the Funds and Bache have filed an application to eliminate the risk of that conclusion by the receipt of an order of the Commission declaring that Mr. Sanford is not an interested person of the Funds, Prudential, Bache or the Other Sponsored Funds solely by reason of his indirect connection to ITT's Broker-Dealer Subsidiaries.

The Funds and Bache believe that the exemptive order requested is necessary and appropriate in the public interest and consistent with the protection of investors and the purposes fairly intended by the policies and provisions of the Act. In this regard the Funds and Bache assert that each of ITT's Broker-Dealer Subsidiaries is a special purpose broker-dealer which deals solely in commercial paper for ITT and a subsidiary thereof, executes securities transactions exclusively for certain other ITT subsidiaries and sells only variable and fixed annuity contracts, respectively. None would, therefore, be able to execute portfolio transactions for the Funds or distribute shares of the Funds. In the event that in the future any of the three Broker Dealer Subsidiaries expands its activities so that it could do business with the Funds or Bache, the Funds and Bache represent that they will not carry on any business with any of the Broker-Dealer Subsidiaries. The Funds and Bache further assert that Mr. Sanford's position as a director of the Funds, ITT and Other Sponsored Funds would not present the potential for conflict of interest against which the provisions of the Act relating to interested persons were designed to guard.

The Funds and Bache believe that Mr. Sanford is well qualified to be, and would, in fact, be, an independent director and that his relationship with the Funds, Other Sponsored Funds, Bache and Prudential would in no way be altered by his affiliation with ITT. The Funds and Bache further believe that Mr. Sanford would be able to discharge his duties as a director free from any conflicts of interest insofar as the Funds, Other Sponsored Funds Bache and Prudential are concerned and that he would exercise the impartial judgment on behalf of the Funds and their shareholders which is expected of an independent director in dealing with Bache and Prudential. According to the application, this is not only because ITT's Broker-Dealer Subsidiaries could not act as broker-dealers for the Funds but also because Mr. Sanford's interest in such subsidiaries is, at best, remote in that they constitute a de minimis portion of the business of ITT and Mr. Sanford has no involvement with their operations. The Funds and Bache further state that, Mr. Sanford's ownership of shares of ITT is de minimis and his financial stake in the three Broker-Dealer Subsidiaries is, therefore, also de minimis.

Section 6(c) of the Act provides, in pertinent part, that the Commission, by order upon application, may conditionally or unconditionally exempt any person from any provision of the Act, if and to the extent that such exemption is necessary or appropriate in the public interest and consistent with the protection of investors and the purposes fairly intended by the policy and provisions of the Act.

Notice is further given that any interested person may, not later than May 10, 1982, at 5:30 p.m., submit to the Commission in writing a request for a hearing on the application accompanied by a statement as to the nature of his interest, the reasons for such request, and the issues, if any, of fact or law proposed to be controverted, or he may request that he be notified if the Commission shall order a hearing thereon. Any such communication should be addressed: Secretary, Securities and Exchange Commission, Washington, D.C. 20549. A copy of such request shall be served personally or by mail upon the Funds and Bache at the address stated above. Proof of such service (by affidavit or, in the case of an attorney-at-law, by certificate) shall be filed contemporaneously with the request. As provided by Rule 0-5 of the Rules and Regulations promulgated under the Act, an order disposing of the application herein will be issued as of course following said date unless the Commission thereafter orders a hearing upon request or upon the Commission's own motion. Persons who request a hearing, or advice as to whether a hearing is ordered, will receive any notices and orders issued in this matter, including the date of the hearing (if ordered) and any postponements thereof.

For the Commission, by the Division of Investment Management, pursuant to delegated authority.

George A. Fitzsimmons,

Secretary.

[FR Doc. 82-10826 Filed 4-20-82; 8:45 am]

BILLING CODE 8010-01-M

[Rel. No. 12374; 812-5118]

Gotham Funds, Filing of Applications

April 14, 1982.

Notice is hereby given that Gotham Funds ("Applicant"), One Boston Place, Boston, Massachusetts 02106, an open-end, diversified management investment company of the "series" type, filed an application on February 24, 1982, for an order of the Commission pursuant to section 6(c) of the Investment Company Act of 1940 ("Act") exempting Applicant from the provisions of section 2(a)(41) of the Act and Rules 2a-4 and 22c-1 thereunder to the extent necessary to permit Applicant to compute the net asset value per share of its Gotham Money Market Fund series (the "Money Fund") and its Gotham Federal Money Market Fund series (the "Federal Money Fund", and collectively referred to with the Money Fund as the "Funds") using the amortized cost method of valuing portfolio securities. All interested persons are referred to the application on file with the Commission for a statement of the representations therein, which are summarized below.

Applicant was organized as a business trust under the laws of the Commonwealth of Massachusetts on February 18, 1982, and filed a registration statement under the Securities Act of 1933 and under the Act on Form N-1 on February 19, 1982. It was established to serve as an investment medium for customers, cardmembers, and employees of American Express Company, to whom shares will be offered without imposition of a sales charge. Applicant states that it will employ The Boston Company Advisors, Inc., an indirect wholly-owned subsidiary of American Express Company, as its investment adviser. The principal underwriter for the Applicant is American Express

Service Corporation, a wholly-owned subsidiary of American Express Company and an affiliate of The Boston Company Advisors, Inc.

Applicant states that the investment objective of the Money Fund is to achieve as high a rate of current income as inconsistent with the preservation of capital and the maintenance of liquidity by investing in money market instruments consiting exclusively of: securities issued or guaranteed as to principal by the Government of the United States or by its agencies or instrumentalities; certificates of deposit issued by domestic banks, provided that certificates issued by banks with less than $1 billion in assets ("small issuer CDs") may be purchased so long as the issuer is a member of the Federal Deposit Insurance Corporation ("FDIC") and so long as the principal amount of the small issuer CD is fully insured by the FDIC and no more than $100,000 in principal amount of small issuer CDs issued by a bank is held by the Money Fund at any one time; domestic bankers' acceptances; high grade corporate obligations with remaining maturities of less than one year; prime commercial paper; and repurchase agreements with respect to any of the foregoing types of instruments.

The Applicant represents that the investment objective of the Federal Money Fund is to seek as high a level of current income as is consistent with the highest level of security of principal and liquidity by investing only in short-term money money market instruments issued or guaranteed as to principal and interest by the Government of the United States or its agencies or instrumentalities ("government money market instruments") except in the case of small issuer CDs as to which only the principal is guaranteed. The Federal Money Fund's purchases of small issuer CDs will be limited to the insured amount of principal ($100,000) in each case; if the principal amount and accrued interest together exceed $100,000, then the excess accrued interest will not be insured. Except to the extent that The Boston Company Advisors, Inc., determines that a readily available market exists for small issuer CDs, the Federal Money Fund will limit its purchases of those instruments together with other illiquid securities (including repurchase agreement maturing in more than seven days) in the aggregate to 10 percent or less of the Fund's total assets. Among the government money market instruments in which the Federal Money Fund will invest are: (1) a variety of United States Treasury securities that differ only in their interest rates, maturities and dates of issuance; (2) securities issued or guaranteed by the Federal Housing Administration, Farmers Home Administration, Export-Import Bank of the United States, Small Business Administration, Government National Mortgage Association, General Services Administration, Central Bank for Cooperatives, Federal Home Loan Banks, Federal Loan Mortgage Corporation, Federal Intermediate Credit Banks, Federal Land Banks, Maritime Administration, The Tennessee Valley Authority, District of Columbia Armory Board, Federal National Mortgage Association and the International Bank for Reconstruction and Development. The Fund may also invest in qualified repurchase agreements secured only by U.S. Government obligations. Instruments subject to repurchase agreements may bear maturities in excess of one year.

Applicant seeks an order of the Commission pursuant to section 6(c) of the Act exempting it from the provisions of section 2(a)(41) of the Act and Rules 2a-4 and 22c-1 thereunder to the extent necessary to permit the Funds' portfolio investments to be valued according to the amortized cost valuation method. As here pertinent, section 2(a)(41) of the Act defines value to mean: (1) With respect to securities for which market quotations are readily available, the market value of such securities, and (2) with respect to other securities and assets, fair value as determined in good faith by the board of directors. Rule 22c-1 adopted under the Act provides, in part, that no registered investment company or principal underwriter therefor issuing any redeemable security shall sell, redeem or repurchase any such security except at a price based on the current net asset value of such security which is next computed after receipt of a tender of such security for redemption or of an order to purchase or sell such security.

Rule 2a-4 adopted under the Act provides, as here relevant, that the "current net asset value" of a redeemable security issued by a registered investment company used in computing its price for the purposes of distribution, redemption and repurchase shall be an amount which reflects calculations made substantially in accordance with the provisions of that rule, with estimates used where necessary or appropriate. Rule 2a-4 further states that portfolio securities with respect to which market quotations are readily available shall be valued at current market value, and other securities and assets shall be valued at fair value as determined in good faith by the board of directors of the investment company. Prior to the filing of the application, the Commission expressed its view that, among other things, (1) Rule 2a-4 under the Act requires that portfolio instruments of "money-market" funds be valued with reference to market factors, and (2) it would be inconsistent, generally, with the provisions of Rule 2a-4 for a "money market" fund to value its portfolio instruments on an amortized cost basis (Investment Company Act Release No. 9786, May 31, 1977).

Section 6(c) of the Act provides, in pertinent part, that the Commission by order upon application, may conditionally or unconditionally exempt any person, security or transaction, or any class or classes of persons, securities or transactions, from any provision or provisions of the Act or of any rule or regulation thereunder, if and to the extent that such exemption is necessary or appropriate in the public interest and consistent with the protection of investors and the purposes fairly intended by the policy and provisions of the Act.

Applicant states that in order for the Funds to be in a position to meet the needs and expectations of potential investors and to offer its shareholders relative stability of principal and a steady flow of predictable income at currently competitive rates, the Funds must be able to price their portfolios at amortized cost.

Applicant has agreed that the following conditions may be imposed in any order of the Commission granting the exemptive relief requested:

1. In supervising the operations of the Funds and delegating special responsibilities involving portfolio management to Applicant's investment manager, Applicant's Trustees undertake—as a particular responsibility within their overall duty of care owed to shareholders of the Funds—to establish procedures reasonably designed, taking into account current market conditions and the Funds' investment objective, to stabilize the Funds' net asset value per share, as computed for the purpose of distribution, redemption and repurchase, at $1.00 per share.

2. Included within the procedures to be adopted by the Trustees of the Applicant shall be the following:

(a) Review by the Trustees, as they deem appropriate and at such intervals as are reasonable in light of current market conditions, to determine the extent of deviation, if any, of the net asset value per share as determined by

using available market quotations from the Funds' $1.00 amortized cost price per share, and maintenance of records of such review.[1]

(b) In the event such deviation from the Funds' $1.00 amortized cost price per share exceeds ½ of 1 percent, a requirement that the Trustees will promptly consider what action, if any, should be initiated.

(c) Where the Trustees believe that the extent of any deviation from the Fund's $1.00 amortized cost price per share may result in material dilution or other unfair results to investors or existing shareholders, they shall take such action as they deem appropriate to eliminate or to reduce to the extent reasonably practicable such dilution or unfair results, which action may include: redeeming shares in kind; selling portfolio instruments prior to maturity to realize capital gains or losses, or shorten the average portfolio maturity of the Funds; withholding dividends; or utilizing a net asset value pe share as determined by using available market quotations.

3. The Funds will maintain a dollar-weighted average portfolio maturity appropriate to their objective of maintaining a stable net asset value per share; provided, however, that the Funds will not (a) purchase any instrument with a remaining maturity of greater than one year, or (b) maintain a dollar-weighted average portfolio maturity which exceeds 120 days.[2]

4. Applicant will record, maintain and preserve permanently in an easily accessible place a written copy of the procedures (and any modifications thereto) described in condition 1 above, and Applicant will record, maintain and preserve for a period of not less than six years (the first two years in an easily accessible place) a written record of the Trustees' considerations and actions taken in connection with the discharge of their responsibilities, as set forth above, to be included in the minutes of the Trustees' meetings. The documents preserved pursuant to this condition shall be subject to inspection by the Commission in accordance with section 31(b) of the Act as though such documents were records required to be maintained pursuant to rules adopted under section 31(a) of the Act.

5. Applicant will limit the Fund's portfolio investments, including repurchase agreements, to those U.S. dollar-denominated instruments which the Trustees determine present minimal credit risks, and which are of high quality as determined by any major rating service, or, in the case of any instrument that is not rated, or comparable quality as determined by the Trustees.

6. Applicant will include in each quarterly report, as an attachment to Form N-1Q, a statement as to whether any action pursuant to condition 2(c) was taken during the preceding fiscal quarter, and, if any action was taken, will describe the nature and circumstances of such action.

Applicant submits that granting its requested exemptive order is appropriate in the public inerest and consistent with the protection of investors and the purposes fairly intended by the policy and provisions of the Act.

Notice is further given that any interested person may, not later than May 10, 1982, at 5:30 p.m., submit to the Commission in writing a request for a hearing on the application accompanied by a statement as to the nature of his interest, the reasons for such request, and the issues, if any, of fact or law proposed to be controverted, or he may request that he be notified if the Commission shall order a hearing thereon. Any such communication should be addressed: Secretary, Securities and Exchange Commission, Washington, D.C. 20549. A copy of such request shall be served personally or by mail upon Applicant at the address stated above. Proof of such service (by affidavit or, in the case of an attorney-at-law, by certificate) shall be filed contemporaneously with the request. As provided by Rule 0-5 of the Rules and Regulations promulgated under the Act, an order disposing of the application herein will be issued as of course following said date unless the Commission thereafter orders a hearing upon request or upon the Commission's own motion. Persons who request a hearing, or advice as to whether a hearing is ordered, will receive any notices and orders issued in this matter, including the date of the hearing (if ordered) and any postponements thereof.

For the Commission, by the Division of Investment Management, pursuant to delegated authority.

George A. Fitzsimmons,

Secretary.

[FR Doc. 82-10827 Filed 4-20-82; 8:45 am]

BILLING CODE 8010-01-M

---

[1] To fulfill this condition, Applicant states that it intends to use actual quotations or estimates of market value reflecting current market conditions chosen by the Trustees in the exercise of their discretion to be appropriate indicators of value, which may include, among others, (i) quotations or estimates of market value for individual portfolio instruments, or (ii) values obtained from yield data relating to classes of money market instruments published by reputable sources.

[2] In fulfilling this condition, if the disposition of a portfolio instrument results in a dollar-weighted average portfolio maturity in excess of 120 days, the Funds will invest their available cash in such a manner as to reduce the dollar-weighted average portfolio maturity to 120 days or less as soon as reasonably practicable.

[Release No. 34-18637; File No. SR-MSTC-82-5]

Self-Regulatory Organizations; Proposed Rule Change By Midwest Securities Trust Co.; Transfer Agent Reject Fees

Comments requested on or before May 12, 1982.

Pursuant to section 19(b)(1) of the Securities Exchange Act of 1934, 15 U.S.C. 78s(b)(1), notice is hereby given that on April 5, 1982 the Midwest Securities Trust Company filed with the Securities and Exchange Commission the proposed rule change as described in Items I, II, and III below, which Items have been prepared by the self-regulatory organization. The Commission is publishing this notice to solicit comments on the proposed rule change from interested persons.

I. Self-Regulatory Organization's Statement of the Terms of Substance of the Proposed Rule Change

Attached to the filing as Exhibit A is the MST Administrative Bulletin dated March 31, 1982.

II. Self-Regulatory Organization's Statement of the Purpose of, and Statutory Basis for, the Proposed Rule Change

In its filing with the Commission, the self-regulatory organization included statements concerning the purpose of and basis for the proposed rule change and discussed any comments it received on the proposed rule change. The text of these statements may be examined at the places specified in Item IV below. The self-regulatory organization has prepared summaries, set forth in Sections (A), (B) and (C) below, of the most significant aspects of such statements.

(A) Self-Regulatory Organization's Statement of the Purpose of, and Statutory Basis for, the Proposed Rule Change

The purpose of the proposed rule change is to pass along charges by transfer agents for rejections or corrections of transfer items attributable to participant errors or omissions. However, if deposits, legal transfer items, or transfer withdrawal requests

which have been accepted by MSTC for processing are subsequently rejected by the transfer agent for errors which MSTC should have discovered, MSTC will absorb the transfer agent reject fee.

The charges passed on to participants will be included in their monthly billing statements as a "miscellaneous charge" and participants will be sent a memo bill with copies of any related papers which MSTC received from the transfer agent to support the charge.

The rule change is consistent with Section 17A of the Act in that participants will be charged only for their errors or omissions and therefore the passed-on transfer agent reject fees are an equitable allocation of reasonable fees for services rendered on behalf of participants.

(B) Self-Regulatory Organization's Statement on Burden on Competition

The Midwest Securities Trust Company does not believe that any burdens will be placed on competition as a result of the proposed rule change.

(C) Self-Regulatory Organization's Statement on Comments on the Proposed Rule Change Received From Members, Participants or Others

Comments have neither been solicited nor received.

III. Date of Effectiveness of the Proposed Rule Change and Timing for Commission Action

The foregoing rule change has become effective pursuant to section 19(b)(3) of the Securities Exchange Act of 1934 and subparagraph (e) of Securities Exchange Act Rule 19b-4. At any time within 60 days of the filing of such proposed rule change, the Commission may summarily abrogate such rule change if it appears to the Commission that such action is necessary or appropriate in the public interest, for the protection of investors, or otherwise in furtherance of the purposes of the Securities Exchange Act.

IV. Solicitation of Comments

Interested persons are invited to submit written data, views and arguments concerning the foregoing. Persons making written submissions should file six copies thereof with the Secretary, Securities and Exchange Commission, 500 North Capitol Street, N.W., Washington, D.C. 20549. Copies of the submission, all subsequent amendments, all written statements with respect to the proposed rule change that are filed with the Commission, and all written communications relating to the proposed rule change between the Commission and any person, other than those that may be withheld from the public in accordance with the provisions of 5 U.S.C. 552, will be available for inspection and copying in the Commission's Public Reference Section, 1100 L Street, NW., Washington, D.C. Copies of such filing will also be available for inspection and copying at the principal office of the above-mentioned self-regulatory organization. All submissions should refer to the file number in the caption above and should be submitted on or before May 12, 1982.

For the Commission by the Division of Market Regulation, pursuant to delegated authority.

George A. Fitzsimmons,

Secretary.

April 12, 1982.

Exhibit A

MST System Administrative Bulletin

March 31, 1982.

To: All participants.

Attention: Senior operations manager or partner.

Subject: Transfer agent reject fees.

The transfer agent community has recently instituted fees for corrections or rejections of transfer items. These fees range from $2.00 to $25.00 per occurrence, with an average of $4.00 to $5.00. Beginning on April 1, 1982, MSTC will pass along to participants those fees which will not pass on to participants are attributable to participant errors or omissions. MSTC will not pass on to participants those fees for which MSTC has caused the error.

MSTC conducts a good delivery check on *every* deposit submitted by a participant. The current and continuing policy is to reject immediately any deposits to participants which are not in good delivery form and which cannot be put into good form by MSTC. Therefore, the number of items subsequently rejected by a transfer agent because a "signature guarantee," "transfer tax," etc. are missing should be small. This unique "good delivery" service provided by MSTC should continue to keep the number of transfer agent rejects at a minimum.

Another service currently provided by MSTC is the review of all transfer withdrawal requests received from participants prior to submission to the transfer agent. Through this review, MSTC presents "acceptable registrations" to the agent on behalf of participants. Examples of this service include: stamping the transfer fanfold "small town" where no street address is present; or reformatting "gifts to minors" registrations to conform with industry requirements. MSTC will continue to provide this unique transfer fanfold edit service for participants. However, the transfer agents' requirements are becoming very strict on registration and a fee may have to be imposed on this service in the future if inconsistency exists with the various agents. Participants should review registration requirements in the Stock Transfer Association Guide (Paragraph Number 110). In any case, this service should also keep the number of transfer agent rejects to a minimum.

MSTC also provides a unique legal transfer service for participants whereby same day credit is provided for legal items. MSTC performs a legal good delivery review of all items and determines if additional forms or documentation is required. If, *in the opinion of MSTC,* additional papers are required, MSTC will reject the item immediately to the participant. If the item is accepted by MSTC and is subsequently rejected by the agent, MSTC will absorb the transfer agent's reject fee, which is usually $12.50 to $25.00. (The actual certificates will be returned to the participant for correction.)

The charges to be passed on to participants will be included in the monthly MCC/MSTC billing statement as a miscellaneous charge. On approximately the tenth business day of each month, a "memo" bill will be included in the participants' daily package from MCC/MSTC. Attached to this memo bill will be copies of any related papers which MSTC received from the transfer agents to support the charge.

MSTC anticipates that the actual charges passed on to participants will be minimal because of the unique services provided by MSTC on deposits, transfer withdrawals and legal items. Our reputation with the transfer agents is good because of the special handling which we give to all items before sending them to the agents. We expect to receive continued cooperation from participants in presenting their items to MSTC, especially in the area of transfer withdrawal requests where special programming to correct errors in the registration field may be needed by participants.

MSTC would be pleased to discuss our unique services or these charges with any interested party. Questions may be directed to the undersigned at (312) 368-2530, to Lou Viola, MSTC Transfer Manager, at (312) 368-2434, or to your MCC/MSTC Account Manager.

Gerald R. Broz,

Vice President, MCC/MSTC.

[FR Doc. 82-10828 Filed 4-20-82; 8:45 am]

BILLING CODE 8010-01-M

[Rel. No. 18644; File No. SR-NYSE-82-7]

Self-Regulatory Organizations; Filing of Proposed Rule Change by the New York Stock Exchange, Inc.

April 14, 1982.

The New York Stock Exchange, Inc. ("NYSE") submitted on April 12, 1982, copies of a proposed rule change pursuant to section 19(b)(1) of the Securities Exchange Act of 1934 (the "Act") and Rule 19b-4 thereunder, to amend various NYSE rules relating to exchange members and statements of accounts to customers. The proposed rule changes would remove the provisions in Rule 314 related to guarantees of loss of sole exchange

members and limited partners, and minimum salary/participation requirements for members and allied members; rescind the requirements specifically related to insurance sales activities (Rule 318); require written requests by member organization personnel to engage in outside financially related employment/activities (Rule 346); clarify a reference to "stockholder" (Rule 409); and amend Rule 411 to permit member organizations to record transactions as of settlement date, require settlement dates to appear on confirmations of transactions sent to customers, and reposition the Supplementary Material into other rules (Rules 36 and 409).

Publication of the submission is expected to be made in the **Federal Register** during the week of April 19, 1982. In order to assist the Commission in determining whether to approve the proposed rule change or institute proceedings to determine whether the proposed rule change should be disapproved, interested persons are invited to submit written data, views and arguments concerning the submission on or before May 12, 1982. Persons desiring to make written comments should file six copies thereof with the Secretary of the Commission, Securities and Exchange Commission, 500 North Capitol Street, Washington, D.C. 20549. Reference should be made to File No. SR-NYSE-82-7.

Copies of the submission, all subsequent amendments, all written statements with respect to the proposed rule change which are filed with the Commission, and all written communications relating to the proposed rule change between the Commission and any person, other than those which may be withheld from the public in accordance with the provisions of 5 U.S.C. 552, will be available for inspection and copying at the Commission's Public Reference Room, 1100 L Street, N.W., Washington, D.C. Copies of the filing and of any subsequent amendments also will be available at the principal office of the above-mentioned self-regulatory organization.

For the Commission, by the Division of Market Regulation pursuant to delegated authority.

George A. Fitzsimmons,

Secretary.

[FR Doc. 82-10829 Filed 4-20-82; 8:45 am]

BILLING CODE 8010-01-M

Boston Stock Exchange, Inc.; Applications for Unlisted Trading Privileges and of Opportunity for Hearing

April 15, 1982.

The above named national securities exchange has filed applications with the Securities and Exchange Commission pursuant to Section 12(f)(1)(B) of the Securities and Exchange Act of 1934 and Rule 12f-1 thereunder, for unlisted trading privileges in the following stocks:

Baldwin-United Corp.
Common Stock $1 Par Value (File No. 7-6206)
Designcraft Jewel Industries, Inc.
Common Stock, $.50 Par Value (File No. 7-6207)
Hitachi, Ltd.
American Depository Shares, Representing Common Stock (50 Yen Par Value) File No. 7-6208)

These securities are listed and registered on one or more other national securities exchanges and are reported in the consolidated transaction reporting system.

Interested persons are invited to submit on or before May 6, 1982 written data, views and arguments concerning the above-referenced applications. Persons desiring to make written comments should file three copies thereof with the Secretary of the Securities and Exchange Commission, Washington, D.C. 20549. Following this opportunity for hearing, the Commission will approve the applications if it finds, based upon all the information available to it that the extensions of unlisted trading privileges pursuant to such applications are consistent with the maintenance of fair and orderly markets and the protection of investors.

For the Commission, by the Division of Market Regulation, pursuant to delegated authority.

George A. Fitzsimmons

Secretary.

[FR Doc. 82-10916 Filed 4-20-82; 8:45 am]

BILLING CODE 8010-01-M

[File No. 1-7769]

Clabir Corp, Common Stock, $.10 Par Value; Application To Withdraw From Listing and Registration

April 16, 1982.

The above named issuer has filed an application with the Securties and Exchange Commission pursuant to Section 12(d) of the Securities Exchange Act of 1934 ("Act") and Rule 12d2-2(d) promulgated thereunder, to withdraw the specified security from listing and registration on the American Stock Exchange, Inc. ("Amex").

The reasons alleged in the application for withdrawing this security from listing and registration include the following:

1. The common stock of Clabir Corporation ("Company") is listed and registered on the Amex. Pursuant to a Registration Statement on Form 8-A which became effective on November 3, 1981, the Company is also listed and registered on the New York Stock Exchange ("NYSE"). The Company has determined that the direct and indirect costs and expenses do not justify maintaining the dual listing of the common stock on the Amex and the NYSE.

2. This application relates solely to withdrawal of the common stock from listing and registration on the Amex and shall have no effect upon the continued listing of such stock on the NYSE. The Amex has posed no objection to this matter.

Any interested person may, on or before May 6, 1982 submit by letter to the Secretary of the Securities and Exchange Commission, Washington, D.C. 20549, facts bearing upon whether the application has been made in accordance with the rules of the Exchange and what terms, if any, should be imposed by the Commission for protection of investors. The Commission, based on the information submitted to it, will issue an order granting the application after the date mentioned above, unless the Commission determines to order a hearing on the matter.

For the Commission, by the Division of Market Regulations, pursuant to delegated authority.

George A. Fitzsimmons

Secretary.

[FR Doc. 82-10910 Filed 4-20-82; 8:45 am]

BILLING CODE 8010-01-M

[File No. 22-11568]

Cummins Engine Co., Inc.; Application and Opportunity for Hearing

April 16, 1982.

Notice is hereby given that Cummins Engine Company, Inc. (the "Company") has filed an application pursuant to clause (ii) of Section 310(b)(1) of the Trust Indenture Act of 1939 (The "Act") for a finding by the Commission that the trusteeship of The Chase Manhattan Bank (National Association) ("Chase") under an indenture of the Company dated as of October 15, 1970 (the "1970 Indenture") which was heretofore

qualified under the Act, and the trusteeship by Chase under an Indenture dated as of December 15, 1981 of Cummins Overseas Finance N.V. and the Company, as Guarantor (the "New Indenture") which is not qualified under the Act, is not so likely to involve a material conflict of interest as to make it necessary in the public interest or for the protection of investors to disqualify Chase from acting as Trustee under the 1970 Indenture and under the New Indenture.

Section 310(b) of the Act, which included in Section 608 of the 1970 Indenture, provides in part that if a trustee under an indenture qualified under the Act has or shall acquire any conflicting interest (as defined in the section), it shall within ninety days after ascertaining that it has such conflicting interest, either eliminate such conflicting interest or resign. Subsection (1) of this section provides, with certain exceptions stated therein, that a trustee under a qualified indenture shall be deemed to have a conflicting interest if such trustee is trustee under another indenture under which any other securities, or certificates of interest or participation in any other securities of the Company are outstanding.

The present application filed pursuant to clause (ii) of Section 310(b)(1) of the Act (as set forth in Section 608 of the 1970 Indenture), seeks to exclude the New Indenture from the operation of Section 310(b)(1) of the Act.

The effect of the proviso contained in clause (ii) of Section 310(b)(1) of the Act on the matter of the present application is such that the New Indenture may be excluded from the operation of Section 310(b)(1) of the Act (as set forth in Section 608 of the 1970 Indenture) if the Company shall have sustained the burden of proving by this application to the Commission and after opportunity for hearing thereon that the trusteeship of Chase under the 1970 Indenture and under the New Indenture is not so likely to involve a material conflict of interest as to make it necessary in the public interest or for the protection of investors to disqualify Chase from acting as trustee under one of these Indentures.

The Company alleges that:

(1) At February 28, 1982, it had outstanding $26,767,000 principal amount of 8⅞% Sinking Fund Debentures due 1995 issued under the 1970 Indenture (the "1970 Debentures"). The Debentures issued pursuant to the 1970 Indenture were registered under the Securities Act of 1933 (Filed No. 2-38515) and the 1970 Indenture was qualified under the Trust Indenture Act of 1939;

(2) Cummins Oveseas Finance N.V. has issued and sold $50,000,000 principal amount of 15½% Guaranteed Notes due December 15, 1991 (the "New Notes") issued under the New Indenture. The New Notes are guaranteed by the Company pursuant to Section 304 of the New Indenture. Chase is trustee under the New Indenture. The New Notes issued pursuant to the New Indenture were not offered or sold directly or indirectly in the United States of America, its territories or possessions, or areas subject to its jurisdiction, or to nationals or residents thereof. The issuance of these New Notes is therefore exempt from the registration requirements of the Securities Act of 1933 and the New Indenture is exempt from the qualification provisions of the Trust Indenture Act of 1939;

(3) The 1970 Indenture and the New Indenture are wholly unsecured and rank pari passu inter se;

(4) The Company's guarantee of the New Notes is neither superior nor inferior in right of payment to the 1970 Debentures;

(5) It is not in default under the 1970 Indenture;

(6) Such differences as exist between the 1970 Indenture and the New Indenture are not likely to involve a material conflict or interest so as to make it necessary in the public interest or for the protection of any of the Debentureholders to disqualify Chase from acting as Trustee under the 1970 Indenture.

The Company has waived notice of hearing, hearing and any and all rights to specify procedures under the Rules of Practice of the Securities and Exchange Commission in connection with this matter.

For a more detailed statement of the matters of fact and law asserted, all persons are referred to said application which is on file in the offices of the Commission's public Reference Section, 1100 L Street, NW., Washington, D.C. 20549.

Notice is further given that an order granting the application may be issued by the Commission at any time on or after May 17, 1982, unless prior thereto a hearing upon the application is ordered by the Commission, as provided in clause (ii) of Section 310(b)(1) of the Trust Indenture Act of 1939. Any interested person may, not later than May 17, 1982 at 5:30 p.m., Eastern Standard Time, in writing, submit to the Commission, his views or any additional facts bearing upon this application or the desirability of a hearing thereon. Any such communiction or request should be addressed: Secretary, Securities and Exchange Commission, 500 North Capitol Street, NW., Washington, D.C. 20549, and should state briefly the nature of the interest of the person submitting such information or requesting a hearing, the reasons for such request, and the issues of fact and law raised by the application which he desires to controvert.

For the Commission, by the Division of Corporation Finance, pursuant to delegated authority.

George A. Fitzsimmons,

Secretary.

[FR Doc. 82-10911 Filed 4-20-82; 8:45 am]

BILLING CODE 8010-01-M

[Rel. No. 12380; 812-4874]

Prudential Insurance Co. of America; Filing of Application

April 15, 1982.

Notice is hereby given that The Prudential Insurance Company of America ("Prudential"), 71 Hanover Road, Florham Park, N.J. 07932 filed an application on May 8, 1981, and amendments thereto on September 8, 1981 and March 25, 1982 for an order of the Commission pursuant to Sections 6(c) and 6(e) of the Investment Company Act of 1940 ("Act") exempting Prudential from the provisions of Sections 7 and 8 of the Act in connection with its operation of The Prudential Variable Contract Account GA-583 ("VCA-GA-583"). All interested persons are referred to the application on file with the Commission for a statement of the representations made therein, which are summarized below.

Prudential is a mutual life insurance company organized under the laws of New Jersey. It is registered as an investment adviser under the Investment Advisers Act of 1940 and as a broker-dealer under the Securities Exchange Act of 1934. The application states that Prudential maintains several separate accounts, including one designated as the Investment Fund of Prudential's Variable Contract Account ("VCA-IF"). According to the application, VCA-IF was established to receive and invest contributions under group annuity contracts issued to employers and trustees in connection with pension and profit-sharing plans meeting the requirements for qualification under Section 401 of the Internal Revenue Code of 1954, as amended ("Code"). The application states that VCA-IF has not registered under the Act in reliance on Section 3(c)(11).

A pension plan established by Yeshiva University ("Yeshiva") is

among those plans the contributions for which have been invested in VCA-IF. Prudential states that as of December 31, 1980, approximately $36.8 million of the assets of VCA-IF, which on that date aggregated $1,984 million, were attributable to contributions received under the Yeshiva plan. According to the application, Prudential discovered that the Yeshiva plan may not meet the requirements of Section 401 of the Code. Thus, if assets attributable to contributions under the plan continued to remain in VCA-IF, that separate account would not be able to rely on the exclusion from the definition of investment company provided in Section 3(c)(11) of the Act, and would have to register under the Act.

Prudential states that with the trustees of the Yeshiva plan, it is revising the plan to provide that assets attributable to contributions made under the Yeshiva plan will be transferred to The Prudential Variable Contract Account-2 ("VCA-2"), a separate account which is registered under the Act, and that future contributions under the Yeshiva plan will also be allocated to VCA-2. Prudential asserts that because of the need to obtain certain regulatory approvals and the consent of participants in the Yeshiva plan, it is not possible to effect a transfer of the assets attributable to contributions made under the Yeshiva plan into VCA-2 at this time. Therefore, Prudential states, it has established a new separate account, VCA-GA-583, and transferred those assets, effective April 30, 1981, to that new separate account as an interim measure. Prudential states that VCA-GA-583 will hold only those assets attributable to the Yeshiva plan.

Prudential seeks exemptive relief from Sections 7 and 8 of the Act in connection with its operation of VCA-GA-583. Section 7, in part, prohibits certain operations in interstate commerce by or on behalf of any investment company not registered under the Act. Section 8, in part, prescribes the procedures for registration under the Act. Prudential submits that the granting of exemptive relief from the provisions of these sections is necessary and appropriate in the public interest, consistent with the protection of investors and the purposes fairly intended by the policy and provisions of the Act. Prudential asserts that because the transfer of those assets held in VCA-GA-583 to VCA-2 is expected to be accomplished by October 30, 1982, and because VCA-GA-583, if it continues to exist at all after that time, will most likely not be deemed to be an investment company within the meaning of the Act by virtue of Section 3(c)(1) of the Act and thus will not be required to be registered under the Act, requiring VCA-GA-583 to register in the interim period would not serve any significant regulatory purpose, but rather would cause needless expense and possible delay of the ultimate transfer of the assets to VCA-2.

The application states that the Yeshiva plan is subject to the provisions of the Employee Retirement Income Security Act of 1974, as amended ("ERISA"), and, further, that Prudential will be a fiduciary with respect to the Yeshiva plan and will be subject, in managing separate account VCA-GA-583, to several provisions of ERISA that prohibit certain transactions with "parties in interest" to the plan and which include restraints upon self-dealing and transactions with affiliated persons that are more comprehensive than those set forth in Section 17 of the Act.

Prudential asserts that its contract with Yeshiva, as originally issued, provides that participants in the Yeshiva plan will be credited with annuity units, the value of which will vary with the investment results of VCA-IF, and that monthly payments, when made, will reflect the then current values of the annuity units the value of which varies with the investment results of VCA-IF. Prudential states that despite the transfer of the assets of the Yeshiva plan out of VCA-IF, it will remain obligated to comply with that contractual provision, and intends to do so. Prudential states that, at the time of the transfer of the assets of VCA-GA-583 to VCA-2, Yeshiva plan participants will be paid or credited with the higher of either the annuity unit values based on the assets of VCA-IF or the annuity unit values based on the assets of VCA-GA-583. Prudential makes an additional undertaking to persons currently receiving annuity payments under the Yeshiva plan that the total payments received by them through December 31, 1984, will reflect the better investment performance of VCA-IF or VCA-2.

Prudential is willing to consent to the following conditions to any order granting the requested exemptions:

(a) That VCA-GA-583 will comply with the record-keeping requirements of Section 31 of the Act to the same extent as if it were a registered investment company;

(b) That, in the period prior to the transfer of Yeshiva plan assets to VCA-2, Prudential will compute annuity unit values and make annuity payments in the manner described above;

(c) That Prudential will follow the investment policies of VCA-IF in investing the assets of VCA-GA-583;

(d) That Prudential will not send to participants in the Yeshiva plan any notices concerning the transfer of the plan's assets to VCA-2 until after the Commission has granted the application;

(e) That Prudential will provide to the Commission's staff for review copies of any notices concerning the transfer before they are sent to participants in the Yeshiva plan; and

(f) That the requested exemptions will be for a period of no longer than 18 months from April 30, 1981.

Section 6(c) of the Act provides, in part, that the Commission by order upon application may contionally or unconditionally exempt any person or transaction from any provision or provisions of the Act if and to the extent that such exemption is necessary or appropriate in this public interest and consistent with the protection of investors and the purposes fairly intended by the policy and provisions of the Act.

Section 6(e) of the Act provides that if, in connection with any rule, regulation, or order under Section 6 exempting any investment company from any provision of Section 7, the Commission deems it necessary or appropriate in the public interest or for the protection of investors that certain specified provisions of this Act pertaining to registered investment companies shall be applicable in respect of such company, the provisions so specified shall apply to such company, and to other persons in their transactions and relations with such company, as though such company were a registered investment company.

Notice is further given that any interested person may, not later than May 10, 1982, at 5:30 p.m., submit to the Commission, in writing, a request for a hearing on the application accompanied by a statement as to the nature of his interest, the reasons for such request and the issues, if any, of fact or law proposed to be controverted, or he may request that he be notified if the Commission shall order a hearing thereon. Any such communication should be addressed: Secretary, Securities and Exchange Commission, Washington, D.C. 20549. A copy of such request shall be served personally or by mail upon Prudential at the address stated above. Proof of such service (by affidavit or, in the case of an attorney-at-law, by certificate) shall be filed contemporaneously with the request. As provided by Rule 0-5 of the Rules and Regulations promulgated under the Act, an order disposing of the application

herein will be issued as of course following said date unless the Commission thereafter orders a hearing upon request or upon the Commission's own motion. Persons who request a hearing, or advice as to whether a hearing is ordered, will receive any notices and orders issued in this matter, including the date of the hearing (if ordered) and any postponements thereof.

By the Commission.

George A. Fitzsimmons,

Secretary.

[FR Doc. 82-10912 Filed 4-20-82; 8:45 am]

BILLING CODE 8010-01-M

[Rel. No. 18651; File No. SR-BSE-82-4]

Boston Stock Exchange, Inc.; Filing and Immediate Effectiveness of Proposed Rule Change

April 15, 1982.

Pursuant to Section 19(b)(1) of the Securities Exchange Act of 1934 (the "Act"), 15 U.S.C. 78s(b)(1), notice is hereby given that on April 1, 1982, the Boston Stock Exchange, Inc. ("BSE") filed with the Securities and Exchange Commission the proposed rule change as described herein. The Commission is publishing this notice to solicit comments on the proposed rule change from interested persons.

The BSE proposes to extend through June 30, 1982, its temporary 15% increase on all BSE billings to its members. The 15% temporary increase was instituted on May 5, 1981 and has previously been twice extended. Without the present (third) extension, the 15% temporary increase would have expired on March 31, 1982. The BSE's stated purpose for this proposal is to generate additional revenues to cover its operating costs. The BSE's stated authority for the proposal is Section 6(b)(4) of the Act.

The foregoing change has become effective, pursuant to Section 19(b)(3)(A) of the Act and subparagraph (e) of Rule 19b-4 under the Act. At any time within 60 days of the filing of such proposed rule change, the Commission may summarily abrogate such rule change if it appears to the Commission that such action is necessary or appropriate in the public interest, from the protection of investors, or otherwise in furtherance of the purposes of the Act.

Interested persons are invited to submit written data, views and arguments concerning the submission on or before May 12, 1982. Persons desiring to make written comments should file six copies thereof with the Secretary of the Commission, Securities and Exchange Commission, 500 North Capitol Street, Washington, D.C. 20549. Reference should be made to File No. SR-BSE-82-4.

Copies of the submission, all subsequent amendments, all written statements with respect to the proposed rule change which are filed with the Commission, and all written communications relating to the proposed rule change between the Commission and any person, other than those which may be withheld from the public in accordance with the provisions of 5 U.S.C. 552, will be available for inspection and copying at the Commission's Public Reference Room 1100 L Street, NW., Washington, D.C. Copies of the filing and of any subsequent amendments also will be available for inspection and copying at the principal office of the above-mentioned self-regulatory organization.

For the Commission, by the Division of Market Regulation pursuant to delegated authority.

George A. Fitzsimmons,

Secretary.

[FR Doc. 82-10913 Filed 4-20-82 8:45 am]

BILLING CODE 8010-01-M

[Rel. No. 18657; File No. SR-PSE-82-6]

Pacific Stock Exchange, Inc.; Filing of Proposed Rule Change

April 15, 1982.

Pursuant to Section 19(b)(1) of the Securities Exchange Act of 1934 (the "Act"), 15 U.S.C. 78s(b)(1), notice is hereby given that on April 12, 1982, the Pacific Stock Exchange, Inc. ("PSE") filed with the Securities and Exchange Commission is publishing this notice to solicit comments on the proposed rule change from interested persons.

The proposed rule change would amend present PSE Rule 10(f) to provide that the PSE has elected, pursuant to Rule 11Ac1-1(b)(5)(i) of the Act, to collect, process and make available bids, offers and quotation sizes with respect to any PSE-traded security which is listed on the New York Stock Exchange, on the American Stock Exchange ("Amex"), or on any regional exchange which substantially meets Amex listing criteria. The PSE's stated purpose for this proposal is to foster cooperation with persons engaged in securities information processing. The PSE states that the proposal is consistent with Section 6(b) of the Act and, in particular, with Section 6(b)(5) of the Act.

In order to assist the Commission in determining whether to approve the proposed rule change or institute proceedings to determine whether the proposed rule change should be disapproved, interested persons are invited to submit written data, views and arguments concerning the submission on or before May 12, 1982. Persons desiring to make written comments should file six copies thereof with the Secretary of the Commission, Securities and Exchange Commission, 500 North Capitol Street, Washington, D.C. 20549. Reference should be made to File No. SR-PSE-82-6.

Copies of the submission, all subsequent amendments, all written statements with respect to the proposed rule change which are filed with the Commission, and all written communications relating to the proposed rule change between the Commission and any person, other than those which may be withheld from the public in accordance with the provisions of 5 U.S.C. 552, will be available for inspection and copying at the Commission's Public Reference Room, 1100 L Street, NW., Washington, D.C. Copies of the filing and of any subsequent amendments also will be available for inspection and copying at the principal office of the above-mentioned self-regulatory organization.

For the Commission, by the Division of Market Regulation pursuant to delegated authority.

George A. Fitzsimmons,

Secretary.

[FR Doc. 82-10914 Filed 4-20-82; 8:45 am]

BILLING CODE 8010-01-M

[Rel. No. 18658; File No. SR-PSE-82-5]

The Pacific Stock Exchange, Inc.; Filing of Proposed Rule Change

April 15, 1982.

Pursuant to Section 19(b)(1) of the Securities Exchange Act of 1934 (the "Act"), 15 U.S.C. 78s(b)(1), notice is hereby given that on April 5, 1982, the Pacific Stock Exchange Inc. ("PSE") filed with the Securities and Exchange Commission the proposed rule change as described herein. The Commission is publishing this notice to solicit comments on the proposed rule change from interested persons.

The PSE proposes to increase its listing fees for the listing of additional shares or warrants. Under the proposed amendment, the PSE would charge a fee of ½ cent per share subject to a $500 minimum fee per listing, and a $5,000 maximum fee per listing. Prior to the proposed increase, the PSE has charged a fee of ½ cent per share, subject to a minimum fee of $500 for listing 100,000 shares or less, and a maximum fee of

$2,500 for listing 500,000 shares or more, with a minimum fee of $500 per application, and a maximum fee of $2,500 per application.

In its filing with the Commission, the PSE has stated that the proposed change in listing fees is intended to generate additional revenues to meet rising expenses, and has indicated that the statutory basis for the proposed change is section 6(b)(4) of the Act, which provides for the equitable allocation of reasonable dues, fees, and other charges among its members and issuers and other persons using its facilities.

In order to assist the Commission in determining whether to approve the proposed rule change or institute proceedings to determine whether the proposed rule change should be disapproved, interested persons are invited to submit written data, views and arguments concerning the submission on or before May 12, 1982. Persons desiring to make written comments should file six copies thereof with the Secretary of the Commission, Securities and Exchange Commission, 500 North Capitol Street, Washington, D.C. 20549. Reference should be made to File No. SR-PSE-82-5.

Copies of the submission, all subsequent amendments, all written statements with respect to the proposed rule change which are filed with the Commission, and all written communications relating to the proposed rule change between the Commission and any person, other than those which may be withheld from the public in accordance with the provisions of 5 U.S.C. 552, will be available for inspection and copying at the Commission's Public Reference Room, 1100 L Street, NW, Washington, D.C. Copies of the filing and of any subsequent amendments also will be available for inspection and copying at the principal office of the above-mentioned self-regulatory organization.

For the Commission, by the Division of Market Regulation pursuant to delegated authority.

George A. Fitzsimmons,

Secretary.

[FR Doc. 82-10915 Filed 4-20-82; 8:45 am]

BILLING CODE 8010-01-M

DEPARTMENT OF THE TREASURY

Internal Revenue Service

Commissioner's Advisory Group; Open Meeting

There will be a meeting of the Commissioner's Advisory Group on May 12 and 13, 1982. The meeting will be held in Room 3313 of the Internal Revenue Service Building. The building is located at 1111 Constitution Avenue NW., Washington, D.C. The meeting will begin at 9:30 a.m. on Wednesday, May 12, and at 8:30 a.m. on Thursday, May 13. The agenda will include the following topics:

Wednesday, May 12, 1982

Status of Taxpayer Service Program
Criminal Investigation Programs
Update on Automated Collection System
Private Letter Rulings Program

Thursday, May 13, 1982

Paperwork Reduction Act—Requirements for Pre-clearance with OMB
Abusive Tax Shelter Program
Individual Retirement Accounts
Problems with Pro Se Taxpayer

The meeting, which will be open to the public, will be in a room that accommodates approximately 50 people. After the Committee members finish discusssing the items on the agenda, there may be time for statements by non-members. If you want to make a statement at the meeting, or if you would like the Committee to consider a written statement, please call or write to John E. Burke, Acting Assistant to the Deputy Commissioner, 1111 Constitution Ave. NW., Washington, D.C. 20224.

For further information contact: John E. Burke, Acting Assistant to the Deputy Commissioner, 202-566-4143 (not toll free).

Roscoe L. Egger, Jr.,

Commissioner.

[FR Doc. 82-10886 Filed 4-20-82; 8:45 am]

BILLING CODE 4830-01-M

[Delegation Order No. 190]

Delegation of Authority

AGENCY: Internal Revenue Service, Treasury.

ACTION: Delegation of authority.

SUMMARY: This Order effects the transfer of the Corporation Tax and Individual Tax Divisions and the Appeals function to the Office of Chief Counsel as prescribed by the Department of the Treasury Order Number 150-95. The text of the Delegation Order appears below.

EFFECTIVE DATE: March 21, 1982.

FOR FURTHER INFORMATION CONTACT: Martha M. Seeman, PM:S:FM:IMD, 1111 Constitution Avenue, N.W., Room 3526, Washington, D.C. 20224, Telephone number 202-566-4273, (Not a Toll-Free telephone number).

This document does not meet the criteria for significant regulations set forth in paragraph 8 of the Treasury directive appearing in the **Federal Register** for Wednesday, November 8, 1978.

Martha M. Seeman,

Acting Chief, Internal Management Documents Branch.

Delegation Order; Transfer of Technical and Appeals Functions to the Office of Chief Counsel.

Date of issue: March 18, 1982.

Pursuant to the authority granted to the Commissioner of Internal Revenue by 26 CFR 1.9100-1, 301.6020-1, 301.6201-1, 301.7701-9, the Int. Rev. Code of 1954, and Treasury Department Orders 150-32, 150-36, 150-37, 150-83, 177-19, and paragraphs 8 and 9 of Treasury Department Order 150-95, paragraph 6 of Treasury Department Order 150-95 is effective, as of the effective date of this Order. The Chief Counsel, or his/her delegate is hereby authorized to perform (1) all functions performed by the Corporation Tax, and Individual Tax Divisions, and (2) the National Office and field functions of Appeals, which were performed prior to the effective date of this Order except as provided below. The functions delegated include, but are not limited to:

1. Exclusive jurisdiction, including engineering and valuation activities, to issue letter rulings (with respect to prospective transactions or completed transactions if the request was made before any affected returns have been filed) and to issue technical advice memoranda; to propose revenue rulings and revenue procedures; to grant a reasonable extension of time fixed by regulations for making an election or application for relief in respect of tax under Subtitle A of the Code, subject to the requirements of 26 CFR 1.9100-1; to prescribe the extent, if any, to which any ruling issued by or pursuant to authorization from the Chief Counsel relating to the Internal Revenue laws shall be applied without retroactive effect; and interpret the tax treaties for the official from time to time designated as the "competent or taxation authority" under tax treaties of the United States;

(a) The authority delegated to the Assistant Commissioner (Technical) in Delegation Order No. 165 (Rev. 3) to respond to appeals filed pursuant to the Freedom of Information Act, 5 U.S.C. 552 (FOIA), is not transferred to the Chief Counsel.

2. The authority to enter into and approve a written agreement with any person relating to the Internal Revenue tax liability of such person (or with the person or estate for whom he/she acts) in cases under his/her jurisdiction, but not to set aside any closing agreement;

3. The authority to represent the Commissioner in determining liability, qualification, exempt status, or foundation qualification for the following types of cases not docketed in the United States Tax Court where the taxpayer does not agree with the determination made by the District Director, Service Center Director, or by the Director of International Operations and such taxpayer requests reconsideration by the Regional Director of Appeals:

(a) Except as qualified below, cases and/or work units determining liability for excise, employment, income, estate (including extensions for paying estate tax under internal Revenue Code Section 6161(a)(2) and determinations whether elections made by estates meet the conditions specified in Internal Revenue Code Sections 6166 and 6166A) and cases and/or work units determining liability for gift taxes including additions to tax, additional amounts and assessable penalties under Chapter 68 of Subtitle F of the Internal Revenue Code of 1954 or corresponding provisions of the Internal Revenue Code of 1939;

(b) Responsibility for the administrative system of tax appeals for cases involving offers-in-compromise, penalty appeals, recommendations concerning settlement offers in refund suits, Code section 534(b) letters, refund claims including Joint Committee cases, and overassessments in which a taxpayer appeals the decision of a District Director, a Service Center director or the Director of International Operations.

(c) Initial or continuing qualification under Subchapter D of Chapter 1 of the Internal Revenue Code 1954 and initial or continuing exempt status and foundation classification *except* when a National Office ruling on the case with respect to exempt status or foundation classification, or National Office technical advice, with respect to qualification, exempt status or foundation classification, has been issued. In certain instances such as cases arising from the Examination function or cases in which a National Office technical advice covers only a portion of the qualification issue of an employee plan, Appeals officials will have jurisdiction over the *proposed action* where a National Office ruling on the case with respect to exempt status or foundation classification or a National Office technical advice, with respect to qualification, exempt status or foundation classification has been issued. If the Appeals proposed disposition is contrary to the National Office ruling on the case with respect to exempt status or foundation classification or the National Office technical advice with respect to qualification, exempt status or foundation classification, the Assistant Commissioner (Employee Plans and Exempt Organizations) or the delegate of the Associate Chief Counsel (Technical) in Code section 521 cases, will make the final decision.

(d) The excise and employment taxes subject to the provisions of this order include any Federal excise or employment tax under the Internal Revenue Code of 1954, except any tax imposed by the following provisions or corresponding provisions of the Internal Revenue Code of 1939:

(i) Subtitle E; or

(ii) Subchapter D, Chapter 78 of Subtitle F, insofar as it relates to taxes imposed under Subtitle E.

4. To utilize Appeals personnel to settle in whole or in part cases docketed in the Tax Court for such periods of exclusive jurisdiction as he/she shall deem appropriate. For purposes of any statutory provisions which may require that a taxpayer exhaust the administrative remedies available within the Internal Revenue Service, all Appeals functions pertaining thereto are considered to be within the Internal Revenue Service notwithstanding any other provision in this Order.

5. To supervise and evaluate the work of all officers and employees of the functions transferred including but not limited to the (i) Appeals functions, and (ii) the Income Tax Division and Corporate Tax Division (formerly under the supervision of the Assistant Commissioner (Technical) and (iii) to take necessary action in all personnel matters pertaining thereto, including those for the appointment, classification, promotion, demotion, reassignment, transfer or separation of such officers and employees, with the exception of promotions, demotions, or separations to or from the Senior Executive Service.

6. In exercising the authority granted in paragraph 5 above with respect to the Appeals function, the Chief Counsel shall utilize the Internal Revenue Service Executive Resources Board for selection of SES and Resource Board covered positions. With regard to the selection of non E.R.B. Appeals personnel a majority of each selection panel will be Appeals personnel. Notwithstanding any other grant of authority in this Order, the Commissioner retains the final authority with regard to all personnel, policies, practices, and matters affecting working conditions as well as all conditions of employment. The appointment of any person as an attorney in the Appeals function or transfer of any person in the Appeals function from non-attorney status to attorney status shall require the Commissioner's approval or the approval of the Deputy Commissioner.

7. The following Commissioner's Delegation Orders are continued in effect, together with any other Delegation Orders where authority has been delegated to Appeals and Technical personnel to the extent transferred by this Order and T.D.O. 150-95. These orders are subject to the provision that the Chief Counsel or his/her delegate may revoke any portion of such orders which pertain to officers and personnel under his/her supervision and redelegate such authority to other officers and employees under his/her supervision provided such authority may be revoked or redelegated: 8 (Rev. 7), 11 (Rev. 12), 25 (Rev. 11), 35 (Rev. 11), 42 (Rev. 15), 77 (Rev. 14), 93 (Rev. 7), 107 (Rev. 5), 109 (Rev. 5), 112 (Rev. 6), 113 (Rev. 6), 136 (Rev. 3), 139 (Rev. 5), 160 (Rev. 2), 171 and 179.

8. The following Commissioner's Delegation orders are supplemented and amended consistent with the provisions of this Order:

(a) All of Orders Nos. 60 (Rev. 5) and 66 (Rev. 10) and

(b) Paragraphs 1, 4 and 5 of Order No. 97 (Rev. 18).

9. Commissioner's Delegation Orders Nos. 96 (Rev. 5), 114 (Rev. 2), and 183 are amended by substituting the words "Associate Chief Counsel (Technical)" for the words "Assistant Commissioner (Technical)" wherever they appear in those orders.

10. With regard to paragraph 3(b) above: Delegation Orders No. 14 (Rev. 1) 154 and 155 are appropriately conformed.

11. Delegation Order Nos. 39 (Rev. 11) and 89 (Rev. 4) are supplemented by adding the words "Chief Counsel" and "Regional Counsel."

12. The authority delegated in Delegation Order No. 9 (Rev. 6) regarding use of Government automobiles is also delegated to the Chief Counsel and to his/her delegate and to the Regional Counsel.

13. Delegation Order No. 153, Nationwide Authority to Make Determinations on Certain Oil Related Issues, is not affected by this Order.

14. As of the effective date of this Order, all personnel performing any previously delegated function prior to the effective date of this Order are hereby authorized to continue to perform such function to the extent not inconsistent with this Order. However,

the Chief Counsel is authorized to take appropriate action to further the orderly and expeditious transfers and excercise of functions described in this Order.

Roscoe Egger,

Commissioner.

[FR Doc. 82-10887 Filed 4-20-82; 8:45 am]

BILLING CODE 4830-01-M

VETERANS ADMINISTRATION

Central Laundry, Veterans Administration Medical Center, Pittsburgh, PA; Finding of No Significant Impact

The Veterans Administration has assessed the potential environmental impact that may occur as a result of the construction of a new laundry at the Veterans Administration Medical Center (VAMC), Highland Drive, Pittsburgh, Pennsylvania.

The project proposes to construct a new laundry building of approximately 27,000 net square feet to handle all the laundry for Highland Drive and both the Pittsburgh and Aspinwall Divisions of the VAMC, University Drive. The proposed site is in the southeast portion of the station, inside the loop road, east of building 8 (Recreation). This poroject is estimated at approximately 10 million dollars, inclusive of the laundry equipment costs, contingencies, professional design services and inflation to the time of construction. Alternatives included combining the laundry with a new warehouse in the same building at Highland Drive. This involved consideration of an alternate site. Consideration was also given to locating the laundry, combined with a new warehouse, at the Aspinwall Division, across the river. The No Action alternative was also considered.

Development of the project will have minor impacts on the human and natural environment from construction noise, dust, fumes and visual impacts during the contruction period. There will be an increase in area water usage, and waste water delivered into the sanitary sewer. The amount of city water consumed and delivered to the treatment plant should not change as this new facility replaces other small laundries throughout Pittsburgh.

Mitigation of the project's impact on the environment includes appropriate erosion, noise, dust and fume control during construction as delineated in Veterans Administration Standard Specifications, Environmental Protection Section, and building design to reduce operational noise to the rest of the station.

The significance of the identified impacts has been evaluated relative to the considerations of both context and intensity, as defined by the Council on Environmental Quality (Title 40 CFR 1508.27).

This Enviornmental Assessment has been performed in accordance with the requirements of the National Environmental Policy Act Regulations, Sections 1501.3 and 1508.9. A "Finding of No Significant Impact" has been reached based on the information presented in this assessment.

The assessment is being placed for public examination at the Veterans Administration, Washington, D.C. Persons wishing to examine a copy of the document may do so at the following office: Mr. Willard Sitler, P.E., Director, Environmental Affairs Staff (005B), Room A14, Veterans Administration, 810 Vermont Avenue NW., Washington, D.C., (202-389-2526). Questions or requests for single copies of the Environmental Assessment may be addressed to: Director, Environmental Affairs Staff (005B), 810 Vermont Avenue, NW., Washington, D.C. 20420.

Dated: April 14, 1982.

Robert P. Nimmo,

Administrator.

[FR Doc. 82-10834 Filed 4-20-82; 8:45 am]

BILLING CODE 8320-01-M

Sunshine Act Meetings

Federal Register
Vol. 47, No. 77
Wednesday, April 21, 1982

This section of the FEDERAL REGISTER contains notices of meetings published under the "Government in the Sunshine Act" (Pub. L. 94-409) 5 U.S.C. 552b(e)(3).

CONTENTS

	Items
Commodity Futures Trading Commission	1, 2
Federal Deposit Insurance Corporation	3, 4, 5
Federal Home Loan Mortgage Corporation	6

1

COMMODITY FUTURES TRADING COMMISSION.

TIME AND DATE: 2:00 p.m., Friday, April 30, 1982.

PLACE: 2033 K Street, NW., Washington, D.C. fifth floor hearing room.

STATUS: Open.

MATTERS TO BE CONSIDERED: Oral Argument Before the Commission in the Mater of Indiana Farm Bureau Cooperative Association, Inc., and Louis M. Johnston—CFTC Docket No. 75-14.

CONTACT PERSON FOR MORE INFORMATION: Jane Stuckey, 254-6314.

[S-579-82 Filed 4-19-82; 12:00 pm]

BILLING CODE 6351-01-M

2

COMMODITY FUTURES TRADING COMMISSION.

TIME AND DATE: 5:45 p.m., Monday, April 19, 1982.

PLACE: 2033 K Street, N.W., Washington, D.C., eighth floor conference room.

STATUS: Closed.

MATTERS TO BE CONSIDERED: Reauthorization.

CONTACT PERSON FOR MORE INFORMATION: Jane Stuckey, 254-6314.

[S-580-82 Filed 4-19-82; 12:00 pm]

BILLING CODE 6351-01-M

3

FEDERAL DEPOSIT INSURANCE CORPORATION

NOTICE OF AGENCY MEETING.

Pursuant to the provisions of the "Government in the Sunshine Act" (5 U.S.C. 552b), notice is hereby given that at 7:00 p.m. on Friday, April 16, 1982, the Board of Directors of the Federal Deposit Insurance Corporation met in closed session, by telephone conference call, to (1) accept the bid of Bank of Tyler, N.A., Tyler, Texas, a newly chartered national bank subsidiary of First Bancshares of Texas, Inc., for the pruchase of certain assets of and the assumption of the liability to pay deposits made in National Security Bank, Tyler, Texas, which was closed by the Acting Comptroller of the Currency at 6:30 p.m. (EST) on Friday, April 16, 1982; and (2) provide such financial assistance, pursuant to section 13(e) of the Federal Deposit Insurance Act (12 U.S.C. 1823(e)), as was necessary to effect the purchase and assumption transaction.

In calling the meeting, the Board determined, on motion of Chairman William M. Isaac, seconded by Director Irvine H. Sprague (Appointive), concurred in by Mr. H. Joe Selby, acting in the place and stead of Director C. T. Conover (Comptroller of the Currency), that Corporation business required its consideration of the matters on less than seven days' notice to the public; that no earlier notice of the meeting was practicable; that the public interest did not require consideration of the matters in a meeting open to public observation; and that the matters could be considered in a closed meeting pursuant to subsections (c)(8), (c)(9)(A)(ii), and (c)(9)(B) of the "Government in the Sunshine Act" (5 U.S.C. 552b(c)(8), (c)(9)(A)(ii), and (c)(9)(B)).

Dated: April 19, 1982.

Federal Deposit Insurance Corporation.

Alan J. Kaplan,

Deputy Executive Secretary.

[S-582-82 Filed 4-19-82; 3:18 pm]

BILLING CODE 6714-01-M

4

FEDERAL DEPOSIT INSURANCE CORPORATION.

Notice of Agency Meeting.

Pursuant to the provisions of the "Government in the Sunshine Act" (5 U.S.C. 552b), notice is hereby given that the Federal Deposit Insurance Corporation's Board of Director will meet in open session at 2:00 p.m. on Monday April 26, 1982, to consider the following matters:

Summary Agenda: No substantive discussion of the following items is anticipated. These matters will be resolved with a single vote unless a member of the Board of Directors requests that an item be moved to the discussion agenda.

Disposition of minutes of previous meetings.

Application of consent to merge and establish seven branches:

The First Bank of Whiting, Whiting, Indiana, for consent to merge under its charter and title, with the First National Bank of Crown Point, Crown Point, Indiana, and South Lake National Bank, Lowell, Indiana, and to establish the six offices of The First National Bank of Crown Point and the sole office of South Lake National Bank as branches of resultant the bank.

Application for consent to acquire assets and assume liabilities and establish one branch:

Commonwealth Bank, Hawthorne, California, for consent to purchase the assets of an assume the liability to pay deposits made in the Equality Savings and Loan Association, San Diego, California, and for consent to establish the sole office of Equality Savings and Loan Association as a branch of the resultant bank.

Recommendations regarding the liquidation of bank's assets acquired by the Corporation in its capacity as receiver, liquidator, or liquidating agent thereof:

Case No. 44,937-SR (Amended)—American Bank & Trust Company New York, New York
Case No. 45,174-SR—Bank of Lake Helen Lake Helen, Florida
Case No. 45,180—The Greenwich Savings Bank New York, New York
Case No. 45,182-L—Franklin National Bank New York, New York

Recommendations with respect to payment for legal services rendered and expenses incurred in connection with receivership and liquidation activities:

Bronson, Bronson & McKinnon, San Francisco, California, in connection with the receivership of United States National Bank, San Diego, California.
Feldstein, Gelpi & Hernandez, Old San Juan, Puerto Rico, in connection with the liquidation of Banco Credito y Ahorro Ponceno, Ponce, Puerto Rico.
Colorado, Martinez, Odell, Calabria & Sierra, Hato Rey, Puerto Rico, in connection with the liquidation of Banco Credito y Ahorro Ponceno, Ponce, Puerto Rico.

Memorandum and Resolution re: Proposed amendment to Part 341 of the Corporation's rules and regulations, entitled "Registration of Securities

Transfer Agents," which would simplify the present registration form and add a section dealing with deregistration of transfer agents.

Memorandum and Resolution re: Semiannual agenda of regulations.

Memorandum and Resolution re: Proposed revisions of the Corporation's "Delegations of Authority Relating to the Staffing Table."

Reports of committees and officers:

Minutes of the actions approved by the standing committees of the Corporation pursuant to authority delegated by the Board by Directors.

Reports of the Division of Bank Supervision with respect to applications or requests approved by the Director or Associate Director of the Division and the various Regional Directors pursuant to authority delegated by the Board of Directors.

Discussion Agenda:

No matters scheduled.

The meeting will be held in the Board Room on the sixth floor of the FDIC Building located at 550-17th Street, NW., Washington, D.C.

Request for information concerning the meeting may be directed to Mr. Hoyle L. Robinson, Executive Secretary of the Corporation, at (202) 389-442.

Dated: April 19, 1982.

Federal Deposit Insurance Corporation.

Alan J. Kaplan,

Deputy Executive Secretary.

[S-583-82 Filed 4-19-82; 3:18 pm]

BILLING CODE 6714-01-M

5

FEDERAL DEPOSIT INSURANCE CORPORATION.

Notice of Agency Meeting.

Pursuant to the provisions of the "Government in the Sunshine Act" (5 U.S.C. 552b), notice is hereby given that at 2:30 p.m. on Monday, April 26, 1982, the Federal Deposit Insurance Corporation's Board of Directors will meet in closed session, by vote of the Board of Directors pursuant to sections 552b(c)(2), (c)(6), (c)(8), (c)(9)(A)(ii), (c)(9)(B), and (c)(10) of Title 5, United States Code, to consider the following matters:

Summary Agenda: No substantive discussion of the following items is anticipated. These matters will be resolved with a single vote unless a member of the Board of Directors requests that an item be moved to the discussion agenda.

Recommendations with request to the initiation, termination, or conduct of administrative enforcement proceedings (cease-and-desist proceedings, termination-of-insurance proceedings, suspension or removal proceedings, or assessment of civil money penalties) against certain insured banks or officers, directors, employees, agents, or other persons participating in the conduct of the affairs thereof:

Names of persons and names and locations of banks authorized to be exempt from disclosure pursuant to the provisions of subsections (c)(6), (c)(8), and (c)(9)(A)(ii) of the "Government in the Sunshine Act" (5 U.S.C. 552b(c)(6), (c)(8), and (c)(9)(A)(ii)).

NOTE.—Some matters falling within the category may be placed on the discussion agenda without further public notice if it becomes likely that substantive discussion of those matters will occur at the meeting.

Reports of committees and officers:

Report of the Director, Division of Liquidation:

Memorandum re: The Hamilton Bank and Trust Company, Atlanta, Georgia

Discussion Agenda:

Recommendations regarding the liquidation of a bank's assets acquired by the Corporation in its capacity as receiver, liquidator, or liquidating agent thereof:

Case No. 45,078-L—Banco Credito y Ahorro Ponceno, Ponce, Puerto Rico

Memorandum and Resolution re: First National Bank & Trust Company of Tuscola, Tuscola, Illinois

Personnel actions regarding appointments, promotions, administrative pay increases, reassignments, retirements, separations, removals, etc.:

Names of employees authorized to be exempt from disclosure pursuant to the provisions of subsection (c)(2) and (c)(6) of the "Government in the Sunshine Act" (5 U.S.C. 552b(c)(2) and (c)(6)).

The meeting will be held in the Board Room on the sixth floor of the FDIC Building located at 550 17th Street, NW., Washington, D.C.

Requests for information concerning the meeting may be directed to Mr. Hoyle L. Robinson, Executive Secretary of the Corporation, at (202) 389-4425.

Dated: April 19, 1982.

Federal Deposit Insurance Corporation.

Alan J. Kaplan,

Deputy Executive Secretary.

[S-584-82 Filed 4-19-82; 3:16 pm]

BILLING CODE 6714-01-M

6

FEDERAL HOME LOAN MORTGAGE CORPORATION.

DATE AND TIME: April 26, 1982, 10 a.m.

PLACE: 1776 G Street, N.W., Washington, D.C., 4th Floor, Conference Room 4-G.

STATUS: Open/Closed.

CONTACT PERSON FOR MORE INFORMATION: Scott R. Daugherty.

MATTERS TO BE CONSIDERED:

open: Private Mortgage Insurer's Eligibility Requirements

Closed:

Minutes of March 31, 1982 Board of Directors' Meeting
President's Report
Complete February Financial Statements
Partial March Financial Statements
Minute Entry
Minutes from March 31, 1982 Financing Strategy Meeting
Financial Strategy May 1982
Minute Entry
Hedging Contract Limit Resolution
Short-term Debt Resolution

April 19, 1982.

[S-581-82 Filed 4-19-82; 1:54 pm]

BILLING CODE 6720-02-M

federal register

Wednesday
April 21, 1982

Part II

Department of Health and Human Services

National Institutes of Health

Recombinant DNA Research; Actions Under Guidelines

DEPARTMENT OF HEALTH AND HUMAN SERVICES

National Institutes of Health

Recombinant DNA Research; Actions Under Guidelines

AGENCY: National Institutes of Health, Public Health Service, HHS.

ACTION: Notice of actions under NIH guidelines for research involving recombinant DNA molecules.

SUMMARY: This notice sets forth actions taken by the Acting Director, National Institute of Allergy and Infectious Diseases, by authority of the Director, National Institutes of Health, under the 1981 Guidelines for Research Invoving Recombinant DNA Molecules (46 FR 34462).

EFFECTIVE DATE: April 21, 1982.

FOR FURTHER INFORMATION CONTACT: Additional information can be obtained from Dr. William Jr. Gartland, Office of Recombinant DNA Activities (ORDA), National Institutes of Health, Bethesda, Maryland 20205 (301) 496-6051.

SUPPLEMENTARY INFORMATION: I am promulgating today a major action under the NIH Guidelines for Research Involving Recombinant DNA Molecules. This action involves a major revision of the Guidelines. In accordance with Section IV-E-1-b of the Guidelines, I find that this action complies with the Guidelines and present no significant risk to health or the environment.

The structure of this announcement is as follows:

I. Background
II. February 8-9, 1982, Meeting of Recombinant DNA Advisory Committee
III. Analysis of Correspondence Received
IV. Summary of Guideline Changes

Immediately following this announcement, there appears in a separate section of the **Federal Register** the revised NIH Guidelines for Research Involving Recombinant DNA Molecules, which are effective today.

I. Background

Drs. David Baltimore and Allan Campbell proposed a major revision of the Guidelines (Baltimore-Campbell proposal) which was published for public comment in the **Federal Register** on March 20, 1981 (46 FR 17995) and was considered by the NIH Recombinant DNA Advisory Committee (RAC) at its April 1981 meeting. At the April 1981 meeting, a Working Group on Revision of the Guidelines was established to review the Baltimore-Campbell proposal as well as other approaches which might lead to a major revision of the Guidelines. The Working Group met on June 1, 1981, and on July 9, 1981. The Working Group prepared a proposal for revising the Guidelines, a summary of its actions, and a document entitled "Evaluation of the Risks Associated with Recombinant DNA Research." Two minority reports were prepared by several members of the Working Group. The Working Group report and the minority reports were distributed to RAC members prior to the September 1981 meeting. The document entitled "Evaluation of the Risks Associated with Recombinant DNA Research" was an extensive analysis with the following headings:

I. History and Introduction
II. Possible Hazards
A. Basic Assumptions
1. Uniqueness of Organisms Created by Recombinant DNA Techniques
a. Limits of the technique
b. Natural exchange mechanisms
c. Counterarguments
2. Dissemination
a. Stability of recombinant DNA
b. Transmission into other potential hosts
3. Harm
a. Breaching prokaryotic—eukaryotic barriers: evolutionary considerations
b. Small pieces in large organisms
c. Specific cases
i. Expression of active peptides: hormones, toxins
ii. Expression of cross-reacting antibodies
iii. Animal virus cloning
B. Human Genetic Engineering
III. Costs
IV. Conclusions

A. Summary Analysis of Risks

B. Possible Responses
1. Maintain the Status Quo
2. Abolish the Guidelines
C. Recommendation

The RAC extensively discussed the Working Group's report and other approaches to revision of the Guidelines at its September 1981 meeting. The RAC passed by a vote of 16 in favor, 3 opposed, with 1 abstention, the elements of its version of a proposed revision of the Guidelines to be published for public comment. Based on these elements, NIH staff prepared the RAC version of proposed revised Guidelines (September 1981 RAC proposal) which was published for public comment in the **Federal Register** of December 4, 1981 (46 FR 59368). The relevant background documents were also published in the December 4, 1981, **Federal Register** announcement, as follow—Annex A: Original proposal of Drs. David Baltimore and Allan Campbell; Annex B: Documents prepared by Working Group on Revision of the Guidelines; Annex C: Minority reports of working group members; Annex D: Draft Minutes of relevant portion of September 10-11, 1981, RAC Meeting; and Annex E: Current NIH Guidelines. Comments were due by February 2, 1982.

The major features of the September 1981 RAC proposal were:

1. The Guidelines would cease to be mandatory and would become a voluntary code of standard practice. Requirements that institutions have an Institutional Biosafety Committee (IBC), that investigators obtain prior approval from the IBC before beginning certain experiments, that investigators obtain prior approval from NIH before beginning certain experiments, and the section of the Guidelines specifying that noncompliance with the Guidelines could lead to loss of NIH funds, would all be eliminated.

2. Section III of the Guidelines giving containment levels would be greatly simplified, and most experiments currently mandated at P2 and P3 containment would be recommended at P1.

3. The prohibitions section (I-D) of the Guidelines would be eliminated, although two of the previous prohibitions would be retained instead as admonishments.

Dr. Susan Gottesman of the National Cancer Institute of the National Institutes of Health prepared an alternative proposal (Gottesman proposal) for a major revision of the Guidelines.

The major features of this proposal were:

1. The Guidelines would continue to be mandatory for institutions receiving NIH funding. Certain experiments would continue to require prior review by NIH, certain experiments would continue to require prior review by an IBC, and certain experiments would require notice to an IBC simultaneously with initiation of the experiment.

2. Section III of the Guidelines would be reorganized and simplified. All experiments would fall into one of four classes. Physical containment requirements for some classes of experiments would be lowered.

3. Three of five prohibitions (I-D-2, I-D-4 and I-D-5 in the July 1981 Guidelines) would be listed in a new section that would continue to require RAC review and NIH approval before initiation. Experiments falling under prohibition I-D-1 and I-D-6 in the July 1981 Guidelines could proceed after IBC approval.

A summary of this (Gottesman) proposal and changes in the July 1981 Guidelines to implement the proposal were published for comment in the **Federal Register** of December 7, 1981 (46 FR 59734). Comments on this proposal were due by February 1, 1982.

ORDA and NIH staff prepared a document summarizing the two proposals (i.e., the September 1981 RAC proposal published for comment in the December 4, 1981, **Federal Register**, and the Gottesman proposal published for comment in the December 7, 1981, **Federal Register**) including a table comparing them with the July 1981 Guidelines. ORDA distributed the summary and copies of the **Federal Register** of December 4, 1981, and December 7, 1981, to over 4,300 individuals and organizations that had expressed interest in being informed about developments affecting the Guidelines and recombinant DNA research. The document invited comments on the two proposals. In addition, notices appeared in a number of periodicals inviting comments on the proposals.

II. February 8–9, 1982, Meeting of Recombinant DNA Advisory Committee

On February 8–9, 1982, the RAC reviewed the proposals for changing the Guidelines. They had been sent in advance: The December 4, 1981, **Federal Register** containing the September 1981 RAC proposal; the December 7, 1981, **Federal Register** containing the Gottesman proposal; the summary of the two proposals including the table comparing them with the July 1981 Guidelines; and all letters of comment on the proposals (a total of 86 letters) received by NIH by February 5, 1982. Part II–A of this announcement contains the draft minutes of the relevant portions of the February 8–9, 1982, RAC meeting. Part II–B of this announcement gives the response of the Acting Director, National Institute of Allergy and Infectious Diseases (NIAID), to the RAC recommendations.

II–A. Draft Minutes of Relevant Portions of February 8–9, 1982, RAC Meeting

Mr. Thornton called the attention of the RAC to the major topic of the February 8–9, 1982 meeting, a discussion of two proposals (tabs 1050, 1056A, 1056B, 1056C/1, 1056C/7, 1056D, 1056E, 1056F, 1056G) to modify the current NIH Guidelines for Research Involving Recombinant DNA molecules.

Mr. Thornton said he would take a moment to give his personal perspective prior to resuming the role as committee chairman. He said former NIH Director, Donald Fredrickson summarized the purposes of the Guidelines as (1) to establish a rapid, complete means of communication, (2) to assure that the Guidelines are conservative yet allow research to proceed, and (3) to permit public participation in the formulation of public policy. Mr. Thornton noted the difficulty of establishing and maintaining communication between public policy decision makers and experts in a scientific field. NIH has devised a mechanism which successfully maintains this communication, and he would not wish to abandon it.

Mr. Thornton then described the Guidelines from a lawyer's perspective. He noted that the Guidelines are not laws; he thought this is good since laws are difficult to formulate and difficult to change. Neither are they regulations; regulations are subject to formal revision procedures much more rigid than those RAC and the NIH follow in modifying the Guidelines. Neither are the Guidelines simply statements of good practice. The RAC and the NIH have been responsive to change, not as quickly perhaps as some would have preferred, but quickly enough that the advance of science has not been significantly impeded.

Mr. Thornton then recognized Dr. Baltimore who referred to the December 4, 1981, proposal which RAC had recommended for publication in the **Federal Register** (46 FR 59368). Dr. Baltimore said that the proposal had elicited tremendous response. He said that conversion to a voluntary code of standard practice, as described in the December 4, 1981, **Federal Register**, is appropriate. Although the current NIH Guidelines are not formal regulations, they have instituted an informal regulatory process. He expressed hope that the philosophy of voluntary compliance expressed in the December 4, 1981, proposal would be accepted.

Dr. Baltimore suggested that some of the concerns expressed about the December 4, 1981, proposal by correspondents could be addressed and met by modifications. Some correspondents had expressed concern that the IBCs would be dismantled. Dr. Baltimore assumed that with the language of the December 4, 1981, proposal, the IBCs would remain in place. He said he had, however, prepared an amendment, which might be added during the discussion, specifying a continuing role for IBCs.

Dr. Baltimore said that in setting P1 containment conditions, the December 4, 1981, proposal implies there could not be deliberate release of recombinant organisms into the environment. It is clear from the letters received in response to the proposal, however, that some people would prefer an explicit statement to that effect. Dr. Baltimore said that if RAC felt it was necessary, he would support an amendment to the December 4, 1981, proposal to accomplish that aim.

Finally, Dr. Baltimore suggested the language of Section I–A might be modified to include a strong statement that although voluntary, adherence to the Guidelines is strongly recommended. He said the December 4, 1981, proposal with these amendments would be responsive to comments received. He then moved the proposal appearing in the December 4, 1981, **Federal Register** (46 FR 59368) as an item for discussion. The motion was seconded by Dr. McGarrity.

Dr. Baltimore made an additional statement in response to certain written comments received. He said that he has never hidden his affiliation with the company, Collaborative Research, of Waltham, Massachusetts. He stressed, however, that if he were acting for the company, he would not be supporting the December 4, 1981, proposal because he said it is not in the interests of any institution in the Boston area, as it might lead to more stringent regulation at the local level. He said he supported the December 4, 1981, proposal becaue he believes it is correct.

Dr. Nightingale said that letters commenting on the proposals indicate many remaining concerns in both the scientific and public sectors. In her view, these concerns are not adequately addressed by the December 4, 1981, proposal even if that proposal were modified as just suggested by Dr. Baltimore.

Dr. Nightingale said that there is not a clear consensus for eliminating the mandatory nature of the Guidelines or eliminating the requirement for IBCs. She said the issue of scale-up needs further discussion. She expressed the belief that removing the mandatory nature of the Guidelines would stimulate a variety of legislative actions across the country, possibly resulting in regulatory variation from location to location. She also suggested that although the probability of an event with disastrous consequences is very small, one must acknowledge that gaps in scientific knowledge exist; if such a very rare event should occur, there could be tremendous backlash against the scientific community.

Dr. Nightingale said the December 7, 1981 (46 FR 59734, Part 7, "Gottesman"), proposal would simplify the Guidelines and remove many restrictions. Dr. Nightingale said she had a list of at least six ways in which the Gottesman proposal could be further simplified, and restrictions further removed, by the next RAC meeting. Dr. Nightingale then moved acceptance of the December 7,

1981, "Gottesman" propoal as a substitute motion with a commitment to continue to review, reorganize, simplify, and remove restrictions from the Guidelines as expeditiously as possible. Dr. Fedoroff seconded the motion.

Dr. Berns said that the current Guidelines are cumbersome and complex. The RAC has several options. The most significant issue is the mandatory nature of the Guidelines. He thought having IBCs is good, and recommended keeping the RAC. He stated a preference for readily understandable Guidelines.

Dr. Mason said RAC has acted responsibly in the process of reviewing the Guidelines. He supported the need for IBCs in both academia and industry. Indeed this type of activity should not be limited to the recombinant DNA field but should be encouraged generically. He feared that RAC, by its endorsement for publication of the December 4, 1981, proposal, did not convey to the public the importance of IBCs. Dr. Mason suggested that certain issues should be carefully scrutinized, including deliberate release or recombinant containing organisms into the environment and the cloning of genes for drug resistance and for certain toxins. Dr. Mason expressed the belief that the NIH Guidelines ultimately should and will become voluntary, but suggested they should remain mandatory for the time being for at least two reasons: (1) More information should be collected, particularly in regard to some of the areas currently prohibited; and (2) the public is not yet ready for voluntary guidelines.

Dr. Goldstein said that he could not support the December 4 proposal. He said that he supports the December 7 proposal as it simplifies the Guidelines, specifies IBCs, and maintains mandatory Guidelines. He stated that haphazard local regulations, varying from community to community, and hindering the research, will result if national oversight is not maintained. He felt the December 7, 1981, proposal does not deal adequately with large-scale work and that area should be reviewed.

Ms. King noted that at the September 8–9, 1981, RAC meeting she had not supported what became the December 4, 1981, proposal. She believes the December 7, "Gottesman" proposal is where the RAC should begin in trying to reach a final position. She expressed the belief that regulation is justified by concerns about safety. Arguments that recombinant DNA is no more dangerous than other forms of biomedical research have been advanced. This does not lead Ms. King to the conclusion that only a voluntary code of conduct is necessary. She suggested, rather, that if other research areas pose similar risk, then perhaps they too should be regulated. She favored mandatory Guidelines with sanctions and a monitoring system. The structure should not yet be dismantled, nor should it be made voluntary. Otherwise a system of fragmented regulations at the state and local level might develop.

Mr. Thornton recognized Dr. Gottesman who had authored the December 7, 1981, proposal. Dr. Gottesman said her proposal is based on the assessment of risks in the document "Evaluation of the Risks Associated with Recombinant DNA" (46 FR 59385). She noted that that document had been generated by the Working Group on Revision of the Guidelines during the summer of 1981. On the basis of that evaluation, she had concluded that there are several types of experiments about which questions remain or about which so little is known that no absolute conclusion can be drawn. For these types of experiments she felt a mandatory record-keeping and oversight mechanism is appropriate.

Dr. Gottesman said her proposal requires RAC review and NIH approval for certain experiments involving toxin genes, drug resistance genes, and release into the environment. Responsibility for oversight of certain other experiments is delegated to the IBCs. The types of experiments to be reviewed and IBC review procedures might be modified by RAC. RAC may wish to permit the IBCs greater leeway in lowering containment for certain experiments. Dr. Gottesman noted that her proposal does not alter the status of currently exempt experiments.

Mr. Daloz said that specialists in general tend to develop tunnel-vision so that their own concerns become uppermost in their minds. He noted that many laws and guidelines regulate our daily lives, and that even if the NIH Guidelines were eliminated, other agencies might institute guidelines or regulations. Mr. Daloz expressed his support for the December 7, 1981, proposal; he said, in any event, the IBCs should be retained.

Dr. McKinney said he had discussed the December 4 and December 7 proposals with scientists, lawyers, and representatives of commercial organizations. He said the researchers he had spoken with are approximately evenly divided in their support of mandatory vs. voluntary Guidelines. Regarding the current prohibitions, Dr. McKinney said many people felt certain experiments should be monitored and controlled.

Dr. McKinney said that previously the RAC had extricated itself from "regulating" large-scale activities. He felt the reintroduction of the question of how to oversee large-scale work was retrogressive; RAC should address science issues and avoid reviewing large-scale activities *per se*. Finally, Dr. McKinney noted that some correspondents mentioned the negative effects the Guidelines have had on research. He said the committee must also take into account the beneficial aspects of the review process; in his view the benefits far outweigh any negative aspects. He said RAC would be remiss if it eliminated oversight over recombinant DNA research before more data are accumulated.

Mr. Mitchell said he had made a rough analysis of the opinions submitted by commentators on the proposals. According to his estimate, approximately half favored the December 4 proposal; the other half favored either the current Guidelines or modest changes therein, or the December 7 proposal.

Mr. Mitchell said the press gives the impression that the recombinant DNA field is advancing very rapidly. These accounts do not support the allegation that the Guidelines have inhibited research. He suggested that should the NIH change the Guidelines substantially, RAC would find itself in an untenable position; it would forfeit the opportunity to "move" the technology on a rational basis, and uniformity of standards would be lost. Mr. Mitchell suggested that adoption of the December 4, 1981, proposal would destroy some of the scientific community's credibility. He said that should Congress ever again consider national legislation, scientists could no longer argue they were following a policy of self-regulation.

Mr. Mitchell said he had attended a panel meeting of the California legislature's Committee on Health on December 14, 1981. He said these legislators, few of whom have a scientific background, spoke in terms of public perceptions. He questioned how many of those legislators would understand the scientific arguments or attempt to comprehend technical presentations.

Mr. Mitchell said he supported the December 7 proposal as it maintains the mandatory nature of the Guidelines and the requirement for IBCs. Dr. Fedoroff said she strongly suppored the December 7 proposal, and urged that a mechanism for further simplification be introduced.

Dr. Saginor said that the recombinant DNA issue could easily become a political football; the Guidelines have restrained politicians from using this as an issue. He added that the RAC as a central committee providing a forum for discussion is necessary. He supported the December 7, 1981, proposal.

Dr. Irving Johnson of Eli Lilly and Company said Eli Lilly had commented favorably on both the December 4 and the December 7 proposals, although he had reservations about both proposals. He said the December 4 proposal provides no "trackability". The December 7 proposal, while it simplifies the Guidelines, perpetuates unnecessary bookkeeping. He said that Eli Lilly and Company recommends mandatory retention of IBCs which should be required to report problems to the RAC.

Dr. Johnson pointed out that representatives of regulatory agencies are on the Interagency Recombinant DNA Committee and have liaison representatives to the RAC. These representatives are there to monitor events and suggest appropriate action to their agencies. For a company involved in interestate commerce such as Eli Lilly and Company, these agencies represent regulations which are mandatory and not voluntary.

Dr. Johnson said he had attended the November 1981 hearings of the California legislature's Committee on Health and had detected little concern over risk at that hearing. Concerns were expressed, however, over moral and ethical problems. Dr. Johnson expressed concern about again raising the issue of large-scale work and cited the safety of large-scale equipment. He proposed amending the December 4, 1981, proposal to require retention of IBCs.

Dr. McGarrity said that he has concluded that recombinant DNA research presents no hazards beyond those normally associated with microbiological research. This is not to say there are no problems in other areas of biomedical research; however, these hazards have been adequately handled. He stated that it is time to stop the discriminatory treatment of recombinant DNA research. He favored the December 4, 1981, proposal with some modifications.

Dr. Holmes said he favored retaining mandatory Guidelines and the requirement for IBCs. He rejected the argument that recombinant DNA activities should not require oversight because other areas of microbiological or biomedical research do not have special oversight. He said he would support the December 7, 1981 proposal with the addition of a recommendation that IBCs also review non-recombinant DNA research that is similar to research covered by Section III of the Guidelines.

Dr. Baltimore reiterated his belief that recombinant DNA research is no more hazardous than experiments in the mainstream of biomedical research. He felt this was the judgement of a majority of the scientific community, and that the December 4, 1981, proposal reflects this consensus. He said fear of local regulation or fear of leaving industry with no code for legal protection were not reasons for maintaining mandatory Guidelines. Adoption of the December 4, 1981, proposal would send a message to States and localities that the RAC concludes that regulations are not necessary. Finally, Dr. Baltimore said that the CDC "Classification of Etiological Agents on the Basis of Hazard" is not appropriate for use in classifying recombinant DNA experiments.

Dr. Lewis of the National Science Foundation suggested greater flexibility in IBC specifications might be desirable. Dr. Landy said that he supported the original Baltimore-Campbell proposal, and subsequently the December 4, 1981, proposal, as the only intellectually honest recognition of the relationship between the unestablished potential risk in recombinant DNA research, and known risk in other areas of research which are not regulated. In attempting to rationalize support for greater controls over recombinant DNA research than over work with known pathogens, Dr. Landy said the training, procedures and restraints applied by the select group of investigators studying pathogens would not necessarily have been followed by all those now using recombinant DNA techniques.

Dr. Gottesman concurred with Dr. Landy's rationalization and added that investigators studying pathogens know the properties of these organisms; recombinant organisms might express unexpected properties.

Dr. Maas said he saw no logic in having guidelines for one type of experimental procedure, which is rapidly becoming a very commonly employed technique, and having no regulations for other types of more dangerous procedures, such as work with chemical carcinogens.

Dr. Gottesman said that mandatory guidelines are not necessarily synonomous with bureaucracy. She noted that the December 7, 1981, proposal no longer requires RAC review and NIH approval for large-scale procedures; rather it specifies that large-scale experiments be approved by the IBC. She said the definition of large-scale might be revised. Dr. Gottesman agreed with Dr. Baltimore that the CDC Classification of Etiological Agents in not perfect, but she said the alternative in the December 4 proposal of "use whatever you have and figure it out yourself" is not better. If RAC cannot find a better mechanism than the CDC classification, IBCs and PIs individually will not be able to make better decisions.

Ms. King said that the central issue is mandatory vs. voluntary guidelines. She said she was concerned with questions of process. She referred to Dr. Baltimore's statement that only a minority of scientists believe there may be some safety concerns with respect to recombinant DNA research. She said the public cannot ascertain whether that statement is accurate. The RAC did not cross-examine those who submitted written comments. Ms. King said RAC members should be aware of what she considers to be defects in process, and therefore err on the side of caution in deciding between the December 4 and December 7 proposals.

Dr. Nightingale praised the more extensive attempts to solicit comments on these proposals than had occurred in the past. As a result of this, the comments received were more varied than in the past. However, she felt it was only one small step in really assessing what the public feels. Referring back to Dr. Baltimore's statement, Dr. Nightingale said that disagreement does exist within the scientific community on whether there are unique risks of recombinant DNA research. She said that a major issue is voluntary vs. mandatory IBCs. She said that the December 7, 1981, proposal could be simplified and reorganized to make it easier to read and less cumbersome. She suggested that Section III–C could be eliminated; that the criteria for defining large-scale could be revised to emphasize inoculum size rather than volume; that Section IV could be simplified and reorganized; that the bureaucracy within IBCs could be greatly simplified; that the section dealing with whole or defective viruses could be simplified; that Section III–B–2–a and Section III–B–2–b dealing with etiological agents could be combined; and that all work in nonpathogens could be performed at PI containment. She viewed the December 7, 1981, proposal as a first, very positive step towards reducing complexity and restrictions.

Dr. Levine attempted to address the question of why recombinant DNA research is singled out for special consideration while other biomedical research, using inherently much more dangerous organisms, is not. He said the answer is in the historical context. Work

with pathogens has had an extraordinary safety record for decades. The reason there was so much interest in control of recombinant DNA is that recombinant DNA technology became available in the 1970s, in an era of regulation. He cited procedures for research involving human subjects, which changed drastically in the early 1970s. He said he supports these constraints as they protect the public, as well as individual subjects, and they facilitate communication between the public and clinical investigators. He said being responsive to the public is very important and if a significant segment of the public is still concerned about recombinant DNA, this committee should be sensitive to that concern. He said that he would like to see something like the December 4, 1981, proposal ultimately adopted, but not immediately.

Dr. Ahmed said he wished to quote and highlight several points from the letter from the Public and Scientific Affairs Board of the American Society for Microbiology. He quoted from that letter that, "Our concern is for the fact that only sparse information is available for other host-vectors. With less characterized systems, new combinations may result in organisms with potentially increased pathogenicity than either the donor or the recipient." Dr. Ahmed further quoted, "We are not only concerned with the paucity of information but also with the lack of mechanisms for its dissemination. Many workers using modern genetic technology are not versed in pathogenic microbiology and cannot be assumed to have proper training or access to up-to-date information."

Dr. Martin said he believed as a scientist that recombinant DNA should not be singled out for special oversight. However, this position must be viewed within the historical contest. He said that the State legislators and County supervisors with whom he had spoken are not primarily interested in the scientific basis for relaxation or eliminataion of the Guidelines, but rather in public opinion. RAC must be careful not to excite a public reaction that could result in greater bureaucratic and regulatory problems from local jurisdictions.

Dr. Saginor said he would like to propose an amendment to the December 7, 1981, proposal, should it pass, that a working group be formed to further refine, simplify and reorganize that proposal, and that this group report to the RAC at a future meeting.

A discussion was held of the proper parliamentary procedure for the Committee to use to proceed. Mr. Thornton suggested that the Committee might vote now on Dr. Nightingale's motion to substitute the December 7 proposal for the December 4 proposal. This would result in the Committee choosing which "vehicle" it wished initially to adopt. Following this, RAC members could propose amendments to "perfect" the vehicle chosen, before the final vote on it.

Dr. Baltimore "called the question." By a vote of nineteen in favor, two opposed, and no abstentions, the RAC agreed to limit further debate and to vote on the motion to substitute the December 7, 1981, proposal for the December 4, 1981, proposal as the vehicle to be used for further amendments. Dr. Baltimore said that although, following this vote, any aspect of the winning proposal would be open for further amendments, he felt the vote should be viewed as a decision about whether "to go in the voluntary or mandatory direction." Dr. Nightingale reminded the RAC that her motion included the commitment to work towards future simplification of the Guidelines. By a vote of sixteen in favor, five opposed, and no abstentions, the RAC adopted the substitute motion, thus choosing the Gottesman proposal as the vehicle to be placed before the Committee, open to further amendments.

Mr. Thornton recognized Dr. Susan Wright. Dr. Wright focused her comments on large-scale applications as she thought that while many other issues are being addressed, the RAC was not adequately addressing that issue. She said the primary focus of RAC has been on the hazards of research, not the hazards of industrial processes. She said that one cannot dismiss change of scale with regard to accidental release of recombinant organisms. She felt the data base on industrial hazards is very poor. She said she had heard some industrialists in other countries were considering using open fermentation tanks. If there is no oversight, companies will use whatever fermentation process they think is in their best interest. There are irresponsible companies willing to cut corners and take risks to try to gain a competitive advantage over responsible companies. Furthermore, there are no risk assessment experiments with organisms making insulin, interferon, etc. She said the committee is assuming that whatever product is being made will be harmless.

Dr. Wright said the RAC recommendation at the previous meeting to exempt from NIH review, certain large-scale experiments utilizing *E. coli* K-12, *Saccharomyces cerevisiae* and *Bacillus subtilis* host-vector systems was an error which produced a major gap in oversight. She urged the RAC to reconsider and re-evaluate its oversight over large-scale work.

Dr. Irving Johnson of Eli Lilly and Company said that industry has produced hundreds of gallons of the causative agents of polio, diphtheria, whooping cough, etc., with no great hazard to workers or to the environment, and in fact with great benefit to the population. Dr. Johnson said the only open vats he is aware of are in the beer brewing industry. Most industrial fermentations are generally highly contained to protect against contamination. Inocula are introduced into the growth tank through a rigid stainless steel structure. The connection does not leak and is steam sterilized.

Dr. Wright said she was not making a categorical statement about hazards, but rather about the data base. In her opinion, the data are extremely poor and incomplete, and assumptions that problems will be uncomplicated or easy to deal with are premature. These new technologies should remain under RAC review until a better data base develops.

Dr. Mason said that many industrial issues, though of concern, are beyond the scope of the RAC. Local, State or Federal authorities that make on-site inspections may wish to evaluate these issues, but RAC should not. Dr. Ahmed felt a distinction should be drawn between organisms concentration and total amount in industrial processes.

Dr. Gottesman said that the December 7, 1981, proposal still requires that non-exempt large-scale procedures be reviewed by the local IBC before the project begins, and PI-LS containment would still apply. It extends to all large-scale experiments the conditions approved by RAC at the previous meeting for certain large-scale experiments.

Dr. Berns questioned the language of Section I-B, *Definition of Recombinant DNA Molecules*, in the December 7, 1981, proposal. The relevant text of Section I-B reads as follows:

> Synthetic DNA segments likely to yield a potentially harmful polynucleotide or polypeptide (e.g., a toxin or a pharmacologically active agent) shall be considered as equivalent to their natural DNA counterpart. If the synthetic DNA segment is not expressed *in vivo* as a polynucleotide or polypeptide product, it is exempt from the Guidelines.

Dr. Gottesman pointed out that this is a reformulation of text which appears as Section III-E of the current (July 1, 1981) Guidelines. Dr. Berns suggested the real issue is whether the synthetic fragment would produce a biologically active product; he proposed to amend the

language by adding the phrase "biologically active" before the word "polynucleotide" in the last sentence. Dr. Nightingale, who had proposed the motion being considered, and Dr. Feroroff, the seconder of the motion, accepted the amendment.

Dr. Saginor then proposed an amendment which would explicitly state that a working group be appointed to review and attempt to simplify further the Guidelines and to report to the RAC at a future meeting. Dr. Nightingale, noting this intent was part of her original motion, accepted the amendment, as did Dr. Fedoroff.

Mr. Thornton called the question on Dr. Nightingale's motion as modified by amendments. By a vote of seventeen in favor, three opposed, and no abstentions the RAC recommended adoption of the December 7, 1981, proposal with amendments. Mr. Thornton said a working group to refine the proposal would be designated at a later date, in accordance with the motion.

Dr. McGarrity asked the committee to state for the record that RAC sees no need for additional state and local ordinances governing recombinant DNA activities. Dr. Liberman, the biological safety officer at MIT, advised against adoption of Dr. McGarrity's statement as he viewed it as counter-productive. Based on his experience as a member of the Boston Biohazards Committee he sees growing community interest in overseeing non-recombinant biohazards as recombinant systems as being handled.

Dr. Ahmed said he thought adoption of Dr. McGarrity's statement would be viewed as arrogance on the part of the RAC, saying "our views are gospel, and don't second guess us."

Mr. Mitchell said that he is in sympathy with the motion since he is concerned about fragmentation at the State and local level. However, knowing the independence of legislative bodies, it might not be well taken. He suggested that if the statement were reworded it might be more successful. Dr. McGarrity agreed and withdrew the proposal in order that revised text could be prepared for consideration later in the meeting.

Dr. Mason spoke against Dr. McGarrity's proposal, as it runs counter to usual regulatory practice, in which States and localities may regulate as long as their requirements are at least as stringent as Federal requirements. He added that RAC's recommendation would not be binding.

Dr. Holmes made a motion that there be added to the Guidelines a statement to the effect that:

> It is not clear that the biohazards associated with recombinant DNA are unique or different from biohazards associated with other work with pathogenic organisms; therefore, RAC encourages local Institutional Biosafety Committees to establish procedures for review of experiments not involving recombinant DNA, which nonetheless, involve biohazards such as those addressed in Section III of the Guidelines.

Dr. Fedoroff seconded the motion. Dr. Landy suggested that a different statement be substituted for Dr. Holmes' proposed language to the effect that:

> The Recombinant DNA Advisory Committee wants to point out the absence of demonstrated risk or danger posed by recombinant DNA research The continuance of the Guidelines for recombinant DNA research is made with full appreciation of the fact that other areas of research in which some risk has been demonstrated are without analogous guidelines.

Dr. Landy said such a statement would make clear to the public that RAC's recommendation to maintain guidelines is not based on demonstrated risk, but on potential risk.

Dr. Ahmed asked whether NIH has the authority to expand the purview of the IBC's as in Dr. Holmes' statement. Dr. Talbot replied that such a statement could be sent to the IBCs as a recommendation.

Dr. Goldstein said that he thought Dr. Landy's proposal could "stir up a hornet's nest," regenerating the situation of previous years with recombinant DNA. Ms. King said she could not support Dr. Landy's proposal as she questioned the phrase "absence of demonstrated risk." Dr. Berns moved to table Dr. Holme's proposal. By a vote of seventeen in favor, three opposed, and no abstentions, the proposal was tabled.

Dr. Levine called the committee's attention to the report of the Working Group on Revision of the Guidelines entitled "Evaluation of the Risks Associated with Recombinant DNA Research" and particularly Part IV-A of the report, "Summary Analysis of Risks" (46 FR 59390). He said the conclusion is that most potential recombinant DNA risks envisaged in 1975 are now considered nonexistent. Ms. King said RAC should emphasize that available data cited in that report support and justify RAC's recommendation of the December 7, 1981, proposal. She suggested the RAC might formally reaffirm the "Summary Analysis of Risks." Dr. Martin suggested this text might be used as a preamble to the introduction of the new Guidelines by the NIH Director. Dr. Holmes moved that the Director is requested to consider the "Summary Analysis of Risks" (46 FR 59390) as he determines a preamble to the revised Guidelines. Dr. Nightingale seconded the motion. She emphasized that her earlier motion for adoption of the December 7, 1981, proposal was based on the document "Evaluation of the Risks Associated with Recombinant DNA." She expected this document would be published as an integral part of the decision document. Dr. Mason hoped the document would note the different options considered by the RAC.

Ms. King suggested Dr. Holmes' motion be amended to call the attention of the NIH Director not just the "Summary Analysis of Risks" (46 FR 59390) but also the motion which originally established the Working Group on Revision of the Guidelines, the Working Group's agenda, and its complete report.

Dr. Talbot asked if the motion might not be withdrawn, with the assurance that NIH would bring all of these items to the Director's attention without the necessity of a motion. Ms. King said she would prefer a specific motion since the Working Group report had not been formally endorsed by the RAC at the September 1981 meeting and since the RAC action today accepting the December 7, 1981, proposal is based on that report. Dr. Holmes reworded his motion to request the summary information discussed be included in the Director's preamble. Dr. Nightingale, who had seconded Dr. Holmes' earlier motion, also agreed.

Dr. Wright said that if there were to be a general statement on risks, then it should be made clear which industrial problems the RAC is not dealing with, so that no one thinks this is a global statement covering both research and industrial risks. Dr. Ahmed suggested that language be inserted indicating that the report does not address industrial scale-up. Dr. Berns noted that the NIH, on the advice of the RAC, had issued "Physical Containment Recommendations for Large-Scale Uses of Organisms Containing Recombinant DNA Molecules;" RAC, however, is no longer evaluating mechanical details in individual large-scale applications. Dr. Landy opposed Dr. Ahmed's suggestion on the introduction of a specific statement on industrial considerations as it would dilute the general policy statement.

Dr. Pinon moved to table the motion; he preferred that ORDA bring these items to the attention of the Director, NIH, without the necessity of a formal motion. By a vote of ten in favor, eight opposed, and two abstentions, the motion to table carried.

After a brief recess, Dr. Mason moved to reconsider the action in order to provide the Director with a clear indication of RAC intent. He felt the previous vote revolved about procedural issues rather than intent. By a vote of ten in favor, four opposed, and three abstentions, the motion to reconsider was adopted.

Ms. King then moved that "the RAC specifically call to the Director's attention that the action taken on the December 7, 1981, proposal results from analysis and consideration of the report entitled 'Evaluation of the Risks Associated with Recombinant DNA Research' prepared by the Working Group on Revision of the Guidelines. The vote on the December 7, 1981, proposal implements the Working Group report."

By a vote of nineteen in favor, none opposed, and one abstention, the RAC adopted Ms. King's motion as a substitute for the previous motion. Mr. Thornton then rules that unless there were objection (which there was not), the substitute motion is adopted by unanimous consent as the recommendation of the RAC.

Following an overnight recess, Mr. Thornton called the committee to order to consider language developed by Dr. McGarrity and Mr. Mitchell regarding local and state legislation. Mr. Mitchell moved acceptance of the following language:

Whereas RAC has voted to recommend significant reductions in mandatory guidelines regarding recombinant DNA activity, and

Whereas RAC in establishing said reduced guidelines did so based upon collective credible scientific knowledge and experience, and

Whereas RAC believes it to be in the best interest of recombinant DNA activity to have a central arena for the dissemination of information and continuous review, and

Whereas RAC believes the existence of uniform guidelines thereby establishes certainty and clarity in the scientific community, and

Whereas RAC believes it would be detrimental to the advancement of recombinant DNA activity to have fragmentation of guidelines across the country,

Therefore, be it resolved that RAC strongly recommends that local and state governments defer to the NIH Guidelines if enacting legislation governing recombinant DNA activity, unless it clearly establishes by credible scientific evidence that unique risk in fact exists in their particular jurisdiction.

Dr. McGarrity seconded the motion. He said the RAC action taken yesterday on the December 7, 1981, proposal would significantly relax the Guidelines. When considered in the context of possible additional local legislation, Mr. Mitchell's statement expressed RAC's judgement that the NIH Guidelines are the best possible approach at this time. It would be counterproductive for RAC to strip away bureaucracy and paperwork at the national level, only to have more bureaucracy and paperwork added at the state and local level.

Dr. Miller of the FDA strongly endorsed the sense of the motion. He said, almost without exception, the mosaic of local regulations has been more draconian and much less enlightened that the NIH Guidelines, and slower to evolve.

Dr. Martin suggested that the phrase "best interests of the public" be substituted for the phrase "best interest of recombinant DNA activity." Mr. Mitchell agreed.

Dr. Nightingale requested a clarification of the word "activity" in the motion. Mr. Mitchell replied that "activity" is an all inclusive term meant to cover research, development, production, etc.

Drs. Ahmed and Goldstein supported the sentiment expressed by the motion. However, Dr. Goldstein said he would vote against the language as he felt local communities would regard it as arrogant. Mr. Mitchell said he had chosen the verb "defer" to avoid the appearance of arrogance. The language urges that any actions be based on scientific grounds, and places the burden of proof upon advocates of local action. Dr. Friedman agreed.

Dr. Ahmed asked whether addition of the phrase "in as much as possible" would soften the language of the sentences:

* * * therefore, be it resolved that RAC strongly recommends that local and State governments defer to the NIH Guide lines * * *

Mr. Thornton thought the verb "defer" alone was actually softer.

Dr. Mason said he could envisage situations in which local action might be necessary because of irresponsible action by a local academic or industrial group. He hoped RAC did not intend to say that local action should not be taken in such cases. Dr. Goldstein stated that communities realize that while universities are under sanctions, industry is not. Dr. Berns said that Mr. Mitchell's language specifies that when local entities legislate, they should defer to the NIH Guidelines in the scientific component of the legislation.

Dr. Mason said that many aspects of industrial scale-up are not covered by the Guidelines, yet the proposed language implies the existence of such guidance. He questioned whether RAC might amend the language to remove such implications. Dr. McGarrity suggested the phrase "DNA activity" be modified to "DNA research activity."

Dr. Ahmed said he supported the resolution but would prefer that a statement, delineating the scope of RAC activities, be appended to the language. If the committee could not formulate such a statement today, he hoped the Director's preamble to the acceptance of the December 7, 1981, proposal would state that neither RAC nor the NIH deals with mechanical aspects of industrial scale-up activities.

Dr. Pinon requested that the word "credible" be deleted from the phrase "credible scientific evidence"; he thought the term redundant. Dr. Saginor, however, disagreed as he felt "scientific" and "credible" are not synonymous to the public.

Dr. Gottesman saw the proposed language as intending to say to local legislators "we are listening to your concerns, we believe we are responding to them, and we hope you will continue to have faith in RAC." She warned, however, that the language might lead legislators who had not previously thought of legislation to consider it. Dr. Nightingale concurred. She thought acceptance of Mr. Mitchell's statement might be counterproductive. Instead she suggested that the Director's preamble to the revised Guidelines might state that these Guidelines are based on the best available information and, it is hoped they will be applied nationally. She preferred this procedure to a motion indicating RAC's concern over possible local legislation. Dr. Holmes agreed, expressing concern that the motion appeard arrogant and would be counter-productive. Dr. Berns called for the question.

By a vote of sixteen in favor, none opposed, and no abstentions, the RAC voted to stop debate and to vote on the motion proposed by Mr. Mitchell, as amended. By a vote of six in favor, nine opposed, and one abstention, the motion offered by Mr. Mitchell was defeated.

II-B. Response of the Acting Director, NIAID, to the RAC Recommendations

As the deciding Federal official, having been delegated responsibility for actions relative to the NIH Guidelines, I note the following:

I was present throughout the entire February 8–9, 1982, RAC meeting (as I have been present throughout all but the first of the twenty-three meetings of the RAC.) I attest to the accuracy of the draft minutes appearing above in Part II-A of this announcement. I commend the diligence with which the RAC

considered the issues, taking into careful account the many letters received.

The first of the motions passed by the RAC discussed above in Part II-A of this announcement was to recommend acceptance of the "Gottesman" proposal for revision of the Guidelines as it had appeared in the December 7, 1981, **Federal Register** with the addition of the phrase "biologically active" before the word "polynucleotide" in the last sentence of Section I-B of the Guidelines, and with the stipulation that a working group be appointed to review and attempt to further simplify the Guidelines, to report to the RAC at a future meeting.

I accept the RAC recommendation of the "Gottesman" proposal for revision of the Guidelines with the addition of the phrase "biologically active." This is discussed further in Part IV of this announcement.

The stipulation "that a working group be appointed to review and attempt to simplify further the guidelines, and to report to the RAC at a future meeting" has been accepted. Members of a new Working Group on Revision of the Guidelines have been appointed by the RAC Chairman, a first meeting is scheduled in April 1982, and it is hoped that the Working Group will have proposed revisions ready to present at the next RAC meeting on June 28, 1982.

A second motion passed by the RAC discussed above in Part II-A of this announcement was to "specifically call to the Director's attention that the action taken on the December 7, 1981, proposal results from analysis and consideration of the report entitled 'Evaluation of the Risks Associated With Recombinant DNA Research' prepared by the Working Group on Revision of the Guidelines." During the discussion prior to the adoption of this motion, there were suggestions that the entire report "Evaluation of the Risks Associated With Recombinant DNA Research" be published as an integral part of the Decision Document accompanying the Guideline revision, i.e., be published in this announcement.

I do note that the RAC action, recommending adoption of the December 7, 1981, proposal was based on analysis and consideration of the report entitled "Evaluation of the Risks Associated With Recombinant DNA Research." I commend this report and urge interested readers to consult it. I have reproduced in Part I of this announcement the headings of the Report. I do not feel, however, that it is necessary to reproduce the entire report here. It can be found on pages 59385-59394 of the **Federal Register** of December 4, 1981.

During RAC discussion of another motion, which was not approved, as discussed above in Part II-A of this announcement, Dr. Ahmed said he hoped that in the Director's preamble to the new Guidelines (i.e., in this announcement) it would be stated that neither RAC nor the NIH deals with mechanical aspects of industrial scale-up activities.

In response to this, it might be helpful to review the history and the current status of the relationship of the NIH Guidelines to industrial scale-up. The Guidelines are mandatory for institutions which receive Federal funds for recombinant DNA research. They are not mandatory for institutions which receive no Federal funds for recombinant DNA research other than in those States and localities which have local ordinances requiring all to follow the NIH Guidelines. The Guidelines promulgated today stipulate, in Section III-B-5, that experiments involving more than 10 liters of culture require prior approval by the IBC (but not NIH). They refer to the "Physical Containment Recommendations for Large-Scale Uses of Organisms Containing recombinant DNA Molecules" which NIH issued in the **Federal Register** on April 11, 1980 (45 FR 24968) and which define the containment levels P1-LS, P2-LS, and P3-LS. Previous versions of the Guidelines, in effect earlier, involved prior RAC review and NIH approval of recombinant DNA experiments over 10 liters in volume. Industry voluntarily submitted requests to go over 10 liters in volume, which were reviewed at RAC meetings in September 1979, December 1979, March 1980, June 1980, September 1980, January 1981, and April 1981. Through the September 1980 meeting the information submitted included details of the "applicant's laboratory practices, containment equipment and facilities * * *" At the request of the RAC, and as accepted by NIH in November 1980 (45 FR 77378), this was changed to no longer require such physical containment details.

During RAC discussion of another motion, which was not approved, as discussed in Part II-A of this announcement, Dr. Nightingale suggested that the Director's preamble to the revised Guidelines (i.e., this announcement) might state that the "Guidelines are based on the best available information, and it is hoped they will be applied nationally." I heartily endorse this sentiment, and hope that any State or local government, contemplating enactment of legislation governing recombinant DNA activities, will first study the documentation of the huge effort the RAC and NIH have put into establishing and modifying the NIH Guidelines, and will defer to the NIH Guidelines unless it clearly establishes by credible scientific evidence that unique risk in fact exists in their particular jurisdiction.

III. Analysis of Correspondence Received

As noted above, 86 letters were received commenting on the proposed revisions of the Guidelines between November 1981 and February 5, 1982. These 86 letters were provided to the RAC and considered at their meeting on February 8-9, 1982. Nine additional letters were received between February 8, 1982, and March 31, 1982. This Part (III) of this announcement contains an analysis of the 95 letters received. Copies of all letters received are available for inspection at the Office of recombinant DNA Activities, Building 31, Room 4A52, NIH, Bethesda, Maryland 20205. It is expected that they will be published in a Volume 7 of "Recombinant DNA Research," a series constituting a public record of documents related to the NIH Guidelines for Research Involving recombinant DNA Molecules.

Letters in Favor of Removing All Guidelines

One commentator wrote:

> I conclude that Drs. Adelberg and Zinder has the correct position: There is no scientific justification for maintaining any Guidelines in force.

Two other commentators also wrote in support of abolishing the Guidelines. This represents one end of the spectrum of views in regard to the Guidelines. The Adelberg-Zinder recommendation was reproduced in Annex C of the December 4, 1981, **Federal Register** (46 FR 59394). As noted in the minutes of the September 10-11, 1981, RAC meeting (Annex D of the December 4, 1981, **Federal Register** (46 FR 59398)):

> Dr. Zinder requested that a motion be introduced in support of the Adelberg-Zinder proposal to eliminate the Guidelines and the RAC. No motion was introduced.

I share the view of the RAC that the proposal does not merit support, at this time.

Support for the September 1981 RAC Proposal

As discussed above, the September 1981 RAC proposal (published for comment in the **Federal Register** on December 4, 1981) would have retained the Guidelines and the RAC, but would have changed the Guidelines from mandatory to voluntary, would have

eliminated the requirement that each institution have an IBC, would have eliminated requirements for prior approval for certain experiments, would have eliminated the prohibitions (although two would be retained instead as admonishments), and would have lowered the containment levels of most experiments mandated at P2 or P3 to P1 recommendation.

Thirty-five commentators supported the principles of the September 1981 RAC proposal. The Chairman of one IBC which unanimously endorsed the September 1981 RAC proposal wrote:

In the Committee's view, recombinant DNA *per se* presents no hazard that is qualitatively different from the hazards associated with the pathogenicity of the hosts or vectors used. Hosts or vectors containing recombinant DNA should, therefore, be handled according to appropriate guidelines already in existence, such as the CDC guidelines for work with infectious organisms.

Another IBC Chairman, reporting the unanimous IBC endorsement of the September 1981 RAC proposal, wrote:

It is our Committee's belief that relaxation of the federal guidelines is based on careful study by RAC and ORDA and that public safety is not jeopardized.

Another IBC Chairman stated:

The adoption of these revisions proposed by the RAC will, in effect, place recombinant DNA research in the same voluntary compliance status as that research which employs any etiologic agent.

A County Health Department also endorsed the September 1981 RAC proposal. A University Vice President in reporting endorsement by the University of the RAC proposal, stated:

While we are in complete agreement that control of biological systems involving recombinant DNA should be at a level consistent with the appraised risk, we do not believe that the *de facto* regulation of recombinant DNA research, as represented by the current 'Guidelines' or by the Gottesman Proposal (FR December 7, 1981, is necessary to achieve adequate control).

A letter stated:

Based on my experience in medical microbiology and my service on the RAC. I believe that the RAC proposal described in the **Federal Register** is appropriate and sensible.

A foreign commentator wrote:

There seems to me to be no valid reason for mandatory regulations via guidelines of recombinant DNA research since there is no evidence that this activity is intrinsically hazardous.

Another commentator wrote:

The principle of prior review should be abandoned. It is needlessly expensive and time-consuming.

A Noble Prize winner wrote:

It makes no sense to have more stringent requirements for research that carries only vague hypothetical risks than for experiments involving known pathogenic microbes. Voluntary guidelines have worked quite well for research involving pathogens and should work equally well for microbes containing recombinant DNA * * * there is no scientific or social justification for retaining rules for recombinant DND research that inhibit creativity and waste resources.

Another Nobel Prize winner wrote:

There is widespread agreement that the risks that were once throught to be so plausible are actually remote or possible nonexistent. If that judgement is correct, and I know of no evidence to indicate otherwise, then it seems wasteful of effort and money, even counter-productive, to maintain the elaborate procedures and organizations that were set up to be guard against the hypothetical risks.

I note that the RAC carefully considered the points made in these letters; however, the RAC members were more convinced by the arguments made in support of the Gottesman proposal. I concur with this analysis.

Positions Intermediate Between the September 1981 RAC Proposal and the Gottesman Proposal

Ten letters were received supporting a position intermediate between the September 1981 RAC proposal and the Gottesman proposal.

One IBC Chairman, on behalf of his biosafety committee, wrote:

We believe the RAC proposal (**Federal Register** December 4, 1981) goes too far in removing certain restrictions, and that the Gottesman proposal (**Federal Register** December 7, 1981) is more restrictive than necessary.

The Executive Director of Industrial Biotechnology Association (IBA), writing on behalf of the Board of Directors of the IBA, commented:

We believe that the December 4 proposal by the RAC and the December 7 proposal by Dr. Susan Gottesman are each persuasive in some respects. Their best features should be combined to replace the present guidelines with a modernized version that will encourage variety and innovation to a greater extent, while retaining the confidence of both the scientific community and the general public.

The IBA proposal would combine the more stringent mandatory "Roles and Responsibilities" of the Gottesman proposal with the more lenient containment levels of the September 1981 RAC proposal. The opposite approach, combining the Gottesman proposal containment levels with the voluntary nature of the September RAC proposal, was advanced by another commentator as follows:

I have studied the two published **Federal Register** proposals for revising the NIH Guidelines for Research Involving Recombinant DNA and have concluded that neither proposal adequately meets the current situation * * *. As a result of this analysis I would like to propose a middle ground between the two published proposals. This takes the form of an advisory set of Guidelines based on the Gottesman proposal. In other words, the Gottesman proposal remains substantively intact, but is put in the form of recommendations or real Guidelines, rather than regulations.

A former member of the RAC wrote:

First, I enthusiastically endorse the concept of reducing both the containment requirements and the administrative review. However, as to the means of achieving the reduction, I don't find either proposal to be fully satisfactory * * *. In summary, I would like to see the regulatory aspects of the guidelines kept in place for a bare handful of experimental systems.

Another commentator wrote:

We urge the Recombinant DNA Advisory Committee to preserve those mechanisms at both the institutional and national levels which ensure continuity in judgement in determining the appropriate containment requirements when new host-vector systems are developed, or for other novel applications of this technology.

Another commentator stated:

However, while endorsing the basic tenants of the Gottesman proposal to retain some oversight of federally-supported research on recombinant DNA, some compromise between the Gottesman and Baltimore/Campbell proposals to avoid some of the unnecessary restrictions of the former would seem appropriate.

Another commentator favored RAC adoption of the Gottesman proposal now "with a *provisional* endorsement of its own proposal as an *objective* to be achieved" in the future.

I note that the RAC carefully considered the points made in these letters, but they favored supporting the December 7 proposal rather than a position intermediate between the December 7 and December 4 proposals. I concur with this analysis.

Support for the Gottesman Proposal

Thirty-two commentators favored the Gottesman proposal. One IBC Chairman reporting unanimous endorsement by the IBC stated:

The Gottesman proposal would greatly simplify the current guidelines, eliminating considerable confusion, while creating a much more usable guide for experimental scientists to follow.

Another IBC Chairman wrote:

After some discussion, the IBC reached a consensus that the current practice does not impair research activities to a significant

degree * * *. The Committee therefore recommends that the Guidelines be adjusted as necessary, but not abolished completely, nor made purely voluntary.

The Chairman of the Cambridge, Massachusetts, Biohazards Committee stated:

We recognize the need to simplify the Guidelines, particularly the specification of containment levels. We do not, however, think it wise or necessary to abolish Institutional Biosafety Committees * * *. Our own perception is that the measured evolution of the Guidelines has been a model of the way new technologies can be introduced into densely populated areas with maximum safety and public reassurance.

A University Vice Chancellor wrote:

The Biosafety Committee feels strongly that whatever the federal action will be, every institution should maintain a strong review board. For reasons of consistency throughout the scientific world and across state lines and to forestall posssible state legislative activities, it was agreed that mandatory controls would be preferable. In addition, the community members commented that "in an era when science is suspect in the community, evidence of institutional review is most important."

An industrial commentator wrote:

First, we would like to support the continuing evolution of the recombinant DNA guidelines as our body of knowledge concerning this technology increases * * *. We believe Dr. Gottesman's proposal in general achieves these objectives, by setting reasonable standards for experiments for which sufficient information exists to do so and by assigning oversight responsibilities for certain experiments to the IBC and the NIH based on a rational assessment of potential hazard.

One member of the RAC wrote:

I believe it to be premature to do away with all mandatory aspects of the guidelines * * * The alternative proposal by Susan Gottesman constructively deals with my concerns while still doing away with much of the undesirable red tape and delays.

Another RAC member wrote:

I feel that to eliminate all mandatory aspects of the Guidelines and replace them with a voluntary code of practices is premature.

Another RAC member wrote:

I have come to the conclusion that, at least for now, we should retain the mandatory nature of the guidelines for recombinant DNA research, and the requirements for Institutional Biosafety Committees * * * My recommendation is to have a limited period of more relaxed regulations by supporting the Gottesman proposal now and immediately work to simplify the guidelines further as appropriate.

Another commentator wrote:

While decreased NIH supervision of large classes of nonhazardous experiments is certainly desirable, the RAC proposal goes rather too far, in my view, in the direction of removing controls. It would seem desirable to maintain some NIH oversight of experiments involving a degree of risk, as in Dr. Gottesman's suggested revision.

Another commentator wrote:

Part of the lesson is to avoid extremes, and that is what Dr. Gottesman's proposal tries to accomplish. I urge the RAC to adopt a final recommendation that embodies the basic philosophy expressed in her proposal.

A biological safety officer stated:

* * * some sort of organized structure for the review of recombinant DNA research activities should still be required at institutions with researchers engaged in this type of work if they are to continue to receive NIH funding.

Another commentator wrote:

I favor strongly the retention of IBC oversight and current NIH compliance requirements as advocated by Dr. Gottesman.

Another commentator wrote:

* * * it is my opinion that the RAC proposal to dismantle the Guidelines and the review process would be a major mistake. Simplification of the Guidelines might well be useful, as proposed in the Alternative Proposal.

Another commentator wrote:

The Baltimore/Campbell proposal described in the **Federal Register** would eliminate any meaningful controls of Recombinant DNA technology and would create a major hazard to the public health and safety.

A Nobel Prize winner wrote:

[I] wish now to register strong objection to the Baltimore/Campbell proposal described in the **Federal Register** of December 4, 1981, proposing that the NIH Guidelines be changed from mandatory to voluntary.

Another Nobel Prize winner wrote:

Philosophically, I am most in sympathy with the proposed guidelines in the December 4 **Federal Register**. Certainly, in hindsight, such guidelines are all that should have been present when recombinant DNA work was just beginning. However, now that we have had the history of the last years and the public concern about recombinant DNA, I question whether we can go back to essentially no regulation. I believe that we cannot and need some mechanism to protect in the future both the infant genetic engineering industry and academic work with recombinant DNA. Therefore, I support a revision like that of Dr. Gottesman described in the December 7 **Federal Register**.

As noted above in Part II-A of this announcement, the RAC at their February 8-9, 1982, meeting recommended that the Gottesman proposal be adopted. My response is given in Parts II-B and IV of this announcement.

Both the December 4 and December 7 Proposals Go Too Far

Ten commentators felt not only the December 4 proposal, but even the December 7 proposal, went too far in the direction of relaxing the Guidelines.

The Chairman to the IBC of the Harvard Medical Area, speaking for the IBC, wrote:

It is our concern that the relaxation of the NIH Guidelines would lead some local communities to regulate this activity with different standards. The impact of such an occurrence could be devastating to the scientific community and the public in the long term * * * It is our committee's opinion that a relaxation such as the ones being proposed will create many problems.

The Chairman of the IBC of the Harvard University Faculty of Arts and Sciences wrote:

Although not quite unanimous, the majority of members carried reservations concerning some of the changes being proposed. Of utmost concern was the speed with which the relaxation is being considered.

A letter on behalf of the Boston, Massachusetts, Biohazards Committee stated:

* * * we wish to indicate our strong disagreement to the three current proposals for revision of existing guidelines for RDNA research (Baltimore-Campbell, RAC, and Gottesman) which appeared in the **Federal Register** of December 4, and 7, 1981. We support the continued measured and reasonable improvement in the current guidelines, but we believe that the current proposals in their present form are not consistent with this aim.

Another commentator wrote:

I would much prefer to see the RAC carry out a full review of its policy of the last three years of progressively dismantling controls, and to decide to strengthen the guidelines * * *

Another commentator wrote:

I favor continuation of the present Guidelines with their capacity for evolution; I would accept the Gottesman revisions as the "lesser-of-two-evils" but do not endorse them.

I note that the RAC carefully considered the points made in these letters, but they were more convinced by the arguments made in support of the Gottesman proposal. I concur with this analysis.

Special Concern for Maintaining the Role of Institutional Biosafety Committees

One letter sent by the three Congressmen, all members of the Committee on Science and Technology, raised concerns about the September 1981 RAC proposal. They wrote:

It is our belief that some scientific experiments should require public review. The scientific community cannot isolate itself from the larger community that it serves and presume that an individual scientist is always capable of deciding that the degree of hazard associated with a particular experiment is acceptable to the public. Moreover, where public funds are spent, the expenditure of those funds carries with it an accountability to the public * * *. Scientists must accept (and we believe the majority do) the need to restrict certain hazardous laboratory practices to protect health and safety while realizing that these restrictions do not represent an infringement of their intellectual freedom * * *. If the Guidelines become only a voluntary code of standard practice, private firms will be more likely not to comply * * *. We would urge at the very least that the system of Institutional Biosafety Committees not be allowed to terminate.

A number of other commentators who wrote in support of the Gottesman proposal also stressed the importance of maintaining the system of IBCs. The proposal recommended by the RAC, and being promulgated today, does indeed continue to mandate the existence and functions of the IBCs. Remaining mandated in the Guidelines are Section IV–D–2, "Membership and Procedures of the IBC" and Section IV–D–3, "Functions of the IBC."

Cloning of Toxin Genes

One commentator wrote:

I see no logic for singling out toxins for special treatment in the guidelines or for dividing toxin experiments into classes dependent on LD_{50}.

The Guidelines continue to single out experiments involving the cloning of toxin genes for special treatment, and to divide such experiments into classes dependent on LD_{50}. This is done in Section III–A–1 and Appendix F of the Guidelines promulgated today. (This text is essentially identical to what was previously Section I–D–2 and Appendix G of the July 1, 1981, version of the Guidelines (46 FR 34462).) The development of these sections of the Guidelines is described in detail in the July 1, 1981. "Decision Document" (46 FR 34455). It involved the deliberation of an expert Ad Hoc Working Group on Toxins, and extensive discussion at the RAC meeting of April 23–24, 1981, prior to their acceptance by NIH Director Donald Frederickson. I believe that these provisions should continue in the current Guidelines.

Title of Section III–B–2

One commentator suggested that the title of Section III–B–2 "should be expanded to include Class 4 and Class 5 agents since these two categories are discussed under this section in subpart III.B.2.b."

This suggestion has been taken. The proposed title as it appeared in the December 7, 1981, **Federal Register** was "III–B–2. *Experiments in Which DNA from CDC Class 2 or Class 3 Agents is Cloned in Nonpathogenic Prokaryotic or Lower Eukaryotic Host-Vector Systems.*" This has been changed in the Guidelines promulgated today to also include Class 4 and Class 5 agents.

Incorrect Citation of and Conclusion from Publication

Two commentators cited a publication by Drs. Portnoy and Falkow as follows:

Unexpected surprises still do arise as was seen by * * * the converting of *E. coli* K–12 into a pathogen through the cloning of a hemolysin determinant from the wild type strain (Portnoy & Falkow, 1981, *J. Bacteriol.* *148*:877)."

* * * it has been shown that K–12 can become significantly pathogenic under certain conditions. For example, a recent report by Portnoy and Falkow (*J. Bacteriol* *148*:877, 1981) describes the acquisition of mouse lethality by K–12 as a consequence of receiving a cloned hemolysin determinant from a wild strain of *E. coli.*

Looking up the paper cited, I find the article by Portnoy and Falkow is the *Journal of Bacteriology 148*, 877–883, 1981, to be entitled "Virulence-Associated Plasmids from *Yersinia enterocolitica* and *Yersinia pestis.*" There are no experiments described in this paper which involve *E. coli* K–12, or the cloning of a hemolysin determinant. There is another paper by Dr. Stanley Falkow which does deal with the cloned hemolysin determinant in *E. coli.* This is "Haemolysin Contributes To Virulence of Extra-intestinal *E. coli* Infections" by R. A. Welch, E. P. Dellinger, B. Minshew and S. Falkow, *Nature,* 294, 665–667, 1981. Data in that paper indicate that while virulence of "wild type" *E. coli* was enhanced considerably by introduction of a DNA sequence coding for hemolysin, only a slight increase in virulence was seen when *E. coli* K–12 was used. Thus, the commentators are incorrect in citing the work as showing "The converting of *E. coli* K–12 into a pathogen through the cloning of a hemolysin determinant."

IV. Summary of Guideline Changes

Immediately following this announcement there appears in a separate section of the **Federal Register** the revised NIH Guidelines for Research Involving Recombinant DNA Molecules, which are effective today. They were derived as follows:

1. The last previous complete version of the NIH Guidelines was published in the **Federal Register** on July 1, 1981 (46 FR 34462).

2. Changes were made in the Guidelines as recommended by the RAC at its September 1981 meeting and promulgated by NIH in the **Federal Register** on October 30, 1981 (46 FR 53980).

Note.—a version of the Guidelines incorporating the October 30, 1981, changes into the July 1, 1981, Guidelines appeared as "Annex E" in the **Federal Register** of December 4, 1981 (46 FR 59398).

3. Changes were made in the Guidelines as recommended in certain actions by the RAC at its February 1982 meeting and promulgated by NIH in the **Federal Register** on March 29, 1982 (47 FR 13308).

4. As noted above in Parts II–A and II–B of this announcement, the RAC recommended, and NIH is accepting today, additional changes as stipulated in the "Gottesman" proposal which had been issued for public comment in the **Federal Register** on December 7, 1981 (46 FR 59734).

5. As noted above in Part III of this announcement, the title of Section III–B–2 is being changed, in response to a written comment received.

6. The "Gottesman" proposal requires that "changes as necessary to reflect changes in Parts I–III" of the Guidelines be made in Parts IV and V of the Guidelines. This has been done.

7. Other changes have been made. For example, in Section III–B–3 of the Guidelines, and its subsections, the term "whole" animal or plant virus has been changed to "infectius" animal or plant virus to reflect more accurately the intent of this section. Section III–C has been reorganized and edited in order to clarify the intent of this section.

Dated: April 12, 1982.

Bernard Tablot,

Acting Director, National Institute of Allergy and Infectious Diseases, National Institutes of Health.

Note.—OMB's "Mandatory Information Requirements for Federal Assistance Program Announcements" (45 FR 39592) requires a statement concerning the official government programs contained in the *Catalog of Federal Domestic Assistance.* Normally NIH lists in its announcements the number and title of affected individual programs for the guidance of the public. Because the guidance in this notice covers not only virtually every NIH program but also essentially every federal research program in which DNA recombinant molecule techniques could be used, it has been determined to be not cost effective or in the public interest to attempt to list these programs. Such a list would likely require several additional pages. In addition, NIH could not be certain that every federal program would be included as many federal

agencies, as well as private organizations, both national and international, have elected to follow the NIH Guidelines. In lieu of the individual program listing, NIH invites readers to direct questions to the information address above about whether individual programs listed in the *Catalog of Federal Domestic Assistance* are affected.

NIH programs are not covered by OMB Circular A-95 because they fit the description of "programs not considered appropriate" in Section 8-(b)-(4) and (5) of the Circular.

[FR Doc. 82-10659 Filed 4-20-82; 8:45 am]

BILLING CODE 4140-01-M

federal register

Wednesday
April 21, 1982

Part III

Department of Health and Human Services

National Institutes of Health

Guidelines for Research Involving Recombinant DNA Molecules

DEPARTMENT OF HEALTH AND HUMAN SERVICES

Guidelines for Research Involving Recombinant DNA Molecules; April 1982

Table of Contents

I. Scope of the Guidelines
I-A—Purpose
I-B—Definition of Recombinant DNA Molecules
I-C—General Applicability (see IV-B)
I-D—General Definitions (see IV-C)
II. Containment
II-A—Standard Practices and Training
I-B—Physical Containment Levels
II-B-1—P1 Level
II-B-1-a—Laboratory Practices
II-B-1-b—Containment Equipment
II-B-1-c—Special Laboratory Design
II-B-2—P2 Level
II-B-2-a—Laboratory Practices
II-B-2-b—Containment Equipment
II-B-2-c—Special Laboratory Design
II-B-3—P3 Level
II-B-3-a—Laboratory Practices
II-B-3-b—Containment Equipment
II-B-3-c—Special Laboratory Design
II-B-4—P4 Level
II-B-4-a—Laboratory Practices
II-B-4-b—Containment Equipment
II-B-4-c—Special Laboratory Design
II-C—Shipment
II-C—Biological Containment
II-D-1—Levels of Biological Containment
II-D-1-a—HV1
II-D-1-b—HV2
II-D-2—Certification of Host-Vector Systems
II-D-2-a—Responsibility
II-D-2-b—Data To Be Submitted for Certification
II-D-3—Distribution of Certified Host-Vectors
III. Containment Guidelines for Covered Experiments
III-A—Experiments That Require RAC Review and NIH and IBC Approval Before Initiation
III-B—Experiments that Require IBC Approval Before Initiation
III-B-1—Experiments Using CDC Class 2, Class 3, Class 4, or Class 5 Agents as Host-Vector Systems
III-B-2—Experiments in which DNA from CDC Class 2, Class 3, Class 4, or Class 5 Agents is Cloned in Nonpathogenic Prokaryotic or Lower Eukaryotic Host-Vector Systems
III-B-3—Experiments Involving the Use of Infectious Animal or Plant Viruses or Defective Animal or Plant Viruses in the Presence of Helper Virus in Tissue Culture Systems
III-B-4—Recombinant DNA Experiments Involving Whole Animals and Plants
III-B-5—Experiments Involving More than 10 Liters of Culture
III-C—Experiments that Require IBC Notice Simultaneously with Initiation of Experiments
III-D—Exempt Experiments
IV. Rules and Responsibilities
IV-A—Policy
IV-B—General Applicability
IV-C—General Definitions
IV-D—Responsibilities of the Institution
IV-D-1—(General)
IV-D-2—Membership and Procedures of the IBC
IV-D-3—Functions of the IBC
IV-D-4—Biological Safety Officer
IV-D-5—Principal Investigator
IV-D-5-a—PI—General
IV-D-5-b—Submissions by the PI to NIH
IV-D-5-c—Submissions by the PI to the IBC
IV-D-5-d—PI Responsibilities Prior to Initiating Research
IV-D-5-e—PI Responsibilities During the Conduct of the Research
IV-E—Responsibilities of NIH
IV-E-1—Director
IV-E-1-a—General Responsibilities of the Director, NIH
IV-E-1-b—Specific Responsibilities of the Director, NIH
IV-E-2—Recombinant DNA Advisory Committee
IV-E-3—The Office of Recombinant DNA Activities
IV-E-4—Other NIH Components
IV-F—Compliance
V. Footnotes and References
VI. Voluntary Compliance
VI-A—Basic Policy
VI-B—IBC Approval
VI-C—Certification of Host-Vector System.
VI-D—Requests for Exemptions and Approvals
VI-E—Protection of Proprietary Data
Appendix A—Exemptions Under III-D-4
Appendix B—Classification of Microorganisms on the Basis of Hazard
Appendix C—Exemptions Under III-D-5
Appendix D—Actions Taken Under the Guidelines
Appendix E—Certified Host-Vector Systems
Appendix F—Containment Conditions for Cloning of Genes Coding for the Biosynthesis of Toxins for Vertebrates

I. Scope of the Guidelines

I-A. *Purpose.* The purpose of these Guidelines is to specify practices for constructing and handling (i) recombinant DNA molecules and (ii) organisms and viruses containing recombinant DNA molecules.

I-B. *Definition of Recombinant DNA Molecules.* In the context of these Guidelines, recombinant DNA molecules are defined as either (i) molecules which are constructed outside living cells by joining natural or synthetic DNA segments to DNA molecules that can replicate in a living cell, or (ii) DNA molecules that result from the replication of those described in (i) above.

Synthetic DNA segments likely to yield a potentially harmful polynucleotide or polypeptide (e.g., a toxin or a pharmacologically active agent) shall be considered as equivalent to their natural DNA counterpart. If the synthetic DNA segment is not expressed *in vivo* as a biologically active polynucleotide or polypeptide product, it is exempt from the Guidelines.

I-C. *General Applicability.* See Section IV-B.

I-D *General Definitions.* See Section IV-C.

II. Containment

Effective biological safety programs have been operative in a variety of laboratories for many years. Considerable information, therefore, already exists for the design of physical containment facilities and the selection of laboratory procedures applicable to organisms carrying recombinant DNAs [6-19]. The existing programs rely upon mechanisms that, for convenience, can be divided into two categories: (i) A set of standard practices that are generally used in microbiological laboratories, and (ii) special procedures, equipment, and laboratory installations that provide physical barriers which are applied in varying degrees according to the estimated biohazard.

Experiments on recombinant DNAs, by their very nature, lend themselves to a third containment mechanism—namely, the application of highly specific biological barriers. In fact, natural barriers do exist which limit either (i) the infectivity of a *vector,* or *vehicle,* (plasmid or virus) for specific hosts or (ii) its dissemination and survival in the environment. The vectors that provide the means for replication of the recombinant DNAs and/or the host cells in which they replicate can be genetically designed to decrease by many orders of magnitude the probability of dissemination of recombinant DNAs outside the laboratory.

As these three means of containment are complementary, different levels of containment appropriate for experiments with different recombinants can be established by applying various combinations of the physical and biological barriers along with a constant use of the standard practices. We consider these categories of containment separately here in order that such combinations can be conveniently expressed in the Guidelines.

In constructing these Guidelines, it was necessary to define boundary conditions for the different levels of physical and biological containment and for the classes of experiments to which they apply. We recognize that these definitions do not take into acount all existing and anticipated information on special procedures that will allow particular experiments to be carried out

under different conditions than indicated here without affecting risk. Indeed, we urge that individual investigators devise simple and more effective containment procedures and that investigators and institutional biosafety committees recommend changes in the Guidelines to permit their use.

II-A. *Standard Practices and Training.* The first principle of containment is a strict adherence to good microbiological practices [6-15]. Consequently, all personnel directly or indirectly involved in experiments on recombinant DNAs must receive adequate instruction. (See Sections IV-D-I-e and IV-D-5-d.) This shall, as a minimum, include instructions in aseptic techniques and in the biology of the organisms used in the experiments, so that the potential biohazards can be understood and appreciated.

Any research group working with agents with a known or potential biohazard shall have an emergency plan which describes the procedures to be followed if an accident contaminates personnel or the environment. The principal investigator must ensure that everyone in the laboratory is familiar with both the potential hazards of the work and the emergency plan. (See Sections IV-D-3-d and IV-D-5-e.) If a research group is working with a known pathogen where there is an effective vaccine it should be made available to all workers. Where serological monitoring is clearly appropriate it shall be provided. (See Section IV-D-1-f.)

II-B. *Physical Containment Levels.* The objective of physical containment is to confine organisms containing recombinant DNA molecules, and thus to reduce the potential for exposure of the laboratory worker, persons outside of the laboratory, and the environment to organisms containing recombinant DNA molecules. Physical containment is achieved through the use of laboratory practices, containment equipment, and special laboratory design. Emphasis is placed on primary means of physical containment which are provided by laboratory practices and containment equipment. Special laboratory design provides a secondary means of protection against the accidental release of organisms outside the laboratory or to the environment. Special laboratory design is used primarily in facilities in which experiments of moderate to high potential hazards are performed.

Combinations of laboratory practices, containment equipment, and special laboratory design can be made to achieve different levels of physical containment. Four levels of physical containment, which are designated as P1, P2, P3, and P4, are described. It should be emphasized that the descriptions and assignments of physical containment detailed below are based on existing approaches to containment of pathogenic organisms. For example, the "Classification of Etiologic Agents on the Basis of Hazard," [7] prepared by the Centers for Disease Control, describes four general levels which roughly correspond to our descriptions for P1, P2, P3, and P4; and the National Cancer Institute describes three levels for research on oncogenic viruses which roughly correspond to our P2, P3, and P4 levels. [8]

It is recognized that several different combinations of laboratory practices, containment equipment, and special laboratory design may be appropriate for containment of specific research activities. The Guidelines, therefore, allow alternative selections of primary containment equipment within facilities that have been designed to provide P3 and P4 levels of physical containment. The selection of alternative methods of primary containment is dependent, however, on the level of biological containment provided by the host-vector system used in the experiment. Consideration will also be given by the Director, NIH, with the advice of the Recombinant DNA Advisory Committee to other combinations which achieve an equivalent level of containment. (See Section IV-E-1-b-(2)-(b).)

II-D-1. *Pl Level.*

II-B-1-a. *Laboratory Practices.*

II-B-1-a-(1). Laboratory doors shall be kept closed while experiments are in progress.

II-B-1-a-(2). Work surfaces shall be decontaminated daily, and immediately following spills of organisms containing recombinant DNA molecules.

II-B-1-a-(3). All biological wastes shall be decontaminated before disposal. Other contaminated materials, such as glassware, animal cages, and laboratory equipment, shall be decontaminated before washing, reuse, or disposal.

II-B-1-a-(4). Mechanical pipetting devices be used; pipetting by mouth is prohibited.

II-B-1-a-(5). Eating, drinking, smoking, and storage of foods are not permitted in the laboratory area in which recombinant DNA materials are handled.

II-B-1-a-(6). Persons shall wash their hands after handling organisms containing recombinant DNA molecules and when they leave the laboratory.

II-B-1-a-(7). Care shall be taken in the conduct of all procedures to minimize the creation of aerosols.

II-B-1-a-(8). Contaminated materials that are to be decontaminated at a site away from the laboratory shall be placed in a durable leak-proof container, which is closed before removal from the laboratory.

II-B-1-a-(9). An insect and rodent control program shall be instituted.

II-B-1-a-(10). The use of laboratory gowns, coats, or uniforms is discretionary with the laboratory supervisor.

II-B-1-a-(11). Use of the hypodermic needle and syringe shall be avoided when alternative methods are available.

II-B-1-a-(12). The laboratory shall be kept neat and clean.

II-B-1-b. *Containment Equipment.* Special containment equipment is not required at the P1 level.

II-B-1-c. *Special Laboratory Design.* Special laboratory design is not required at the P1 level.

II-B-2. *P2 Level.*

II-B-2-a. *Laboratory Practices.*

II-B-2-a-(1). Laboratory doors shall be kept closed while experiments are in progress.

II-B-2-a-(2). Work surfaces shall be decontaminated daily, and immediately following spills of organisms containing recombinant DNA molecules.

II-B-2-a-(3). All laboratory wastes shall be steam-sterilized (autoclaved) before disposal. Other contaminated materials such as glassware, animal cages, laboratory equipment, and radioactive wastes shall be decontaminated by a means demonstrated to be effective before washing, reuse, or disposal.

II-B-2-a-(4). Mechanical pipetting devices shall be used; pipetting by mouth is prohibited.

II-B-2-a-(5). Eating, drinking, smoking, and storage of food are not permitted in the laboratory area in which recombinant DNA materials are handled.

II-B-2-a-(6). Persons shall wash their hands after handling organisms containing recombinant DNA molecules and when they leave the laboratory.

II-B-2-a-(7) Care shall be exercised to minimize the creation of aerosols. For example, manipulations such as inserting a hot inoculation loop or needle into a culture, flaming an inoculating loop or needle so that it splatters, and forceful ejection of fluids from pipettes or syringes shall be avoided.

II-B-2-a-(8). Contaminated materials that are to be steam sterilized (autoclaved) or decontaminated at a site away from the laboratory shall be placed in a durable leak-proof container,

which is closed before removal from the laboratory.

II–B–2–a–(9). Only persons who have been advised of the nature of the research being conducted shall enter the laboratory.

II–B–2–a–(10). The universal biohazard sign shall be posted on all laboratory access doors when experiments requiring P2 containment are in process. Freezers and refrigerators or other units used to store organisms containing recombinant DNA molecules shall also be posted with the universal biohazard sign.

II–B–2–a–(11). An insect and rodent control programs shall be instituted.

II–B–2–a–(12). The use of laboratory gowns, coats, or uniforms is required. Laboratory clothing shall not be worn to the lunch room or outside of the building in which the laboratory is located.

II–B–2–a–(13). Animals not related to the experiment shall not be permitted in the laboratory.

II–B–2–a–(14). Use of the hypodermic needle and syringe shall be avoided when alternative methods are available.

II–B–2–a–(15). The laboratory shall be kept neat and clean.

II–B–2–a–(16). Experiments of lesser biohazard potential can be carried out concurrently in carefully demarcated areas of the same laboratory.

II–B–2–b. *Containment Equipment.* Biological safety cabinets [20] shall be used to contain aerosol-producing equipment, such as blender, lyophilizers, sonicators, and centrifuges, when used to process organisms containing recombinant DNA molecules, except where equipment design provides for containment of the potential aerosol. For example, a centrifuge may be operated in the open if a sealed head or safety centrifuge cups are used.

II–B–2–c. *Special Laboratory Design.* An autoclave for sterilization of wastes and contaminated materials shall be available in the same building in which organisms containing recombinant DNA molecules are used.

II–B–3. *P3 Level.*

II–B–3–a. *Laboratory Practices.*

II–B–3–a–(1). Laboratory doors shall be kept closed while experiments are in progress.

II–B–3–a–(2). Work surfaces shall be decontaminated following the completion of the experimental activity, and immediately following spills of organisms containing recombinant DNA molecules.

II–B–3–a–(3). All laboratory wastes be steam-sterilized (autoclaved) before disposal. Other contaminated materials, such as glassware, animal cages, laboratory equipment, and radioactive wastes, shall be decontaminated by a method demonstrated to be effective before washing, reuse, or disposal.

II–B–3–a–(4). Mechanical pipetting devices shall be used; pipetting by mouth is prohibited.

II–B–3–a–(5). Eating, drinking, smoking, and storage of food are not permitted in the laboratory area in which recombinant DNA materials are handled.

II–B–3–a–(6). Persons shall wash their hands after handling organisms containing recombinant DNA molecules and when they leave the laboratory.

II–B–3–a–(7). Care shall be exercised to minimize the creation of aerosols. For example, manipulations such as inserting a hot inoculating loop or needle into a culture, flaming an inoculation loop or needle so that it splatters, and forceful ejection of fluids from pipettes or syringes shall be avoided.

II–B–3–a–(8). Contaminated materials that are to be steam-sterilized (autoclaved) or decontaminated at a site away from the laboratory shall be placed in a durable leak-proof container, which is closed before removal from the laboratory.

II–B–3–a–(9). Entry into laboratory shall be through a controlled access area. Only persons who have been advised of the nature of the research being conducted shall enter the controlled access area. Only persons required on the basis of program or support needs shall be authorized to enter the laboratory. Such persons shall be advised of the nature of the research being conducted before entry, and shall comply with all required entry and exit procedures.

II–B–3–a–(10). Persons under 16 years of age shall not enter the laboratory.

II–B–3–a–(11). The universal biohazard sign shall be posted on the controlled access area door and on all labortory doors when experiments requiring P3-level containment are in progress. Freezers and refrigerators or other units used to store organisms containing recombinant DNA molecules shall also be posted with the universal biohazard sign.

II–B–3–a–(12). An insect and rodent control program shall be instituted.

II–B–3–a–(13). Laboratory clothing that protects street clothing (e.g., long-sleeve solid-front or wrap-around gowns, no-button or slipover jackets) shall be worn in the laboratory. Front-button laboratory coats are unsuitable. Laboratory clothing shall not be worn outside the laboratory and shall be decontaminated before it is sent to the laundry.

II–B–3–a–(14). Raincoats, overcoats, topcoats, coats, hats, caps, and such street outer-wear shall not be kept in the laboratory.

II–B–3–a–(15). Gloves shall be worn when handling materials requiring P3 containment. They shall be removed aseptically immediately after the handling procedure and decontaminated.

II–B–3–a–(16). Animals and plants are not related to the experiment shall not be permitted in the laboratory.

II–B–3–a–(17). Vacuum outlets shall be protected by filter and liquid disinfectant traps.

II–B–3–a–(18). Use of hypodermic needle and syringe shall be avoided when alternative methods are available.

II–B–3–a–(19). The laboratory shall be kept neat and clean.

II–B–3–a–(20). If experiments involving other organisms which require lower levels of containment are to be conducted in the same laboratory concurrently with experiments requiring P3-level physical containment, they shall be conducted in accordance with all P3-level laboratory practices.

II–B–3–b. *Containment Equipment.*

II–B–3–b–(1). Biological safety cabinets [20] shall be used for all equipment and manipulations that produce aerosols—e.g., pipetting, dilutions, transfer operations, plating, flaming, grinding, blending, drying sonicating, shaking, centrifuging—where these procedures involve organisms containing recombinant DNA molecules, except where equipment design provides for containment of the potential aerosol.

II–B–3–b–(2). Laboratory animals held in a P3 area shall be housed in partial-containment caging systems, such as Horsfall units [19A], open cages placed in ventilated enclosures, solid-wall and -bottom cages covered by filter bonnets, or solid-wall and -bottom cages placed on holding racks equipped with ultraviolet radiation lamps and reflectors.

(Note.—Conventional caging systems may be used, provided that all personnel wear appropriate personal protective devices. These shall include, at a minimum, wrap-around gowns, head covers, gloves, shoe covers, and respirators. All personnel shall shower on exit from areas where these devices are required.)

II–B–3–b–(3). *Alternative Selection of Containment Equipment.* Experimental procedures involving a host-vector system that provides a one-step higher level of biological containment than that specified can be conducted in the P3 laboratory using containment equipment specified for the P2 level of physical containment. Experimental procedures involving a host-vector system that

provides a one-step lower level of bilogical containment than that specified can be conducted in the P3 laboratory using containment equipment specified for the P4 level of physical containment. Alternative combinations of containment safeguards are shown in Table I.

TABLE I—COMBINATIONS OF CONTAINMENT SAFEGUARDS

Classification of experiment		Alternative combinations of physical and biological containment			
		Physical Containment			
Physical containment	Biological[1] containment	Laboratory design specified for—	Laboratory practices specified for—	Containment equipment specified for—	Biological containment
P3	HV2	P3	P3	P3	HV2.
P3	HV2	P3	P3	P4	HV1.
P3	HV1	P3	P3	P3	HV1.
P3	HV1	P3	P3	P2	HV2.

[1] See section II-D for description of biological containment.

II–B–3–c. *Special Laboratory Design.*

II–B–3–c–(1). The laboratory shall be separated by a controlled access area from areas that are open to unrestricted traffic flow. A controlled access area is an anteroom, a change room, an air lock or any other double-door arrangement that separates the laboratory from areas open to unrestricted traffic flow.

II–B–3–c–(2). The surfaces of walls, floors, and ceilings shall be readibly cleanible. Penetrations through these surfaces shall be sealed or capable of being sealed to facilitate space decontamination.

II–B–3–c–(3). A foot-, elbow-, or automatically-operated hand-washing facility shall be provided near each primary laboratory exit area.

II–B–3–c–(4). Windows in the laboratory shall be sealed.

II–B–3–c–(5). An autoclave for sterilization of wastes and contaminated materials shall be available in the same building (and preferably within the controlled laboratory area) in which organisms containing recombitant DNA molecules are used.

II–B–3–c–(6). The laboratory shall have a ventilation system that is capable of controlling air movement. The movement of air shall be from areas of lower contamination potential to areas of higher contamination potential (i.e., from the controlled access area to the laboratory area). If the ventilation system provides positive pressure supply air, the system shall operate in a manner that prevents the reversal of the direction of air movement or shall be equipped with an alarm that would be actuated in the event that reversal in the direction of air movement were to occur. The exhaust air from the laboratory area shall not be recirculated to other areas of the building unless the exhaust air is filtered by HEPA filters or equivalent. The exhaust air from the laboratory area can be discharged to the outdoors without filtration or other means for effectively reducing an accidental aerosol burden provided that I can be dispersed clear of occupied buildings and air intakes.

II–B–3–c–(7). The treated exhaust-air from Class I and Class II biological safety cabinets [20] may be discharged either to the laboratory or to the outdoors. The treated exhaust-air from a Class III cabinet shall be discharged directly to the outdoors. If the treated exhaust-air from these cabinets is to be discharged to the outdoors through a building exhaust air system, it shall be connected to this system so as to avoid any interference with the air balance of the cabinet and the building ventilation system.

II–B–4–4. *P4 Level.*

II–B–4–a. *Laboratory Practices.*

II–B–4–a–(1). Laboratory doors shall be kept closed while experiments are in progress.

II–B–4–a–(2). Work surfaces shall be decontaminated following the completion of the experimental activity and immediately following spills of organisms containing recombinant DNA molecules.

II–B–4–a–(3). All laboratory wastes shall be steam-sterilized (autoclaved) before disposal. Other contaminated materials such as glassware, animal cages, laboratory equipment, and radioactive wastes shall be decontaminated by a method demonstrated to be effective before washing, reuse, or disposal.

II–B–4–a–(4). Mechanical pipetting devices shall be used; pipetting by mouth is prohibited.

II–B–4–a–(5). Eating, drinking, smoking, and storage of food are not permitted in the P4 facility.

II–B–4–a–(6). Persons shall wash their hands after handling organisms containing recombinant DNA molecules and when they leave the laboratory.

II–B–4–a–(7). Care shall be exercised to minimize the creation of aerosals. For example, manipulations such as inserting a hot inoculating loop or needle into a culture, flaming an inoculation loop or needle so that it splatters, and forceful ejection of fluids from pipettes or syringes shall be avoided.

II–B–4–a–(8). Biological materials to be removed from the P4 facility in a viable or intact state shall be transferred to a nonbreakable sealed container, which is then removed from the P4 facility through a pass-through disinfectant dunk tank or fumigation chamber.

II–B–4–a–(9). No materials, except for biological materials that are to remain in a viable or intact state, shall be steam sterilized in the double-door autoclave of the P4 facility. Other materials which may be damaged by temperature or steam shall be removed from the P4 facility unless they have been steam-sterilized (autoclaved) or decontaminated by a means demonstrated to be effectve as they pass out of the P4 facility. All wastes and other materials as well as equipment not damaged by high temperature or steam shall be removed from the P4 facility through a pass-through fumigation chamber.

II–B–4–a–(10). Materials within the Class III cabinets shall be removed from the cabinet system only after being steam-sterilized in an attached double-door autoclave or after being contained in a nonbreakable sealed container, which is then passed through a disinfectant dunk tank or a fumigation chamber.

II–B–4–a–(11). Only persons whose entry into the P4 facility is required to meet program of support needs shall be authorized to enter. Before entering, such persons shall be advised of the nature of the research being conducted and shall be instructed as to the appropriate safeguards to ensure their safety. They shall comply with instructions and all other required procedures.

II–B–4–a–(12). Persons under 18 years of age shall not enter the P4 facility.

II–B–4–a–(13). Personnel shall enter into and exit from the P4 facility only through the clothing and shower rooms. Personnel shall shower at each egress from the P4 facility. Air locks shall not be used for personnel entry or exit except for emergencies.

II–B–4–a–(14). Street clothing shall be removed in the outer side of the clothing-change area and kept there. Complete laboratory clothing, including undergarments, head cover, shoes, and either pants and shirts or jumpsuits, shall be used by all persons who enter

the P4 facility. Upon exit, personnel shall store this clothing in lockers provided for this purpose or discard it into collection hampers before entering the shower area.

II-B-4-a-(15). The universal biohazard sign is required on the P4 facility access doors and on all interior doors to individual laboratory rooms where experiments are conducted. The sign shall also be posted on freezers, refrigerators, or other units used to store organisms containing recombinant DNA molecules.

II-B-4-a-(16). An insect and rodent control program shall be instituted.

II-B-4-a-(17). Animals and plants not related to the experiment shall not be permitted in the laboratory in which the experiment is being conducted.

II-B-4-a-(18). Vacuum outlets shall be protected by filter and liquid disinfectant traps.

II-B-4-a-(19). Use of the hypodermic needle and syringe shall be avoided when alternate methods are available.

II-B-4-a-(20). The laboratory shall be kept neat and clean.

II-B-4-a-(21). If experiments involving other organisms which require lower levels of containment are to be conducted in the P4 facility concurrently with experiments requiring P4-level containment, they shall be conducted in accordance with all P4-level laboratory practices specified in this section.

II-B-4-b. *Containment Equipment.*

II-B-4-b-(1). Experimental procedures involving organisms that require P4-level physical containment shall be conducted either in (i) a Class III cabinet system or in (ii) Class I or Class II cabinets that are located in a specially designed area in which all personnel are required to wear one-piece positive-pressure isolation suits.

II-B-4-b-(2). Laboratory animals involved in experiments requiring P4-level physical containment shall be housed either in cages contained in Class III cabinets or in partial containment caging systems (such as Horsfall units[19A], open cages placed in ventilated enclosures, or solid-wall and -bottom cages covered by filter bonnets, or solid-wall and -bottom cages placed on holding racks equipped with ultraviolet irradiation lamps and reflectors) that are located in a specially designed area in which all personnel are required to wear one-piece positive-pressure suits.

II-B-4-b-(3). *Alternative Selection of Containment Equipment.*

Experimental procedures involving a host-vector system that provides a one-step higher level of biological containment than that specified can be conducted in the P4 facility using containment equipment requirements specified for the P3 level of physical containment. Alternative combinations of containment safeguards are shown in Table II.

TABLE II—COMBINATIONS OF CONTAINMENT SAFEGUARDS

Classification of experiment		Alternate combinations of physical and biological containment			
		Physical containment			
Physical containment	Biological[1] containment	Laboratory design specified for—	Laboratory practices specified for—	Containment equipment specified for—	Biological containment
P4	HV1	P4	P4	P4	HV1.
P4	HV1	P4	P4[2]	HV2	
			3		

[1] See section II-D for description of biological containment.
[2] In this case gloves shall be worn, in addition to the clothing requirements specified in II-B-4-a-(14).

II-B-4-c. *Special Laboratory Design.*

II-B-4-c-(1). The laboratory shall be located in a restricted-access facility which is either a separate building or a clearly demarcated and isolated zone within a building. Clothing-change areas and shower rooms shall be provided for personnel entry and egress. These rooms shall be arranged so that personnel leave through the shower area to the change room. A double-door ventilated vestibule or ultraviolet air lock shall be provided for passage of materials, supplies, and equipment which are not brought into the P4 facility through the change room area.

II-B-4-c-(2). Walls, floors, and ceilings of the P4 facility are constructed to form an internal shell which readily allows vapor-phase decontamination and is animal- and insect-proof. All penetrations through these structures and surfaces are sealed. (The integrity of the walls, floors, ceilings, and penetration seals should ensure adequate containment of a vapor-phase decontaminant under static pressure conditions. This requirement does not imply that these surfaces must be airtight.)

II-B-4-c-(3). A foot-, elbow-, or automatically-operated handwashing facility shall be provided near the door within each laboratory in which experiments involving recombinant DNA are conducted in openface biological safety cabinets.

II-B-4-c-(4). Central vacuum systems are permitted. The system, if provided, shall not serve areas outside the P4 facility. The vacuum system shall include in-line HEPA filters near each use point or service cock. The filters shall be installed so as to permit in-place decontamination and replacement. Water supply, liquid and gaseous services provided to the P4 facility shall be protected by devices that prevent backflow.

II-B-4-c-(5). Drinking water fountains shall not be installed in labortory or animal rooms of the P4 facility. Foot-operated water fountains are permitted in the corridors of the P4 facility. The water service provided to such fountains shall be protected from the water services to the laboratory areas of the P4 facility.

II-B-4-c-(6). Laboratory doors shall be self-closing.

II-B-4-c-(7). A double-door autoclave shall be provided for sterilization of material passing out of the P4 facility. The autoclave doors shall be interlocked so that both doors will not be open at the same time.

II-B-4-c-(8). A pass-through dunk tank or fumigation chamber shall be provided for removal from the P4 facility of materials and equipment that cannot be heat-sterilized.

II-B-4-c-(9). All liquid effluents from the P4 facility shall be collected and decontaminated before disposal. Liquid effluents from biological safety cabinets and laboratory sinks shall be sterilized by heat. Liquid effluents from the shower and hand washing facilities may be activited by chemical treatment. HEPA filters shall be installed in all vents from effluent drains.

II-B-4-c-(10). An individual supply and exhaust-air ventiliation system shall be provided. The system shall maintain pressure differentials and directional air flow as required to ensure inflow from areas outside the facility toward areas of highest potential risk within the facility. The system shall be designed to prevent the reversal of air flow. The system shall sound an alarm in the event of system malfunction.

II-B-4-c-(11). Air within individual laboratories of the P4 facility may be recirculated if HEPA filtered.

II-B-4-c-(12). The exhaust air from the P4 facility shall be HEPA filtered and discharged to the outdoors so that it is dispersed clear of occupied buildings and air intakes. The filter chambers shall be designed to allow *in situ* decontamination before removal and to

facilitate certification testing after replacement.

II-B-4-c-(13). The treated exhaust-air from Class I and Class II biological safety cabinets[20] may be discharged directly to the labaratory room environment or to the outdoors. The treated exhaust-air from Class III cabinets shall be discharged to the outdoors. If the treated exhaust-air from these cabinets is to be discharged to the outdoors through the P4 facility exhaust air system, it shall be connected to this system so as to avoid any interference with the air balance of the cabinets or the facility exhaust air system.

II-B-4-c-(14). As noted in Section II-B-4-b-(1), the P4 facility may contain specially designed areas in which all personnel are required to wear one-piece positive-pressure isolation suits. Such areas shall be airtight. The exhaust-air from the suit area shall be filtered by two sets of HEPA filters installed in series, and a duplicate filtration unit and exhaust fan shall be provided. The air pressure within the suit area shall be less than that in any adjacent area. An emergency lighting system, communication systems, and power source shall be provided. A double-door autoclave shall be provided for sterilization of all waste materials to be removed from the suit area.

Personnel who enter this area shall wear a one-piece positive-pressure suit that is ventilated by a life-support system. The life-support system shall be provided with alarms and emergency backup air. Entry to this area is through an airlock fitted with airtight doors. A chemical shower area shall be provided to decontaminate the surfaces of the suite before removal.

II-C. *Shipment.* Recombinant DNA molecules contained in an organism or virus shall be shipped only as an etiologic agent under requirements of the U.S. Public Health Service, and the U.S. Department of Transportation (Section 72.3, Part 72, Title 42, and Sections 173.386-.388, Part 173, Title 49 U.S. Code of Federal Regulations (CFR)) as specified below:

II-C-1. Recombinant DNA molecules contained in an organism or virus reguiring Pl, P2, or P3 physical containment, when offered for transportation or transported, are subject to all requirements of Section 72.3(a)-(e), Part 72, Title 42 CFR, and Sections 173.386-.388, Part 173, Title 49 CFR.

II-C-2. Recombinant DNA molecules contained in an organism or virus requiring P4 physical containment, when offered for transportation or transported, are subject to the requirements listed above under II-C-1 and are also subject to Section 72.3(f), Part 72, Title 42 CFR.

II-C-3. Additional information on packaging and shipment is given in the "Laboratory Safety Monograph—A Supplement to the NIH Guidelines for Recombinant DNA Research."

II-D. *Biological Containment.*

II-D-1. *Levels of Biological Containment.* In consideration of biological containment, the vector (plasmid, organelle, or virus) for the recombinant DNA and the host (bacterial, plant, or animal cell) in which the vector is propagated in the laboratory will be considered together. Any combination of vector and host which is to provide biological containment must be chosen or constructed so that the following types of "escape" are minimized: (i) Survival of the vector in its host outside the laboratory and (ii) transmission of the vector from the propagation host to other nonlaboratory hosts.

The following levels of biological containment (HV, or *H*ost-*V*ector, systems) for prokaryotes will be established; specific criteria will depend on the organisms to be used.

II-D-1-a. *HV1.* A host-vector system which provides a moderate level of containment. *Specific systems:*

II-D-1-a-(1). *EK1.* The host is always *E. coli* K-12 or a derivative thereof, and the vectors include nonconjugative plasmids (e.g., pSC101, ColEl, or derivatives thereof [21-27]) and variants of bacteriophage, such as lambda [28-33]. The *E. coli* K-12 hosts shall not contain conjugation-proficient plasmids, whether antonamous or integrated, or generalized transducing phages.

II-D-1-a-(2). *Other Prokaryotes.* Hosts and vectors shall be, at a minimum, comparable in containment to *E. coli* K-12 with a non conjugative plasmid or bacteriophage vector. The data to be considered and a mechanism for approval of such HV1 systems are described below (Section II-D-2).

II-D-1-b. *HV2.* These are host-vector systems shown to provide a high level of biological containment as demonstrated by data from suitable tests performed in the laboratory. Escape of the recombinant DNA either via survival of the organisms or via transmission of recombinant DNA to other organisms should be less than $1/10^8$ under specified conditions. *Specific systems:*

II-D-1-b-(1). For EK2 host-vector systems in which the vector is a plasmid, no more than one in 10^8 host cells should be able to perpetuate a cloned DNA fragment under the specified nonpermissive laboratory conditions designed to represent the natural environment, either by survival of the original host or as a consequence of transmission of the coloned DNA fragment.

II-D-1-b-(2). For EK2 host-vector systems in which the vector is a phage, no more than one in 10^8 phage particles should be able to perpetuate a cloned DNA fragment under the specified nonpermissive laboratory conditions designed to represent the natural environment either (i) as a prophage (in the inserted or plasmid form) in the laboratory host used for phage propagation or (ii) by surviving in natural environments and transferring a cloned DNA fragment to other hosts (or their resident prophages).

II-D-2. *Certification of Host-Vector Systems.*

II-D-2-a. *Responsibility.* HV1 systems other than *E. coli* K-12, and HV2 host-vector systems, may not be designated as such until they have been certified by the Director, NIH. Application for certification of a host-vector system is made by written application to the Office of Recombinant DNA Activities, National Institutes of Health, Bethesda, Maryland 20205.

Host-vector systems that are proposed for certification will be reviewed by the National Institutes of Health (NIH) Recombinant DNA Advisory Committee (RAC). (See Section IV-E-1-b-(1)-(c).) This will first involve review of the data on construction, properties, and testing of the proposed host-vector system by a Working Group composed of one or more members of the RAC and other persons chosen because of their expertise in evaluating such data. The Committee will then evaluate the report of the Working Group and any other available information at a regular meeting. The Director, NIH, is responsible for certification after receiving the advice of the RAC. Minor modifications of existing certified host-vector systems, where the modifications are of minimal or no consequence to the properties relevant to containment may be certified by the Director, NIH, without review by RAC. (See Section IV-E-1-b-(3)-(c).)

When new host-vector systems are certified, notice of the certification will be sent by the Office of Recombinant DNA Activities (ORDA) to the applicant and to all Institutional Biosafety Committees (IBCs) and will be published in the *Recombinant DNA Technical Bulletin.* Copies of a list of all currently certified host-vector systems may be obtained from ORDA at any time.

The Director, NIH, may at any time rescind the certification of any host-vector system. (See Section IV-E-1-b-

(3)-(d).) If certification of a host-vector system is rescinded, NIH will instruct investigators to transfer cloned DNA into a different system, or use the clones at a higher physical containment level unless NIH determines that the already constructed clones incorporate adequate biological containment.

Certification of a given system does not extend to modifications of either the host or vector component of that system. Such modified systems must be independently certified by the Director, NIH. If modifications are minor, it may only be necessary for the investigator to submit data showing that the modifications have either improved or not impaired the major phenotypic traits on which the containment of the system depends. Substantial modifications of a certified system require the submission of complete testing data.

II-D-2-b. *Data To Be Submitted for Certification.*

II-D-2-b-(1). *HV1 Systems Other than E. Coli K-12.* The following types of data shall be submitted, modified as appropriate for the particular system under consideration. (i) A description of the organism and vector; the strain's natural habitat and growth requirements; its physiological properties, particularly those related to its reproduction and survival and the mechanisms by which it exchanges genetic information; the range of organisms with which this organism normally exchanges genetic information and what sort of information is exchanged; and any relevant information on its pathogenicity or toxity. (ii) A description of the history of the particular strains and vectors to be used, including data on any mutations which render this organism less able to survive or transmit genetic information. (iii) A general description of the range of experiments contemplated, with emphasis on the need for developing such an H1L system.

II-D-2-b-(2). *HV2 Systems.* Investigators planning to request HV2 certification for host-vector systems can obtain instructions from ORDA concerning data to be submitted [33A, 33B]. In general, the following types of data are required: (i) Description of construction steps, with indication of source, properties, and manner of introduction of genetic traits. (ii) Quantitative data on the stability of genetic traits that contribute to the containment of the system. (iii) Data on the survival of the host-vector system under nonpermissive laboratory conditions designed to represent the relevant natural environment. (iv) Data on transmissibility of the vector and/or a cloned DNA fragment under both permissive and nonpermissive conditions. (v) Data on all other properties of the system which affect containment and utility, including information on yields of phage or plasmid molecules, ease of DNA isolation, and ease of transfection or tranformation. (vi) In some cases, the investigator may be asked to submit data on survival and vector transmissibility from experiments in which the host-vector is fed to laboratory animals (e.g., rodents). Such *in vivo* data may be required to confirm the validity of predicting *in vivo* survival on the basis of *in vitro* experiments.

Data must be submitted in writing to ORDA. Ten to twelve weeks are normally required for review and circulation of the data prior to the meeting at which such data can be considered by the RAC. Investigators are encouraged to publish their data on the construction, properties, and testing of proposed HV2 systems prior to consideration of the system by the RAC and its subcommittee. More specific instructions concerning the type of data to be submitted to NIH for proposed EK2 systems involving either palsmids or bacteriophage in *E. coli* K-12 are available from ORDA.

II-D-3. *Distribution of Certified Host-Vectors.* Certified HV2 host-vector systems (plus appropriate control strains) must be obtained from the NIH or its designees, one of whom will be the investigator who developed the system. NIH shall announce the availability of the system by publication of notices in appropriate journals.

Plasmid vectors will be provided in a suitable host strain, and phage vectors will be distributed as small-volume lysates. If NIH propagates any of the host strains or phage, a sample will be sent to the investigator who developed the system or to an appropriate contractor, prior to distribution, for verification that the material is free from contamination and unchanged in phenotypic properties.

In distributing the certified HV2 host-vector systems, NIH or its designee will (i) send out a complete description of the system; (ii) enumerate and describe the tests to be performed by the user in order to verify important phenotypic traits; (iii) remind the user that any modification of the system necessitates independent approval of the system by the NIH; and (iv) remind the user of responsibility for notifying ORDA of any discrepancies with the reported properties or any problems in the safe use of the system.

NIH may also distribute certified HV1 host-vector systems.

III. Containment Guidelines for Covered Experiments

Part III discusses experiments involving recombinant DNA. These experiments have been divided into four classes:

III-A. Experiments which require specific RAC review and NIH and IBC approval before initiation of the experiment;

III-B. Experiments which require IBC approval before initiation of the experiment;

III-C. Experiments which require IBC notification at the time of initiation of the experiment;

III-D. Experiments which are exempt from the procedures of the Guidelines.

IF AN EXPERIMENT FALLS INTO BOTH CLASS III-A AND ONE OF THE OTHER CLASSES, THE RULES PERTAINING TO CLASS III-A MUST BE FOLLOWED. If an experiment falls into class III-D and into either class III-B or III-C as well, it can be considered exempt from the requirements of the Guidelines.

Changes in containment levels from those specified here may not be instituted without the express approval of the Director, NIH, (See Sections IV-E-1-b-(1), IV-E-1-b-(2), and subsections.)

III-A. *Experiments that Require RAC Review and NIH and IBC Approval Before Initiation.* Experiments in this category cannot be initiated without submission of relevant information on the proposed experiment to NIH, the publication of the proposal in the Federal Register for thirty days of comment, review by the RAC, and specific approval by NIH. The containment conditions for such experiments will be recommended by RAC and set by NIH at the time of approval. Such experiments also require the approval of the IBC before initiation. Specific experiments already approved in this section and the appropriate containment conditions are listed in Appendices D and F.

III-A-1. Deliberate formation of recombinant DNAs containing genes for the biosynthesis of toxins lethal for vertebrates at an LD_{50} of less than 100 nanograms per kilogram body weight (e.g., the botulinum toxins, tetanus toxin, diphtheria toxin, *Shigella dysenteriae* neurotoxin). Specific approval has been given for the cloning in *E. coli* K-12 of DNAs containing genes coding for the biosynthesis of toxins which are lethal to vertebrates at 100 nanograms to 100 micrograms per kilogram body weight. Containment levels for these

experiments are specified in Appendix F.

III-A-2. Deliberate release into the environment of any organism containing recombinant DNA.

III-A-3. Deliberate transfer of a drug resistance trait to microorganisms that are not known to acquire it naturally [2A], if such acquisition could compromise the use of the drug to control disease agents in human or veterinary medicine or agriculture.

III-B. *Experiments that Require IBC Approval Before Initiation.* Investigators performing experiments in this category must submit to their Institutional Biosafety Committee (IBC), prior to initiation of the experiments, a registration document that contains a description of: (a) The source(s) of DNA, (b) the nature of the inserted DNA sequences, (c) the hosts and vectors to be used, (d) whether a deliberate attempt will be made to obtain expression of a foreign gene, and, if so, what protein will be produced, and (e) the containment conditions specified in these Guidelines. This registration document must be dated and signed by the investigator and filed only with the local IBC. The IBC shall review all such proposals prior to initiation of the experiments. Requests for lowering of containment for experiments in this category will be considered by NIH. (See Section IV-E-1-b-(3).)

III-B-1. *Experiments Using CDC Class 2, Class 3, Class 4, or Class 5 Agents [1] as Host-Vector Systems.*

III-B-1-a. Experiments involving the introduction of recombinant DNA into CDC Class 2 agents can be carried out at P2 containment.

III-B-1-b. Experiments involving the introduction of recombinant DNA into CDC Class 3 agents can be carried out at P3 containment.

III-B-1-c. Experiments involving the introduction of recombinant DNA into CDC Class 4 [51] or Class 5 agents can be carried out at P4 containment. A USDA permit is required for work with Class 5 agents [48].

III-B-2. *Experiments in Which DNA from CDC Class 2, Class 3, Class 4, or Class 5 Agents [1] is Cloned in Nonpathogenic Prokaryotic or Lower Eukaryotic Host-Vector Systems.*

III-B-2-a. Recombinant DNA experiments in which DNA from CDC Class 2 or Class agents [1] is transferred into nonpathogenic prokaryotes or lower eukaryotes may be performed under P2 containment. Specific lowering of containment to P1 for particular experiments can be approved by the IBC. Many experiments in this category will be exempt from the Guidelines (See Sections III-D-4 and III-D-5). Experiments involving the formation of recombinant DNAs for certain toxin genes require RAC review and NIH approval (see Section III-A-1), or must be carried out under NIH specified conditions as described in Appendix F.

III-B-2-b. Recombinant DNA experiments in which DNA from CDC Class 4 [51] or Class 5 agents is transferred into nonpathogenic prokaryotes or lower eukaryotes can be performed at P2 containment after demonstration that only a totally and irreversibly defective fraction of the agent's viral genome is present in a given recombinant. In the absence of such a demonstration, P4 containment should be used.

Note.—A USDA permit is required for work with Class 5 pathogens [48].

III-B-3. *Experiments Involving the Use of Infectious Animal or Plant Viruses or Defective Animal or Plant Viruses in the Presence of Helper Virus in Tissue Culture Systems.*

Note: Recombinant DNA molecules which contain less than two-thirds of the genome of any eukaryotic virus (all virus from a single Family [36] being considered identical [50]) may be considered defective and can be used, in the absence of helper, under the conditions specified in Section III-C.

III-B-3-c. Experiments involving the use of infectious CDC Class 2 animal viruses [1], or defective CDC Class 2 animal viruses in the presence of helper virus, can be performed at P2 containment.

III-B-3-b. Experiments involving the use of infectious CDC Class 3 animal viruses [1], or defective CDC Class 3 animal viruses in the presence of helper virus, can be carried out at P3 containment.

III-B-3-a. Experiments involving the use of infectious CDC Class 4 or Class 5 animal virus [1], or defective CDC Class 4 or Cass 5 animal viruses in the presence of helper virus, may be carried out under P4 containment. A USDA permit is required for work with CDC Class 5 pathogens [48].

III-B-3-d. Experiments involving the use of infectious animal or plant viruses, or defective animal or plant viruses in the presence of helper virus, not covered by Sections III-B-3-a, III-B-3-b, or III-B-3-c may be carried out under P1 containment.

III-B-4. *Recombinant DNA Experiments Involving Whole Animals and Plants.*

III-B-4-a. DNA from any source except for greater than one quarter of a eukaryotic viral genome may be transferred to any non-human vertebrate organism and propagated under conditions of physical containment comparable to P1 and appropriate to the organism under study [2A].

III-B-4-b. For all experiments involving whole animals and plants and not covered by III-B-4-a, the appropriate containment will be determined by the IBC.

III-B-5. *Experiments Involving More Than 10 Liters of Culture.* The appropriate containment will be decided by the IBC. Where appropriate, the large-scale containment recommendations of the NIH should be used (45 FR 24968).

III-C. *Experiments That Require IBC Notice Simultaneously With Initiation of Experiments.* Experiments not included in Sections III-A, III-B, III-D, and subsections of these Sections are to be considered in Section III-C. All such experiments can be carried out at P1 containment. For experiments in this category, a registration document as described in Section III-B must be dated and signed by the investigator and filed with the local IBC. The IBC shall review all such proposals, but IBC review prior to initiation of the experiment is not required.

For example, experiments in which all components derive from non-pathogenic prokaryotes and non-pathogenic lower eukaryotes fall under Section III-C and can be carried out at P1 containment.

Caution: Experiments Involving Formation of Recombinant DNA Molecules Containing no more Than Two-Thirds of the Genome of any Eukaryotic Virus. Recombinant DNA molecules containing no more than two-thirds of the genome of any eukaryotic virus (all viruses from a single Family [36] being considered identical [50] may be propagated and maintained in cells in tissue culture using P1 containment. For such experiments, it must be shown that the cells lack helper virus for the specific Families of defective viruses being used. If helper virus is present, procedures specified under Section III-B-3 should be used. The DNA may contain fragments of the genome of viruses from more than one Family but each fragment must be less than two-thirds of a genome.

III-D. *Exempt Experiments.* The following recombinant DNA moleculses are exempt from these Guidelines and no registration with the IBC is necessary.

III-D-1. Those that are not in organisms or viruses.

III-D-2. Those that consist entirely of DNA segments from a single nonchromosomal or viral DNA source,

though one or more of the segments may be a synthetic equivalent.

III-D-3. Those that consist entirely of DNA from a prokayotic host, including its indigenous plasmids or viruses, when propagated only in that host (or a closely related strain of the same species) or when transferred to another host by well established physiological means; also, those that consist entirely of DNA from an eukaryotic host, including its chloroplasts, mitochondria, or plasmids (but excluding viruses), when propagated only in that host (or a closely related strain of same species).

III-D-4. Certain specified recombinant DNA molecules that consist entirely of DNA segments from different species that exchange DNA by known physiological processes, though one or more of the segments may be a synthetic equivalent. A list of such exchangers will be prepared and periodically revised by the Director, NIH, with advice of the RAC, after appropriate notice and opportunity for public comment. (See Section IV-E-1-b-(1)-(d).) Certain classes are exempt as of publication of these Revised Guildelines. The list is in Appendix A. An updated list may be obtained from the Office of Recombinant DNA Activities, National Institutes of Health, Bethesda, Maryland 20205.

III-D-5. Other classes of recombinant DNA Molecules, if the Director, NIH, with advice of the RAC, after appropriate notice and opportunity for public comment, finds that they do not present a significant risk to health or the environment. (See Section IV-E-1-b-(1)-(d).) Certain classes are exempt as of publication of these Revised Guidelines. The list is in Appendix C. An updated list may be obtained from the Office of Recombinant DNA Activities, National Institutes of Health, Bethesda, Maryland 20205.

IV. Roles and Responsibilities

IV-A. *Policy.* Safety in activities involving recombinant DNA depends on the individual conducting them. The Guidelines cannot anticipate every possible situation. Motivation and good judgment are the key essentials to protection of health and the environment.

The Guidelines are intended to help the Institution, the Institutional Biosafety Committee (IBC), the Biological Safety Officer, and the Principal Investigator determine the safeguards that should be implemented. These Guidelines will never be complete or final, since all conceivable experiments involving recombinant DNA cannot be foreseen. Therfore, it is the responsibility of the Institution and those associated with it to adhere to the *purpose* of the Guidelines as well as to their specifics.

Each Institution (and the IBC acting on its behalf) is responsible for ensuring that recombinant DNA activities comply with the Guidelines. General recognition of institutional authority and responsibility properly establishes accountability for safe conduct of the research at the local level.

The following roles and responsibilities constitute an administrative framework in which safety is an essential and integral part of research involving recombinant DNA molecules. Further clarifications and interpretations of roles and responsibilities will be issued by NIH as necessary.

IV-B. *General Applicability.* The Guidelines are applicable to all recombinant DNA research within the United States or its territories which is conducted at or sponsored by an Institution that receives any support for recombinant DNA research from NIH. This includes research performed by NIH directly.

An individual receiving support for research involving recombinant DNA must be associated with or sponsored by an Institution that can and does assume the responsibilities assigned in these Guidelines.

The Guidelines are also applicable to projects done abroad if they are supported by NIH funds. If the host country however, has established rules for the conduct of recombinant DNA projects, then a certificate of compliance with those rules may be submitted to NIH in lieu of compliance with the NIH Guidelines. NIH reserves the right to withhold funding if the safety practices to be employed abroad are not reasonably consistent with the NIH Guidelines.

IV-C. *General Definitions.* The following terms, which are used throughout the Guidelines, are defined as follows:

IV-C-1. "DNA" means deoxyribonucleic acid.

IV-C-2. "Recombinant DNA" or "recombinant DNA molecules" means either (i) molecules which are constructed outside living cells by joining natural or synthetic DNA segments to DNA molecules that can replicate in a living cell, or (ii) DNA molecules which result from the replication of a molecule described in (i) above.

IV-C-3. "Institution" means any public or private entity (including Federal, State, and local government agencies).

IV-C-4. " institutional Biosafety Committee" or "IBC" means a committee that (i) meets the requirements for membership specified in Section IV-D-2, and (ii) reviews, approves, and oversees projects in accordance with the responsibilities defined in Sections IV-D-2 and -3.

IV-C-5. "NIH Office of Recombinant DNA Activities" or "ORDA" means the office within NIH with responsibility for (i) reviewing and coordinating all activities of NIH related to the Guidelines, and (ii) performing other duties as defined in Section IV-E-3.

IV-C-6. "Recombinant DNA Advisory Committee" or "RAC" means the public advisory committee that advises the Secretary, the Assistant Secretary for Health, and the Director of the National Institutes of Health concerning recombinant DNA research. The RAC shall be constituted as specific in Section IV-E-2.

IV-C-7. "Director, NIH" or "Director" means the Director of the National Institutes of Health and any other officer or employee of NIH to whom authority has been delegated.

IV-C-8. "Federal Interagency Advisory Committee on Recombinant DNA Research" means the committee established in October 1976 to advise the Secretary, HHS, the Assistant Secretary for Health, and the Director, NIH, on the coordination of those aspects of all Federal programs and activities which relate to recombinant DNA research.

IV-C-9. "Laboratory Safety Monograph" or "LSM" means a publication describing practices, equipment, and facilities in detail.

IV-D. *Responsibilities of the Institution.*

IV-D-1. Each Institution conducting or sponsoring recombinant DNA research covered by these Guidelines is responsible for ensuring that the research is carried out in full conformity with the provisions of the Guidelines. In order to fulfill this resonsibility, the Institution shall:

IV-D-1-a. Establish and implement policies that provide for the safe conduct of recombinant DNA research and that ensure compliance with the Guidelines. The Institution, as part of its general responsibilities for implementing the Guidelines, may establish additional procedures, as deemed necessary, to govern the Institution and its components in the discharge of its responsibilities under the Guidelines. This may include (i) statements formulated by the Institution for general implementation of the Guidelines and (ii) whatever additional precautionary

steps the Institution may deem appropriate.

IV–D–1–b. Establish an Institutional Biosafety Committee (IBC) that meets the requirements set forth in Section IV–D–2 and carries out the functions detailed in Section IV–D–3.

IV–D–1–c. If the Institution is engaged in recombinant DNA research at the P3 or P4 containment level, appoint a Biological Safety Officer (BSO), who shall be a member of the IBC and carry out the duties specified in Section IV–D–4.

IV–D–1–d. Require that investigators responsible for research covered by these Guidelines comply with the provisions of Section IV–D–5, and assist investigators to do so.

IV–D–1–e. Ensure appropriate training for the IBC chairperson and members, the BSO, Principal Investigators (PIs), and laboratory staff regarding the Guidelines, their implementation, and laboratory safety. Responsibility for training IBC members may be carried out through the IBC chairperson. Responsibility for training laboratory staff may be carried out through the PI. The Institution is responsible for seeing that the PI has sufficient training, but may delegate this responsibility to the IBC.

IV–D–1–f. Determine the necessity, in connection with each project, for health surveillance of recombinant DNA research personnel, and conduct, if found appropriate, a health surveillance program for the project. [The Laboratory Safety Monograph (LSM) discusses various possible components of such a program—for example, records of agents handled, active investigation of relevant illnesses, and the maintenance of serial serum samples for monitoring serologic changes that may result from the employees' work experience. Certain medical conditions may place a laboratory worker at increased risk in any endeavor where infectious agents are handled. Examples given in the LSM include gastrointestinal disorders and treatment with steroids, immunosuppressive drugs, or antibiotics. Workers with such disorders or treatment should be evaluated to determine whether they should be engaged in research with potentially hazardous organisms during their treatment or illness.]

IV–D–1–g. Report within 30 days to ORDA any significant problems with and violations of the Guidelines and significant research-related accidents and illnesses, unless the Institution determines that the PI or IBC has done so.

IV–D–2. *Membership and Procedures of the IBC.* The Institution shall establish an Institutional Biosafety Committee (IBC) meeting the following requirements:

IV–D–2–a. The IBC shall comprise no fewer than five members so selected that they collectively have experience and expertise in recombinant DNA technology and the capability to assess the safety of recombinant DNA research experiments and any potential risk to public health or the environment. At least two members (but not less than 20 percent of the membership of the committee) shall not be affiliated with the Institution (apart from their membership on the IBC) and shall represent the interest of the surrounding community with respect to health and protection of the environment. Members meet this requirement if, for example, they are officials of State or local public health or environmental protection agencies, members of other local governmental bodies, or persons active in medical, occupational health, or environmental concerns in the community. The Biological Safety Officer (BSO) mandatory when research is being conducted at the P3 and P4 levels, shall be a member (see Section IV–D–4).

IV–D–2–b. In order to ensure the professional competence necessary to review recombinant DNA activities, it is recommended that (i) the IBC include persons from disciplines relevant to recombinant DNA technology, biological safety, and engineering; (ii) the IBC include, or have available as consultants, persons knowledgeable in institutional commitments and policies, applicable law, standards of professional conduct and practice, community attitudes, and the environment; and (iii) at least one member be a nondoctoral person from a laboratory technical staff.

IV–D–2–c. The Institution shall identify the committee members by name in a report to the NIH Office of Recombinant DNA Activities (ORDA) and shall include relevant background information on each member in such form and at such times as ORDA may require.

IV–D–2–d. No member of an IBC may be involved (except to provide information requestd by the IBC) in the review or approval of a project in which he or she has been, or expects to be, engaged or has a direct financial interest.

IV–D–2–e. The Institution may establish procedures that the IBC will follow in its inital and continuing review of applications, proposals, and activities. (IBC review procedures are specified in Section IV–D–3–a.)

IV–D–2–f. Central to implementation of the Guidelines is the review of experiments by the IBC.

IV–D–2–g. Institutions are encouraged to open IBC meetings to the public whenever possible, consistent with protection of privacy and proprietary interests.

IV–D–2–h. Upon request, the Institution shall make available to the public all minutes of IBC meetings and any documents submitted to or received from funding agencies which the latter are required to make available to the public (e.g., reports of Guideline violations and significant research-related accidents, and agency directives to modify projects). If comments are made by members of the public on IBC actions, the Institution shall forward to NIH both the comments and the IBC's response.

IV–D–3. *Functions of the IBC.* On behalf of the Institution, the IBC is responsible for:

IV–D–3–a. Reviewing for compliance with the NIH Guidelines recombinant DNA research as specified in Part III conducted at or sponsored by the Institution, and approving those research projects that it finds are in conformity with the Guidelines. This review shall include:

IV–D–3–a–(1). An independent assessment of the containment levels required by these Guidelines for the proposed research, and

IV–D–3–a–(2). An assessment of the facilities, procedures, and practices, and of the training and expertise of recombinant DNA personnel.

Note.—See Laboratory Safety Monograph (pages 187–190) for suggested guidance in conducting this review.

IV–D–3–b. Notifying the Principal Investigator (PI) of the results of their review.

IV–D–3–c. Reviewing periodically recombinant DNA research being conducted at the Institution, to ensure that the requirements of the Guidelines are being fulfilled.

IV–D–3–d. Adopting emergency plans covering accidental spills and personnel contamination resulting from such research.

Note.—Basic elements in developing specific procedures for dealing with major spills of potentially hazardous materials in the laboratory are detailed in the Laboratory Safety Monograph. Included are information and references on decontamination and emergency plans. NIH and the Centers for Disease Control are available to provide consultation, and direct assistance if necessary, as posted in the LSM. The Institution shall cooperate with the State and local public health departments, reporting

any significant research-related illness or accident that appears to be a hazard to the public health.

IV-D-3-e. Reporting within 30 days to the appropriate institutional official and to the NIH Office of Recombinant DNA Activities (ORDA) any significant problems with or violations of the Guidelines, and any significant research-related accidents or illnesses, unless the IBC determines that the PI has done so.

IV-D-3-f. The IBC may not authorize initiation of experiments not explicitly covered by the Guidelines until NIH (with the advice of the RAC when required) establishes the containment requirement.

IV-D-3-g. Performing such other functions as may be delegated to the IBC under Section IV-D-1.

IV-D-4. *Biological Safety Officer.* The Institution shall appoint a BSO if it engages in recombinant DNA research at the P3 or P4 containment level. The officer shall be a member of the Institutional Biosafety Committee (IBC), and his or her duties shall include (but need not be limited to):

IV-D-4-a. Ensuring through periodic inspections that laboratory standards are rigorously followed;

IV-D-4-b. Reporting to the IBC and the Institution all significant problems with and violations of the Guidelines and all significant research-related accidents and illinesses of which the BSO becomes aware, unless the BSO determines that the Principal Investigator (PI) has done so;

IV-D-4-c. Developing emergency plans for dealing with accidental spills and personnel contamination, and investigating recombinant DNA research laboratory accidents;

IV-D-4-d. Providing advice on laboratory security;

IV-D-4-e. Providing technical advice to the PI and the IBC on research safety procedures.

Note.—See Laboratory Safety Monograph for additional information on the duties of the BSO.

IV-D-5. *Principal Investigator.* On behalf of the Institution, the PI is responsible for complying fully with the Guidelines in conducting any recombinant DNA research.

IV-D-5-a. *PI—General.* As part of this general responsibility, the PI shall:

IV-D-5-a-(1). Initiate or modify no recombinant DNA research requiring approval by the IBC prior to initiation (see Sections III-A and III-B) until that research, or the proposed modification thereof, has been approved by the IBC and has met all other requirements of the Guidelines;

IV-D-5-a-(2). Report within 30 days to the IBC and NIH (ORDA) all significant problems with and violations of the Guidelines and all significant research-related accidents and illnesses;

IV-D-5-a-(3). Report to the IBC and to NIH (ORDA) new information bearing on the Guidelines;

IV-D-5-a-(4). Be adequately trained in good microbiological techniques;

IV-D-5-a-(5). Adhere to IBC-approved emergency plans for dealing with accidential spills and personnel contamination; and

IV-D-5-a-(6). Comply with shipping requirements for recombinant DNA molecules. (See Section II-C for shipping requirements and Laboratory Safety Monograph for technical recommendations.)

IV-D-5-b. *Submissions by the PI to NIH.* The PI shall:

IV-D-5-b-(1). Submit information to NIH (ORDA) in order to have new host-vector systems certified;

IV-D-5-b-(2). Petition NIH, with notice to the IBC, for exemptions to these Guidelines;

IV-D-5-b-(3). Petition NIH, with concurrence of the IBC, for approval to conduct experiments specified in Section III-A of the Guidelines;

IV-D-5-b-(4). Petition NIH for determination of containment for experiments requiring case-by-case review;

IV-D-5-b-(5). Petition NIH for determination of containment for experiments not covered by the Guidelines.

IV-D-5-c. *Submissions by the PI to the IBC.* The PI shall:

IV-D-5-c-(1). Make the initial determination of the required levels of physical and biological containment in accordance with the Guidelines;

IV-D-5-c-(2). Select appropriate microbiological practices and laboratory techniques to be used in the research;

IV-D-5-c-(3). Submit the initial research protocol if covered under Guidelines Sections III-A, III-B, or III-C (and also subsequent changes—e.g., changes in the source of DNA or host-vector system) to the IBC for review and approval or disapproval; and

IV-D-5-c-(4). Remain in communication with the IBC throughout the conduct of the project.

IV-D-5-d. *PI Responsibilities Prior to Initiating Research.* The PI is responsible for:

IV-D-5-d-(1). Making available to the laboratory staff copies of the protocols that describe the potential biohazards and the precautions to be taken;

IV-D-5-d-(2). Instructing and training staff in the practices and techniques required to ensure safety and in the procedures for dealing with accidents; and

IV-D-5-d-(3). Informing the staff of the reasons and provisions for any precautionary medical practices advised or requested, such as vaccinations or serum collection.

IV-D-5-e. *PI Responsibilities During the Condust of the Research.* The PI is responsible for:

IV-D-5-e-(1). Supervising the safety performance of the staff to ensure that the required safety practices and techniques are employed;

IV-D-5-e-(2). Investigating and reporting in writing to ORDA, the Biological Safety Officer (where applicable), and the IBC any significant problems pertaining to the operation and implementation of containment practices and procedures;

IV-D-5-e-(3). Correcting work errors and conditions that may result in the release of recombinant DNA materials;

IV-D-5-e-(4). Ensuring the integrity of the physical containment (e.g., biological safety cabinets) and the biological containment (e.g., purity, and genotypic and phenotypic characteristics); and

IV-D-5-e-(5). *Publications.* PIs are urged to include, in all publications reporting on recombinant DNA research, a description of the physical and biological containment procedures employed.

IV-E. *Responsibilities of NIH.*

IV-E-1. *Director.* The Director, NIH, is responsible for (i) establishing the NIH Guidelines for Research Involving Recombinant DNA Molecules, (ii) overseeing their implementation, and (iii) their final interpretation.

The Director has a number of responsibilities under the Guidelines that involve the NIH Office of Recombinant DNA Activities (ORDA) and the Recombinant DNA Advisory Committee (RAC). ORDA's responsibilities under the Guidelines are administrative. Advice from the RAC is primarily scientific and technical. In certain circumstances, there is specific opportunity for public comment, with published response, before final action.

IV-E-1-a. *General Responsibilities of the Director, NIH.* The responsibilities of the Director shall include the following:

IV-E-1-a-(1). Promulgating requirements as necessary to implement the Guidelines;

IV-E-1-a-(2). Establishing and maintaining the RAC to carry out the responsibilities set forth in Section IV-E-2. The RAC's membership is specified in its charter and in Section IV-E-2;

IV-E-1-a-(3). Establishing and maintaining ORDA to carry out the

responsibilities defined in Section IV-E-3; and

IV-E-1-a-(4). Maintaining the Federal Interagency Advisory Committee on Recombinant DNA Research established by the Secretary, HEW, for advice on the coordination of all Federal programs and activities relating to recombinant DNA, including activities of the RAC.

IV-E-1-b. *Specific Responsibilities of the Directors, NIH.* In carrying out the responsibilities set forth in this Section, the Director shall weigh each proposed action, through appropriate analysis and consultation, to determine that is complies with the Guidelines and presents no significant risk to health or the environment.

IV-E-1-b-(1). *The Director is responsible for the following major actions.* (For these, the Director must seek the advice of the RAC and provide an opportunity for public and Federal agency comment. Specifically, the agenda of the RAC meeting citing the major actions will be published in the **Federal Register** at least 30 days before the meeting, and the Director will also publish the proposed actions in the **Federal Register** for comment at least 30 days before the meeting. In addition, the Director's proposed decision, at his discretion, may be published in the **Federal Register** for 30 days of comment before final action is taken. The Director's final decisions, along with response to the comments, will be published in the **Federal Register** and the *Recombinant DNA Technical Bulletin.* The RAC and IBC chairpersons will be notified of this decision):

IV-E-1-b-(1)-(a). Changing containment levels for types of experiments that are specified in the Guidelines when a major action is involved;

IV-E-1-b-(1)-(b). Assigning containment levels for types of experiments that are not explicity considered in the Guidelines when a major action is involved;

IV-E-1-b-(1)-(c). Certifying new host-vector systems, with the exception of minor modifications of already certified systems. (The standards and procedures for certification are described in Section II-D-2-a. Minor modifications constitute, for example, those of minimal or no consequence to the properties relevant to containment);

IV-E-1-b-(1)-(d). Promulgating and amending a list of classes of recombinant DNA molecules to be exempt from these Guidelines because they constist entirely of DNA segments from species that exchange DNA by known physiological processes, or otherwise do not present a significant risk to health or the environment;

IV-E-1-b-(1)-(e). Permitting experiments specified by Section III-A of the Guidelines; and

IV-E-1-b-(1)-(f). Adopting other changes in the Guidelines.

IV-E-1-b-(2). *The Director is also responsibile for the following lesser actions* (For these, the Director must seek the advice of the RAC. The Director's decision will be transmitted to the RAC and IBC chairpersons and published in the Recombinant DNA Technical Bulletin);

IV-E-1-b-(2)-(b). *The Director is also responsible for the following lesser actions* (For these, the Director must seek the advice of the RAC. The Director's decision will be transmitted to the RAC and IBC chairpersons and published in the Recombinant DNA Technical Bulletin):

IV-E-1-b-(2)-(a). Interpreting and determining containment levels, upon request by ORDA;

IV-E-1-b-(2)-(b). Changing containment levels for experiments that are specified in the Guidelines (see Section III);

IV-E-1-b-(2)-(c). Assigning containment levels for experiments not explicity considered in the Guidelines;

IV-E-1-b-(2)-(d). Designating certain class 2 agents as class 1 for the purpose of these Guidelines (see Footnote 1 and Appendix B);

IV-E-1-b-(3). *The Director is also responsibile for the following actions.* (The Director's decision will be transmitted to the RAC and IBC chairpersons and published in the *Recombinant DNA Technical Bulletin):*

IV-E-1-b-(3)-(a). Interpreting the Guidelines for experiments to which the Guidelines specifically assign containment levels;

IV-E-1-b-(3)-(b). Determining appropriate containment conditions for experiments according to case precedents developed under Section IV-E-1-b-(2)-(c).

IV-E-1-b-(3)-(c). Approving minor modifications of already certified host-vector systems. (The standards and procedures for such modifications are described in Section II-D-2);

IV-E-1-b-(3)-(d). Decertifying already certified host-vector systems;

IV-E-1-b-(3)-(e). Adding new entries to the list of toxins for vertebrates (see Appendix F); and

IV-E-1-b-(3)-(f). Approving the cloning of toxin genes in host-vector systems other than *E. coli* K-12 (see Appendix F).

IV-E-1-b-(4). The Director shall conduct, support, and assist training programs in laboratory safety for Institutional Biosafety Committee members, Biological Safety Officers, Principal Investigators, and laboratory staff.

IV-E-2. *Recombinant DNA Advisory Committee.* The NIH Recombinant DNA Advisory Committee (RAC) is responsible for carrying out specified functions cited below as well as others assigned under its charter or by the Secretary, HHS, the Assistant Secretary for Health, and the Director, NIH.

The members of the committee shall be chosen to provide, collectively, expertise in scientific fields relevant to recombinant DNA technology and biological safety—e.g., microbiology, molecular biology, virology, genetics, epidemiology, infectious diseases, the biology of enteric organisms, botany, plant pathology, ecology, and tissue culture. At least 20 percent of the members shall be persons knowledgeable in applicable law, standards of professional conduct and practice, public attitudes, the environment, public health, occupational health, or related fields. Representatives from Federal agencies shall serve as nonvoting members. Nominations for the RAC may be submitted to the NIH Office of Recombinant DNA Activities, Bethesda, Md. 20205.

All meetings of the RAC will be announced in the **Federal Register**, including tentative agenda items, 30 days in advance of the meeting, with final agendas (if modified) available at least 72 hours before the meeting. No item defined as a major action under Section IV-E-1-b-(1) may be added to an agenda after it appears in the **Federal Register.**

The RAC shall be responsible for advising the Director, NIH, on the actions listed in Section IV-E-1-b-(1) and -(2).

IV-E-3. *The Office of Recombinant DNA Activities.* ORDA shall serve as a focal point for information on recombinant DNA activities and provide advice to all within and outside NIH, including Institutions, Biological Safety Committees, Principal Investigators, Federal agencies, State and local governments, and institutions in the private sector. ORDA shall carry out such other functions as may be delegated to it by the Director, NIH, including those authorities described in Section IV-E-1-b-(3). In addition, ORDA shall be responsible for the following:

IV-E-3-a. Reviewing and approving Institutional Biosafety Committee (IBC) membership;

IV-E-3-b. Publishing in the **Federal Register:**

IV-E-3-b-(1). Announcements of Recombinant DNA Advisory Committee

(RAC) meetings and agendas at least 30 days in advance;

Note.—If the agenda for an RAC meeting is modified, ORDA shall make the revised agenda available to anyone, upon request, at least 72 hours in advance of the meeting.

IV-E-3-b-(2). Proposed major actions of the type falling under Section IV-E-1-b-(1) at least 30 days prior to the RAC meeting at which they will be considered; and

IV-E-3-b-(3). The NIH Director's final decision on recommendations made by the RAC.

IV-E-3-c. Publishing the *Recombinant DNA Technical Bulletin;* and

IV-E-3-d. Serving as executive secretary of the RAC.

IV-E-4. *Other NIH Components.* Other NIH components shall be responsible for:

IV-E-4-a. Certifying P4 facilities, inspecting them periodically, and inspecting other recombinant DNA facilities as deemed necessary; and

IV-E-4-b. Announcing and distributing certified HV2 host-vector systems (see Section II-D-3).

IV-F. *Compliance.* As a condition for NIH funding of recombinant DNA research, Institutions must ensure that such research conducted at or sponsored by the Institution, irrespective of the source of funding, shall comply with these Guidelines. The policies on noncompliance are as follows:

IV-F-1. All NIH-funded projects involving recombinant DNA techniques must comply with the NIH Guidelines. Noncompliance may result in (i) suspension, limitation, or termination of financial assistance for such projects and of NIH funds for other recombinant DNA research at the Institution, or (ii) a requirement for prior NIH approval of any or all recombinant DNA projects at the Institution.

IV-F-2. All non-NIH funded projects involving recombinant DNA techniques conducted at or sponsored by an Institution that receives NIH funds for projects involving such techniques must comply with the NIH Guidelines. Noncompliance may result in (i) suspension, limitation, or termination of NIH funds for recombinant DNA research at the Institution, or (ii) a requirement for prior NIH approval of any or all recombinant DNA projects at the Institution.

IV-F-3. Information concerning noncompliance with the Guildelines may be brought forward by any person. It should be delivered to both NIH (ORDA) and the relevant Institution. The Institution, generally through the IBC, shall take appropriate action. The Institution shall forward a complete report of the incident to ORDA, recommending any further action indicated.

IV-F-4. In cases where NIH proposes to suspend, limit, or terminate financial assistance because of noncompliance with the Guidelines, applicable DHEW and Public Health Service Procedures shall govern.

IV-F-5. *Voluntary Compliance.* Any individual, corporation, or institution that is not otherwise covered by the Guidelines is encouraged to conduct recombinant DNA research activities in accordance with the Guidelines, through the procedures set forth in Part VI.

V. Footnotes and References

1. The reference to organisms as Class 1, 2, 3, 4, or 5 refers to the classification in the publication *Classification of Etiologic Agents on the Basis of Hazard,* 4th Edition, July 1974; U.S. Department of Health, Education, and Welfare, Public Health Service, Centers for Disease Control, Office of Biosafety, Atlanta, Georgia 30333. The list of organisms in each class, as given in this publication, is reprinted in Appendix B to these Guidelines.

The Director, NIH, with advice of the Recombinant DNA Advisory Committee, may designate certain of the agents which are listed as Class 2 in the *Classification of Etiologic Agents on the Basis of Hazard,* 4th Edition, July 1974, as Class 1 agents for the Purposes of these Guidelines (see Section IV-E-1-b-(2)-(d)). An updated list of such agents may be obtained from the Office of Recombinant DNA Activities (ORDA), National Institutes of Health, Bethesda, Maryland 20205.

The entire *Classification of Etiologic Agents on the Basis of Hazard* is in the process of revision.

2A. In Part III of the Guidelines, there are a number of places where judgments are to be made. In all these cases the principal investigator is to make the judgment on these matters as part on his responsibility to "make the initial determination of the required levels of physical and biological containment in accordance with the Guidelines" (Section IV-D-5-c-(1)). In the cases falling under Sections III-A, -B or -C, this judgment is to be reviewed and approved by the Institutional Biosafety Committee as part of its responsibility to make "an independent assessment of the containment levels required by these Guidelines for the proposed research" (Section IV-D-3-a-(1)). If the IBC wishes, any specific cases may be referred to the NIH Office of Recombinant DNA Activities as part of ORDA's functions to "provide advice to all within and outside NIH" (Section IV-E-3), and ORDA may request advice from the Recombinant DNA Advisory Committee as part of the RAC's responsibility for "interpreting and determining containment levels upon request by ORDA" (Section IV-E-1-b-(2)-(a)).

6. *Laboratory Safety at the Center for Disease Control* (Sept. 1974). U.S. Department of Health, Education and Welfare Publication No. CDC 75-8118.

7. *Classification of Etiologic Agents on the Basis of Hazard.* (4th Edition, July 1974). U.S. Department of Health, Education and Welfare. Public Health Service. Centers for Disease Control, Office of Biosafety, Atlanta, Georgia 30333.

8. *National Cancer Institute Safety Standards for Research Involving Oncogenic Viruses* (Oct. 1974). U.S. Department of Health, Education and Welfare Publication No. (NIH) 75-790.

9. *National Institutes of Health Biohazards Safety Guide* (1974). U.S. Department of Health, Education, and Welfare, Public Health.

10. *Biohazards in Biological Research* (1973). A. Hellman, M. N. Oxman, and R. Pollack (ed.) Cold Spring Harbor Laboratory.

11. *Handbook of Laboratory Safety* (1971). Second Edition. N. V. Steere (ed.). The Chemical Rubber Co., Cleveland.

12. Bodily, J. L. (1970). *General Administration of the Laboratory,* H. L. Bodily, E. L. Updyke, and J. O. Mason (eds.), Diagnostic Procedures for Bacterial, Mycotic and Parasitic Infections. American Public Health Association, New York, pp. 11-28.

13. Darlow, H. M. (1969). *Safety in the Microbiological Laboratory.* In J. R. Norris and D. W. Robbins (ed.), Methods in Microbiology. Academic Press, Inc. New York. pp. 169-204.

14. *The Prevention of Laboratory Acquired Infection* (1974). C. H. Collins, E. G. Hartley, and R. Pilsworth. Public Health Laboratory Service, Monograph Series No. 6.

15. Chatigny, M. A. (1961). *Protection Against Infection in the Microbiological Laboratory: Devices and Procedures.* In W. W. Umbreit (ed.). Advances in Applied Microbiology. Academic Press, New York, N.Y. 3:131-192.

16. *Design Criteria for Viral Oncology Research Facilities* (1975). U.S. Department of Health, Education and Welfare, Public Health Service, National Institutes of Health, DHEW Publication No. (NIH) 75-891.

17. Kuehne, R. W. (1973). *Biological Containment Facility for Studying Infectious Disease.* Appl. Microbiol. 26-239-243.

18. Runkle, R. S., and G. B. Phillips (1969). *Microbial Containment Control Facilities.* Van Nostrand Reinhold, New York.

19. Chatigny, M. A., and D. I. Clinger (1969). *Contamination Control in Aerobiology.* In R. L. Dimmick and A. B. Akers (eds.). An Introduction to Experimental Aerobiology. John Wiley & Sons, New York, pp. 194-263.

19A. Horsfall, F. L., Jr., and J. H. Baner (1940). *Individual Isolation of Infected Animals in a Single Room.* J. Bact. *40,* 569-580.

20. Biological safety cabinets referred to in this section are classified as *Class I, Class II,* or *Class III* cabinets. A *Class I* is a ventilated cabinet for personnel protection having an inward flow of air away from the operator. The exhaust air from this cabinet is filtered through a high-efficiency particulate air (HEPA) filter. This cabinet is used in three operational modes: (1) with a full-width open front, (2) with an installed front closure panel (having four 8-inch diameter openings) without gloves, and (3) with an installed front closure panel equipped with arm-length

rubber gloves. The face velocity of the inward flow of air through the full-width open front is 75 feet per minute or greater. A *Class II* cabinet is a ventilated cabinet for personnel and product protection having an open front with inward air flow for personnel protection, and HEPA filtered mass recirculated air flow for product protection. The cabinet exhaust air is filtered through an HEPA filter. The face velocity of the inward flow of air through the full-width open front is 75 feet per minute or greater. Design and performance specifications for *Class II* cabinets have been adopted by the National Sanitation Foundation, Ann Arbor, Michigan. A *Class III* cabinet is a closed-front ventilated cabinet of gas-tight construction which provides the highest level of personnel protection of all biohazard safety cabinets. The interior of the cabinet is protected from contaminants exterior to the cabinet. The cabinet is fitted with arm-length rubber gloves and is operated under a negative pressure of at least 0.5 inch water gauge. All supply air is filtered through HEPA filters. Exhaust air is filtered through two HEPA filters or one HEPA filter and incinerator before being discharged to the outside environment.

21. Hershfield, V., H. W. Boyer, C. Yanofsky, M. A. Lovett, and D. R. Helinski (1974). *Plasmid ColEl as a Molecular Vehicle for Cloning and Amplification of DNA.* Proc. Nat. Acad. Sci. USA *71*, 3455–3459.

22. Wensink, P. C., D. J. Finnegan, J. E. Donelson, and D. S. Hogness(1974). *A System for Mapping DNA Sequences in the Chromosomes of Drosophila Melanogaster.* Cell *3*, 315–335.

23. Tanaka, T., and B. Weisblum (1975). *Construction of a Colicin El-R Factor Composite Plasmid In Vitro: Means for Amplification of Deoxyribonucleic Acid.* J. Bacteriol. *121*, 354–362.

24. Armstrong, K. A., V. Hershfield, and D. R. Helinski (1977). *Gene Cloning and Containment Properties of Plasmid Col El and Its Derivatives,* Science *196*, 172–174.

25. Bolivar, F., R. L. Rodriguez, M. C. Bethlach, and H. W. Boyer(1977). *Construction and Characterization of New Cloning Vehicles: I. Ampicillin-Resistant Derivative of pMB9.* Gene *2*, 75–93.

26. Cohen, S. N., A. C. W. Chang, H. Boyer, and R. Helling (1973). *Construction of Biologically Functional Bacterial Plasmids in Vitro.* Proc. Natl. Acad. Sci. USA *70*, 3240–3244.

27. Bolivar, F., R. L. Rodriguez, R. J. Greene, M. C. Batlach, H. L. Reyneker, H. W. Boyer, J. H. Crosa, and S. Falkow(1977). *Construction and Characterization of New Cloning Vehicles: II. A Multi-Purpose Cloning System.* Gene *2*, 95–113.

28. Thomas, M., J. R. Cameron, and R. W. Davis (1974). *Viable Molecular Hydrids of Bacteriophage Lambda and Eukaryotic DNA.* Proc. Nat. Acad. Sci. USA *71*, 4579–4583.

29. Murray, N. E., and K. Murray (1974). *Manipulation of Restriction Targets in Phage Lambda to Form Receptor Chromosomes for DNA Fragments.* Nature *251*, 476–481.

30. Rambach, A., and P. Tiollais (1974). *Bacteriophage Having EcoRI Endonuclease Sites Only in the Non-Essential Region of the Genome.* Proc. Nat. Acad. Sci., USA *71*, 3927–3930.

31. Blattner, F. R., B. G. Williams, A. E. Bleche, K. Denniston-Thompson, H. E. Faber, L. A. Furlong, D. J. Gunwald, D. O. Kiefer, D. D. Moore, J. W. Shumm, E. L. Sheldon, and O. Smithies (1977). *Charon Phages: Safer Derivatives of Bacteriophage Lambda for DNA Cloning.* Science *196*, 163–169.

32. Donoghue, D. J., and P. A. Sharp (1977). *An Improved Lambda Vector: Construction of Model Recombinants Coding for Kanamycin Resistance,* Gene *1*, 209–227.

33. Leder, P., D. Tiemeier and L. Enquist (1977). *EK2 Derivatives of Bacteriophage Lambda Useful in the Cloning of DNA from Higher Organisms: The λgt WES System.* Science *196*, 175–177.

33A. Skalka, A. (1978). *Current Status of Coliphage λ EK2 Vectors.* Gene *3*, 29–35.

33B. Szybalski, W., A. Skalka, S. Gottesman, A. Campbell, and D. Botstein (1978). *Standardized Laboratory Tests for EK2 Certification.* Gene *3*, 36–38.

35. Defined as observable under optimal laboratory conditions by transformation, transduction, phage infection, and/or conjugation with transfer of phage, plasmid, and/or chromosomal genetic information. Note that this definition of exchange may be less stringent than that applied to exempt organisms under Section III–D–4.

36. As classified in the Third Report of the International Committee on Taxonomy of Viruses: Classification and Nomenclature of Viruses, R. E. F. Matthews, Ed. Intervirology 12 (129–296) 1979.

48. A USDA permit, required for import and interstate transport of pathogens, may be obtained from the Animal and Plant Health Inspecton Service, USDA, Federal Building, Hyattsville, MD 20782.

49. A subset of non-conjugative plasmid vectors are also poorly mobilizable (e.g., pBR 322, pBR313). Where practical, these vectors should be employed.

50. I.e., the total of all genomes within a Family shall not exceed two-thirds of the genome.

51. All activities, inlcuding storage of variloa and whitepox are restricted to the single national facility (World Health Organization (WHO) Collaborating Center for Smallpox Research, Centers for Disease Control, in Atlanta).

VI. Voluntary Compliance

VI–A. *Basic Policy.* Individuals, corporations, and institutions not otherwise covered by the Guidelines are encouraged to do so by following the standards and procedures set forth in Parts I–IV of the Guidelines. In order to simplify discussion, references hereafter to "institutions" are intended to encompass corporations, and individuals who have no organizational affiliation. For purposes of complying with the Guidelines, and individual intending to carry out research involving recombinant DNA is encouraged to affiliate with an institution that has an Institutional Biosafety Committee approved under the Guidelines.

Since commercial organizations have special concerns, such as protection of proprietary data, some modifications and explanations of the procedures in Parts I–IV are provided below, in order to address these concerns.

VI–B. *IBC Approval.* The NIH Office of Recombinant DNA Activities (ORDA) will review the membership of an institution's Institutional Biosafety Committee (IBC) and, where it finds the IBC meets the requirements set forth in Section IV–D–2, will give its approval to the IBC membership.

It should be emphasized that employment of an IBC member solely for purposes of membership on the IBC does not itself make the member an institutionally affiliated member for purposes of Section IV–D–2–a.

Except for the unaffiliated members, a member of an IBC for an institution not otherwise covered by the Guidelines may participate in the review and approval of a project in which the member has a direct financial interest, so long as the member has not been and does not expect to be engaged in the project. Section IV–D–2–d is modified to that extent for purposes of these institutions.

VI–C. *Certification of Host-Vector Systems.* A host-vector system may be proposed for certification by the Director, NIH, in accordance with the procedures set forth in Section II–D–2–a.

Institutions not otherwise covered by the Guidelines will not be subject to Section II–D–3 by complying with these procedures.

In order to ensure protection for proprietary data, any public notice regarding a host-vector system which is designated by the institution as proprietary under Section VI–E–1 will be issued only after consultation with the institution as to the content of the notice.

VI–D. *Requests for Exemptions and Approvals.* Requests for exemptions or other approvals required by the Guidelines should be requested by following the procedures set forth in the appropriate sections in Parts I–IV of the Guidelines.

In order to ensure protection for proprietary data, any public notice regarding a request for an exemption or other approval which is designated by the institution as proprietary under Section VI–E–1 will be issued only after consultation with the institution as to the content of the notice.

VI–E. *Protection of Proprietary Data.* In general, the Freedom of Information Act requires Federal agencies to make their records available to the public upon request. However, this requirement does not apply to, among other things, "trade secrets and commercial and

financial information obtained from a person and privileged or confidential." 18 U.S.C. 1905, in turn makes it a crime for an officer or employee of the United States or any Federal department or agency to publish, divulge, disclose, or make known "in any manner or to any extent not authorized by law any information coming to him in the course of his employment or official duties or by reason of any examination or investigation made by, or return, report or record made to or filed with, such department or agency or officer or employee thereof, which information concerns or relates to the trade secrets, [or processes . . . of any person, firm, partnership, corporation, or association." This provision applies to all employees of the Federal Government, including special Government employees. Members of the Recombinant DNA Advisory Committee are "special Government employees."

VI-E-1. In submitting information to NIH for purposes of complying voluntarily with the Guidelines, an institution may designate those items of information which the institution believes constitute trade secrets or privileged or confidential commerical or financial information.

VI-E-2. If NIH receives a request under the Freedom of Information Act for information so designated, NIH will promptly contact the institution to secure its views as to whether the information (or some portion) should be released.

VI-E-3. If the NIH decides to release this information (or some portion) in response to a Freedom of Information request or otherwise, the institution will be advised; and the actual release will not be made until the expiration of 15 days after the institution is so advised, except to the extent that earlier release, in the judgement of the Director, NIH, is necessary to protect against an imminent hazard to the public or the environment.

VI-E-4. Presubmission Review.

VI-E-4-a. Any institution not otherwise covered by the Guidelines, which is considering submission of data or information voluntarily to NIH, may request presubmission review of the records involved to determine whether, if the records are submitted, NIH will or will not make part or all of the records available upon request under the Freedom of Information Act.

VI-E-4-b. A request for presubmission review should be submitted to ORDA, along with the records involved. These records must be clearly marked as being the property of the institution, on loan to NIH solely for the purpose of making a determination under the Freedom of Information Act. ORDA will then seek a determination from the HHS Freedom of Information Officer, the responsible official under HHS regulations (45 CFR Part 5), as to whether the records involved (or some portion) are or are not available to members of the public under the Freedom of Information Act. Pending such a determination, the records will be kept separate from ORDA files, will be considered records of the institution and not ORDA, and will not be received as part of ORDA files. No copies will be made of the records.

VI-E-4-c. ORDA will inform the institution of the HHS Freedom of Information Officer's determination and follow the institution's instructions as to whether some or all the records involved are to be returned to the institution or to become a part of ORDA files. If the institution instructs ORDA to return the records, no copies or summaries of the records will be made or retained by HHS, NIH, or ORDA.

VI-E-4-d. The HHS Freedom of Information Officer's determination will represent that official's judgement, as of the time of the determination, as to whether the records involved (or some portion) would be exempt from disclosure under the Freedom of Information Act, if at the time of the determination the records were in ORDA files and a request was received from them under the Act.

Appendix A.—Exemptions Under III-D-4

Section III-D-4 states that exemption from these Guidelines are "certain specified recombinant DNA molecules that consist entirely of DNA segments from different species that exchange DNA by known physiological processes, though one or more of the segments may be a synthetic equivalent. A list of such exchangers will be prepared and periodically revised by the Director, NIH, with advice of the RAC, after appropriate notice and opportunity for public comment. (See Section IV-E-1-b-(1)-(d).) Certain classes are exempt as of publication of these Revised Guidelines. The list is in Appendix A."

Under section III-D-4 of these Guidelines are recombinant DNA molecules that are (1) composed entirely of DNA segments from one or more of the organisms within a sublist and (2) to be propagated in any of the organisms within a sublist. (Classification of *Bergey's Manual of Determinative Bacteriology*, eighth edition. R. E. Buchanan and N. E. Gibbons, editors. Williams and Wilkins Company: Baltimore, 1974.)

Sublist A

1. Genus *Escherichia*
2. Genus *Shigella*
3. Genus *Salmonella* (including *Arizona*)
4. Genus *Enterobacter*
5. Genus *Citrobacter* (including *Levinea*)
6. Genus *Klebsiella*
7. Genus *Erwinia*
8. *Pseudomonas aeruginosa, Pseudomonas putida* and *Pseudomonas fluorescens*
9. *Serratia marcescens*
10. *Yersinia enterocolitica*

Sublist B

1. *Bacillus subtilis*
2. *Bacillus licheniformis*
3. *Bacillus pumilus*
4. *Bacillus globigii*
5. *Bacillus niger*
6. *Bacillus nato*
7. *Bacillus amyloliquefaciens*
8. *Bacillus aterrimus*

Sublist C

1. *Streptomyces aureofaciens*
2. *Streptomyces rimosus*
3. *Streptomyces coelicolor*

Sublist D

1. *Streptomyces griseus*
2. *Streptomyces cyaneus*
3. *Streptomyces venezuelae*

Sublist E

One way transfer of *Streptococcus mutans* or *Streptococcus lactis* DNA into *Streptococcus sanguis*.

Sublist F

1. *Streptococcus sanguis*
2. *Streptococcus pneumoniae*
3. *Streptococcus faecalis*
4. *Streptococcus pyogenes*

Appendix B.—Classification of Microorganisms on the Basis of Hazard

I. Classification of Etiologic Agents on the Basis of Hazard (1)

A. Class 1 Agents

All bacterial, parasitic, fungal, viral, rickettsial, and chlamydial agents not included in higher classes.

B. Class 2 Agents

1. Bacterial Agents

Actinobacillus—all species except *A. mallei*, which is in Class 3
Arizona hinshawii—all sterotypes
Bacillus anthracis
Bordetella—all species
Borrelia recurrentis, B. vincenti
Clostridium botulinum,
Cl. chauvoei, Cl. haemolyticum,
Cl. histolyticum, Cl. novyi
Cl. septicum, Cl. tetani
Corynebacterium diptheriae,
C. equi, C. haemolyticum,
C. pseudotuberculosis
C. pyogenes, C. renale
Diplococcus (Streptococcus) pneumoniae
Erysipelothrix insidiosa
Escherichia coli—all enteropathogenic serotypes
Haemophilus ducreyi, H. influenzae
Herellae vaginicola
Klebsiella—all species and all serotypes
Leptospira interrogans—all serotypes
Listeria—all species
Mima polymorpha
Moraxella—all species
Mycobacteria—all species except those listed in Class 3

Mycoplasma—all species except *Mycoplasma mycoides* and *Mycoplasma agalactiae*, which are in Class 5
Neisseria gonorrhoeae, N. Meningitidis
Pasteurella—all species except those listed in Class 3
Salmonella—all species and all serotypes
Shigella—all species and all serotypes
Sphaerophorus necrophorus
Staphylococcus aureus
Streptobacillus moniliformis
Streptococcus pyogenes
Treponema carateum, T. pallidum, and T. pertenue
Vibrio fetus, V. comma, including biotype El Tor, and *V. parahemolyticus*

2. Fungal Agents

***Actinomycetes* (including *Nocardia* species and *Actinomyces species and Arachnia propionica)*
Blastomyces dermatitidis
Cryptococcus neoformans
Paracoccidioides brasiliensis

3. Parasitic Agents

Endamoeba histolytica
Leishmania sp.
Naegleria gruberi
Toxoplasma gondii
Toxocara canis
Trichinella spiralis
Trypanosoma cruzi

4. Viral, Rickettsial, and Chlamydial Agents

Adenoviruses—human—all types
Cache Valley virus
Coxsackie A and B viruses
Cytomegaloviruses
Echoviruses—all types
Encephalomyocarditis virus (EMC)
Flanders virus
Hart Park virus
Hepatitis-associated antigen material
Herpes viruses—except *Herpesvirus simiae* (Monkey B virus) which is in Class 4
Corona viruses
Influenza viruses—all types except A/PR8/34, which is in Class 1
Langat virus
Lymphogranuloma venereum agent
Measles virus
Mumps virus
Parainfluenza virus—all types except Parainfluenza virus 3, SF4 strain, which is in Class 1
Poliviruses—all types, wild and attenuated
Poxviruses—all types except *Alastrim, Smallpox, Monkey pox, and Whitepox*, which depending on experiments, are in Class 3 or Class 4
Rabies virus—all strains except *Rabies street virus*, which should be classified in Class 3 when inoculated into carnivores
Reoviruses—all types
Respiratory syncytial virus
Rhinoviruses—all types
Rubella virus
Simian viruses—all types except *Herpesvirus simiae (Monkey B virus) and Marburg virus*, which are in Class 4
Sindbis virus
Tensaw virus
Turlock virus
Vaccinia virus
Varcella virus
Vole rickettsia
Yellow fever virus, 17D vaccine strain

C. Class 3 Agents

1. Bacterial Agents

*Actinobacillus mallei**
Bartonella—all species
Brucella—all species
Francisella tularensis
Mycobacterium avium, M. bovis, M. tuberculosis
Pasteurella multocide type B ("buffalo" and other foreign virulent strains*)
*Pseudenionas Pseudomallei**
Yersenia pestis

2. Fungal Agents

Coccidioides immitis
Histoplasma capsulatum
Histoplasma capsulatum var. *duboisii*

3. Parasitic Agents

Schistosoma mansoni

4. Viral, Rickettsial, and Chlamydial Agents

****Alastrim, Smallpox, Monkey pox, and Whitepox*, when used in *vitro*
Arboviruses—all strains except those in Class 2 and 4 (Arboviruses indigenous to the United States are in Class 3, except those listed in Class 2. *West Nile* and *Semliki Forest* viruses may be classified up or down, depending on the conditions of use and geographical location of the laboratory.)
Dengue virus, when used for transmission or animal inoculation experiments
Lymphocytic choriameningitis virus (LCM)
Psittacosis-Ornithosis-Trachoma group of agents
Rabies street virus, when used in inoculations of carnivores (See Class 2)
Rickettsia—all species except *Vole rickettsia* when used for transmission or animal inoculation experiments
*Vesicular stomatitis virus**
Yellow fever virus—wild, when used *in vitro*

D. Class 4 Agents

1. Bacterial Agents: None
2. Fungal Agents: None
3. Parasitic Agents: None
4. Viral, Rickettsial, and Chlamydial Agents

****Alasatrim, Smallpox, Monkey pox, and Whitepox*, when used for transmission or animal inoculation experiments
Hemorrhagic fever agents, including *Crimean hemorrhagic fever, (Congo), Junin*, and *Machupo* viruses, and others as yet undefined
Herpesvirus simiae (Monkey B virus)
Lassa virus
Marburg virus
Tick-borne encephalitis virus complex, including *Russian spring-summer encephalitis, Kyasanur forest disease, Omsk hemorrhagic fever*, and *Central European encephalitis viruses*
Venezuelan equine encephalitis virus, epidemic strains, when used for transmission or animal inoculation experiments
Yellow fever virus—wild, when used for transmission or animal inoculation experiments

II. Classification of Oncogenic Viruses on the Basis of Potential Hazard (2)

A. Low-Risk Oncogenic Viruses

Rous Sarcoma
SV-40
CELO
Ad7-SV40
Polyoma
Bovine papilloma
Rat mammary tumor
Avian Leukosis
Murine Leukemia
Murine Sarcoma
Mouse mammary tumor
Rat Leukemia
Hamster Leukemia
Bovine Leukemia
Dog Sarcoma
Mason-Pfizer Monkey Virus
Marek's
Guinea Pig Herpes
Lucke (Frog)
Adenovirus
Shope Fibroma
Shope Papilloma

B. Moderate-Risk Oncogenic Viruses

Ad2-SV40
FeLV
HV Saimiri
EBV
SSV-1
GaLV
HV ateles
Yaba
FeSV

III. Animal Pathogens (3)

A. Animal disease organisms which are forbidden entry into the United States by Law (CDC Class 5 agents)

1. Foot and mouth disease virus

B. Animal disease organisms and vectors which are forbidden entry into the United States by USDA Policy (CDC Class 5 Agents)

African horse sickness virus
African swine fever virus
Besnoitia besnoiti
Borna disease virus
Bovine infectious petechial fever
Camel pox virus
Ephemeral fever virus
Fowl plague virus
Goat pox virus
Hog cholera virus
Louping ill virus
Lumpy skin disease virus
Nairobi sheep disease virus
Newcastle disease virus (Asiatic strains)
Mycoplasma mycoides (contagious bovine pleuropneumonia)
Mycoplasma agalactiae (contagious agalactia of sheep)
Rickettsia ruminatium (heart water)
Rift valley fever virus
Rinderpest virus
Sheep pox virus
Swine vesicular disease virus
Teschen disease virus
Trypanosoma vivax (Nagana)
Trypanosoma evansi
Theileria prava (East Coast fever)
Theileria annulata
Theileria lawrencei
Theileria bovis
Theileria hirci
Vesicular exanthema virus
Wesselsbron disease virus
Zyonema

Footnotes and references of Appendix B

* A USDA permit, required for import and interstate commerce of pathogens, may be obtained from the Animal and Plant Health Inspection Service, USDA, Federal Building, Hyattsville, MD 20782.

** Since the publication of the classification in 1974 [1], the *Actinomycetes* have been reclassified as bacterial rather than fungal agents.

*** All activities, including storage of variola and whitepox are restricted to the single national facility (World Health Organization (WHO) Collaborating Center for Smallpox Research, Center for Disease Control, in Atlanta).

1. *Classification of Etiologic Agents on the Basis of Hazard.* (4th Edition, July 1974). U.S. Department of Health, Education and Welfare, Public Health Service, Center for Disease Control, Office of Biosafety, Atlanta, Georgia 30333.

2. *National Cancer Institute Safety Standards for Research Involving Oncogenic Viruses* (October 1974). U.S. Department of Health, Education, and Welfare Publication No. (NIH) 75-790.

3. U.S. Department of Agriculture, Animal and Plant Health Inspection Service.

Appendix C—Exemptions Under III-D-5

Section III-D-5 states that exempt from these Guidelines are "Other classes of recombinant DNA molecules, if the Director, NIH, with advice of the RAC, after appropriate notice and opportunity for public comment, finds that they do not present a significant risk to health or the environment. (See Section IV-E-1-b-(1)-(d)). Certain classes are exempt as of publication of these Revised Guidelines."

The following classes of experiments are exempt under Section III-D-5 of the Guidelines:

1. *Recombinant DNAs in Tissue Culture.* Recombinant DNA molecules derived entirely from non-viral components (that is, no component is derived from a eukaryotic virus), that are propagated and maintained in cells in tissue culture are exempt from these Guidelines with the exceptions listed below.

Exceptions. Experiments described in Section III-A which require specific RAC review and NIH approval before initiation of the experiment.

Experiments involving DNA from Class 3, 4, or 5 organisms [1] or cells known to be infected with these agents.

Experiments involving the deliberate introduction of genes coding for the biosynthesis of toxins potent for vertebrates. (See Appendix F.)

2. *Experiments Involving E. coli K-12 Host-Vector Systems.* Experiments which use *E. coli* K-12 host-vector systems, with the exception of those experiments listed below, are exempt from these Guidelines provided that (a) the *E. coli* host shall not contain conjugation proficient plasmids or generalized transducing phages, and (b) lambda or lambdoid or Ff bacteriophages or nonconjugative plasmids [49] shall be used as vectors. However, experiments involving the insertion into *E. coli* K-12 of DNA from prokaryotes that exchange genetic information [35] with *E. coli* may by performed with any *E. coli* K-12 vector (e.g., conjugative plasmid). When a nonconjugative vector is used, the *E. coli* K-12 host may contain conjugation-proficient plasmids either autonomous or integrated, or generalized transducing phages.

For these exempt experiments, P1 physical containment conditions are recommended.

Exceptions. Experiments described in Section III-A which require specific RAC review and NIH approval before initiation of the experiment.

Experiments involving DNA from Class 3, 4, or 5 organisms [1] or from cells known to be infected with these agents may be conducted under containment conditions specified in Section III-B-2 with prior IBC review and approval.

Large-scale experiments (e.g., more than 10 liters of culture) require prior IBC review and approval. (See Section III-B-5.)

Experiments involving the deliberate cloning of genes coding for the biosynthesis of toxins potent for vertebrates. (See Appendix F.)

3. *Experiments Involving Saccharomyces cerevisiae Host-Vector Systems.* Experiments which use *Saccharomyces cerevisiae* host-vector systems, with the exception of experiments listed below, are exempt from these Guidelines provided that laboratory strains are used.

For these exempt experiments, P1 physical containment conditions are recommended.

Exceptions. Experiments described in Section III-A which require specific RAC review and NIH approval before initiation of the experiment.

Experiments involving CDC Class 3, 4, or 5 organisms [1] or cells known to be infected with these agents may be conducted under containment conditions specified in Section III-B-2 with prior IBC review and approval.

Large-scale experiments (e.g., more than 10 liters of culture) require prior IBC review and approval. (See Section III-B-5.)

Experiments involving the deliberate cloning of genes coding for the biosynthesis of toxins potent for vertebrates. (See Appendix F.)

4. *Experiments Involving Bacillus subtilis Host Vector-Systems.* Any asporogenic *Bacillus subtilis* strain which does not revert to a sporeformer with a frequency greater than 10^{-7} can be used for cloning DNA, with the exception of those experiments listed below. Indigenous *Bacillus* plasmids and phages, whose host-range does not include *Bacillus cereus* or *Bacillus anthracis,* may be used as vectors.

For these exempt experiments P1 physical containment conditions are recommended.

Exceptions. Experiments described in Section III-A which require specific RAC review and approval before initiation of the experiment.

Experiments involving CDC Class 3, 4, or 5 organisms [1] or cells known to be infected with these agents may be conducted under containment conditions specified by Section III-B-2 with prior IBC review and approval.

Large-scale experiments (e.g., more than 10 liters of culture) require prior IBC review and approvall. (See Section III-B-5.)

Experiments involving the deliberate cloning of genes coding for the biosynthesis of toxins potent for vertebrates. (See Appendix F.)

Appendix D—Actions Taken Under The Guidelines

As noted in the subsections of Sections IV-E-1-b-(1) and IV-E-1-b-(2), the Director, NIH, may take certain actions with regard to the Guidelines after consideration by the RAC.

Some of the actions taken to date include the following:

1. Permission is granted to clone Foot-and-Mouth Disease Virus in the EK1 host-vector system consisting of *E. coli* K-12 and the vector pBR322, all work to be done at the Plum Island Animal Disease Center.

2. Certain specified clones derived from segments of the Foot-and-Mouth Disease Virus may be transferred from Plum Island Animal Disease Center to the facilities of Genentech, Inc., of South San Franciso, California. Further development of the clones at Genentech has been approved under P1+EK1 conditions.

3. The Rd strain of *Hemophilus influenzae* can be used as a host for the propagation of the cloned Tn 10 tet R gene derived from *E. coli* K-12 employing the non-conjugative *Haemophilus* plasmid, pRSF0885, under P1 conditions.

4. Permission is granted to clone certain subgenomic segments of Foot-and-Mouth Disease Virus in HV1 *Bacillus Subtilis* and *Saccharomyces cerevisiae* host-vector systems under P1 conditions at Genentech, Inc., South San Francisco, California.

5. Permission is granted to Dr. Ronald Davis of Stanford University to field test corn plants modified by recombinant DNA techniques under specified containment conditions.

6. Permission is granted to clone in *E. coli* K-12, under P1 physical containment conditions, subgenomic segments of Rift Valley Fever Virus subject to conditions which have been set forth by the RAC.

7. Attenuated laboratory strains of *Salmonella typhimurium* may be used under P1 physical containment conditions to screen for the *Saccharomyces cerevisiae* pseudouridine synthetase gene. The plasmid YEp13 will be employed as the vector.

8. Permission is granted to transfer certain clones of subgenomic segments of Foot-and-Mouth Disease Virus from Plum Island Animal Disease Center to the laboratories of Molecular Genetics, Inc., Minnetonka, Minnesota, and to work with these clones under P1 containment conditions. Approval is contingent upon review of data on infectivity testing of the clones by a working group of the RAC.

Appendix E—Certified Host-Vector Systems

While many experiments using *E. coli* K-12, *Saccharomyces cerevisiae* and *Bacillus subtilis* are currently exempt from the Guidelines under Exemption III-D-5, some derivatives of these host-vector systems were previously classified as HV1 or HV2. A listing of those systems follows:

HV1—The following plasmids are accepted as the vector components of certified *B.*

subtilis HV1 systems: pUB110, pC194, pS194, pSA2100, pE194, pT127, pUB112, pC221, pC223, and pAB124. *B. subtilis* strains RUB 331 and BGSC 1S53 have been certified as the host component of HV1 systems based on these plasmids.

HV2—The asporogenic mutant derivative of *Bacillus subtilis*, ASB 298, with the following plasmids as the vector component: pUB110, pC194, pS194, pSA2100, pE194, pT127, pUB112, pC221, pC223, and pAB124.

HV2—The following sterile strains of *Saccharomyces cerevisiae*, all of which have the ste-VC9 mutation, SHY1, SHY2, SHY3, and SHY4. The following plasmids are certified for use: YIp1, YEp2, YEp4, YIp5, YEp6, YRp7, YEp20, YEp21, YEp24, YIp25, YIp26, YIp27, YIp28, YIp29, YIp30, YIp31, YIp32, and YIp33.

EK2 Plasmid Systems. The *E. coli* K–12 strain chi–1776. The following plasmids are certified for use: pSC101, pMB9, pBR313, pBR322, pDH24, pBR327, pGL101, pHB1. The following *E. coli/S. cerevisiae* hybrid plasmids are certified as EK2 vectors when used in *E. coli* chi–1776 or in the sterile yeast strains, SHY1, SHY2, SHY3 and SHY4: YIp1, YEp2, YEp4, YIp5, YEp6, YRp7, YEp20, YEp21, YEp24, YIp25, YIp26, YIp27, YIp28, YIp29, YIp30, YIp31, YIp32 and YIp33.

EK2 Bacteriophage Systems. The following are certified EK2 systems based on bacteriophage lambda:

Vector	Host
λgt*WES*λ*B* [1]	DP50*supF*
λgt*WES*λ*B**	DP50*supF*
λgtZJ*vir*λ*B* [1]	T3E*coli* K–12
λgtALOλB*	DP50*supF*
Charon 3A	DP50 or DP50*supF*
Charon 4A	DP50 or DP50*supF*
Charon 16A	DP50 or DP50*supF*
Charon 21A	DP50*supF*
Charon 23A	DP50 or DP50*supF*
Charon 24A	DP50 or DP50*supF*.

E. coli K–12 strains chi–2447 and chi–2281 are certified for use with lambda vectors that are certified for use with strain DP50 or DP50supF provided that the su^0 strain not be used as a propagation host.

E. coli K–12 strains chi–1984, chi–2705, chi–2001, and chi–2363 are certified for use with lambda vectors that are certified for use with strain DP50 or SP50supF provided that the su^0 strains not be used as propagation hosts.

Additional certified host-vector systems are as follows:

HV1—The following specified strains of *Neurospora crassa* which have been modified to prevent aerial dispersion:

(1) inl (inositolless) strains 37102, 37401, 46316, 64001, and 89601.

(2) csp-1 strain UCLA37 and csp-2 strains FS 590, UCLA101 (these are conidial separation mutants).

(3) eas strain UCLA191 (an "easily wettable" mutant).

HV1—The following *Streptomyces* species: *Streptomyces coelicolor, S. lividans, S. parvulus*, and *S. griseus*. The following are accepted as vector components of certified *Streptomyces* HV1 systems: *Streptomyces* plasmids SCP2, SLP1.2, pIJ101, actinophage phi C31, and their derivatives.

HV1—*Pseudomonas putida* strain KT2440 with plasmid vectors pKT262, pKT263, and pKT264.

Appendix F—Containment Conditions for Cloning of Genes Coding for the Biosynthesis of Toxins for Vertebrates

1. *General Information.* Appendix F specifies the containment to be used for the deliberate cloning of genes coding for the biosynthesis of toxins for vertebrates. Cloning of genes coding for toxins for vertebrates that have an LD_{50} of less than 100 nanograms per kilogram body weight (e.g., the botulinum toxins, tetanus toxin, diphtheria toxin, *Shigella dysenteria* neurotoxin) is prohibited. No specific restrictions shall apply to the cloning of genes if the protein specified by the gene has an LD_{50} of 100 micrograms or more per kilogram of body weight. Experiments involving genes coding for toxins with an LD_{50} of 100 micrograms or less per kilogram body weight shall be registered with ORDA prior to initiating the experiments. A list of toxins classified as to LD_{50} is available from ORDA. Testing procedures for determining toxicity of toxins not on the list are available from ORDA. The results of such tests shall be forwarded to ORDA, which will consult with the *ad hoc* Working Group on toxins prior to inclusion of the toxin on the list. (See Section IV–E–1–b–(3)–(e).)

2. *Containment Conditions for Cloning of Toxin Genes in E. coli K–12.* (a) Cloining of genes coding for toxins for vertebrates that have an LD_{50} in the range of 100 nanograms to 1000 nanograms per kilogram body weight (e.g., abrin, *Clostridium perfringens* epsilon toxin) may proceed under P2+EK2 or P3+EK1 containment conditions.

(b) Cloning of genes for the biosynthesis of toxins for vertebrates with an LD_{50} in the range of 1 microgram to 100 micrograms per kilogram body weight may proceed under P1+EK1 containment conditions (e.g., *Staphylococcus aureus* alpha toxin, *Staphylococcus aureus* beta toxin, ricin, *Pseudomonas aeruginosa* exotoxin A, *Bordatella pertussis* toxin, the lethal factor of *Bacillus anthracis*, the *Pasteurella pestis* murine toxins, the oxygen-labile hemolysins such as streptolysin O, and certain neurotoxins present in snake venoms and other venoms).

(c) Some enterotoxins are substantially more toxic when administered enterally than parenterally. The following enterotoxins shall be subject to P1+EK1 containment conditions: cholera toxin, the heat labile toxins of *E. coli, Klebsiella*, and other related proteins that may be identified by neutralization with an antiserum monospecific for chlorea toxin, and heat stable toxins of *E. coli* and of *Yersinia enterocolitica*.

3. *Containment Conditions for Cloning of Toxins Genes in Organisms Other than E. coli K–12.* Requests involving the cloning of genes coding for toxins for vertebrates in host-vector systems other than *E. coli* K–12 will be evaluated by ORDA, which will consult with the *ad hod* working group on toxins. (See Section IV–E–1–b–(3)–(f).)

4. *Specific Approvals.* a. Permission is granted to clone the Exotoxin A gene of *Pseudomonas aeruginosa* under P1 conditions in *Pseudomonas aeruginosa*.

b. The pyrogenic endotoxin type A (Tox A) gene of *Staphylococcus aureus* may be cloned in an HV2 *Bacillus subtilis* host-vector system under P3 containment conditions.

c. Permission is granted to clone in *E. coli* K–12, in high containment Building 550 at the Frederick Cancer Research Facility, restriction fragments of *Corynephage Beta* carrying the structural gene for diphtheria toxin. Laboratory practices and containment equipment are to be specified by the IBC.

d. The genes coding for the Staphylococcus aureus determinants, A, B, and F, which may be implicated in toxic shock syndrome, may be cloned in *E. coli* K–12 under P2+EK1 conditions. The *Staphylococcus aureus* strain used as the donor is to be alpha toxin minus. It is suggested that, if possible, the donor *Staphylococcus aureus* strain should lack other toxins with LD_{50}s in the range of one microgram per kilogram body weight, such as the exfoliative toxin.

e. Fragments F–1 and F–2 of the diphtheria toxin gene (tox) may be cloned in *E. coli* K–12 under P1+EK1 containment conditions. Fragment F–1 and fragment F–2 both contain (i) some or all of the transcriptional control elements of *tox*, (ii) the signal peptide, and (iii) fragment A (the center responsible for ADP-ribosylation of elongation factor 2).

f. The gene(s) coding for a toxin (designated LT-like) isolated from *E. coli* which is similar to the *E. coli* heat labile enterotoxin (LT) with respect to its activities and mode of action, but is not neutralized by antibodies against cholera enterotoxin or against LT from human or porcine *E. coli* strains and sequences homologous to the *E. coli* LT-like toxin gene may be cloned under P1+EK1 conditions.

Dated: April 12, 1982.

Bernard Talbot,

Acting Director, National Institute of Allergy and Infectious Diseases.

Note.—OMB's "Mandatory Information Requirements for Federal Assistance Program Announcements" (45 FR 39592) requires a statement concerning the official government programs contained in the *Catalog of Federal Domestic Assistance*. Normally NIH lists in its announcements the number and title of affected individual programs for the guidance of the public. Because the guidance in this notice covers not only vitually every NIH Program but also essentially every federal research program in which DNA recombinant molecule techniques could be used, it has been determined to be not cost effective or in the public interest to attempt to list these

programs. Such a list would likely require several additional pages. In addition, NIH could not be certain that every federal program would be included as many federal agencies, as well as private organizations, both national and international, have elected to follow the NIH Guidelines. In lieu of the individual program listing, NIH invites readers to direct questions to the information address above about whether individual programs listed in the *Catalog of Federal Domestic Assistance* are affected.

NIH programs are not covered by OMB Circular A-95 because they fit the description of "programs not considered appropriate" in Section 8-(b)- (4) and (5) of that Circular.

[FR Doc. 82-10660 Filed 4-20-82; 8:45 am]
BILLING CODE 4140-01-M

federal register

Wednesday
April 21, 1982

Part IV

Department of Education

PLUS Program Regulations

DEPARTMENT OF EDUCATION

34 CFR Part 683

PLUS Program

AGENCY: Department of Education.

ACTION: Final regulations.

SUMMARY: The Secretary of Education issues regulations for the PLUS program. These regulations implement the statutory changes under Title IV, Part B of the Higher Education Act of 1965, that authorize a program of loan insurance for independent undergraduate students, graduate and professional students and the parents of dependent undergraduate students who wish to borrow funds to meet educational costs. These regulations constitute a comprehensive package of program requirements based on the January 19, 1981 notice of proposed rulemaking (NPRM). The regulations apply to the portion of the PLUS program that is administered by State and private nonprofit guarantee agencies, and the direct Federal loan guarantee program known as the Federal PLUS program.

EFFECTIVE DATE: Unless Congress takes certain adjournments, these regulations will take effect 45 days after publication in the **Federal Register** except §§ 683.11, 683.12, 683.13, 683.14, 683.16, 683.17, 683.30, 683.31, 683.32, 683.33, 683.34, 683.35, 683.36, 683.37, 683.51, 683.52, 683.53, 683.54, 683.56, 683.57, 683.58, 683.59, 683.60, 683.61, 683.62, 683.63, 683.65, 683.66, 683.80, 683.81, 683.82, 683.83, 683.84, 683.87, 683.88, 683.89, 683.90, and 683.91 which contain information collection requirements which are under review at the Office of Management and Budget (OMB). These sections will take effect after OMB's approval has been obtained and the public has been notified to that effect through a Notice published in the **Federal Register**. If you want to know the effective date of these regulations, call or write the Department of Education contact person. At a future date, the Secretary will publish a notice in the **Federal Register** stating the effective date of those sections of the regulations that are subject to OMB review.

FOR FURTHER INFORMATION CONTACT: Cheryl Leibovitz, Program Specialist, or Larry Oxendine, Policy Section Chief, Guaranteed Student Loan Branch, Division of Policy and Program Development, Department of Education, 400 Maryland Ave., SW., Washington, D.C. 20202 (202) 245–2475.

SUPPLEMENTARY INFORMATION:

Background

The Education Amendments of 1980 added provisions to Part B of Title IV of the Higher Education Act of 1965 (Act), authorizing the Federal Government to insure or reinsure loans made to parents of dependent undergraduate students. This program was named the PLUS program. The Postsecondary Student Assistance Amendments of 1981 further extended borrower eligibility under the PLUS program to include independent undergraduate students and graduate or professional students, and raised the interest rate from 9 percent to 14 percent for all new loans. The Act specifies that unless otherwise specified in the Act, PLUS loans have the same terms, conditions and benefits as those governing loans to students under the Guaranteed Student Loan Program (GSLP). The Act:

1. Limits the amount a parent may borrow to $3,000 per academic year on behalf of each dependent undergraduate student, with an aggregate maximum of $15,000 for each student;
2. Limits the amount a graduate or professional student may borrow to $3,000 per academic year, with an aggregate maximum of $15,000, in addition to the amounts he or she may borrow under the GSLP;
3. Limits the amount an independent undergraduate student may borrow to $2,500 per academic year, with an aggregate maximum of $12,500, under both the GSLP and PLUS program;
4. Limits the amount a student may borrow and, if a parent borrows, the combined amount a parent and student may borrow, for an academic year to the student's allowable cost of attendance less estimated financial assistance;
5. Sets the interest rate for these loans at 14 percent but provides that, based on 91-day Treasury bill rates, the interest rate may in the future be reduced to 12 percent for new loans;
6. Prohibits the payment of interest benefits (payments of interest by the Federal Government on the borrower's behalf during authorized periods) to lenders, although lenders may qualify for special allowance payments (Federal payments to supplement interest on loans to provide a yield roughly corresponding to the current market rate);
7. Requires that repayment begin within 60 days of the disbursement of the loan; and
8. Requires the Secretary of Education to define the terms "parents of a dependent undergraduate student" and "graduate or professional student."

Under the PLUS program, as under the GSLP, loans may be insured either by a State or private nonprofit guarantee agency or by the Federal Government. In the case of loans insured by a guarantee agency, the Federal Government reimburses a guarantee agency for up to 100 percent of its losses on default claims, pays the agency certain administrative cost allowances, and provides advances to strengthen the agency's reserve fund. To be eligible for the 100 percent Federal reimbursement, a guarantee agency must provide insurance for all categories of eligible PLUS loan borrowers.

The circumstances under which the Federal PLUS program may operate are statutorily different from those permitting the Federal Insured Student Loan Program (FISLP) to operate. The Federal PLUS program may operate in the following circumstances:

1. In a State that is not served by a State or private nonprofit guarantee agency.
2. In a State served by a State or private nonprofit guarantee agency, if such agency has not authorized a PLUS program and is not prohibited from doing so by State law.
3. In a State served by a State or private nonprofit guarantee agency, if such agency is prohibited by State law enacted prior to January 1, 1981, from authorizing a PLUS program, one hundred and twenty days after the adjournment of the first regular session of the State legislature which convenes after January 1, 1981.

The result of these statutory provisions is that if a State guarantee agency is currently prohibited by State law from authorizing a PLUS program, the availability of the PLUS program depends upon the meeting schedule of the State legislature. Where there are no State legal prohibitions to implementing the PLUS program, a period of time will be required in most States after the guarantee agency authorizes the program before it can be implemented. Where such a program is possible under State law but is not established, or where a guarantee agency fails to authorize a PLUS program within the period mentioned above, the Federal Government stands ready to insure PLUS loans directly as necessary to assure their availability for all eligible borrowers. Unlike the FISLP, the Federal PLUS program is not authorized under any circumstances to insure loans for a lender in a State served by a guarantee agency PLUS program.

Major Provisions of the Regulations

To the extent possible, the Secretary has modeled the PLUS program regulations on the existing GSLP

regulations, which were formerly in 45 CFR Part 177 but are now found in 34 CFR Part 682. The Secretary has done this in an effort to initiate the PLUS program with a minimum of confusion, since lenders and schools are already familiar with the GSLP requirements. The following summarizes the major differences between the GSLP and the PLUS program requirements and cites other provisions that are unique to the PLUS program.

1. *Eligibility of a Borrower.* (a) *Definition of Parent.* By statute, the Secretary is responsible for defining the term "parent." The Secretary has defined "parent" as a person's natural or adoptive mother or father or legal guardian, which is consistent with the definition of "parent" used in the other Federally administered financial aid programs.

(b) *Number of Borrowers and Applicable Loan Limits.* In the PLUS program there may be two persons who are eligible to borrow on behalf of one dependent student: the student's mother and father. Therefore, these regulations permit, but do not require, eligible parents to obtain a loan on behalf of a student as co-makers (persons who are jointly liable). It is important to note that if two parents obtain a loan as co-makers, both parents are equally liable for the entire amount of the loan. Therefore, both parents must qualify for any special benefit associated with the loan, such as deferment or cancellation of payments. Endorsers (persons who are secondarily liable) are prohibited by statute on Federal PLUS loans, unless the borrower is a minor and cannot under applicable local law create a legally binding obligation by his or her own signature, and are authorized to the extent permitted by guarantee agency policy or State law on guarantee agency PLUS loans.

The comments received reveal considerable confusion regarding the limitation on the total loan amount applicable when two parents borrow separately on behalf of the same student for an academic year. The $3,000 limitation applies to the total amount that may be borrowed on behalf of a student for one academic year, whether one parent obtains one PLUS loan, two parents obtain one PLUS loan as co-makers, or two parents obtain two loans independently on the student's behalf.

(c) *Creditworthiness.* The regulations impose no requirements related to an applicant's credit background or current ability to pay. Also, as in the GSLP, the PLUS loan must be made without security, and, in the Federal PLUS program, unless the borrower is a minor and cannot under applicable local law create a legally binding obligation by his or her own signature, without endorsement.

Lenders may use their own discretion in requiring a prospective borrower to undergo a credit analysis prior to making a loan. Guarantee agencies may also impose requirements on their participating lenders that would make credit analysis part of the lender's due diligence requirements in making the loan. However, in either case, the lender may not pass on the cost of credit analysis to the borrower.

2. *Federal Reinsurance.* PLUS loans guaranteed by State and private nonprofit guarantee agencies are eligible for Federal reinsurance on the same basis as GSLP loans. Guarantee agencies that meet certain statutory criteria qualify to receive reimbursement of 80 percent of their default losses (reinsurance). Further, guarantee agencies which meet additional criteria may receive reimbursement of up to 100 percent of their losses (supplemental reinsurance).

It has been suggested that a guarantee agency establish its eligibility for reinsurance or supplemental reinsurance jointly for both the GSLP and the PLUS program. Although this would be the least burdensome procedure administratively for both the Department of Education and the guarantee agencies, it is contrary to the statutory provisions governing reinsurance. Therefore, these regulations require the guarantee agency to qualify for Federal reinsurance separately for the GSLP and the PLUS program. With separate eligibility criteria it is possible, although not probable, that a guarantee agency may not qualify, or wish to qualify, for the same extent of reinsurance coverage for both the GSLP and the PLUS program. In this event, these regulations require that the guarantee agency maintain segregated records for the two programs.

3. *Lender Agreements.* Section 433A of the Act, added by the Education Amendments of 1980, requires that the Secretary of Education enter into an agreement with each eligible lender in the GSLP and the PLUS program to ensure that each lender provides thorough and accurate loan information to student and parent borrowers.

The Secretary has recently revised the Federal contract of insurance to fulfill this requirement under the FISLP and the Federal PLUS program.

In addition, the Secretary has provided the required agreement for all guarantee agency lenders. No GSLP or PLUS loan may be insured or guaranteed for a lender without an executed contract or agreement on file with the Secretary.

This new statutory provision has been incorporated into the PLUS program regulations. It does not appear in current GSLP regulations but will be added.

4. *Insurance Premium.* The insurance premium rate which the guarantor may charge the lender under the PLUS program is the same as that authorized under the GSLP: one-fourth of 1 percent per year for a loan insured by the Federal Government, and a maximum of 1 percent per year for a loan guaranteed by a State or private nonprofit guarantee agency. Under the GSLP, the Federal Government and many guarantee agencies calculate the insurance premium from the date of disbursement to the beginning of the repayment period. In the event an agency also charges a premium during the repayment period, GSLP regulations require it to make a refund if the borrower prepays, defaults, dies, becomes totally and permanently disabled, or has the loan discharged in bankruptcy. The refund requirement has proved to be costly and troublesome; therefore, to avoid making refunds, as a matter of practice, no guarantee agency under the GSLP charges the premium during the repayment period.

Under the PLUS program, the borrower enters the repayment period on the date the loan is disbursed. Therefore, the formula used in the GSLP cannot be applied to PLUS loans. Under the Federal PLUS program, the Secretary plans to calculate the insurance premium on the declining principal balance over the life of the loan, which would result in a maximum premium of approximately $37 on a $3,000 loan. Guarantee agencies retain the option to charge up to 1 percent of the declining principal balance and may extend that over the life of the loan. This would result in a maximum premium of approximately $150. No refund is required under the PLUS program, whether the loan is insured by a guarantee agency or by the Federal Government.

5. *Deferment and Cancellation Provisions.* Borrowers under the PLUS program are entitled to the same deferment and cancellation provisions as authorized under the GSLP. In the event a borrower is eligible for an authorized deferment (e.g., for military service) at the time the borrower obtains the loan and, simultaneously, enters the repayment period, the borrower is entitled to an immediate deferment. However, it should be pointed out that if the loan was obtained by two eligible parents as co-makers, both parties to the

loan must qualify for a deferment before payments may be deferred. Unless both co-makers qualify for a deferment, both parents remain liable; neither is relieved of the obligation to pay. If there is an endorser who is secondarily liable, only the borrower need qualify for the deferment or cancellation. Likewise, with respect to cancellation because of death or total and permanent disability, if the loan was obtained by eligible parents as co-makers, both borrowers must be eligible for cancellation; otherwise payments must be made when due. Also, if only one parent's debts are discharged in bankruptcy, the co-maker is responsible for repayment of the loan. It should be noted that where a PLUS loan is made to a parent borrower, the deferment and cancellation provisions apply solely to the parent; the status of the student has no bearing on the parent's obligation to repay a PLUS loan.

The deferment and cancellation provisions are identical to those in the GSLP regulations. The regulations include the new deferment provisions authorized by the Education Amendments of 1980 and one provision authorized in 1978. These new provisions were added to the GSLP regulations in a final regulation published on January 16, 1981 (46 FR 3866).

6. *Check Disbursement and School Notification.* a. *Federal PLUS requirements.* Under the GSLP regulations, a FISLP check must be mailed directly to the school named on the loan application for delivery to the borrower. The Secretary has chosen a different check disbursement procedure for the Federal PLUS program, requiring that the loan check be sent directly to the borrower, payable to the borrower.

The Secretary has chosen this method in the belief that, at least initially, most PLUS loans will be made to parents, who, unlike student borrowers, will not be at the school named on the loan application to receive the check directly. Negotiating checks through the mail, especially in cases of jointly payable checks, would be cumbersome and administratively burdensome for both parents and schools. Since the Secretary believes that it would be confusing to lenders to establish one PLUS check disbursement procedure for parent borrowers and a different procedure for student borrowers, the regulations require all PLUS loan checks to be delivered to the borrowers.

The proposed regulations for the Federal PLUS program required that the lender notify the school of the name of the student for whom the loan was intended and the amount of the loan. The statute requires that the school be notified of this information, but provides the option that notification be made by either the lender or the guarantor.

Rather than burden the lender with having to send a separate notice to the school, since the Federal PLUS loan check *may not* be sent to the school, the school notification requirement which appeared in § 683.59 of the NPRM has been deleted. Instead, the Secretary, as the insurer of Federal PLUS loans, will notify the school of the loan amount and the name of the lender.

b. *Guarantee agency PLUS program requirements.* Since the statute provides that the insurer may require the lender to mail the check to the school for delivery to the borrower, this option is retained in these regulations for guarantee agencies. However, guarantee agencies are urged to review carefully the ramifications this procedure would have on the PLUS program before adopting it.

Public Comment

On January 19, 1981 a Notice of Proposed Rulemaking (NPRM) was published in the **Federal Register** (46 FR 4956) inviting comments on the proposed rules governing the PLUS program which, at that time, permitted only parent borrowers. In addition, both before the NPRM was published and after the Postsecondary Student Assistance Amendments of 1981 extended borrower eligibility to graduate and professional students and independent undergraduate students, the Secretary consulted with representitives from various groups that participate in the GSLP and that will play active roles in the PLUS program. Since the rules required by the 1981 amendments did not require significant policy-making, they have been incorporated into these final regulations without being published as an NPRM.

Twenty-five commenters, representing schools, lenders, guarantee agencies and professional groups, responded to the January 19, 1981 NPRM. A summary of the comments and the Secretary's responses to them is included in this Preamble. The comments and responses appear in the numerical sequence of the regulations and are identified by the number and title of the section to which they relate.

In establishing these regulations the Secretary has carefully considered all options and recommendations offered by the public. Some comments, however, concern aspects of the regulations that are dictated by the statute. These comments are beyond the scope of regulatory change and are not addressed in the comments and responses that appear in this Preamble. There were also many comments that concern requirements that apply to the GSLP regulations (34 CFR Part 682) as well as to the PLUS program (for example, changing the number of clock hours for purposes of determining an academic year and a full-time student at an institution which uses clock hours, or modifying the extent to which a school must provide information to prospective students). These recommendations, along with other proposals for program improvements, will be addressed in an NPRM covering the GSLP and the PLUS program. To minimize confusion and errors in administration, every effort will be made to assure uniformity, consistent with the Act, in regulations governing the GSLP and the PLUS program.

Summary of Comments and Responses

Many comments were received on three issues which appear in several sections of the regulations. To avoid repeating the comments for each section, the Secretary addresses these three issues (allocation of refunds; statement of educational purpose; and the PLUS loan check) in a "general" section.

General.

Allocation of tuition refunds to PLUS loans. Comment. Quite a few commenters objected to the provision requiring the student to authorize the school to return any refund of school charges allocable to the PLUS loan to the lender. They argued that the PLUS loan proceeds belong only to the parent borrower and that the student, on whose behalf the loan was made, has no right to the refund. This provision appears several times in the regulation, and comments were received in reference to § 683.11(b)(9) (Parent and student eligibility) and § 683.31(b)(4)(ii)(E) (Basic PLUS program agreement) in the NPRM.

Response. Since a PLUS loan is obtained to cover educational costs, it is appropriate to apply a portion of a refund of those costs toward payment of the loan, even where the borrower is the student's parent. Where the borrower is a parent, the student must authorize the school named on the loan application to pay to the parent's lender any refund that the school allocated to the PLUS program. The student's authorization is necessary because, although the responsibility for repaying the PLUS loan is the parent's the refund belongs to the student, who is responsible for paying the school tuition. The requirement that the student authorize the school to send the PLUS portion of the refund to the lender reduces the

possibility of abuse resulting from the student's taking the money for non-educational costs.

Statement of educational purpose. Comment. One commenter proposed that, to prevent a parent from obtaining a PLUS loan without the student's knowledge, and using the loan proceeds for something other than the student's education, the student should sign a statement authorizing the school to process the application.

Response. Both the prospective borrower and the student on whose behalf a loan is made, if the borrower is a parent, must complete a section of the PLUS application. This requirement should prevent a parent's borrowing without the student's knowledge.

Comment. Several commenters recommended that lenders be afforded some recourse if the parent fails to use the loan funds for educational purposes.

Response. The regulations contain provisions designed to ensure that borrowers use PLUS loan funds for educational purposes only. The prospective borrower must certify on the loan application that the loan funds will be used for educational costs. The promissory note, which must be signed by the borrower, contains a similar statement of educational purpose. Section 683.57(e) stipulates that if a student never enrolls, the full amount of the loan is immediately due and payable. These requirements provide a basis for recourse against the borrower if the student does not enroll.

Comment. One commenter suggested a regulation that addressed the return of funds in cases where a student enrolls and withdraws early.

Response. As noted in a preceding response, if a student withdraws early and the school attributes a portion of a tuition refund to the PLUS loan, the school must send that portion to the lender. Also, if the student never enrolls, the entire loan is immediately due and payable. However, if the student enrolls and then withdraws early, the regulations do not require the student to return to the lender the loan funds intended to meet educational costs other than tuition that are unused or unearned. Since the borrower may use the PLUS loan for educational costs such as room and board, books, and transportation, it would be difficult to determine what portion of the loan should be returned if a student withdraws from school. The determination would involve the borrower, the lender, and the school in burdensome computations to determine the educational expenses actually incurred. Since the borrower begins repaying the loan within 60 days after it is made, the benefit of such computations is minimized. These regulations regarding tuition refunds, failure to enroll, and early withdrawal are the same as the GSLP regulations governing those issues.

The PLUS loan check. Comments. Numerous comments were received regarding the handling of the PLUS loan check. The majority of commenters were of the option that, where the borrower is a parent, the school's involvement in the loan transaction is minimal, as the PLUS loan is a transaction between the lender and the parent. Therefore, these commenters believed that the school should not handle the loan check. Several commenters felt that jointly payable checks would be an administratively burdensome requirement; one commenter proposed a technical amendment to the law prohibiting guarantee agencies from allowing jointly payable checks. Another commenter suggested that all checks be made jointly payable and mailed to the school.

Response. The Secretary is requiring that all Federal PLUS checks be sent directly to the borrower, bearing only the borrower' name. The Secretary expects that many PLUS borrowers will be parents who, unlike student borrowers, will not be enrolled at the school for which the loan was intended to receive the check directly. The Secretary agrees that negotiating checks through the mail, especially in cases of jointly payable checks, would be administratively burdensome. Since the statute provides that the insurer may require the lender to mail the check to the school for delivery to the borrower, this option is retained in these regulations for guarantee agencies. However, guarantee agencies are urged to review carefully the ramifications this procedure will have on the PLUS program before adopting it.

Subpart B—General Provisions

§ 683.10 General Definitions.

"Cost of Attendance." Comment. One commenter felt that, in the case of a student whose tuition and fee charges will be less than those assessed a full-time student, the financial aid administrator should be required to calculate the actual amount of tuition and fees.

Response. The definition of the cost of attendance set forth in the Postsecondary Student Assistance Amendments of 1981 is virtually identical to that in the GSLP legislation prior to the enactment of the Middle Income Student Assistance Act, enacted in 1978. The definition authorizes a financial aid administrator to calculate, for a less than full-time student, the actual tuition and fees. The new definition is included in these regulations.

Comment. One commenter said the reference to academic year 1981–82 was confusing and should either be clarified or deleted.

Response. The Secretary agrees and has eliminated the reference to academic year 1981–82.

"Borrower." Comment. Two commenters felt that, as written, the definition of "borrower" implied that *each* parent can borrow $3,000 on behalf of one son or daughter for the same academic year.

Response. The Secretary disagrees. The definition of "borrower" merely states that there may be more than one parent *eligible* to borrow on behalf of a student. However, the sections of the regulations which address loan limits (§§ 683.31 and 683.55) specify a maximum annual loan amount for an eligible student that may not be exceeded. Therefore, the amount that can be borrowed on behalf of one eligible student is limited to $3,000, regardless of whether one or two parents borrow.

Comment. One commenter questioned the ability of a parent who is also a student to borrow simultaneously under the GSLP on his or her own behalf and under the PLUS program on behalf of one or more dependent students.

Response. As long as all the eligibility requirements for both programs are met, the Secretary imposes no restrictions prohibiting a parent who is a student from financing his or her own education under the GSLP—and now under the PLUS program—and his or her children's education under the PLUS program. However, it should be noted that when a borrower applies for either a GSLP or PLUS loan, the applicant must list all outstanding debts under any Federal loan program on the application, and the lender may take these debts into account in determining whether or not it will make the loan.

Comment. One commenter recommended adding the definition of "co-maker" to the regulations.

Response. The Secretary has clarified the regulation to make clear that if there are co-makers, both co-makers are considered to be the "borrower." The term "co-maker" is referenced in the definition of "borrower."

"Estimated Financial Assistance." Comment. Several commenters asked how to treat PLUS loans and GSLP loans in determining a student's estimated financial assistance. As written in the

NPRM, inclusion of a PLUS loan as "estimated financial assistance" is optional. One commenter strongly urged that when making this determination, inclusion of the PLUS loan either be required or prohibited.

Response. The Secretary agrees that the inclusion of a PLUS loan as estimated financial assistance is not discretionary and has changed the regulation. The PLUS regulation (like the current GSLP regulation) now states that any assistance awarded to a student in "Federal, State, . . . grant, work or loan programs" is included as "estimated financial assistance," which encompasses PLUS loans and GSLP loans.

Comment. One commenter asked if a PLUS loan could be considered as part of the expected family contribution, if the student has applied for need-based aid.

Response. The Postsecondary Student Assistance Amendments of 1981 permit any loan made under the PLUS program to be counted as part of the student's expected family contribution in the determination of need under any other Title IV program. Neither the GSLP nor the PLUS regulations address the treatment of either loan program in relation to the expected family contribution.

"Independent Student." Comment. The comments received on this subject dealt primarily with the concept of the independent student rather than the proposed definition. One commenter strongly objected to the school being responsible for determining the dependent or independent status of the student, citing that the majority of the students at his school did not receive need-based aid and that, therefore, the pertinent information was not readily accessible. His suggestion was to include the status determination in the PLUS application.

Response. In formulating regulations for Title IV programs, the Secretary made an effort to standardize definitions wherever possible. As a result, the definition of "independent student" is the same for all Title IV programs, and the same three questions are used in determining the student's dependent or independent status. Schools are responsible for making the student status determination. The regulations permit the school to rely on information provided by the student in determining the student's status. The Secretary expects that most students who borrow or whose parents borrow a PLUS loan will have applied for other Federal student aid as well. Since the same independent/dependent determination must be made for other Federal student aid programs, the determination will already have been made for most students who seek or whose parents seek PLUS loans. Therefore, this regulation under the PLUS program will impose no additional burden on most schools.

In conjunction with the guarantee agencies, the Secretary has developed a common PLUS application form for the Federal and guarantee agency PLUS programs. The Secretary has not included the independent/dependent status determination on the PLUS application because: (1) The necessary information is available for most students through information supplied by them in connection with other Federal student aid, and (2) the yearly modification of forms would be burdensome and costly.

Comment. One commenter cited the possibility of a student applying for and receiving a GSLP loan as an independent student and the student's parents later applying for a PLUS loan. The commenter asked what to do in such a case.

Response. Under the application process developed by the Secretary in conjunction with the guarantee agencies, this conflict should not arise. If a student has been determined to be an independent student for purposes of the GSLP—or any other Title IV program—he or she is not eligible to have the parent borrow on his or her behalf under the PLUS program.

Comment. One commenter questioned the rationale for using fiscal years ending June 30 for determining the independent or dependent status of the student.

Response. In an effort to standardize the administration of Title IV student financial aid programs, the Secretary has adopted the July 1–June 30 dates in the PLUS program for determining dependency because they coincide with the dates used by schools for the campus-based and Pell Grant programs.

§ 683.11 Parent and student eligibility.

Comment. One commenter noted that the term "good standing" has been eliminated from the law.

Response. The Secretary has changed the regulation accordingly.

Comment. One commenter asked for clarification as to who was requried to certify the default status of the parent and the student required by § 683.11(f) of the NPRM.

Response. The Secretary agrees that clarification was needed and accordingly has modified the regulation. The regulation now requires that the borrower certify on the application that he or she is not in default, and requires that a student, on whose behalf a parent is borrowing, certify that he or she is not in default. The school may rely on those statements as long as the school does not have contrary information.

Comment. Two commenters questioned the reasonableness and effectiveness of the provision which limits the parent and student default experiences under Title IV aid to the same school which the student is or will be attending (§ 683.11 (d)(1) and (d)(2) of the NPRM).

Response. Section 484(a)(4) of the Act stipulates that a student is ineligible for further Title IV aid if that student is in default on a loan obtained for attendance at the same school he or she is or will be attending. All loans made under the PLUS program are subject to that restriction. In addition, guarantee agencies may (and many do) consider a student ineligible if he or she has defaulted on a Title IV loan received for attendance at any school.

Comment. One commenter asked if a guarantor could deny further loan eligibility to an applicant who had previously had his or her debts discharged in bankruptcy, even though the Secretary does not consider a PLUS, GSLP, or NDSL loan that is discharged in bankruptcy to be a default.

Response. The Secretary does not consider the prior bankruptcy of an applicant as grounds for ineligibility. However, if, while making a credit decision, the lender discovers a pattern which indicates the applicant is unlikely to repay, the lender may deny that application.

§ 683.16 Provision of loan information by lenders.

Comment. One commenter suggested that, where the borrower is a parent, the lender supply the student as well as the parent with a copy of the terms and conditions of the PLUS loan.

Response. In such circumstances, under the statute, the rights and responsibilities for the repayment of a PLUS loan rest only with the parent borrower, not the student. Therefore, the Secretary has not adopted this suggestion as a requirement, although lenders who wish to follow the suggestion may do so.

Subpart C—Guarantee Agency Programs

§ 683.31 Basic agreement.

Comment. One commenter expressed concern regarding the 15-year maximum repayment period for a PLUS loan, since the maximum repayment period under the Federal Credit Union Act is 12 years.

Response. Since the 15-year maximum repayment period begins to run at the same time the 10-year period begins, when the PLUS loan is made and the repayment period commences, the 15-year "life of the loan" rule has no effect on PLUS loans and has been dropped from the regulation. A lender may establish any repayment period for a PLUS loan, as long as it is at least five years and no more than 10 years, excluding periods of deferment and forbearance, consistent with the rule that payments total at least $600 per year, unless the lender and borrower agree to a lesser amount

Comment. One commenter asked if the date of disbursement, which determines the beginning of the repayment period, is the date that appears on the check.

Response. The date of disbursement is the date the check is sent to the borrower. The date that appears on the check may be considered evidence of the date of disbursement.

Subpart D—Federal PLUS Program

§ 683.50 Circumstances under which loans may be insured.

Comment. One commenter expressed concern over the more general statement of circumstances under which PLUS loans may be insured under the Federal program, as opposed to the requirements in effect for the GSLP. The commenter requested the regulation be rewritten more specifically.

Response. As written, the regulation mirrors the law. The authority for limited Federal insurance in a State served by a guarantee agency, which exists in the GSLP, does not exist in the PLUS Program. For example, lenders with customers in several States may not obtain Federal insurance on PLUS loans, as they can for student loans, on the grounds that due to residency requirements one guarantee agency will not serve substantially all of their borrowers.

§ 683.59 Due diligence in making and disbursing a loan.

Comment. Two commenters expressed concern that, in certain cases such as long-distance lending, a personal interview with the prospective borrower may be impossible.

Response. The Secretary recognizes that a personal interview is not always possible. The use of the word "should" in § 683.59(d) of the NPRM indicates that an interview is recommended, but not required.

Comment. One commenter questioned the relevance of a lender notifying a school of a late disbursement at the time it sends the loan check to the school, as required by § 683.86(f) in the NPRM.

Response. In deleting § 683.86, the Secretary has also deleted this provision.

§ 683.62 Assignment of a loan.

Comment. One commenter noticed that the word "not" was missing from the first sentence of the section.

Response. The Secretary agrees and has made the correction.

Subpart E—Requirements, Standards and Payments for Participating Schools

§ 683.84 Certifications by a participating school in connection with a parent's loan application.

Comment. One commenter suggested deleting the requirement that the school certify the eligibility of a parent for a PLUS loan.

Response. In certifying the eligibility of a parent for a PLUS loan, the Secretary requires the school to certify that, to the best of its knowledge, the information on the application is true, and the parent is not in default on a loan and does not owe a refund on a grant under Title IV received for costs of attendance at that school. Under § 683.11, the school is allowed to accept statements signed by parents and students concerning their default status, unless the school has information to the contrary.

§ 683.85 Administrative cost allowance to participating schools.

Comment. Several commenters requested clarification of how students and parents will be counted for purposes of the PLUS program and the GSLP.

Response. This question is no longer relevant, since the Postsecondary Student Assistance Amendments of 1981 repealed the Secretary's authority to pay an administrative cost allowance to a school.

§ 683.89 Payment of a refund to a lender.

Comment. Several commenters questioned how a school should handle cases in which a student receives too much aid as a result of GSLP and PLUS loans and campus-based aid, and what refunds or award adjustments are due.

Response. When completing a loan application under the GSLP or PLUS program, a financial aid administrator is required to consider all the financial aid information available on both the loan applicant and the student, when the applicant is a parent. If a financial aid administrator learns later, but before the lender disburses the full amount of the GSLP or PLUS loan, that a student has received additional financial assistance which results in a loan amount in excess of the student's cost of attendance, the financial aid administrator may inform the lender of this fact, and the lender may lower the loan amount accordingly. The school has no authority to adjust the amount of a GSLP or PLUS loan by withholding funds from the student.

The Student Assistance General Provisions regulations (34 CFR Part 668) establish procedures for apportioning tuition refunds and handling overawards in the Title IV programs. The National Direct Student Loan program regulations (34 CFR 674.14) provide procedures for handling overawards of NDSL funds. The Supplemental Educational Opportunity Grant Program regulations (§ 676.14) provide procedures for handling overawards of SEOG funds. The College Work-Study regulations also provide refund procedures (34 CFR 675.14).

Comment. One commenter suggested requiring schools to track the student's enrollment status on a Student Confirmation Report (SCR) or a similar document.

Response. The main purpose of the SCR in the GSLP is to alert lenders to the beginning of a student's grace period which begins on the date the school reports that the student has graduated, withdrawn or ceased to be enrolled on at least a half-time basis. In the PLUS program, the borrower is in the repayment period as soon as the loan is disbursed and, therefore, the student's status is relevant only if the full amount of the loan is immediately due (because the student fails to enroll); if a refund is due to the lender; or if a student borrower whose principal payments have been deferred ceases to be enrolled on a full-time basis (which signals the resumption of the repayment period). The Secretary does not believe a regular reporting system like the SCR is necessary for these limited purposes.

§ 683.91 Records, reports and inspection requirements for participating schools.

Comment. Several commenters questioned the need for the schools to keep PLUS loan information on file, including the name of the lender.

Response. The schools must keep the name of the lender on file so that if a refund which is allocable to a PLUS loan is due, the school will know where to send it.

Executive Order 12291

These regulations have been reviewed by the Department of Education in accordance with Executive Order 12291

and are classified as non-major because they do not meet the criteria for major regulations established in the Order.

Assessment of Educational Impact

The Secretary requests comments on whether these regulations would require institutions of higher education to transmit information that is already being gathered by, or is available from, any other agency or authority of the United States.

Paperwork Reduction Act of 1980

In accordance with the Paperwork Reduction Act of 1980 (Pub. L. 96-511), the recordkeeping provisions that are included in these regulations have been or will be submitted for approval to the Office of Management and Budget (OMB). These provisions (§§ 683.11, 683.12, 683.13, 683.14, 683.16, 683.17, 683.30, 683.31, 683.32, 683.33, 683.34, 683.35, 683.36, 683.37, 683.51, 683.52, 683.53, 683.54, 683.56, 683.57, 683.58, 683.59, 683.60, 683.61, 683.62, 683.63, 683.65, 683.66, 683.80, 683.81, 683.82, 683.83, 683.84, 683.87, 683.88, 683.89, 683.90, and 683.91) are not effective until OMB approval has been obtained and the public has been notified to that effect through a Notice published in the **Federal Register.**

Regulatory Flexibility Act Certification

The Secretary certifies that these regulations will not have a significant economic impact on a substantial number of small entities. Participating guarantee agencies and lending institutions in the insured loan program are reimbursed for their costs.

Certain reporting, recordkeeping, and compliance requirements are imposed on institutions of higher education. These regulations, however, are modeled on existing GSLP regulations and will not have a significant economic impact on these institutions because the additional burdens will be minimal.

List of Subjects in 34 CFR Part 683

Administrative practice and procedure, Education, Loan Programs—Education, Parent aid, Student aid, Vocational education.

Citation of Legal Authority

A citation of statutory authority for the regulations is placed in parentheses on the line following each substantive provision of these regulations.

Dated: April 15, 1982.

(Catalog of Federal Domestic Assistance (No. 84.032, PLUS Program))

T. H. Bell,

Secretary of Education.

The Secretary adds Part 683 of Title 34 of the Code of Federal Regulations, with the exception of § 683.10 issued January 21, 1981, 46 FR 6329, which is revised to read as follows:

PART 683—PLUS PROGRAM

Subpart A—Purpose and Scope

Sec.
683.1 The Plus program.
683.2 Guarantee agency programs.
683.3 The Federal PLUS program.
683.4 Applicability of subparts of this regulation.

Subpart B—General Provisions

683.10 General definitions.
683.11 Borrower and student eligibility.
683.12 Statement of educational purpose.
683.13 Permissible charges to borrowers.
683.14 Special allowance payments to lenders.
683.15 Prohibited transactions.
683.16 Provision of loan information by lenders.
683.17 Treatment of refunds by lenders.

Subpart C—Guarantee Agency Programs

683.30 Agreements between a guarantee agency and the Secretary.
683.31 Basic PLUS program agreement.
683.32 Death, disability, and bankruptcy payments.
683.33 Applicability of GSLP Federal advance funds provisions.
683.34 Federal reinsurance agreement.
683.35 Supplemental Federal reinsurance.
683.36 Administrative cost allowances for guarantee agencies.
683.37 Records, reports, and inspection requirements for guarantee agency programs.

Subpart D—Federal PLUS Program

683.50 Circumstances under which loans may be insured.
683.51 Extent of Federal insurance.
683.52 Application to become a lender under the Federal PLUS program.
683.53 The lender insurance contract.
683.54 Issuance of Federal loan insurance.
683.55 Limitations on maximum loan.
683.56 Insurance premiums.
683.57 Repayment of loans.
683.58 Deferment.
683.59 Due diligence in making and disbursing a loan.
683.60 Due diligence in collecting a loan.
683.61 Forbearance.
683.62 Assignment of a loan.
683.63 Death, disability, and bankruptcy.
683.64 Cessation of lender collection activity in certain cases.
683.65 Procedures for filing claims.
683.66 Determination of amount of loss on claims.
683.67 The Secretary's collection efforts after payment of a default claim.
683.68 Records, reports, and inspection requirements for lenders.

Subpart E—Requirements, Standards, and Payments for Participating Schools

683.80 Participation agreement between an eligible school and the Secretary for participation in the PLUS Program.
Sec.
683.81 Agreement between the Secretary and a school that makes or originates PLUS loans.
683.82 Providing information to prospective students.
683.83 Correspondence school schedule requirements.
683.84 Certifications by a participating school in connection with a PLUS loan application.
683.85 (Reserved).
683.86 (Reserved).
683.87 Refund policy.
683.88 Determining the date of a student's withdrawal.
683.89 Payment of a refund to a lender.
683.90 Termination of a school's lending eligibility.
683.91 Records, reports and inspection requirements for participating schools.

Subpart F—Limitation, Suspension, or Termination of Lender Eligibility Under the Federal PLUS Program

683.100 Purpose and scope.
683.101 Definitions of terms used in this subpart.
683.102 Effect on prior participation.
683.103 Informal compliance procedure.
683.104 Emergency action.
683.105 Suspension proceedings.
683.106 Limitation or termination proceedings.
683.107 Initial and final decisions.
683.108 Verification of mailing dates.
683.109 Effect of suspension or termination proceeding.
683.110 Limitation.
683.111 Reimbursements, refunds, and offsets.
683.112 Reinstatement after termination.
683.113 Removal of limitation.
Appendix A—Standards for Acceptable Refund Policies by Participating Schools.

Authority: Title IV, Part B, of the Higher Education Act of 1965, as amended (20 U.S.C. 1071 to 1087-2), unless otherwise noted.

Subpart A—Purpose and Scope

§ 683.1 The PLUS program.

(a) The PLUS program makes loans available to independent undergraduate students, graduate and professional students, and the parents of dependent undergraduate students. Each borrower must use the loan funds to pay for the student's educational costs. Lenders loan their own funds, and the Federal Government or a guarantee agency insures against loss. The program has two parts: guarantee agency programs and the Federal PLUS program.

(1) State agencies or private nonprofit agencies guarantee loans and are reimbursed by the Secretary for part or all of the insurance claims they pay to lenders. Guarantee agency programs must meet certain Federal requirements, but there may be variation among programs in such areas as the loan maximums and the borrower's or the student's eligibility.

(2) The Federal PLUS program operates in States not served by guarantee agencies and in certain prescribed circumstances in which a guarantee agency program does not authorize loans to serve eligible PLUS borrowers in a State. The Secretary directly insures lenders against losses on Federal PLUS loans.

(b) *Participation in the PLUS program.* (1) Banks, savings and loan associations, credit unions, pension funds, insurance companies, schools, State agencies and, in certain instances, the Student Loan Marketing Association may be lenders. The Student Loan Marketing Association and some State agencies purchase and hold loans and function as secondary markets.

(2) Most colleges and universities, and many vocational, technical, and correspondence schools are eligible to participate as educational institutions.

(3) Information for parents and students about the PLUS program and the Guaranteed Student Loan program (GSLP) is available on request from the Department of Education.

(4) All lenders, schools, parent borrowers, and students must meet certain requirements in order to participate in the PLUS program. These regulations contain all of the eligibility requirements for the Federal PLUS program and Federal eligibility requirements for participation in guarantee agency programs. Each guarantee agency may establish additional requirements within these Federal limits.

(c) *Repayment.* A borrower who obtains a loan under the PLUS program is obligated to repay the lender the full amount borrowed, plus interest. Unlike the interest on a student loan under the GSLP, interest on a PLUS loan is not subsidized by the Secretary, and the borrower must begin payment on the loan within 60 days after the loan is disbursed. In some cases repayment may be deferred for a time, but the borrower is still responsible for repaying the entire loan amount plus interest. The borrower's obligation to repay is cancelled only if he or she dies or becomes totally and permanently disabled or if the loan is discharged in bankruptcy.

(d) *Default.* If a borrower defaults on a loan, the Secretary or the guarantee agency pays the lender the amount of its loss. The borrower then owes the debt to the Secretary or the guarantee agency. The Secretary or guarantee agency actively attempts to collect the debt.

(20 U.S.C. 1071 to 1087-2)

§ 683.2 Guarantee agency programs.

(a) The Secretary pays special allowance to lenders on guarantee agency loans. The Secretary also pays a PLUS borrower's loan obligation if the borrower dies, or becomes totally and permanently disabled, or if the loan is discharged in bankruptcy.

(b) The Secretary pays 80 percent of the guarantee agency's default losses under a reinsurance agreement. If the guarantee agency meets additional requirements, the Secretary pays up to 100 percent of the agency's default losses, depending on its default experience.

(c) The Secretary encourages State and private nonprofit guarantee agencies to establish PLUS programs. Federal loan advances are available to help start or strengthen an agency's reserve fund, which backs its PLUS and GSLP loan guarantees. Administrative cost allowances based upon the dollar amount of the GSLP and PLUS loans guaranteed are also available to the agencies.

(d) To administer a PLUS program and to qualify itself and lenders for these benefits, an agency must meet the requirements under subparts B, C, and E.

(20 U.S.C. 1071, 1072, 1078-1, 1078-2, 1082, 1087, 1087-1)

§ 683.3 The Federal PLUS program.

(a) *Where does the Federal PLUS program operate?* The specific conditions under which the Federal PLUS program may operate in a State are given in § 683.50. In general, the Federal PLUS program is available to all lenders in a State if there is no guarantee agency program in that State which insures PLUS loans.

(b) *Payments to lenders.* Lenders qualify for the payment of special allowance on Federal PLUS loans. The Secretary pays a borrower's loan obligation if the borrower dies or becomes totally and permanently disabled or if the loan is discharged in bankruptcy. The Secretary also pays the lender's insurance claim if the borrower defaults.

(c) To qualify for Federal insurance and special allowance benefits, the lender must meet certain requirements established by law and these regulations.

(20 U.S.C. 1071 to 1087-2)

§ 683.4 Applicability of subparts of this regulation.

Subpart B contains general provisions that are applicable to all PLUS program participants. In addition, guarantee agency programs are subject to subparts C and E, and the Federal PLUS program is subject to subparts D, E, and F. Schools are governed by subpart E.

(20 U.S.C. 1071 to 1087-2)

Subpart B—General Provisions

§ 683.10 General definitions.

Academic year: (a) A period of time, typically eight or nine months, in which a full-time student is expected to complete the equivalent of at least two semesters, two trimesters or three quarters at a school using credit hours; or

(b) At least 900 clock hours of training for a program at a school using clock hours; or

(c) Eighteen months for a correspondence program.

Act: Title IV, Part B of the Higher Education Act of 1965, as amended (20 U.S.C. 1071 *et seq.*).

Borrower: A graduate or professional student, an independent undergraduate student, or one or more of a dependent undergraduate student's parents to whom a PLUS loan is made. If the loan is made to two eligible parents as co-makers, each parent is liable for repayment of the entire amount of the loan, including interest, and the loan is eligible for deferment, cancellation, or forbearance benefits only if both parents qualify for such benefits.

Clock hour: A period of time that is the equivalent of—

(a) A 50 to 60 minute class, lecture, or recitation; or

(b) A 50 to 60 minute faculty-supervised laboratory, shop training, or internship.

Commercial lender: A commercial bank, savings and loan association, credit union, or mutual savings bank.

Default: The failure of a borrower to make an installment payment when due, or to meet other terms of the promissory note under circumstances where the Secretary or the pertinent guarantee agency finds it reasonable to conclude that the borrower no longer intends to honor the obligation to repay, provided that this failure persists for—

(a) 120 days for a loan repayable in monthly installments; or;

(b) 180 days for a loan repayable in less frequent installments.

Dependent student: A student who is not an independent student.

Disbursement: The transfer of funds by a lender to a borrower by means of issuing a check or draft payable to the order, and requiring the personal endorsement, of the borrower.

Due diligence: The utilization by a lender in the making and collection of PLUS loans of practices at least as

extensive and forceful as those generally practiced by financial institutions for consumer loans. The procedures for establishing due diligence under the Federal PLUS program are described in § 683.59 and § 683.60. The procedures for establishing due diligence under a guarantee agency program are set forth by the guarantee agency.

Endorser: An individual who is secondarily liable for the loan obligation.

Enrolled: The status of a student who—

(a) Has completed the registration requirements at the school he or she is attending and has commenced the attendance period; or

(b) Has been admitted into a correspondence study program and has submitted one lesson, completed by him or her after acceptance for enrollment and without the help of a representative of the school.

Estimated cost of attendance: (a) Except as provided in paragraph (b) of this definition, the tuition and fees applicable to a student, plus the school's estimate of other expenses reasonably related to attendance at that school for the period for which the loan is sought. These costs include, but are not limited to, reasonable transportation and commuting costs, costs for room, board, books, and supplies, the insurance premium for the loan, and, if the student has applied for a GSLP loan for the same academic period, the origination fee and insurance premium for that loan.

(b) For a student enrolled in a correspondence study program, only the insurance premium for the loan and the contract price of the program, and, if the student has applied for a GSLP loan for the same academic period, the origination fee and insurance premium for that loan. However, costs described in paragraph (a) of this definition that are incurred by the student for fulfilling a required period of residential training in connection with the correspondence study program may also be included in the estimated cost of attendance.

Estimated financial assistance: For the period for which a loan is sought, the estimated amount of assistance that a school is aware a student has been or will be awarded in Federal, State, or privately supported scholarship, grant, work, or loan programs. Any other PLUS loans applied for by the student or the student's parents on that student's behalf are considered financial assistance. The following are also considered financial assistance:

(a) Veterans' benefits paid under chapters 32, 34 and 35 of Title 38 of the United States Code.

(b) Students' benefits under Social Security.

Resources or financial support from the student or the student's family may not be considered financial assistance.

Federal Insured Student Loan Program (FISLP): The component of the GSLP under which the Secretary directly insures loans, with no participation by guarantee agencies.

Full-time student: (a) A student enrolled in an institution of higher education (other than a correspondence school) who is carrying a full-time academic workload as determined by the school, under standards applicable to all students enrolled in that student's particular program. The student's workload may include any combination of courses, work experience, research, or special studies, whether or not for credit, that the school considers sufficient to classify the student as a full-time student; or

(b) A student enrolled in a vocational school (other than a correspondence school) who is carrying a workload of not less than 24 clock hours per week or 12 semester or quarter hours of instruction, or its equivalent.

Graduate or professional student: A student who—

(a) Is pursuing a program, or has a bachelor's degree and is enrolled in courses which are normally part of a program, leading to a graduate or professional degree or certificate at an institution of higher education; and

(b) Has successfully completed the equivalent of at least three years of full-time study at an institution of higher education either prior to entrance into the program or as part of the program itself.

Guarantee agency: A State or private nonprofit agency that administers a loan insurance program under the Act.

Guaranteed Student Loan program (GSLP): A loan program as described in 34 CFR Part 682 in which the Federal Government insures or reinsures low-interest, long-term educational loans to eligible students. Loans are made under the GSLP by eligible lending institutions, such as commercial lenders, pension funds or insurance companies.

(a) Under the Federal Insured Student Loan program (FISLP), loans are directly insured by the Federal Government.

(b) Under guarantee agency programs, loans are insured by State agencies or private nonprofit agencies and are re-insured by the Federal Government.

Half-time student: An enrolled student who is carrying a half-time academic workload, as determined by the school, and that amounts to at least one-half the workload of a full-time student. A student enrolled solely in an eligible program of study by correspondence is considered a half-time student.

Holder: An eligible lender in possession of a PLUS loan.

Incentive payment: A payment to a lender that is made for the purpose of encouraging the lender to make one or more PLUS loans.

Independent student: (a) For a period of instruction which begins prior to July 1, 1982, a student who for 1980 and 1981—

(1) Has not lived and will not live for more than six weeks in each year in the home of his or her parent(s);

(2) Has not been claimed and will not be claimed as an exemption for Federal income tax purposes by his or her parent(s);

(b) For a period of instruction beginning on or after July 1, 1982, but before July 1, 1983—

(1) A single student who for 1981 and 1982—

(i) Has not lived and will not live for more than six weeks in either year in the home of his or her parent(s);

(ii) Has not been claimed and will not be claimed as a dependent for Federal income tax purposes by his or her parent(s); and

(iii) Has not received and will not receive financial assistance of more than $750 in either year from his or her parent(s); or

(2) A married student who for 1982—

(i) Has not lived and will not live for more than six weeks in the home of his or her parent(s);

(ii) Has not been claimed and will not be claimed as a dependent for Federal income tax purposes by his or her parent(s); and

(iii) Has not received and will not receive financial assistance of more than $750 from his or her parent(s).

(3) If a student's mother and father are divorced or separated, only one parent will be considered to be the parent of the student for purposes of applying the criteria in paragraphs (1) and (2) of this definition. To determine that parent—

(i) Choose the parent with whom the student resided for the greater portion of the 12 month period preceding the date of application.

(ii) If the preceding criterion does not apply, choose the parent who provided the greater portion of the student's support for the 12 month period preceding the date of application.

(iii) If neither of the preceding criteria apply, choose the parent who provided the greater support for the period commencing January 1 of the calendar year which immediately precedes the first calendar year of the period of

instruction and ending 12 months prior to the date of applications.

(4) If either of the parents have died, the institution shall consider only the surviving parent as the parent for purposes of applying the criteria in paragraphs (1) and (2) of this definition. If both parents have died, the institution shall not consider either parent.

Institution of higher education:

(a) An institution that—

(1) Is in a State;

(2) Admits as a regular student only a person who—

(i) Has a certificate of graduation or its equivalent from a secondary school; or

(ii) Is beyond the age of compulsory school attendance;

(3) Is legally authorized in each State in which it is physically located to provide, and provides within that State, a program of postsecondary education that—

(i) Awards a bachelor's degree; and

(ii) Provides not less than a two-year program which is acceptable for full credit toward such a degree;

(4) Is a public or other nonprofit institution; and

(5)(i) Is accredited by a nationally recognized accrediting agency or association approved by the Secretary for this purpose; or

(ii) If not so accredited—

(A) Is an institution which the Secretary determined will meet the accreditation standards of such an agency or association within a reasonable period of time; or

(B) Is an institution whose credits are transferrable, for credit on the same basis, to at least three institutions that are so accredited; or

(iii) If the Secretary determined that there is no nationally recognized accrediting agency or association qualified to accredit the type of school applying for eligibility, is approved by an advisory committee that the Secretary established, in accordance with the standards of content, scope and quality that the Committee prescribes for that purpose.

(b) The term also includes an institution that provides not less than a one-year program of training to prepare students for gainful employment in a recognized occupation and, with the exception of paragraph (a)(3), meets the other requirements of paragraph (a) of this section.

(c) The Secretary publishes a list of nationally recognized accrediting agencies or associations that the Secretary has determined to be reliable authorities as to the quality of education or training offered.

Legal guardian: An individual who is appointed by a court as a "legal guardian" of a person and who is specifically required by the court to use his or her own financial resources to support that person.

Lender: A lender, including a subsequent holder, that is—

(a) A National or State-chartered bank, a mutual savings bank, a savings and loan association, or a credit union that—

(1) Is subject to examination and supervision in its capacity as a lender by an agency of the United States or of the State in which its principal place of operation is established; and

(2) Does not make or hold loans to students and parents under the GSLP and the PLUS program that total more than one-half of its consumer credit loan dollar volume, including home mortgages, unless it is a bank that is wholly owned by a State; or

(b) A pension fund as defined in the Employees Retirement Income Security Act; or

(c) An insurance company that is subject to examination and supervision by an agency of the United States or a State; or

(d) In any State, a single agency of the State or a single private nonprofit agency designated by the State; or

(e) For purposes only of purchasing and holding loans made by other lenders under this program, the Student Loan Marketing Association or an agency of any State functioning as a secondary market; or

(f) For purposes of making loans of last resort, to the extent authorized, under Section 428(h) or Section 439(q) of the Act—

(1) The Student Loan Marketing Association; or

(2) A State or private nonprofit guarantee agency; or

(3) A single agency of a State or a single non-profit private agency designated by a State; or

(4) An agency of a State functioning as a secondary market; or

(g) A participating school that—

(1) Is not a correspondence school. An eligible school that offers both correspondence study and noncorrespondence study programs may be an eligible lender only for students or parents of students enrolled in the noncorrespondence study programs; and

(2) Employs at least one full-time financial aid administrator.

National of the United States: (a) A citizen of the United States.

(b) A person who, though not a citizen of the United States, owes permanent allegiance to the United States.

Origination: A special relationship between a school and a lender, in which the lender delegates to the school substantial functions or responsibilities normally performed by lenders before making loans. In this situation, the school is considered to have "originated" a loan made by the lender. The Secretary determines that "origination" exists if—

(a) A school determines who will receive a loan and the amount of the loan; or

(b) The lender has the school verify the identity of the borrower or complete forms normally completed by the lender.

Parent: A person's mother or father or legal guardian. An adoptive parent is considered to be the person's mother or father.

Participating school: A school that has entered into an agreement with the Secretary under § 683.80 to participate in the PLUS program.

Post-deferment grace period: For a loan made prior to October 1, 1981, the six-month period following a deferment period during which time payments of principal and interest are suspended.

School: (a) An educational institution that is—

(1) An institution of higher education or a vocational school; or

(2) With respect to students who are nationals of the United States, a school outside the United States that is comparable to an institution of higher education or to a vocational school and that has been approved by the Secretary for purposes of the PLUS program.

(b) The term includes only those individual units or programs within a school that have been determined by the Secretary to meet all the requirements for school eligibility.

(c) A school that employs or uses commissioned salespersons to promote the availability of the GSLP or the PLUS program is not eligible to participate in either program. For this purpose—

(1) A "commissioned salesperson" is one who receives compensation in any form or amount that is related to, or calculated on the basis of, student applications for enrollment, student enrollments, or student acceptances for enrollment; and

(2) "Promote the availability" means provide prospective or enrolled students or their parents with application forms, names of eligible lenders or other information designed to encourage the students or the parents of eligible students to seek GSLP or PLUS loans. This term does not include providing general financial aid information to prospective or enrolled students or their parents.

School lender: Any participating school that has been approved as a lender and has entered into a contract of insurance under the Federal PLUS program or a guarantee agency PLUS program.

State lender: In any State, a State agency or a single, private nonprofit agency designated by the State that has been approved as a lender and has entered into a contract of insurance under the Federal PLUS program or a guarantee agency PLUS program.

Totally and permanently disabled: Unable to engage in any substantial gainful activity because of a medically determinable impairment that is expected to continue for a long and indefinite period of time or to result in death.

Undergraduate student: A student who is not a graduate or professional student.

Vocational school: (a) A business or trade school, or technical institution, or other technical or vocational school that—

(1) Is in a State;

(2) Admits as a regular student only a person who—

(i) Has completed or left elementary or secondary school; and

(ii) Has the ability to benefit from the training offered by the school.

(3) Is legally authorized in each State in which it is physically located to provide, and provides within that State, a program of postsecondary vocational or technical education that—

(i) Is designed to provide occupational skills more advanced than those generally offered at the high school level and to fit individuals for useful employment in recognized occupations; and

(ii) Provides no less than 300 clock hours of classroom instruction or its equivalent, or in the case of a program offered by correspondence, requires not less than an average of 12 hours of preparation per week over each 12-week period and completion in not less than 6 months; and

(iii) In the case of a flight school program, maintains current valid certification by the Federal Aviation Administration;

(4) Has been in existence for 2 years or has been specially determined by the Secretary to be a school meeting the other requirements of this paragraph and to be eligible to participate in the PLUS program; and

(5)(i) Is accredited by a nationally recognized accrediting agency or association recognized by the Secretary for this purpose; or

(ii) In the case of a public institution offering postsecondary vocational education, is approved by a State approval agency recognized by the Secretary for this purpose; or

(iii) If the Secretary determines that there is no nationally recognized accrediting agency or association qualified to accredit the type of school applying for eligibility, is approved by a State approval agency recognized by the Secretary for this purpose; or

(iv) If the Secretary determines that there is no nationally recognized accrediting agency or association or State approval agency qualified to accredit or approve the type of school applying for eligibility, is approved by the National Advisory Committee on Accreditation and Institutional Eligibility, in accordance with the standards of content, scope, and quality that the Committee prescribes for that purpose. A school that has been approved by the Committee must, in order to remain an eligible school, become accredited within 3 years after the Secretary has designated a nationally recognized accrediting or State approval agency for the type of school applying for eligibility.

(b) For the purpose of this definition, the Secretary publishes a list of nationally recognized accrediting agencies or associations and State approval agencies that the Secretary has determined to be reliable authorities as to the quality of education or training offered.

(20 U.S.C. 1071 to 1087-2; 1088 to 1094)

§ 683.11 Borrower and student eligibility.

(a) *Eligible parent borrower.* A parent, as defined in § 683.10, is eligible to receive a PLUS loan if the parent—

(1) Is borrowing to pay for the educational costs of an eligible student, set forth in paragraph (c) of this section;

(2) Meets one of the qualifications pertaining to citizenship and residency status, set forth in paragraph (e) of this section; and

(3) Is eligible under the provisions concerning defaults and over payments, set forth in paragraphs (f) and (g) of this section.

(b) *Eligible student borrower.* An independent undergraduate student or a graduate or professional student, as defined in § 683.10, is eligible to receive a PLUS loan if he or she—

(1) Is enrolled or accepted for enrollment on at least a half-time basis in a participating school;

(2) Is eligible under provisions concerning enrollment set forth in paragraph (d) of this section;

(3) Meets one of the qualifications pertaining to citizenship and residency status, set forth in paragraph (e) of this section, and

(4) Is eligible under the provisions concerning defaults and overpayments, set forth in paragraphs (f) and (g) of this section.

(c) *Eligible student.* A student is eligible to have his or her parent borrow a PLUS loan to pay for his or her educational costs if he or she—

(1) Is a dependent student;

(2) Is enrolled or accepted for enrollment as an undergraduate student on at least a half-time basis in a participating school;

(3) Is eligible under the provisions concerning enrollment set forth in paragraph (d) of this section;

(4) Authorizes the school to return any refund of school charges attributable to the PLUS loan to the lender;

(5) Meets one of the qualifications pertaining to citizenship and residency status, set forth in paragraph (e) of this section; and

(6) Is eligible under the provisions concerning defaults and overpayments, set forth in paragraphs (f) and (g) of this section.

(d) *Enrollment status.* To be eligible to borrow or to have his or her parents borrow, a student must meet the following requirements—

(1) If currently enrolled, is maintaining satisfactory progress, as determined by the school;

(2) If enrolled or accepted for enrollment in a vocational school, is attending neither elementary nor secondary school and has the ability to benefit from the training offered, as required under the definition of "vocational school" in § 683.10;

(3) If enrolled or accepted for enrollment in a school outside the United States, is a national of the United States;

(4) If enrolled in a flight school program at a vocational school or an institution of higher education, meets the additional requirements in paragraph (h) of this section.

(e) *Citizenship and residency status.* Each borrower and each student for whom a parent is borrowing, must be one of the following:

(1) A citizen of the United States.

(2) A national of the United States.

(3) A permanent resident of the United States.

(4) In the United States for other than a temporary purpose and able to provide evidence from the Immigration and Naturalization Service of his or her intent to become a permanent resident.

(5) A permanent resident of the Trust Territory of the Pacific Islands or the Northern Mariana Islands.

(f) *Effect of default on eligibility.* (1) Except as provided in paragraph (f)(4) of

this section, a parent is ineligible to borrow a PLUS loan if the parent is in default on a National Defense or Direct Student Loan (NDSL) or a GSLP or PLUS loan received to cover costs of attendance at the school in which the student for whom the parent is borrowing is enrolled or accepted for enrollment.

(2) Except as provided in paragraph (f)(4) of this section, a dependent undergraduate student is ineligible to have a parent borrow on his or her behalf if the student is in default on an NDSL, GSLP or PLUS loan received to cover costs of attendance at the school in which the student is enrolled or accepted for enrollment.

(3) Except as provided in paragraph (f)(4) of this section, an independent undergraduate or a graduate or professional student is ineligible to borrow a PLUS loan if the student is in default on an NDSL, GSLP or PLUS loan received to cover costs of attendance at the school in which the student is enrolled or accepted for enrollment.

(4) In a situation described in paragraph (f)(1), (f)(2) or (f)(3), the borrower may receive a PLUS loan only after the person who is in default has made satisfactory arrangements to repay the defaulted loan. The person who is in default must make arrangements to repay that—

(i) In the case of a default on a Federal PLUS or FISLP loan, are satisfactory to the Secretary;

(ii) In the case of a default on a PLUS or GSLP loan insured by a guarantee agency, are satisfactory to that guarantee agency; or

(iii) In the case of a default on an NDSL loan, are satisfactory to the Secretary or the school.

(5)(i) In determining whether a parent is in default on an NDSL, GSLP, or PLUS loan, a school may rely on that parent's written statement that he or she is not in default, unless the school has information to the contrary.

(ii) In determining whether a student is in default on an NDSL, GSLP, or PLUS loan, a school may rely on that student's written statement that he or she is not in default, unless the school has information to the contrary.

(6) The Secretary does not consider an NDSL, GSLP, or PLUS loan that is discharged in bankruptcy to be in default for purposes of this section.

(g) *Effect of overpayment of a grant on eligibility.* (1) Except as provided in paragraph (g)(2) of this section, a parent is ineligible to borrow a PLUS loan, a student is ineligible to borrow a PLUS loan, and a student is ineligible to have a parent obtain a PLUS loan on the student's behalf, if the parent or student owes a refund on a grant made under Title IV of the Act that was received to cover costs of attendance at the school in which the student is enrolled or accepted for enrollment.

(2) In a situation described in paragraph (g)(1), the borrower may receive a PLUS loan, or a parent may borrow on the student's behalf, under the following conditions:

(i) *Overpayment of a Pell Grant.* If the borrower or student for whom the loan is intended is overpaid on a Pell Grant, that parent or student may still be eligible under the PLUS program if—

(A) The borrower and student are otherwise eligible; and

(B) The overpayment can be eliminated in the award year (as defined in 34 CFR 690.2) in which it occurred by adjusting the subsequent Pell Grant payments for that award year.

(ii) *Overpayment of a Pell Grant due to school error.* If the borrower or student for whom the loan is intended is overpaid as a result of school error, and the overpayment cannot be eliminated by adjusting subsequent Pell Grant payments in the award year, that borrower or student may still be eligible under the PLUS program if—

(A) The borrower and student are otherwise eligible; and

(B) The person who received the overpayment acknowledges in writing the amount of the Pell Grant overpayment and agrees to repay it in a reasonable period of time.

(iii) *Overpayment on a Supplemental Educational Opportunity Grant.* If the borrower or student for whom the loan is intended is overpaid on a Supplemental Educational Opportunity Grant, that borrower or student may still be eligible under the PLUS program if—

(A) The borrower and student are otherwise eligible; and

(B) An adjustment in subsequent financial aid payments (other than Pell Grants) eliminates the overpayment in the same award year (as defined in 34 CFR 676.2) in which it occurred.

(h) *Additional eligibility requirements for a student attending flight school.* A student is eligible to borrow or to have a parent borrow a PLUS loan on his or her behalf for enrollment in a flight school program at a vocational school or an institution of higher education only if that student—

(1) Plans to pursue or is pursuing a full-time program leading to commercial flight ratings;

(2) Has completed ground school training or is taking it concurrently with flight training;

(3) Holds a private pilot's certificate or has sufficient flight hours to qualify for such certificate; and

(4) Holds at least a Class II medical certificate.

(i) For purposes of this Part—

(1) "Overpayment of a grant" means that a borrower or student received payment of a grant greater than the amount he or she was entitled to receive;

(2) A "grant made under Title IV of the Act" means—

(i) A Pell Grant, authorized under Part A, Subpart I of Title IV of the Act;

(ii) A Supplemental Educational Opportunity Grant, authorized under Part A, Subpart 2 of Title IV of the Act; and

(iii) A State Student Incentive Grant, authorized under Part A, Subpart 3 of Title IV of the Act.

(3) A "National Defense Student Loan" (NDSL) means a loan made under Title II of the National Defense Education Act; and

(4) A "National Direct Student Loan" (NDSL) means a loan made under Part E of Title IV of the Act.

(20 U.S.C. 1077, 1078, 1078–2, 1091)

§ 683.12 Statement of educational purpose.

(a) No loan may be insured under this program unless the borrower declares in a written statement that the loan proceeds will be used solely for costs of attendance at the school that the borrower or the student on whose behalf the parent is borrowing is or will be attending.

(b) The statement must be in a form approved by the Secretary.

(c) The borrower must file his or her statement with the lender. The lender shall retain a copy of the statement as required in § 683.68 or by a guarantee agency that insures the loan.

(20 U.S.C. 1078–2, 1082, 1091).

§ 683.13 Permissible charges to borrowers.

(a) *Interest*—(1) *Rate.* (i) Exclusive of any insurance premium, and unless affected by section 427A(c)(2) of the Act, a lender may charge a maximum interest rate of 14 percent per year on the unpaid principal balance of a PLUS loan.

(ii) The unpaid principal balance of a loan may include capitalized interest under circumstances described in paragraph (a)(3) of this section.

(2) *Method of calculation.* The lender shall calculate the interest from the date of disbursement of funds to the borrower. In calculating the interest, the lender may use either of the following methods:

(i) The "Approximate Time-Ordinary Interest" method; or

(ii) The "Exact Time-Exact Interest" method. Use of the "Banker's Rule" ("Exact Time-Ordinary Interest") is prohibited because this method results in an actual rate in excess of the allowable rate of interest.

(3) *Capitalizing interest.* "Capitalization" means increasing the unpaid principal of a loan through the addition of accrued interest to the previously unpaid principal balance.

(i) *Federal PLUS program.* For a PLUS loan insured under the Federal program, a lender may capitalize accrued interest covering any period in which the borrower is unable or is not required to make payments of principal. The lender may add this accrued interest to the borrower's unpaid principal balance when the borrower is scheduled to begin or resume repayment.

(ii) *Guarantee agency PLUS programs.* For a PLUS loan insured under a guarantee agency program, a lender may add accrued interest and unpaid insurance premiums to the borrower's unpaid principal balance as authorized by guarantee agency policy.

(4) *Payment.* Interest is payable by the borrower in installments over the life of the loan. However, a lender may permit a borrower to postpone payment of interest as described in paragraph (a)(3) of this section. This accrued interest either may be paid when payment of principal begins or resumes or may be capitalized.

(b) *Insurance premium.* (1) The term "insurance premium" covers those charges made by the guarantee agency or the Secretary to the lender to insure the lender of a PLUS loan against losses it may suffer if the borrower defaults or files a bankruptcy petition. The insurance premium also may be used by the guarantee agency or the Secretary to cover costs incurred in the administration of the applicable loan insurance program. Premiums may not be retained by the lender to cover the costs of making a loan or for any other purpose.

(2) Specific rules on insurance premiums, including the rate that may be charged the lender and passed on to the borrower and the method of calculation, are contained in § 683.31 for PLUS loans insured under guarantee agency programs, and § 683.56 for Federal PLUS loans.

(c) *Late charges.* To the extent provided in the promissory note and permitted by State law, the lender may require that the borrower pay a late charge if the borrower fails to pay any or all of a required installment payment within 10 days after its due date or fails to provide written evidence that verifies eligibility for authorized deferment of the payment. The late charge may not exceed 5 cents for each dollar of each installment due or $5 for each installment, whichever is less.

(d) *Collection charges*—(1) *Permissible charges.* If provided in the note, the lender also may require that the borrower pay the lender for certain reasonable costs incurred by the lender or its agent in collecting any installment not paid when due. These costs may include attorney's fees, court costs, telegrams, and long-distance phone calls.

(2) *Non-permissible charges.* Other than those authorized by this section, no charges may be passed on to the borrower, either directly or indirectly. Examples of charges that are not permitted are as follows:

(i) Normal collection costs associated with preparing letters or notices or making personal contacts or local telephone calls.

(ii) Fees charged by a servicing or collection or skip-tracing agency, to the extent they exceed permissible charges.

(iii) Loan origination fees.

(20 U.S.C. 1077, 1077a, 1078, 1078-2, 1079, 1082, 1087-1)

§ 683.14 Special allowance payments to lenders.

(a) *General.* (1) The Secretary pays a special allowance to lenders on all PLUS loans. The special allowance is equal to a percentage of the average unpaid principal balance, including capitalized interest, for all PLUS loans a lender has held during a 3-month period. The 3-month periods end (1) March 31; (2) June 30; (3) September 30; and (4) December 31 of each year.

(2) If a lender makes or purchases loans with funds obtained from an Authority issuing obligations, the income from which is exempt from taxation under the Internal Revenue Code of 1954, the Secretary pays the special allowance to that lender on those loans only if the Authority has had a plan for doing business approved by the Secretary in accordance with section 420(b) of the Education Amendments of 1980 (Pub. L. 96-374).

(b) *Lender's reports.* To receive the special allowance payment, a lender shall submit periodic reports to the Secretary stating the average unpaid principal balance for all its PLUS loans. These reports must be in a form prescribed by the Secretary.

(c) *Determining the special allowance rate for loans made prior to October 1, 1981.* The percentage rate for the special allowance for a 3-month period is determined by—

(1) Subtracting 5.5 percent from the average of the bond equivalent rates of the 91-day Treasury bills auctioned during the 3-month period;

(2) Rounding the resulting percent upward to the nearest one-eighth of one percent; and

(3) Dividing the resulting percent by 4.

(d) *Determining the special allowance rate for loans made on or after October 1, 1981.* Unless affected by Section 427A(c)(2) of the Act, the percentage rate for the special allowance for a 3-month period is determined by—

(1) Subtracting 14 percent from the average of the bond equivalent rates of the 91-day Treasury bills auctioned during the 3-month period;

(2) Adding 3.5 percent to the resulting percent; and

(3) Dividing the resulting percent by 4.

(e) *Announcing the rate.* After the close of each 3-month period, the Secretary announces the rate of the special allowance for that period.

(f) *Determining the average unpaid principal balance.* (1) There are two methods a lender may use to determine the average unpaid principal balance for purposes of the special allowance:

(i) *The average quarterly balance method.* Add the unpaid principal balance of all loans outstanding on the first day of the 3-month period to the unpaid principal balance of all loans outstanding on the last day of the period and divide by 2.

(ii) *The average daily balance method.* Add the unpaid principal balance of all loans outstanding on each day of the 3-month period and divide by the number of days in that period.

(2) The lender may not change its method of determining the average unpaid principal balance without the prior written approval of the Secretary.

(3) For the purpose of this determination, a loan is considered outstanding if—

(i) The borrower has not repaid the loan;

(ii) The lender has not received payment on a claim for loss on the loan; and

(iii) The lender has not been advised that the Secretary or a guarantee agency has finally refused a claim for loss on the loan.

(g) *Special allowance rates applicable to loans made or purchased with funds obtained from the issuance of obligations that generate tax-exempt income.* (1) The Secretary pays a lender one-half the special allowance rate computed under paragraph (c) or (d) of this section for PLUS loans, for loans made prior to October 1, 1981, no less than the rate specified in paragraph (g)(2) of this section, if that lender

makes or purchases those loans with funds obtained by the lender—

(i) From the issuance of obligations the income from which is exempt from taxation under the Internal Revenue Code of 1954; or

(ii) From collections, default reimbursements, interest, special allowances or other income obtained from loans made or purchased with funds obtained as described in subdivision (i) of this subparagraph; or

(iii) From the investment of funds obtained as described in subdivisions (i) or (ii) of this subparagraph.

(2) The minimum special allowance the Secretary pays to a lender for PLUS loans described in paragraph (g)(1) of this section, on loans made prior to October 1, 1981, is 0.5 percent per year.

(h) *Payment of penalty interest.* (1) If the Secretary has not authorized the United States Department of the Treasury to pay special allowance within 30 days after receipt of an accurate, timely and complete request for payment from any lender, the Secretary pays that lender an additional amount known as penalty interest.

(2) *Determining the amount of penalty interest.* (i) Penalty interest is the daily interest that accrues on the special allowance payments otherwise due to the lender. That interest is computed at the daily equivalent rate of the sum of the special allowance on a loan for the 3-month period for which the special allowance is being paid plus the interest rate at which the loan was made.

(ii) The Secretary pays penalty interest for—

(A) The 31st day after receipt of the request for payment or the 31st day after the final day of the period (or periods) covered by the request, whichever is later; and

(B) Each succeeding day until the Secretary authorizes payment. The day on which payment is authorized is also counted.

(20 U.S.C. 1078-2, 1082, 1087-1, Pub. L. 96-374, § 420(b))

§ 683.15 Prohibited transactions.

(a)(1) No points, premiums, payments, or additional interest of any kind may be paid or otherwise extended to any eligible lender or other party in order to—

(i) Secure funds for making PLUS loans; or

(ii) Induce a lender to make PLUS loans to either students or the parents of students of a particular school or a particular category of students or their parents.

(2) The following are examples of transactions which, if entered into for the purposes described in paragraph (a)(1)(i) or (ii) of this section, are prohibited:

(i) Cash payments by or on behalf of a school made to a lender or other party.

(ii) The maintaining of a compensating balance by or on behalf of a school with a lender.

(iii) Payments ostensibly made for other purposes.

(iv) Payments by or on behalf of a school to a lender of servicing costs on loans that the school does not own.

(v) Payment by or on behalf of a school to a lender of unreasonably high servicing costs on loans that the school does own.

(vi) Purchase by or on behalf of a school of stock of the lender.

(b) Except when purchased by the Student Loan Marketing Association or an agency of any State functioning as a secondary market or in other circumstances approved by the Secretary, notes, or any interest in notes, shall not be sold or otherwise transferred at discount if the underlying loans were made—

(1) By a school; or

(2) To students or parents of students attending a school by a lender having common ownership with that school.

(c) Except to secure a loan from the Student Loan Marketing Association or an agency of a State functioning as a secondary market or in other circumstances approved by the Secretary, a school, or a lender with respect to a loan made to a student or parent of a student attending a school having common ownership with the lender, may not pledge a loan made under the PLUS program as security for any loan bearing aggregate interest and other charges in excess of the sum of the interest rate applicable to the loan plus the rate of the then most recently prescribed special allowance under § 683.14.

(d) The prohibitions described in paragraphs (a), (b), and (c) of this section apply to any school or lender which would be a party to the prescribed transactions.

(e) If a school has an agreement with the Secretary under § 683.81, the performance by the school of substantial functions or responsibilities normally performed by a lender, which results in the school "originating" loans made by the lender, is not a prohibited transaction.

(f) *Warranty.* (1) Nothing in this section shall preclude a buyer of loans made by a school from obtaining a warranty from the seller of those loans.

(2) The warranty may cover future reductions by the Secretary or a guarantee agency in computing the amount of insurable loss, if any, on default claims filed on the loans where the reductions are attributable to an act or failure to act of the seller or previous holder.

(3) The warranty shall not cover matters for which a purchaser is charged with responsibility under this part, such as due diligence in collecting loans.

(g) Section 490(c) of the Act provides that any person who knowingly and willfully makes an unlawful payment to an eligible lender as an inducement to make, or to acquire by assignment, a PLUS loan, shall upon conviction thereof be fined not more than $1,000 or imprisoned not more than one year, or both.

(20 U.S.C. 1078-2, 1082, 1097)

§ 683.16 Provision of loan information by lenders.

By no later than when the note is signed, the lender shall provide the borrower with thorough and accurate information regarding a loan made under the PLUS program including—

(a) The annual and aggregate maximum amounts that may be borrowed;

(b) The terms on which repayment of the loan will begin;

(c) The maximum number of years in which the loan must be repaid;

(d) The interest rate that must be paid, and the minimum amount of required monthly payment;

(e) Rights or options the borrower may have for deferment, cancellation, prepayment, consolidation, or other refinancing of the loan;

(f) A definition of default and the consequences to the borrower if he or she should default, including a description of any arrangements made with credit bureaus; and

(g) To the extent practicable, the effect that accepting the loan has on the eligibility of the borrower for future borrowing under the PLUS program and on the eligibility of that borrower and, in the case of a parent borrower, that student to receive student financial assistance under the Act.

(20 U.S.C. 1078-2, 1082, 1083a)

§ 683.17 Treatment of refunds by lenders.

(a) A lender shall treat a payment from a school representing a refund to a student who obtained a PLUS loan or whose parent obtained a PLUS loan on his or her behalf from that lender as a credit against the amount owed by the borrower on the PLUS loan.

(b) If a lender receives from a school a refund payment on a loan that is no longer held by that lender, the lender shall—

(1) Transmit the amount of the refund payment to the holder to whom the loan has been assigned with an explanation of the payment's source; and

(2) Provide simultaneous written notice to the borrower that a payment has been transferred to the new holder.

(20 U.S.C. 1078-2, 1082)

Subpart C—Guarantee Agency Programs

§ 683.30 Agreements between a guarantee agency and the Secretary.

(a) The Secretary enters into agreements with a guarantee agency, enabling the agency to participate in the PLUS program, if the Secretary and the guarantee agency have corresponding agreements in effect under the GSLP and the Secretary determines that the guarantee agency program meets the requirements of this subpart. Separate agreements, based on various requirements, are necessary for the agency to receive some or all of the benefits available to it.

(b) *Types of agreements.* There are six agreements. Specific requirements for each agreement, and additional requirements for receiving some benefits, are described in this subpart.

(1) *Basic PLUS program agreement.* A guarantee agency must have a basic agreement to participate in the PLUS program in any way. Under this agreement—

(i) Lenders may receive special allowance and penalty interest payments and, through the guarantee agency, death, disability, and bankruptcy claim payments;

(ii) The guarantee agency may apply for the primary administrative cost allowance, and for the agreements listed below.

(2) *Federal advances for reserve fund agreement.* A guarantee agency must have this agreement to receive and use Federal advances to help establish or strengthen the reserve fund that backs the agency's PLUS loan guarantees.

(3) *Additional Federal advances for claim payments agreements.* A guarantee agency must have this agreement to receive and use Federal advances to pay PLUS program insurance claims.

(4) *Reinsurance agreement.* A guarantee agency must have a reinsurance agreement to receive reimbursement of 80 percent of its losses on default claims.

(5) *Supplemental reinsurance agreement.* A guarantee agency, with this agreement, receives reimbursement of up to 100 percent of its losses on default claims.

(6) *Secondary administrative cost allowance agreement.* A guarantee agency establishes this agreement by applying for and receiving the secondary administrative cost allowance.

(c) *Failure to comply with agreements.* If the Secretary finds that a guarantee agency has made incomplete or incorrect statements in connection with an agreement, or has failed to comply with an agreement or with applicable Federal law or regulations, the Secretary takes actions necessary to protect the interests of the United States. These actions may include—

(1) Withholding payments to the guarantee agency;

(2) Requiring reimbursement of payments; or

(3) Suspending or terminating an agreement.

(d) *Remedial actions.*

(1)(i) The Secretary or the guarantee agency may terminate any agreement upon 60 days written notice.

(ii) The Secretary terminates an agreement only under circumstances described in paragraph (c) of this section.

(iii) Termination does not affect obligations incurred under the agreement before the effective date of the termination.

(2) The Secretary's suspension or termination of an agreement, requirement of reimbursement, or withholding of payments is not final until the guarantee agency has been given reasonable notice of the intended action and an opportunity for a hearing. The Secretary withholds payments or suspends an agreement prior to giving notice and opportunity for a hearing only if the Secretary finds this emergency action necessary to prevent substantial harm to Federal interests.

(e) The Secretary's execution of an agreement does not indicate acceptance of any current or past standards or procedures used by the agency.

(f) All the agreements are subject to subsequent changes in the Act or regulations.

(20 U.S.C. 1072, 1078, 1078-1, 1078-2, 1082, 1087, 1087-1)

§ 683.31 Basic PLUS program agreement.

(a) *General.* (1) The basic agreement is required for all participation by a guarantee agency in the PLUS program. In this agreement, the guarantee agency assures the Secretary that its program meets the requirements of paragraph (b) of this section and agrees to maintain the administrative and fiscal standards of paragraph (c) of this section.

(2) The basic agreement shall contain other provisions and be supported by any material required by the Secretary.

(b) *Program requirements.* The guarantee agency ensures, through its policies and the requirements that it imposes on participating lenders, schools, parents, and students, that its program meets the requirements of this paragraph.

(1) *Aggregate loan limits.* The aggregate insured unpaid principal amount for all PLUS loans made to a borrower may not exceed—

(i) $12,500, including any amounts borrowed under the GSLP, in the case of an independent undergraduate student; or

(ii) $15,000, in the case of a graduate or professional student or a parent borrower on behalf of each eligible student.

(2) *Annual amounts.* (i) The maximum loan amount authorized for any one academic year must be at least $1,000, but may not exceed

(A) $2,500, including any amounts borrowed under the GSLP for the same academic year or its equivalent, in the case of an independent undergraduate student; or

(B) $3,000, in the case of a graduate or professional student or a parent borrower on behalf of each eligible student.

(ii) If the program insures loans to an eligible half-time student borrower or a parent borrower on behalf of an eligible half-time student, the loan maximum must be at least $500 for such borrower for each half-time student in any academic year.

(iii) A guarantee agency does not violate paragraphs (b)(2) (i) or (ii) of this section if it makes the maximum loan amounts listed in those paragraphs applicable to either of the following periods:

(A) A period that does not exceed 12 months; or

(B) A period in which the student on whose behalf the loan is made earns the credits required by the student's school to advance in academic standing, as normally measured on an academic year basis (for example, from freshman to sophomore).

(iv) In no case may the amount of a loan exceed the student's estimated cost of attendance less estimated financial assistance.

(3) *Duration of borrower eligibility.* A parent is eligible to borrow funds on behalf of a dependent undergraduate student in any year of study at a participating school. A graduate or professional student and an independent undergraduate student are eligible to borrow funds in any year of study at a participating school.

(4) *Borrower responsibilities.* (i) The borrower shall promptly notify the lender of any change of his or her name or address.

(ii) The borrower shall give the lender, as part of the loan application process—

(A) The statement, described in § 683.12, that the loan will be used for the cost of the student's attendance;

(B) Information that provides a basis for determining that the borrower is eligible;

(C) Information concerning the borrower's outstanding PLUS, GSLP and NDSL loans and, if the borrower is a parent, information on the outstanding PLUS, GSLP and NDSL loans of the student on whose behalf the parent is borrowing;

(D) A statement from the student which authorizes the school to release information contained in the student's file relevant to the student's eligibility to borrow or to have the parent borrow on his or her behalf (e.g., the student's enrollment status, financial assistance and employment records).

(E) A statement from the student which authorizes the school to return any refund attributable to the PLUS loan to the lender; and

(F) Information from the school that provides a basis for determining that the student qualifies as an eligible student and the maximum amount that may be borrowed by or on behalf of the student.

(5) *Disbursement requirements.* (i) The lender shall disburse the loan funds by means of a check made payable to the borrower or, if authorized by the borrower in writing, jointly to the borrower and the school named on the loan application. The check must require the personal endorsement of the borrower. For this purpose, a check is a draft drawn on a bank and payable on demand, and deposit of the check by the borrower in his or her own account at a bank or other financial institution constitutes endorsement.

(ii) The borrower must personally endorse the check and may not authorize anyone else to endorse it on his or her behalf.

(iii) Neither a lender nor a school may obtain a borrower's power of attorney or other authorization to endorse a check on behalf of a borrower.

(iv) The lender may not disburse loan funds earlier than is reasonably necessary to meet the student's cost of attendance for the period for which the loan is intended.

(6) *School notification requirements.* For each PLUS loan, as a condition of insurance, the school that certified the student's enrollment shall be notified of the insurance, the name of borrower (if the borrower is a parent), the name of the student on whose behalf the loan is made, the amount of the loan, the name of the lender, and the address of the lender. This notification may be made either—

(i) By the lender or the guarantee agency informing the school of these facts no later than 30 days after the initial disbursement of the loan; or

(ii) By the lender sending all loan checks to the school for delivery to the borrower, specifying in each case the name of the student on whose behalf the loan is made.

(7) *Commencement of repayment.* The borrower's repayment period shall begin on the day the loan is disbursed. The borrower's first payment shall be due within 60 days after the loan is disbursed.

(8) *Length of repayment period.* In general, the lender must allow the borrower at least 5 years but not more than 10 years to repay a loan, calculated from the beginning of the repayment period. There are exceptions, however, to this rule;

(i) If the borrower receives a deferment or has been granted forbearance under procedures approved by the guarantee agency, the period of deferment or forbearance is not counted in the 5- and 10-year periods.

(ii) If the minimum annual repayment required in subdivision (iv) of this paragraph would result in complete repayment of the loan in less than 5 years, the borrower is not entitled to the full 5-year period.

(iii) *Prepayment.* The borrower may prepay the whole or any part of the loan at any time without penalty.

(iv) *Minimum annual payment.* During each year of the repayment period, the borrower's payments to all holders of his or her PLUS and GSLP loans must total at least $600 or the unpaid balance of all the loans including interest, whichever amount is less. There are, however, exceptions to this rule:

(A) If the borrower and lender agree, the amount paid may be less.

(B) If the borrower and his or her spouse have separate PLUS loans, or one or more PLUS loans and one or more GSLP loans, their combined annual payment must meet this requirement. The provisions of subparagraphs (A) and (B) may not result in an extension of the 10-year repayment period maximums, unless forbearance has been granted under procedures approved by the guarantee agency.

(9) *Deferment.* During the repayment period, principal payments are postponed during specified periods and under conditions described in § 683.58. However, during these periods, interest shall accrue and be payable unless the lender capitalizes the interest, if permitted by the guarantee agency.

(10) *Interest.* (i) Exclusive of any insurance premium and unless affected by Section 427A(c)(2) of the Act, a lender may charge a maximum interest rate of 14 percent per year on the unpaid principal balance of a PLUS loan. The unpaid principal balance of a loan may include capitalized interest to the extent authorized by the guarantee agency.

(ii) No payments to reduce interest costs shall be paid by the Secretary on PLUS loans.

(11) *Insurance premiums.* (i) The guarantee agency may charge an insurance premium to the lender on each loan. This insurance premium may be used only to insure loans and to cover costs incurred by the guarantee agency in the administration of its PLUS loan insurance program. The lender may pass this charge on to the borrower. The insurance premium may not be used for incentive payments to lenders.

(ii) *Rate.* The insurance premium may not exceed one percent per year of the unpaid principal balance of the loan, excluding interest or other charges that may have been added to the principal.

(iii) *Refund requirement.* The insurance premium need not be refunded by either the guarantee agency or lender to the borrower, even if the borrower prepays, defaults, dies, becomes totally and permanently disabled or files a bankruptcy petition.

(12) *Insurance liability.* The guarantee agency must insure at least 80 percent of the unpaid principal balance of each loan insured.

(13) *Guarantee agency administration.* In the case of a State loan insurance program, the program shall be administered by a single State agency, or by one or more private nonprofit institutions or organizations under the supervision of a single State agency. For this purpose, "supervision" includes setting policies and procedures for, and having full responsibility for, the operation of the program.

(14) *Loan assignment.* A loan may be assigned only to—

(i) An eligible lender; or

(ii) The guarantee agency, in the case of a borrower's default, death, total and permanent disability, or filing of a bankruptcy petition. "Assigned" means any kind of transfer, including transfer as security.

(15) *Loan information.* Each eligible lender shall provide thorough and accurate loan information to borrowers as specified in § 683.16. The lender shall enter into an agreement with the Secretary to ensure that it will provide this information.

(c) *Administrative and fiscal standards required of the guarantee agency.*—(1) *Establishment of procedures.* To enter into a basic PLUS agreement, the guarantee agency shall establish administrative and fiscal procedures that the Secretary may require to ensure proper administration of the agency's loan insurance program.

(2) *Dissemination of standards and procedures.* The guarantee agency shall establish and disseminate to concerned parties its standards and procedures for—

(i) School and lender participation in its program;

(ii) Limitation, suspension, or termination of school and lender participation;

(iii) Approval of forbearance;

(iv) Timely filing of default, death, disability, and bankruptcy claims by lenders; and

(v) Due diligence in making and collecting loans.

(3) *Due diligence.* The guarantee agency shall ensure that due diligence, including resort to litigation as appropriate, will be exercised by lenders in making and collecting loans. The guarantee agency also shall exercise due diligence, including resort to litigation as appropriate, in collecting loans on which default claims have been paid. "Due diligence" is defined in § 683.10.

(20 U.S.C. 1078, 1078-2, 1082; 42 U.S.C. 5055(e))

§ 683.32 Death, disability, and bankruptcy payments.

(a) If a borrower dies or becomes totally and permanently disabled, the Secretary cancels the borrower's obligation on a PLUS loan by paying the lender the amount owed. If a borrower files a petition for bankruptcy, the Secretary pays the amount owed. However, if both parents of a student obtained a loan as co-makers, in order for a loan to qualify for these payments the applicable condition must apply to both parents. The Secretary cancels these loans whether the holder of the loan is a lender or the guarantee agency.

(b) The procedures in § 683.63 concerning death, total and permanent disability, and bankruptcy apply to guarantee agency programs with the following modifications:

(1) The references to the Secretary in §§ 683.63(a)(3) and 683.63(c)(2) shall be understood to mean the guarantee agency if the loan is held by a lender.

(2) References to the Federal PLUS program shall be understood to mean the guarantee agency program.

(3) References to the lender shall be understood to mean the guarantee agency if the loan is held by a guarantee agency.

(c) No death, disability, or bankruptcy claim may be paid if the loan is not considered insurable by the guarantee agency or if a default claim for that loan previously has been disapproved by the guarantee agency.

(d) *Claim procedures for loans held by a lender*—(1) *Claim submission.* (i) The lender shall submit evidence to the guarantee agency that the borrower has died, has become totally and permanently disabled, or has filed a bankruptcy petition. The agency shall return to the lender any submission that is not accurate and complete.

(ii) After determining that a claim is valid the guarantee agency may pay the lender the amount authorized by paragraph (d)(2) of this section. The Secretary periodically reimburses the guarantee agency for these payments.

(2) *Amount of claim payment.* The Secretary determines the amount of the loss to be paid the lender according to the standards used to determine claim payments under the Federal PLUS program. These are found in § 683.66 (a) and (b), with the following modifications:

(i) References to Federal PLUS program insurance shall be understood to mean guarantee agency insurance.

(ii) Paragraph (b)(1)(i) of § 683.66 shall be understood to mean the period prescribed by the guarantee agency.

(iii) References to the Secretary shall be understood to mean the guarantee agency in paragraphs (b)(1)(ii) and (iii) of § 683.66.

(e) *Claim procedures for loans held by the guarantee agency.*

(1) The Secretary pays a death, disability, or bankruptcy claim on a loan held by the guarantee agency after payment of a default claim to the lender only if—

(i) The borrower (in the case of co-makers, both parents), dies, becomes totally and permanently disabled, or files a bankruptcy petition within 10 years of the date the loan went into repayment, exclusive of periods of deferment or periods of forbearance granted by the lender that extend the 10-year period;

(ii) The guarantee agency has not written off the loan as uncollectible; and

(iii) The guarantee agency exercised due diligence in the collection of the loan until the borrower died, became totally and permanently disabled, or filed a bankruptcy petition.

(2) *Amount of claim payment.* (i) The Secretary pays the guarantee agency the amount owed on the loan, including accrued interest. The Secretary pays interest that accrues for a period of up to 60 days from the date the guarantee agency determines that the borrower is dead or totally and permanently disabled, or has filed a bankruptcy petition until the guarantee agency submits the claim to the Secretary. The amount of the payment is reduced by the amount of any reinsurance claim paid by the Secretary for the loan, less any subsequent reimbursement to the Secretary from amounts collected from or on behalf of the borrower.

(ii) If the guarantee agency receives any payments from or on behalf of the borrower on a loan on which the Secretary paid a bankruptcy claim, the guarantee agency shall submit 100 percent of these payments to the Secretary.

(3) If a loan that the Secretary has paid as a bankruptcy claim under this paragraph is not discharged in bankruptcy it will be treated as a default. The guarantee agency shall pay to the Secretary the difference between the amount received from the Secretary as a bankruptcy claim and the amount it would have received as a default claim. In determining the difference, the guarantee agency shall take into account any payments made by or on behalf of the borrower that the agency would have retained on a default claim but submitted to the Secretary under paragraph (e)(2)(ii) of this section.

(20 U.S.C. 1078-2, 1082, 1087)

§ 683.33 Applicability of GSLP Federal advance funds provisions.

(a) Funds appropriated under Section 422 of the Act may be used for purposes of the PLUS program under the terms and conditions specified in § 682.403 and § 682.404 of the GSLP regulations.

(b) The Secretary considers both PLUS loans and GSLP loans in determining the amount of advance funds an agency is eligible to receive under Section 422(c)(2) of the Act.

(c) A guarantee agency shall include both PLUS loans and GSLP loans in making the calculation required under § 682.404(e)(3) of the GSLP regulations.

(20 U.S.C. 1072, 1078-2, and 1082)

§ 683.34 Federal reinsurance agreement.

(a) The Secretary may enter into a reinsurance agreement with a guarantee agency that has a basic PLUS program agreement. Under a reinsurance agreement, the Secretary will reimburse the guarantee agency for 80 percent of its losses on PLUS loans. This agreement is a prerequisite for the supplemental reinsurance agreement, under which the Secretary reimburses the guarantee agency for up to 100 percent of its losses.

(1) *Definition of losses.* In this section, "losses" means the amount the agency pays a lender for a default claim minus payments made by, or on behalf of, the borrower after the lender's claim is paid and before the Secretary reimburses the agency. Losses may include unpaid principal and accrued interest.

(2) *Exclusion.* Death and disability claims on loans are not covered by the reinsurance agreement. Claims on loans to borrowers who have filed a bankruptcy petition also are not covered. Those claims are paid under § 683.32.

(b) The Secretary will enter into a reinsurance agreement only if the agreement would be consistent with any State laws or regulations, and with agreements between lenders and the guarantee agency, regarding the maintenance of the guarantee agency's reserve fund.

(c) The Secretary may find that there is a Federal interest in other aspects of the guarantee agency's operations and may review those operations in deciding whether to enter into or extend a reinsurance agreement.

(d) In deciding whether to enter into a reinsurance agreement, or, if an agreement has terminated, whether to make a subsequent agreement, the Secretary may consider the adequacy of—

(1) The lenders' and the guarantee agency's efforts to collect defaulted loans; and

(2) The guarantee agency's efforts to provide PLUS loans for all eligible borrowers.

(e) Losses on loans that are covered by a reinsurance agreement and were outstanding when the reinsurance agreement was entered into are covered by the agreement only if the default occurs after that time or, if later, after the effective date of the agreement.

(f) *Terms and conditions.* The agreement must contain terms and conditions that the Secretary finds necessary to promote the purposes of the PLUS program and to protect the United States from unreasonable loss, including the following terms and conditions:

(1) The guarantee agency shall assure the Secretary that, for every reinsurance claim it submits—

(i) The terms of the loan comply with all Federal requirements;

(ii) All reasonable efforts have been made by the lender that submitted the default claim to collect the loan;

(iii) The loan was in default before the lender was paid for the claim; and

(iv) The agency will make all reasonable efforts to collect the loan after the Secretary pays the reinsurance claim.

(2) The Secretary prescribes the documentation required to receive payment, and the manner in which payment is made. The Secretary may subtract amounts owed by the guarantee agency from amounts owed to the guarantee agency.

(3) An amount equal to each reinsurance payment shall be credited promptly by the agency to its reserve fund.

(4) Payments made by the borrower to the guarantee agency on a defaulted loan after the Secretary has paid a reinsurance claim on that loan may be applied first to reduce either the principal or interest owed. The borrower's payments may be applied to other charges, such as late charges or attorney's fees, only after the repayment of all principal and interest. If the borrower's repayment schedule or actual payments result in payments that are too small to pay the interest as it accrues, the guarantee agency shall review the borrower's financial situation at least every six months. If feasible, the agency shall adjust the distribution of each payment between principal and interest so that the principal will be paid within a reasonable time.

(5) The guarantee agency shall pay the Secretary an equitable share of any payment made by or on behalf of a defaulted borrower after the Secretary has reimbursed the agency.

(6) Unless the Secretary approves otherwise, the guarantee agency shall submit the Secretary's equitable share of borrower payments to the Secretary within 60 days of its receipt of the payments.

(7) Except as provided in paragraph (h), there is no other subrogation of the United States to the rights of the guarantee agency on any loan that is subject to this agreement.

(8) Nothing in a reinsurance agreement shall be construed to keep a lender from granting forbearance to a borrower under published criteria of the guarantee agency.

(g) The "Secretary's equitable share" of borrower payments is defined in Section 428(c)(6) of the Act, and is calculated for a complete fiscal year.

(1) The term "overhead" used in that definition includes space and utilities costs.

(2) By December 31 of the succeeding fiscal year, the guarantee agency must submit to the Secretary, in a manner prescribed by the Secretary, information concerning its total borrower payments received and its total administrative costs of collection of loans and preclaims assistance for default prevention incurred during the fiscal year. If this submission shows that the guarantee agency has not paid all the "Secretary's equitable share" of borrower payments to the Secretary for the fiscal year, the guarantee agency must at that time pay the additional amount due to the Secretary.

(h) To protect the Federal fiscal interest, the Secretary may require the guarantee agency to assign to the Secretary any loan which it holds on which the Secretary has paid a reinsurance claim.

(20 U.S.C. 1078, 1078-2, 1082)

§ 683.35 Supplemental Federal reinsurance.

(a) The Secretary may enter into a supplemental reinsurance agreement annually with a guarantee agency that has a reinsurance agreement and that meets the conditions of this section.

(b) *Amount of supplemental reinsurance payments.* (1) The Secretary reimburses a guarantee agency having supplemental reinsurance for 100 percent of its losses, with the following exceptions:

(i) *When reinsurance claims paid by the Secretary to a guarantee agency for any fiscal year reach 5 percent of the "amount of loans in repayment" at the end of the preceding fiscal year.* In this event, the Secretary's reinsurance liability on a claim subsequently paid for that fiscal year will be 90 percent of the amount of the unpaid principal balance plus accrued interest.

(ii) *When reinsurance claims paid by the Secretary to a guarantee agency for any fiscal year reach 9 percent of the "amount of loans in repayment" at the end of the preceding fiscal year.* In this event, the Secretary's reinsurance liability on a claim subsequently paid for that fiscal year will be 80 percent of the amount of the unpaid principal balance plus accrued interest.

(2) *Exception for a new guarantee agency.* For a guarantee agency that entered into a basic agreement under Section 428(b) of the Act after September 30, 1976, or was not actively carrying on a program covered by such agreement on October 1, 1976, the Secretary pays 100 percent of its losses for five consecutive fiscal years beginning with the first year of its operation. The Secretary monitors programs of this type and, if an agency does not prudently administer its program, the Secretary may determine that it does not continue to qualify for this exception.

(c) *Consolidation of the GSLP and PLUS programs for supplemental reinsurance.* (1) If a guarantee agency

enters into a supplemental reinsurance agreement for both the GSLP and PLUS programs, that agency shall consolidate the two programs for the purpose of:

(i) Calculating the amount of loans in repayment as described in paragraph (d)(2) of this section, and

(ii) Calculating the extent of the Secretary's liability as described in paragraph (b) of this section.

(2) If a guarantee agency that has a reinsurance agreement for PLUS loans and GSLP loans under § 683.34 enters into a supplemental reinsurance agreement for one but not both of the GSLP and PLUS programs, the guarantee agency shall not consolidate the two programs, but must continue to maintain records on reinsurance separately for each program to enable the Secretary to determine:

(i) The rate of reimbursement to be paid on reinsurance claims under each program, and

(ii) The Secretary's equitable share of collections received after payment of a reinsurance claim under each program.

(d) *Definitions.* (1) "Losses" is defined in § 683.34(a)(1).

(2) For purposes of this section, the "amount of loans in repayment" means the original principal amount of all loans subject to a supplemental reinsurance agreement insured by the agency minus—

(i) The original principal amount of loans on which—

(A) Under the GSLP, the borrower has not yet reached the repayment period;

(B) Payment in full by the borrower has been made; or

(C) The borrower was in deferment status at the time repayment of principal was scheduled to begin, and remains in deferment status; and

(ii) The amount paid by the agency for insurance claims on loans.

(e) *Program requirements.* To enter into a supplemental reinsurance agreement, a guarantee agency program must meet the following conditions:

(1) *Annual amounts.* The maximum annual amount, attributable to one or more PLUS loans, that the agency shall insure for or on behalf of an eligible student who is carrying at least a half-time workload for an academic year or its equivalent must be—

(i) $2,500, including any amounts borrowed under the GSLP, in the case of an independent undergraduate student;

(ii) $3,000, in the case of a graduate or professional student; and

(iii) At least $2,500 but not more than $3,000, in the case of a parent borrower on behalf of each eligible student.

(2) *Aggregate loan limits.* The agency shall insure a maximum aggregate unpaid principal amount of:

(i) $12,500, including any amounts borrowed under the GSLP, in the case of an independent undergraduate student;

(ii) $15,000, in the case of a graduate or professional student; and

(iii) At least $12,500 but not more than $15,000, in the case of a parent borrower on behalf of each eligible student.

(3) *Extent of insurance.* The agency shall insure 100 percent of the unpaid principal of loans made by lenders under its program.

(4) *School eligibility.* Except in the case of correspondence schools, the agency's eligibility criteria for schools may not be more stringent than those of the Federal PLUS program. However, the agency may exclude a school if—

(i) The school's eligibility is limited, suspended, or terminated by the Secretary under 34 CFR Part 668, or by the agency under comparable standards and procedures; or

(ii) There is a State constitutional prohibition affecting a school's eligibility.

(5) *Out-of-State schools.* The agency shall insure loans made to borrowers who are legal residents of the State where the agency operates, but who attend out-of-State schools or, in the case of parent borrowers, are legal residents of the State where the agency operates, but are borrowing on behalf of students attending out-of-State schools. In insuring these loans, the agency shall not impose any restrictions not applicable to borrowers who are legal residents of the State who attend in-State schools or to parent borrowers who are legal residents of the State and are borrowing for students attending in-State schools.

(6) *School lender provisions.* (i) The agency shall provide that a school may be a lender under reasonable criteria unless—

(A) The school's lending eligibility has been limited, suspended or terminated by the Secretary under § 683.90 or Subpart F or by the agency under comparable criteria and procedures; or

(B) There is a State constitutional prohibition affecting the school's lending eligibility.

(ii) The agency may not insure loans made by school lenders that are not located in the geographic area that the agency serves.

(7) *Reports.* The agency shall agree to report to the Secretary by July 1 of each year regarding—

(i) Its school lender eligibility criteria;

(ii) Its procedures for the limitation, suspension, and termination of school lenders;

(iii) A list of all schools that applied for lender eligibility in the preceding 12 months, and a summary of the actions taken on the applications; and

(iv) A list of all eligible school lenders under the agency's program.

(f) *Terms and conditions.* The supplemental reinsurance agreement will contain, at a minimum, the following terms and conditions, in addition to other provisions of the basic agreement or the reinsurance agreement that the Secretary includes:

(1) *Adherence to qualifying standards.* The agency shall assure that the program requirements of paragraph (e) of this section are continuously met.

(2) Reports and records. The agency shall make reports and keep records that the Secretary reasonably requires. It shall give the Secretary access to those records to verify their correctness.

(3) *Application of payments.* If a borrower makes payments on a loan after the Secretary has paid a reinsurance claim on that loan, the agency shall return to the Secretary an equitable share of the payments. The "Secretary's equitable share" is defined in Section 428(c)(6) of the Act and is calculated for a complete fiscal year.

(i) The term "overhead" used in that definition includes space and utilities costs.

(ii) By December 31 of the succeeding fiscal year, the guarantee agency must submit to the Secretary, in a manner prescribed by the Secretary, information concerning its total borrower payments received and its total administrative costs of collection of loans and preclaim assistance for default prevention incurred during the fiscal year. If this submission shows that the guarantee agency has not paid all of the "Secretary's equitable share" of borrower payments to the Secretary for the fiscal year, the guarantee agency must at that time pay the additional amount due to the Secretary.

(4) An agreement is renewed only if the agency's program complies with all the terms of the agreement and all pertinent provisions of these regulations.

(5) Before the Secretary pays a supplemental reinsurance claim, the guarantee agency must give the Secretary a statement of its "amount of loans in repayment" at the end of the preceding fiscal year. The method for determining this amount is given in paragraph (d)(2) of this section.

(20 U.S.C. 1078, 1078-1, 1078-2)

§ 683.36 Administrative cost allowances for guarantee agencies.

(a) *General.* To the extent that funds are appropriated by Congress in any fiscal year for this purpose, the Secretary may make payments to a

guarantee agency having a basic PLUS program agreement for the primary and secondary administrative cost allowances.

(1) *Total payments.* Payments of allowances to a guarantee agency for any fiscal year made under paragraphs (b) and (c) of this section do not exceed, for each allowance, one-half of 1 percent of the total principal amount of loans for which the guarantee agency issued insurance during that fiscal year.

(i) If the amount appropriated for any fiscal year is insufficient to pay all guarantee agencies the full amounts for which they would otherwise be eligible, payments to all agencies are proportionately reduced.

(ii) In the event of such an insufficiency, if additional funds become available for making payments for that fiscal year, additional payments are distributed on the same basis as they were reduced.

(2) *Application.* The guarantee agency shall submit an application for each allowance to the Secretary by January 1 of the fiscal year for which it is requesting the allowance. The application must contain information and assurances that the Secretary reasonably requires, including the following—

(i) Information showing the agency's ability to collect loans and provide preclaim assistance to its lenders, including descriptions of staff size and activities in these areas;

(ii) An estimate of the costs that will be eligible for payments under this section (categorized by the types of costs listed in paragraph (a)(3)(i) of this section);

(iii) Assurances that sufficient administrative and fiscal procedures, including an annual independent audit or, if a State guarantee agency is subject to State audit procedures not under its control, a biennial independent audit, will be used to ensure that the administrative allowances are used in accordance with the provisions of this section, and that the audit report will be made available to the Secretary on request;

(iv) Assurances that the guarantee agency will furnish any further information, including estimates, that the Secretary may reasonably require to carry out the provisions of this section;

(v) For the primary allowance application only, an estimate of the total amount on new loan volume expected to be insured during the fiscal year; and

(vi) For the secondary allowance only, assurance that the agency's program—

(A) Meets all the requirements for a supplemental reinsurance agreement;

(B) Insures loans for independent undergraduate students and graduate or professional students who are not legal residents of the State, but who are attending participating schools in the State other than correspondence schools, without imposing any restrictions not imposed on legal residents of the State who attend schools in the State other than correspondence schools; and

(C) Insures loans to parent borrowers who are not legal residents of the State, but who are borrowing for students attending participating schools in the State (other than correspondence schools), without imposing any restrictions not imposed on borrowers who are legal residents of the State and who borrow for students attending schools in the State other than correspondence schools.

(3) *Definitions.* (i) The terms "administrative costs of promotion of commercial lender participation," "administrative costs of collection of loans," and "administrative costs of preclaims assistance for default prevention," as used in paragraphs (b) and (c) of this section, are defined in Section 428(f)(3) of the Act. The term "administrative costs of monitoring the enrollment status of students and the repayment status of borrowers," as used in paragraphs (b) and (c) of this section, has the same meaning as "administrative costs of monitoring the enrollment and repayment status of students," as defined in Section 428(f)(3) of the Act, except that the reference to repayment status in cases where the borrower is a parent, shall be understood to refer to that of the parent borrowers.

(ii) The term "overhead costs" used in those definitions includes space and utilities costs.

(b) *Primary allowance*—(1) *Basic qualification.* The agency must have a basic PLUS program agreement.

(2) *Use of funds.* The primary allowance must be used by the agency only to meet administrative costs of promotion of commercial lender participation, administrative costs of collection of loans, administrative costs of preclaim assistance for default prevention, administrative costs of monitoring the enrollment status of students and the repayment status of borrowers, and other administrative costs related to the PLUS program of the guarantee agency. Also, this allowance must be used to meet only administrative costs not taken into account by the agency under the formula for determining the "Secretary's equitable share" of borrower payments made after the Secretary has paid reinsurance claims to the agency.

(c) *Secondary allowance.* (1) Payment of the secondary allowance is made in addition to payment of the primary allowance.

(2) *Basic qualification.* The agency must have a reinsurance agreement for PLUS loans.

(3) *Use of funds.* The secondary allowance must be used by the agency only to meet administrative costs of promotion of commercial lender participation, administrative costs of collection of loans, administrative costs of preclaim assistance for default prevention, administrative costs of monitoring the enrollment status of students and the repayment status of borrowers, and other administrative costs related to the program of the guarantee agency. Also, this allowance must be used to meet only administrative costs not taken into account by the agency under the formula for determining the "Secretary's equitable share" of borrower payments made after the Secretary has paid reinsurance claims to the agency.

(4) The Secretary's payment of the secondary allowance establishes an agreement between the Secretary and the guarantee agency with respect to the assurances contained in the application.

(20 U.S.C. 1078, 1078-1, 1078-2, 1082)

§ 683.37 Records, reports, and inspection requirements for guarantee agency programs.

(a) *Records.* (1) A guarantee agency shall keep the records specifically required by this section and the records necessary to make reports required by this subpart. The guarantee agency shall retain records for each loan for at least five years after the loan is paid in full or has been determined to be uncollectible. For the purposes of this section, the term "paid in full" includes loans paid by the Secretary on account of the borrower's death, permanent and total disability, or bankruptcy. These records must be as complete and accurate as is necessary to document fully the agency's reports.

(2) The guarantee agency shall require participating lenders to keep records on guaranteed loans as prescribed by the Secretary. These shall include complete and accurate records of each loan account, showing each transaction and affording ready identification of the borrower's status. A lender shall retain records of a loan for at least five years from the date the loan has been paid in full by the borrower or the lender has been reimbursed for a loss on the loan by the guarantee agency. The Secretary may, in particular cases, require the

retention of records beyond this 5-year minimum period.

(3) Guarantee agencies and lenders may store records in microfilm or computer format. However, the lender or guarantee agency holding a promissory note shall retain the actual note until the loan is paid in full or determined by the guarantee agency to be uncollectible. When repayment is complete, the lender or guarantee agency shall return the actual note to the borrower and retain a copy for the prescribed period. If a loan is written off as uncollectible, the original note need not be retained, but a copy must be retained for the prescribed period.

(b) *Reports.* (1) The agency shall submit reports to the Secretary upon request concerning the status of its reserve funds, and the operations of its loan insurance program.

(2) The agency shall submit to the Secretary, at least annually, a report of the total insured loan volume and default volume and rate on all loans insured after December 31, 1980, for each of the following categories of lenders:

(A) Schools.

(B) State or private nonprofit direct lenders.

(C) Commercial financial institutions (banks, savings and loan associations, or credit unions).

(D) All other types of institutions or agencies. Loan volume and default data shall be reported according to the category of original lender, not subsequent holder. If a guarantee agency operates in more than one State, a separate report must be submitted for each State of operation.

(3) The agency shall submit to the Secretary its application forms, promissory notes, regulations, and statements of procedures and standards—including standards for due diligence and timely claims filing—as well as other materials that substantially affect the operation of the agency's program, whenever requested to do so by the Secretary and whenever changes or new materials are proposed. The Secretary reviews these materials for administrative and fiscal sufficiency and for conformance to statutory and regulatory provisions.

(4) Lenders shall submit to the agency the information necessary for the agency to complete its reports to the Secretary.

(5) The agency shall submit, or require its lenders to submit, upon the Secretary's request, information the Secretary needs to determine the amount of special allowance to be paid on the agency's insured loans.

(c) *Inspections.* (1) A guarantee agency shall give the Secretary, or other agencies of the government designated by the Secretary, access to its records in order to assure the accuracy of the reports described in paragraph (b) of this section.

(2) A guarantee agency shall provide in its agreement with a lender or in its statements of procedures that the lender shall give the Secretary, or other agencies of the government designated by the Secretary, and the agency access to the lender's records in order to assure the accuracy of the reports required under paragraphs (b) (4) and (5) of this section.

(20 U.S.C. 1072, 1078, 1078-2, 1082)

Subpart D—Federal PLUS Program

§ 683.50 Circumstances under which loans may be insured.

The Secretary may insure PLUS loans made by lenders located in a State in the following curcumstances:

(a) Where no guarantee agency is insuring GSLP loans in that State;

(b) Where a guarantee agency is insuring GSLP loans in that State and is not prohibited by State law from authorizing PLUS loan insurance, but is not insuring PLUS loans; or

(c) Where a guarantee agency is insuring GSLP loans in that State, but State law enacted prior to January 1, 1981 prohibits any guarantee agency from insuring PLUS loans in that State, after 120 days following the adjournment of the first regular session of the State legislature which convenes after January 1, 1981.

(20 U.S.C. 1078-2, 1082)

§ 683.51 Extent of Federal insurance.

(a) *General rule.* Except as provided in paragraph (b) of this section, the Secretary's insurance liability on any Federal PLUS loan is 100 percent of the unpaid principal balance and accrued interest.

(b) *Special provisions for State lenders.* For purposes of this calculation, State lenders may consolidate FISLP and PLUS loans.

(1) Except as described in subparagraph (2), of this paragraph, the Secretary's insurance liability is less than 100 percent under the following conditions:

(i) *When the total of default claims for the FISLP and PLUS program paid by the Secretary to a State lender for any fiscal year reaches 5 percent of the amount of the FISLP and PLUS loans in repayment at the end of the preceding fiscal year.* In this event, the Secretary's insurance liability on a claim subsequently paid for that fiscal year will be 90 percent of the amount of the unpaid principal balance plus accrued interest.

(ii) *When the total of default claims for the FISLP and PLUS program paid by the Secretary to a State lender for any fiscal year reaches 9 percent of the amount of the FISLP and PLUS loans in repayment at the end of the preceding fiscal year.* In this event, the Secretary's insurance liability on a claim subsequently paid for that fiscal year will be 80 percent of the amount of the unpaid principal balance plus accrued interest.

(2) The potential reduction in insurance liability does not apply to a State lender during the first Federal fiscal year of its operation as a lender in the FISLP or PLUS program and during each of the four succeeding fiscal years. If the lender has not previously participated in the FISLP, this 5-year period begins to run with the first Federal fiscal year of the State lender's operation as a lender under the Federal PLUS program.

(3) For purposes of this section, the "amount of loans in repayment" means the original principal amount of both FISLP and PLUS loans insured by the Secretary minus—

(i) The original principal amount of loans on which—

(A) Under the FISLP, the borrower has not yet reached the repayment period;

(B) Payment in full by the borrower has been made; or

(C) The borrower was in deferment status at the time repayment of principal was scheduled to begin, and remains in deferment status; and

(ii) The amount paid by the Secretary for insurance claims on loans.

(4) For purposes of this paragraph, payments by the Secretary on a loan that the original lender assigned to a subsequent holder are considered payments made to the original lender.

(20 U.S.C. 1075, 1078-2, 1082)

§ 683.52 Application to become a lender under the Federal PLUS program.

(a) *General.* To participate in the Federal PLUS program, a lender that does not hold a Federal contract of insurance must submit an application to the Secretary for an insurance contract. The Secretary responds to the lender's request to participate in the Federal PLUS program within 30 days of receipt of an application.

(b) *Criteria for evaluating an application.* In determining whether to enter into an insurance contract with an applicant and what the terms of that contract should be, the Secretary may consider the following criteria:

(1) Whether the applicant is capable of complying with these regulations as they apply to lenders.

(2) Whether the applicant is capable of implementing adequate procedures for making and collecting loans.

(3) If the applicant has had prior experience with a similar Federal, State or private nonprofit student or parent loan program, the amount and rate of loans that currently are delinquent or in default under that program.

(4) The financial resources of the applicant.

(5) In the case of a school that is seeking approval as a lender, its accreditation status, with the preferred condition being accredited.

(c) The Secretary requires an applicant to submit sufficient materials with its application so that the Secretary may evaluate it fairly in accordance with these criteria.

(d) *Denial of participation.* (1) If the Secretary decides not to approve the application for an insurance contract, the reason for the decision is included in the Secretary's response.

(2) The Secretary provides an opportunity for the lender to meet with a designated Department of Education official if the lender wishes to appeal the Secretary's decision.

(3) However, the Secretary need not explain the reasons for the denial, or grant the lender an opportunity to appeal, if the lender submits its application within 6 months of a previous denial.

(20 U.S.C. 1078-2, 1082.)

§ 683.53 The lender insurance contract.

(a) *Approval of insurance contract.* (1) If the Secretary approves a lender's application to be a Federal PLUS lender, the Secretary and the lender sign an insurance contract. No loan is insured unless covered by an insurance contract.

(2) In general, under an insurance contract the lender agrees to comply with all laws, regulations and other requirements applicable to its participation as a lender in the Federal PLUS program, and the Secretary agrees to insure each eligible Federal PLUS loan held by the lender against the borrower's default, death, total and permanent disability, or bankruptcy.

(3) The Secretary's insurance liability is the amount of unpaid principal and interest, except for certain loans made by a State lender as provided in § 683.51(b).

(4) The contract may contain a limit on the duration of the contract and the number or amount of Federal PLUS loans a lender may make or hold.

(b)(1) Except as otherwise approved by the Secretary, an insurance contract with a school lender shall limit the PLUS loans made by that school lender which will be covered by Federal loan insurance to those made to students or to parents borrowing on behalf of students—

(i) Who are in attendance at that school; or

(ii) Who are in attendance at other schools under the same ownership; or

(iii) Who are employees, or whose parents are employees, of that school lender or other schools under the same ownership, under circumstances the Secretary considers appropriate for insurance.

(2) A limit imposed under paragraph (a)(4) of this section on a school lender which makes loans to students or parents of students in attendance at other schools under the same ownership, or to employees or parents or dependents of employees of those other schools, may be imposed on a school-by-school basis.

(20 U.S.C. 1078-2, 1079, 1082)

§ 683.54 Issuance of Federal loan insurance.

(a) *Application for insurance.* A lender having an insurance contract shall submit an application to the Secretary for Federal loan insurance on each intended PLUS loan that the lender determines to be eligible for insurance. The application shall be on a form prescribed by the Secretary. The Secretary notifies the lender whether the loan is or is not insurable and the amount of the insurance. No disbursement on a loan made prior to the Secretary's approval of that loan is insurable.

(b) *Conditions of insurance coverage.* The Secretary issues insurance on a PLUS loan in reliance on the implied representations of the lender that all requirements for the initial insurability of the loan have been met. As described in § 683.66, the continuance of the insurance is conditioned upon compliance by all holders of the loan with these regulations. The delegation of functions to a servicing agency or party does not relieve the lender of its responsibilities in the making and collection of a Federal PLUS loan.

(20 U.S.C. 1078-2, 1079, 1082)

§ 683.55 Limitations on maximum loan amounts.

(a) *Annual amounts.* The Secretary does not insure a loan that would exceed the student's estimated cost of attendance for the academic period for which the loan is intended less estimated financial assistance awarded for that period. The maximum annual loan amount, attributable to one or more PLUS loans, that the Secretary insures for or on behalf of an eligible student who is carrying at least a half-time workload for any academic year or its equivalent, does not exceed:

(1) $2,500, including any amounts borrowed under the GSLP, in the case of an independent undergraduate student;

(2) $3,000, in the case of a graduate or professional student; and

(3) $3,000, in the case of a parent borrower on behalf of each eligible student.

(b) *Aggregate loan limits.* The Secretary insures a maximum aggregate unpaid principal amount of:

(1) $12,500, including any amounts borrowed under the GSLP, in the case of an independent undergraduate student;

(2) $15,000, in the case of a graduate or professional student; and

(3) $15,000, in the case of a parent borrower on behalf of each eligible student.

(c) *Limitation on a loan to or on behalf of a student enrolled in a correspondence course.* The Secretary does not insure a loan to or on behalf of a student enrolled in a correspondence course in an amount which exceeds tuition and fees and, if required, books and supplies, and travel and room and board costs incurred specifically in fulfilling a required period of residential training.

(20 U.S.C. 1075, 1078-2, 1079, 1082, 1089)

§ 683.56 Insurance premiums.

(a) *General.* The Secretary charges the lender an insurance premium for each loan that is insured.

(b) *Rate.* The rate of the insurance premium is one-fourth of one percent per year of the loan principal, excluding interest or other charges that may have been added to the principal.

(c) *How the insurance premium is calculated.* (1) The insurance premium is calculated by:

(i) Using the actual repayment period as a base;

(ii) Amortizing the loan in monthly installments over the repayment period;

(iii) Determining one-fourth of one percent of each monthly declining balance; and

(iv) Totaling the monthly amounts derived in subdivision (iii) of this subparagraph.

(d) *Collection from lenders.* (1) The Secretary requires the lender to pay the insurance premium when the disbursement is reported. At the Secretary's discretion, the Secretary may collect the insurance premium by

offsetting it against amounts payable by the Secretary to the lender.

(2) Insurance coverage on a Federal PLUS loan ceases to be effective when the lender fails to pay the insurance premium within 60 days of the date payment is due. The Secretary may, however, excuse late payment of an insurance premium, and reinstate the insurance on a loan, if the Secretary is satisfied that the loan is not in default and the borrower is not delinquent in making installment payments.

(e) *Collection from borrowers.* The lender may pass along the cost of the insurance premium to the borrower in the form of a one-time charge. The lender may bill the borrower for the insurance premium or may deduct the amount from the loan proceeds. The lender must clearly identify to the borrower the amount of insurance premium and the method of calculation.

(f) *Refund provisions.* The premium is not refundable by the Secretary, and need not be refunded by the lender to the borrower, even if the borrower prepays, defaults, dies, becomes totally and permanently disabled, or files a petition for bankruptcy.

(20 U.S.C. 1077, 1078-2, 1079, 1082)

§ 683.57 Repayment of loans.

(a) *Commencement of repayment.* (1) The repayment period begins on the day the loan is disbursed, and interest begins to accrue on that date. The first payment is due within 60 days after the date of disbursement.

(2) If a condition that justifies a deferment of repayment exists when the loan is disbursed, both the deferment and the accrual of interest commence on that date. Repayment of principal begins following the expiration of the deferment period or the post-deferment grace period (as defined in § 683.10).

(b) *Length of repayment period.* In general, a lender shall allow a borrower at least 5 years, but not more than 10 years, to repay a loan, calculated from the beginning of the repayment period. There are exceptions, however, to this rule:

(1) If the borrower receives an authorized deferment or has been granted forbearance, as described in § 683.61(c), the periods of deferment or forbearance are generally excluded from determinations of the 5- and 10-year periods. However, the 5- and 10-year periods include each post-deferment grace period (as defined in § 683.10) following each authorized deferment.

(2) If the minimum annual repayment required in paragraph (d) of this section would result in complete repayment of the loan in less than 5 years, the borrower is not entitled to the full 5-year period.

(c) *Prepayment.* The borrower may prepay the whole or any part of a loan at any time without penalty.

(d) *Minimum annual payment.* (1) During each year of the repayment period, a borrower's total payments to all holders of his or her PLUS loans and GSLP loans must total at least $600 or the unpaid balance of all loans, including interest, whichever amount is less. There are, however, two exceptions to this rule:

(i) If the borrower and the lender agree, the amount paid may be less.

(ii) If the borrower and his or her spouse have separate PLUS loans, or one or more PLUS loans and one or more GSLP loans, their combined annual payment must meet this requirement.

(2) The provisions of subparagraphs (1)(i) and (ii) may not result in an extension of the 10-year repayment period maximum, unless forbearance has been approved under § 683.61(c).

(e) *Student failure to enroll on at least a half-time basis.* If a lender disburses a PLUS loan and later learns that the borrower or student on whose behalf the parent borrowed has not been or will not be enrolled on at least a half-time basis at a participating school during the period for which the loan was intended, the lender shall notify the borrower that full payment of the loan is immediately due. If the lender determines such action is necessary to prevent default, the lender may allow the borrower to repay the loan in installments. However, the borrower is not entitled to periods of deferment under § 683.58.

(f) *Repayment schedule.* The repayment schedule may provide for substantially equal installment payments or for installment payments that increase in amount over the repayment period. If a graduated repayment schedule is established, it may not provide for any single installment that is more than 3 times greater than any other installment.

(g) *Supplemental repayment agreement.* (1) For a loan made by a school lender, the lender and the borrower may enter into an agreement supplementing the regular repayment schedule under paragraph (f) of this section. Under a supplemental repayment agreement, the lender agrees that the borrower is deemed to meet the terms of the regular repayment schedule as long as the borrower makes payments in accordance with a separate schedule. However, the regular schedule must provide for equal installments.

(2) The purpose of a supplemental repayment agreement is to extend the 10-year repayment period maximum and to permit a lender to offer a borrower a repayment schedule based on other than equal or graduated payments. For example, a supplemental repayment agreement may base the amount of the borrower's payment on the borrower's income.

(3) The agreement and separate schedule must contain terms that the Secretary believes do not unduly burden the borrower and do not subject the Secretary to undue liability. A lender and borrower may not enter into a supplemental repayment agreement unless the lender has obtained the Secretary's prior approval of its terms.

(4) The borrower may not insist upon the establishment of a supplemental repayment agreement.

(5) A lender may assign a loan subject to a supplemental repayment agreement only if the buyer agrees to accept the loan subject to the terms of the supplemental agreement.

(6) For purposes of the special allowance, and the determination of the amount of loss on an insurance claim, the unpaid principal balance of the loan is based on the regular repayment agreement.

(20 U.S.C. 1077, 1078, 1078-2, 1079, 1082.)

§ 683.58 Deferment.

(a) *Borrower eligibility.* (1) A borrower is entitled to have periodic installment payments of principal deferred during authorized periods. For a loan made prior to October 1, 1981, the borrower is also entitled to have periodic installment payments of principal deferred during the post-deferment grace period, which commences after the completion of each deferment period or combination of such periods. Except as provided in paragraphs (e)(4) and (f)(4) of this section, a period of authorized deferment begins when the condition entitling a borrower to deferment first exists. Interest accrues and is payable by the borrower during the deferment and (if applicable) the post-deferment grace period. The borrower shall provide to the lender all documentation required to establish eligibility for a specific type of deferment.

(2) A deferment cannot be denied by a lender when the borrower meets the eligibility criteria, even though the borrower may be delinquent, but not in default, in making required installment payments. The 120- or 180-day period required to establish a default does not run during the deferment and post-deferment grace periods. When the deferment or, if applicable, the post-deferment grace period expires, a borrower resumes any delinquent status

that existed when the deferment period began.

(3) A borrower whose loan is in default is not eligible for a deferment unless the borrower has made satisfactory arrangements with the lender to bring the account current.

(4) If both parents of a student have obtained a loan as co-makers, both parents must be eligible for a deferment in order for payments of principal to be deferred.

(b) *Authorized deferments.* Deferment is authorized during periods when a borrower is engaged in at least one of the following activities:

(1)(i) Full-time study at a participating school, unless the borrower is not a national of the United States and is pursuing a course of study at a school not located in a State; or

(ii) Full-time study at a school which meets the definition of an institution of higher education or a vocational school and is operated by an agency of the Federal Government (e.g., the service academies), unless the borrower is not a national of the United States and is pursuing a course of study at a school not located in a State.

(2) Study under an eligible graduate fellowship program, as described in paragraph (c) of this section.

(3) Up to 3 years of active duty service in the United States Armed Forces or of service as an officer in the Commissioned Corps of the United States Public Health Service.

(4) Up to 3 years of volunteer service under the Peace Corps Act.

(5) Up to 3 years of service as a full-time volunteer under Title I of the Domestic Volunteer Service Act of 1973 (ACTION programs).

(6) Up to 3 years of full-time volunteer service which the Secretary has determined is comparable to service referred to in paragraphs (b)(4) and (b)(5) of this section for a tax-exempt organization, as described in paragraph (d) of this section.

(7) Conscientiously seeking but unable to find full-time employment in the United States over a single period of up to 12 months, as described in paragraph (e) of this section.

(8) Pursuing a course of study under a rehabilitation training program for disabled individuals, as described in paragraph (f) of this section.

(9) Up to 2 years of service as an intern, as described in paragraph (g) of this section.

(10) Up to 3 years during which the borrower is temporarily totally disabled, as described in paragraph (h) of this section, or during which the borrower is unable to secure employment because he or she is caring for a spouse who is temporarily totally disabled, as described in paragraph (i) of this section.

(c) *Graduate fellowship deferment.* To qualify for a deferment for study under a graduate fellowship program, a borrower shall provide the lender with a statement from an official of the borrower's fellowship program certifying that—

(1) The fellowship program—

(i) Provides sufficient financial support to graduate fellows to allow for full-time study for at least 6 months;

(ii) Requires, prior to the award of that financial support, a written statement from each applicant which explains the applicant's objectives; and

(iii) Requires a graduate fellow to submit periodic reports, projects, or other evidence of the graduate fellow's progress; and

(2) The borrower—

(i) Holds at least a baccalaureate degree conferred by an institution of higher education;

(ii) Is engaged in full-time study, that may be independent of an educational or cultural institution, in an academic or professional subject area for which the borrower has shown an interest and ability; and

(iii) Has been recommended by an institution of higher education for acceptance into the graduate fellowship program.

(d) *Full-time volunteer service for a tax-exempt organization deferment.* To qualify for a deferment for full-time volunteer service for a tax-exempt organization comparable to volunteer service in the Peace Corps or full-time volunteer service in a program administered by the ACTION agency, a borrower shall provide the lender with a statement from an official of the borrower's volunteer program certifying that—

(1) The borrower serves in an organization which is exempt from taxation under Section 501(c)(3) of the Internal Revenue Code of 1954;

(2) The borrower provides service to low-income persons and their communities to assist them in eliminating poverty and poverty-related human, social, and environmental conditions;

(3) The borrower's compensation does not exceed the compensation received by a full-time volunteer in the Peace Corps or in a program administered by the ACTION agency. Compensation includes a subsistence allowance, necessary travel expenses and stipends;

(4) The borrower, as part of his or her duties, does not give religious instruction, conduct worship services, engage in religious proselytizing, or engage in fundraising to support religious activities; and

(5) The borrower has agreed to serve on a full-time basis for a term of at least one year.

(e)(1) *Basic eligibility for an unemployment deferment.* (i) For purposes of this section, full-time employment involves at least 30 hours of work per week and is expected to last at least 3 months.

(ii) A borrower is entitled to the deferment whether or not he or she has been previously employed. If previously employed, the borrower is entitled to a deferment regardless of the circumstances under which the employment ended.

(iii) An unemployment deferment is not justified if the borrower has sought employment only in kinds of positions or at salary and responsibility levels for which he or she feels qualified by virtue of education or previous experience.

(2) *Submission of request.* To receive an unemployment deferment, a borrower shall submit a request for the deferment in writing to the holder of the loan. To continue the deferment for more than 6 months, the borrower shall submit a second request by the end of the first 6-month period. Each request must be signed and dated and contain the following:

(i) A statement from the borrower describing his or her conscientious search for full-time employment.

(ii) The borrower's latest permanent home address and, if applicable, the borrower's latest temporary address.

(iii) Certification that the borrower has registered with a public or private employment agency, if one is accessible, specifying its name and address.

(iv) The borrower's agreement to notify the lender promptly when he or she becomes employed full-time.

(3) *Lender's approval or disapproval of request.* (i) The lender must review the borrower's request and notify the borrower of its decision within one month after receipt of the request.

(ii) The lender may rely upon the written statements provided by the borrower, unless the lender has information to the contrary.

(iii) If the lender is satisfied that the borrower has conscientiously searched for full-time employment and otherwise meets the requirements for an unemployment deferment, the lender shall approve the request.

(iv) If the borrower's request does not justify an unemployment deferment, the lender may grant the borrower forbearance if authorized under § 683.61.

(4) *When the unemployment deferment begins.* An unemployment deferment begins—

(i) On the date that the lender approves the request; or

(ii) On a date not in excess of 60 days prior to the lender's approval if the unemployment existed at the earlier date.

(5) *When the unemployment deferment ends.* An unemployment deferment ends on the earliest of—

(i) The date the lender learns that the borrower has become employed full-time;

(ii) One month after the date when a certification of unemployment deferment eligibility is due from the borrower but has not been received; or

(iii) Twelve months after the commencement of the deferment period.

(f) *Rehabilitation training program deferment.* (1) To qualify for this deferment—

(i) The borrower must either be receiving or be scheduled to receive services under a program designed to rehabilitate disabled individuals; and

(ii) The borrower's rehabilitation program must meet the Secretary's criteria for approval.

(2) *Criteria for the Secretary's approval of rehabilitation training programs.* The Secretary approves a rehabilitation training program if the organization providing rehabilitation services to the borrower—

(i) Is licensed, approved, certified or otherwise recognized as providing rehabilitation training to disabled individuals by any of the following agencies—

(A) A State vocational rehabilitation agency;

(B) A State agency for drug abuse treatment;

(C) A State agency for mental health services;

(D) A State agency for alcohol abuse treatment; or

(E) The Veterans Administration; and

(ii) Provides or has agreed to provide that borrower with services for his or her rehabilitation under a rehabilitation plan that is—

(A) Written;

(B) Individualized to meet the borrower's needs;

(C) Specific as to the date on which the services to the borrower are expected to end; and

(D) Structured in a way that requires a substantial commitment by the borrower to his or her rehabilitation. The Secretary considers a substantial commitment by the borrower to be a commitment of time or effort that would normally prevent an individual from engaging in full-time employment either because of the number of hours that he or she must devote to rehabilitation or because of the nature of his or her rehabilitation. For the purpose of this paragraph, full-time employment involves at least 30 hours of work per week.

(3) *Qualification for a rehabilitation training program deferment.* (i) To obtain a rehabilitation training deferment, a borrower shall provide the lender with evidence that he or she qualifies for the deferment, in a form approved by the Secretary. The evidence must include—

(A) A statement from the provider of the rehabilitation services certifying that the borrower either is receiving or is scheduled to receive rehabilitation training services from the provider; and

(B) A statement from an agency described in paragraph (f)(2)(i) of this section certifying that the provider from which the borrower is receiving services or is scheduled to receive services meets the requirements of paragraph (f)(2) of this section with respect to that borrower.

(ii) On receipt of the properly completed request, the lender shall grant the borrower a deferment.

(4) *When the rehabilitation training program deferment begins.* The rehabilitation training deferment begins—

(i) In the case of a borrower who is actually receiving rehabilitation services under an approved program, on the date specified in the borrower's rehabilitation plan as the beginning date for his or her receipt of services through that program;

(ii) In the case of a borrower who is requesting deferment based on being scheduled to receive rehabilitation services under an approved program, on the date that the borrower provides the lender with a properly completed request.

(5) *When the rehabilitation training program deferment ends.* Unless the borrower re-establishes his or her eligibility to continue a deferment by repeating the request procedure described in paragraph (f)(3) of this section, the rehabilitation training program deferment ends on the earliest of the following dates:

(i) In the case of a borrower who was granted deferment based on being scheduled to receive rehabilitation services under an approved program, the date the borrower informs the lender that he or she no longer plans to receive rehabilitation services; or

(ii) In the case of a borrower who was granted deferment based on being scheduled to receive rehabilitation services under an approved program, the date three months from the date the borrower submitted a request for a rehabilitation deferment; or

(iii) The date the borrower ceases rehabilitation training; or

(iv) The date specified in the borrower's rehabilitation plan as the date on which rehabilitation services to the borrower are anticipated to end; or

(v) The date twelve months from the date the borrower begins receiving services.

(g) *Internship deferment.* To qualify for an internship, deferment, a borrower shall provide the lender with evidence that he or she is serving in an eligible internship, after obtaining a baccalaureate or professional degree, which the Secretary has determined is required to receive professional recognition required to begin professional practice or service. The Secretary has determined that medical residency is an example of such an internship.

(h) *Borrower's temporary total disability deferment.* (1) To qualify for this deferment the borrower shall give the lender an affidavit of a qualified physician stating that the borrower is temporarily totally disabled.

(2) For purposes of this section, a borrower who is "temporarily totally disabled" is one who, by reason of injury or illness, cannot be expected to be able to attend school or to be gainfully employed during an extended period of time needed to recover from such an injury or illness.

(i) *Deferment for a borrower whose spouse is temporarily totally disabled.* To qualify for this deferment the borrower shall give the lender—

(1) An affidavit of a qualified physician stating that the borrower's spouse is temporarily totally disabled. For purposes of this section a spouse who is "temporarily totally disabled" is one who, by reason of injury or illness, cannot be expected to be gainfully employed during an extended period of time needed to recover from such injury or illness and who during that period requires continuous nursing or other similar services; and

(2) A statement from the borrower certifying that he or she is unable to secure employment because he or she is providing this care.

(20 U.S.C. 1077, 1078, 1078-2, 1082, 1085; 42 U.S.C. 5055(e))

§ 683.59 Due diligence in making and disbursing a loan.

(a) *General.* (1) The loan-making process includes the processing of necessary forms, the approval of a borrower for a loan, the determination

of the loan amount, the explanation to a borrower of the borrower's responsibilities under the loan, the completion by the borrower of the promissory note, and the disbursement of the loan proceeds.

(2) Except as may be authorized by the Secretary, a lender may not delegate its loan-making functions except to a school with whom the lender has an origination relationship. If an origination relationship exists, the lender may rely in good faith upon statements of the borrower and, if the borrower is a parent, the student contained in the loan application, but may not rely upon statements made by the school in the application. A non-school lender which does not have an origination relationship with a school may rely in good faith upon statements of the borrower, the student and the school which are contained in the application. A school lender may rely in good faith upon statements made by the borrower and, if the borrower is a parent, the student in the loan application.

(b) *Processing of forms.* Before making a loan, a lender must, subject to paragraph (a)(2) of this section, determine that all required forms have been accurately completed by the borrower, the student, the school, and the lender. A lender must not ask the borrower to sign any form before all information requested of the borrower on that form has been supplied.

(c) *Approval of borrower and determination of loan amount.* (1) A lender may make a loan only to an eligible borrower. To the extent authorized in paragraph (a)(2), the lender may make its determination as to the eligibility of the borrower based on the information provided on the application by the school, the borrower, and, if the borrower is a parent, the student on whose behalf the loan is intended.

(2) In determining the amount of the loan to be made, within the limitations of § 683.55, the lender should review the data on the student's cost of attendance and estimated financial assistance which is provided on the application form. In no case may the loan amount exceed the student's estimated cost of attendance for the academic period for which the loan is intended less estimated financial assistance.

(d) *Borrower interview.* (1) Before making an initial loan to a borrower, a lender should meet personally with the borrower to ensure that the borrower understands his or her rights and responsibilities under the loan.

(2) In particular, the lender should explain that the loan funds may be applied only toward educational expenses of the student listed on the application.

(e) *Establishing repayment terms.* When establishing repayment terms, the lender should take into consideration the financial obligations and the current and potential income of the borrower. The lender should design a repayment schedule that retires the loan obligation as soon as possible, as permitted under § 683.57(b), without leading to default caused by the borrower's inability to make payments.

(f) *Providing loan information.* By no later than when the note is signed, the lender shall provide the borrower with information, including the repayment schedule, concerning his or her rights and responsibilities under the loan, as set forth in § 683.16.

(g) *Promissory note.* (1) The lender shall obtain from the borrower an executed promissory note for each loan as proof of the borrower's indebtedness.

(2) The Secretary periodically makes an approved promissory note form available to Federal PLUS lenders. A lender may not add any clauses to, or modify any of the provisions of, the most current promissory note provided by the Secretary without the Secretary's prior approval.

(3) The lender must give the borrower a copy of each executed note.

(4) The lender shall retain the original promissory note until the loan is paid in full. Within 30 days of the date the loan has been paid in full, the lender shall give the borrower the original promissory note.

(h) *Security, endorsement and co-makers.* (1) A Federal PLUS loan must be made without security.

(2) Except as provided in paragraph (4), a Federal PLUS loan must be made without endorsement or other secondary liability on the note.

(3) A Federal PLUS loan may be made to two eligible parents who agree to be jointly liable for repayment of the loan as co-makers.

(4) A lender may require an endorsement by another person on the borrower's PLUS note only if the borrower is a minor and cannot under applicable State law create a legally binding obligation by his or her own signature. For purposes of this paragraph, "endorsement" means a signature of any party—other than the borrower—who is to assume secondary liability on the loan.

(i) *Loan disbursement.* (1)(i) A lender may not disburse a loan prior to the issuance of the insurance commitment by the Secretary. The lender shall disburse loan funds by means of a check payable only to the borrower. The check must require the personal endorsement of the borrower. Deposit of the check by the borrower in his or her account at a bank or other financial institution constitutes endorsement for purposes of this paragraph.

(ii) The lender shall disburse the loan check directly to the borrower, either in person or by mail. The lender may not disburse the check to the borrower earlier than is reasonably necessary to meet the student's cost of attendance for the period for which the loan is made and in no case, without the Secretary's approval, earlier than 30 days prior to the date on which the student is scheduled to enroll.

(2) Neither a lender nor a school may obtain a borrower's power of attorney or other authorization to endorse a disbursement check on behalf of a borrower. The borrower shall personally endorse the check and may not authorize anyone else to endorse it on his or her behalf.

(3) For purposes of the Federal PLUS program, a check is a draft drawn on a bank and payable on demand.

(4) *Late disbursements.* (i) Under certain circumstances a lender, with the prior approval of the Secretary, may disburse a Federal PLUS loan after the student has ceased to be enrolled on at least a half-time basis or after the expiration date of the insurance commitment.

(ii) The Secretary will approve a lender's request to make a disbursement under these circumstances only if satisfied that the loan proceeds will be used for the student's cost of attendance for the period of enrollment for which the loan was intended.

(20 U.S.C. 1077, 1078-2, 1080, 1082, 1083, 1085)

§ 683.60 Due diligence in collecting a loan.

(a) *General.* (1) A lender must exercise due diligence in the collection of a Federal PLUS loan. In order to exercise due diligence, a lender, except as provided in subparagraph (2) of this paragraph, shall implement the following procedures when a borrower fails to honor his or her payment obligation. If two borrowers are liable for repayment of a loan as co-makers, the lender shall follow these procedures with respect to both borrowers.

(2) A lender shall respond on a timely basis to written inquiries and other communications from a borrower.

(3) Paragraphs (b) through (f) of this section shall not apply—

(i) After it has been determined, or while a lender is seeking to have a determination made, that a borrower has died, become totally and permanently disabled, or has filed a

bankruptcy petition as set forth in § 683.63; or

(ii) After it has been determined that any of the conditions for filing a default claim without previous collection efforts exist, as set forth in §§ 683.64 and 683.66(e).

(b) *Initial delinquency.* When a borrower is delinquent in making a payment, the lender shall remind the borrower within 15 working days of the date the payment was due by means of a letter, notice, telephone call, or personal contact. If payments do not begin or resume, the lender must attempt to contact the borrower at least 3 more times at regular intervals during the rest of the 4-month period that started on the due date of the delinquent payment. These contacts should become progressively more forceful in tone.

(c) *Skip-tracing assistance.* Whenever a lender does not know the borrower's current address, the lender shall attempt to locate the borrower through normal commercial collection techniques, including contacting any other individuals named on the borrower's loan application. If these efforts are unsuccessful, the lender shall attempt to learn the borrower's current address through use of the Department of Education's skip-tracing assistance. The Secretary does not pay insurance on a default claim if the lender did not know the borrower's address but failed to request this skip-tracing assistance. If the lender obtains knowledge of the borrower's address prior to filing a default claim, the lender must attempt to contact the borrower.

(d) *Pre-claim assistance.* When a borrower is 60 days delinquent in making payment, the lender must request pre-claim assistance from the Department of Education. This pre-claim assistance consists of a series of letters being sent to the borrower, urging the borrower to contact the lender and begin or resume payments. The Secretary does not pay insurance on a default claim if the lender failed to request this pre-claim assistance.

(e) *Final demand letter.* A lender must send a final demand letter to the borrower at least 30 days before the lender files a default claim. The lender must allow the borrower at least 30 days to respond to the final demand letter. However, a lender need not send a final demand letter to a borrower whose address is unknown.

(f) *Litigation.* (1) If the borrower's loan is in default and the lender determines that the borrower has the ability to repay the loan, the lender may bring suit against the borrower to recover the amount of the unpaid principal and interest together with reasonable attorney's fees. Prior to bringing suit the lender shall—

(i) Obtain the Secretary's approval. A lender may seek the Secretary's approval to bring suit in anticipation that the lender's collection efforts will be unsuccessful. The Secretary will normally approve a lender's request to bring suit if the Secretary is satisfied that the borrower has the ability to repay the loan and that the collection efforts required by this section have been, or will be, made prior to the lender's bringing suit;

(ii) Notify the borrower that the Secretary's approval to bring suit has been obtained, and that suit will be brought unless the borrower cures the default; and

(iii) Indicate to the borrower that the lender will seek a judgment under which the borrower will be legally liable for payment of reasonable attorney's fees and court costs in addition to the unpaid principal and interest. The lender shall mail the notice to the borrower by certified mail, return receipt requested.

(2) The lender may bring suit if the borrower does not meet the terms of the lender's demand for payment within 10 days following the date of delivery of the notice to the borrower indicated on the receipt.

(3) A lender may first apply the proceeds of any judgment against its reasonable attorney's fees and court costs, whether or not the judgment provides for these fees and costs.

(20 U.S.C. 1078-2, 1080, 1082, 1085)

§ 683.61 Forbearance.

(a) *General.* (1) The Secretary encourages a lender under the Federal PLUS program to grant forbearance for the benefit of a borrower in order to prevent a borrower from defaulting on his or her payment obligations. "Forbearance" means permitting the temporary cessation of payments, allowing an extension of time for making payments, or accepting smaller payments than were previously scheduled.

(2) A lender may grant forbearance of payments of principal and/or interest under paragraph (b) or (c) whenever—

(i) poor health or other personal problems affect the ability of the borrower to make scheduled payments, or

(ii) the borrower's payments of principal are deferred.

If two borrowers are liable for repayment of a loan as co-makers, the lender shall grant forbearance only when the ability of both borrowers to make scheduled payments is adversely affected.

(3) If payments of interest are forborne they may be added to the principal amount of the loan obligation on the date that repayment resumes or at the end of the period of forbearance.

(b) *Basic repayment terms.* A lender may grant forbearance on terms that are consistent with the minimum annual payment requirement and the 10-year limitation on length of repayment if the lender and the borrower agree in writing to the new terms, or, in the case of forbearance of interest during a period of deferment, if the lender informs the borrower in writing at the time the deferment is granted that interest payments are to be forborne.

(c) *Inconsistent repayment terms.* A lender may also grant forbearance for a period of up to one year at a time on terms that are inconsistent with the minimum annual repayment requirement and the 10- and 15-year limitations on length of repayment if the lender complies with these requirements:

(1) The lender must reasonably believe that the borrower intends to repay the loan but is currently unable to make payments in accordance with the terms of the loan note. The lender shall state the basis for its belief in writing and maintain that statement in its loan file on that borrower.

(2) Both the borrower and an authorized official of the lender shall sign a written agreement of forbearance.

(3) If the agreement between the borrower and lender provides for postponement of all payments, the lender shall contact the borrower at least every 3 months during the period of forbearance in order to remind the borrower of the outstanding obligation to repay.

(20 U.S.C. 1078-2, 1080, 1082)

§ 683.62 Assignment of a loan.

(a) *General.* A Federal PLUS note may not be assigned except to another eligible lender. In this section "seller" means any kind of assignor, "buyer" means any kind of assignee, and "assignment" means any kind of transfer, including assignment as security.

(b) *Procedure.* (1) A Federal PLUS note assigned from one lender to another must be subject to a blanket endorsement together with other Federal PLUS notes being assigned or must individually bear effective words of assignment. Either the blanket endorsement or the note must be signed and dated by an authorized official of the seller.

(2) The buyer must—

(i) Notify the Secretary of the assignment if the right to receive special allowance has been assigned; and

(ii) Ensure that the borrower is notified promptly if the assignment results in the borrower being required to make installment payments, or direct other matters connected with the loan, to a party other than the party whom the borrower dealt with before the assignment. The buyer must include in the notice to the borrower a clear statement of all the borrower's rights and responsibilities which arise from the assignment of the loan, including a statement regarding the consequences of making payments to the seller or any prior holder of the loan, subsequent to receipt of the notice.

(c)(1) *Risks assumed by the buyer.* Upon acquiring a Federal PLUS note, a new holder assumes responsibility for the consequences of any previous violation of applicable statutes or regulations or the terms of the note. A Federal PLUS note is not a negotiable instrument, and a subsequent holder is not a holder in due course. If the borrower has a valid legal defense that could be asserted against the original holder, the borrower can also assert the defense against the new holder. If the new holder files a default claim on a loan, the Secretary denies the default claim if there was a legal defect affecting the initial validity or insurability of the loan and to the extent of the borrower's legal defenses. Furthermore, when a new holder files a claim on a Federal PLUS loan, it must provide the Secretary with the same documentation that would have been required of the original lender.

(2) *Special additional rules for assignment of loans made or originated by a school.* The buyer shall not be entitled to rely upon the statements provided by a school in the making or origination of a loan by the school. In addition, the Secretary considers any unpaid tuition refund that was due to the student under § 683.87 before the assignment from a school that made or originated the loan as having been paid to the subsequent holder on the borrower's behalf.

(d) *The Secretary's approval.* (1) The approval of the Secretary is required prior to the assignment of a note to any eligible lender which has not entered into a Federal PLUS insurance contract with the Secretary. The Secretary approves such an assignment only if the Secretary is satisfied that one of the parties to the assignment will comply with all the requirements applicable to lenders under the PLUS program regulations.

(2) Any arrangement where the loan is assigned to an eligible lender that would hold the loan in trust must receive the Secretary's prior approval. A lender that holds a loan as a trustee assumes responsibility for complying with all applicable statutory and regulatory requirements imposed on a holder of a loan.

(e) *Warranty.* (1) Nothing in this section precludes the buyer of a Federal PLUS loan from obtaining a warranty from the seller covering certain future reductions by the Secretary in computing the amount of insurable loss, if any, on a claim filed on the loan.

(2) The warranty may only cover reductions which are attributable to an act or failure to act of the seller or other previous holder.

(3) The warranty may not cover matters that the buyer is responsible for under the PLUS program regulations.

(20 U.S.C. 1078-2, 1079, 1080, 1082)

§ 683.63 Death, disability, and bankruptcy.

(a) *Death.* (1) If a borrower dies, the borrower's obligation to make any further payments of principal and interest on a Federal PLUS loan is cancelled. However, if the loan was obtained by two parents as co-makers, and only one of the borrowers dies, the surviving borrower remains obligated to repay the loan and the loan is not eligible for cancellation.

(2) The lender may not attempt to collect on the loan from the borrower's estate.

(3) The lender may make a determination that the borrower has died on the basis of a death certificate or other proof of death which is acceptable under applicable State law. If a death certificate or other acceptable proof of death is not available, the borrower's obligation on the loan is cancelled only upon a determination by the Secretary on the basis of other evidence that the Secretary finds conclusive.

(4) The lender shall return to the sender any payments received from the estate of the borrower or paid on behalf of the borrower after the date of death.

(b) *Disability.* (1) If the lender determines that a borrower is totally and permanently disabled, the borrower's obligation to make any further payments of principal and interest on a Federal PLUS loan is cancelled. However, if the loan was obtained by two parents as co-makers, and only one of the borrowers becomes totally and permanently disabled, the other borrower remains obligated to repay the loan and the loan is not eligible for cancellation. A borrower is not considered totally and permanently disabled on the basis of a condition that existed prior to his or her loan application unless the borrower's condition has substantially deteriorated since he or she submitted the loan application.

(2) After being notified by the borrower or the borrower's representative that the borrower claims to be totally and permanently disabled, the lender may not attempt to collect on the loan from the borrower. The lender shall promptly request that the borrower or his or her representative obtain a certification from a physician who is a doctor of medicine or osteopathy and legally authorized to practice, on a form provided by the Secretary, that the borrower is totally and permanently disabled. If the form is not submitted to the lender within 60 days of the date the lender requested it, the lender may resume collection unless the physician has notified the lender that a longer period of time is required to make the determination.

(3) If the lender receives a certification from a physician, as described in paragraph (b)(2), that the borrower is totally and permanently disabled, the lender must return to the borrower any payments that it may have received from or on behalf of the borrower after being notified that the borrower claims to be totally and permanently disabled.

(c) *Bankruptcy.* (1) If a borrower has filed a bankruptcy petition, the Secretary will assume the borrower's liability for unpaid principal and interest. However, if the loan was obtained by two parents as co-makers and only one of the borrowers has filed a bankruptcy petition, the other borrower remains obligated to repay the loan and the Secretary does not assume liability for unpaid principal and interest.

(2) Once a lender determines that a borrower has filed a bankruptcy petition, the lender may not attempt to collect on the loan and must file a bankruptcy claim with the Secretary.

(3) The lender may determine that a borrower has filed a bankruptcy petition upon receipt of notice of the first meeting of creditors from the bankruptcy court.

(4) If the loan obligation is not discharged in bankruptcy, the Secretary shall treat the claim as a default claim. The lender shall not be required to repurchase the loan.

(20 U.S.C. 1078-2, 1082, 1087)

§ 683.64 Cessation of lender collection activity in certain cases.

(a) A lender shall cease collection activity on a Federal PLUS loan, and file a default claim with the Secretary within 60 days after the lender determines that any of the following conditions exist, whether or not the borrower is eligible for deferment:

(1) The school in which the student on whose behalf the loan was made was enrolled terminated its teaching activities involving that student during the academic period covered by the loan.

(2) The Secretary—

(i) Has instituted an action to limit, suspend, or terminate the eligibility of the school in which the student was enrolled for the academic period covered by the loan, or the eligibility of any lender that has held the loan; and

(ii) Has directed that a claim be filed on the loan.

(3)(i) A school or a lender is the subject of a lawsuit or Federal administrative proceeding and the Secretary determines that the proceeding involves allegations that, if proven, would entitle the borrower to refuse to repay all or a portion of the loan, or to obtain a judgment to recover payments made on the loan, or would entitle the student to take such action if the loan had been made to the student; and

(ii) The Secretary has directed that a claim be filed on the loan.

(b)(1) If the Secretary finds that a determination made by a lender under this section is correct, the Secretary pays the default claim as otherwise provided for under these regulations.

(2) If the Secretary finds that the lender's determination is not correct, the Secretary refuses payment and the lender shall resume normal collection activity on the loan.

(c) A lender may not, as a result of a default claim filed with the Secretary under this section, make a report to any credit bureau or other third party concerning the borrower's failure to repay his or her loan.

(20 U.S.C. 1078-2, 1080, 1082)

§ 683.65 Procedures for filing claims.

(a)(1) A lender may file an insurance claim for any of the following reasons:

(i) The loan is in default. A loan is not in default until the 120-day or, if applicable, the 180-day period, described in the definition of "default" in § 683.10, has elapsed. If the borrower fails to make an installment payment when due, the 120- or 180-day period begins the day after the due date of that installment.

(ii) Any of the conditions for filing a default claim without collection efforts exist, as set forth in §§ 683.64 and 683.66(e).

(iii) The borrower has died.

(iv) The borrower is totally and permanently disabled.

(v) The borrower has filed a bankruptcy petition.

(2) If a loan was obtained by two eligible parents as co-makers, the applicable condition described above must apply to both parents.

(b) *Filing a claim application.* A lender shall file an insurance claim on a form provided by the Secretary. The lender shall attach to the claim all documentation that the Secretary may require. Failure to submit the required documentation may result in a claim not being honored. The Secretary may also deny a claim that is not filed on time.

(c) *Documentation required for all PLUS program claims.* The Secretary requires the following documentation for all claims:

(1) The original promissory note.

(2) The loan application.

(3) A payment history, as described in § 683.68(a)(1)(viii), if any payments have been made.

(4) A collection history, as described in § 683.68(a)(1)(ix).

(d) *Assignment of note.* The Secretary's payment of a claim is contingent upon receipt of an assignment to the United States of America of all right, title, and interest of the lender in the note underlying the claim. The lender shall agree to reimburse the Secretary for any overpayments of special allowance that the Secretary may have made for the loan.

(e) *Specific procedures applicable to the individual claim categories.* A lender must also comply with the following requirements for filing default, death, disability, and bankruptcy claims:

(1) *Default claims.* (i) Unless a lender has notified the Secretary that it has filed suit against the defaulted borrower, after obtaining the Secretary's approval for the suit, it must file a default claim with the Secretary within 90 days after the loan has been determined to be in default, or the lender has determined that any of the conditions for filing a default claim without collection efforts exist, as set forth in §§ 683.64 and 683.66(e).

(ii) In addition to the documentation required for all claims, the lender must submit with its default claim the following:

(A) A copy of the final demand letter, if required under § 683.60(e).

(B) The original or a copy of all personal correspondence addressed to or from, or on behalf of, the borrower relevant to the amount owed by the borrower, whether that correspondence involved the original lender, a subsequent holder, or an independent servicing agency.

(C) Evidence of the lender's requests to the Department of Education for pre-claim assistance and, if a request was required under § 683.60(c), skip-tracing assistance.

(iii) If the lender files a default claim on a loan and subsequently receives a notice of the first meeting of creditors in the proceeding of the borrower in bankruptcy, the lender shall promptly forward that notice to the Department of Education. The lender may not file a proof of claim with the bankruptcy court in this situation.

(2) *Death claims.* A lender shall file a death claim with the Secretary within 60 days after the lender determines that a borrower is dead. In addition to the documentation required for all claims, the lender shall submit with its death claim those documents which formed the basis for its determination of death.

(3) *Disability claims.* A lender shall file a disability claim with the Secretary within 60 days after it receives a certification from a licensed physician that a borrower is totally and permanently disabled. In addition to the documentation required for all claims, the lender shall submit with its disability claim a copy of the certification.

(4) *Bankruptcy claims.* A lender shall file a bankruptcy claim with the Secretary within 60 days after the lender receives a notice that the borrower has filed a bankruptcy petition. In addition to the documentation required for all claims, the lender shall submit with its bankruptcy claim to the Secretary the following:

(i) An assignment to the United States of America of its proof of claim.

(ii) Evidence that a bankruptcy petition has been filed and all pertinent documents sent to or received from the bankruptcy court.

(iii) A statement of any facts of which the lender is aware that may form the basis for an objection to the bankrupt's discharge or an exception to the discharge.

(20 U.S.C. 1078-2, 1080, 1082, 1087)

§ 683.66 Determination of amount of loss on claims.

(a) *General.* The amount of loss to be paid on a claim for a Federal PLUS loan shall be equal to the unpaid balance of the principal and interest. The unpaid principal amount of the loan may include capitalized interest.

(b) *Payment of insured interest.* (1) The payment of an approved claim covers the unpaid interest that accrues during the following periods:

(i) During the period before the claim is filed, not to exceed the period permitted under paragraph (e) of § 683.65 for filing the claim.

(ii) During a period not to exceed 30 days following the return of the claim to the lender by the Secretary for additional documentation necessary for the claim to be approved by the Secretary.

(iii) During the period required by the Secretary to approve the claim and to authorize payment.

(2) The Secretary pays the unpaid interest that accrues during other periods which are tied to the type of claim involved:

(i) The payment on a default claim covers unpaid interest that accrues through the date of default.

(ii) The payment on a bankruptcy claim covers unpaid interest that accrues before the lender receives notice that the borrower filed a bankruptcy petition.

(iii) The payment on a death claim covers unpaid interest that accrues before the lender determines that the borrower is deceased.

(iv) The payment on a disability claim covers unpaid interest that accrues before the lender receives a certification from a physician that the borrower is totally and permanently disabled.

(c) *Factors affecting the insurability of a loan.* (1) In determining whether to approve an insurance claim for payment, the Secretary considers legal defects affecting the initial validity or insurability of the loan.

(2) The Secretary also deducts from a claim any amount that is not a legally enforceable obligation of the borrower.

(3) The Secretary further considers whether all holders of the loan have complied with the requirements of the Federal PLUS regulations, including those concerned with making and collecting a loan, the timely filing of a claim, and the submission of documents with a claim.

(4) The Secretary does not pay a death, disability, or bankruptcy claim for a loan after a default claim for that loan has been disapproved by the Secretary.

(d) *Special rules for a loan acquired by assignment.* If a claim is filed by a lender that obtained a loan by assignment, that lender is not entitled to any payment under this section greater than that to which a previous holder would have been entitled. In particular, the Secretary deducts from the claim any amounts that are attributable to payments made by the borrower to a prior holder of the loan before the borrower received proper notice of the assignment of the loan.

(e) *Special rules for loans made by school lenders.*

(1) If the loan for which a claim is filed was originally made by a school and the claim is filed by that school, the Secretary deducts from the claim—

(i) An amount equal to any unpaid refund that the school owes the student to whom or on whose behalf the loan was made under § 683.87; or

(ii) An amount attributable to any portion of the program of study that the student was unable to complete because the school terminated its teaching activities during the period of time for which the borrower obtained a Federal PLUS loan. If this situation occurs, the lender shall immediately file a default claim with the Secretary. The Secretary reimburses the lender in an amount which bears the same ratio to the total amount of the claim as the amount of the educational services that the student received before the school terminated its teaching activities bears to the total services which the student would have received, during the period for which the loan was obtained, had the school not terminated its teaching activities.

(2) If the loan for which a claim is filed was originally made by a school but the claim is filed by another lender that obtained the note by assignment, the Secretary deducts from the claim—

(i) An amount equal to any unpaid refund that the school owed the student to whom or on whose behalf the loan was made under § 683.87 prior to the assignment of the loan to a subsequent holder;

(ii) An amount attributable to any portion of the program of study that the student was unable to complete because the school terminated its teaching activities during the period of time for which the borrower obtained a Federal PLUS loan. If this situation occurs, the lender shall immediately file a default claim with the Secretary. The Secretary reimburses the lender in an amount which bears the same ratio to the total amount of the claim as the amount of the educational services that the student received before the school terminated its teaching activities bears to the total services which the student would have received, during the period for which the loan was obtained, had the school not terminated its teaching activities.

(f) *Special rules for a loan originated by a school.* For purposes of this section, a loan which is originated by a school shall be treated in accordance with paragraph (e)(1) of this section as if it were a loan made and still held by a school.

(g) *Circumstances under which defects in claims may be cured or excused.* (1) The Secretary may permit a lender to cure certain defects in a specified manner as a condition for payment of a default claim.

(2) The Secretary may excuse certain defects—

(i) If the holder submitting the default claim satisfies the Secretary that the defect did not contribute to the default or prejudice the Secretary's attempt to collect on the loan from the borrower; or

(ii) If the defect arose while the holder submitting the default claim was holding the loan but the Secretary had previously found that the holder had procedures in effect sufficient to ensure that such a defect would not normally arise.

(3) The Secretary may also excuse certain defects if the Secretary is satisfied that—

(i) The defect arose while the loan was held by another lender;

(ii) The assignment of the loan was an arm's length transaction;

(iii) The present holder did not know of the defect at the time of the assignment; and

(iv) (A) The present holder could not have become aware of the defect through an examination of the loan documents; or

(B) The present holder had relied on a finding by the Secretary that the lender holding the loan when the defect arose had procedures in effect sufficient to ensure that such a defect would not normally arise.

(20 U.S.C. 1078-2, 1080, 1082)

§ 683.67 The Secretary's collection efforts after payment of a default claim.

After paying a default claim on a Federal PLUS loan, the Secretary attempts to collect from the borrower in accordance with the Federal Claims Collection Standards (4 CFR Parts 101-105). The Secretary attempts collection of all unpaid principal and accrued interest, except in the following situations:

(a) *The borrower has a valid defense on the loan.* In this situation, the Secretary refrains from collection against the borrower to the extent of any defense that the borrower may have.

(b) *A school owes the student to whom or on whose behalf the loan was made a refund for the period covered by the loan.* In this situation, the Secretary refrains from collection to the extent of the unpaid refund the school owes the student on whose behalf the loan was

made under § 683.87, if the student assigns to the Secretary the right to receive the refund and the borrower agrees in writing to pay the Secretary the remaining portion of his or her indebtedness on the loan.

(c) *The school attended by the student to whom or on whose behalf the loan was made closes during the academic period covered by the loan.* (1) In this situation, the Secretary refrains from collection against the borrower to the extent that the student would have had a defense on the loan if the loan was—

(i) Made by the school to the student;

(ii) Part of the same transaction as the enrollment at the school of the student; and

(iii) Paid to the school in consideration for the educational services that were to be provided by the school.

(2) As a condition of this forgiveness—

(i) The student must assign to the Secretary the right to receive any refund that the school owes the student under § 683.87; and

(ii) The borrower must agree in writing to pay the Secretary the remaining portion of his or her indebtedness on the loan.

(d) *A school or lender is the subject of a lawsuit or Federal administrative proceeding.* In this situation, if the Secretary determines that the proceeding involves allegations that, if proven, would provide the borrower with a full or partial defense on the loan, or, if the borrower is a parent, would provide the student on whose behalf the loan was made with such a defense if the loan had been made to the student, then the Secretary may suspend collection activity on all or part of a loan until the proceeding ends. The Secretary suspends collection activity only for so long as the Secretary believes that the proceeding is being prosecuted in good faith and that the allegations that relate to the borrower's or student's defense are reasonably likely to be proven. When a final resolution is reached, the Secretary collects from the borrower to the extent appropriate.

(e) *A school or lender is the subject of a limitation, suspension, or termination action by the Secretary.* In this situation, if the Secretary determines that the final outcome of the action could provide the borrower with a full or partial defense on the loan, or, if the borrower is a parent, would provide the student on whose behalf the loan was made with such a defense if the loan had been made to the student, then the Secretary may suspend collection activity pending the final resolution of the action. When a final resolution is reached, the Secretary collects from the borrower to the extent appropriate.

(f) *The borrower dies, becomes totally and permanently disabled, or has the Federal PLUS loan discharged in bankruptcy.* In this situation, the Secretary terminates all collection activity against the borrower. However, if the loan was obtained by two eligible parents as co-makers, and only one of the borrowers meets one of these conditions, the Secretary continues collection activity against the other borrower.

(20 U.S.C. 1078-2, 1080, 1082)

§ 683.68 Records, reports, and inspection requirements for lenders.

(a) *Records.* (1) A lender shall keep complete and accurate records of each Federal PLUS loan which it holds. The records must be organized in a way that permits ready identification of the current status of each loan. The required records include—

(i) The loan application;

(ii) The original promissory note until it is paid in full, after which a copy is required;

(iii) A record of each disbursement of loan proceeds;

(iv) Notices of changes in a borrower's address;

(v) Evidence of the borrower's eligibility for a deferment;

(vi) The documents required for the exercise of forbearance;

(vii) Documentation of the assignment of the loan;

(viii) A payment history showing the date and amount of each payment received from or on behalf of the borrower, and the amounts attributable to principal and interest;

(ix) A collection history showing the date and subject of each communication with the borrower for collection of a delinquent loan; and

(x) Any additional records as specifically required by these regulations which are necessary to document the validity of an insurance claim or to make any reports required by the Secretary under these regulations.

(2)(i) A lender shall retain the records required for each loan for not less than 5 years following the date the loan is repaid in full by the borrower or the lender is reimbursed on a claim. However, in particular cases the Secretary may require the retention of records beyond this minimum period.

(ii) The lender may store records in microfilm or computer format. However, the holder of a promissory note must retain the original note until the loan is fully repaid. At that time the lender shall return the original note to the borrower, and retain copies for the prescribed period.

(b) *Reports.* A lender shall submit reports to the Secretary at the time and in the manner the Secretary may reasonably require, including but not limited to the following:

(1) The Lender's Manifest.

(2) The Lender's Request for Payment of Interest and Special Allowance.

(3) The Lender's Annual Report on Outstanding Loans.

(c) *Inspections.* Upon request, a lender shall afford the Secretary, the Comptroller General of the United States, and any of their authorized representatives access to its records in order to assure the correctness of its reports.

(20 U.S.C. 1077, 1078, 1078-2, 1079, 1080, and 1082)

Subpart E—Requirements, Standards, and Payments for Participating Schools

§ 683.80 Participation agreement between an eligible school and the Secretary for participation in the PLUS program.

(a) *General.* Participation of a school in the PLUS program means that its students and the parents of the school's dependent undergraduate students are eligible to receive PLUS loans for the cost of attendance of students at that school. To participate in the PLUS program, under either the Federal PLUS program or a guarantee agency program, a school must—

(1) Establish its basic eligibility as an institution of higher education or a vocational school, as defined in § 683.10, through certification by the Division of Eligibility and Agency Evaluation, Office of Postsecondary Education, Department of Education; and

(2) Enter into a written agreement with the Secretary. The agreement must be signed by an appropriate official of the school on a form provided by the Secretary.

(b) *Program participation agreement.* In the agreement, the school promises to comply with the applicable provisions of—

(1) The Act and the PLUS regulations;

(2) 34 CFR Part 668 (Student Assistance General Provisions); and

(3) 34 CFR Part 678 (Student Consumer Information Services).

(c) *Time to respond.* The Secretary responds to a school's request for an agreement to participate in the PLUS program within 30 days after receiving the request.

(d) *Denial or limitation of participation.* (1) If the Secretary

decides not to approve a request for an agreement or approves only limited participation in the PLUS program by the school, the reason for the decision is included in the response.

(2) The Secretary provides an opportunity for the school to meet with a designated Department of Education official, if the school wishes to appeal a decision involving either—

(i) Denial of an agreement for participation; or

(ii) Approval of an agreement that limits the school's participation.

(3) The Secretary does not, however, grant an opportunity for appeal or give reasons for denying the participation, or approving only the limited participation, of a school if the school submits its request within 6 months of a previous denial or limited approval for either the PLUS program or the GSLP.

(e) *Change in ownership or form of control.* A PLUS program participation agreement automatically terminates when a school changes its ownership or form of control. The termination is effective at the time the change occurs. A new agreement must be signed and approved by the Secretary for the school to participate under the new ownership or form of control.

(f) *Extension of current agreements.* Until the Secretary makes a PLUS program participation agreement available, a school that has entered into an agreement with the Secretary to participate in the GSLP may participate in the PLUS program in accordance with applicable provisions of these regulations.

(20 U.S.C. 1078-2, 1082, 1094)

§ 683.81 Agreement between the Secretary and a school that makes or originates PLUS loans.

(a) *General.* (1) A school must have an agreement with the Secretary in order to make or originate PLUS loans under either the Federal PLUS program or a guarantee agency PLUS program. The definition of origination is in § 683.10.

(2) *Extension of current agreements.* Until the Secretary makes a PLUS program origination agreement available, a school that has entered into an agreement with the Secretary to make or originate loans in the GSLP may make or originate loans under the PLUS program in accordance with applicable provisions of these regulations.

(b) *Terms of the agreement.* An agreement to allow a school either to make or originate loans contains the following terms:

(1) The school will not make or originate GSLP or PLUS loans which would be outstanding to or on behalf of more than 50 percent of its undergraduate students who are in attendance at that school on at least a half-time basis or to the parents of such students. An exception to this rule, however, is contained in paragraph (d) of this section.

(2) The school will inform any undergraduate student or parent who seeks to obtain a PLUS loan from the school that unless the school has previously made or originated a PLUS loan to or on behalf of that student, the borrower must first make a good faith effort to obtain a loan from a commercial lender.

(3)(i) The school will not make or originate a loan for an academic period to a borrower described in paragraph (b)(2) of this section until the borrower provides the school with evidence of denial of a loan by a commercial lender for the same academic period. Evidence acceptable for this purpose is described in paragraph (c) of this section.

(ii) In determining whether a school has complied in good faith with this requirement, the Secretary may take into consideration any pattern reflected by letters of denial or borrowers' statements referred to in paragraph (c) of this section that indicate that the school has not given sufficient counseling to borrowers to seek loans first from a commercial lender. An example of an unacceptable pattern would be if all loan denials to the students or parents of a school's students were made by a small number of lenders.

(c) *Establishing a loan denial by a commercial lender.* (1) To ensure under paragraph (b)(3) of this section that a borrower has sought and been denied a loan from a commercial lender for an academic period, the school shall obtain from the borrower—

(i) A written statement from a commercial lender indicating that the lender denied the borrower a loan for that academic period; or

(ii) The borrower's statement, made under penalty of perjury, indicating both the refusal of a loan by a commercial lender and that lender's refusal to provide a written statement of the denial.

(2) If the borrower statement, is used to establish the denial of a loan, that statement must include—

(i) The name of the lender that denied the loan;

(ii) The approximate date on which the loan was denied;

(iii) The name of the official who communicated the denial to the borrower and

(iv) The borrower's signature. The statement must be signed by the borrower under penalty of perjury.

(3) The refusal of a lender to make a loan to a borrower for the entire amount requested by the borrower constitutes a denial of a loan, if the school determines that the borrower is eligible for a loan of that amount. If the denial is based upon the borrower's inability to obtain the entire amount requested, the school may either—

(i) Make or originate a loan to that borrower for the entire amount; or

(ii) Supplement the loan that the commercial lender is willing to make with a second loan to the borrower.

(d) *Waiver of the 50 percent lending limit.* A school may request a waiver of the 50 percent lending limit under paragraph (b)(1) if adherence to that limit would create a substantial hardship to the school's present or prospective students or their parents. The Secretary determines whether to grant the school a waiver after considering the following:

(1) The extent to which the school provides, and expects to continue providing, educational opportunities to economically disadvantaged students, as measured by the percentage of these students enrolled at the school who—

(i) Fall within the "low-income family" category used by the Bureau of the Census;

(ii) Would not be able to enroll, or continue their enrollment, at that school without a GSLP or PLUS loan made or originated by the school; and

(iii) Would not be able to obtain a comparable education at another school.

(2) The extent to which the school offers academic programs that—

(i) Are unique in the geographical area the school serves; and

(ii) Would not be available to some students if the school adhered to the 50 percent lending limit.

(3) The quality of improvements expected in the school's—

(i) Management of student financial assistance programs; and

(ii) Conformance with sound business practices.

(20 U.S.C. 1075, 1078, 1078-2, 1082, 1083)

§ 683.82 Providing information to prospective students.

(a) *General.* (1) A school shall present each of its prospective students with a complete and accurate statement containing information about the school. The statement must be in written form and must be presented to the prospective student prior to the time that he or she becomes obligated to pay the school any tuition or fees.

(2) The statement provided by the school must include information pertaining to—

(i) The school's current academic or training programs in which the student has expressed interest;

(ii) The school's faculty in those programs; and

(iii) The school's facilities relating to those programs.

(b) *Providing employment data.* In addition to the information required by paragraph (a) of this section, a school that offers programs or courses of study designed to prepare students for a particular vocational, trade or career field (e.g., truck driving, teaching or pharmacy) shall provide a prospective student in that field with a written statement regarding the employment of students previously enrolled in those programs or courses.

(1) The employment information must include data regarding the percentage of previously enrolled students who entered positions of employment directly related to their enrollment at the school and data regarding the average starting salaries of those students.

(2) The school may provide the prospective student with the most recent comparable regional or national statistical student employment data in lieu of the information about the school's own students if—

(i) After a reasonable effort, the school cannot obtain meaningful data on the employment of its own students; or

(ii) The data the school possesses regarding its own students is more than 3 years old and cannot, after a reasonable effort, be updated.

(3) To the extent that information is available, the school should provide a prospective student with information regarding the long-range prospects for employment in the particular vocational, trade or career field that the student intends to prepare for at the school.

(20 U.S.C. 1078-2, 1082, 1085, 1094)

§ 683.83 Correspondence school schedule requirements.

(a) *General.* A school offering a course of study by correspondence shall establish a schedule for submission of lessons by its students. This schedule must be given to a prospective student prior to that person's enrollment.

(b) *Information in the schedule.* The school shall include the following information in its schedule:

(1) The number of lessons in the course.

(2) The intervals at which lessons are to be submitted.

(3) The date by which the course is to be completed.

(4) The period of time within which any resident training must be completed.

(c) *Additional requirements.* The schedule must conform to the requirements set forth in paragraph (a)(3)(ii) of the definition of "vocational school" in § 683.10.

(20 U.S.C. 1078-2, 1082)

§ 683.84 Certifications by a participating school in connection with a PLUS loan application.

A school shall accurately and completely fill out its portion of a PLUS loan application. In determining whether an undergraduate student is a dependent or an independent student, the school may rely in good faith and in the absence of information to the contrary upon statements of the student. The information requested of the school pertains to the following:

(a) The student's eligibility for a loan, as determined in accordance with § 683.11.

(b) The student's eligibility to have a parent borrow on his or her behalf, as determined in accordance with § 683.11, if the borrower is a parent.

(c) The parent's eligibility for a loan, as determined in accordance with § 683.11, if the borrower is a parent.

(d) The student's estimated cost of attendance for the period for which the loan is sought.

(e) The student's estimated financial assistance for the period for which the loan is sought.

(20 U.S.C. 1077, 1078, 1078-2, 1085, 1094)

§ 683.85 Reserved.

§ 683.86 Reserved.

§ 683.87 Refund policy.

(a) *General.* (1) A school shall have a fair and equitable refund policy under which it will make a refund of unearned tuition, fees and room and board charges to a student who received a PLUS loan or whose parent received a PLUS loan on his or her behalf if the student—

(i) Does not enroll for the academic period for which the loan was intended; or

(ii) Does not complete the academic period for which the loan was made.

(2) The school shall state its refund policy clearly in writing. The school shall include in its refund policy the procedure a student would follow to obtain a refund.

(3) The school shall provide the written statement containing its refund policy to a prospective student prior to the student's acceptance for initial enrollment. The school shall also make its refund policy known to currently enrolled students. If the school changes its refund policy, the school shall ensure that all students are made aware of the new policy.

(b) *Fair and equitable refund policy.* A school's refund policy is fair and equitable if that policy conforms with—

(1) The requirements of applicable State law; and

(2)(i) Specific refund standards set by the school's nationally recognized accrediting agency and approved by the Secretary; or

(ii) If no such standards exist, other specific refund policy standards, either contained in Appendix A to this Part or set by another association of institutions of postsecondary education and approved by the Secretary.

(20 U.S.C. 1078-2, 1082, 1094)

§ 683.88 Determining the date of a student's withdrawal.

(a) *Purpose.* This section establishes rules for how a school must determine the date (to include day, month and year) on which a student withdraws from the school for the purpose of calculating the amount of a refund due the student.

(b) *The withdrawal date.* The school shall establish the date of a student's withdrawal as follows:

(1) Generally, the student's withdrawal date is the earlier of—

(i) The date the student notifies the school of his or her withdrawal; or

(ii) The date the school determines that a student has withdrawn.

Paragraphs (b)(2) and (b)(3) contain additional rules applicable to particular situations.

(2) If the student has not returned to school at the expiration of a leave of absence approved under paragraph (c), the student's withdrawal date is the date of the first day of the leave of absence.

(3) If the student is enrolled in a program of study by correspondence, the student's withdrawal date is normally 60 days after the due date of a required lesson that the student failed to submit in accordance with the schedule for lessons established by the school under § 683.83. However, if the student establishes in writing, within the 60-day period, a desire to continue in the program and an understanding that the required lessons must be submitted on time, the school may grant that student a restoration to in-school status. However, the school may not grant the student more than one restoration to in-school status on this basis.

(c) *Leaves of absence.* A student who is absent from school and who has been granted a leave of absence by the school, in accordance with this paragraph, is not considered to have withdrawn from school for purposes of

this section. A school may grant a leave of absence to a student provided—

(1) The student has made a written request to be granted a leave of absence;

(2) The leave of absence involves no additional charges by the school to the students;

(3) The leave of absence does not—

(i) Exceed 60 days; or

(ii) Exceed 6 months if either of the following circumstances exists:

(A) The school is not a correspondence school and the school's next period of enrollment after the start of the leave of absence would begin more than 60 days after the first day of the leave of absence; or

(B) The absence is requested because of the student's medically determinable conditions. In this case, the student must provide the school with a recommendation from a physician for a leave of absence longer than 60 days; and

(4) The student has not previously been granted a leave of absence by the school. Additional leaves of absence for a student must be approved by the Secretary.

(20 U.S.C. 1078-2, 1082, 1094)

§ 683.89 Payment of a refund to a lender.

(a)(1) A school shall pay that portion of the student's refund that is allocable to a PLUS loan to—

(i) The original lender; or

(ii) A subsequent holder, if the loan has been transferred and the school knows the new holder's identity.

(2) When the school pays refund monies to a lender on behalf of a student who received a PLUS loan or whose parent received a PLUS loan on his or her behalf, the school shall provide simultaneous written notice of the action to the borrower and, if the borrower is a parent, the student on whose behalf the loan was made.

(b) *Calculating what portion of the refund to allocate to the loan.* In determining what portion of a student's refund for an academic period is allocable to a PLUS loan received by the borrower for the same academic period, the school must follow the procedures established in 34 CFR Part 668 (Student Assistance General Provisions).

(c) *Timely payment of refund.* A school shall pay each refund that is due in accordance with the following:

(1) Within 40 days after the date of the student's withdrawal from the school, as determined in accordance with § 683.88(b); or

(2) In the case of a student who does not return to school at the expiration of an approved leave of absence (see § 683.88(b)), within 40 days after the last day of that leave of absence.

(d) *Transition requirements.* In the event of a school's closure, termination, suspension of operations, or change in ownership, the school or its successors shall make provision for compliance with the requirements of this section with regard to students who obtained or on whose behalf parents obtained loans for periods of attendance at the school prior to the school's change in status.

(20 U.S.C. 1078-2, 1082, 1094)

§ 683.90 Termination of a school's lending eligibility.

(a) *General.* The Secretary terminates a school's eligibility to make GSLP and PLUS loans, under the Federal PLUS program, the FISLP or a guarantee agency program, if the school reaches the 15 percent limit on loan defaults described in paragraph (b) of this section.

(b) *The 15 percent limit.* (1) The Secretary terminates a school's eligibility to make GSLP and PLUS loans if, during each of the two most recent consecutive one-year periods for which data is available, the total amount of loans described in paragraph (b)(1)(i) of this section is equal to or greater than 15 percent of the total amount of loans described in paragraph (b)(1)(ii) of this section.

(i) The original principal amount of loans the school has ever made that went into default during that period.

(ii) The original principal amount of all loans the school has ever made, including loans in deferment status, that—

(A) Were in repayment status at the beginning of that period; or

(B) Entered repayment status during that period.

(2) In making the determination required by this section, the Secretary considers the status of all GSLP and PLUS loans made by the school, whether the loans are held by the school or a subsequent holder.

(c) *Exception based on hardship.* The Secretary does not terminate a school's lending eligibility under paragraph (a) of this section if the Secretary determines that the termination would result in a hardship for the school or its students. The Secretary makes this determination if the school shows that—

(1) Termination is not justified in light of recent improvements the school has made in its collection capabilities that will cause the school's loan delinquency rate to improve within the next year. Examples of these improvements include the following:

(i) Adopting more efficient collection procedures.

(ii) Employing increased collection staff; or

(2) Termination would cause a substantial hardship to the school's current or prospective students or their parents based on—

(i) The extent to which the school provides, and expects to continue to provide, educational opportunities to economically disadvantaged students, as measured by the percentage of students enrolled at the school who—

(A) Fall within the "low-income family" category used by the Bureau of the Census;

(B) Would not be able to enroll, or continue their enrollment, at that school without a GSLP or PLUS loan from the school; and

(C) Would not be able to obtain a comparable education at another school.

(ii) The extent to which the school offers academic programs that—

(A) Are unique in the geographical area the school serves; and

(B) Would not be available to some students if they or their parents could not obtain loans from the school.

(iii) The quality of improvements the school has made in its—

(A) Management of student financial assistance programs; and

(B) Conformance with sound business practices.

(d) *Termination procedures.* The Secretary does not terminate the lending eligibility of a school under this section until the school has been notified of the impending action and has had an opportunity for a hearing.

(1) *The termination notice.* A Department of Education official designated by the Secretary begins a termination action by sending a notice to the school. The notice is sent by certified mail with a return receipt requested. In the notice, the designated official—

(i) Informs the school of the intent to terminate the school's lending eligibility because of the school's default experience;

(ii) Specifies the proposed effective date of the termination as the next October 1;

(iii) Informs the school that it has 15 days to do the following—

(A) Submit any written material it wants considered in determining whether its lending eligibility should be terminated under paragraph (a) of this section, including written material in support of a hardship exception under paragraph (c) of this section; or

(B) Request a hearing to show why the school should not be terminated.

(2) *If a hearing is not requested.* If the school does not request a hearing but

submits written material, the designated official considers that material and notifies the school as to whether the termination action will be taken.

(3) *The hearing.* The designated official schedules the date and place of a hearing for a school that has requested a hearing. The date of the hearing is at least 15 days from the date that the designated official received the request.

(i) A presiding officer (defined in § 683.101) conducts the hearing.

(ii) The presiding officer considers all written material presented before the hearing and any other material presented during the hearing.

(iii) The presiding officer determines if termination of the school's lending eligibility is warranted.

(4) *Review of a termination of a school's lending eligibility.* The decision of the presiding officer, or of the designated official, in the event that the school has submitted written material but has not requested a hearing, is subject to review by the Secretary.

(e) *Reinstatement of lending eligibility.* (1) A school that has its lending eligibility terminated under this section may not make further GSLP or PLUS loans unless it has entered into a new lending agreement with the Secretary under § 683.81.

(2) A new agreement may not take effect until at least one year after a school's lending eligibility has been terminated under this section.

(f) *Schools under the same ownership.* If a school makes loans to students or parents of such students in attendance at other schools under the same ownership, the Secretary may make the determinations required by this section by—

(1) Treating all the schools as one; or

(2) Treating each school on a school-by-school basis.

(20 U.S.C. 1078-2, 1082, 1085)

§ 683.91 Records, reports and inspection requirements for participating schools.

(a) *General.* (1) Each school shall establish and maintain proper administrative and fiscal procedures and all necessary records, as set forth in these regulations and 34 CFR Part 668 (Student Assistance General Provisions), in order to—

(i) Protect the rights of parents and students;

(ii) Protect the United States from unreasonable risk of loss due to defaults; and

(iii) Comply with any specific requirements in these regulations and 34 CFR Part 668.

(2) Each school shall submit such reports as the Secretary determines are necessary to comply with these regulations and 34 CFR Part 668.

(b) *Loan record requirements.* In addition to records required by 34 CFR Part 668, for each loan received by its students or the parents of its students a school shall maintain a record of—

(1) The name of the borrower;

(2) If the borrower is a parent, the name of the student on whose behalf the loan was made;

(3) The name of the lender;

(4) The address of the lender;

(5) The amount of the loan;

(6) The period for which the loan was intended;

(7) The data used to construct an individual student budget or the school's itemized standard budget used in calculating the student's estimated cost of attendance;

(8) The amount of the student's tuition and fees paid for that period; and

(9) The date the student paid the tuition and fees.

(c) *Reporting Student Status.* (1) When a school is notified that a PLUS loan has been made to or on behalf of a student who has been accepted for enrollment at that school but who fails to enroll on at least a half-time basis for the period for which the loan was intended, the school should immediately report this information to the lender.

(2) When a school becomes aware that a full-time student to whom a PLUS loan was made has ceased to be enrolled on a full-time basis, the school should immediately report this information to the lender.

(d) *Retention requirement for records and reports.*

(1) Unless otherwise directed by the Secretary, the school shall keep all records required under these regulations for 5 years, following the last date of the period for which the loan was intended.

(2) Unless otherwise directed by the Secretary, the school shall also keep, for 5 years after their completion, copies of reports and other forms utilized by the school related to PLUS loans.

(3) In the event of the closure, termination, suspension or change of ownership of a participating school, that school or its successor must make provision for the retention of the records and reports required by these regulations and for access to these records and reports for purposes of paragraph (e) of this section.

(4) Records and reports may be kept on microfilm or computer format.

(e) *Federal audits.* For purposes of audit and examination, the school shall give the Secretary, the Comptroller General of the United States, or any of their duly authorized representatives access to records required by these regulations and by 34 Part 668 and to any other pertinent books, documents, papers and records.

(f) *Non-Federal audits.* (1) The school shall, in conformance with 34 CFR Part 668, audit or have audited under its direction, all of the school's PLUS transactions to determine at a minimum—

(i) The fiscal integrity of financial transactions and reports; and

(ii) Whether the transactions are in compliance with the applicable laws and regulations.

(2) Audits shall be performed in accordance with the Department of Education's "Audit Guide for the Guaranteed Student Loan Program."

(3) The school shall have an audit performed at least once every two years. Each audit must cover the entire period of time that elapsed since the last audit that was performed.

(4) The school shall submit the audit report to the appropriate regional office of the Department of Education's Audit Agency for review.

(20 U.S.C. 1078-2, 1082, 1083, 1094)

Subpart F—Limitation, Suspension, or Termination of Lender Eligibility Under the Federal PLUS Program

§ 683.100 Purpose and scope.

(a) This subpart establishes rules for the limitation, suspension, or termination of the eligibility of an otherwise eligible lender to participate in the Federal PLUS Program. These rules apply to a lender that violates any provision of the Federal PLUS program statute or any regulation, special arrangement, agreement, or limitation prescribed under the Federal PLUS program.

(b) This subpart does not apply to a determination that an organization fails to meet the definition of "lender" in § 683.10, nor to a school's loss of lending eligibility due to its default experience under § 683.90.

(c) This subpart also does not apply to administrative action by the Department of Education, based on any alleged violation of—

(1) Title VI of the Civil Rights Act of 1964, which is governed by 34 CFR Parts 100 and 101;

(2) Title IX of the Education Amendments of 1972 (relating to sex discrimination), which is governed by 34 CFR Part 106; or

(3) The Family Educational Rights and Privacy Act of 1974 (§ 438 of the General Education Provisions Act, as amended) which is governed by 34 CFR Part 99.

(20 U.S.C. 1078-2, 1080, 1082, 1094)

§ 683.101 Definitions of terms used in this subpart.

Designated ED official: An official of the United States Department of Education to whom the Secretary has delegated the responsibility for initiating and pursuing limitation, suspension, and termination procedures.

Limitation: The continuation of a lender's eligibility, subject to compliance with special conditions set by the Secretary as a result of a limitation or termination proceeding.

Presiding officer: An impartial person who has no prior involvement with the facts giving rise to a limitation, suspension or termination proceeding, and who is selected by the Secretary to conduct a hearing.

Suspension: The removal of a lender's eligibility for a specified period of time or until the lender meets certain requirements.

Termination: The removal of a lender's eligibility for an indefinite period of time.

(20 U.S.C. 1078-2, 1080, 1082, 1094)

§ 683.102 Effect on prior participation.

Limitation, suspension, or termination proceedings do not affect a lender's responsibilities, or rights to benefits and claim payments, that are based on the lender's prior participation in the program, except as provided in § 683.109.

(20 U.S.C. 1078-2, 1080, 1082, 1094)

§ 683.103 Informal compliance procedure.

(a) If the Secretary receives a complaint, or other information that the Secretary believes to be reliable, indicating that a lender may be violating applicable laws, regulations, special arrangements, agreements, or limitations, the Secretary may give the lender a reasonable opportunity to—

(1) Respond to the complaint or other information;

(2) Show that the matter has been corrected; or

(3) Submit an acceptable plan to correct the violation and prevent its recurrence.

(b) Limitation, suspension or termination procedures need not be delayed during the informal compliance procedure under paragraph (a) if the Secretary believes—

(1) The delay would harm the Federal PLUS program; or

(2) The informal compliance procedure would not correct the alleged violation.

(20 U.S.C. 1078-2, 1080, 1082, 1094)

§683.104 Emergency action.

(a) The Secretary, through a designated ED official, may take emergency action to stop issuing insurance commitments to a lender if the designated ED official—

(1) Receives information, which the official believes to be reliable, that the lender is violating applicable laws, regulations, special arrangements, agreements, or limitations;

(2) Determines that immediate action is necessary to prevent the likelihood of substantial losses by the Federal Government, students or parents; and

(3) Determines that the likelihood of loss outweighs the importance of following the procedures for limitation, suspension, or termination.

(b) The designated ED official begins an emergency action by notifying the lender, by certified mail with return receipt requested, of the action and the reasons for it. The effective date of the action is the date that the notice is mailed.

(c) An emergency action does not exceed 30 days unless a limitation, suspension, or termination proceeding is begun before that period expires. In that event, the emergency action may be extended until the completion of the proceeding, including any appeal that may be made to the Secretary.

(d) If a limitation, suspension, or termination proceeding is begun, the Secretary provides the lender, upon request, an opportunity to demonstrate that the emergency action is unwarranted.

(20 U.S.C. 1078-2, 1080, 1082, 1094)

§ 683.105 Suspension proceedings.

(a) *Scope and consequences.* A suspension removes a lender's eligibility under the Federal PLUS program for a period of time. That period does not exceed 60 days from the effective date of the suspension unless—

(1) The lender and the designated ED official agree to an extension, if the lender has not requested a hearing; or

(2) The designated ED official begins a limitation or termination proceeding.

(b) *Procedure.* (1) The designated ED official begins a suspension proceeding by sending a notice to the lender by certified mail with return receipt requested. In the notice, the designated ED official—

(i) Informs the lender of the Secretary's intent to suspend the lender's eligibility, cites the consequences of that action, and identifies the alleged violations on which that action is based;

(ii) Specifies the proposed effective date of the suspension, which is at least 20 days after the date of mailing of the notice of intent;

(iii) Informs the lender that the suspension will not take effect on the date specified in the notice if the designated ED official receives, at least 5 days before that date, a request for a hearing or written material showing why the suspension should not take place; and

(iv) Asks the lender to correct voluntarily the alleged violation(s).

(2) If the lender does not request a hearing but submits written material the designated ED official considers that material and notifies the lender that—

(i) The proposed suspension is dismissed; or

(ii) The suspension is effective as of a specified date.

(3) If the lender requests a hearing within the time specified in paragraph (b)(1)(iii) of this section, the designated ED official sets the date and place. The date is at least 15 days after the designated ED official receives the request. No suspension takes place until a hearing is held.

(4) A presiding officer conducts the hearing and a written record of the hearing is made.

(5) At the hearing, the presiding officer shall consider any written material presented before the hearing and all other evidence presented during the hearing.

(6) If the presiding officer concludes that the suspension is warranted, the presiding officer issues an initial decision suspending the lender's eligibility.

(7) The Secretary reviews the initial decision of the presiding officer and issues a final decision. The Secretary adopts the initial decision unless it is clearly unsupported by the evidence.

(c) Notice of the suspension is promptly mailed to the lender. The suspension takes effect either on the date that the initial decision notice is mailed to the lender or on the original proposed effective date stated in the notice of intent, whichever is later.

(d) If the designated ED official begins a limitation or termination proceeding before the suspension period ends, the suspension period may be extended until the completion of that proceeding, including any appeal to the Secretary.

(20 U.S.C. 1078-2, 1080, 1082, 1094)

§ 683.106 Limitation or termination proceedings.

(a) *Scope and consequences.* A limitation or termination either—

(1) Limits in a specified manner the eligibility of a lender to participate in the Federal PLUS program; or

(2) Removes the eligibility of a lender to make any new Federal PLUS loans.

(b) *Procedure.* (1) The designated ED official begins a limitation or

termination proceeding, whether or not a suspension proceeding has begun, by sending the lender a notice by certified mail with return receipt requested. In the notice, the designated ED official—

(i) Informs the lender of the Secretary's intent to limit or terminate the lender's eligibility, cites the consequences of that action, identifies the alleged violations on which that action is based, and in the case of a limitation states the limits which may be imposed;

(ii) Specifies the proposed effective date of the limitation or termination, which is at least 20 days after the date of mailing of the notice of intent;

(iii) Informs the lender that the limitation or termination will not take effect on the date specified in the notice if the designated ED official receives, at least 5 days before that date, a request for a hearing or written material showing why the limitation or termination should not take place; and

(iv) Asks the lender to correct voluntarily the alleged violation(s).

(2) If the lender does not request a hearing but submits written material, the ED official considers that material and notifies the lender that either—

(i) The proposed action is dismissed;

(ii) Limitations are effective as of a specified date; or

(iii) The termination is effective as of a specified date.

(3) If the lender requests a hearing within the time specified in paragraph (b)(1)(iii) of this section, the designated ED official sets the date and place. The date is at least 15 days after the designated ED official receives the request. No proposed limitation or termination takes place until after a hearing is held.

(4) A presiding officer conducts the hearing, and a written record of the hearing is made.

(5) At the hearing the presiding officer shall consider any written material presented before the hearing and all other evidence presented during the hearing.

(6) If the presiding officer concludes that limitation or termination is warranted, the presiding officer issues an initial decision that limits or terminates the lender's eligibility.

(7) If a termination action is brought against a lender, and the presiding officer believes a limitation to be more appropriate, the presiding officer may issue a decision imposing one or more limitations on a lender rather than terminating its eligibility.

(c) *Expedited hearings.* With the approval of the presiding officer and the consent of the designated ED official and the lender, any time schedule specified in this section may be shortened.

(20 U.S.C. 1078–2, 1080, 1082, 1094)

§ 683.107 Initial and final decisions.

(a) The presiding officer issues an initial decision in any limitation, suspension, or termination proceeding based on findings of fact and conclusions of law. The presiding officer shall base findings of fact only on evidence considered at the hearing and matters given official notice. The presiding officer's initial decision is mailed promptly to the lender.

(b) In a suspension proceeding, the Secretary reviews the presiding officer's initial decision and issues a final decision. The Secretary adopts the initial decision unless it is clearly unsupported by the evidence.

(c)(1) In a limitation or termination proceeding, the presiding officer's initial decision automatically becomes the Secretary's final decision 20 days after it is issued unless, within that 20-day period, the lender or designated ED official appeals the decision to the Secretary.

(2) Within a period of time specified by the Secretary the appealing party may submit additional written material including exceptions to the initial decision, proposed findings and conclusions, and supporting briefs and statements. The Secretary sets a time by which the opposing party shall respond. Any party submitting material to the Secretary shall provide a copy to each party that participated in the hearing.

(3) The presiding officer's initial decision limiting or terminating the lender's eligibility does not take effect pending the appeal, unless the Secretary determines that a stay of the effective date would seriously and adversely affect the Federal PLUS program, students or parents.

(4) After an appeal the Secretary issues a final decision affirming, modifying, or reversing the initial decision, including a statement of reasons for the Secretary's decision.

(20 U.S.C. 1078–2, 1080, 1082, 1094)

§ 683.108 Verification of mailing dates.

The Department of Education's mailing dates are verified by the original receipts from the United States Postal Service.

(20 U.S.C. 1078–2, 1080, 1082, 1094)

§ 683.109 Effect of suspension or termination proceeding.

After the effective date of a lender's suspension or termination, the Secretary does not insure new loans made by that lender. Also, the Secretary may prohibit the lender from making further disbursements on a loan for which an insurance commitment already has been issued.

(20 U.S.C. 1078–2, 1080, 1082, 1094)

§ 683.110 Limitation.

A limitation may include—

(a) A limit on the number or total amount of Federal PLUS loans that a lender may make, purchase, or hold;

(b) A limit on the number or total amount of Federal PLUS loans a lender may make to students or parents on behalf of students at a particular school; and

(c) Other reasonable requirements or conditions.

(20 U.S.C. 1078–2, 1080, 1082, 1094)

§ 683.111 Reimbursements, refunds, and offsets.

(a) The Secretary, designated ED official, or presiding officer may require a lender to take reasonable corrective action to remedy a violation of applicable laws, regulations, special arrangements, agreements, or limitations.

(b) The corrective action may include payment to the Secretary or to designated recipients of any funds that the lender improperly received, withheld, disbursed, or caused to be disbursed. Corrective action may, for example, relate to—

(1) Special allowance and other claims paid by the Secretary; or

(2) Required refunds to students who receive or on whose behalf parents receive PLUS loans, in the case of a school lender.

(c) If a final decision requires a lender to reimburse or make any payment to the Secretary, the Secretary may offset these claims against any benefits or claims due the lender.

(20 U.S.C. 1078–2, 1080, 1082, 1094)

§ 683.112 Reinstatement after termination.

(a) A lender whose eligibility has been terminated may file a request for reinstatement of its eligibility. This request may not, however, be filed within 18 months of the effective date of the termination.

(b) The reinstatement request must be in writing and must show that the lender has corrected the violation(s) on which its termination was based and meets all qualifications for eligibility.

(c) A school lender whose eligibility as a participating school has been terminated under 34 CFR Part 668 may not be reinstated as a Federal PLUS program lender until it is reinstated as a participating school. However, the school may request reinstatement as

both a school and a lender at the same time.

(d) The Secretary, within 60 days of receiving the reinstatement request either—

(1) Grants the request;

(2) Denies the request; or

(3) Grants the request subject to limitations.

(e)(1) If the Secretary denies the request or establishes other limitations, the lender, upon request, will be granted an opportunity, including a meeting, to show why its eligibility should be fully reinstated.

(2) A lender that is reinstated with limitations may participate in the Federal PLUS program under the limitations pending this appeal.

(20 U.S.C. 1078-2, 1080, 1082, 1094)

§ 683.113 Removal of limitation.

(a) A lender may request removal of the Secretary's limitation imposed under these regulations no sooner than 12 months after the effective date of the limitation.

(b) The request must be in writing and show that the lender has corrected the violation(s) on which the limitation was based.

(c) The Secretary within 60 days of receiving the request, either—

(1) Grants the request;

(2) Denies the request; or

(3) Grants the request subject to other limitations.

(d) If the Secretary denies the request or establishes other limitations, the lender, upon request, will be granted an opportunity, including a meeting, to show why its eligibility should be fully reinstated.

(e) The lender may participate in the Federal PLUS program under the limitations pending this appeal.

(20 U.S.C. 1078-2, 1080, 1082, 1094)

Appendix A—Standards for Acceptable Refund Policies by Participating Schools

For purposes of § 683.87(b) the Secretary considers guidelines VI, VII, and VIII of the following document to be acceptable elements of a fair and equitable school refund policy. The document, which is reproduced in its entirety for the convenience of the reader, was developed by the National Association of College and University Business Officers. The document does not affect a school's obligation to comply with other Department of Education regulations.

Policy Guidelines for Refund of Student Charges

(I) *The governing board of the institution should review and approve the schedule of all institutional charges and refund policies applicable to students.* The pricing of services and refund policies have important consequences to students, parents, the institution, and society; as such, pricing and refund policies should receive board attention and approval.

(II) *Institutions should seek consumer views in the process of establishing and amending charge and refund structures.* Decisions regarding institutional funds are ultimately the sole responsibility of the institution's legally designated fund custodians. However, consumer concerns do affect decision-making, and involving consumers in decision-making related to charges and refunds is a desirable approach for assessing student needs and creating public awareness of institutional requirements.

(III) *Institutions should publish a current schedule of all student charges, a statement of the purpose for such charges, and related refund policies, and have them readily available free of charge to current and prospective students.* Students and parents have a right to know what charges they will be expected to pay and what will or will not be refunded. They also have a right to know what services accompany payment of the charges. Informational materials published free for students and prospective students are ideal for this purpose.

(IV) *Institutions should clearly designate all optional charges as "optional" in all published schedules and related materials. Clearly, charges that are mandatory and charges that are optional must be plainly differentiated in all printed materials.* Also, the institution should state clearly in its schedule if a charge is optional for some students but required for others. Statements accompanying the schedule may include institutional endorsements of the optional program or service.

(V) *Institutions should clearly identify charges and deposits that are nonrefundable as "nonrefundable" on all published schedules.* Institutions determine on an individual basis which of their charges are refundable or non-refundable. In general, admission fees, application fees, laboratory fees, facility and student activity fees, and other similar charges are not refundable. These fees are generally charged to cover the cost of activities such as processing applications and other student information, reserving academic positions, and establishing the limits of institutional programs and services, reserving housing space, and otherwise setting the fixed costs of the institution for the coming academic periods.

Institutions determine on an individual basis which of their deposits are refundable or nonrefundable. Some deposits will be nonrefundable or will be credited to a student's account (e.g., tuition deposits). Others are refundable according to the terms of the deposit agreement (e.g., deposits for breakage).

(VI) *Institutions should refund housing rental charges, less a deposit, so long as written notification of cancellation is made prior to a well-publicized date that provides reasonable opportunity to make the space available to other students.* Written notification on or before the beginning of the term of the contract is necessary to ensure utilization of housing units. During the term of the contract, room charges are generally not refundable. However, based on the program offered, space availability, debt service requirements, State and local laws, and other individual circumstances, institutions may provide for some more flexible refund guideline for housing.

(VII) *Institutions should refund board charges in full, less a deposit, if written notification of cancellation is made prior to a well-publicized date that falls on or before the beginning of the term of the contract. Subsequent board charges should be refunded on a pro rata basis less a withdrawal fee.* It is reasonable to make a refund for those goods and services not consumed. The withdrawal charge should reflect that portion of an institution's costs that are fixed for the term of the contract.

(VIII) *The institutional tuition refund policy for an academic period should include the following minimum guidelines:*

A. *The institution should refund 100 percent of the tuition charge, less a deposit fee, if written notification of cancellation is made prior to a well-publicized date that falls on or before the first day of classes.*

B. *The institution should refund at least 25 percent of the tuition charge if written notification of withdrawal is made during the first 25 percent of the academic period.* It is reasonable to refund tuition charges on a sliding scale if a student withdraws from his or her program prior to the end of the first 25 percent of the academic period unless State law imposes a more restrictive refund policy.

(IX) *The institution should assess no penalty charges where the institution, as opposed to the student, is in error. The institution should make refunds in cases where the institution has assessed charges in error.* Penalty charges, such as those involving late registration fees,

change of schedule fees, late payment fees, should not be assessed if it is determined that the student is not responsible for the action causing the charge to be levied.

(X) *Institutions should advise students that any notifications of withdrawal or cancellation and requests for refund must be in writing and addressed to the designated institutional officer.* A student's written notification of withdrawal or cancellation and request for a refund provides an accurate record of transactions and also ensures that such request will be processed on a timely basis. Acceptance of oral requests is an undesirable practice.

(XI) *Institutions should pay or credit refunds due on a timely basis.* The definition of "timely basis" should include the time required to process a formal student request for refund, to process a check if required, and to allow for mail delivery, when necessary. If an institution has a policy that a refund of an inconsequential amount will not be made, this policy should be published in part of all materials related to refund policies.

(XII) *Institutions should publicize, as a part of their dissemination of information on charges and refunds, that an appeals process exists for students or parents who feel that individual circumstances warrant exceptions from published policy. The informational materials should include the name, title, and address of the official responsible.* Although charges and refund policies should reflect extensive consideration of student and institutional needs, it will not be possible to encompass in these structures the variety of personal circumstances that may exist or develop. Institutions are required to provide a system of due process to their students, and charges and refund policies are legitimately a part of that process. Students and parents should be informed regularly of procedures for requesting information concerning exceptions to published policies.

[FR. Doc. 81-10693 Filed 4-20-82; 8:45 am]
BILLING CODE 4000-01-M

federal register

Wednesday
April 21, 1982

Part V

Department of the Interior

Office of Surface Mining Reclamation and Enforcement

Surface Mining and Reclamation Operations Under a Federal Program for Georgia

DEPARTMENT OF THE INTERIOR

Office of Surface Mining Reclamation and Enforcement

30 CFR Part 910

Surface Mining and Reclamation Operations Under a Federal Program for Georgia

AGENCY: Office of Surface Mining Reclamation and Enforcement, Interior.

ACTION: Proposed rule.

SUMMARY: The Office of Surface Mining Reclamation and Enforcement (OSM) proposes to revise the final regulations containing the Federal program for the regulation by the Secretary of the Interior of coal exploration and surface coal mining and reclamation operations including the surface effects of underground coal mining on non-Federal and non-Indian lands within the State of Georgia (47 FR 10372; March 10, 1982). The proposal is necessary to reflect changes being made to the permanent program regulations on which the Georgia Federal Program is based.

DATES: Written comments must be received on or before June 23, 1982, not later than 5:00 p.m. EST. Requests to testify at the public hearing must be received by June 11, 1982. Hearing date: 9:00 EST on June 18, 1982.

ADDRESSES: Written comments to OSM Alabama State Office, 228 W. Valley Avenue, Homewood, Alabama 35209. Site of hearing: Office of Regional Solicitor, U.S. Interior Department, Russell Federal Bldg. 75 Spring St. S.W., Suite 1328, Atlanta, Georgia 30303. All written comments received, transcripts of any public hearings, summaries of any meetings, and other documents making up the administrative record will be available for public inspection during regular business hours at both the OSM Alabama State Office and the Regional Solicitor's Office.

FOR FURTHER INFORMATION CONTACT: Mr. John T. Davis, State Director, 228 W. Valley Avenue, Homewood, Alabama 35209 (205-254-0890).

SUPPLEMENTARY INFORMATION:

Public Participation

OSM appreciates any and all comments on this proposal, but those most useful will be as specific as possible, focus on the issues of this proposed rulemaking, and provide reasons for any recommendations. OSM will not consider comments that do not pertain to the issues in this proposal. Nor can OSM asure consideration of comments delivered after the comment period ends or those delivered to an address other than that specified.

Public Comment Period

The comment period on the proposed revision will extend until June 23, 1982. All written comments must be received at the location above under "ADDRESSES" by 5:00 p.m. on that date.

Public Hearing

A public hearing on these proposed rules will be held on June 18, 1982. Persons wishing to testify at the public hearing on this proposed regulation should contact the person listed under "FOR FURTHER INFORMATION CONTACT" on or before June 11, 1982. If no persons indicate an intention to testify by June 11, 1982, the hearing may be cancelled. In the event of cancellation, anyone may submit written comments and meet personally with OSM representatives between June 14, 1982 and June 23, 1982.

Individual testimony at the hearing will be limited to 15 minutes. The hearing will be transcribed. Filing of a written statement at the time of giving oral testimony would be helpful and would facilitate the job of the court reporter. Submission of written statements in advance of the hearing would greatly assist OSM officials who will attend the hearing.

Advance submissions will give these officials an opportunity to consider appropriate questions which could be asked for clarification or to request more specific information from the person testifying. The public hearing will continue on the day identified above until all persons scheduled to speak have been heard. Persons in the audience who have not been scheduled to speak and wish to do so will be heard following the scheduled speakers. The hearing will end after all persons scheduled to testify and persons present in the audience who wish to speak have been heard. Persons not scheduled to testify, but wishing to do so, assume the risk of having the public hearing adjourned unless they are present in the audience at the time all scheduled speakers have been heard.

Proposed Program Revision

On March 10, 1982 a final rule for a Federal program under the Surface Mining Control and Reclamation Act (the "Act"), Pub. L. 95-87, 30 U.S.C. 1201 *et. seq.*, was published for the State of Georgia to become effective immediately. The program for Georgia was promulgated largely unchanged from the proposal in order to meet the exigencies caused by the unregulated surface mining of coal in Georgia. *See* 45 FR 61120 (September 15, 1980) for the proposed Federal program. Revision of the program is being proposed in order to reflect changes which are under consideration for the permanent program regulations on which the Georgia Federal program is based.

Pursuant to 30 CFR 736.22, a Federal program for a State is to include, at a minimum, the provisions in 30 CFR Parts 700 and 701 which contain general provisions and definitions, Part 707 on the exemption from regulation for government financed construction, Parts 760, 761, 762 and 765 on designation of lands unsuitable for surface mining, Subchapter G on permit applications, Subchapter J on bonding, Subchapter K on performance standards, Parts 842 and 843 on inspection and enforcement, and Subchapter M on blaster training and certification. The Georgia program was the first Federal Program implemented. It included the just-cited Parts and Subchapters by adopting them virtually verbatim. Some changes were made in order to give effect to section 505(b) of the Act which provides that State laws which establish more stringent land use and environmental protection provisions shall not be deemed to be inconsistent with the Act and regulations. Some additions were also made to set time periods not specified in the permanent program regulations.

In January 1981 the Secretary directed that the Department review all regulations with a goal of eliminating those which are burdensome, excessive and unnecessary. That effort is resulting in a large scale revision of the national permanent program regulations on which the current Georgia Federal program is based. *See* semi-annual Calendar of Federal Regulations notice on OSM regulations under review, 47 FR 1709 (January 13, 1982). Also *see*, e.g., proposed revision of the bonding regulations, 30 CFR Subchapter J, at 46 FR 45082 (September 9, 1981), and inspection and enforcement regulations, 30 CFR Subchapter L, at 46 FR 58464 (December 1, 1981).

Over time, all of the premanent program regulations will undergo review. Many will be revised. In order to take advantage of the results which revision of the permanent program regulations will achieve, OSM proposes to modify the Federal program in the following manner.

Rather than repeating in the Georgia Federal program the full text of the permanent program regulations being revised, there would be a cross-reference to the permanent program regulations. For example, for criteria for the designation of lands unsuitable for

surface coal mining, (30 CFR Part 910, Subpart 762, in the current Georgia Federal program), the substance of the rules now found in Subparts 910.762-4—910.762-14 would be modified to state merely that "the Secretary may designate lands unsuitable * * * pursuant to the criteria in 30 CFR Part 762." The effect of this proposed cross-referencing to the permanent program regulations, insofar as revision of the latter rules is concerned, would be that as the permanent program regulations are revised the Georgia Federal program would be similarly revised. No separate rulemaking would be undertaken for revision of the Georgia Federal program if the cross-referencing alternative becomes effective. A statement would appear in both the proposed and final permanent program rulemaking notices advising the public that the change in the permanent program rule would also result in a change in the Georgia Federal program. The statement for the permanent program proposed rule will invite comments on necessary modifications to accommodate unique or unusual aspects of surface mining in Georgia. The final rule for Georgia will be tailored for the State as necessary.

If this proposed rule becomes effective, the current Georgia Federal program automatically would be revised section-by-section as each section of the Federal permanent program rules is revised. However, there would be no modification of the substance of the current Federal program by promulgation of a cross-referencing program. Few changes were made in the permanent program rules as they were adopted for the current full text Federal program. For the changes made in the permanent program rules under the current program, see the proposed rulemaking notice, 46 FR at 61127-31. In order not to change the substance of the current program through cross-referencing, a separate statement is proposed to be added under each section which indicates the changes made in the permanent program regulation for the Georgia Federal program. These statements will generally be found in subsection (b) of the section in which changes have been made.

This same approach of cross-referencing the permanent program rules may be used in the promulgation of other Federal programs. Proposed Federal coal exploration program rules published for the States of Massachusetts, Rhode Island, Michigan and Oregon, use some cross-referencing. 47 FR 560 (January 5, 1982). Public comment on the cross-referencing method as it affects other Federal programs, however, should be directed to those particular notices.

In order to tailor the Georgia Federal program to the particular conditions in the State, as required by section 504(a) of the Act, OSM reviewed relevant State statutes and regulations and identified those which set more stringent standards than those set by the permanent program regulations. *See* 45 FR at 61127-61131. According to section 505(a) of the Act, these more stringent State standards shall not be deemed to be inconsistent with Federal law and regulations. Also, section 504(g) of the Act requires the Secretary to identify any State laws which interfere with achievement of the purposes and requirements of the Act and regulations. The Georgia Surface Mining Act and implementing regulations were identified as interfering with achievement of the Federal program. 45 FR at 61125. The State statutes and regulations which either interfere with achievement of the program or set more stringent standards are identified in § 910.3 of the proposed revision of the Georgia program set out below.

The Act in section 504(h) also requires that a Federal program include a process for coordinating the review and issuance of permits for surface mining and reclamation operations with any other Federal or state permit process applicable to the proposed operation. This requirement was fulfilled in the current program by indicating in § 910.770-12 that, in addition to enumerated Federal statutes and regulations, ten State statutes administered by the Georgia Department of Natural Resources and one rule administered by the Georgia Fire Safety Commission require coordination. These same statutes and rules are proposed to be listed in § 910.770 for the cross-referencing Federal program. Public comment is invited on this method of effectuating these requirements of sections 504 (a) and (g) and 505 of the Act.

Several provisions of the permanent program regulations are already applicable to the Georgia Federal program because they were fully promulgated for application to all regulatory programs. Those provisions are 30 CFR Chapter VII, Subchapter P—Protection of Employees, Part 706—Restrictions on Financial Interests of Federal Employees, and Part 769—Petition Process for Designation of Federal Lands Unsuitable for Surface Coal Mining. They need not be separately cross-referenced for the Georgia Federal program. In addition, 30 CFR Part 765—Designating Lands Unsuitable for Surface Coal Mining under a Federal Program will not be included in the cross-referencing revision. The current program adopts 30 CFR Part 764—Designating Lands Unsuitable Under a State Program with appropriate changes. Changes which reflect that a Federal program is involved are noted under § 910.764 for the cross-referencing program.

Throughout the permanent program regulations reference is made to the regulatory authority. Under section 701(22) of the Act and 30 CFR 700.5, the Secretary of the Interior is the regulatory authority for a Federal program. Thus, no change is proposed to state in the cross-referencing Georgia program that the Secretary is the regulatory authority. However, the current program uses the term "Office" and "Director" in lieu of "regulatory authority." The Office of Surface Mining, of which the Director is the head, is delegated all of the Secretary's authority for implementing, maintaining and enforcing a Federal program. No change in the officials charged with carrying out the program would occur by promulgation of the cross-referencing program.

The current numbering system for the cross-referencing program is proposed to be retained. It correlates with the related part of the permanent program regulations. However, the designation "subpart" is proposed to be changed to "section" since the number "910" before each is sufficient to indicate that reference is being made only to the Georgia Federal program.

With regard to bonding regulations (Subchapter J), only Part 800 is proposed to be cross-referenced because OSM has proposed to revise Subchapter J to include just one part, Part 800. 46 FR 45082 (September 9, 1981) (proposed).

Determinations Under Executive Order 12291, the Regulatory Flexibility Act and the National Environmental Policy Act. OSM has examined these proposed rules according to the criteria of Executive Order 12291 (46 13193, February 19, 1981) and determined that they do not constitute a major rule. There would be no economic impact through adoption of cross-referencing because no substantive revision of regulations is involved as a result of the rulemaking. However, economic effects will occur as a result of revision of the permanent program regulations. Separate determinations will be made for each revision of the permanent program regulations; these would include the economic effects in Georgia

if the cross-referencing program is promulgated.

This proposed rulemaking has been examined pursuant to the Regulatory Flexibility Act, 5 U.S.C. 601 *et seq.*, and OSM has determined that the proposed revision of the rules will not have a significant impact on a substantial number of small entities. Separate determinations of effect will be prepared for all revisions of the permanent program rules and would consider the effects on small entities in the State of Georgia.

Section 702(d) of the Act provides that promulgation of a Federal program shall not constitute a major Federal action under the National Environmental Policy Act, 42 U.S.C. 4332. Thus, no EA is required for this rulemaking.

Information Collection and Recordkeeping Requirements

The recordkeeping and reporting requirements of the proposed rule are the same as the national permanent program regulations which have been approved by the Office of Management and Budget under 44 U.S.C. 3507 and assigned clearance numbers as shown in the table below.

OMB Clearance Numbers

Part	Clearance No.
700	1029-0020
701	NA
761	1029-0029
762	NA
764	1029-0030
770	NA
776	1029-0033
787	NA
800	1029-0043
805	1029-0044
806	1029-0045
809	NA
815	NA
840	1029-0051
843	1029-0052
845	1029-0053

Although this rule would contain information and recordkeeping requirements, we anticipate less than ten respondents. Under the Paperwork Reduction Act and the Federal Reports Act, clearance of information collection forms is required only if ten or more respondents are expected. If in the future the number of respondents appears to be increasing, the proper forms will be submitted to the Office of Management and Budget with accompanying notices in the **Federal Register**, in accordance with the requirements of 44 U.S.C. Chapter 35.

List of Subjects in 30 CFR Part 910

Coal mining, Surface mining, Underground mining.

Accordingly 30 CFR Part 910 is proposed to be amended by removing the existing 30 CFR Part 910 and replacing it with the proposed 30 CFR Part 910 as set forth herein.

D. N. Miller, Jr.,
Assistant Secretary, Energy & Minerals.
March 31, 1982.

PART 910—GEORGIA

Sec.
910.1 Scope.
910.2 Applicability.
910.3 Supersession by more stringent laws and regulations.
910.701 General program provisions.
910.707 Exemption for coal extraction incident ot government-financed highway or other construction.
910.761 Areas designated unsuitable for surface coal mining by act of Congress.
910.762 Criteria for designating areas as unsuitable for surface coal mining operations.
910.764 Process for designating areas unsuitable for surface coal mining operations.
910.770 General requirements for permit and exploration procedures.
910.771 General requirements for permits and permit applications.
910.776 General requirements for coal exploration.
910.778 Surface mining permit applications—minimum requirements for legal, financial, compliance, and related information.
910.779 Surface mining permit applications—minimum requirements for information on environmental resources.
910.780 Surface mining permit applications—minimum requirements for reclamation and operations plan.
910.782 Underground mining permit applications—minimum requirements for legal, financial, compliance, and related information.
910.783 Underground mining permit applications—minimum requirements for information and environmental resources.
910.784 Underground mining permit applications—minimum requirements for reclamation and operation plan.
910.785 Requirements for permits for special categories of mining.
910.786 Review, public participation, and approval or disapproval of permit terms and conditions.
910.787 Administrative and judicial review of decisions on permit applications.
910.788 Permit reviews, revisions, and renewals, and transfer, sale, and assignment of rights granted under permits.
910.795 Small operator assistance.
910.800 Requirements for bonding of surface coal mining and reclamation operations.
910.815 Performance standards—coal exploration.
910.816 Performance standards—surface mining activities.
910.817 Performance standards—underground mining activities.
Sec.
910.818 Special performance standards—concurrent surface and underground mining.
910.819 Special performance standards—auger mining.
910.823 Special performance standards—operations on prime farmland.
910.824 Special performance standards—mountaintop removal.
910.826 Special performance standards—operations on steep slopes.
910.827 Special performance standards—coal processing plants and support facilities not located at or near the minesite or not within the permit area for a mine.
910.828 Special performance standards—in situ processing.
910.842 Federal inspections.
910.843 Federal enforcement.
910.845 Civil penalties.

Authority: Pub. L. 95-87, Surface Coal Mining Reclamation Act of 1977 (30 U.S.C. 201 et seq.)

§ 910.1 Scope.

This part contains all rules applicable to surface coal mining operations in Georgia that have been adopted under the Surface Mining Control and Reclamation Act of 1977.

§ 910.2 Applicability.

The rules in Part 910 apply to all surface coal mining operations in Georgia conducted on non-Federal and non-Indian lands. The rules in 30 CFR Subchapter D apply to operations on Federal lands in Georgia. The rules in Part 910 cross-reference pertinent parts of the permanent program regulations in 30 CFR Chapter VII. For the text of the regulations, reference must be made to the permanent program rule cited under the relevant section of the Georgia Federal program.

§ 910.3 Supersession by more stringent laws and regulations.

(a) The following provisions of Georgia laws and regulations provide for more stringent environmental control and regulation of surface coal mining operations than do the provisions of the Surface Mining Control and Reclamation Act and the regulations in 30 CFR Chapter VII. Therefore, pursuant to section 505(b) of the Act, they shall not be construed to be inconsistent with the Act:

(1) Georgia Code Ann. Section 56-412 pertaining to limitation of risks for insurance companies.

(2) Georgia Code Ann. Section 414-1306 pertaining to the limitation on loan amounts made to banks.

(3) Georgia Safe Dams Act of 1978 and the Rules for Dam Safety, Chapter 391-3-8 of the Rules of the Georgia Department of Natural Resources, Environmental Protection Division.

(4) Chapter 391-34 of the rules of the Department of Natural Resources, Environmental Protection Division, pertaining to solid waste management.

(5) Georgia Seed Laws and Rules and Regulations containing the Noxious Weed List.

(b) The following are Georgia laws and regulations that interfere with the purposes and requirements of the Surface Mining Control and Reclamation Act and which are, in accordance with Section 504(g), preempted and superseded:

(1) The Georgia Surface Mining Act of 1968, Ga. Code Anno. Section 43-1401 *et seq.*, but not to the extent that it regulates surface coal mining operations which affect two acres or less or are otherwise not regulated by the Surface Mining Control and Reclamation Act.

(2) Rules for Land Reclamation, Georgia Department of Natural Resources, Environmental Protection Division, Chapt. 391-3-3 (1976), but not to the extent that such regulations apply to surface coal mining operations which affect two acres or less or are otherwise not regulated by the Surface Mining Control and Reclamation Act.

§ 910.701 General program provisions.

The general requirements for the Georgia Federal program shall be the requirements set forth in 30 CFR 700.5, 700.11, 700.12, 700.13, 700.14, 700.15, and Part 701 and shall apply to surface coal mining operations in Georgia located on non-Indian and non-Federal lands, except that the definition of "public road" in Georgia Code Ann. Section 95A-104 shall be used in lieu of the definition of "road" in § 701.5.

§ 910.707 Exemption for coal extraction incident to government-financed highway or other construction.

The provisions of this Part do not apply to any operations which meet the criteria for the exemption in 30 CFR Part 707.

§ 910.761 Areas designated unsuitable for surface coal mining by act of congress.

The Secretary may prohibit or limit surface coal mining operations on or near certain private Federal and other public lands pursuant to 30 CFR Part 761 beginning one year after the effective date of the program. No person shall conduct surface mining operations in violation of any such prohibition or limitation.

§ 910.762 Criteria for designating areas as unsuitable for surface coal mining operations.

Beginning one year after ———, 1982 [insert effective date of the program], the Secretarty may designate lands unsuitable for surface coal mining pursuant to the criteria in 30 CFR Part 762.

§ 910.764 Process for designating areas unsuitable for surface coal mining operations.

Beginning one year after ———, 1982 [insert effective date of the program], the Secretary may designate areas unsuitable for surface coal mining pursuant to those procedures in 30 CFR Part 764 pertaining to petitioning, initial processing, hearing requirements, decision, data base and inventory system, public information, and regulatory responsibilities.

§ 910.770 General requirements for permit and exploration procedures.

No person shall conduct coal exploration which results in the removal of more than 250 tons of coal or shall conduct surface coal mining operations without a permit issued by the Secretary pursuant to 30 CFR Part 770 and permits issued pursuant to the Georgia Water Quality Control Act (section 17-501); Georgia Solid Waste Management Act (section 43-1661) (permit required from Georgia Department of Natural Resources); Georgia Air Quality Act of 1973; Georgia Safe Dams Act of 1973; Georgia Hazardous Waste Management Act of 1979; Georgia Groundwater Use Act; and Rules of the Georgia Fire Safety Commission (blaster permit). The permit issued by the Secretary shall incorporate the requirements of the Georgia Wildflower Preservation Act of 1973 (section 43-1801 *et seq.*); the Georgia Endangered Wildlife Act of 1973 (section 43-2101 *et seq.*); the Georgia Heritage Trust Act of 1975 (section 43-2301 *et seq.*); and the Georgia Cave Protection Act of 1977 (section 43-2501 *et seq.*). The Secretary shall coordinate review and issuance of a coal exploration or surface coal mining permit with the review and issuance of other Federal and State permits listed in this section and 30 CFR Part 770.

§ 910.771 General requirements for permits and permit applications.

(a) The general requirements for permits and permit applications are set forth in 30 CFR Part 771.

(b) In addition to the requirements in Part 771, a person who wishes to conduct new surface coal mining and reclamation operations or who wishes to revise a permit shall file a complete application at least 12 months prior to the date upon which permit issuance is desired, and shall pay to the Secretary a permit fee in accordance with 30 CFR 736.25.

§ 910.776 General requirements for coal exploration.

(a) Coal exploration shall be conducted in compliance with 30 CFR Part 776.

(b) In addition to the requirements of Part 776, within one week after the determination is made that an application is complete the applicant shall post notice of an application for approval of exploration at the county courthouse of the county in which exploration is to occur. Any person with an interest which is or may be adversely affected shall have the right to file written comments with the Secretary within 15 days of posting of the notice.

(c) The Office shall make every effort to act on an exploration application within 60 days of receipt.

§ 910.778 Surface mining permit applications—minimum requirements for legal, financial, compliance, and related information.

The applicant for a surface coal mining permit shall submit legal, financial, compliance, and related information as required in 30 CFR Part 778.

§ 910.779 Surface mining permit applications—minimum requirements for information on environmental resources.

The applicant for a surface mining permit shall submit environmental resources information in compliance with 30 CFR Part 779.

§ 910.780 Surface mining permit applications—minimum requirements for reclamation and operation plan

(a) The applicant for a surface coal mining permit shall submit a reclamation and operation plan as required in 30 CFR Part 780.

(b) In addition to the requirements of Part 780, the application for a permit shall demonstrate compliance with the air quality control standards in Chapter 391-B-1 of the Rules and Regulations for Air Quality Control of the Georgia Department of Natural Resources.

§ 910.782 Underground mining permit applications—minimum requirements for legal, financial, compliance, and related information.

The applicant for an underground coal mining permit shall submit legal, financial, compliance and related information as required in 30 CFR Part 782.

§ 910.783 Underground mining permit applications—minimum requirements for information on environmental resources.

The applicant for an underground coal mining permit shall submit information

on environmental resources as required in 30 CFR Part 783.

§ 910.784 Underground mining permit applications—minimum requirements for reclamation and operation plan.

(a) The applicant for an underground coal mining permit shall submit a reclamation and operation plan pursuant to the terms of 30 CFR Part 784.

(b) In addition to the requirements of Part 784, the application shall demonstrate specific compliance with Chapter 391-3-1, Rules and Regulations of the Georgia Department of Natural Resources.

§ 910.785 Requirements for permits for special categories of mining.

The applicant for a permit who plans to conduct operations which fall within special categories of mining shall submit additional information as required by 30 CFR Part 785.

§ 910.786 Review, public participation and approval or disapproval of permit terms and conditions.

(a) The Secretary shall review, and approve or disapprove permit terms and conditions for permit applications and shall provide for public participation pursuant to 30 CFR Part 786.

(b) In addition to the requirements of Part 786, the applicant shall place an advertisement in a local newspaper of general circulation concerning the permit after the Secretary determines the application to be complete. Written comments on the permit application shall be submitted to the Secretary within 30 days of receipt of the application. The Secretary shall provide an informal conference within 30 days of a request.

§ 910.787 Administrative and judicial review of decisions on permit applications.

Decisions on permit applications shall be subject to administrative and judicial review in accordance with 30 CFR Part 787 and sections 520, 525 and 526 of the Act.

§ 910.788 Permit reviews, revisions, and renewals, and transfers, sales, and assignments of rights granted under permits.

(a) Permit reviews, revisions and renewals, and transfers, sales and assignments of rights granted under permits shall be in accordance with 30 CFR Part 788.

(b) In addition to the requirements of Part 788, a person whose interests are or may be affected may file written comments within 30 days of placement of any advertisement announcing an application for permit transfer, assignment or sale.

§ 910.795 Small operator assistance.

Small operator assistance shall be provided pursuant to 30 CFR Part 795.

§ 910.800 Requirements for bonding of surface coal mining and reclamation operations.

(a) The operator shall bond surface coal mining and reclamation operations in compliance with 30 CFR Part 800.

(b) In addition to the requirements of Part 800, surety bonds shall be subject to Georgia Code Ann. Section 56-412 and letters of credit shall be subject to Georgia Code Ann. Sections 41A-1306 and 1309.

§ 910.815 Performance standards—coal exploration.

Coal exploration shall be conducted in compliance with 30 CFR Part 815.

§ 910.816 Performance standards—surface mining activities.

(a) The permittee shall conduct surface mining activities in compliance with the performance standards of 30 CFR Part 816.

(b) In addition to the requirements under Part 816, the operator shall comply with the Georgia Safe Dams Act and Rules for Dam Safety of the Department of Natural Resources as provided in 30 CFR 816.49(a), the Solid Waste Management Rules of the Georgia Department of Natural Resources, Environmental Protection Division, Chapter 391-3-4, as provided in 30 CFR 816.89(b) and the Georgia Seed Laws and Regulation 4 as provided in 30 CFR 816.112(d).

§ 910.817 Performance standards—underground mining activities.

(a) The permittee shall conduct underground mining activities in compliance with 30 CFR Part 817.

(b) In addition to the requirements under Part 817, the operator must comply with the Georgia Safe Dams Act and Rules for Dam Safety of the Department of Natural Resources, Environmental Protection Division, as provided in 30 CFR 817.49(a), the Georgia Department of Natural Resources, Environmental Protection Division, Solid Waste Management Rules, Chapter 391-3-4, as provided in 30 CFR 817.89(b) and the Georgia Seed Laws and Regulation 4 as provided in 30 CFR 817.112(d).

§ 910.818 Special performance standards—concurrent surface and underground mining.

The permittee shall conduct concurrent surface and underground mining operations in compliance with 30 CFR Part 818.

§ 910.819 Special performance standards—auger mining.

The permittee shall conduct auger mining in compliance with 30 CFR Part 819.

§ 910.823 Special performance standards—operations on prime farmland.

Operations on prime farmland shall be conducted in compliance with 30 CFR Part 823.

§ 910.824 Special performance standards—mountaintop removal.

Mountaintop removal shall be conducted in compliance with 30 CFR Part 824.

§ 910.826 Special performance standards—operations on steep slopes.

Operations on steep slopes shall be conducted in compliance with 30 CFR Part 826.

§ 910.827 Special performance standards—coal processing plants and support facilities not located at or near the minesite or not within the permit area for a mine.

Coal processing plants and support facilities not located at or near the minesite or not within the permit area for a mine shall be conducted in compliance with 30 CFR Part 827.

§ 910.828 Special performance standards—in situ processing.

In situ processing shall be conducted in compliance with 30 CFR Part 828.

§ 910.842 Federal inspections.

(a) Federal inspections shall be conducted in accordance with 30 CFR Part 842.

(b) In addition to the requirements of Part 842, the Secretary will furnish a copy of inspection reports or enforcement action documents to the Georgia Department of Natural Resources upon request.

§ 910.843 Federal enforcement.

(a) The Secretary shall take enforcement actions pursuant to 30 CFR Part 843.

(b) In addition to the requirements of Part 843, the Secretary will furnish a copy of any order to show cause to the Georgia Department of Natural Resources upon request.

§ 910.845 Civil penalties.

Civil penalties shall be assessed and paid and conferences and hearings shall be provided pursuant to 30 CFR Part 845.

[FR Doc. 82-10853 Filed 4-20-82; 8:45 am]
BILLING CODE 4310-05-M

federal register

Wednesday
April 21, 1982

Part VI

Department of Education

Office of Elementary and Secondary Education

Grants to State Educational Agencies To Improve the Interstate and Intrastate Coordination of Migrant Educational Activities

DEPARTMENT OF EDUCATION

Office of Elementary and Secondary Education

34 CFR Part 205

Grants to State Educational Agencies To Improve the Interstate and Intrastate Coordination of Migrant Education Activities

AGENCY: Office of Elementary and Secondary Education, ED.

ACTION: Final regulations.

SUMMARY: The Secretary issues final regulations for the Migrant Education Interstate and Intrastate Coordination Program authorized under Section 143 of Title I of the Elementary and Secondary Education Act of 1965 as amended (Title I). This program provides financial assistance to State educational agencies (SEAs) for projects designed to improve interstate and intrastate coordination of migrant education activities among SEAs, local educational agencies (LEAs), and other operating agencies—including those SEAs, LEAs, and other operating agencies participating in the Migrant Education Program authorized under Section 141 of Title I.

EFFECTIVE DATE: Unless the Congress takes certain adjournments, these regulations will take effect 45 days after publication in the **Federal Register.** If you want to know if there has been a change in the effective date of these regulations, call or write the Department of Education (ED) contact person. At a future date, the Secretary will publish a notice in the **Federal Register** stating the effective date of these regulations.

FOR FURTHER INFORMATION CONTACT: Mr. Vidal Rivera, Jr., Acting Director, Migrant Education Programs, Office of Elementary and Secondary Education, U.S. Department of Education, 400 Maryland Avenue SW. (Donohoe Bldg., Room 1100), Washington, D.C. 20202. Telephone No. (202) 245-2181.

SUPPLEMENTARY INFORMATION: The purpose of this program is to provide financial assistance to SEAs for special projects designed to improve interstate and intrastate coordination of migrant education activities among SEAs, LEAs, and other operating agencies—including those SEAs, LEAs, and other operating agencies participating in the Migrant Education Program authorized under Section 141 of Title I.

This program is specifically authorized by Section 143 of Title I as amended by the Education Amendments of 1978 (Pub. L. 95-561). In the Education Amendments of 1979 (Pub. L. 96-46), the Congress revised section 143 to require ED to "reserve * * * for any fiscal year * * * not * * * less than $6,000,000 nor more than 5 per centum of the amount * * * [appropriated for carrying out the Title I Migrant Education Program]." Accordingly, ED reserved the minimum amount of $6 million from the Title I Migrant Education Program's FY 1980 (school year 1980-1981) appropriation of $245 million. ED has also reserved $7.36 million from the FY 1981 (school year 1981-1982) appropriation of $266.4 million.

Section 143 of Title I authorizes the Secretary to make grants or contracts with SEAs for special projects designed to improve the interstate and intrastate coordination of migrant education activities. For a number of years, ED has contracted with an SEA to provide for the transfer of student records (the Migrant Student Record Transfer System (MSRTS)). Because of the nature of this particular interstate and intrastate coordination project, a contract is the most appropriate instrument to continue to procure the desired services and products. Since these regulations govern only grants under this program, the "transfer of student records" activity described in the authorizing program statute is not covered by these regulations.

These regulations are needed to—

(a) Provide guidance to SEAs in their administration of these projects; and

(b) Establish appropriate criteria for the Secretary's evaluation of applications.

Chapter 1 of the Education Consolidation and Improvement Act (Chapter 1) (Pub. L. 97-35), which was signed by the President on August 13, 1981, will supercede Title I with a simplified program to meet the special educational needs of educationally deprived children. The regulations for the Migrant Education Interstate and Intrastate Coordination Program will be reviewed as part of the simplification resulting from Chapter 1 and may be altered. However, any changes that are made as a result of that review will apply only to grants and contracts awarded under a Chapter 1 appropriation that would become available on July 1, 1982.

Nevertheless, the Secretary has decided to issue these final regulations to implement Section 143 of the Title I, as amended by the Education Amendments of 1978. By issuing these regulations, the Secretary establishes appropriate criteria for the selection of projects to be funded from the FY 1981 appropriation that became available on July 1, 1981, and provides guidance to SEAs in administering those projects.

Public Participation

The Secretary has taken a number of steps to maximize public participation in the development of these final regulations.

Two meetings were held with SEAs prior to the development of the proposed regulations for this program which were published in the **Federal Register** on October 22, 1981 (46 FR 51879). As a result of those meetings, the Secretary has developed some broad parameters for the types of projects that may be funded and the types of activities that may be conducted under those projects. The scope of these projects and activities was described in the proposed regulations.

In addition, prior to the development of the proposed regulations ED staff members had the opportunity, at a number of regularly-scheduled regional and national meetings of State directors of migrant education and other migrant education meetings involving other migrant education personnel and personnel from related agencies and organizations, to discuss all matters that need to be resolved and to solicit ideas, suggestions, and other advice.

In the October 22, 1981 notice of proposed regulations for this program, the Secretary established a 45-day period for the submission of written comments. Comments were encouraged particularly from SEA migrant education offices, State and local migrant education project personnel, education organizations with a particular interest in migrant education, migrant and seasonal farmworker advocacy organizations, and Hispanic advocacy organizations with a particular interest in migrant education. Furthermore, copies of the proposed regulations and accompanying information papers were mailed to these agencies and organizations.

The Secretary received six comments. Those comments and the Secretary's responses are summarized in the section of this preamble entitled "Summary of Comments and Responses."

During the comment period for the proposed regulations, ED staff members had a number of further opportunities—at regularly-scheduled national and regional meetings—to meet with State and local migrant education personnel and representatives of relevant agencies and organizations.

To assist ED in complying with the specific requirements of Executive Order 12291 and its overall requirement of reducing regulatory burden, public comment is invited on whether there may be further opportunities to reduce

any regulatory burden found in these regulations.

Summary of Comments and Responses

The following is a summary of the comments received on the proposed regulations for the Migrant Education Interstate and Intrastate Coordination Program. Each comment is followed by the Secretary's response.

Comment. One commenter recommended that the regulations be revised to include a specific mention of institutions of higher education (IHEs) with each reference to "SEAs, LEAs, and other operating agencies" to encourage SEAs to "fully utilize the experience, curriculum resources, program evaluation strategies, and technical assistance expertise of those IHEs operating HEP and CAMP programs [High School Equivalency Program and College Assistance Migrant Program projects authorized under Title IV, Section 418A, of the Higher Education Act]."

Response. No change has been made. The Migrant Education Interstate and Intrastate Coordination Program is intended to improve the coordination of migrant education activities, including those activities conducted under the Title I Migrant Education Program. Under that basic program, an SEA may conduct services directly, through subgrants to LEAs, or through special arrangements with other operating agencies. An "operating agency" may include—according to the definition in the Title I Migrant Education Program Regulations (34 CFR Part 204)—any public or nonprofit private agency, including an IHE. Therefore, in each instance that these regulations refer to "operating agencies," that term includes IHEs that may be serving as operating agencies under the Title I Migrant Education Program. Since some IHEs do serve as operating agencies under the Title I Migrant Education program, the Secretary expects that projects under the Migrant Education Interstate and Intrastate Coordination Program will include appropriate IHEs.

Comment. One commenter recommended that "interstate, or regional, parent advisory councils" should be included in the illustrative listing of activities—in § 205.11 of the regulations—that may be conducted under a project.

Response. No change has been made. The Secretary recognizes that interstate or regional parent advisory councils (PACs) may be useful endeavors. However, since parent advisory councils are not required under Chapter 1 of the Education Consolidation and Improvement Act of 1981, the Secretary has decided not to include such councils in the illustrative list of activities which may be supported under this program.

Comment. One commenter asked whether any consultation with the States had occurred—as required by the authorizing program statute—prior to the publication of the proposed regulations, particularly with respect to the listing of activities, in § 205.11 of the proposed regulations, that may be conducted under a project. This commenter further inquired concerning how the "appropriate consultation with SEAs"—mentioned in § 205.33 of the proposed regulations—would occur.

Response. No change is required by this comment. However, in response to the commenter's questions, the following information is provided.

Two meetings were held with SEAs—prior to the development of the proposed regulations—to meet the statutory mandate that the Secretary make grants and contracts under this program "in consultation with the States." As a result of those meetings, the Secretary developed some broad parameters for the types of projects that may be funded and the types of activities that may be conducted under those projects. The scope of these projects and activities was described in the proposed regulations.

Also—prior to the development of the proposed regulations—ED staff members had the opportunity, at a number of regularly-scheduled regional and national meetings of State directors of migrant education and other migrant education meetings involving other migrant education personnel, to discuss all matters that needed to be resolved and to solicit ideas, suggestions, and other advice.

As stated in § 205.33 (How are the annual priorities for funding established?), the Secretary "establishes * * * funding priorities to reflect * * * [a]ppropriate consultation with SEAs." The Secretary expects to effect this consultation through regular and special meetings with SEA migrant education personnel.

Comment. One commenter questioned the inclusion of the term "other operating agencies" in the proposed regulations, particularly in the statement of purpose in § 205.1. This commenter pointed out that the authorizing program statute only mentions SEAs and LEAs as the agencies among which projects should improve coordination of migrant education.

Response. No change has been made. Section 143 of Title I expresses the intent of the Congress, through this program, to improve the interstate and intrastate coordination of the educational programs available for migratory students. Specifically, the Congressional intent was to provide for coordination of migrant education activities conducted under the Title I Migrant Education Program authorized by Section 141 of Title I. Because the Title I Migrant Education Program provides services to migratory children through SEAs, LEAs, and other operating agencies, it is important to improve coordination of the migrant education activities conducted by all such agencies.

Comment. One commenter noted that the proposed regulations seemed to suggest that projects should be designed to improve coordiantion only among those SEAs, LEAs, and other operating agencies participating in the Title I Migrant Education Program, and not among any other agencies. This commenter pointed out that the authorizing program statute does not include this restriction.

Response. A change has been made. The authorizing program statute does not require a project to restrict its coordination to only SEAs and LEAs participating in the Title I Migrant Education Program. Similarly, these final regulations do not impose any such restriction. The Secretary agrees that it is important to coordinate all educational programs serving migratory children, including the programs of those SEAs and LEAs not participating in the Title I Migrant Education Program.

Accordingly, both § 205.1 (What is the Migrant Education Interstate and Intrastate Coordination Program?) and § 205.10 (What types of projects may be funded?) of these final regulations state that this program "is designed to improve interstate and intrastate coordination of migrant education activities among SEAs, LEAs, and other operating agencies, *including* those SEAs, LEAs, and other operating agencies participating in the * * * [basic program] * * *." Also, § 205.2 (Who is eligible to participate as a grantee?) of these final regulations opens the eligibility for a grant under this program to any SEA.

Comment. One commenter recommended that the coordination of summer school projects should be included in the illustrative listing of activities that may be conducted under a project. This commenter pointed out that the Congress has identified summer school projects (as well as secondary school projects—an area that is included in the listing) as an important concern and priority in migrant education.

Response. A change has been made. The Secretary agrees that summer

school projects are an important element of migrant education, and have been so identified by the Congress. The Secretary further agrees that improving the coordination of migrant education summer school projects (particularly, successful summer school project designs and elements) is highly desirable. Accordingly, the illustrative listing of activities—in § 205.11 of the final regulations (What types of activities may be conducted?) of the regulations—has been expanded to include "summer school project services."

Executive Order 12291

These regulations have been reviewed by the Department in accordance with Executive Order 12291 and are classified as non-major because they do not meet the criteria for major regulations established in the order.

The purpose of Executive Order 12291 of February 17, 1981, is to relieve regulatory burdens. The order requires ED, when promulgating new regulations, to—

(a) Base administrative decisions on adequate information concerning the need for, and consequences of, proposed government action;

(b) Ensure that a regulation's benefits to society outweigh its costs to society;

(c) Choose regulatory objectives that maximize the net benefits to society; and

(d) Choose the regulatory approach involving the least net cost to society.

The Secretary has, to the maximum extent possible, incorporated these requirements as part of the Department's procedures for promulgating regulations.

Regulatory Flexibility Act

The Secretary certifies that these regulations will not have a significant economic impact on a substantial number of small entities. Under these regulations, grants and contracts are available only to State agencies. As defined in the Regulatory Flexibility Act, "small entities" only includes small businesses, small organizations, and small governmental jurisdictions. The definition of "small governmental jurisdiction" does not include States or State agencies.

List of Subjects in 34 CFR Part 205

Education, Education of disadvantaged, Elementary and secondary education, Grant programs—education, Migrant labor.

Citation of Legal Authority

A citation of statutory or other legal authority is placed in parentheses on the line following each substantive provision of these regulations. References to "sec." in these citations refer to sections of Title I of the Elementary and Secondary Education Act of 1965, as amended by the Education Amendments of 1978.

Dated: April 15, 1982.

(Catalog of Federal Domestic Assistance No. 84.144; Migrant Education/Interstate and Intrastate Coordination Program)

T. H. Bell,

Secretary of Education.

The Secretary amends Title 34 of the Code of Federal Regulations by adding a new Part 205 to read as follows:

PART 205—GRANTS TO STATE EDUCATIONAL AGENCIES TO IMPROVE THE INTERSTATE AND INTRASTATE COORDINATION OF MIGRANT EDUCATION ACTIVITIES

Subpart A—General

Sec.
205.1 What is the Migrant Education Interstate and Intrastate Coordination Program?
205.2 Who is eligible to participate as a grantee?
205.3 What regulations apply to this program?
205.4 What definitions apply to this program?

Subpart B—What Kind of Activities Does the Secretary Assist Under This Program?

205.10 What types of projects may be funded?
205.11 What types of activities may be conducted?

Subpart C—How Does an SEA Apply for a Grant?

205.20 What specific information must be included in a group application?
205.21 How must an SEA develop its application?

Subpart D—How Is a Grant Made to an SEA?

205.30 How is an application evaluated?
205.31 What are the selection criteria for reviewing an application?
205.32 What are the factors considered in awarding a grant?
205.33 How are the annual priorities for funding established?

Authority: Part B, Subpart 1, Section 143 of Title I of the Elementary and Secondary Education Act of 1965 (Pub. L. 89–10), as amended by the Education Amendments of 1978 (Pub. L. 95–561) (20 U.S.C. 2763).

Subpart A—General

§ 205.1 What is the Migrant Education Interstate and Intrastate Coordination Program?

The Migrant Education Interstate and Intrastate Coordination Program is designed to provide financial assistance to State educational agencies (SEAs) for projects designed to improve interstate and intrastate coordination of migrant education activities among SEAs, local educational agencies (LEAs), and other operating agencies, including those SEAs, LEAs, and other operating agencies participating in the Migrant Education Program authorized by Section 141 of Title I of the Elementary and Secondary Education Act of 1965, as amended (Title I).

(Sec. 143; 20 U.S.C. 2763)

§ 205.2 Who is eligible to participate as a grantee?

Only SEAs, either individually or cooperatively (i.e., through a group or consortium), may apply for a grant under this program.

(Sec. 143; 20 U.S.C. 2763)

§ 205.3 What regulations apply to this program?

The following regulations apply to this program:

(a) The Education Department General Administrative Regulations (EDGAR) in 34 CFR Part 75 (Direct project grant and contract programs) and 34 CFR Part 77 (Definitions).

(b) The regulations in this Part 205.

(Sec. 143; 20 U.S.C. 2763)

§ 205.4 What definitions apply to this program?

The definitions in the Title I Migrant Education Program Regulations (34 CFR Part 204) apply to this program.

(Sec. 143; 20 U.S.C. 2763)

Subpart B—What Kinds of Activities Does the Secretary Assist Under This Program?

§ 205.10 What types of projects may be funded?

The Secretary may make grants to an SEA or SEAs to carry out projects designed to improve interstate and intrastate coordination of migrant education activities among SEAs, LEAs, and other operating agencies, including those SEAs, LEAs, and other operating agencies participating in the Title I Migrant Education Program.

(Sec. 143; 20 U.S.C. 2763)

§ 205.11 What types of activities may be conducted?

The projects may include, but are not limited to, the following activities:

(a) *Parental involvement.* This type of project might include activities such as—

(1) Identifying and designing—for dissemination on a regional and nationwide basis—effective strategies for parental involvement and the

training of parent advisory council members; and

(2) Providing technical assistance to SEAs, LEAs, and other operating agencies in implementing strategies—that have been effective in other migrant education projects—for parental involvement and the training of parent advisory council members.

(b) *Resource centers.* This type of project might include activities such as—

(1) Identifying and designing—for dissemination on a regional and nationwide basis—effective materials (such as instruments and procedures for needs assessment surveys, student assessment instruments, curricular materials, and evaluation methods and materials); and

(2) Disseminating information about the availability of experts and other resources in the field of migrant education.

(c) *Identification and recruitment of children.* This type of project might include activities such as—

(1) Identifying and designing—for dissemination on a regional and nationwide basis—effective strategies and materials for the identification and recruitment of migratory children;

(2) Developing a coordinated nationwide program for the identification and recruitment of migratory children, including the design of model strategies and materials; and

(3) Providing technical assistance to SEAs, LEAs, and other operating agencies in implementing project designs and materials—that have been effective in other migrant education projects—for the identification and recruitment of migratory children.

(d) *Secondary school services.* This type of project might include activities such as—

(1) Identifying and designing—for dissemination on a regional and nationwide basis—effective project designs and materials from migrant education secondary school projects (such as career education, vocational instruction, dropout prevention projects, and the transfer of school credits); and

(2) Providing technical assistance to SEAs, LEAs, and other operating agencies in implementing project designs and materials—that have been effective in other migrant education projects—for secondary school services for migratory children.

(e) *Information and dissemination center.* This type of project might include activities such as—

(1) Conducting a nationwide awareness project for the Title I Migrant Education Program—directed to the parents of eligible children, other parents and adults, the general education and educational research communities, and other family and child service agencies; and

(2) Designing and disseminating Title I Migrant Education Program informational materials.

(f) *Staff development services.* This type of project might include activities such as identifying and designing effective interstate training strategies for Title I Migrant Education Program staff members.

(g) *Interagency coordination.* This type of project might include activities such as—

(1) Identifying and designing—for dissemination on a regional and nationwide basis—effective strategies of interagency coordination of services to migratory children; and

(2) Providing technical assistance to SEAs, LEAs, and other operating agencies in implementing strategies—that have been effective in other migrant education projects—of interagency coordination of services to migratory children.

(h) *Record transfer system uses.* This type of project might include activities such as exploring and designing strategies of additional uses for the existing migrant student record transfer system (e.g., program management information, instructional information refinements, skills information transmittal) to facilitate coordination of services among school districts and to enhance the continuity of education for migratory children.

(i) *Project evaluation.* This type of project might include activities such as—

(1) Identifying and designing—for dissemination on a regional and nationwide basis—effective evaluation strategies and materials for migrant education projects (e.g., strategies for short-term projects, strategies for using the migrant student record transfer system in assessment and evaluation); and

(2) Providing technical assistance to SEAs, LEAs, and other operating agencies in implementing strategies—that have been effective in other migrant education projects—for the evaluation of migrant education projects.

(j) *Summer school project services.* This type of project might include activities such as—

(1) Identifying and designing—for dissemination on a regional and nationwide basis—effective project designs from migrant education summer school projects; and

(2) Providing technical assistance to SEAs, LEAs, and other operating agencies in implementing project designs and materials—that have been effective in other migrant education projects—for summer school project services for migratory children.

(Sec. 143; 20 U.S.C. 2763)

Subpart C—How Does an SEA Apply for a Grant?

§ 205.20 What specific information must be included in a group application?

In applying for a grant, an SEA shall provide information relevant to any proposed consortium of SEAs (for a group application only) including—

(a) An identification of each SEA that is to participate in the consortium;

(b) A statement of commitment, indicating the terms of the commitment, from each SEA proposed to participate in the consortium;

(c) A description of the proposed objectives of the consortium; and

(d) A description of how each SEA proposed to participate in the consortium was involved in the development of the proposed objectives and activities of the project.

(Sec. 143; 20 U.S.C. 2763)

§ 205.21 How must an SEA develop its application?

An applicant SEA under the Migrant Education Interstate and Intrastate Coordination Program shall plan and develop its project in consultation and coordination with other SEAs or with participating LEAs, as appropriate.

(Sec. 143; 20 U.S.C. 2763)

Subpart D—How Is a Grant Made to an SEA?

§ 205.30 How is an application evaluated?

(a) The Secretary evaluates an application under this program on the basis of the criteria in § 205.31 of this Part.

(b) The Secretary awards up to 100 possible points for meeting these criteria.

(c) The maximum number of points possible for meeting each individual criterion is indicated in parentheses after the heading for that criterion.

(Sec. 143; 20 U.S.C. 2763; 20 U.S.C. 1221e–3(a)(1))

§ 205.31 What are the selection criteria for reviewing an application?

(a) *Plan of operation.* (35 points)

(1) The Secretary reviews each application for information that shows the quality of the plan of operation for the project.

(2) The Secretary looks for information that shows the following:

(i) High quality in the design of the project.

(ii) An effective plan of management that insures proper and efficient administration of the project.

(iii) A clear description of how the objectives of the project relate to the purpose of the program.

(iv) A clear description of the way that the applicant SEA plans to use its resources and personnel to achieve each objective of the project.

(v) A clear description of how the applicant SEA will provide equal access and treatment for eligible project participants who are members of groups that have been traditionally underrepresented, such as—

(A) Members of racial or ethnic minority groups;

(B) Women; and

(C) Handicapped persons.

(vi) A clear description of how the applicant SEA will provide an opportunity for participation of students enrolled in private schools.

(b) *Evaluation plan.* (20 points)

(1) The Secretary reviews each application for information that shows the quality of the evaluation plan for the project.

Cross-reference. See 34 CFR 75.590 of EDGAR (Evaluation by the grantee).

(2) The Secretary looks for information that shows methods of evaluation that are appropriate for the project and, to the extent possible, are objective and produce data that are quantifiable.

(c) *Quality of key personnel.* (15 points)

(1) The Secretary reviews each application for information that shows adequate qualifications of the key personnel the applicant SEA plans to use in the project.

(2) The Secretary looks for information that shows the following:

(i) The qualifications of the project director (if one is to be used).

(ii) The qualifications of each of the other key personnel to be used in the project.

(iii) The time that each person referred to in paragraphs (c)(2) (i) and (ii) of this section plans to commit to the project.

(iv) The extent to which the applicant SEA, as part of its nondiscriminatory employment practices, encourages applications for employment from persons who are members of groups that have been traditionally underrepresented, such as—

(A) Members of racial or ethnic minority groups;

(B) Women;

(C) Handicapped persons; and

(D) The elderly.

(3) To determine personnel qualifications, the Secretary considers experience and training—in fields related to the objectives of the project—as well as other information that the applicant SEA provides.

(d) *Adequacy of resources.* (10 points)

(1) The Secretary reviews each application for information that shows that the applicant SEA plans to devote adequate resources to the project.

(2) The Secretary looks for information that shows the following:

(i) The facilities that the applicant SEA plans to use are adequate.

(ii) The equipment and supplies that the applicant SEA plans to use are adequate.

(e) *Annual priorities.* (10 points)

(1) The Secretary reviews each application for information that shows the extent to which the applicant SEA's proposed project addresses one of the annual priorities for funding under this program, as announced in the program's application notice published in the **Federal Register.**

(2) The Secretary looks for information that shows that the applicant SEA's proposed project addresses one of the annual priorities for funding.

(f) *Budget and cost effectiveness.* (5 points)

(1) The Secretary reviews each application for information that shows that the project has an adequate budget and is cost effective.

(2) The Secretary looks for information that shows the following:

(i) The budget for the project is adequate to support the project activities.

(ii) Costs are reasonable in relation to the objectives of the project.

(g) *Interstate and intrastate consultation and coordination.* (5 points)

(1) The Secretary reviews each application for information that shows the quality of the applicant SEA's consultation and coordination with other SEAs or with participating LEAs, as appropriate.

(2) The Secretary looks for information that shows that the applicant SEA—

(i) Has consulted and coordinated adequately with other SEAs or participating LEAs, as appropriate, in planning and developing its project; and

(ii) Will consult and coordinate adequately with other SEAs or participating LEAs, as appropriate, in implementing and evaluating its project.

(Sec. 143; 20 U.S.C. 2763; 20 U.S.C. 1221e-3(a)(1))

§ 205.32 What are the factors considered in awarding a grant?

In awarding grants, the Secretary considers—

(a) The amount of funds available for grants under the program; and

(b) The rank order of the applications—as determined by using the criteria listed in § 205.31 of this Part.

(Sec. 143; 20 U.S.C. 2763; 20 U.S.C. 1221e-3(a)(1))

§ 205.33 How are the annual priorities for funding established?

(a) *General.*

(1) Each fiscal year, the Secretary announces in the application notice—published in the **Federal Register**—any national priorities relating to the types of projects to be considered for funding under this program.

(2) The Secretary may select one or more of these priorities from the illustrative list of activities in § 205.11 of this Part.

(b) *Basis for determining priorities.* The Secretary establishes these funding priorities to reflect—

(1) Any unmet national needs in interstate and intrastate coordination; and

(2) Appropriate consultation with SEAs.

(Sec. 143; 20 U.S.C. 2763; 20 U.S.C. 2734)

[FR Doc. 82-10881 Filed 4-20-82; 8:45 am]
BILLING CODE 4000-01-M

federal register

Wednesday
April 21, 1982

Part VII

Department of Education

Cooperative Education Program

DEPARTMENT OF EDUCATION

34 CFR Parts 631, 632, 633, 634, and 635

Cooperative Education Program

AGENCY: Department of Education.

ACTION: Final regulations.

SUMMARY: The Secretary of Education issues final regulations governing the administration of the Cooperative Education Program. The regulations are published to interpret and implement the statutory provisions authorizing the program and to carry out the deregulation initiatives of the President and Secretary. The regulations provide information to enable eligible applicants to apply for funds, establish the criteria by which the Secretary evaluates applications, and set minimum standards and requirements for the operation of funded projects.

EFFECTIVE DATE: Unless Congress takes certain adjournments, these regulations will take effect 45 days after publication in the **Federal Register.** If you want to know the effective date of these regulations, call or write the Department of Education contact person. At a future date the Secretary will publish a notice in the **Federal Register** stating the effective date of these regulations.

FOR FURTHER INFORMATION CONTACT: Barbara W. Freeman, Department of Education, 400 Maryland Avenue, SW., (Room 3053, ROB-3), Washington, D.C. 20202, Telephone: (202) 245-2146.

SUPPLEMENTARY INFORMATION:

Background

On August 4, 1981, the Secretary published final regulations for this program in the **Federal Register,** Vol. 46, No. 149. Those regulations govern only grants awarded in fiscal year 1981. The Secretary requested but received only one public comment on those final regulations. The Secretary, however, has reviewed those regulations for further opportunities for deregulation and has further considered certain comments received on the proposed rules published in December 1980. In these final regulations, the Secretary has deleted certain provisions imposing requirements on grantees that he has determined to be overly prescriptive and to intrude into institutional responsibilities. In addition, the Secretary has incorporated in these final regulations comments received on the proposed rules published in December 1980.

Following is a summary of the comments received on the proposed rules published on December 31, 1980, that have been incorporated into these regulations:

Section 632.2 Eligible applicants.

Comment. Five commenters objected to the limitation on the number of institutions of higher education that may form a combination of institutions for purposes of applying for an administration grant. One of the commenters also objected to the requirement that member institutions be located within the boundaries of a State and suggested that if any reference is made to the location of the institutions, that reference should be based on geographic commonality rather than on the boundaries of a State.

Response. A change has been made. The Secretary has removed the limit on the number of institutions of higher education that may enter into a combination. Further, member institutions need not be located within the boundaries of a State. The Secretary encourages members of combinations to use grant funds primarily for activities that directly benefit students, rather than for coordinating activities of the member institutions.

Section 632.30 (formerly § 632.31) Selection criteria the Secretary uses.

Comment. Two commenters pointed out the redundancy of the criteria in paragraphs (l), (n), and (p), all of which relate to the work experience for students. One commenter suggested the deletion of paragraphs (l) and (m), which address related work experiences and employer acceptance, since, in the opinion of the commenter, the responses to these criteria are likely to result in verbiage and have no substance.

This commenter and an additional commenter suggested combining paragraph (g), which addresses administrative support for the proposed project, and paragraph (k), which addresses how applicants will continue the project after Federal assistance ends, since both refer to essentially the same criteria.

The latter commenter also stated that paragraphs (e) and (i) are very similar because those paragraphs address, respectively, the resources an applicant will devote to the proposed project and how those resources will be used.

One of the commenters suggested that the Secretary increase the point value of paragraph (n), which addresses the integration of the work experiences, because that integration is a major component of quality programming in Cooperative Education. The commenter also requested a clarification of how an applicant demonstrates—in response to paragraph (q)—the extent and quality of the supervision students will receive during their work experiences.

This commenter was joined by another commenter in recommending a reassessment of paragraph (r), which addresses an applicant's experience in providing Cooperative Education opportunities.

Response. A change has been made. The Secretary has grouped the elements of the criteria according to their relatedness. The former criteria to evaluate *Institutional philosophy, Administrative support,* and *Continuing support of Cooperative Education* have been combined into one criterion entitled *Institutional commitment,* valued at 19 points. The Secretary has made similar and appropriate modifications to the selection criteria the Secretary uses to evaluate applications for demonstration and exploration grants and training grants.

Section 634.2 Eligible applicants.

Comment. Three commenters suggested that public and nonprofit agencies and organizations be allowed to apply for research and training grants without serving as the coordinating and fiscal agent for a combination of institutions of higher education.

Response. A change has been made. Public and nonprofit private agencies and organizations may apply for research and training grants without serving as the coordinating and fiscal agent for an institution of higher education or a combination of institutions of higher education.

The Secretary has made the following additional changes:

The regulations have been edited to clarify certain provisions and to adhere more closely to statutory intent.

With the exception of general administrative selection criteria, the Secretary has deleted from these regulations provisions that duplicate requirements in the Education Department General Administrative Regulations (EDGAR). Readers are advised to read carefully all applicable provisions in 34 CFR Part 75 (Direct Grant Programs) and Part 77 (Definitions), as well as applicable provisions in Part 74 (Administration of Grants) that are referred to in Part 75.

Executive Order 12291

These regulations have been reviewed by the Department in accordance with Executive Order 12291. They are classified as non-major regulations because they do not meet the criteria for major regulations established in the order.

Regulatory Flexibility Act Certification

The Secretary certifies that these regulations will not have a significant economic impact on a substantial number of small entities. The estimated costs of complying with these regulations, including the costs of gathering the information and completing the forms necessary to obtain funds, will not have a significant economic impact on small entities participating in this program.

Invitation to Comment

To assist the Department in complying with the specific requirements of Executive Order 12291 and its overall objective of reducing regulatory burden, public comment is invited on whether there may be further opportunities to reduce any regulatory burdens found in these regulations, especially with regard to paperwork and compliance requirements.

List of Subjects in 34 CFR Parts 631, 632, 633, 634, and 635

Colleges and universities, Education, Employment, Grant programs—education, Manpower training programs, Student aid, Students, Teachers, Educational research (Parts 631 and 634 only).

Citation of Legal Authority

A citation of statutory or other legal authority is placed in parentheses on the line following each substantive provision of these regulations.

(Catalog of Federal Domestic Assistance Numbers 84.055, Cooperative Education Program; 84.055A, Cooperative Education—Administration; 84.055B, Cooperative Education—Demonstration and Exploration; 84.055C, Cooperative Education—Research; 84.055D, Cooperative Education—Training)

Dated: April 16, 1982.

T. H. Bell,

Secretary of Education.

The Secretary revises Part 631 of Title 34 of the Code of Federal Regulations to read as follows:

PART 631—COOPERATIVE EDUCATION PROGRAM—GENERAL

Subpart A—General

Sec.
631.1 The Cooperative Education Program.
631.2 Regulations that apply to the Cooperative Education Program.
631.3 Definitions that apply to the Cooperative Education Program.

Subpart B—Projects the Secretary Assists Under This Program

631.10 General.
631.11 Administration projects.
631.12 Demonstration and exploration projects.
631.13 Research projects.
631.14 Training projects.

Subpart C—How To Apply for a Grant

631.20 Limitation on number of applications.
631.21 Application information.

Subpart D—How the Secretary Makes a Grant

631.30 How the Secretary makes a grant.
631.31 Evaluation of applications from combinations of institutions of higher education.
631.32 Minimal acceptable score.

Subpart E—Conditions That Must Be Met by a Grantee

631.40 Allowable costs.
631.41 Unallowable costs.

Authority: Title VIII of the Higher Education Act of 1965, as amended (20 U.S.C. 1133–1133b), unless otherwise noted.

Subpart A—General

§ 631.1 The Cooperative Education Program.

The Cooperative Education Program provides Federal financial assistance to help institutions of higher education offer students paid work experiences closely related to their academic and career pursuits, and to provide for specialized training and research to improve the effectiveness of programs of Cooperative Education.

(20 U.S.C. 1133)

§ 631.2 Regulations that apply to the Cooperative Education Program.

The following regulations apply to the Cooperative Education Program:

(a) The Education Department General Administrative Regulations (EDGAR) in 34 CFR Part 75 (Direct Grant Programs) and 34 CFR Part 77 (Definitions).

(b) The regulations in this Part 631.

(c) The regulations in the following parts, as applicable:

(1) 34 CFR Part 632—Cooperative Education—Administration Projects.

(2) 34 CFR Part 633—Cooperative Education—Demonstration and Exploration Projects.

(3) 34 CFR Part 634—Cooperative Education—Research Projects.

(4) 34 CFR Part 635—Cooperative Education—Training Projects.

(20 U.S.C. 1221e-3(a)(1), 1133)

§ 631.3 Definitions that apply to the Cooperative Education Program.

(a) *Definitions in EDGAR.* The following terms used in 34 CFR Parts 631 through 635 are defined in 34 CFR Part 77:

Applicant
Application
Contract
EDGAR
Equipment
Grant
Grantee
Nonprofit
Private
Project
Project period
Public
Secretary
State

(b) *Definitions that apply to the Cooperative Education Program.* The following definitions apply to terms used in 34 CFR Parts 631 through 635:

"Act" means Title VIII of the Higher Education Act of 1965, as amended.

"Alternating periods of study and employment" means rotating periods of classroom study by, and supervised employment of, a student in a Cooperative Education project.

"Combination of institutions of higher education" means a group consisting of institutions of higher education that have entered into a cooperative arrangement for the purpose of carrying out a common objective.

"Comprehensive Cooperative Education project" means a Cooperative Education project in an institution of higher education that—

(1) Expands an existing program of Cooperative Education into all or nearly all of the academic disciplines or departments of the institution;

(2) Serves, to the extent possible, a diverse student population, as described in 34 CFR 75.111;

(3) Enables students to participate in work experiences with a variety of employers; and

(4) Acts as a liaison between high schools and the institution's admissions office to inform high school students of the availability and advantages of Cooperative Education.

"Cooperative Education" means an educational approach that provides alternating or parallel periods of classroom study and public or private employment in which—

(1) There is a formal agreement among the institution of higher education, the student, and the employer;

(2) The rotation between academic study and work is structured to further the student's education and employability;

(3) The work experience is related to the student's academic study or career goals, so far as practicable;

(4) The student's work experience is supervised and evaluated; and

(5) The student is employed and compensated in conformity with Federal, State, and local laws.

"Institution of higher education" means an educational institution as

defined in Section 1201(a) of the Higher Education Act of 1965, as amended, but does not include any institution that does not meet the provision of clause (3) of Section 1201(a).

"Parallel periods of study and employment" means periods of both classroom study by, and supervised employment of, a student in a Cooperative Education project, with the study and employment having nearly equal significance during the course of the same school day or the school week.

"Student" means a person—

(1) Enrolled in an institution of higher education other than by correspondence;

(2) Enrolled in a degree program of not fewer than two years; and

(3) Carrying at least half the academic workload normally required of persons who are full-time degree candidates.

"Unit of an institution of higher education."

(1) This term means the organizational entity that has the final, noncentral administrative authority to recommend or administer the requirements, standards, and credits necessary to earn academic degrees.

(2)(i) In a university, unit means a college or its equivalent within the university.

(ii) In a four-year college, unit means a school or its equivalent within the college.

(iii) In a two-year college, unit means a department or division, whichever is the higher level, within the college.

(20 U.S.C. 1221e-3(a)(1), 1133-1133b)

Subpart B—Projects the Secretary Assists Under This Program

§ 631.10 General.

Under the Cooperative Education Program the Secretary awards grants for four types of projects:

(a) Administration projects.

(b) Demonstration and exploration projects.

(c) Research projects.

(d) Training projects.

(20 U.S.C. 1133-1133b)

§ 631.11 Administration projects.

(a) Administration projects are designed to enrich the quality of higher education by providing students with paid work experiences related, to the extent practicable, to the student's academic or occupational objectives.

(b) The purpose of the work experiences is to enable students to—

(1) Gain knowledge that better prepares them to achieve their career objectives; and

(2) Earn funds needed for educational expenses.

(20 U.S.C. 1133a)

§ 631.12 Demonstration and exploration projects.

(a) Demonstration projects are comprehensive Cooperative Education projects designed to demonstrate the feasibility of expanding Cooperative Education in an institution to encompass—

(1) Nearly all academic departments or disciplines; and

(2) A significant majority of a diverse student population.

(b) Exploration projects are projects designed to explore the feasibility of instituting innovative methods for carrying out Cooperative Education projects—

(1) In various geographic settings or in various types and sizes of institutions;

(2) To meet the special problems or needs of special populations; or

(3) Both.

(20 U.S.C. 1133b)

§ 631.13 Research projects.

Research projects have broad implications for an application to the entire Cooperative Education community—including, but not limited to, students, institutions, and employers—rather than application to the immediate needs of an individual institution of higher education.

(20 U.S.C. 1133b)

§ 631.14 Training projects.

Training projects offer a variety of activities and experiences—

(a) Designed to carry out one or more of the types of training specified in 34 CFR 635.10; and

(b) Directed to individuals who participate or are interested in participating in the planning, establishment, administration, or coordination of Cooperative Education projects.

(20 U.S.C. 1133b)

Subpart C—How to Apply for a Grant

§ 631.20 Limitation on number of applications.

In any year, the Secretary evaluates from the same applicant no more than one application under each of the four types of projects listed in § 631.10.

(20 U.S.C. 1221e-3(a)(1), 1133-1133b)

§ 631.21 Application information.

In addition to the information specified in 34 CFR 75.107, an application must contain a detailed narrative budget for each year of the proposed project period, including, where applicable—

(a) The purposes of staff travel; and

(b) The reasons for the use of consultants.

(20 U.S.C. 1221e-3(a)(1), 1133a, 1133b)

Subpart D—How the Secretary Makes a Grant

§ 631.30 How the Secretary makes a grant

(a) The Secretary evaluates an application on the basis of the selection criteria located in the appropriate part governing the component under which an application is made.

(b) The Secretary awards up to 100 possible points for these criteria.

(c) The maximum possible score for each criterion is indicated in parentheses following the title of that criterion in 34 CFR 632.30, 633.30, 633.31, 634.30, or 635.30, as applicable.

(20 U.S.C. 1221e-3(a)(1), 1133-1133b)

§ 631.31 Evaluation of applications from combinations of institutions of higher education.

(a) The Secretary evaluates an application from a combination of institutions of higher education as a single application.

(b) The Secretary does not give member institutions of a group individual scores for the applicable selection criteria.

(20 U.S.C. 1221e-3(a)(1), 1133-1133b)

§ 631.32 Minimal acceptable score.

The Secretary does not give further consideration for funding to any application that receives an average score of 50 points or less from the panel of experts in the review process conducted in accordance with 34 CFR 75.217.

(20 U.S.C. 1221e-3(a)(1), 1133-1133b)

Subpart E—Conditions That Must Be Met by a Grantee

§ 631.40 Allowable costs.

Consistent with the terms of 34 CFR 75.530, governing allowable costs, a grantee may pay from its grant the costs of—

(a) Release time (that is, time in which a faculty member is freed from his or her normal teaching load to serve the project) or overload time (that is, time beyond a faculty member's normal teaching load at that institution) to faculty who assist the project in such activities as—

(1) Student counseling and assessment of students' progress in work experiences that are part of the project;

(2) Job development, location, and placement; or

(3) Promotional activities for Cooperative Education.

(b) Conducting Cooperative Education seminars or courses for students.

(c) Student travel, if a student is a member of an advisory board for the project.

(d) Fees, per diem expenses, and travel costs of consultants and trainers.

(e) Per diem expenses and travel costs of unpaid consultants, such as key personnel of other Cooperative Education projects.

(f) In-service training related to Cooperative Education.

(g) Registration for training sessions sponsored by the Cooperative Education Program.

(h) Developing, printing, and disseminating materials related to the project, including material designed to recruit into the project students from the enrolled student body, and films, audio-visual materials, and other illustrative materials for classroom use.

(i) Compensating graduate students who serve as research assistants.

(20 U.S.C. 1221e-3(a)(1), 1133-1133b)

§ 631.41 Unallowable costs.

In addition to those costs disallowed under 34 CFR 75.519 and 34 CFR 75.530 through 75.533, a grantee may not use its grant to pay for the following:

(a) Compensation or expenses of students enrolled in the Cooperative Education project.

(b) Teaching salaries for academic courses.

(c) Consultant fees or honoraria to key personnel—such as directors, coordinators, job developers, job locators, and career counselors—who are—

(1) Involved in carrying out the objectives of a Cooperative Education project;

(2) Salaried, in whole or in part, under the Cooperative Education Program, or any other Federal program; or

(3) Both.

(d) Purchase or lease of equipment, unless specifically authorized by the Secretary.

(e) Recruitment activities to encourage students to enroll in the grantee institution.

(f) Computer usage and materials—such as pre-programmed materials, purchase of computer time, programming, and rental of terminals for the project—unless specifically approved by the Secretary.

(g) Individual membership fees in professional organizations.

(h) Institutional membership fees in organizations that devote a substantial part of their activities to influencing the passage or defeat of legislation.

(20 U.S.C. 1221e-3(a)(1), 1133-1133b)

The Secretary revises Part 632 of Title 34 of the Code of Federal Regulations to read as follows:

PART 632—COOPERATIVE EDUCATION PROGRAM—ADMINISTRATION PROJECTS

Subpart A—General

Sec.
632.1 The Cooperative Education Program—Administration Projects.
632.2 Eligible applicants.
632.3 Eligible students.

Subpart B—Projects the Secretary Assists Under This Program

632.10 Eligible projects.

Subpart C—How to Apply for a Grant

632.20 Application information.

Subpart D—How the Secretary Makes a Grant

632.30 Selection criteria the Secretary uses.
632.31 Extent of Federal support.
632.32 Limit on years of participation.

Subpart E—Conditions That Must Be Met by a Grantee

632.40 Fiscal requirements.

Subpart F—Administrative Responsibilities of a Grantee

632.50 Frequency and duration of work experiences.
632.51 Assessment of a student's progress.
632.52 In-service training.

Authority: Title VIII of the Higher Education Act of 1965, as amended (20 U.S.C. 1133-1133a), unless otherwise noted.

Subpart A—General

§ 632.1 The Cooperative Education Program—Administration Projects.

Administration projects are described in 34 CFR 631.11.

(20 U.S.C. 1133a)

§ 632.2 Eligible applicants.

The following are eligible to apply for grants under this part:

(a) Institutions of higher education.

(b) Combinations of institutions of higher education.

(20 U.S.C. 1133a)

§ 632.3 Eligible students.

To participate in a Cooperative Education project funded under this part, an individual must meet the definition of the term "student" in 34 CFR 631.3(b).

(20 U.S.C. 1221e-3(a)(1), 1133a)

Subpart B—Projects the Secretary Assists Under This Program

§ 632.10 Eligible projects.

The Secretary makes awards under this part for Cooperative Education projects in which students engage in alternating or parallel periods of study and employment.

(20 U.S.C. 1133a)

Subpart C—How To Apply for a Grant

§ 632.20 Application information.

In addition to the provisions specified in 34 CFR 631.21 an applicant must provide the following:

(a) A list of the units of the institution of higher education.

(b) The number of years each unit has received prior funding for an administration project under Title VIII or under the former Title IV, Part D, of the Higher Education Act of 1965, the predecessor of Title VIII.

(20 U.S.C. 1221e-3(a)(1), 1133a)

Subpart D—How the Secretary Makes a Grant

§ 632.30 Selection criteria the Secretary uses.

The Secretary uses the following criteria in evaluating applications for grants under this part.

(a) *Plan of operation.* (10 points) (1) The Secretary reviews each application for information that shows the quality of the plan of operation for the project.

(2) The Secretary looks for information that shows—

(i) High quality in the design of the project;

(ii) An effective plan of management that ensures proper and efficient administration of the project;

(iii) A clear description of how the objectives of the project relate to the purpose of the program;

(iv) The way the applicant plans to use its resources and personnel to achieve each objective; and

(v) A clear description of how the applicant will provide equal access and treatment for eligible project participants who are members of groups that have been traditionally under represented, such as—

(A) Members of racial or ethnic minority groups;

(B) Women;

(C) Handicapped persons; and

(D) The elderly.

(b) *Involvement in planning and implementation.* (4 points) The Secretary reviews each application for information that shows the extent to which and the manner in which the applicant has involved administrators, faculty, students, employers, and Cooperative Education specialists in planning and—if a Cooperative Education project is already in existence—in carrying out the project for which funds are requested.

(c) *Expansion of Cooperative Education.* (10 points) The Secretary reviews each application for information that shows the extent to which and the manner in which the applicant has used or will use its own resources to increase the size, scope, and quality of its Cooperative Education project during the period of Federal support.

(d) *Curriculum and calendar adaptability.* (5 points) The Secretary reviews each application for information that shows the extent to which the applicant has modified or will modify its schedule of courses as necessary to meet the particular needs of students in the proposed project.

(e) *Supervision of students.* (4 points) The Secretary reviews each application for information that shows the extent and quality of the supervision students will receive from the grantee or employer during the work experiences.

(f) *Recordkeeping.* (3 points) The Secretary reviews each application for information that shows the extent to which the applicant has developed or will develop a recordkeeping system that documents each student's status, while enrolled in the Cooperative Education project.

(g) *Quality of key personnel.* (7 points) (1) The Secretary reviews each application for information that shows the quality of the key personnel the applicant plans to use on the project.

(2) The Secretary looks for information that shows—

(i) The qualifications of the project director or principal investigator;

(ii) The qualifications of each of the other key personnel to be used in the project;

(iii) The time that each person referred to in paragraphs (b)(2) (i) and (ii) of this section will commit to the project; and

(iv) The extent to which the applicant, as part of its nondiscriminatory employment practices, encourages, applications for employment from persons who are members of groups that have been traditionally underrepresented, such as members of racial or ethnic minority groups, women, handicapped persons, and the elderly.

(3) To determine personnel qualifications, the Secretary considers evidence of past experinece and training, in fields related to the objectives of the project, as well as other information that the applicant provides.

(h) *Budget and cost effectiveness.* (5 points) (1) The Secretary reviews each application for information that shows that the project has an adequate budget and is cost effective.

(2) The Secretary looks for information that shows—

(i) The budget for the project is adequate to support the project activities; and

(ii) Costs are reasonable in relation to the objectives of the project.

(i) *Evaluation plan.* (5 points) (1) The Secretary reviews each application for information that shows the quality of the evaluation plan for the project.

(See 34 CFR 75.590—Evaluation by the grantee)

(2) The Secretary looks for information that shows methods of evaluation that are appropriate for the project and, to the extent possible, are objective and produce data that are quantifiable.

(j) *Adequacy of resources.* (3 points) (1) The Secretary reviews each application for information that shows that the applicant plans to devote adequate resources to the project.

(2) The Secretary looks for information that shows—

(i) The facilities that the applicant plans to use are adequate; and

(ii) The equipment and supplies that the applicant plans to use are adequate.

(k) *Institutional commitment.* (19 points) The Secretary reviews each application for evidence that shows—

(1) The applicant has a written Cooperative Education philosophy appropriate to the needs and characteristics of the applicant and has a clear understanding of the Title VIII Cooperative Education Program;

(2) The extent to which and the manner in which the principal administrators of the applicant have supported or are likely to support the proposed project; and

(3) The applicant demonstrates its intention and ability to continue Cooperative Education at the same or an increased level after Federal financial assistance ends.

(l) *Work experiences.* (10 points) (1) The Secretary reviews each application for information that describes the work experiences that will be provided by the project.

(2) The Secretary looks for information that shows—

(i) The extent to which the applicant has arranged for a sufficient number of employment opportunities that are related to the educational, professional, or occupational objectives of students involved or to be involved in the project;

(ii) That the number, frequency, length, and quality of the work experiences are sufficient to make a significant contribution toward meeting the educational, professional, and occupational objectives of the student's involved or to be involved in the project; and

(iii) The extent to which the applicant will develop, promote, and monitor close relationships between the students' work experiences and their academic studies.

(m) *Employer acceptance.* (15 points) The Secretary reviews each application for evidence that shows the extent to which employers have provided or will provide opportunities for work and career development related to the academic disciplines of students involved or to be involved in the project.

(20 U.S.C. 1221e-3(a)(1), 1133a)

§ 632.31 Extent of Federal support.

(a) If the Secretary selects a project for assistance under this part, the Secretary awards Federal funds that do not exceed the following:

(1) One hundred percent of the total project cost in the first fiscal year for which an award is made.

(2) Ninety percent of the total project costs in the second fiscal year for which an award is made.

(3) Eighty percent of the total project cost in the third fiscal year for which an award is made.

(4) Sixty percent of the total project cost in the fourth fiscal year for which an award is made.

(5) Thirty percent of the total project cost in the fifth fiscal year for which an award is made.

(b) If an applicant has previously received funds under this part for the applicant as a whole or for a unit of the the applicant, the Secretary, in computing the maximum possible Federal share of an award, counts as one year each previous fiscal year for which an award was made to the applicant or unit.

(20 U.S.C. 1133a)

§ 632.32 Limit on years of participation.

A unit of an institution of higher education, individually or as a participant in a combination of institutions of higher education, may not receive funding under this part for more than five fiscal years.

(20 U.S.C. 1133a)

Subpart E—Conditions That Must Be Met by a Grantee

§ 632.40 Fiscal requirements.

A grantee shall expend for its Cooperative Education project during the fiscal year in which a grant is received not less than the amount the grantee expended from non-Federal funds for its project during the previous

fiscal year for which Federal funds were received.

(20 U.S.C. 1133a)

Subpart F—Administrative Responsibilities of a Grantee

§ 632.50 Work experiences.

(a) A Cooperative Education project in an institution of higher education must provide to participating students work experiences which, to the extent practicable, relate to the student's program of academic study and career or occupational objectives.

(b) The work experiences provided under paragraph (a) of this section must—

(1) Be of a duration consistent with the grantee's academic calendar; and

(2) Provide sufficient opportunities for each student to gain in-depth experience in an area related to his or her academic or career goals.

(20 U.S.C. 1133a)

§ 632.51 Assessment of a student's progress.

During a student's work experience, a grantee shall assess the student's progress to ensure that the work experience satisfies the objectives of the student and the Cooperative Education Program.

(20 U.S.C. 1221e-3(a)(1), 1133a)

§ 632.52 In-service training.

A grantee shall ensure that the staff of its Cooperative Education project has an opportunity to participate in training that will enable the staff to—

(a) Operate the project more effectively; and

(b) Develop the expertise to expand and continue the Cooperative Education project.

(20 U.S.C. 1221e-3(a)(1), 1133a)

The Secretary revises Part 633 of Title 34 of the Code of Federal Regulations to read as follows:

PART 633—COOPERATIVE EDUCATION PROGRAM—DEMONSTRATION AND EXPLORATION PROJECTS

Subpart A—General

Sec.
633.1 The Cooperative Education Program—Demonstration and Exploration Projects.
633.2 Eligible applicants.
633.3 Eligible students.

Subpart B—Projects the Secretary Assists Under This Program

633.10 Eligible projects.

Subpart C—[Reserved]

Subpart D—How the Secretary Makes a Grant

633.30 Selection criteria the Secretary uses for demonstration projects.
633.31 Selection criteria the Secretary uses for exploration projects.

Subpart E—Conditions That Must Be Met by a Grantee

633.40 Status of project director.

Subpart F—Administrative Responsibilities of a Grantee

633.50 Administrative requirements.

Authority: Title VIII of the Higher Education Act of 1965, as amended (20 U.S.C. 1133, 1133b), unless otherwise noted.

Subpart A—General

§ 633.1 The Cooperative Education Program—Demonstration and Exploration Projects.

Demonstration and exploration projects are described in 34 CFR 631.12.

(20 U.S.C. 1221e-3(a)(1), 1133b)

§ 633.2 Eligible applicants.

The following are eligible to apply for grants under this part:

(a) Institutions of higher education.

(b) Combinations of institutions of higher education.

(20 U.S.C. 1133b)

§ 633.3 Eligible students.

To participate in a Cooperative Education project funded under this part, an individual must meet the definition of the term "student" in 34 CFR 631.3(b).

(20 U.S.C. 1221e-3(a)(1), 1133b)

Subpart B—Projects the Secretary Assists Under This Program

§ 633.10 Eligible projects.

Demonstration and exploration projects are described in 34 CFR 631.12.

(20 U.S.C. 1133b)

Subpart C [Reserved]

Subpart D—How the Secretary Makes a Grant

§ 633.30 Selection criteria the Secretary uses for demonstration projects.

The Secretary uses the following selection criteria in evaluating applications for demonstration grants under this part:

(a) *Plan of operation.* (10 points)

(1) The Secretary reviews each application for information that shows the quality of the plan of operation for the project.

(2) The Secretary looks for information that shows—

(i) High quality in the design of the project;

(ii) An effective plan of management that ensures proper and efficient administration of the project;

(iii) A clear description of how the objectives of the project relate to the purpose of the program;

(iv) The way the applicant plans to use its resources and personnel to achieve each objective; and

(v) A clear description of how the applicant will provide equal access and treatment for eligible project participants who are members of groups that have been traditionally under-represented, such as—

(A) Members of racial or ethnic minority groups;

(B) Women;

(C) Handicapped persons; and

(D) The elderly.

(b) *Planning for a demonstration project.* (6 points). The Secretary reviews each application for information that shows the extent to which the applicant has conducted the necessary initial planning for the project.

(c) *Integration of the demonstration project.* (25 points)

(1) The Secretary reviews each application for information that shows the extent of the action taken, or to be taken, by administrators and faculty to integrate the project into the academic policies, programs, and objectives of the institution.

(2) The Secretary looks for information that shows the extent to which the schedule for incorporating the project into the academic policies, programs, and objectives of the institution, including the rationale and procedures associated with each activity, will result in changes, if any in—

(i) The organizational structure of the applicant;

(ii) The curricula to meet the needs of students participating in Cooperative Education;

(iii) The academic calendar;

(iv) The awarding of credits;

(v) Increased faculty involvement;

(vi) Increased employer involvement;

(vii) Counseling techniques; and

(viii) Supervision of students during their work experiences.

(d) *Quality of key personnel.* (7 points)

(1) The Secretary reviews each application for information that shows the quality of the key personnel the applicant plans to use on the project.

(2) The Secretary looks for information that shows—

(i) The qualifications of the project director or principal investigator;

(ii) The qualifications of each of the other key personnel to be used in the project;

(iii) The time that each person referred to in paragraphs (b)(2) (i) and (ii) of this section will commit to the project; and

(iv) The extent to which the applicant, as part of its nondiscriminatory employment practices, encourages applications for employment from persons who are members of groups that have been traditionally underrepresented, such as members of racial or ethnic minority groups, women, handicapped persons, and the elderly.

(3) To determine personnel qualifications, the Secretary considers evidence of past experience and training, in fields related to the objectives of the project, as well as other information that the applicant provides.

(e) *Budget and cost effectiveness.* (5 points)

(1) The Secretary reviews each application for information that shows that the project has an adequate budget and is cost effective.

(2) The Secretary looks for information that shows—

(i) The budget for the project is adequate to support the project activities; and

(ii) Costs are reasonable in relation to the objectives of the project.

(f) *Evaluation plan.* (5 points)

(1) The Secretary reviews each application for information that shows the quality of the evaluation plan for the project. (See 34 CFR 75.590—Evaluation by the grantee.)

(2) The Secretary looks for information that shows methods of evaluation that are appropriate for the project and, to the extent possible, are objective and produce data that are quantifiable.

(g) *Adequacy of resources.* (3 points)

(1) The Secretary reviews each application for information that shows that the applicant plans to devote adequate resources to the project.

(2) The Secretary looks for information that shows—

(i) The facilities that the applicant plans to use are adequate; and

(ii) The equipment and supplies that the applicant plans to use are adequate.

(h) *Continuing support.* (15 points)

(1) The Secretary reviews each application for information that shows the applicant will continue the comprehensive Cooperative Education project after Federal financial assistance ends.

(2) The Secretary looks for information that shows—

(i) The amount of funds and resources (other than personnel) that will be committed to provide Cooperative Education in all or nearly all academic departments or disciplines in the institution; and

(ii) The extent to which the faculty and other staff will provide academic and career counseling to increase the paticipation in Cooperative Education of students in all or nearly all academic departments or disciplines.

(i) *Employer support.* (10 points)

(1) The Secretary reviews each application for information that shows employer support for the project.

(2) The Secretary looks for information that shows commitments by employers to—

(i) Maintain or increase the number of students they accept for work experiences; and

(ii) Accept students from a variety of academic departments or disciplines.

(j) *Commitment to Cooperative Education.* (4 points) The Secretary reviews each application for evidence that shows the extent to which the institution has developed a written philosophy of Cooperative Education that—

(1) Is reflected in the applicant's mission statement (that is, a broad statement of the applicant's fundamental purpose); and

(2) Clearly supports the expansion of Cooperative Education throughout the institution as described in 34 CFR 631.12.

(k) *Work experiences.* (10 points)

(1) The Secretary reviews each application for information that describes the work experiences that will be provided by the project.

(2) The Secretary looks for information that shows—

(i) The extent to which the applicant has arranged for a sufficient number of employment opportunities that are related to the educational, professional, or occupational objectives of students involved or to be involved in the project;

(ii) That the number, frequency, length, and quality of the work experiences are sufficient to make a significant contribution toward meeting the educational, professional, or occupational objectives of the students involved or to be involved in the project; and

(iii) The extent to which the applicant will develop, promote, and monitor close relationships between the students' work experiences and their academic studies.

(20 U.S.C. 1221e-3(a)(1), 1133(b))

§ 633.31 Selection criteria the Secretary uses for exploration projects.

The Secretary uses the following criteria in evaluating applications for exploration grants under this part.

(a) *Plan of operation.* (10 points)

(1) The Secretary reviews each application for information that shows the quality of the plan of operation for the project.

(2) The Secretary looks for information that shows—

(i) High quality in the design of the project;

(ii) An effective plan of management that ensures proper and efficient administration of the project;

(iii) A clear description of how the objectives of the project relate to the purpose of the program;

(iv) The way the applicant plans to use its resources and personnel to achieve each objective; and

(v) A clear description of how the applicant will provide equal access and treatment for eligible project participants who are members of groups that have been traditionally under-represented, such as—

(A) Members of racial or ethnic minority groups;

(B) Women;

(C) Handicapped persons; and

(D) The elderly.

(b) *Planning for an exploration project.* (10 points) The Secretary reviews each application for information that shows the extent to which the applicant has conducted the necessary initial planning for the project, including the extent to which the plan for implementing the project has been accepted by the —

(1) Responsible officials of the institution;

(2) Faculty;

(3) Employers; and

(4) Students.

(c) *Quality of key personnel.* (7 points)

(1) The Secretary reviews each application for information that shows the quality of the key personnel the applicant plans to use on the project.

(2) The Secretary looks for information that shows—

(i) The qualification of the project director or principal investigator;

(ii) The qualifications of each of the other key personnel to be used in the project;

(iii) The time that each person referred to in paragraphs (b)(2)(i) and (ii) of this section will commit to the project; and

(iv) The extent to which the applicant, as part of its nondiscriminatory employment practices, encourages applications for employment from

persons who are members of groups that have been traditionally underrepresented, such as members of racial or ethnic minority groups, women, handicapped persons, and the elderly.

(3) To determine pesonnel qualifications, the Secretary considers evidence of past experience and training, in fields related to the objectives of the project, as well as other information that the applicant provides.

(d) *Budget and cost effectiveness.* (5 points)

(1) The Secretary reviews each application for information that shows that the project has an adequate budget and is cost effective.

(2) The Secretary looks for information that shows—

(i) The budget for the project is adequate to support the project activities; and

(ii) Costs are reasonable in relation to the objectives of the project.

(e) *Evaluation plan.* (5 points)

(1) The Secretary reviews each application for information that shows the quality of the evaluation plan for the project. (See 34 CFR 75.590—Evaluation by the grantee.)

(2) The Secretary looks for information that shows methods of evaluation that are appropriate for the project and, to the extent possible, are objective and produce data that are quantifiable.

(f) *Adequacy of resources.* (5 points)

(1) The Secretary reviews each application for information that shows that the applicant plans to devote adequate resources to the project.

(2) The Secretary looks for information that shows—

(i) The facilities that the applicant plans to use are adequate; and

(ii) The equipment and supplies that the applicant plans to use are adequate.

(g) *Need for the exploration project.* (20 points) The Secretary reviews each application for a substantive explanation of the need for the exploration project.

(h) *Innovation.* (15 points) The Secretary reviews each application to determine the extent to which the applicant demonstrates an innovative or unique approach to Cooperative Education and relevance of the exploration project to institutions, students, and employers involved or interested in Cooperative Education.

(i) *Employer support.* (13 points) The Secretary reviews each application for evidence that shows the extent of employer support for the development of the exploration project.

(j) *Work experiences.* (10 points)

(1) The Secretary reviews each application for information that describes the work experiences that will be provided by the project.

(2) The Secretary looks for information that shows—

(i) The extent to which the applicant has arranged for a sufficient number of employment opportunities that are related to the educational, professional or occupational objectives of students involved or to be involved in the project;

(ii) That the number, frequency, length, and quality of the work experiences are sufficient to make a significant contribution toward meeting the educational, professional, or occupational objectives of the students involved or to be involved in the project; and

(iii) The extent to which the applicant will develop, promote, and monitor close relationships between the students' work experiences and their academic studies.

(20 U.S.C. 1221e-3(a)(1), 1133b)

Subpart E—Conditions That Must Be Met by a Grantee

§ 633.40 Status of project director.

In addition to the requirements of 34 CFR 75.510, the director of a demonstration project shall be full-time. The Secretary, however, may waive the full-time requirement, as specified in 34 CFR 75.511.

(20 U.S.C. 1221e-3(a)(1), 1133b)

Subpart F—Administrative Responsibilities of a Grantee

§ 633.50 Administrative requirements.

A grantee under this part shall meet all requirements in 34 CFR 632.50 through 632.52.

(20 U.S.C. 1221e-3(a)(1), 1133b)

The Secretary revises Part 634 of Title 34 of the Code of Federal Regulations to read as follows:

PART 634—COOPERATIVE EDUCATION PROGRAM—RESEARCH PROJECTS

Subpart A—General

Sec.
634.1 The Cooperative Education Program—Research Projects.
634.2 Eligible applicants.

Subpart B—Projects the Secretary Assists Under This Program

634.10 Eligible projects.

Subpart C—[Reserved]

Subpart D—How the Secretary Makes a Grant

634.30 Selection criteria the Secretary uses.

Sec.
634.31 Priority areas for research.

Subpart E—[Reserved]

Subpart F—[Reserved]

Authority: Title VIII of the Higher Education Act of 1965, as amended (20 U.S.C. 1133, 1133b), unless otherwise noted.

Subpart A—General

§ 634.1 The cooperative education program—research projects.

Research projects are described in 34 CFR 631.13.

(20 U.S.C. 1133b)

§ 634.2 Eligible applicants.

The following are eligible to apply for grants under this part:

(a) Institutions of higher education.

(b) Combinations of institutions of higher education.

(c) Public or private nonprofit agencies or organizations.

(20 U.S.C. 1133b)

Subpart B—Projects the Secretary Assists Under This Program

§ 634.10 Eligible projects.

The Secretary makes awards under this part for research projects dealing with, but not limited to, the following:

(a) Improving the operation of the Cooperative Education Program;

(b) Promoting the use of Cooperative Education as an alternative educational approach to assist students to prepare for careers and to finance their educational pursuits; and

(c) Developing better cooperation among high schools, institutions of higher education, business, and industry to enhance the opportunity for students to participate in work experiences related to their academic or career objectives.

(20 U.S.C. 1133b)

Subpart C—[Reserved]

Subpart D—How the Secretary Makes a Grant

§ 634.30 Selection criteria the Secretary uses.

The Secretary uses the following selection criteria in evaluating applications for grants under this part:

(a) *Plan of operation.* (20 points)

(1) The Secretary reviews each application for information that shows the quality of the plan of operation for the project.

(2) The Secretary looks for information that shows—

(i) High quality in the design of the project;

(ii) An effective plan of management that ensures proper and efficient administration of the project;

(iii) A clear description of how the objectives of the project relate to the purpose of the program;

(iv) The way the applicant plans to use its resources and personnel to achieve each objective; and

(v) A clear description of how the applicant will provide equal access and treatment for eligible project participants who are members of groups that have been traditionally underrepresented, such as—

(A) Members of racial or ethnic minority groups;

(B) Women;

(C) Handicapped persons; and

(D) The elderly.

(b) *Quality of key personnel.* (20 points)

(1) The Secretary reviews each application for information that shows the quality of the key personnel the applicant plans to use on the project.

(2) The Secretary looks for information that shows—

(i) The qualifications of the project director or principal investigator;

(ii) The qualifications of each of the other key personnel to be used in the project;

(iii) The time that each person referred to in paragraphs (b)(2)(i) and (ii) of this section will commit to the project; and

(iv) The extent to which the applicant, as part of its nondiscriminatory employment practices, encourages applications for employment from persons who are members of groups that have been traditionally underrepresented, such as members of racial or ethnic minority groups, women, handicapped persons, and the elderly.

(3) To determine personnel qualifications, the Secretary considers evidence of past experience and training, in fields related to the objectives of the project, as well as other information that the applicant provides.

(c) *Budget and cost effectiveness.* (7 points)

(1) The Secretary reviews each application for information that shows that the project has an adequate budget and is cost effective.

(2) The Secretary looks for information that shows—

(i) The budget for the project is adequate to support the project activities; and

(ii) Costs are reasonable in relation to the objectives of the project.

(d) *Evaluation plan.* (5 points)

(1) The Secretary reviews each application for information that shows the quality of the evaluation plan for the project. (See 34 CFR 75.590—Evaluation by the grantee.)

(2) The Secretary looks for information that shows methods of evaluation that are appropriate for the project and, to the extent possible, are objective and produce data that are quantifiable.

(e) *Adequacy of resources.* (3 points)

(1) The Secretary reviews each application for information that shows that the applicant plans to devote adequate resources to the project.

(2) The Secretary looks for information that shows—

(i) The facilities that the applicant plans to use are adequate; and

(ii) The equipment and supplies that the applicant plans to use are adequate.

(f) *Relevancy of research.* (20 points) The Secretary reviews each application for evidence that shows—

(1) The extent to which the applicant demonstrates that the proposed research is responsive to a major problem or need in Cooperative Education; and

(2) The extent to which the findings would be of value to institutions, students, or employers involved or interested in Cooperative Education.

(g) *Design of research.* (25 points) The Secretary reviews each application for evidence that shows the quality of the research design. The Secretary looks for—

(1) A clear description of the problem at which the research is directed;

(2) A well-defined hypothesis, if applicable;

(3) A clear description of research methods;

(4) The sampling method to be used, if applicable, including the size and proportion of the sample population to the universe of the population being studied; and

(5) A well-defined and organized plan for analyzing data.

(20 U.S.C. 1221e-3(a)(1), 1133b)

§ 634.31 Priority areas for research.

(a) The Secretary may, from time to time, select for priority funding research into one or more of the following:

(1) Identification and assessment of common elements in quality Cooperative Education projects.

(2) Measurement of factors influencing an institution of higher education to continue or not continue Cooperative Education after Federal financial assistance has ended.

(3) Assessment of the impact of factors that hinder or enhance—

(i) Student participation;

(ii) Faculty participation; or

(iii) Employer participation.

(4) Costs in relation to benefits of Cooperative Education, by identifying and assessing various methods of financing Cooperative Education.

(5) Longitudinal studies on former Cooperative Education students to determine the correlation between the students' Cooperative Education work experiences and one or more of the following:

(i) Initial job placement.

(ii) Job satisfaction.

(iii) Job advancement.

(6) Assessment of the impact of Cooperative Education on the retention and academic achievement of students participating in Cooperative Education.

(7) Assessment of the impact of a comprehensive Cooperative Education project on—

(i) Student at the institution;

(ii) Faculty;

(iii) Community resources; or

(iv) Employment opportunities.

(b) The Secretary announces subjects for priority funding, if any, in the annual application notice published in the **Federal Register.**

(20 U.S.C. 1221e-3(a)(1), 1133b)

Subpart E—[Reserved]

Subpart F—[Reserved]

The Secretary revises Part 635 of Title 34 of the Code of Federal Regulations to read as follows:

PART 635—COOPERATIVE EDUCATION PROGRAM—TRAINING PROJECTS

Subpart A—General

Sec.
635.1 The Cooperative Education Program—Training Projects.
635.2 Eligible applicants.
635.3 Eligible participants.

Subpart B—Projects the Secretary Assists Under This Program

635.10 Eligible projects.

Subpart C—[Reserved]

Subpart D—How the Secretary Makes a Grant

635.30 Selection criteria the Secretary uses.
635.31 Priorities for funding.
635.32 Geographic distribution.

Subpart E—[Reserved]

Subpart F—Administrative Responsibilities of a Grantee

635.50 Announcements of training sessions.

Authority: Title VIII of the Higher Education Act of 1965, as amended (20 U.S.C. 1133, 1133b), unless otherwise noted.

Subpart A—General

§ 635.1 The Cooperative Education Program—Training Projects.

Training projects are described in 34 CFR 631.14.

(20 U.S.C. 1133b)

§ 635.2 Eligible applicants.

The following are eligible to apply for grants under this part:

(a) Institutions of higher education.

(b) Combinations of institutions of higher education.

(c) Public or private nonprofit agencies or organizations.

(20 U.S.C. 1133b)

§ 635.3 Eligible participants.

Individuals with a need for training in the planning, establishment, administration, or coordination of programs of Cooperative Education are eligible to participate in training assisted under this part, including—

(a) Presidents and administrators of institutions of higher education, whether or not an institution administers a Federally funded Cooperative Education project.

(b) Faculty and staff of institutions of higher education, whether or not an institution administers a Federally funded Cooperative Education project.

(c) High school personnel responsible for career and academic guidance.

(d) Employers or prospective employers of students in a Cooperative Education project.

(e) Members of professional and scholarly organizations that participate or have an interest in Cooperative Education.

(20 U.S.C. 1133b)

Subpart B—Projects the Secretary Assists Under This Program

§ 635.10 Eligible projects.

(a) The Secretary makes awards under this part for training projects designed to develop skills necessary to administer Cooperative Education projects.

(b) Training projects may focus on, but are not limited to, the following:

(1) Responsibilities of directors or coordinators in administering Cooperative Education projects.

(2) Modification of teaching practices, academic calendars, and curricula.

(3) Relationships between work experiences and students' academic programs.

(4) Roles and responsibilities of employers and supervisors of students in Cooperative Education projects.

(5) Integration of Cooperative Education project into the academic programs and policies of institution of higher education.

(6) Placement, supervision, and evaluation of students in Cooperative Education work experiences.

(7) Placement and supervision of special student populations in work experiences under Cooperative Education projects.

(8) Cooperative Education for graduate students.

(9) Effective communications and relationships with regard to Cooperative Education within institutions, agencies, or organizations assisted under the Cooperative Education Program.

(10) Improving employer participation in Cooperative Education.

(11) Improving relations between institutions and the community with regard to Cooperative Education.

(12) Improving faculty attitudes toward and participation in Cooperative Education.

(13) Development of large-scale or comprehensive Cooperative Education projects.

(14) Planning, management, and evaluation of Cooperative Education projects.

(15) Improving the administration of Cooperative Education projects through the use of computers or other types of information systems.

(20 U.S.C. 1133b)

Subpart C—[Reserved]

Subpart D—How the Secretary Makes a Grant

§ 635.30 Selection criteria the Secretary uses.

The Secretary uses the following selection criteria in evaluating applications for grants under this part:

(a) *Plan of operation.* (10 points)

(1) The Secretary reviews each application for information that shows the quality of the plan of operation for the project.

(2) The Secretary looks for information that shows—

(i) High quality in the design of the project;

(ii) An effective plan of management that ensures proper and efficient administration of the project;

(iii) A clear description of how the objectives of the project relate to the purpose of the program;

(iv) The way the applicant plans to use its resources and personnel to achieve each objective; and

(v) A clear description of how the applicant will provide equal access and treatment for eligible project participants who are members of groups that have been traditionally underrepresented, such as—

(A) Members of racial or ethnic minority groups;

(B) Women;

(C) Handicapped persons; and

(D) The elderly.

(b) *Needs assessment.* (20 points) The Secretary reviews each application for information that shows the extent to which the applicant provides evidence of a need for—

(1) Training in its geographic area; or

(2) Training of a specialized nature addressed to a nationwide clientele.

(c) *Thoroughness of training design.* (10 points) The Secretary reviews each application for information that shows the extent to which the applicant provides evidence of thorough planning for the proposed project, including the procedures used in developing the design.

(d) *Scope of training.* (15 points) The Secretary reviews each application for evidence that shows the extent to which the proposed project will address the needs of the constituency selected to receive training, based on the use of needs analysis data.

(e) *Anticipated results.* (10 points) The Secretary reviews each application for evidence that shows the extent to which the proposed project has promise of fulfilling the proposed objectives identified in the needs assessment, training design, and scope of training.

(f) *Quality of key personnel.* (15 points)

(1) The Secretary reviews each application for information that shows the quality of the key personnel the applicant plans to use on the project.

(2) The Secretary looks for information that shows—

(i) The qualifications of the project director or principal investigator;

(ii) The qualifications of each of the other key personnel to be used in the project;

(iii) The time that each person referred to in paragraphs (b)(2) (i) and (ii) of this section will commit to the project; and

(iv) The extent to which the applicant, as part of its nondiscriminatory employment practices, encourages applications for employment from persons who are members of groups that have been traditionally underrepresented, such as members of racial or ethnic minority groups, women, handicapped persons, and the elderly.

(3) To determine personnel qualifications, the Secretary considers evidence of past experience and training, in fields related to the objectives of the project, as well as

other information that the applicant provides.

(g) *Budget and cost effectiveness.* (5 points)

(1) The Secretary reviews each application for information that shows that the project has an adequate budget and is cost effective.

(2) The Secretary looks for information that shows—

(i) The budget for the project is adequate to support the project activities; and

(ii) Costs are reasonable in relation to the objectives of the project.

(h) *Evaluation plan.* (10 points)

(1) The Secretary reviews each application for information that shows the quality of the evaluation plan for the project. (See 34 CFR 75.590—Evaluation by the grantee.)

(2) The Secretary looks for information that shows methods of evaluation that are appropriate for the project and, to the extent possible, are objective and produce data that are quantifiable.

(i) *Adequacy of resources.* (5 points)

(1) The Secretary reviews each application for information that shows that the applicant plans to devote adequate resources to the project.

(2) The Secretary looks for information that shows—

(i) The facilities that the applicant plans to use are adequate; and

(ii) The equipment and supplies that the applicant plans to use are adequate.

(20 U.S.C. 1221e-3(a)(1), 1133b)

§ 635.31 Priorities for funding.

(a) The Secretary may, from time to time, select for priority funding training projects that focus on one or more of the subjects in paragraph (b) of this section. Each of the items in paragraph (b) incorporates one or more of the eligible projects listed in § 635.10.

(b) The Secretary may select one or more of the following as a funding priority:

(1) The development of appropriate skills to manage Cooperative Education projects effectively.

(2) The development of Cooperative Education demonstration and exploration projects.

(3) The development of student, faculty, and employer receptivity to Cooperative Education.

(4) The integration of programs of Cooperative Education into the academic programs and policies of institutions.

(5) The development of Cooperative Education trainers.

(6) The determination of costs and potential income benefits of Cooperative Education for various constituents of the Cooperative Education community.

(c) The Secretary announces subjects for priority funding, if any, in the annual application notice published in the Federal Register.

(20 U.S.C. 1221e-3(a)(1), 1133b)

§ 635.32 Geographic distribution.

Among applications of relatively equal quality, the Secretary may give priority to applications that ensure a wide geographic distribution throughout the United States of training projects funded under this part.

(20 U.S.C. 1221e-3(a)(1), 1133b)

Subpart E—[Reserved]

Subpart F—Administrative Responsibilities of a Grantee

§ 635.50 Announcements of training sessions.

All announcements inviting participants to training sessions must contain information that will fully describe eligibility requirements for participants, activities, anticipated results or skills to be acquired, and registration or other fees.

(20 U.S.C. 1221e-3(a)(1), 1133b)

[FR Doc. 82-10862 Filed 4-20-82; 8:45 am]
BILLING CODE 4000-01-M

federal register

Wednesday
April 21, 1982

Part VIII

Federal Deposit Insurance Corporation

Unsafe and Unsound Banking Practices; Request for Comments on Proposed Accrual Accounting Rule

FEDERAL DEPOSIT INSURANCE CORPORATION

12 CFR Part 337

Unsafe and Unsound Banking Practices; Request for Comment on Proposed Accrual Accounting Rule

AGENCY: Federal Deposit Insurance Corporation.

ACTION: Proposed Rule

SUMMARY: This proposed rule reflects the judgment of the Corporation in response to a recommendation by the Federal Financial Institutions Examination Council that all federally supervised commercial and State-chartered mutual savings banks be required to maintain their books and records of account on the accrual basis of accounting. The effect of this rule would be to improve the quality of financial data on banks.

DATE: Comments on this proposed regulation must be received on or before June 21, 1982.

ADDRESS: Comments should be sent to the Executive Secretary, Federal Deposit Insurance Corporation, 550 17th Street, NW., Washington, D.C. 20429, or delivered to Room 6108 at the same address between 9 a.m. and 5 p.m. on business days. Comments received may be inspected at Room 6108 between 9 a.m. and 4:15 p.m. on business days.

FOR FURTHER INFORMATION CONTACT: Felicite Macfarlane, Planning and Program Development Specialist, Federal Deposit Insurance Corporation, Washington, D.C. 20429, telephone 202/ 389-4141.

SUPPLEMENTARY INFORMATION: The proposal under consideration is based on concern that accrual accounting is pertinent and necessary to the accuracy of financial reports used for internal management, shareholder, and other public disclosure and regulatory purposes.

If the proposal is adopted, all insured State-chartered nonmember commercial and mutual savings banks would have to maintain their books and records of account on the accrual basis of accounting. This requirement would become effective as of January 1, 1983 for all such banks with assets of more than $10 million as of December 31, 1981. As of January 1, 1985 the requirement would apply to all such banks regardless of size.

A substantially similar proposed rule is being published contemporaneously by the Office of the Comptroller of the Currency ("OCC").

Both the Federal Deposit Insurance Corporation ("FDIC") and the OCC favor greater deregulation of the banking industry. Regulations which unduly restrict bank managements' ability to serve the banking needs of the public should be eliminated. For a bank to prosper in a less regulated environment, its management must have sufficient information to make appropriate choices on matters in which it had no choices prior to deregulation. An accurate financial picture of the bank's condition and results of operations is vital. Thus, although the imposition of accrual accounting may increase the operating expenses of a bank not presently using accrual accounting, such imposition is a necessary precondition for the bank regulatory agencies and the banking industry to implement a comprehensive but prudent course toward significant deregulation of banking.

On August 11, 1981, the Federal Financial Institutions Examination Council ("Council") issued a **Federal Register** Notice requesting comment on a then proposed accrual accounting guideline. The proposed guideline would have required the use of accrual accounting by all insured commercial and State-chartered mutual savings banks, both for maintenance of accounts and for reports submitted to the three Federal bank regulatory agencies (the Board of Governors of the Federal Reserve System, the Federal Deposit Insurance Corporation and the Office of the Comptroller of the Currency).

Upon consideration of the comment letters and the issues involved the Council, on November 5, 1981, acted to extend to all banks the current accrual reporting requirement for banks with assets of more than $25 million. The accrual reporting requirement is effective January 1, 1983, for banks having assets of more than $10 million. As of January 1, 1985, the requirement would apply to all banks regardless of size. This Council action requires all insured commercial and State-chartered mutual savings banks to prepare their Reports of Condition and Income, as well as any other reports that may be filed with the three Federal bank regulatory agencies, on an accrual accounting basis.

In a separate action, also taken on November 5, 1981, the Council voted to recommend that the three Federal bank regulatory agencies represented on the Council issue accounting guidelines that would require all federally supervised banks to maintain their books and records on an accrual basis. The Council recommended that such an accounting requirement become effective concurrently with the aforementioned reporting requirement. This proposed rule reflects the Council's recommendation. Rather than adopting the Council's recommended "guideline," the Corporation determined to address this matter as a proposed rule because a rule is more clearly enforceable.

There are several excellent publications that are available to assist bank maintenance of an accrual basis accounting system. For example, *A Financial Information System for Community Banks: Part 3, Accrual Accounting*, published by the Bank Administration Institute, furnishes detailed guidelines and methods useful in converting to an accural system.

The Reports of Condition and Income that are filed with the FDIC by insured State nonmember commercial and mutual savings banks provide information that is essential to the effective discharge of supervisory responsibilities. Accrual accounting requires recognition of revenues and expenses in the periods to which they are attributable. Under a cash basis accounting system, revenues and expenses are recorded when cash is received or disbursed. In cases in which cash flows associated with assets and liabilities do not occur regularly over the life of an item that spans more than one accounting period, significant distortions may result from use of a cash basis. The previously permitted modified cash basis of reporting by smaller banks no longer statisfactorily portrays a bank's financial position and results of operations.

In the past when interest rates were lower and less volatile, banks with stable patterns of operations were likely to have reported similar results of operation using either accounting method. However, in the current environment of rapidly changing banking practices, portfolio patterns and interest rates, cash basis operating results may differ significantly from accrual basis results. The loss of traditional low cost deposits and increased competition have forced banks to offer new higher cost deposit instruments and services. Cash basis financial information does not reflect the effects of such changes in a timely manner. Conversely, accrual accounting, by recording the effects of such changes as they occur, provides a more realistic portrayal of an institution's performance and condition, and is more pertinent to the needs of management, shareholders and supervisors.

Banks that currently maintain their books on a modified cash basis would be encouraged to convert to an accrual

system prior to the dates specified above.

This proposed rule would require the establishment of recording procedures whereby all material (1) adjustments to balance sheet accounts and (2) adjustments to items of income and expense are consistently recognized in the periods in which they are earned or incurred.

The following is a listing of major accounts that normally would be maintained on an accrual basis:

1. Interest on loans;
2. Interest on investment securities (for example, to reflect premium amortization and discount accretion);
3. Fixed asset and depreciation expense;
4. Loan loss allowance and expense provision for loan losses;
5. Lease income;
6. Interest expense on deposits (including savings accounts and certificates of deposit);
7. Interest expense on all obligations for the purpose of borrowing money; and
8. Estimated provisions for applicable income taxes (including both currently payable and deferred income taxes).

Trust department income as well as immaterial items of income and expense may be recorded on the cash basis as long as the results do not differ significantly from those that would be obtained from the accrual basis of accounting.

Cost and Benefits of the Proposal

The legal authority for this proposed rule is section 9 of the Federal Deposit Insurance Act (12 U.S.C. 1819). Requiring smaller banks to adopt accrual accounting would impose some burden on affected institutions. In an effort to minimize the immediate impact of that burden, the Corporation contemplates a fairly long lead time for the implementation of the proposed rule. The smallest of the affected banks, those with assets of $10 million or less, would be given more than two full calendar years to plan for implementation.

According to a March 1981 Federal Financial Institutions Examination Council survey, of 5,003 nonmember banks with assets of less than $25 million, approximately 2,600 were reported on a modified cash basis. Of these, about 1,500 banks have assets of $10 to $25 million and 1,100 have assets less than $10 million. Because the survey was taken a year ago and because most new banks adopt accrual accounting, these estimates may overstate slightly the number of banks reporting on a modified cash basis.

NUMBER OF INSURED STATE NONMEMBER COMMERCIAL BANKS WITH ASSETS OF LESS THAN $25 MILLION; ANALYSIS AS TO ACCRUAL OR MODIFIED CASH BASIS REPORTING SURVEY ESTIMATES

Asset size	Accrual		Modified cash		Total
Under $10 million	704	38%	1,132	62%	1,836 100%
$10 to $25 million	1,706	54%	1,461	46%	3,167 100%
Total	2,410	48%	2,593	52%	5,003 100%

The survey also endeavored to ascertain the costs associated with conversion to and maintenance of an accrual accounting system. The survey results indicated a median cost to convert from a modified cash to an accrual basis of approximately $2,000 per bank plus additional operating costs of $1,000 per year. Thus, the cost to the 2,600 affected banks would aggregate about $5 million, with additional operating expenses for those banks, aggregating $3 million per year.

Since virtually all new banks must adopt accrual accounting, it has been suggested that the continuing effects of inflation would eventually result in a disappearance of cash basis accounting in banks as bank footings grow. However, an analysis of the growth rates of banks in various size groups below $25 million has revealed that it would likely take seven years for the average bank which currently has assets between $10–$25 million to reach the $25 million threshold, and 16 years for the average bank which now has assets of less than $10 million to attain a size of $25 million.

The adoption of the proposed accrual accounting rule would assure certain benefits for the banking industry and the public. Bank management and directors would be able to evaluate their institution's current performance and plan for the future using financial data free from the distortions associated with cash basis accounting. Publicly available financial data from these institutions would be a more reliable source of information for shareholders and other interested parties. The regulatory agencies would benefit from better and more comparable data for supervisory purposes.

The costs of on-site examinations that are conducted would be reduced if such examinations could be targeted to concentrate on particular problem areas within the bank. The General Accounting Office has urged the bank regulatory agencies to develop systems and procedures to permit such concentrations of examination effort. Since accrual accounting would improve regulator confidence in monitoring systems, it would serve this objective.

The regulatory agencies' monitoring and surveillance systems fulfill a public responsibility to identify problem institutions in a timely and efficient manner. Again, to the extent that accrual accounting enhances monitoring systems, this public obligation is served.

The proposed rule, taken in connection with recent changes to require (after a transition period) universal accrual basis reporting, is an integral part of FDIC's efforts to implement a strategy permitting shorter and less frequent bank examinations. FDIC recognizes that accrual reporting and bookkeeping imposes an additional burden on banks and might necessitate professional accounting services. However, the reduction in burden that will result from shorter, less frequent bank examinations is expected to more than offset such short-term increase in burden to banks. Furthermore, most banks, other than the smaller institutions, are already in substantial compliance with the proposed rule.

As required by the Regulatory Flexibility Act and based upon the issues discussed above, the FDIC Board of Directors certifies that adoption of this proposal will not have a significant economic impact on a substantial number of small entities.

Alternative

As an alternative, the Corporation considered expanding the visitations by bank examiners to enable them to develop and obtain the information which would be captured under the proposed rule. This alternative was rejected due to the higher costs of an increased visitation schedule which, although initially borne by FDIC, are ultimately borne entirely by insured banks.

Issues for Specific Comment

The Corporation seeks comments on any and all aspects of this proposal. In any commentary, indicate asset size of bank discussed. Because cost estimates published by the Federal Financial Institutions Examination Council in its earlier related request for comments were questioned by bankers, we would

particularly appreciate comments, including specific facts, relating to the following points:

(1) Actual cost to banks that have converted to accrual basis books and records of account.

(2) The itemized expected cost of compliance with the proposed rule, including the cost to banks already having assets over $25 million which therefore already "report" on the accrual basis. State basis of estimated cost. Cover cost of increased staff or professional accounting service.

(3) Does this proposal allow affected banks sufficient time to effect necessary conversions? Explain.

(4) State banking supervisors' views on this proposed rule.

List of Subjects in 12 CFR Part 337

Accounting, Banks, banking.

PART 337—UNSAFE AND UNSOUND BANKING PRACTICES

For the reasons set forth in the preamble, Part 337 of Chapter III of Title 12 of the Code of Federal Regulations is proposed to be amended as follows:

1. The authority citation for Part 337 reads as follows:

Authority: Sec. 9, Pub. L. No. 797, 64 Stat. 881-882 (12 U.S.C. 1819).

2. In Part 337, § 337.4 is added to read as follows:

§ 337.4 Accrual accounting.

(a) *Purpose, scope, and effective dates.*—(1) *Purpose.* This section is issued to assure that financial data provided or available to Federal regulators, bank shareholders, customers, and the general public are timely, consistent, and fairly present the results of operations and financial condition of the bank.

(2) *Scope.* This section applies to all insured State nonmember banks (including mutual savings banks).

(3) *Effective dates.* This section is effective upon the following dates: January 1, 1983 for banks with assets of more than $10 million as of December 31, 1981; January 1, 1985 for all banks.

(b) *Accrual accounting requirement.* As of the effective dates specified in § 337.4(a), banks shall maintain their books and records on the accrual basis of accounting. Banks shall make all material accrual adjustments to assure that periodic revenues and expenses are matched for recording purposes to the specific periods of time, such as the day or month, in which they are earned or incurred, without regard to the actual date of receipt or payment of cash. The posting frequency for accruals shall be appropriate to (1) the specific transaction involved, (2) the volatility of the underlying asset or liability, and (3) the schedule for reporting for internal bank management; and, normally, frequency of posting will be at least monthly.

This requirement in no way prohibits banks from maintaining such cash basis records or other records as are necessary for tax or other purposes in accordance with Federal or other statutes or regulations.

By order of the Board of Directors, dated March 1, 1982.

Hoyle L. Robinson,

Executive Secretary.

[FR Doc. 82-10543 Filed 4-20-82; 8:45 am]

BILLING CODE 6714-01-M

federal register

Wednesday
April 21, 1982

Part IX

Department of the Interior

Office of Surface Mining Reclamation and Enforcement

State Regulatory Authority Inspection and Enforcement; Federal Inspection and Monitoring; Procedures and Criteria For Approval or Disapproval of State Program Submissions

DEPARTMENT OF THE INTERIOR

Office of Surface Mining Reclamation and Enforcement

30 CFR Parts 732, 840 and 843

State Regulatory Authority Inspection and Enforcement; Federal Inspection and Monitoring; Procedures and Criteria For Approval or Disapproval of State Program Submissions

AGENCY: Office of Surface Mining Reclamation and Enforcement, Interior.

ACTION: Notice of intent.

SUMMARY: The Office of Surface Mining (OSM) will soon issue final rules with respect to the changes proposed on December 1, 1981, to its inspection and enforcement regulations, and the changes proposed on December 4, 1981, to its State program approval criteria and procedures. However, OSM is hereby giving notice of its intent not to issue final rules with respect to two proposed changes concerning citizens' rights to sue in State courts and to participate in inspections resulting from citizens' requests. OSM also has decided to defer issuance of a final rule on the proposed change to the inspection and enforcement regulations regarding OSM's authority to take enforcement action in oversight.

Final rulemaking action on two issues raised as a result of a petition by several western States regarding attorneys' fees is also being deferred.

FOR FURTHER INFORMATION CONTACT: Peter J. Schaumberg, Assistant Solicitor, Branch of Regulatory Programs, Division of Surface Mining, Washington, D.C. 20240, telephone (202) 343-5207.

SUPPLEMENTARY INFORMATION:

Background

On December 1, 1981 (46 FR 58464), OSM issued a proposed revision to its rules in 30 CFR Parts 840, 842, 843, and 845 which set forth the standards and procedures governing State regulatory authority inspection and enforcement, Federal inspection and enforcement and civil penalty assessments. On December 4, 1981 (46 FR 59482), OSM published proposed changes to its rules in 30 CFR Parts 730, 731, 732, 733 and 736 concerning State and Federal programs. OSM soon will issue final rules which will adopt many of the proposed changes. However, OSM already has decided not to adopt some of the proposed changes and to defer final action on others. The purpose of this notice is to inform the public as soon as possible of OSM's determinations. The proposed changes affected by this notice are described below. Further details on the actions announced today will be provided in the preambles to the final State program criteria rule and to the final inspection and enforcement rule when they are issued. Responses to comments received on the two notices of proposed rulemaking, for which the comment periods closed on January 18, 1982, and February 1, 1982, respectively, will also be included in the preambles.

Citizens' Participation

Section 840.15 currently requires that State programs contain provisions for public participation in enforcement "consistent with 30 CFR Parts 842, 843, and 845 and 43 CFR Part 4." Section 732.15(b)(10) contains a similar provision, requiring that State programs contain provisions for public participation "consistent with the public participation requirements of the Act and this chapter." The **Federal Register** preamble to the existing provisions states that OSM interprets the regulation to require State programs to provide for citizens' suits in State courts and for citizens' rights to participate in inspections resulting from citizens' requests. See 44 FR 14965 and 15297 (March 13, 1979).

In the December 1, 1981, and December 4, 1981 proposals, OSM proposed to revise §§ 840.15 and 732.15(b)(10) so that State programs would not be required to provide for citizens' suits in State courts or for citizens' participation in inspections.

OSM has determined not to alter the finding set out at 44 FR 14965 and 44 FR 15297 that approved State programs must provide at least the same level of citizen participation, including provisions for citizens' suits and citizen access to mine sites, as do the Federal statute and regulations. Accordingly, OSM has decided to retain without change 30 CFR 840.15 and its preamble (44 FR 15297) and the citizens' rights provisions of 30 CFR 732.15 and its preamble (44 FR 14965). This includes retention of the existing requirement that State programs provide for the award of costs and attorneys' fees in citizens' suits brought under subsections 520(d) and (f) of the Surface Mining Control and Reclamation Act of 1977, 30 U.S.C. 1201 *et seq.* (the Act), subject to the unresolved question concerning States' possible sovereign immunity, described below.

Notices of Violation in Oversight

OSM proposed in the December 1, 1981, notice to amend § 843.12(a)(2) to reflect OSM's view that OSM may not have authority in certain situations to issue notices of violation. OSM has decided that the proposed revision to § 843.12(a)(2) will not be finalized at this time. Final action on this proposed regulation will be deferred until it has been examined in the Supplemental Environmental Impact Statement (EIS) which OSM is preparing to analyze fully environmental impacts resulting from OSM's regulatory reform effort. The comment period on the proposed change to § 843.12(a)(2) will be reopened to coincide with the comment period on the draft supplemental EIS.

Attorneys' Fees

OSM will defer final rulemaking action on two issues raised as a result of a petition filed by several western States on May 12, 1981 and November 21, 1981 until after a decision on the petition itself has been rendered. The issues pertain to the following: (1) those portions of the proposals (at 46 FR 58466 and at 46 FR 59486) that deal with the question of costs and expenses, including attorneys' fees, in proceedings provided under section 525(e) of the Act, and 43 CFR Part 4, and (2) the question of the States' invocation of sovereign immunity, by statute or otherwise, to protect the States against attorneys' fees awards in all proceedings, including those provided under subsections 520(d) and (f) of the Act.

In the December 1, 1981, notice, OSM discussed the petition submitted by the western States requesting the deletion of the requirement in § 840.15 that each State program provide for public participation consistent with 43 CFR 4.1294(b) which permits the award of appropriate costs and expenses, including attorneys' fees, from OSM to any person other than a permittee or his or her representative, if the person initiates or participates in any proceeding under the Act, upon a finding that the person made a substantial contribution to a full and fair determination of the issues. In that notice, OSM specifically requested comments as to whether the petition should be granted in whole or in part.

In OSM's view, the importance of the attorneys' fees issues specified above requires further study. Thus, these issues will not be resolved until after the issuance of the final rule addressing the other matters contained in the December 1, 1981, proposal. OSM will issue another notice of proposed rulemaking to solicit public comment on the attorneys' fee issues and the petition of the western States.

Dated: April 16, 1982.

Daniel N. Miller, Jr.,

Assistant Secretary, Energy and Minerals.

[FR Doc. 82-11006 Filed 4-20-82; 8:45 am]

BILLING CODE 4310-05-M

Reader Aids

Federal Register

Vol. 47, No. 77

Wednesday, April 21, 1982

INFORMATION AND ASSISTANCE

PUBLICATIONS

**Code of Federal Regulations**	
CFR Unit	202-523-3419
	523-3517
General information, index, and finding aids	523-5227
Incorporation by reference	523-4534
Printing schedules and pricing information	523-3419
**Federal Register**	
Corrections	523-5237
Daily Issue Unit	523-5237
General information, index, and finding aids	523-5227
Privacy Act	523-5237
Public Inspection Desk	523-5215
Scheduling of documents	523-3187
**Laws**	
Indexes	523-5282
Law numbers and dates	523-5282
	523-5266
Slip law orders (GPO)	275-3030
**Presidential Documents**	
Executive orders and proclamations	523-5233
Public Papers of the President	523-5235
Weekly Compilation of Presidential Documents	523-5235
**United States Government Manual**	523-5230

SERVICES

Agency services	523-4534
Automation	523-3408
Library	523-4986
Magnetic tapes of FR issues and CFR volumes (GPO)	275-2867
Public Inspection Desk	523-5215
Special Projects	523-4534
Subscription orders (GPO)	783-3238
Subscription problems (GPO)	275-3054
TTY for the deaf	523-5229

FEDERAL REGISTER PAGES AND DATES, APRIL

13757-14130.............................1
14131-14474.............................2
14475-14666.............................5
14667-14884.............................6
14885-15090.............................7
15091-15308.............................8
15309-15556.............................9
15557-15760.............................12
15761-16000.............................13
16001-16160.............................14
16161-16310.............................15
16311-16610.............................16
16611-16758.............................19
16759-17032.............................20
17033-17270.............................21

CFR PARTS AFFECTED DURING APRIL

At the end of each month, the Office of the Federal Register publishes separately a list of CFR Sections Affected (LSA), which lists parts and sections affected by documents published since the revision date of each title.

1 CFR

51.......................................15210

3 CFR

Proclamations:

4571 (Superseded by
  Proc. 4934)....................16767
4916.......................................14475
4917.......................................14477
4918.......................................14667
4919.......................................14669
4920.......................................14670
4921.......................................14673
4922.......................................14885
4923.......................................14887
4924.......................................14889
4925.......................................15091
4926.......................................15761
4927.......................................16001
4928.......................................16003
4929.......................................16313
4930.......................................16759
4931.......................................16761
4932.......................................16763
4933.......................................16765
4934.......................................16767

Executive Orders:

July 2, 1910
  (Revoked in part
  by PLO 6230)................14157
December 12, 1917
  (Revoked in part
  by PLO 6238)................17060
June 5, 1919
  (Revoked in part
  by PLO 6237)................16628
March 8, 1920
  (Revoked in part
  by PLO 6237)................16628
May 14, 1920
  (Revoked in part
  by PLO 6320)................14157
August 27, 1921
  (Revoked in part
  by PLO 6237)................16628
October 3, 1929
  (Revoked in part
  by PLO 6237)................16628
5407 (Revoked in part
  by PLO 6237)................16628
6019 (Revoked in part
  by PLO 6237)................16628
6277 (Revoked in part
  by PLO 6237)................16628
11896 (Amended by
  EO 12357)....................15093
12065 (Revoked by
  EO 12356,
  effective
  August 1, 1982)............14874
12336 (Amended by
  EO 12355)....................14479
12355.......................................14479
12356.......................................14874
12356 (correction)............15557
12357.......................................15093
12358.......................................16311

5 CFR

Ch. XIV.....................................16611
890...14868
1201.......................................15309

Proposed Rules:

412...16341
890...15996

7 CFR

1...15559
2...15559
210..........14131, 14134, 15978
215.......................14131, 14134
220.......................14131, 14134
225...15309
230...14134
235...14134
240...15978
245...14134
250...15978
253...14135
331...14891
418.......................14675, 17033
600...14683
729...15966
905...16005
907..........13757, 15095, 16161
908...15095
910..........14137, 15310, 16315
925...15095
985...16769
991.......................16770, 17034
1007.......................................16611
1032.......................................16315
1068.......................................16613
1093.......................................13757
1106.......................................17035
1139.......................................17036
1423.......................................15310
1425.......................................15763
1427.......................................15764
1468.......................................15096
1472.......................................16161
1900.......................................13758
2003.......................................15560

Proposed Rules:

210...15342
210...15342
246...15348
272...14160
273...14160
274...14160
284...15346
331...14915
413...14915
426...13826

1007.....14919
1822.....15589
1944.....15589

8 CFR

3.....16771

9 CFR

82.....16772
92.....15097

Proposed Rules:

78.....13827
151.....17068
166.....16534
319.....14168
381.....14168

10 CFR

Ch. II.....13767
2.....16005
20.....16162
50.....15569
60.....13774
72.....13774
81.....13774
417.....16166
455.....15765
500.....15311, 17037
501.....15311, 17037
503.....15311
504.....17037

Proposed Rules:

35.....15798
50.....15801
430.....14424
795.....14490

11 CFR

110.....15098

12 CFR

29.....13775
204.....14481
208.....14684
217.....14483, 16773, 16774
225.....14684
265.....16169
525.....16316
526.....13776
544.....13776, 16170
545.....13776
555.....13776
561.....13776
563.....13776
564.....13776
701.....16775
707.....16775
708.....16775
745.....16775
1204.....14690, 15098

Proposed Rules:

Ch. I.....16033
Ch. II.....13827
210.....15349
528.....16633
31.....17069
337.....17264
618.....13834

13 CFR

305.....15101

14 CFR

39.....13784-13788, 15102, 15569-15576, 16614-16617
67.....16298
71.....13789, 13790, 15103-15106, 15577-15579, 16170, 16171, 16618-16620
75.....16171
97.....14485, 16621
205.....16172
373.....16006
399.....14892
1204.....14893, 15767

Proposed Rules:

Ch. I.....14014
Ch. V.....14923
39.....15600
45.....14128
71.....13834, 13835, 15601, 16642, 16643
75.....15143
217.....15350
221.....15144
241.....15350
296.....15144
297.....15144
399.....16792, 16795

15 CFR

359.....14692
368.....15106
369.....15106
370.....15106, 16622
371.....14695, 15106
372.....14695, 15106
373.....15106
374.....15106
375.....15106
376.....15106
377.....15106
379.....15106
385.....15106, 16623
386.....15106, 16623
387.....15106
388.....15106
389.....15106
390.....15106
399.....16624
806.....14138, 15579

16 CFR

1306.....14366

Proposed Rules:

1405.....14420
1406.....14711
1500.....16041

17 CFR

140.....16173
240.....17046
249.....17046

Proposed Rules:

190.....16187
230.....16043
240.....16043
270.....16341

18 CFR

271.....15315-15317, 17054
357.....16317

Proposed Rules:

260.....16644
271.....14490-14492, 15353-15356, 15805, 15806, 17070
290.....13836

19 CFR

Proposed Rules:

Ch. I.....16033
123.....17072
134.....14493
201.....13791
210.....13791

20 CFR

Ch. VI.....14696
404.....13792, 14894
416.....13792, 15319
676.....15988

Proposed Rules:

404.....15602
416.....15602

21 CFR

5.....16010, 16318
73.....16319
74.....14138
81.....14137, 14138
82.....14138
106.....17016
176.....14697
177.....14697, 14697, 16776
178.....14700, 16319
193.....14894, 14895
430.....15767
436.....15767
440.....15767
444.....16320
452.....15326
510.....14148, 14700, 15327
520.....14148, 14149, 14701, 15327, 15328, 15770, 16776
522.....14148, 14149, 14702, 14703, 15327, 15328
524.....15771, 16320
526.....15772
540.....14148, 14150, 16320
556.....15770
558.....14148, 14149, 14151, 14700, 14703, 14704, 15773, 16320
561.....14896-14898, 16011
640.....15329
884.....14705

Proposed Rules:

Ch. I.....14464
168.....15357
357.....16796
874.....16796

22 CFR

42.....15773

23 CFR

625.....13794
626.....13794
1205.....15116
1252.....15116

Proposed Rules:

Ch. I.....14014
Ch. II.....14014

24 CFR

58.....15750
200.....14487
203.....16776
204.....16776
220.....16776
221.....16776
222.....16776
226.....16776
227.....16776
233.....16776
235.....16776
237.....16776
240.....16776
300.....17055
570.....15290
571.....16321

Proposed Rules:

201.....14712
203.....14495, 14713
204.....14713
213.....14713
220.....14713
221.....14713
222.....14713
226.....14713
227.....14713
233.....14713
234.....14713
235.....14713
237.....14713
240.....14713

25 CFR

700.....15774

Proposed Rules:

Ch. I.....16936
43d.....17072

26 CFR

1.....15122
5c.....15330

Proposed Rules:

Ch. I.....16033
1.....16797

27 CFR

Proposed Rules:

Ch. I.....16033

28 CFR

24.....15774

29 CFR

Subtitle A.....14696, 14706
Ch. XVII.....14696, 14706
1405.....15779
1915.....16984
1916.....16984
1917.....16984
2619.....15780
2645.....14899

Proposed Rules:

Ch. XIV.....15807
1910.....14169, 14716, 15358, 16348
1918a.....14716

30 CFR

Ch. I.....14696, 14706
251.....15781
916.....16012
936.....14152

Proposed Rules:

Ch. II.....16936
Ch. VII.....16936
221.....17076
250.....16349
701.....16152, 16592
715.....16349
716.....15605
732.....17268

780 ... 16349
784 ... 16604
785 ... 15605, 16152, 16604
816 ... 16152, 16349, 16592, 16604
817 ... 16152, 16349, 16592, 16604
818 ... 16604
824 ... 16152
826 ... 16152
840 ... 17268
843 ... 17268
850 ... 16349
901 ... 16797
910 ... 17240
913 ... 16046
917 ... 15605
920 ... 13836
931 ... 16188
936 ... 14170
938 ... 15368
943 ... 14170

31 CFR

Proposed Rules:
515 ... 17030
51 ... 16033

32 CFR

56 ... 15122
199 ... 16014, 16321
213 ... 14899
892 ... 16780

Proposed Rules:
505 ... 14925

33 CFR

3 ... 13796
81 ... 13798
84 ... 15135
85 ... 15135
86 ... 15135
87 ... 16173
88 ... 16174
89 ... 13800
100 ... 16176
117 ... 15136
165 ... 13802, 16177
402 ... 13803
403 ... 13803

Proposed Rules:
Ch. I ... 14014
Ch. IV ... 14014
100 ... 15144
110 ... 15145
117 ... 13838, 15146, 16188
175 ... 15606
181 ... 15606
207 ... 16046
401 ... 13838

34 CFR

4 ... 16780
205 ... 17246
630 ... 15582
631 ... 17252
632 ... 17252
633 ... 17252
634 ... 17252
635 ... 17252
655 ... 14112
656 ... 14112
657 ... 14112
658 ... 14112
660 ... 14112
683 ... 17200
730 ... 15582

35 CFR

Proposed Rules:
133 ... 16360

36 CFR

72 ... 15137
223 ... 16178

Proposed Rules:
Ch. I ... 16936
Ch. XII ... 16936
7 ... 16047, 16048, 17055

38 CFR

1 ... 16322
36 ... 15137

Proposed Rules:
21 ... 16797
36 ... 14172

39 CFR

Proposed Rules:
10 ... 14862
111 ... 14862
233 ... 14862

40 CFR

17 ... 16797
52 ... 14707, 15140, 15579-15587, 15782-15795, 16015-16018, 16324-16332, 16784
60 ... 16564, 16582
81 ... 15587, 16333
86 ... 14904, 16182, 16334
122 ... 15304
123 ... 15307, 16544, 16625, 17055
124 ... 15304
162 ... 16020
180 ... 14905-14910, 16019-16022, 17056-17058
201 ... 14709
256 ... 14910-14912, 17059
264 ... 15032, 16544
265 ... 15032, 16544

Proposed Rules:
Ch. I ... 15702, 16799
52 ... 15147, 15368, 15609, 15810-15814, 16361
65 ... 14177
81 ... 15815, 16805
86 ... 16646, 16806
122 ... 15147, 15368, 15369, 16049
123 ... 14925, 14926, 15147, 15369, 15609, 16049
124 ... 15147, 15369, 16049
146 ... 15147, 15369, 16049
180 ... 16050, 16051, 17078

41 CFR

Ch. 1 ... 16335
Ch. 7 ... 14914
Ch. 60 ... 14696
Ch. 101 ... 15141
4-1 ... 16335
8-1 ... 15332
8-3 ... 15332
8-95 ... 15333
9-15 ... 16730
9-50 ... 16730
101-43 ... 15797

Proposed Rules:
Ch. 12 ... 14014
Ch. 14 ... 16936

42 CFR

405 ... 16339
441 ... 16339

Proposed Rules:
421 ... 15370

43 CFR

1820 ... 14487

Public Land Orders:
6153 ... 14157
6230 ... 14157
6231 ... 14158
6233 ... 16626
6234 ... 16627
6235 ... 16627
6236 ... 16628
6237 ... 16628
6238 ... 17060
6239 ... 17061

Proposed Rules:
Subtitle A ... 16936
Ch. I ... 16936
Ch. II ... 16936
3130 ... 16807

44 CFR

64 ... 16023-1627
70 ... 17061-17064

Proposed Rules:
64 ... 13806
67 ... 15373, 17078-17082
302A ... 14500
2800 ... 15284
2880 ... 15286

45 CFR

302 ... 16027
303 ... 16027
400 ... 16183

Proposed Rules:
Ch. XI ... 14734
5 ... 15610

46 CFR

31 ... 15210
32 ... 15210
33 ... 15210
35 ... 15210
37 ... 15210
38 ... 15210
63 ... 15210
71 ... 15210
75 ... 15210
76 ... 15210
77 ... 15210
78 ... 15210
79 ... 15210
92 ... 15210
94 ... 15210
96 ... 15210
97 ... 15210
99 ... 15210
110 ... 15210
111 ... 15210
112 ... 15210
113 ... 15210
161 ... 15210
162 ... 16648
190 ... 15210
192 ... 15210
196 ... 15210
401 ... 13808
510 ... 16030
530 ... 14709

Proposed Rules:
Ch. I ... 14014, 15147
Ch. III ... 14014
25 ... 16648
26 ... 16648
56 ... 13838
58 ... 13838
502 ... 14734

47 CFR

0 ... 15333, 16031
1 ... 15333
2 ... 13809, 16786
22 ... 14710, 16786
67 ... 15142
73 ... 13812-13816, 15334-15336, 16629, 16630, 16786, 17065
74 ... 26786
81 ... 15333
83 ... 15333
87 ... 15333
90 ... 14489, 15337, 15588, 16789
97 ... 16789, 16790

Proposed Rules:
Ch. I ... 17083
67 ... 15374
73 ... 13839-13844, 14177, 15376-15379, 16052, 16652-16661, 16807, 16809
90 ... 16052, 16661
95 ... 14178
97 ... 14197

48 CFR

Proposed Rules:
35 ... 16189
42 ... 16189

49 CFR

1 ... 16631
171 ... 13816, 16632
173 ... 13816, 16183, 16632
178 ... 13816, 16183, 16632
192 ... 13818
630 ... 13825
1005 ... 14710
1008 ... 14710
1031 ... 16186
1047 ... 15142
1051 ... 14710

Proposed Rules:
Subtitle A ... 14014
Ch. I ... 14014
Ch. II ... 14014
Ch. III ... 14014
Ch. IV ... 14014
Ch. V ... 14014
Ch. VI ... 14014
171 ... 16286
172 ... 16286
222 ... 16189
525 ... 14501
531 ... 14501
533 ... 14501
537 ... 14501
555 ... 14501
556 ... 14501
569 ... 14501
571 ... 13845, 14501, 15612
573 ... 14501
574 ... 14501

575....................................14501
576....................................14501
577....................................14501
580....................................14501
581....................................14501

50 CFR

26....................................17066
611....................................15341
619....................................16632, 16791
640....................................15588
651....................................14158
652....................................14158
656....................................15341
657....................................15341
671....................................16339
Proposed Rules:
Ch. I....................................16936
Ch. IV....................................16936
20....................................15614, 16718
23....................................14472, 14664
83....................................14739
91....................................16810
285....................................14501, 17086

AGENCY PUBLICATION ON ASSIGNED DAYS OF THE WEEK

The following agencies have agreed to publish all documents on two assigned days of the week (Monday/Thursday or Tuesday/Friday).

This is a voluntary program. (See OFR NOTICE 41 FR 32914, August 6, 1976.)

Monday	Tuesday	Wednesday	Thursday	Friday
DOT/SECRETARY	USDA/ASCS		DOT/SECRETARY	USDA/ASCS
DOT/COAST GUARD	USDA/FNS		DOT/COAST GUARD	USDA/FNS
DOT/FAA	USDA/REA		DOT/FAA	USDA/REA
DOT/FHWA	USDA/SCS		DOT/FHWA	USDA/SCS
DOT/FRA	MSPB/OPM		DOT/FRA	MSPB/OPM
DOT/MA	LABOR		DOT/MA	LABOR
DOT/NHTSA	HHS/FDA		DOT/NHTSA	HHS/FDA
DOT/RSPA			DOT/RSPA	
DOT/SLSDC			DOT/SLSDC	
DOT/UMTA			DOT/UMTA	

Documents normally scheduled for publication on a day that will be a Federal holiday will be published the next work day following the holiday. Comments on this program are still invited.

Comments should be submitted to the Day-of-the-Week Program Coordinator, Office of the Federal Register, National Archives and Records Service, General Services Administration, Washington, D.C. 20408.

List of Public Laws

Note: No public bills which have become law were received by the Office of the Federal Register for inclusion in today's **List of Public Laws.**

Last Listing April 15, 1982
